# Nickel-catalyzed deaminative Sonogashira coupling of alkylpyridinium salts enabled by NN_2_ pincer ligand

**DOI:** 10.1038/s41467-021-25222-1

**Published:** 2021-08-12

**Authors:** Xingjie Zhang, Di Qi, Chenchen Jiao, Xiaopan Liu, Guisheng Zhang

**Affiliations:** Key Laboratory of Green Chemical Media and Reactions, Ministry of Education, Collaborative Innovation Center of Henan Province for Green Manufacturing of Fine Chemicals, Henan Key Laboratory of Organic Functional Molecules and Drug Innovation, School of Chemistry and Chemical Engineering, Henan Normal University, Xinxiang, Henan China

**Keywords:** Homogeneous catalysis, Synthetic chemistry methodology

## Abstract

Alkynes are amongst the most valuable functional groups in organic chemistry and widely used in chemical biology, pharmacy, and materials science. However, the preparation of alkyl-substituted alkynes still remains elusive. Here, we show a nickel-catalyzed deaminative Sonogashira coupling of alkylpyridinium salts. Key to the success of this coupling is the development of an easily accessible and bench-stable amide-type pincer ligand. This ligand allows naturally abundant alkyl amines as alkylating agents in Sonogashira reactions, and produces diverse alkynes in excellent yields under mild conditions. Salient merits of this chemistry include broad substrate scope and functional group tolerance, gram-scale synthesis, one-pot transformation, versatile late-stage derivatizations as well as the use of inexpensive pre-catalyst and readily available substrates. The high efficiency and strong practicability bode well for the widespread applications of this strategy in constructing functional molecules, materials, and fine chemicals.

## Abstract (web summary)

Alkynes are amongst the most valuable functional groups in organic chemistry, however, the preparation of alkyl-substituted alkynes still remains elusive. Here the authors show a nickel-catalyzed deaminative Sonogashira coupling of alkylpyridinium salts.

## Introduction

**Fig. 1:**
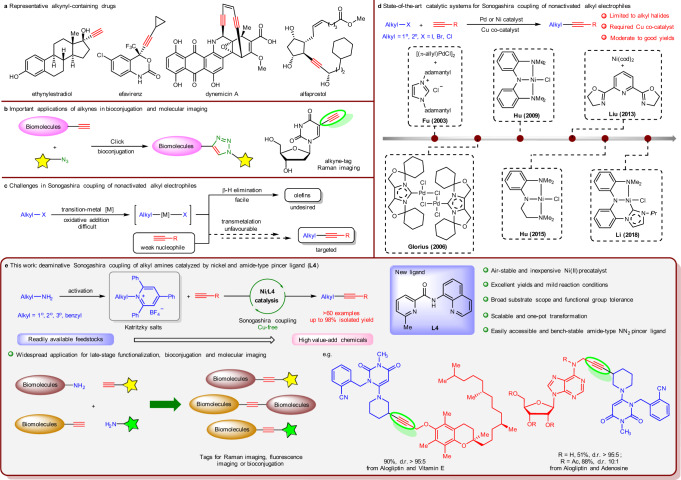
Significances and strategies for Sonogashira coupling of nonactivated alkyl electrophiles. **a** Representative alkynyl-containing drugs (ethynylestradiol, hormone drug for estrogen medication; efavirenz, an antiretroviral drug for HIV/AIDS; dynemicin A, an antitumor drug for cancer treatments; alfaprostol, a veterinary drug for breeding control). **b** Important applications of alkynes in bioconjugation and molecular imaging. **c** Challenges in Sonogashira coupling of nonactivated alkyl electrophiles. **d** State-of-the-art catalytic systems for Sonogashira coupling of nonactivated alkyl electrophiles. **e** This work: deaminative Sonogashira coupling of alkyl amines catalyzed by nickel and amide-type pincer ligand (**L4**).

Alkynes are one of the most valuable functional groups in organic chemistry because they are not only served as versatile synthetic building blocks for diversified chemical transformations, but also common structural motifs in a wide range of natural products, bioactive molecules and organic materials^1–3^. For example, the introduction of an alkyne into a drug molecule could provide remarkable benefits in its biological activity, such as enhanced lipophilicity, bioavailability, and metabolic stability (Fig. 1a). In addition to the widely used as functional tags in biochemistry for bioconjugation based on “alkyne-azide click chemistry”^4^, recent researches also indicated that alkynes have a privileged application in Raman imaging due to their unique and strong Raman scattering peaks in a cellular silent region that is free of interference from most endogenous molecules (Fig. 1b)^5–9^. Therefore, lots of efforts have been made to develop efficient methods for the construction of alkynes. Among these available transformations, the transition-metal-catalyzed Sonogashira coupling of aryl/vinyl electrophiles with terminal alkynes has proven to be one of the most powerful approaches for C(*sp*^2^)–C(*sp*) bond formation^10,11^. However, the incorporation of nonactivated, β-H-containing alkyl electrophiles in Sonogashira reaction to construct C(*sp*^3^)–C(*sp*) bond still remains a formidable challenge, presumably due to the following issues (Fig. 1c): (1) the reluctance of alkyl electrophiles to undergo oxidative addition with a metal catalyst, (2) the propensity of the resulting alkylmetal intermediates to undergo intramolecular β-hydride elimination, (3) the poor nucleophilicity of the *sp*- hybridized carbon in alkynes, and (4) the low concentration of the transmetalating species generated in situ in the reaction medium. Moreover, the facile cyclotrimerization and/or oligomerization of terminal alkynes under the catalysis of low-valent metal is another obstacle that renders such coupling a more intractable objective^12,13^. In a pioneering study, Fu and co-workers realized Pd/Cu-cocatalyzed Sonogashira coupling of nonactivated primary alkyl iodides and bromides by the use of an N-heterocyclic carbene (NHC) ligand^14^. Later on, a few elegant strategies for this transformation were developed based on the discovery of different catalytic systems including Pd/bisoxazoline-derived NHC ligand^15^, Ni/NN_2_ pincer ligand^16,17^, Ni/pyridine bisoxazoline (pybox) system^18^, and NHC pincer nickel(II) complex^19^ (Fig. 1d). Furthermore, Lalic et al. discovered an excellent protocol of photoinduced copper-catalyzed alkylation of terminal alkyne with alkyl iodides as the alkyl source^20^. More recently, Wang and co-workers further expanded the scope of alkyl to the cheap and easily accessible carboxylic acid derivatives by the utilization of redox-active esters^21^. Despite these significant advances, the scope for alkyl-Sonogashira-type reactions is still relatively limited. Particularly, the electrophilic partners in such reactions are largely limited to alkyl halides^22^, and the need of copper(I) salt as cocatalyst might also cause some detrimental effects to the reaction, such as the undesired Glaser coupling of terminal alkynes and the complicated procedure in workup^23^. Thus, developing simple approaches to access such coupling with more alternatives especially in copper-free conditions is highly important and appealing.

Alkyl amines are naturally abundant and readily available feedstock chemicals, and the prevalence of amino groups in numerous bioactive molecules, pharmaceuticals, and natural products provide expedient opportunities for late-stage functionalization and bioconjugation^24,25^. In this context, using alkyl amines as alkylating agents in organic synthesis would have many privileged advantages when compared to the traditional platforms using alkyl halides. However, such a promising transformation is still underexploited owing to the high bond dissociation energy of C(*sp*^3^)–N bond^26–28^. In a seminal work, Watson et al. demonstrated that pyridinium salts^29^, also known as “Katritzky salts” which are easily formed from primary amines and pyrylium salt, could be used as alkyl radical precursors in cross-coupling with arylboronic acids^30^. Since then, many elegant approaches based on the utilization of these redox-active amines for deaminative functionalization^31–34^, such as arylation^35–40^, borylation^41–43^, alkenylation^44–46^, allylation^47^, alkyl-Heck-type reaction^48,49^, carbonylation^50–54^, alkylation^55–58^, difluoromethylation^59^, and C-heteroatom bond-forming reactions^60–62^ have been established. However, the deaminative alkynylation of alkyl amines to form the C(*sp*^3^)–C(*sp*) bond still remains elusive. Recently, the Gryko group developed a nice protocol to access such transformation by visible-light-mediated desulfonylative alkynylation of secondary alkyl- and benzylpyridinium salts with alkynyl sulfones^63^. Han and co-workers reported an efficient nickel-catalyzed reductive cross-electrophile coupling of Katritzky salts with triisopropylsilyl (TIPS)-substituted bromoethyne to achieve the challenging C(*sp*^3^)–C(*sp*) bond^64^. Nevertheless, these methods rely mainly upon the use of preformed and activated alkynyl sulfones or bromides as alkynylating reagents. In addition, the limited substrate scope and the utilization of largely excess reductants (e.g. zinc flake) further disfavored their wide applications in organic synthesis. Therefore, the direct coupling of terminal alkynes with alkylpyridinium salts in a redox-neutral fashion for the synthesis of important alkynes would be highly desirable in terms of both atom-economy and practical application. To the best of our knowledge, however, such a straightforward and practical protocol has not been achieved.

Following our keen interest in nickel-catalyzed cross-coupling reactions^65,66^, herein, we report the general and efficient nickel-catalyzed Sonogashira coupling of alkylpyridinium salts via C–N bond activation under Cu-free conditions (Fig. 1e). The easily accessible and bench-stable amide-type NN_2_ pincer ligand (6-methyl-*N*-(quinolin-8-yl)picolinamide **L4**) is found to be crucial for this transformation, allowing the coupling to occur under mild reaction conditions with excellent yields and high functional group tolerance.

## Results

### Optimization study

Initially, the coupling of phenethylpyridinium salt **1a** and phenylacetylene **2a** was selected as the model reaction for optimization (Table 1). To realize such transformation, we envisaged that a pincer ligand might be feasible due to its strong and tridentate bonding mode to the metal center, thereby possibly stabilizing the alkylnickel intermediate^67^ and suppressing the undesired Glaser coupling^68,69^. Thus, the ligands were firstly screened by using 10 mol% NiCl_2_(glyme) as a catalyst, K_3_PO_4_ as a base in tetrahydrofuran (THF) at 80 °C. When pybox, the most efficient ligand in Liu’s work^18^, was applied to this reaction, the desired product **3a** was obtained in 4% yield and the main product was 1,4-diphenylbutadiyne derived from the homocoupling of **2a** (entry 1). While the use of a more electron-rich and bulky 4,4′,4″-tri-*tert*-butyl terpyridine (ttbtpy) in this process, the yield of **3a** was improved to 53% (entry 2). Much to our delight, the yields of **3a** could be further improved to 87% and 83%, respectively, when amide-type pincer ligand (e.g. *N*-(pyridin-2-ylmethyl)picolinamide (**L1**) or *N*-(quinolin-8-yl)picolinamide (**L2**)) was used (entries 3–4), though they were seldom used as ligands in transition metal-catalyzed cross-coupling reactions^70–73^. This discovery encouraged us to synthesize two sterically more hindered methylated derivatives **L3** and **L4** as ligands. Gratifyingly, the yield was significantly improved to 96% by employing **L4** (entry 6). The reasons for the high efficiency of **L4** are still unclear at present but probably related to its steric hindrance and rigidity. Screening of nickel catalysts revealed that Ni(acac)_2_ was ineffective (entry 7), whereas the inexpensive, air-stable and moisture-stable NiCl_2_·6H_2_O gave the best result (entry 8). Subsequently, the effect of the base was examined. K_2_CO_3_ resulted in a slightly diminished yield (entry 9). However, the reaction completely shut down by using Et_3_N, a frequently used base in palladium-catalyzed Sonogashira coupling of aryl halides (entry 10)^11^. Lowering the amount of catalyst or reaction temperature led to a reduced yield to different extent (entries 11–12). Control experiments indicated that NiCl_2_·6H_2_O, **L4,** and K_3_PO_4_ were all essential for achieving the transformation (entries 13–15) (For a detailed optimization study, see Supplementary Tables 1–5).

**Table 1:** Optimization of the reaction conditions^a^.


Entry	Catalyst	Ligand	Base	Yield (%)^b^
1	NiCl_2_(glyme)	pybox	K_3_PO_4_	4
2	NiCl_2_(glyme)	ttbtpy	K_3_PO_4_	53
3	NiCl_2_(glyme)	**L1**	K_3_PO_4_	87
4	NiCl_2_(glyme)	**L2**	K_3_PO_4_	83
5	NiCl_2_(glyme)	**L3**	K_3_PO_4_	40
6	NiCl_2_(glyme)	**L4**	K_3_PO_4_	96
7	Ni(acac)_2_	**L4**	K_3_PO_4_	7
8	NiCl_2_·6H_2_O	**L4**	K_3_PO_4_	99 (97)^c^
9	NiCl_2_·6H_2_O	**L4**	K_2_CO_3_	95
10	NiCl_2_·6H_2_O	**L4**	Et_3_N	0
11^d^	NiCl_2_·6H_2_O	**L4**	K_3_PO_4_	91
12^e^	NiCl_2_·6H_2_O	**L4**	K_3_PO_4_	68
13	–	**L4**	K_3_PO_4_	0
14	NiCl_2_·6H_2_O	–	K_3_PO_4_	0
15	NiCl_2_·6H_2_O	**L4**	–	0

*pybox* pyridine bisoxazoline, *ttbtpy* 4,4′,4″-tri-*tert*-butyl terpyridine.

^a^Conditions: **1a** (0.3 mmol), **2a** (0.45 mmol), catalyst (10 mol%), ligand (10 mol%), base (1.3 equiv), THF (1.5 mL), 80 °C.

^b^Determined by ^1^H NMR using 1,3,5-trimethoxybenzene as an internal standard.

^c^Isolated yield.

^d^5 mol% NiCl_2_·6H_2_O and **L4** were used.

^e^Reaction was conducted at 60 °C.

### Substrate scope

**Fig. 2:**
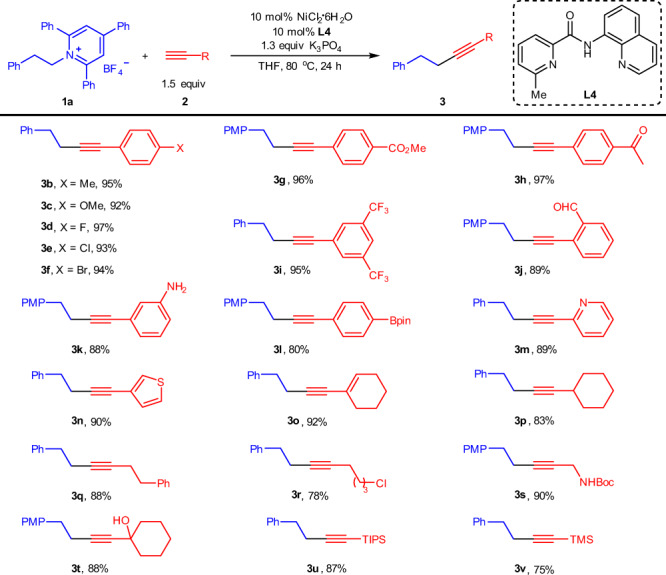
Scope of terminal alkynes. Reaction conditions: **1a** (0.3 mmol), **2** (0.45 mmol), NiCl_2_·6H_2_O (10 mol%), **L4** (10 mol%), K_3_PO_4_ (1.3 equiv), THF (1.5 mL), 80 °C. Isolated yields. For **3g**, **3h**, **3j**, **3k**, **3l**, **3s** and **3t**, reactions were conducted using *p*-methoxyphenethylpyridinium salt **1b** instead of **1a**. PMP *p*-methoxyphenyl.

With the optimized coupling conditions in hand, the scope of alkynes was first evaluated using **1a** as the coupling partner. For some cases that the products were unseparated from the excess terminal alkynes, *p*-methoxylphenethylpyridinium salt **1b** was used instead of **1a**. As shown in Fig. 2, the arynes bearing both electron-donating and electron-withdrawing groups could participate in this transformation delivering the products (**3b–3k**) in excellent yields. Various synthetically important functional groups including methoxyl, arylhalide, ester, acetyl, trifluoromethyl, formyl, and free amino were all perfectly accommodated. Particularly noteworthy was that aryl chlorides and bromides, popular electrophilic partners in Sonogashira reactions^10^, remained inert under our optimized reaction conditions, highlighting the exquisite chemoselectivity of this transformation. Additionally, the presence of an ortho formyl did not hamper the reaction. Strikingly, terminal alkyne (**2l**) containing a boronate ester group was also successfully engaged in this transformation with its C–B bond intact, thus allowing for further diversification. Heteroaromatic rings such as pyridine and thiophene might deactivate a metal catalyst by coordination, and 1-ethynylcyclohexene could also smoothly undergo the transformation giving the corresponding products (**3m–3o**) in excellent yields. More importantly, aliphatic alkynes (**2p–2t**) could also be coupled in high efficiency. The functional groups such as Cl, NHBoc, and OH were well tolerated, affording the products (**3r–3t**) in high to excellent yields with excellent selectivity. Finally, TIPS- and trimethylsilyl-capped alkynes were also suitable substrates to obtain the products (**3u–3v**) in high yields.

**Fig. 3:**
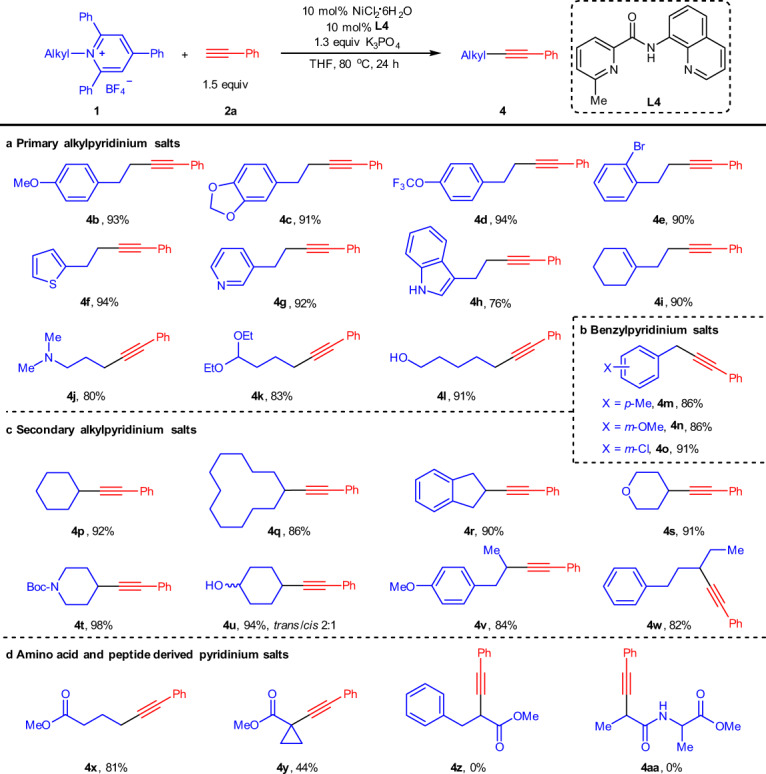
Scope of alkyl amines. **a** Scope of primary alkylpyridinium salts. **b** Scope of benzylpyridinium salts. **c** Scope of secondary alkylpyridinium salts. **d** Scope of amino acid and peptide-derived pyridinium salts. Reaction conditions: **1** (0.3 mmol), **2a** (0.45 mmol), NiCl_2_·6H_2_O (10 mol%), **L4** (10 mol%), K_3_PO_4_ (1.3 equiv), THF (1.5 mL), 80 °C. Isolated yields. For **4g**, **4h**, **4p**, **4q**, **4r**, **4s**, **4t**, **4u**, **4v**, **4w**, **4y**, **4z**, and **4aa**, reactions were conducted in DMF (1.5 mL). For **4m**, **4n**, and **4o**, reactions were conducted at 50 °C.

Next, the generality of alkyl amines was evaluated as shown in Fig. 3. Various primary alkyl (**1b–1l**) and benzylpyridinium salts (**1m–1o**) were all suitable substrates for this transformation, and the desired products (**4b–4o**) could be obtained in high to excellent yields. However, the secondary alkylpyridinium salts (e.g. **1t**) exhibited a dramatic drop in reaction efficiency (**4t**, 74%) under the optimized conditions. Then reoptimization of secondary alkylpyridinium salts was conducted by exploring various reaction parameters. Gratifyingly, 98% yield of **4t** could be obtained by changing the solvent to DMF. Under the slightly modified conditions, diverse secondary alkylpyridinium salts underwent this coupling smoothly to give the desired products (**4p–4w**) in high to excellent yields. Similarly, good functional group tolerance was observed, as exemplified by the well compatible with methoxyl, trifluoromethoxyl, bromide, indole NH, alkenyl, *tert*-amine, acetal, hydroxyl, and chloride. More importantly, heterocyclic units such as thiophene (**1f**), pyridine (**1g**), indole (**1h**), tetrahydropyran (**1s**), and piperidine (**1t**) which are prevalent in medicinally relevant molecules were competent substrates. In addition, benzylpyridinium salts especially electron-rich benzylic salts which are not suitable in Gryko’s work^63^ could be coupled with high efficiency (**4m–4n**), emphasizing the robustness of our strategy in synthetic applications. It is worth noting that both cyclic (**1p–1u**) and acyclic secondary amines (**1v**–**1w**) could be readily applied to this protocol with high to excellent yields. Moreover, *γ*-amino acid-derived pyridinium salt (**1x**) proceeded well under the standard conditions. Notably, a quaternary carbon center could be successfully constructed by using a tertiary amine derivative (**1y**), albeit in a 44% yield. However, when phenylalanine (**1z**) and dipeptide (**1aa**) were employed in this reaction, complex products distributions were observed, and none of the desired deaminative alkynylation products were obtained.

**Fig. 4:**
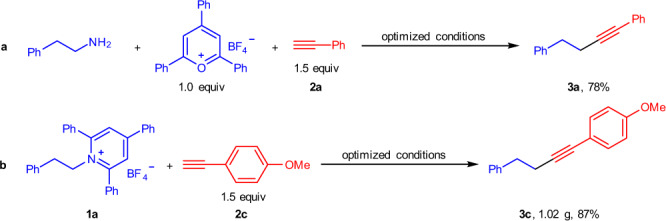
Further applications. **a** One-pot transformation. **b** Gram-scale study.

It is worth highlighting that this protocol was amenable to a one-pot transformation in which pyrylium salt, alkyl amine and the cross-coupling reagents were added simultaneously in a single step, and 78% yield of the product **3a** could be obtained without further reoptimizing the reaction conditions (Fig. 4a). Additionally, a gram-scale reaction was successfully performed using **1a** and **2c** under the optimized conditions producing **3c** in 87% yield, exemplifying the practicability and scalability of this process (Fig. 4b).

### Late-stage derivatizations

**Fig. 5:**
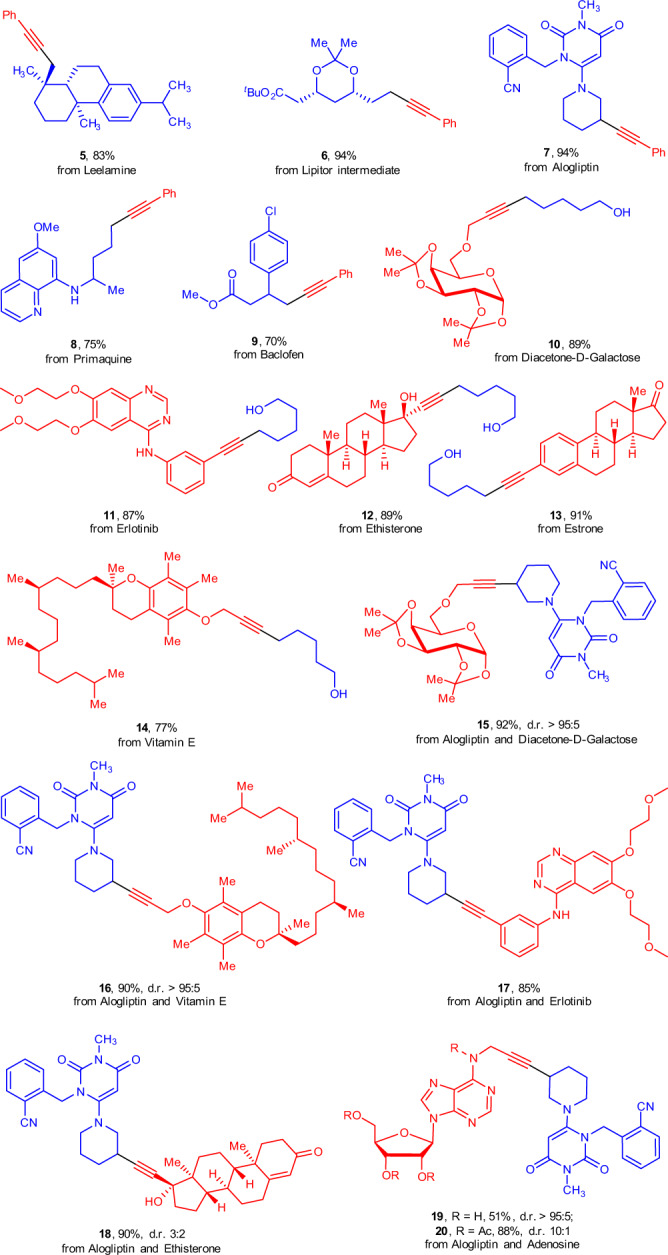
Late-stage modification of natural products and medicinally relevant molecules. Reaction conditions: pyridinium salt (0.3 mmol), alkyne (0.45 mmol), NiCl_2_·6H_2_O (10 mol%), **L4** (10 mol%), K_3_PO_4_ (1.3 equiv), THF (1.5 mL), 80 ^o^C. Isolated yields. For **7**, **9**, **15**, **16**, **17**, **18**, **19** and **20**, reactions were conducted in DMF (1.5 mL).

To further demonstrate the broad applicability of this method, late-stage functionalization of natural products and medicinally relevant molecules were conducted (Fig. 5). A series of pyridinium salts and alkynes derived from drugs and bioactive compounds underwent this transformation with good to excellent yields (**5–20**). This general protocol could be successfully applied for the rapid construction of alkyne-labeled derivatives of biomolecules (**5–9**). The readily attached alkynyl group is expected to serve as a labeling tool to facilitate further chemical biology studies and as a handle for rapid entry to complex derivatives. Likewise, this versatile method can be also applied in the further functionalization of alkynyl-containing bioactive molecules or intermediates (**10–14**). Notably, the virtues of the current method were further illustrated by the successful coupling of two drug molecules for assembling their drug-like hybrids **15–20**, highlighting the potential applications of this chemistry in the discovery of pharmaceutical candidates.

### Mechanistic studies

**Fig. 6:**
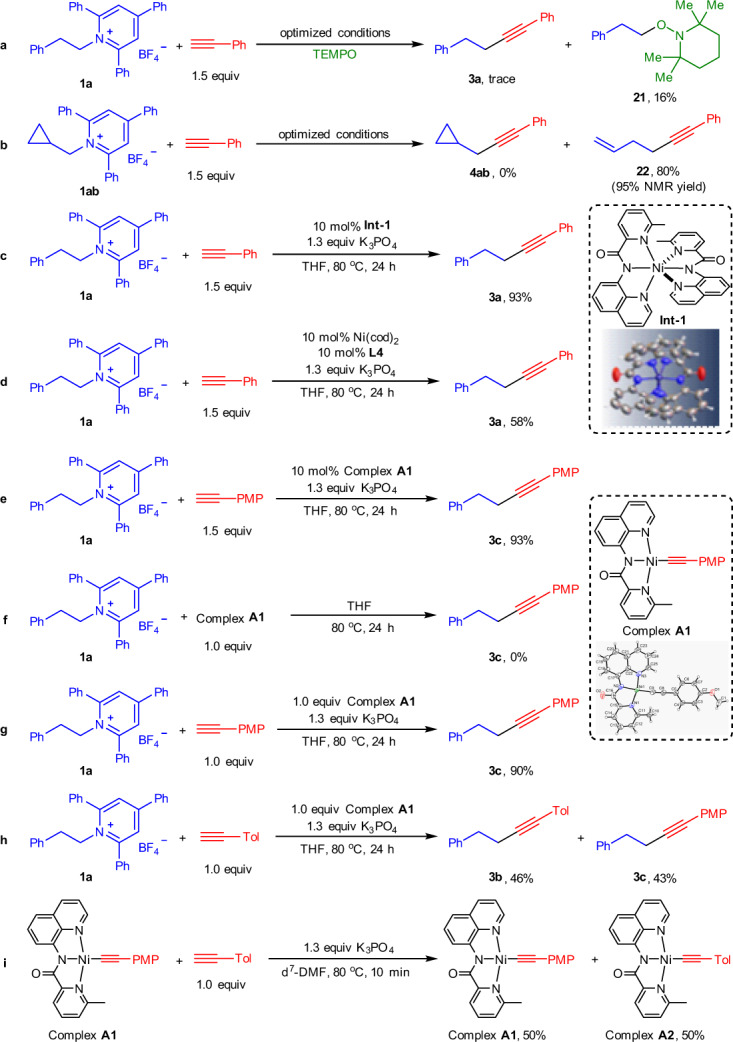
Preliminary mechanistic studies. **a** Radical trap experiment. **b** Radical clock experiment. **c** Catalytic transformation using **Int-1** as catalyst. **d** Catalytic transformation using Ni(cod)_2_ as catalyst. **e** Catalytic transformation using complex **A1** as catalyst. **f** Stoichiometric reaction of complex **A1**. **g** Stoichiometric reaction of complex **A1** in the presence of terminal alkyne and base. **h** Crossover experiment of complex **A1**. **i** Alkyne exchange experiment of complex **A1**. PMP *p*-methoxyphenyl, Tol *p*-methylphenyl.

To understand the reaction mechanism, a series of experiments were performed. When the radical trapping reagent TEMPO was added to the reaction mixture, the only a trace of **3a** was obtained with the concurrent formation of TEMPO-adduct **21** in 16% yield (Fig. 6a). In addition, a radical-clock experiment was also conducted by employing cyclopropylmethyl pyridinium salt **1ab**. Instead of normal cross-coupling product **4ab**, a ring-opened product **22** was achieved in high yield (Fig. 6b). These results suggest that an alkyl radical may be involved in this transformation. To further elucidate the role of the nickel catalyst in this reaction, a Ni complex **Int-1** was synthesized by simple exposure of NiCl_2_·6H_2_O and **L4** in THF at room temperature and characterized by X-ray crystallography. Gratifyingly, a high yield of **3a** was obtained when **Int-1** was applied to this catalytic transformation (Fig. 6c). However, when Ni(cod)_2_ was used as the catalyst, a remarkable decrease in efficiency was observed and only a moderate yield of **3a** was achieved (Fig. 6d). These results indicate that the six-coordinate Ni complex **Int-1** also exhibits an excellent catalytic activity, while a Ni(0) species is not likely involved in this chemistry. To gain more insight into the mechanism of this reaction, a Ni-alkynyl complex **A1** was formed by the reaction of NiCl_2_(glyme), **L4,** and K_3_PO_4_ with *p*-methoxyphenylethyne in DMF, and its structure was confirmed by X-ray diffraction. Employing 10 mol% complex **A1** as the catalyst, the reaction of **1a** with *p*-methoxyphenylethyne delivered the product **3c** in 93% yield, which was similar to the result obtained using NiCl_2_·6H_2_O and **L4** as the catalyst (Fig. 6e). However, the stoichiometric reaction of complex **A1** with **1a** did not give **3c** in any detectable yield (Fig. 6f). Interestingly, when this reaction was conducted in the presence of 1.0 equiv of *p*-methoxyphenylethyne and 1.3 equiv of K_3_PO_4_, an almost identical yield of **3c** to that of the catalysis could be obtained (Fig. 6g). These results show that complex **A1** itself could not react with alkyl pyridinium salt, and it needs to be activated by an additional alkynyl anion before reaction with alkyl pyridinium salt. To probe the role of alkyne on the activation of complex **A1**, a crossover experiment between complex **A1** and *p*-methylphenylacetylene with **1a** was performed in the presence of K_3_PO_4_. Surprisingly, both **3b** and **3c** were achieved in comparable yields, with an overall yield of 89% (Fig. 6h). This result implies that the two alkynyl fragments in the active Ni-species are equivalent and/or exchangeable. To further investigate whether a fast alkynyl exchange process occurred on the complex **A1**, the reaction of complex **A1** with equal amounts of *p*-methylphenylacetylene was carried out under catalytically relevant conditions (Fig. 6i). A nickel complex **A2** was immediately observed by NMR analysis of the reaction mixture (For details, see Supplementary Fig. 1). Then it quickly reached an equilibrium with complex **A1** in a roughly 1:1 ratio, which means that the alkynyl ligated on complex **A1** is exchangeable. Although the real active species for this deaminative alkynylation reaction is still unclear at present, it might be tentatively assigned to the Ni bis(acetylide) intermediate considering the outcomes achieved in Fig. 6e–i. Moreover, similar results were also observed by Hu et al. ^17^ further supporting the possibility of Ni bis(acetylide) intermediate as the active species for this coupling reaction.

## Discussion

**Fig. 7:**
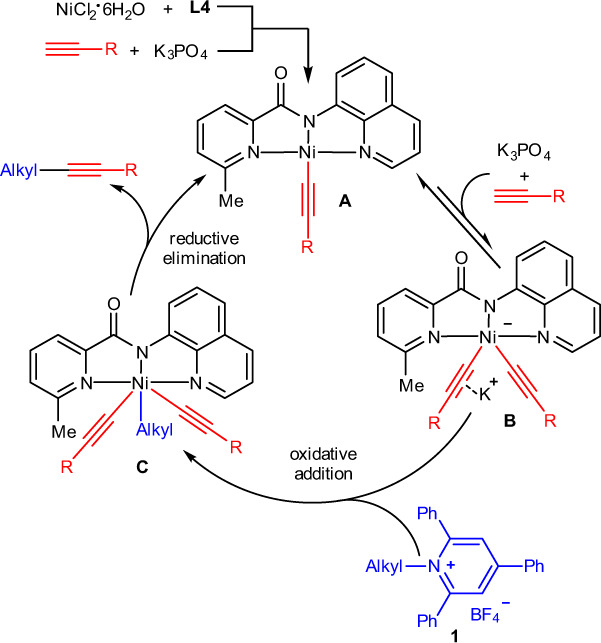
Proposed reaction mechanism for the nickel-catalyzed deaminative Sonogashira coupling of alkylpyridinium salts. A plausible mechanism involving a more electron-rich Ni bis(acetylide) species is tentatively proposed.

Although a detailed mechanism awaits further studies, a plausible mechanism is depicted in Fig. 7 based on the above results and Ni/pincer-ligand system catalyzed cross-coupling of alkyl electrophiles^16,17,19,74–77^. Initially, coordination of **L4** to the Ni center followed by base promoted transmetalation with terminal alkyne to form a complex **A**. However, this species possesses no reactivity toward pyridinium **1** as demonstrated by Fig. 6f. Moreover, the cyclic voltammogram of complex **A1** showing an oxidation wave at 1.19 V in DMF further indicates that the direct coupling of complex **A** with **1** (*E*_red_ = −0.90 V vs. SCE in DMF) is not possible. (For cyclic voltammogram of pyridinium **1a** and complex **A1**, see Supplementary Figs. 2 and 3.) Thus, a further transmetalation of complex **A** with alkyne was needed to generate a more electron-rich anionic species **B**, which is thermodynamically unstable and could rapidly disassociate an alkynyl anionic to reach an equilibrium with the dormant complex **A**. At this stage, the K(I) ion in the Ni bis(acetylide) intermediate is probably coordinated to the triple bond of alkyne, similar to that of binding a copper reported by Hartwig^78^. Then, the more active species **B** might undergo oxidative addition with **1** to give intermediate **C**, during which a radical process is likely involved based on the results obtained from Fig. 6a, b. Finally, reductive elimination from **C** delivers the C(*sp*^3^)–C(*sp*) coupling product and regenerates complex **A** for the next catalytic cycle. The reasons for the high selectivity of cross-coupling products are unclear at now, but probably related to the fast alkyl–alkynyl reductive elimination promoted by the NN_2_ pincer ligand^17,76^. Additionally, the oxidation state of Ni in intermediate **C** seems to be a Ni^IV^, but it might also be described as a Ni^III^–ligand radical complex when considering the redox-active of NN_2_ pincer ligand^75,79^. Therefore, the current catalytic cycle is not in contradiction with the proposed mechanism in Ni catalysis.

In summary, we have achieved a highly efficient and general Sonogashira coupling of alkylpyridinium salts by the development of a Ni/NN_2_ pincer ligand catalytic system. Noteworthy was the realization of the coupling of terminal alkynes with naturally abundant alkyl amines, expanding the substrate scopes used in Sonogashira reaction. The virtues of this reaction are illustrated by the broad substrate scope, well functional group tolerance in both coupling partners as well as the efficient diversification of natural products and medicinally relevant molecules. Further mechanism investigation and application of this catalytic system for the cross-coupling with other electrophiles are currently ongoing in our laboratories.

## Methods

### General procedure 1

In a nitrogen-filled glovebox, NiCl_2_·6H_2_O (0.03 mmol, 7.1 mg), **L4** (0.03 mmol, 7.9 mg), anhydrous K_3_PO_4_ (0.39 mmol, 82.8 mg), primary alkylpyridinium salt (0.3 mmol), and THF (1.5 mL) were successively added to an oven-dried sealable Schlenk tube (10.0 mL) followed by addition of terminal alkyne (0.45 mmol) via microliter syringe *(If terminal alkyne is solid, it was added before the solvent)*. Then the tube was securely sealed and taken outside the glovebox. And it was immersed into an oil bath preheated at 80 or 50 °C. After stirring for 24 h, the reaction mixture was cooled to room temperature and filtered through a short pad of silica gel. Then the filter cake was washed with dichloromethane or ethyl acetate. The resulting solution was concentrated under vacuum and the residue was purified by column chromatography on silica gel to afford the corresponding product.

### General procedure 2

In a nitrogen-filled glovebox, NiCl_2_·6H_2_O (0.03 mmol, 7.1 mg), **L4** (0.03 mmol, 7.9 mg), anhydrous K_3_PO_4_ (0.39 mmol, 82.8 mg), secondary alkylpyridinium salt (0.3 mmol), and *N,N*-dimethylformamide (1.5 mL) were successively added to an oven-dried sealable Schlenk tube (10.0 mL) followed by addition of phenylacetylene (0.45 mmol, 46.0 mg) via microliter syringe. Then the tube was securely sealed and taken outside the glovebox. And it was immersed into an oil bath preheated at 80 °C. After stirring for 24 h, the reaction mixture was cooled to room temperature and quenched with water. Then it was extracted with ethyl acetate or diethyl ether, washed with water and brine, and dried over anhydrous Na_2_SO_4_. The resulting solution was concentrated under vacuum and the residue was purified by column chromatography on silica gel to afford the corresponding product.

## Supplementary information

Supplementary Information

## Data Availability

Detailed experimental procedures and characterization of all new compounds can be found in the Supplementary Information. The authors declare that all the data supporting the findings of this study are available within the article and Supplementary Information files, and are also available from the corresponding authors upon reasonable request. CCDC 2035475 (**Int-1**) and 2055846 (complex **A1**) contain the supplementary crystallographic data for this paper. These data can be obtained free of charge from The Cambridge Crystallographic Data Centre via www.ccdc.cam.ac.uk/data_request/cif.
