# Supplementary material for: Nickel-catalyzed deaminative Sonogashira coupling of alkylpyridinium salts enabled by NN2 pincer ligand
Source: Nat Commun. 2021 Aug 12;12:4904. doi: 10.1038/s41467-021-25222-1 (PMC8361081; doi:10.1038/s41467-021-25222-1)

Supplementary Information for
Nickel-catalyzed deaminative Sonogashira coupling of alkylpyridinium salts enabled by NN_2 pincer ligand

Xingjie Zhang[1,*], Di Qi[1,2], Chenchen Jiao[1,2], Xiaopan Liu[1] & Guisheng Zhang[1,*]

[1]Key Laboratory of Green Chemical Media and Reactions, Ministry of Education, Collaborative Innovation Center of Henan Province for Green Manufacturing of Fine Chemicals, Henan Key Laboratory of Organic Functional Molecule and Drug Innovation, School of Chemistry and Chemical Engineering, Henan Normal University, Xinxiang, Henan 453007, China. [2]These authors contributed equally: Di Qi, Chenchen Jiao. Corresponding authors. E-mail: zhangxingjie@htu.edu.cn; zgs@htu.cn.

Table of Contents

Supplementary note 1	S3
Supplementary methods	S4
General procedure for optimization studies	S4-S6
General procedure A: synthesis of amide-type NN_2 pincer ligand	S6-S8
Synthesis of alkylpyridinium salts	S8-S18
Synthesis of terminal alkynes	S18
Nickel-catalyzed deaminative Sonogashira coupling of alkylpyridinium salts	S19
General procedure B: Sonogashira coupling of **1a** with alkynes	S19-S31
General procedure C: Sonogashira coupling of primary alkylpyridinium salts	S32-S40
General procedure D: Sonogashira coupling of secondary alkylpyridinium salts	S40-S46
Late-stage modification of natural products and medicinally relevant molecules	S47-S61
Gram scale study	S61
One-pot transformation	S61-S62
Supplementary discussion	S62
Mechanistic studies	S62
Radical trap experiment	S62-S63
Radical clock experiment	S63-S64
Synthesis of Ni complex **Int-1**	S64
Ni complex **Int-1** catalyzed deaminative Sonogashira coupling of **1a**	S65
$Ni(cod)_2$ catalyzed deaminative Sonogashira coupling of **1a**	S65-S66
Synthesis of complex **A1**	S66
Complex **A1** catalyzed deaminative Sonogashira coupling of **1a**	S67
Stoichiometric reaction of complex **A1** with **1a**	S67-S68
Stoichiometric reaction of complex **A1** with **1a** in the presence of alkyne and base	S68
Crossover experiment of complex **A1** and *p*-methylphenylacetylene with **1a**	S68-S69
Synthesis of complex **A2**	S69-S70
Alkyne exchange experiment between complex **A1** and *p*-methylphenylacetylene	S70-S71
Cyclic voltammetry studies	S71-S73
Supplementary references	S73-S74

X-ray crystal structure of Ni complex **Int-1** and complex **A1** S75
NMR spectra of all new compounds S76-S252

Supplementary Note 1

Unless otherwise noted, commercially available reagents were used as received without further purification and all reactions were carried out using standard Schlenk technique or a dry box technique under a nitrogen atmosphere. Tetrahydrofuran (THF), dimethyl sulfoxide (DMSO) and acetonitrile (CH_3CN) were dried using Eminex Solvent Purifier (EX-SPS5-800). Toluene was distilled from sodium and benzophenone. Anhydrous *N, N*-dimethylformamide (DMF) and 1-methyl-2-pyrrolidinone (NMP) were purchased from J&K Chemical Company. $NiCl_2·6H_2O$, $Ni(OAc)_2·4H_2O$ and $Ni(acac)_2$ were purchased from J&K Chemical Company. NiI_2 was purchased from Alfa Aesar. $NiCl_2$(glyme) and $NiBr_2$(glyme) were purchased from Sigma-Aldrich. Anhydrous K_3PO_4 was purchased from Acros. Flash column chromatography was performed on silica gel (200-300 mesh). ^1H and ^{13}C NMR spectra were recorded on Bruker 400 MHz and 600 MHz spectrometers at room temperature in $CDCl_3$, DMSO-d_6, CD_3OD, C_6D_6 or DMF-d_7 (containing 0.03% TMS) solutions. ^1H NMR spectra was recorded with tetramethylsilane (0.00 ppm) or solvent residual peak ($CDCl_3$: 7.26 ppm; DMSO-d_6: 2.50 ppm; CD_3OD: 3.31 ppm; C_6D_6: 7.16 ppm) as internal reference; ^{13}C NMR spectra was recorded with $CDCl_3$ (77.00 ppm) or DMSO-d_6 (39.52 ppm) as internal reference. Data are represented as follows: chemical shift, multiplicity (br = broad, s = singlet, d = doublet, t = triplet, q = quartet, dd = doublet of doublets, m = multiplet), coupling constants (Hz) and integration. High-resolution mass spectra were obtained by using Bruker UHR-ES-TOF MS, Bruker autoflex maX MALDI-TOF, Waters Premier GC-TOF MS, JEOL-AccuTOF-GCv4G-GCT MS or Thermo Scientific Q Exactive HF Orbitrap-FTMS. The IR spectra were measured on a PerkinElmer Spectrum 400 FT-IR/FT-FIR spectrometer. Single crystal X-ray diffraction data was collected at 295 K for Ni complex **Int-1** and at 298 K for complex **A1** on a SuperNova diffractometer. 2,4,6-Triphenylpyrylium tetrafluoroborate[1] was prepared according to literature procedures.

Supplementary Methods

General procedure for optimization studies

In a nitrogen-filled glovebox, nickel catalyst (0.03 mmol), ligand (0.03 mmol), base (0.39 mmol), phenethylpyridinium salt **1a** (0.3 mmol, 150.0 mg) and solvent (1.5 mL) were successively added to an oven-dried sealable Schlenk tube (10.0 mL) followed by addition of phenylacetylene (0.45 mmol, 46.0 mg) via microliter syringe. Then the tube was securely sealed and taken outside the glovebox. And it was immersed into an oil bath preheated at 80 °C. After stirring for 24 h, the reaction mixture was cooled to room temperature and filtered through a short pad of silica gel. Then the filter cake was washed with dichloromethane. The resulting solution was concentrated under vacuum and the residue was dissolved in $CDCl_3$. The NMR yields were obtained by ^{1}H NMR analysis of the crude mixture using 1,3,5-trimethoxybenzene (0.3 mmol, 50.5 mg) as an internal standard.

Supplementary Table 1. Effect of ligand

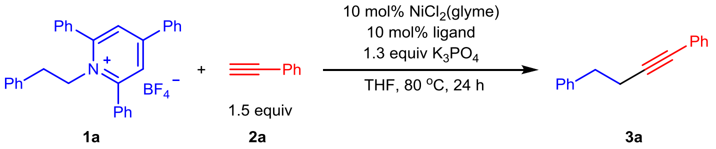

entry	ligand	yield (%)^b
1	bipy	4
2	phen	6
3	pybox	4
4	terpy	9
5	ttbtpy	53
6	**L1**	87
7	**L2**	40
8	**L3**	83
9	**L4**	96

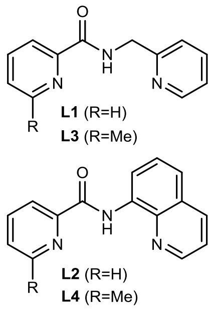

^{a}Conditions: **1a** (0.3 mmol), **2a** (0.45 mmol), $NiCl_2$(glyme) (10 mol%), ligand (10 mol%), K_3PO_4 (1.3 equiv), THF (1.5 mL), 80 °C. ^{b}Yields determined by ^{1}H NMR using 1,3,5-trimethoxybenzene as an internal standard.

Supplementary Table 2. Effect of nickel source

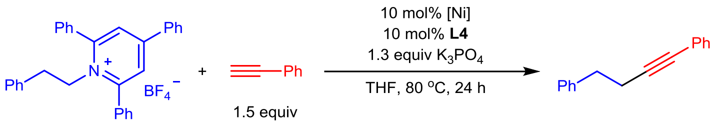

1a **2a** **3a**

entry	[Ni]	yield (%)[b]
1	$NiCl_2$(glyme)	96
2	$NiBr_2$(glyme)	92
3	NiI_2	95
4	$Ni(acac)_2$	7
5	$Ni(OAc)_2 \cdot 4H_2O$	49
6	$NiCl_2 \cdot 6H_2O$	99 (97)[c]

[a]Conditions: **1a** (0.3 mmol), **2a** (0.45 mmol), [Ni] (10 mol%), **L4** (10 mol%), K_3PO_4 (1.3 equiv), THF (1.5 mL), 80 °C. [b]Yields determined by ^{1}H NMR using 1,3,5-trimethoxybenzene as an internal standard. [c]Isolated yield.

Supplementary Table 3. Effect of solvent

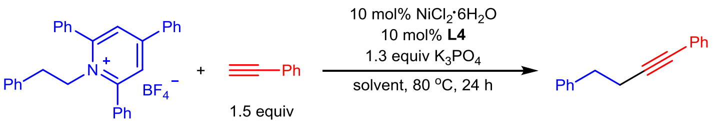

1a **2a** **3a**

entry	solvent	yield (%)[b]
1	THF	99 (97)[c]
2	CH_3CN	67
3	DMF	90
4	DMSO	85
5	NMP	88

[a]Conditions: **1a** (0.3 mmol), **2a** (0.45 mmol), $NiCl_2 \cdot 6H_2O$ (10 mol%), **L4** (10 mol%), K_3PO_4 (1.3 equiv), solvent (1.5 mL), 80 °C. [b]Yields determined by ^{1}H NMR using 1,3,5-trimethoxybenzene as an internal standard. [c]Isolated yield.

Supplementary Table 4. Effect of base

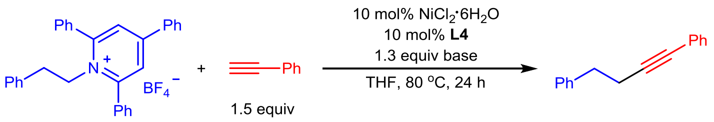

1a **2a** **3a**

entry	base	yield (%)[b]
1	K_3PO_4	99 (97)[c]
2	Na_3PO_4	38
3	K_2CO_3	95
4	Cs_2CO_3	65
5	Na_2CO_3	11
6	DBU	3
7	Et_3N	0

[a]Conditions: **1a** (0.3 mmol), **2a** (0.45 mmol), $NiCl_2 \cdot 6H_2O$ (10 mol%), **L4** (10 mol%), base (1.3 equiv), THF (1.5 mL), 80 °C. [b]Yields determined by ^{1}H NMR using 1,3,5-trimethoxybenzene as an internal standard. [c]Isolated yield.

Supplementary Table 5. Effect of catalyst loading, reaction temperature and blank experiment

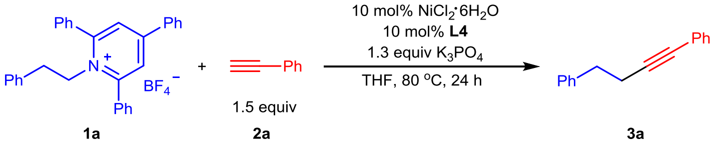

entry	NiCl$_2$·6H$_2$O (mol%)	**L4** (mol%)	K$_3$PO$_4$ (mol%)	yield (%)[b]
1	5	5	130	91
2[c]	10	10	130	68
3	-	10	130	0
4	10	-	130	0
5	10	10	-	0

[a]Conditions: **1a** (0.3 mmol), **2a** (0.45 mmol), NiCl$_2$·6H$_2$O (10 mol%), **L4** (10 mol%), K$_3$PO$_4$ (1.3 equiv), THF (1.5 mL), 80 °C. [b]Yields determined by ^{1}H NMR using 1,3,5-trimethoxybenzene as an internal standard. [c]60 °C.

General procedure A: synthesis of amide-type NN$_2$ pincer ligand

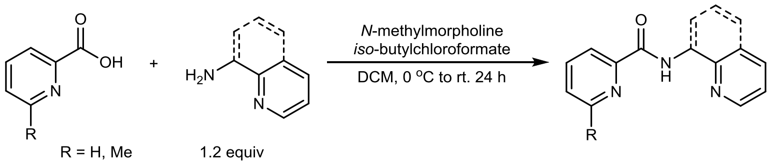

To an oven-dried round-bottomed flask was added carboxylic acid under nitrogen. Then dichloromethane (0.2 M) was added via syringe, followed by addition of *N*-methylmorpholine (1.5 equiv). When the reaction mixture was cooled to 0 °C, *iso*-butylchloroformate (1.2 equiv) was added, and the mixture was stirred at the same temperature for 30 min. Then amine (1.2 equiv) dissolved in dichloromethane was added and it was allowed to warm to room temperature. After stirring for 24 h, the reaction mixture was quenched with water and extracted with dichloromethane. The combined organic extracts were washed with water and brine, and dried over anhydrous Na$_2$SO$_4$. The solvent was concentrated under vacuum and the residue was purified by column chromatography on silica gel which was pretreated with triethylamine before loading the sample.

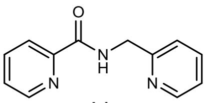

L1

***N*-(Pyridin-2-ylmethyl)picolinamide (L1).** 10.0 mmol scale, according to **general**

procedure A, the title product was obtained in 80% yield (1.70 g) as a white solid. ^{1}H NMR (400 MHz, $CDCl_3$): δ 4.81 (d, *J* = 5.6 Hz, 2H), 7.20 (dd, *J* = 4.8, 7.0 Hz, 1H), 7.35 (d, *J* = 8.0 Hz, 1H), 7.42-7.45 (m, 1H), 7.67 (td, *J* = 1.6, 7.6 Hz, 1H), 7.86 (td, *J* = 1.6, 7.6 Hz, 1H), 8.23 (d, *J* = 8.0 Hz, 1H), 8.59-8.61 (m, 2H), 8.94 (br, 1H). ^{13}C NMR (100 MHz, $CDCl_3$): δ 44.74, 121.88, 122.26, 122.29, 126.17, 136.73, 137.26, 148.26, 149.36, 149.89, 156.98, 164.50. IR (neat): 3274, 3057, 2919, 1669, 1589, 1568, 1523, 1462, 1434, 1413, 1355, 1303, 1288, 1254, 1239, 1216, 1172, 1151, 1089, 1058, 1049, 1037, 1004, 995, 957, 911, 891, 822, 764, 747, 725, 683, 620, 603, 523, 464, 426, 410 cm^{-1}. HRMS (ESI) calcd. for $C_{12}H_{12}N_3O$ $[M+H]^+$: 214.0975, found 214.0975.

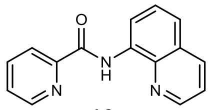

L2

***N*-(Quinolin-8-yl)picolinamide (L2).** 10.0 mmol scale, according to **general procedure A**, the title product was obtained in 70% yield (1.75 g) as a white solid. ^{1}H NMR (400 MHz, $CDCl_3$): δ 7.41-7.46 (m, 2H), 7.51-7.60 (m, 2H), 7.88 (t, *J* = 7.6 Hz, 1H), 8.12 (d, *J* = 8.0 Hz, 1H), 8.34 (d, *J* = 8.0 Hz, 1H), 8.74 (d, *J* = 4.4 Hz, 1H), 8.92 (d, *J* = 2.8 Hz, 1H), 9.01 (d, *J* = 7.6 Hz, 1H), 12.26 (br, 1H). ^{13}C NMR (100 MHz, $CDCl_3$): δ 116.63, 121.48, 121.90, 122.27, 126.17, 127.12, 127.98, 134.31, 136.05, 137.30, 139.16, 148.39, 148.56, 150.35, 162.55. IR (neat): 3290, 3052, 1684, 1589, 1569, 1521, 1485, 1464, 1434, 1425, 1387, 1324, 1284, 1241, 1208, 1177, 1131, 1087, 1052, 1042, 1000, 988, 976, 902, 827, 811, 797, 780, 765, 753, 736, 685, 640, 622, 598, 524, 423 cm^{-1}. HRMS (ESI) calcd. for $C_{15}H_{12}N_3O$ $[M+H]^+$: 250.0975, found 250.0976.

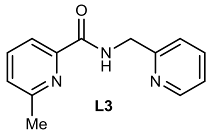

L3

6-Methyl-*N*-(pyridin-2-ylmethyl)picolinamide (L3). 10.0 mmol scale, according to **general procedure A**, the title product was obtained in 74% yield (1.68 g) as a white solid. ^{1}H NMR (400 MHz, $CDCl_3$): δ 2.59 (s, 3H), 4.80 (d, *J* = 6.0 Hz, 2H), 7.20 (dd, *J* = 5.2, 7.2

Hz, 1H), 7.28 (d, *J* = 8.0 Hz, 1H), 7.37 (d, *J* = 7.6 Hz, 1H), 7.67 (td, *J* = 1.6, 7.6 Hz, 1H), 7.73 (t, *J* = 8.0 Hz, 1H), 8.03 (d, *J* = 7.6 Hz, 1H), 8.60 (d, *J* = 4.4 Hz, 1H), 8.92 (br, 1H). ^{13}C NMR (100 MHz, $CDCl_3$): δ 24.26, 44.82, 119.34, 122.01, 122.29, 125.91, 136.79, 137.39, 149.14, 149.31, 157.30, 157.38, 164.75. IR (neat): 3278, 3051, 2981, 2932, 1659, 1591, 1567, 1520, 1474, 1448, 1426, 1378, 1358, 1321, 1306, 1259, 1219, 1177, 1151, 1098, 1081, 1052, 1037, 1008, 995, 967, 908, 825, 786, 771, 743, 704, 663, 633, 609, 565, 527, 509, 427, 419, 403 cm^{-1}. HRMS (ESI) calcd. for $C_{13}H_{14}N_3O$ [M+H]$^+$: 228.1131, found 228.1132.

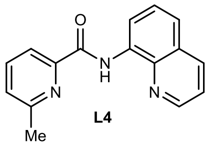

6-Methyl-*N*-(quinolin-8-yl)picolinamide (L4). 10.0 mmol scale, according to **general procedure A**, the title product was obtained in 68% yield (1.80 g) as a white solid. ^{1}H NMR (600 MHz, $CDCl_3$): δ 2.71 (s, 3H), 7.30 (d, *J* = 7.8 Hz, 1H), 7.43 (dd, *J* = 4.2, 8.4 Hz, 1H), 7.51 (d, *J* = 8.4 Hz, 1H), 7.57 (t, *J* = 8.4 Hz, 1H), 7.75 (t, *J* = 7.8 Hz, 1H), 8.13 (t, *J* = 7.8 Hz, 2H), 8.92 (d, *J* = 4.2 Hz, 1H), 9.00 (d, *J* = 7.2 Hz, 1H), 12.29 (br, 1H). ^{13}C NMR (151 MHz, $CDCl_3$): δ 24.41, 116.68, 119.39, 121.47, 121.82, 125.97, 127.17, 128.01, 134.50, 136.04, 137.44, 139.32, 148.57, 149.73, 157.44, 162.88. IR (neat): 3278, 3067, 1676, 1591, 1577, 1523, 1486, 1466, 1448, 1425, 1372, 1324, 1260, 1239, 1201, 1175, 1157, 1143, 1085, 1055, 992, 930, 885, 844, 822, 806, 787, 753, 705, 665, 637, 602, 553, 514, 482, 432, 421 cm^{-1}. HRMS (ESI) calcd. for $C_{16}H_{14}N_3O$ [M+H]$^+$: 264.1131, found 264.1131.

Synthesis of alkylpyridinium salts

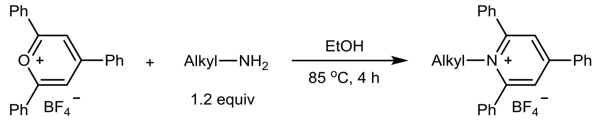

According to previous reported procedures, to a round-bottomed flask were added 2,4,6-triphenylpyrylium tetrafluoroborate (1.0 equiv), EtOH (1.0 M) and primary amine

(1.2 equiv). Then the flask was fitted with a reflux condenser and immersed into an oil bath preheated at 85 °C. After stirring for 4 h, the reaction mixture was cooled to room temperature. If product precipitation occurred, the solid was filtered, washed with EtOH and Et_2O, and dried under high vacuum. If product precipitation did not ocurr, then Et_2O was added to the reaction mixture and the solution was vigorously stirred for 1 h to induce trituration. The resulting solid was filtered and washed with Et_2O. If the pyridinium salt still did not precipitate, the reaction mixture was concentrated under vacuum and purified by column chromatography (acetone/dichloromethane).

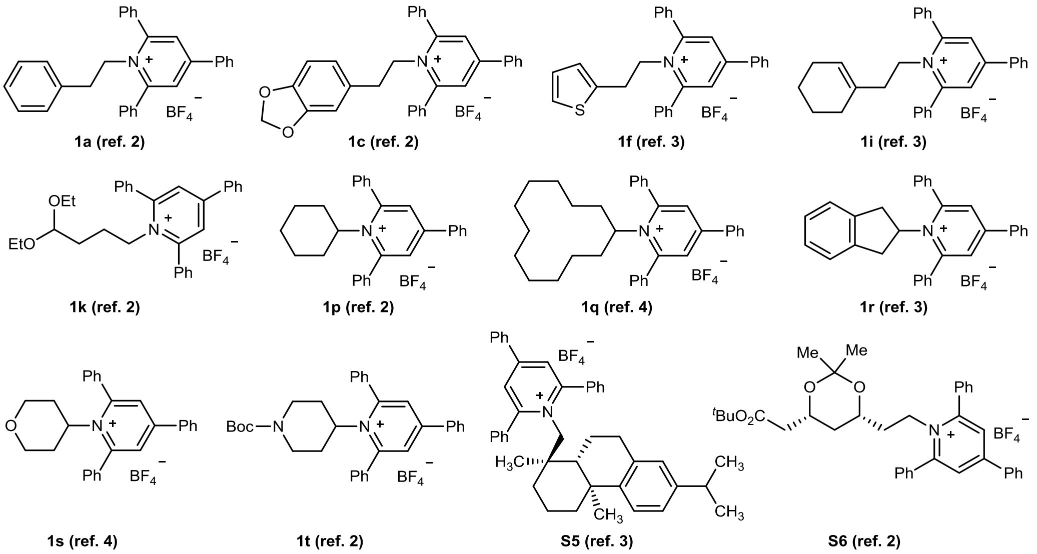

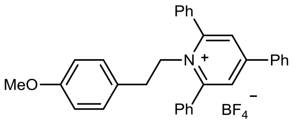

1b

1-(4-Methoxyphenethyl)-2,4,6-triphenylpyridin-1-ium tetrafluoroborate (1b). 5.0 mmol scale, the title product was obtained in 83% yield (2.20 g) as a white solid. ^{1}H NMR (400 MHz, $CDCl_3$): δ 2.61-2.65 (m, 2H), 3.69 (s, 3H), 4.57-4.62 (m, 2H), 6.20 (d, J = 8.4 Hz, 2H), 6.57-6.59 (m, 2H), 7.49-7.65 (m, 9H), 7.76-7.80 (m, 6H), 7.88 (s, 2H). ^{13}C NMR (100 MHz, $CDCl_3$): δ 34.64, 55.12, 55.88, 114.04, 126.56, 127.13, 128.06, 129.04, 129.11, 129.19, 129.51, 130.93, 131.89, 132.72, 133.95, 155.73, 156.31, 158.59. IR (neat): 3063, 1624, 1583, 1561, 1514, 1494, 1459, 1446, 1417, 1353, 1334, 1305, 1284, 1252, 1179, 1156, 1080, 1046, 1035, 997, 937, 898, 869, 825, 797, 767, 743, 726, 700, 650, 635, 610,

596, 554, 519, 504, 453, 429 cm^{-1}. HRMS (ESI) calcd. for $C_{32}H_{28}NO$ $[M-BF_4]^+$: 442.2165, found 442.2165.

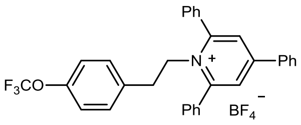

1d

2,4,6-Triphenyl-1-(4-(trifluoromethoxy)phenethyl)pyridin-1-ium tetrafluoroborate (1d). 3.0 mmol scale, the title product was obtained in 94% yield (1.65 g) as a white solid. ^{1}H NMR (400 MHz, $CDCl_3$): δ 2.69-2.74 (m, 2H), 4.58-4.62 (m, 2H), 6.28-6.31 (m, 2H), 6.89 (d, J = 8.0 Hz, 2H), 7.46-7.50 (m, 2H), 7.53-7.57 (m, 1H), 7.58-7.66 (m, 6H), 7.72-7.74 (m, 2H), 7.78-7.81 (m, 4H), 7.85 (s, 2H). ^{13}C NMR (100 MHz, $CDCl_3$): δ 34.60, 55.36, 120.20 (q, J = 255.7 Hz), 121.11, 126.61, 128.06, 129.03, 129.18, 129.45, 129.52, 130.95, 131.86, 132.66, 133.99, 134.23, 148.05 (q, J = 2.2 Hz), 155.87, 156.21. IR (neat): 3066, 1625, 1601, 1583, 1566, 1510, 1496, 1460, 1446, 1417, 1330, 1254, 1224, 1198, 1163, 1051, 920, 892, 871, 851, 800, 764, 704, 696, 672, 649, 613, 593, 533, 521, 499, 445 cm^{-1}. HRMS (ESI) calcd. for $C_{32}H_{25}F_3NO$ $[M-BF_4]^+$: 496.1883, found 496.1881.

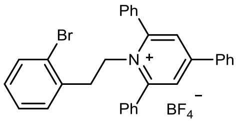

1e

1-(2-Bromophenethyl)-2,4,6-triphenylpyridin-1-ium tetrafluoroborate (1e). 3.0 mmol scale, the title product was obtained in 85% yield (1.47 g) as a white solid. ^{1}H NMR (600 MHz, $CDCl_3$): δ 2.82 (t, J = 7.8 Hz, 2H), 4.78 (t, J = 7.8 Hz, 2H), 6.15 (dd, J = 1.8, 7.2 Hz, 1H), 6.94-6.98 (m, 2H), 7.26-7.28 (m, 1H), 7.48 (t, J = 7.2 Hz, 2H), 7.54 (t, J = 7.2 Hz, 1H), 7.57-7.60 (m, 6H), 7.74 (d, J = 7.8 Hz, 2H), 7.78-7.80 (m, 4H), 7.84 (s, 2H). ^{13}C NMR (151 MHz, $CDCl_3$): δ 35.52, 53.77, 124.05, 126.78, 127.85, 128.12, 128.99, 129.31, 129.44, 129.60, 130.30, 130.92, 132.02, 132.75, 132.80, 133.97, 135.16, 155.96, 156.61. IR (neat): 3068, 1622, 1601, 1567, 1512, 1495, 1462, 1446, 1417, 1355, 1329, 1286, 1247, 1183, 1163, 1049, 1026, 894, 852, 827, 780, 760, 697, 660, 613, 597, 578, 518, 495, 453 cm^{-1}. HRMS (ESI) calcd. for $C_{31}H_{25}BrN$ $[M-BF_4]^+$: 490.1165, found 490.1167.

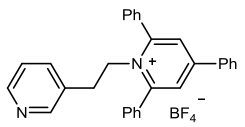

1g

2,4,6-Triphenyl-1-(2-(pyridin-3-yl)ethyl)pyridin-1-ium tetrafluoroborate (1g). 3.0 mmol scale, the title product was obtained in 67% yield (1.0 g) as a white solid. ^{1}H NMR (400 MHz, $CDCl_3$): δ 2.71 (t, *J* = 8.0 Hz, 2H), 4.50 (t, *J* = 8.0 Hz, 2H), 6.55 (d, *J* = 7.6 Hz, 1H), 6.93-6.96 (m, 1H), 7.38 (t, *J* = 7.2 Hz, 2H), 7.46-7.64 (m, 10H), 7.73 (s, 2H), 7.80 (d, *J* = 7.2 Hz, 4H), 8.28 (d, *J* = 4.4 Hz, 1H). ^{13}C NMR (100 MHz, $CDCl_3$): δ 32.36, 54.93, 123.42, 126.52, 127.97, 128.89, 129.11, 129.35, 130.95, 131.03, 131.78, 132.50, 133.88, 135.58, 148.30, 149.11, 155.81, 156.03. IR (neat): 3070, 1623, 1600, 1567, 1494, 1481, 1461, 1445, 1419, 1354, 1327, 1285, 1255, 1239, 1182, 1165, 1049, 1035, 913, 894, 805, 782, 764, 738, 698, 637, 621, 595, 578, 516, 496, 411 cm^{-1}. HRMS (ESI) calcd. for $C_{30}H_{25}N_2$ $[M-BF_4]^+$: 413.2012, found 413.2015.

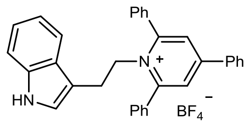

1h

1-(2-(1*H*-Indol-3-yl)ethyl)-2,4,6-triphenylpyridin-1-ium tetrafluoroborate (1h). 2.0 mmol scale, the title product was obtained in 81% yield (0.87 g) as a light-yellow solid. ^{1}H NMR (600 MHz, DMSO-*d*$_6$): δ 2.78 (t, *J* = 8.4 Hz, 2H), 4.58 (t, *J* = 7.8 Hz, 2H), 6.32 (d, *J* = 7.8 Hz, 1H), 6.53 (d, *J* = 1.8 Hz, 1H), 6.71 (t, *J* = 7.8 Hz, 1H), 7.00 (t, *J* = 7.8 Hz, 1H), 7.27 (d, *J* = 7.8 Hz, 1H), 7.63 (t, *J* = 7.8 Hz, 2H), 7.66-7.70 (m, 5H), 7.73 (t, *J* = 7.2 Hz, 2H), 7.80 (d, *J* = 7.8 Hz, 4H), 8.27 (d, *J* = 7.8 Hz, 2H), 8.48 (s, 2H), 10.82 (s, 1H). ^{13}C NMR (151 MHz, DMSO-*d*$_6$): δ 25.12, 55.33, 107.96, 111.53, 117.00, 118.55, 121.25, 123.03, 126.04, 126.28, 128.72, 129.06, 129.30, 129.60, 130.86, 132.40, 133.06, 133.21, 136.02, 154.24, 156.07. IR (neat): 3364, 3050, 1623, 1600, 1566, 1494, 1458, 1446, 1418, 1352, 1331, 1287, 1240, 1183, 1160, 1078, 1064, 1006, 937, 893, 861, 818, 799, 767, 739, 724, 700, 660, 645, 626, 595, 559, 517, 497, 466, 424, 408 cm^{-1}. HRMS (ESI) calcd. for $C_{33}H_{27}N_2$ $[M-BF_4]^+$: 451.2169, found 451.2170.

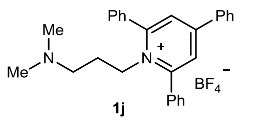

1j

1-(3-(Dimethylamino)propyl)-2,4,6-triphenylpyridin-1-ium tetrafluoroborate (1j). 3.0 mmol scale, the title product was obtained in 67% yield (0.97 g) as a white solid. ^{1}H NMR (600 MHz, $CDCl_3$): δ 1.60-1.62 (m, 2H), 1.68-1.71 (m, 8H), 4.43 (t, *J* = 8.4 Hz, 2H), 7.45 (t, *J* = 7.2 Hz, 2H), 7.51 (t, *J* = 7.2 Hz, 1H), 7.58 (br, 6H), 7.70 (d, *J* = 7.2 Hz, 2H), 7.79 (br, 6H). ^{13}C NMR (151 MHz, $CDCl_3$): δ 27.42, 44.54, 53.57, 55.85, 126.72, 128.07, 128.98, 129.20, 129.52, 130.84, 131.82, 132.75, 134.07, 155.66, 156.28. IR (neat): 3070, 2945, 2820, 2777, 1625, 1602, 1583, 1568, 1496, 1460, 1447, 1418, 1373, 1359, 1326, 1281, 1250, 1182, 1165, 1035, 960, 893, 851, 833, 816, 794, 778, 762, 725, 696, 646, 611, 596, 548, 521, 495, 456, 430 cm^{-1}. HRMS (ESI) calcd. for $C_{28}H_{29}N_2$ $[M-BF_4]^+$: 393.2325, found 393.2327.

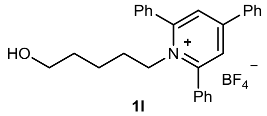

1l

1-(5-Hydroxypentyl)-2,4,6-triphenylpyridin-1-ium tetrafluoroborate (1l). 3.0 mmol scale, the title product was obtained in 67% yield (0.96 g) as a white solid. ^{1}H NMR (400 MHz, $CDCl_3$): δ 0.77-0.84 (m, 2H), 0.97-1.04 (m, 2H), 1.42-1.49 (m, 2H), 1.87 (t, *J* = 5.2 Hz, 1H), 3.24 (q, *J* = 5.6 Hz, 2H), 4.39 (t, *J* = 8.0 Hz, 2H), 7.47 (t, *J* = 7.6 Hz, 2H), 7.53 (t, *J* = 7.2 Hz, 1H), 7.57-7.58 (m, 6H), 7.71 (d, *J* = 7.6 Hz, 2H), 7.76-7.77 (m, 4H), 7.81 (s, 2H). ^{13}C NMR (100 MHz, $CDCl_3$): δ 22.24, 29.18, 30.64, 54.71, 61.46, 126.61, 128.05, 128.96, 129.26, 129.62, 131.00, 132.01, 132.65, 133.91, 155.58, 156.44. IR (neat): 3588, 3071, 2927, 2860, 1624, 1601, 1567, 1496, 1460, 1446, 1418, 1357, 1326, 1283, 1241, 1165, 1049, 893, 852, 763, 697, 609, 596, 521, 496, 408 cm^{-1}. HRMS (ESI) calcd. for $C_{28}H_{28}NO$ $[M-BF_4]^+$: 394.2165, found 394.2167.

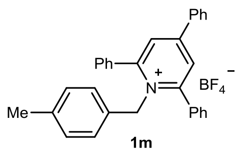

1m

1-(4-Methylbenzyl)-2,4,6-triphenylpyridin-1-ium tetrafluoroborate (1m). 5.0 mmol

scale, the title product was obtained in 83% yield (2.08 g) as a white solid. ^{1}H NMR (400 MHz, $CDCl_3$): δ 2.25 (s, 3H), 5.73 (s, 2H), 6.35 (d, *J* = 8.4 Hz, 2H), 6.91 (d, *J* = 8.0 Hz, 2H), 7.45-7.51 (m, 6H), 7.53-7.60 (m, 3H), 7.64-7.66 (m, 4H), 7.82-7.84 (m, 2H), 7.96 (s, 2H). ^{13}C NMR (100 MHz, $CDCl_3$): δ 20.64, 57.76, 125.84, 126.16, 127.83, 128.77, 129.13, 129.39, 130.41, 130.59, 131.98, 132.47, 133.29, 137.84, 155.77, 157.02 *(1 aromatic carbon signal is not observed due to signal overlap)*. IR (neat): 3062, 1619, 1600, 1563, 1517, 1495, 1446, 1416, 1345, 1284, 1187, 1163, 1032, 999, 932, 890, 856, 784, 763, 750, 698, 611, 595, 549, 520, 507, 482 cm^{-1}. HRMS (ESI) calcd. for $C_{31}H_{26}N$ $[M-BF_4]^+$: 412.2060, found 412.2058.

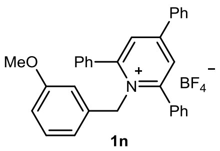

1n

1-(3-Methoxybenzyl)-2,4,6-triphenylpyridin-1-ium tetrafluoroborate (1n). 3.0 mmol scale, the title product was obtained in 85% yield (1.32 g) as a white solid. ^{1}H NMR (400 MHz, $CDCl_3$): δ 3.59 (s, 3H), 5.71 (s, 2H), 5.93 (s, 1H), 6.05 (d, *J* = 7.6 Hz, 1H), 6.67 (d, *J* = 8.0 Hz, 1H), 7.03 (t, *J* = 8.0 Hz, 1H), 7.43-7.56 (m, 9H), 7.65 (d, *J* = 7.2 Hz, 4H), 7.76 (d, *J* = 7.2 Hz, 2H), 7.88 (s, 2H). ^{13}C NMR (100 MHz, $CDCl_3$): δ 55.09, 58.02, 111.98, 113.62, 118.21, 126.34, 128.04, 128.99, 129.03, 129.67, 129.91, 130.83, 132.30, 132.59, 133.50, 135.27, 156.15, 157.38, 159.57. IR (neat): 3063, 1620, 1600, 1563, 1493, 1461, 1416, 1367, 1340, 1281, 1264, 1159, 1030, 997, 890, 851, 762, 698, 612, 597, 557, 519, 491, 444 cm^{-1}. HRMS (ESI) calcd. for $C_{31}H_{26}NO$ $[M-BF_4]^+$: 428.2009, found 428.2005.

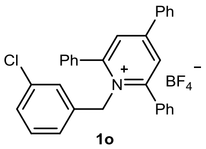

1o

1-(3-Chlorobenzyl)-2,4,6-triphenylpyridin-1-ium tetrafluoroborate (1o). 3.0 mmol scale, the title product was obtained in 66% yield (1.03 g) as a white solid. ^{1}H NMR (400 MHz, $CDCl_3$): δ 5.72 (s, 2H), 6.31 (s, 1H), 6.45 (d, *J* = 6.4 Hz, 1H), 7.06-7.10 (m, 2H), 7.44-7.56 (m, 9H), 7.65 (d, *J* = 7.2 Hz, 4H), 7.76 (d, *J* = 7.6 Hz, 2H), 7.89 (s, 2H). ^{13}C

NMR (100 MHz, $CDCl_3$): δ 57.46, 124.55, 126.50, 126.60, 128.12, 128.41, 129.00, 129.19, 129.71, 130.28, 131.04, 132.39, 132.49, 133.52, 134.43, 135.59, 156.47, 157.32. IR (neat): 3063, 1618, 1599, 1578, 1560, 1495, 1477, 1459, 1435, 1413, 1347, 1263, 1184, 1156, 1046, 998, 949, 888, 850, 782, 767, 754, 703, 684, 612, 589, 521, 507, 436 cm^{-1}. HRMS (ESI) calcd. for $C_{30}H_{23}ClN$ $[M-BF_4]^+$: 432.1514, found 432.1512.

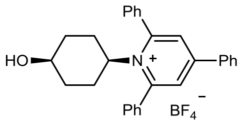

1u

***cis*-1-(4-Hydroxycyclohexyl)-2,4,6-triphenylpyridin-1-ium tetrafluoroborate (1u).** 3.0 mmol scale, the title product was obtained in 65% yield (0.96 g) as a white solid. ^1H NMR (600 MHz, $CDCl_3$): δ 0.92 (t, *J* = 13.2 Hz, 2H), 1.60 (d, *J* = 13.8 Hz, 2H), 1.83 (t, *J* = 12.0 Hz, 3H), 1.99 (q, *J* = 12.6 Hz, 2H), 3.64 (s, 1H), 4.62 (t, *J* = 12.0 Hz, 1H), 7.44 (t, *J* = 7.2 Hz, 2H), 7.51 (t, *J* = 7.2 Hz, 1H), 7.55-7.58 (m, 6H), 7.70-7.71 (m, 6H), 7.77 (s, 2H). ^{13}C NMR (151 MHz, $CDCl_3$): δ 26.89, 32.69, 62.94, 71.47, 127.92, 128.15, 128.82, 129.09, 129.52, 130.81, 131.88, 133.78, 133.82, 154.81, 157.17. IR (neat): 3395, 3064, 2927, 1621, 1601, 1565, 1495, 1446, 1415, 1357, 1288, 1242, 1224, 1186, 1141, 1052, 970, 936, 909, 893, 778, 763, 749, 699, 643, 621, 600, 536, 520, 494, 428 cm^{-1}. HRMS (ESI) calcd. for $C_{29}H_{28}NO$ $[M-BF_4]^+$: 406.2165, found 406.2169.

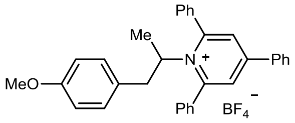

1v

1-(1-(4-Methoxyphenyl)propan-2-yl)-2,4,6-triphenylpyridin-1-ium tetrafluoroborate (1v). 3.0 mmol scale, the title product was obtained in 55% yield (0.90 g) as a light-yellow solid. ^1H NMR (400 MHz, $CDCl_3$): δ 1.29 (d, *J* = 6.8 Hz, 3H), 2.51 (dd, *J* = 9.6, 13.6 Hz, 1H), 3.25 (dd, *J* = 5.2, 13.6 Hz, 1H), 3.69 (s, 3H), 5.04-5.09 (m, 1H), 6.50 (d, *J* = 8.4 Hz, 2H), 6.61 (d, *J* = 8.8 Hz, 2H), 7.39 (t, *J* = 7.2 Hz, 2H), 7.48 (t, *J* = 7.2 Hz, 1H), 7.53-7.61 (m, 7H), 7.66 (d, *J* = 7.6 Hz, 3H), 7.70 (s, 2H), 7.76 (br, 2H). ^{13}C NMR (100 MHz, $CDCl_3$): δ 20.81, 41.90, 55.12, 68.27, 113.96, 128.11, 128.14, 128.70, 129.42, 129.55, 130.82,

131.78, 133.78, 133.84, 154.93, 157.12, 158.60 *(2 aromatic carbon signal is not observed due to signal overlap)*. IR (neat): 3061, 1618, 1562, 1512, 1494, 1445, 1412, 1354, 1303, 1249, 1181, 1027, 889, 818, 761, 704, 639, 615, 598, 555, 519 cm^{-1}. HRMS (ESI) calcd. for $C_{33}H_{30}NO$ $[M-BF_4]^+$: 456.2322, found 456.2322.

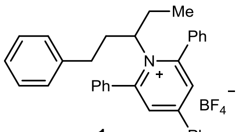

1w

2,4,6-Triphenyl-1-(1-phenylpentan-3-yl)pyridin-1-ium tetrafluoroborate (1w). 3.0 mmol scale, the title product was obtained in 64% yield (1.03 g) as a white solid. ^1H NMR (600 MHz, $CDCl_3$): δ 0.65 (t, *J* = 7.8 Hz, 3H), 1.54-1.59 (m, 1H), 1.79-1.82 (m, 1H), 1.97-2.02 (m, 1H), 2.24-2.31 (m, 3H), 4.66 (t, *J* = 6.6 Hz, 1H), 6.93 (d, *J* = 7.2 Hz, 2H), 7.14-7.20 (m, 3H), 7.42 (t, *J* = 7.8 Hz, 2H), 7.49-7.57 (m, 8H), 7.66-7.81 (m, 7H). ^{13}C NMR (151 MHz, $CDCl_3$): 11.14, 28.66, 32.52, 35.42, 72.72, 126.39, 126.61, 127.93, 128.23, 128.60, 129.50, 129.93, 130.89, 132.00, 133.61, 139.03, 155.10, 156.66, 158.23 *(1 aromatic carbon signal is not observed due to signal overlap)*. IR (neat): 3061, 2933, 1618, 1599, 1561, 1494, 1446, 1411, 1352, 1283, 1240, 1184, 1162, 1050, 999, 892, 762, 698, 620, 579, 519, 493, 406 cm^{-1}. HRMS (ESI) calcd. for $C_{34}H_{32}N$ $[M-BF_4]^+$: 454.2529, found 454.2530.

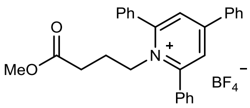

1x

1-(4-Methoxy-4-oxobutyl)-2,4,6-triphenylpyridin-1-ium tetrafluoroborate (1x). 5.0 mmol scale, the title product was obtained in 70% yield (1.73 g) as a white solid. ^1H NMR (600 MHz, $CDCl_3$): δ 1.75-1.78 (m, 2H), 1.82 (t, *J* = 7.2 Hz, 2H), 3.42 (s, 3H), 4.51 (t, *J* = 7.8 Hz, 2H), 7.50 (t, *J* = 7.2 Hz, 2H), 7.55 (t, *J* = 7.2 Hz, 1H), 7.60-7.61 (m, 6H), 7.73-7.75 (m, 2H), 7.80-7.81 (m, 4H), 7.85 (s, 2H). ^{13}C NMR (100 MHz, $CDCl_3$): δ 24.60, 30.50, 51.63, 53.89, 126.71, 128.07, 129.00, 129.29, 129.58, 130.99, 131.99, 132.59, 133.92, 155.85, 156.49, 171.50. IR (neat): 3068, 1734, 1624, 1601, 1567, 1504, 1464, 1448, 1417,

1366, 1330, 1273, 1250, 1214, 1197, 1173, 1163, 1095, 1049, 968, 891, 839, 782, 767, 736, 697, 638, 610, 596, 538, 520, 491, 411 cm^{-1}. HRMS (ESI) calcd. for $C_{28}H_{26}NO_2$ $[M-BF_4]^+$: 408.1958, found 408.1951.

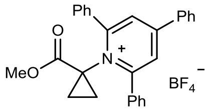

1y

1-(1-(Methoxycarbonyl)cyclopropyl)-2,4,6-triphenylpyridin-1-ium tetrafluoroborate (1y). 5.0 mmol scale, the title product was obtained in 64% yield (1.59 g) as a white solid. ^{1}H NMR (600 MHz, $CDCl_3$): δ 1.24-1.26 (m, 2H), 1.75-1.77 (m, 2H), 7.52-7.59 (m, 10H), 7.73 (br 3H), 7.83-7.84 (m, 2H), 7.93 (s, 2H). ^{13}C NMR (100 MHz, $CDCl_3$): δ 23.88, 50.47, 53.64, 127.59, 128.34, 128.86, 129.55, 130.85, 132.04, 133.68, 134.23, 157.42, 158.86, 171.94 *(1 aromatic carbon signal is not observed due to signal overlap)*. IR (neat): 3066, 1730, 1623, 1599, 1562, 1497, 1446, 1415, 1337, 1286, 1252, 1209, 1200, 1173, 1094, 1049, 1034, 1001, 971, 901, 891, 769, 753, 726, 702, 682, 609, 542, 520, 510, 490 cm^{-1}. HRMS (ESI) calcd. for $C_{28}H_{24}NO_2$ $[M-BF_4]^+$: 406.1802, found 406.1795.

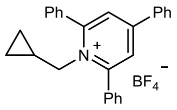

1ab

1-(Cyclopropylmethyl)-2,4,6-triphenylpyridin-1-ium tetrafluoroborate (1ab). 3.0 mmol scale, the title product was obtained in 77% yield (1.04 g) as a white solid. ^{1}H NMR (600 MHz, $CDCl_3$): δ -0.35 (s, 2H), 0.29 (d, *J* = 6.6 Hz, 2H), 0.67 (t, *J* = 6.0 Hz, 1H), 4.53 (d, *J* = 6.6 Hz, 2H), 7.52-7.59 (m, 3H), 7.61 (br, 6H), 7.79 (d, *J* = 7.2 Hz, 2H), 7.83-7.84 (m, 4H), 7.90 (s, 2H). ^{13}C NMR (151 MHz, $CDCl_3$): δ 4.83, 10.41, 58.97, 126.41, 127.88, 129.11, 129.24, 129.50, 130.86, 131.94, 133.02, 133.58, 155.34, 156.58. IR (neat): 3062, 1619, 1600, 1563, 1496, 1460, 1446, 1414, 1325, 1284, 1186, 1152, 1047, 998, 932, 889, 854, 835, 766, 735, 700, 596, 531, 520, 490 cm^{-1}. HRMS (ESI) calcd. for $C_{27}H_{24}N$ $[M-BF_4]^+$: 362.1903, found 362.1900.

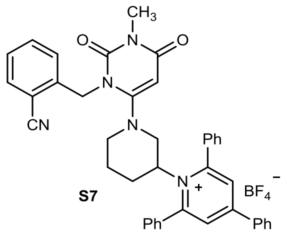

S7

1-(1-(3-(2-Cyanobenzyl)-1-methyl-2,6-dioxo-1,2,3,6-tetrahydropyrimidin-4-yl)piperidin-3-yl)-2,4,6-triphenylpyridin-1-ium tetrafluoroborate (S7). 5.0 mmol scale, the title product was obtained in 57% yield (2.05 g) as a white solid. ^{1}H NMR (600 MHz, $CDCl_3$): δ 1.16-1.18 (m, 1H), 1.58 (d, *J* = 11.4 Hz, 1H), 1.82 (br, 1H), 2.01 (br, 2H), 2.36 (d, *J* = 11.4 Hz, 1H), 2.84-2.91 (m, 4H), 3.65 (d, *J* = 10.2 Hz, 1H), 4.72 (br, 1H), 4.81 (d, *J* = 15.0 Hz, 1H), 4.88 (t, *J* = 11.4 Hz, 1H), 4.97 (s, 1H), 7.11 (d, *J* = 7.2 Hz, 1H), 7.29 (s, 1H), 7.34-7.42 (m, 8H), 7.46-7.55 (m, 3H), 7.69-7.70 (m, 6H), 7.78 (s, 2H). ^{13}C NMR (100 MHz, $CDCl_3$): δ 24.56, 27.31, 31.50, 46.12, 50.54, 55.13, 66.42, 90.17, 110.51, 116.84, 127.95, 128.33, 128.49, 129.29, 130.02, 130.77, 131.71, 132.83, 132.92, 133.48, 133.83, 139.90, 151.26, 155.22, 156.36, 157.88, 162.45 *(2 aromatic carbon signal is not observed due to signal overlap)*. IR (neat): 3064, 2224, 1702, 1649, 1618, 1599, 1562, 1493, 1442, 1353, 1283, 1218, 1051, 1030, 947, 891, 854, 809, 764, 702, 603, 551, 520, 480, 414 cm^{-1}. HRMS (ESI) calcd. for $C_{41}H_{36}N_5O_2$ $[M-BF_4]^+$: 630.2864, found 630.2870.

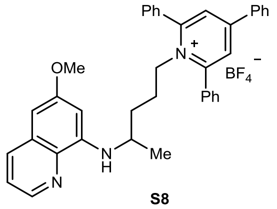

S8

1-(4-((6-Methoxyquinolin-8-yl)amino)pentyl)-2,4,6-triphenylpyridin-1-ium tetrafluoroborate (S8). 3.0 mmol scale, the title product was obtained in 56% yield (1.07 g) as a light-yellow solid. ^{1}H NMR (400 MHz, $CDCl_3$): δ 0.96-1.08 (m, 5H), 1.56-1.59 (m, 2H), 3.18 (br, 1H), 3.83 (s, 3H), 4.41 (t, *J* = 8.0 Hz, 2H), 5.63 (d, *J* = 8.0 Hz, 1H), 5.95 (s, 1H), 6.33 (s, 1H), 7.29-7.32 (m, 1H), 7.38-7.49 (m, 9H), 7.62 (d, *J* = 7.6 Hz, 2H), 7.71 (br, 6H), 7.92 (d, *J* = 8.0 Hz, 1H), 8.50 (d, *J* = 3.2 Hz, 1H). ^{13}C NMR (100 MHz, $CDCl_3$): δ 19.93, 25.98, 32.45, 46.35, 54.48, 55.05, 91.86, 96.57, 121.78, 126.27, 127.83, 128.68, 128.98, 129.40, 129.65, 130.72, 131.82, 132.39, 133.64, 134.65, 134.93, 144.01, 144.09,

155.31, 156.21, 159.09. IR (neat): 2929, 1619, 1565, 1518, 1496, 1456, 1421, 1387, 1348, 1220, 1160, 1047, 889, 825, 787, 763, 701, 636, 596, 520, 483 cm^{-1}. HRMS (ESI) calcd. for $C_{38}H_{36}N_3O$ [M-BF_4]$^+$: 550.2853, found 550.2855.

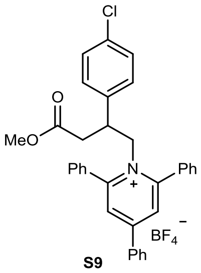

S9

1-(2-(4-Chlorophenyl)-4-methoxy-4-oxobutyl)-2,4,6-triphenylpyridin-1-ium tetrafluoroborate (S9). 3.0 mmol scale, the title product was obtained in 70% yield (1.27 g) as a white solid. ^{1}H NMR (400 MHz, $CDCl_3$): δ 2.07 (dd, *J* = 6.4, 16.0 Hz, 1H), 2.23 (dd, *J* = 8.0, 16.4 Hz, 1H), 3.12-3.19 (m, 1H), 3.38 (s, 3H), 4.93 (dd, *J* = 8.4, 14.8 Hz, 1H), 5.05 (dd, *J* = 6.4, 14.4 Hz, 1H), 6.35 (d, *J* = 8.4 Hz, 2H), 7.02 (d, *J* = 8.4 Hz, 2H), 7.47-7.60 (m, 10H), 7.72-7.94 (m, 7H). ^{13}C NMR (100 MHz, $CDCl_3$): δ 36.99, 40.83, 51.82, 59.07, 126.48, 128.04, 128.32, 129.12, 129.50, 129.75, 131.23, 132.43, 132.72, 133.38, 133.67, 136.44, 155.99, 157.29, 170.22 *(1 aromatic carbon signal is not observed due to signal overlap)*. IR (neat): 3062, 1732, 1617, 1598, 1558, 1493, 1445, 1413, 1351, 1281, 1187, 1156, 1051, 999, 939, 887, 861, 832, 822, 785, 773, 746, 705, 653, 611, 598, 558, 520 cm^{-1}. HRMS (ESI) calcd. for $C_{34}H_{29}ClNO_2$ [M-BF_4]$^+$: 518.1881, found 518.1881.

Synthesis of terminal alkynes

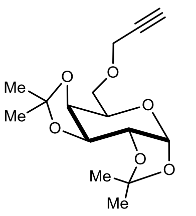

S10 (ref. 5)

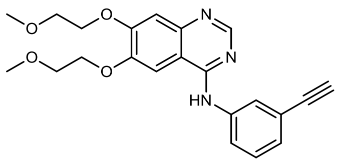

S11 (ref. 6)

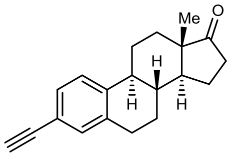

S13 (ref. 7)

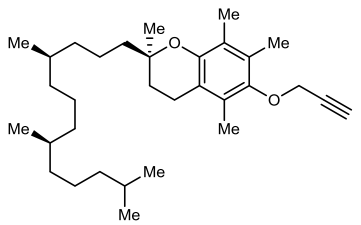

S14 (ref. 7)

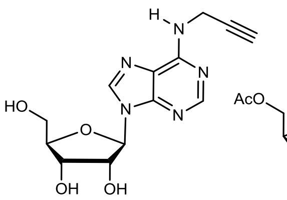

S19 (ref. 8)

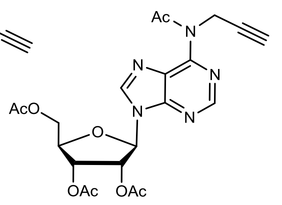

S20 (ref. 8)

Nickel-catalyzed deaminative Sonogashira coupling of alkylpyridinium salts

General procedure B: Sonogashira coupling of 1a with alkynes

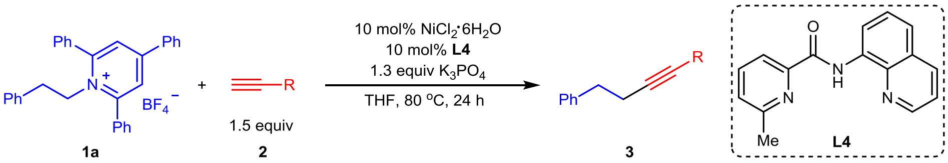

In a nitrogen-filled glovebox, $NiCl_2 \cdot 6H_2O$ (0.03 mmol, 7.1 mg), **L4** (0.03 mmol, 7.9 mg), anhydrous K_3PO_4 (0.39 mmol, 82.8 mg), phenethylpyridinium salt **1a** (0.3 mmol, 150.0 mg) and tetrahydrofuran (1.5 mL) were successively added to an oven-dried sealable Schlenk tube (10.0 mL) followed by addition of terminal alkyne (0.45 mmol) via microliter syringe *(If terminal alkyne is a solid, it was added before the solvent)*. Then the tube was securely sealed and taken outside the glovebox. And it was immersed into an oil bath preheated at 80 °C. After stirring for 24 h, the reaction mixture was cooled to room temperature and filtered through a short pad of silica gel. Then the filter cake was washed with dichloromethane or ethyl acetate. The resulting solution was concentrated under vacuum and the residue was purified by column chromatography on silica gel.

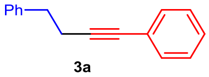

3a

But-1-yne-1,4-diyldibenzene (3a). 0.3 mmol scale, $NiCl_2 \cdot 6H_2O$ (0.03 mmol, 7.1 mg), **L4** (0.03 mmol, 7.9 mg), anhydrous K_3PO_4 (0.39 mmol, 82.8 mg), phenethylpyridinium salt **1a** (0.3 mmol, 150.0 mg), phenylacetylene (0.45 mmol, 46.0 mg) and tetrahydrofuran (1.5 mL) were stirred at 80 °C for 24 h. Then the reaction mixture was filtered through a short pad of silica gel and washed with dichloromethane. Purification of the crude product by column chromatography on silica gel (eluent: petroleum ether: dichloromethane = 100:1 to 50:1, gradient) afforded the title product in 97% yield (60.1 mg) as a colorless oil. ^1H NMR (400 MHz, $CDCl_3$): δ 2.68 (t, J = 7.6 Hz, 2H), 2.91 (t, J = 7.6 Hz, 2H), 7.19-7.32 (m, 8H), 7.35-7.38 (m, 2H). ^{13}C NMR (100 MHz, $CDCl_3$): δ 21.64, 35.14, 81.30, 89.47, 123.83, 126.27, 127.58, 128.16, 128.35, 128.51, 131.50, 141.67. IR (neat): 3062, 3028, 2927, 1599, 1490, 1454, 1442, 1341, 1071, 1030, 913, 754, 715, 691, 580, 530, 497 cm^{-1}. HRMS (EI)

calcd. for $C_{16}H_{14}$ $[M]^+$: 206.1090, found 206.1088.

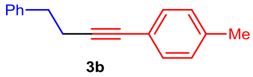

3b

1-Methyl-4-(4-phenylbut-1-yn-1-yl)benzene (3b). 0.3 mmol scale, $NiCl_2·6H_2O$ (0.03 mmol, 7.1 mg), **L4** (0.03 mmol, 7.9 mg), anhydrous K_3PO_4 (0.39 mmol, 82.8 mg), phenethylpyridinium salt **1a** (0.3 mmol, 150.0 mg), 1-ethynyl-4-methylbenzene (0.45 mmol, 52.3 mg) and tetrahydrofuran (1.5 mL) were stirred at 80 °C for 24 h. Then the reaction mixture was filtered through a short pad of silica gel and washed with dichloromethane. Purification of the crude product by column chromatography on silica gel (eluent: petroleum ether: dichloromethane = 50:1) afforded the title product in 95% yield (62.8 mg) as a colorless oil. ^1H NMR (400 MHz, $CDCl_3$): δ 2.23 (s, 3H), 2.59 (t, J = 7.6 Hz, 2H), 2.82 (t, J = 7.6 Hz, 2H), 6.98 (d, J = 8.0, 2H), 7.11-7.23 (m, 7H). ^{13}C NMR (100 MHz, $CDCl_3$): δ 21.35, 21.67, 35.23, 81.31, 88.66, 120.74, 126.25, 128.33, 128.51, 128.93, 131.37, 137.55, 140.74. IR (neat): 3027, 2922, 1603, 1509, 1496, 1454, 1428, 1341, 1278, 1180, 1106, 1077, 1031, 815, 747, 697, 575, 529, 497 cm^{-1}. HRMS (EI) calcd. for $C_{17}H_{16}$ $[M]^+$: 220.1247, found 220.1249.

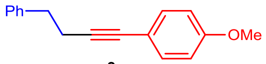

3c

1-Methoxy-4-(4-phenylbut-1-yn-1-yl)benzene (3c). 0.3 mmol scale, $NiCl_2·6H_2O$ (0.03 mmol, 7.1 mg), **L4** (0.03 mmol, 7.9 mg), anhydrous K_3PO_4 (0.39 mmol, 82.8 mg), phenethylpyridinium salt **1a** (0.3 mmol, 150.0 mg), 1-ethynyl-4-methoxybenzene (0.45 mmol, 59.5 mg) and tetrahydrofuran (1.5 mL) were stirred at 80 °C for 24 h. Then the reaction mixture was filtered through a short pad of silica gel and washed with dichloromethane. Purification of the crude product by column chromatography on silica gel (eluent: petroleum ether: dichloromethane = 20:1) afforded the title product in 92% yield (65.4 mg) as a colorless oil. ^1H NMR (400 MHz, $CDCl_3$): δ 2.58 (t, J = 7.6 Hz, 2H), 2.82 (t, J = 7.6 Hz, 2H), 3.68 (s, 3H), 6.71 (d, J = 8.4 Hz, 2H), 7.12-7.23 (m, 7H). ^{13}C NMR (100 MHz, $CDCl_3$): δ 21.64, 35.26, 55.16, 80.99, 87.83, 113.77, 115.95, 126.22,

128.31, 128.49, 132.80, 140.76, 159.05. IR (neat): 3021, 2934, 2838, 1889, 1605, 1566, 1508, 1454, 1440, 1303, 1287, 1246, 1180, 1172, 1106, 1078, 1027, 911, 830, 816, 796, 763, 734, 701, 665, 641, 582, 534, 498, 478, 420 cm^{-1}. HRMS (ESI) calcd. for $C_{17}H_{17}O$ $[M+H]^+$: 237.1274, found 237.1270.

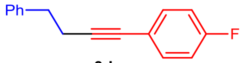

3d

1-Fluoro-4-(4-phenylbut-1-yn-1-yl)benzene (3d). 0.3 mmol scale, $NiCl_2·6H_2O$ (0.03 mmol, 7.1 mg), **L4** (0.03 mmol, 7.9 mg), anhydrous K_3PO_4 (0.39 mmol, 82.8 mg), phenethylpyridinium salt **1a** (0.3 mmol, 150.0 mg), 1-ethynyl-4-fluorobenzene (0.45 mmol, 54.1 mg) and tetrahydrofuran (1.5 mL) were stirred at 80 °C for 24 h. Then the reaction mixture was filtered through a short pad of silica gel and washed with dichloromethane. Purification of the crude product by column chromatography on silica gel (eluent: petroleum ether: dichloromethane = 50:1) afforded the title product in 97% yield (65.0 mg) as a colorless oil. ^{1}H NMR (400 MHz, $CDCl_3$): δ 2.66 (t, J = 7.6 Hz, 2H), 2.90 (t, J = 7.6 Hz, 2H), 6.92-6.97 (m, 2H), 7.20-7.34 (m, 7H). ^{13}C NMR (100 MHz, $CDCl_3$): δ 21.55, 35.09, 80.25, 89.09 (d, J = 1.4 Hz), 115.37 (d, J = 21.7 Hz), 119.87 (d, J = 2.9 Hz), 126.32, 128.36, 128.49, 133.27 (d, J = 8.0 Hz), 140.60, 162.08 (d, J = 246.3 Hz). IR (neat): 3028, 2928, 1602, 1505, 1454, 1429, 1342, 1219, 1155, 1092, 1078, 1030, 1014, 834, 810, 748, 698, 661, 632, 576, 530, 498 cm^{-1}. HRMS (ESI) calcd. for $C_{16}H_{14}F$ $[M+H]^+$: 225.1074, found 225.1071.

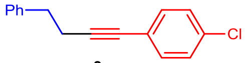

3e

1-Chloro-4-(4-phenylbut-1-yn-1-yl)benzene (3e). 0.3 mmol scale, $NiCl_2·6H_2O$ (0.03 mmol, 7.1 mg), **L4** (0.03 mmol, 7.9 mg), anhydrous K_3PO_4 (0.39 mmol, 82.8 mg), phenethylpyridinium salt **1a** (0.3 mmol, 150.0 mg), 1-chloro-4-ethynylbenzene (0.45 mmol, 61.5 mg) and tetrahydrofuran (1.5 mL) were stirred at 80 °C for 24 h. Then the reaction mixture was filtered through a short pad of silica gel and washed with dichloromethane. Purification of the crude product by column chromatography on silica gel (eluent:

petroleum ether: dichloromethane = 50:1) afforded the title product in 93% yield (67.0 mg) as a light-yellow oil. ^{1}H NMR (400 MHz, $CDCl_3$): δ 2.67 (t, *J* = 7.6 Hz, 2H), 2.90 (t, *J* = 7.6 Hz, 2H), 7.20-7.32 (m, 9H). ^{13}C NMR (100 MHz, $CDCl_3$): δ 21.61, 35.01, 80.28, 90.54, 122.32, 126.34, 128.38, 128.48, 132.72, 133.53, 140.52 *(1 aromatic carbon signal is not observed due to signal overlap)*. IR (neat): 3026, 2954, 1601, 1488, 1450, 1423, 1396, 1271, 1089, 1076, 1030, 1012, 827, 756, 740, 697, 638, 614, 527, 505, 442 cm^{-1}. HRMS (EI) calcd. for $C_{16}H_{13}Cl$ $[M]^+$: 240.0700, found 240.0702.

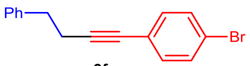

3f

1-Bromo-4-(4-phenylbut-1-yn-1-yl)benzene (3f). 0.3 mmol scale, $NiCl_2·6H_2O$ (0.03 mmol, 7.1 mg), **L4** (0.03 mmol, 7.9 mg), anhydrous K_3PO_4 (0.39 mmol, 82.8 mg), phenethylpyridinium salt **1a** (0.3 mmol, 150.0 mg), 1-bromo-4-ethynylbenzene (0.45 mmol, 81.5 mg) and tetrahydrofuran (1.5 mL) were stirred at 80 °C for 24 h. Then the reaction mixture was filtered through a short pad of silica gel and washed with dichloromethane. Purification of the crude product by column chromatography on silica gel (eluent: petroleum ether: dichloromethane = 50:1) afforded the title product in 94% yield (80.1 mg) as a light-yellow oil. ^{1}H NMR (400 MHz, $CDCl_3$): δ 2.66 (t, *J* = 7.6 Hz, 2H), 2.90 (t, *J* = 7.6 Hz, 2H), 7.19-7.32 (m, 7H), 7.38 (d, *J* = 8.4 Hz, 2H). ^{13}C NMR (100 MHz, $CDCl_3$): δ 21.64, 34.97, 80.34, 90.77, 121.70, 122.78, 126.34, 128.37, 128.47, 131.40, 132.96, 140.50. IR (neat): 3022, 2946, 1599, 1487, 1447, 1423, 1394, 1272, 1070, 1028, 1010, 976, 820, 756, 733, 701, 605, 522, 499, 432, 416 cm^{-1}. HRMS (EI) calcd. for $C_{16}H_{13}Br$ $[M]^+$: 284.0195, found 284.0198.

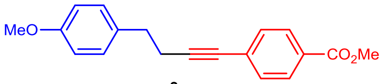

3g

Methyl 4-(4-(4-methoxyphenyl)but-1-yn-1-yl)benzoate (3g). 0.3 mmol scale, $NiCl_2·6H_2O$ (0.03 mmol, 7.1 mg), **L4** (0.03 mmol, 7.9 mg), anhydrous K_3PO_4 (0.39 mmol, 82.8 mg), *p*-methoxyphenethylpyridinium salt **1b** (0.3 mmol, 158.8 mg), methyl 4-ethynylbenzoate (0.45 mmol, 72.1 mg) and tetrahydrofuran (1.5 mL) were stirred at 80

°C for 24 h. Then the reaction mixture was filtered through a short pad of silica gel and washed with ethyl acetate. Purification of the crude product by column chromatography on silica gel (eluent: petroleum ether: ethyl acetate = 30:1) afforded the title product in 96% yield (85.1 mg) as a white solid. ^{1}H NMR (400 MHz, $CDCl_3$): δ 2.67 (t, J = 7.6 Hz, 2H), 2.86 (t, J = 7.6 Hz, 2H), 3.78 (s, 3H), 3.89 (s, 3H), 6.84-6.86 (m, 2H), 7.17 (d, J = 8.4 Hz, 2H), 7.41 (d, J = 8.4 Hz, 2H), 7.93-7.95 (m, 2H). ^{13}C NMR (100 MHz, $CDCl_3$): δ 21.96, 34.00, 52.05, 55.15, 80.76, 93.05, 113.74, 128.63, 128.87, 129.32, 129.38, 131.37, 132.51, 158.13, 166.53. IR (neat): 3003, 2949, 2835, 2217 , 1716, 1605, 1584, 1557, 1511, 1463, 1443, 1433, 1405, 1307, 1271, 1244, 1177, 1106, 1096, 1033, 1018, 958, 860, 829, 816, 769, 726, 696, 640, 580, 525, 464, 434 cm^{-1}. HRMS (ESI) calcd. for $C_{19}H_{18}NaO_3$ $[M+Na]^+$: 317.1148, found 317.1141.

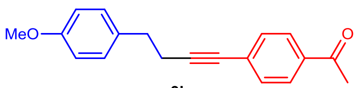

3h

1-(4-(4-(4-Methoxyphenyl)but-1-yn-1-yl)phenyl)ethan-1-one (3h). 0.3 mmol scale, $NiCl_2·6H_2O$ (0.03 mmol, 7.1 mg), **L4** (0.03 mmol, 7.9 mg), anhydrous K_3PO_4 (0.39 mmol, 82.8 mg), *p*-methoxyphenethylpyridinium salt **1b** (0.3 mmol, 158.8 mg), 1-(4-ethynylphenyl)ethan-1-one (0.45 mmol, 64.9 mg) and tetrahydrofuran (1.5 mL) were stirred at 80 °C for 24 h. Then the reaction mixture was filtered through a short pad of silica gel and washed with ethyl acetate. Purification of the crude product by column chromatography on silica gel (eluent: petroleum ether: ethyl acetate = 10:1) afforded the title product in 97% yield (80.7 mg) as a white solid. ^{1}H NMR (400 MHz, $CDCl_3$): δ 2.56 (s, 3H), 2.67 (t, J = 7.6 Hz, 2H), 2.86 (t, J = 7.6 Hz, 2H), 3.78 (s, 3H), 6.85 (d, J = 8.4 Hz, 2H), 7.18 (d, J = 8.4 Hz, 2H), 7.43 (d, J = 8.0 Hz, 2H), 7.85 (d, J = 8.4 Hz, 2H). ^{13}C NMR (100 MHz, $CDCl_3$): δ 21.96, 26.44, 33.96, 55.13, 80.76, 93.45, 113.72, 128.07, 128.80, 129.37, 131.53, 132.46, 135.66, 158.12, 197.22. IR (neat): 2922, 1679, 1611, 1598, 1555, 1511, 1447, 1425, 1401, 1357, 1302, 1286, 1259, 1242, 1175, 1108, 1074, 1030, 957, 833, 816, 775, 746, 631, 593, 568, 530, 469, 421 cm^{-1}. RMS (ESI) calcd. for $C_{19}H_{18}NaO_2$ $[M+Na]^+$: 301.1199, found 301.1199.

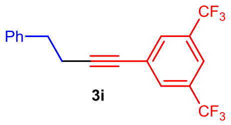

3i

1-(4-Phenylbut-1-yn-1-yl)-3,5-bis(trifluoromethyl)benzene (3i). 0.3 mmol scale, $NiCl_2 \cdot 6H_2O$ (0.03 mmol, 7.1 mg), **L4** (0.03 mmol, 7.9 mg), anhydrous K_3PO_4 (0.39 mmol, 82.8 mg), phenethylpyridinium salt **1a** (0.3 mmol, 150.0 mg), 1-ethynyl-3,5-bis-(trifluoromethyl)benzene (0.45 mmol, 107.2 mg) and tetrahydrofuran (1.5 mL) were stirred at 80 °C for 24 h. Then the reaction mixture was filtered through a short pad of silica gel and washed with dichloromethane. Purification of the crude product by column chromatography on silica gel (eluent: petroleum ether: dichloromethane = 100:1) afforded the title product in 95% yield (97.1 mg) as a light-yellow oil. ^{1}H NMR (400 MHz, $CDCl_3$): δ 2.72 (t, *J* = 7.6 Hz, 2H), 2.93 (t, *J* = 7.6 Hz, 2H), 7.22-7.34 (m, 5H), 7.74-7.76 (m, 3H). ^{13}C NMR (100 MHz, $CDCl_3$): δ 21.54, 34.71, 78.90, 93.61, 120.99 (q, *J* = 3.6 Hz), 123.02 (q, *J* = 270.9 Hz), 126.17, 126.56, 128.48, 128.50, 131.45 (q, *J* = 2.9 Hz), 131.76 (q, *J* = 33.2 Hz), 140.19. IR (neat): 3031, 2231, 1614, 1497, 1456, 1383, 1275, 1234, 1172, 1128, 1106, 1078, 1030, 1001, 896, 847, 747, 716, 698, 683, 582, 505, 427 cm^{-1}. HRMS (EI) calcd. for $C_{18}H_{12}F_6$ $[M]^+$: 342.0838, found 342.0841.

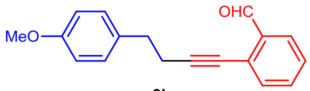

3j

2-(4-(4-Methoxyphenyl)but-1-yn-1-yl)benzaldehyde (3j). 0.3 mmol scale, $NiCl_2 \cdot 6H_2O$ (0.03 mmol, 7.1 mg), **L4** (0.03 mmol, 7.9 mg), anhydrous K_3PO_4 (0.39 mmol, 82.8 mg), *p*-methoxyphenethylpyridinium salt **1b** (0.3 mmol, 158.8 mg), 2-ethynylbenzaldehyde (0.45 mmol, 58.6 mg) and tetrahydrofuran (1.5 mL) were stirred at 80 °C for 24 h. Then the reaction mixture was filtered through a short pad of silica gel and washed with ethyl acetate. Purification of the crude product by column chromatography on silica gel (eluent: petroleum ether: ethyl acetate = 30:1) afforded the title product in 89% yield (70.9 mg) as a light-yellow oil. ^{1}H NMR (400 MHz, $CDCl_3$): δ 2.74 (t, *J* = 7.6 Hz, 2H), 2.88 (t, *J* = 7.6 Hz, 2H), 3.79 (s, 3H), 6.84-6.88 (m, 2H), 7.16-1.19 (m, 2H), 7.33-7.37 (m, 1H), 7.44-7.50 (m, 2H), 7.85-7.87 (m, 1H), 10.36 (s, 1H). ^{13}C NMR (100 MHz, $CDCl_3$): δ 21.96, 33.80, 55.17,

77.07, 97.12, 113.82, 126.78, 127.57, 127.88, 129.34, 132.24, 133.17, 133.55, 135.95, 158.22, 192.03. IR (neat): 2932, 2835, 2746, 2229, 1692, 1611, 1594, 1566, 1511, 1476, 1450, 1388, 1340, 1300, 1273, 1242, 1191, 1177, 1159, 1107, 1034, 879, 821, 760, 698, 637, 575, 555, 539, 520, 443 cm^{-1}. HRMS (ESI) calcd. for $C_{18}H_{16}NaO_2$ $[M+Na]^+$: 287.1043, found 287.1042.

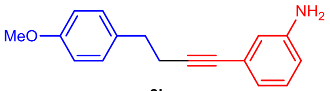

3k

3-(4-(4-Methoxyphenyl)but-1-yn-1-yl)aniline (3k). 0.3 mmol scale, $NiCl_2·6H_2O$ (0.03 mmol, 7.1 mg), **L4** (0.03 mmol, 7.9 mg), anhydrous K_3PO_4 (0.39 mmol, 82.8 mg), *p*-methoxyphenethylpyridinium salt **1b** (0.3 mmol, 158.8 mg), 3-ethynylaniline (0.45 mmol, 52.7 mg) and tetrahydrofuran (1.5 mL) were stirred at 80 °C for 24 h. Then the reaction mixture was filtered through a short pad of silica gel and washed with ethyl acetate. Purification of the crude product by column chromatography on silica gel (eluent: petroleum ether: ethyl acetate: dichloromethane = 10:1:3) afforded the title product in 88% yield (66.6 mg) as a yellow oil. ^1H NMR (400 MHz, $CDCl_3$): δ 2.62 (t, *J* = 7.6 Hz, 2H), 2.83 (t, *J* = 7.6 Hz, 2H), 3.53 (br, 2H), 3.76 (s, 3H), 6.56 (dd, *J* = 1.6, 8.0 Hz, 1H), 6.67 (s, 1H), 6.78 (d, *J* = 8.0 Hz, 1H), 6.83-6.85 (m, 2H), 7.04 (t, *J* = 8.0 Hz, 1H), 7.16-7.21 (m, 2H). ^{13}C NMR (100 MHz, $CDCl_3$): δ 21.85, 34.21, 55.14, 81.38, 88.95, 113.68, 114.64, 117.78, 121.85, 124.45, 129.05, 129.40, 132.82, 146.12, 158.01. IR (neat): 3432, 3350, 3226, 2927, 2840, 1627, 1611, 1595, 1580, 1510, 1484, 1441, 1318, 1301, 1241, 1205, 1178, 1162, 1107, 1021, 1000, 877, 822, 812, 784, 751, 690, 638, 560, 515, 463, 435, 410 cm^{-1}. HRMS (ESI) calcd. for $C_{17}H_{18}NO$ $[M+H]^+$: 252.1383, found 252.1384.

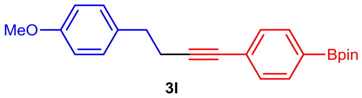

3l

2-(4-(4-(4-Methoxyphenyl)but-1-yn-1-yl)phenyl)-4,4,5,5-tetramethyl-1,3,2-dioxaborolane (3l). 0.3 mmol scale, $NiCl_2·6H_2O$ (0.03 mmol, 7.1 mg), **L4** (0.03 mmol, 7.9 mg), anhydrous K_3PO_4 (0.39 mmol, 82.8 mg), *p*-methoxyphenethylpyridinium salt **1b** (0.3 mmol, 158.8 mg), 2-(4-ethynylphenyl)-4,4,5,5-tetramethyl-1,3,2-dioxaborolane (0.45

mmol, 102.6 mg) and tetrahydrofuran (1.5 mL) were stirred at 80 °C for 24 h. Then the reaction mixture was filtered through a short pad of silica gel and washed with ethyl acetate. Purification of the crude product by column chromatography on silica gel (eluent: petroleum ether: ethyl acetate = 100:1 to 50:1, gradient) afforded the title product in 80% yield (86.8 mg) as a white solid. ^{1}H NMR (400 MHz, $CDCl_3$): δ 1.32 (s, 12H), 2.65 (t, J = 7.6 Hz, 2H), 2.85 (t, J = 7.6 Hz, 2H), 3.77 (s, 3H), 6.84 (d, J = 8.4 Hz, 2H), 7.17 (d, J = 8.4 Hz, 2H), 7.37 (d, J = 7.6 Hz, 2H), 7.72 (d, J = 8.0 Hz, 2H). ^{13}C NMR (100 MHz, $CDCl_3$): δ 22.00, 24.80, 34.20, 55.15, 81.46, 83.80, 91.13, 113.73, 126.61, 129.42, 130.68, 132.72, 134.46, 158.10 *(the signal for the aromatic carbon attached to boron is not observed due to quadrupolar relaxation)*. IR (neat): 2977, 2930, 1607, 1513, 1455, 1441, 1397, 1356, 1320, 1301, 1274, 1241, 1215, 1180, 1139, 1087, 1034, 1019, 961, 856, 837, 825, 764, 740, 702, 669, 655, 575, 536, 518, 451, 434, 407 cm^{-1}. HRMS (ESI) calcd. for $C_{23}H_{27}BNaO_3$ $[M+Na]^+$: 385.1945, found 385.1945.

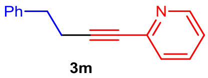

3m

2-(4-Phenylbut-1-yn-1-yl)pyridine (3m). 0.3 mmol scale, $NiCl_2 \cdot 6H_2O$ (0.03 mmol, 7.1 mg), **L4** (0.03 mmol, 7.9 mg), anhydrous K_3PO_4 (0.39 mmol, 82.8 mg), phenethylpyridinium salt **1a** (0.3 mmol, 150.0 mg), 2-ethynylpyridine (0.45 mmol, 46.4 mg) and tetrahydrofuran (1.5 mL) were stirred at 80 °C for 24 h. Then the reaction mixture was filtered through a short pad of silica gel and washed with ethyl acetate. Purification of the crude product by column chromatography on silica gel (eluent: petroleum ether: ethyl acetate = 10:1) afforded the title product in 89% yield (55.2 mg) as a colorless oil. ^{1}H NMR (400 MHz, $CDCl_3$): δ 2.72 (t, J = 7.6 Hz, 2H), 2.95 (t, J = 7.6 Hz, 2H), 7.14-7.17 (m, 1H), 7.20-7.33 (m, 6H), 7.57 (td, J = 1.6, 7.6 Hz, 1H), 8.53 (d, J = 4.4 Hz, 1H). ^{13}C NMR (100 MHz, $CDCl_3$): δ 21.43, 34.65, 80.85, 89.98, 122.26, 126.26, 126.69, 128.33, 135.92, 140.35, 143.65, 149.71 *(1 aromatic carbon signal is not observed due to signal overlap)*. IR (neat): 3061, 3027, 2928, 2229, 1582, 1561, 1496, 1463, 1427, 1341, 1272, 1149, 1091, 1077, 1049, 1030, 984, 777, 739, 698, 629, 582, 537, 501 cm^{-1}. HRMS (ESI) calcd. for $C_{15}H_{14}N$ $[M+H]^+$: 208.1121, found 208.1127.

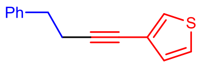

3n

3-(4-Phenylbut-1-yn-1-yl)thiophene (3n). 0.3 mmol scale, $NiCl_2 \cdot 6H_2O$ (0.03 mmol, 7.1 mg), **L4** (0.03 mmol, 7.9 mg), anhydrous K_3PO_4 (0.39 mmol, 82.8 mg), phenethylpyridinium salt **1a** (0.3 mmol, 150.0 mg), 3-ethynylthiophene (0.45 mmol, 48.7 mg) and tetrahydrofuran (1.5 mL) were stirred at 80 °C for 24 h. Then the reaction mixture was filtered through a short pad of silica gel and washed with dichloromethane. Purification of the crude product by column chromatography on silica gel (eluent: petroleum ether: dichloromethane = 100:1) afforded the title product in 90% yield (57.5 mg) as a colorless oil. ^{1}H NMR (400 MHz, $CDCl_3$): δ 2.66 (t, J = 7.6 Hz, 2H), 2.90 (t, J = 7.6 Hz, 2H), 7.03 (dd, J = 1.2, 4.8 Hz, 1H), 7.18-7.26 (m, 4H), 7.28-7.32 (m, 3H). ^{13}C NMR (100 MHz, $CDCl_3$): δ 21.60, 35.11, 76.32, 88.97, 122.75, 124.97, 126.28, 127.66, 128.35, 128.47, 129.90, 140.63. IR (neat): 3107, 3027, 2927, 1604, 1520, 1495, 1453, 1428, 1356, 1338, 1180, 1077, 1030, 860, 844, 778, 746, 697, 626, 580, 514, 492 cm^{-1}. HRMS (ESI) calcd. for $C_{14}H_{13}S$ $[M+H]^+$: 213.0732, found 213.0732.

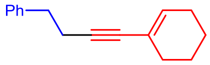

3o

(4-(Cyclohex-1-en-1-yl)but-3-yn-1-yl)benzene (3o). 0.3 mmol scale, $NiCl_2 \cdot 6H_2O$ (0.03 mmol, 7.1 mg), **L4** (0.03 mmol, 7.9 mg), anhydrous K_3PO_4 (0.39 mmol, 82.8 mg), phenethylpyridinium salt **1a** (0.3 mmol, 150.0 mg), 1-ethynylcyclohex-1-ene (0.45 mmol, 47.8 mg) and tetrahydrofuran (1.5 mL) were stirred at 80 °C for 24 h. Then the reaction mixture was filtered through a short pad of silica gel and washed with dichloromethane. Purification of the crude product by column chromatography on silica gel (eluent: petroleum ether: dichloromethane = 100:1) afforded the title product in 92% yield (58.3 mg) as a colorless oil. ^{1}H NMR (400 MHz, $CDCl_3$): δ 1.54-1.64 (m, 4H), 2.05-2.07 (m, 4H), 2.56 (t, J = 7.6 Hz, 2H), 2.83 (t, J = 7.6 Hz, 2H), 6.00 (s, 1H), 7.18-7.22 (m, 3H), 7.26-7.30 (m, 2H). ^{13}C NMR (100 MHz, $CDCl_3$): δ 21.54, 22.34, 25.50, 29.43, 35.39, 82.98, 86.48, 120.86, 126.14, 128.26, 128.44, 133.37, 140.81 *(1 aliphatic carbon signal is not observed due to signal overlap)*. IR (neat): 3027, 2928, 2859, 1673, 1496, 1453, 1435,

1343, 1136, 1077, 1030, 918, 841, 800, 748, 697, 585, 497 cm^{-1}. HRMS (EI) calcd. for $C_{16}H_{18}$ $[M]^+$: 210.1403, found 210.1410.

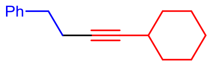

3p

(4-Cyclohexylbut-3-yn-1-yl)benzene (3p). 0.3 mmol scale, $NiCl_2·6H_2O$ (0.03 mmol, 7.1 mg), **L4** (0.03 mmol, 7.9 mg), anhydrous K_3PO_4 (0.39 mmol, 82.8 mg), phenethylpyridinium salt **1a** (0.3 mmol, 150.0 mg), ethynylcyclohexane (0.45 mmol, 48.7 mg) and tetrahydrofuran (1.5 mL) were stirred at 80 °C for 24 h. Then the reaction mixture was filtered through a short pad of silica gel and washed with dichloromethane. Purification of the crude product by column chromatography on silica gel (eluent: petroleum ether: dichloromethane = 100:1) afforded the title product in 83% yield (52.6 mg) as a colorless oil. ^1H NMR (400 MHz, $CDCl_3$): δ 1.21-1.30 (m, 3H), 1.34-1.41 (m, 2H), 1.48-1.53 (m, 1H), 1.63-1.70 (m, 2H), 1.73-1.76 (m ,2H), 2.29-2.33 (m, 1H), 2.44 (td, *J* = 2.0, 7.6 Hz, 2H), 2.79 (t, *J* = 7.6 Hz, 2H), 7.17-7.22 (m, 3H), 7.26-7.29 (m, 2H). ^{13}C NMR (100 MHz, $CDCl_3$): δ 21.04, 24.89, 25.92, 29.11, 33.04, 35.71, 79.27, 85.40, 126.08, 128.22, 128.51, 141.05. IR (neat): 3028, 2927, 2853, 1605, 1496, 1449, 1341, 1299, 1077, 1030, 889, 749, 698, 492 cm^{-1}. HRMS (EI) calcd. for $C_{16}H_{20}$ $[M]^+$: 212.1560, found 212.1556.

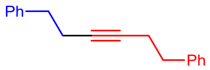

3q

1,6-Diphenylhex-3-yne (3q). 0.3 mmol scale, $NiCl_2·6H_2O$ (0.03 mmol, 7.1 mg), **L4** (0.03 mmol, 7.9 mg), anhydrous K_3PO_4 (0.39 mmol, 82.8 mg), phenethylpyridinium salt **1a** (0.3 mmol, 150.0 mg), but-3-yn-1-ylbenzene (0.45 mmol, 58.6 mg) and tetrahydrofuran (1.5 mL) were stirred at 80 °C for 24 h. Then the reaction mixture was filtered through a short pad of silica gel and washed with dichloromethane. Purification of the crude product by column chromatography on silica gel (eluent: petroleum ether: dichloromethane = 100:1 to 10:1, gradient) afforded the title product in 88% yield (62.0 mg) as a colorless oil. ^1H NMR (400 MHz, $CDCl_3$): δ 2.42 (t, *J* = 7.6 Hz, 4H), 2.77 (t, *J* = 7.6 Hz, 4H), 7.16-7.20 (m, 6H),

7.27 (t, J = 7.2 Hz, 4H). ^{13}C NMR (100 MHz, $CDCl_3$): δ 20.92, 35.43, 80.20, 126.12, 128.26, 128.41, 140.91. IR (neat): 3062, 3027, 2927, 2860, 1604, 1495, 1454, 1431, 1342, 1077, 1031, 747, 696, 567, 500 cm^{-1}. HRMS (EI) calcd. for $C_{18}H_{18}$ $[M]^+$: 234.1403, found 234.1401.

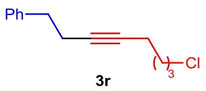

3r

(8-Chlorooct-3-yn-1-yl)benzene (3r). 0.3 mmol scale, $NiCl_2{\cdot}6H_2O$ (0.03 mmol, 7.1 mg), **L4** (0.03 mmol, 7.9 mg), anhydrous K_3PO_4 (0.39 mmol, 82.8 mg), phenethylpyridinium salt **1a** (0.3 mmol, 150.0 mg), 6-chlorohex-1-yne (0.45 mmol, 52.5 mg) and tetrahydrofuran (1.5 mL) were stirred at 80 °C for 24 h. Then the reaction mixture was filtered through a short pad of silica gel and washed with dichloromethane. Purification of the crude product by column chromatography on silica gel (eluent: petroleum ether: dichloromethane = 100:3) afforded the title product in 78% yield (51.7 mg) as a colorless oil. ^1H NMR (600 MHz, $CDCl_3$): δ 1.57-1.61 (m, 2H), 1.79-1.84 (m, 2H), 2.17 (t, J = 6.6 Hz, 2H), 2.44 (t, J = 7.2 Hz, 2H), 2.79 (t, J = 7.2 Hz, 2H), 3.51 (t, J = 6.6 Hz, 2H), 7.18-7.23 (m, 3H), 7.27-7.29 (m, 2H). ^{13}C NMR (151 MHz, $CDCl_3$): δ 17.94, 20.85, 26.04, 31.42, 35.42, 44.59, 80.01, 80.11, 126.13, 128.25, 128.41, 140.85. IR (neat): 3028, 2930, 2862, 1604, 1496, 1453, 1433, 1339, 1301, 1275, 1077, 1030, 748, 698, 650, 578, 507 cm^{-1}. HRMS (EI) calcd. for $C_{14}H_{17}Cl$ $[M]^+$: 220.1013, found 220.1009.

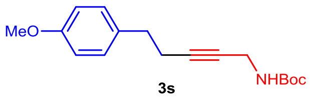

3s

***tert*-Butyl (5-(4-methoxyphenyl)pent-2-yn-1-yl)carbamate (3s).** 0.3 mmol scale, $NiCl_2{\cdot}6H_2O$ (0.03 mmol, 7.1 mg), **L4** (0.03 mmol, 7.9 mg), anhydrous K_3PO_4 (0.39 mmol, 82.8 mg), *p*-methoxyphenethylpyridinium salt **1b** (0.3 mmol, 158.8 mg), *tert*-butyl prop-2-yn-1-ylcarbamate (0.45 mmol, 69.8 mg) and tetrahydrofuran (1.5 mL) were stirred at 80 °C for 24 h. Then the reaction mixture was filtered through a short pad of silica gel and washed with ethyl acetate. Purification of the crude product by column chromatography on silica gel (eluent: petroleum ether: ethyl acetate = 20:1) afforded the

title product in 90% yield (78.4 mg) as a yellow oil. ^{1}H NMR (400 MHz, $CDCl_3$): δ 1.50 (s, 9H), 2.39-2.42 (m, 2H), 2.73 (t, J = 7.6 Hz, 2H), 3.78 (s, 3H), 3.87 (s, 2H), 4.70 (br, 1H), 6.83 (d, J = 8.4 Hz, 2H), 7.11 (d, J = 8.4 Hz, 2H). ^{13}C NMR (100 MHz, $CDCl_3$): δ 21.05, 28.28, 30.69, 34.05, 55.12, 76.77, 79.62, 82.84, 113.68, 129.29, 132.66, 155.24, 158.03. IR (neat): 3357, 2989, 2943, 1675, 1612, 1509, 1459, 1446, 1389, 1363, 1303, 1266, 1243, 1181, 1165, 1108, 1050, 1029, 963, 873, 851, 820, 805, 782, 765, 753, 714, 607, 561, 544, 520, 464, 433 cm^{-1}. HRMS (ESI) calcd. for $C_{17}H_{23}NNaO_3$ $[M+Na]^+$: 312.1570, found 312.1570.

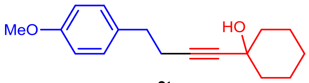

3t

1-(4-(4-Methoxyphenyl)but-1-yn-1-yl)cyclohexan-1-ol (3t). 0.3 mmol scale, $NiCl_2·6H_2O$ (0.03 mmol, 7.1 mg), **L4** (0.03 mmol, 7.9 mg), anhydrous K_3PO_4 (0.39 mmol, 82.8 mg), *p*-methoxyphenethylpyridinium salt **1b** (0.3 mmol, 158.8 mg), 1-ethynylcyclohexan-1-ol (0.45 mmol, 55.9 mg) and tetrahydrofuran (1.5 mL) were stirred at 80 °C for 24 h. Then the reaction mixture was filtered through a short pad of silica gel and washed with ethyl acetate. Purification of the crude product by column chromatography on silica gel (eluent: petroleum ether: ethyl acetate = 15:1) afforded the title product in 88% yield (68.1 mg) as a white solid. ^{1}H NMR (400 MHz, $CDCl_3$): δ 1.17-1.26 (m, 1H), 1.40-1.55 (m, 5H), 1.60-1.65 (m, 2H), 1.81-1.85 (m, 2H), 2.08 (br, 1H), 2.46 (t, J = 7.6 Hz, 2H), 2.76 (t, J = 7.6 Hz, 2H), 3.77 (s, 3H), 6.81-6.84 (m, 2H), 7.13 (d, J = 8.8 Hz, 2H). ^{13}C NMR (100 MHz, $CDCl_3$): δ 21.06, 23.26, 25.15, 34.22, 40.10, 55.17, 68.64, 83.86, 84.67, 113.65, 129.36, 132.77, 158.01. IR (neat): 3404, 2931, 2856, 1612, 1585, 1512, 1445, 1340, 1300, 1243, 1177, 1132, 1108, 1057, 1034, 962, 904, 867, 821, 755, 700, 549, 519, 431 cm^{-1}. HRMS (ESI) calcd. for $C_{17}H_{22}NaO_2$ $[M+Na]^+$: 281.1512, found 281.1511.

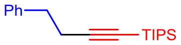

3u

Triisopropyl(4-phenylbut-1-yn-1-yl)silane (3u). 0.3 mmol scale, $NiCl_2·6H_2O$ (0.03 mmol, 7.1 mg), **L4** (0.03 mmol, 7.9 mg), anhydrous K_3PO_4 (0.39 mmol, 82.8 mg),

phenethylpyridinium salt **1a** (0.3 mmol, 150.0 mg), ethynyltriisopropylsilane (0.45 mmol, 82.1 mg) and tetrahydrofuran (1.5 mL) were stirred at 80 °C for 24 h. Then the reaction mixture was filtered through a short pad of silica gel and washed with dichloromethane. Purification of the crude product by column chromatography on silica gel (eluent: petroleum ether: dichloromethane = 100:1) afforded the title product in 87% yield (75.2 mg) as a colorless oil. ^{1}H NMR (400 MHz, $CDCl_3$): δ 0.96-1.07 (m, 21H), 2.54 (t, *J* = 7.6 Hz, 2H), 2.83 (t, *J* = 7.6 Hz, 2H), 7.16-7.29 (m, 5H). ^{13}C NMR (100 MHz, $CDCl_3$): δ 11.27, 18.58, 22.04, 35.27, 80.97, 108.13, 126.17, 128.28, 128.51, 140.65. IR (neat): 3029, 2942, 2864, 2172, 1605, 1497, 1463, 1383, 1366, 1337, 1242, 1076, 1042, 995, 919, 883, 746, 697, 675, 660, 640, 620, 577, 530, 512, 457, 419 cm^{-1}. HRMS (EI) calcd. for $C_{19}H_{30}Si$ $[M]^+$: 286.2111, found 286.2109.

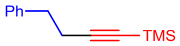

3v

Trimethyl(4-phenylbut-1-yn-1-yl)silane (3v). 0.3 mmol scale, $NiCl_2·6H_2O$ (0.03 mmol, 7.1 mg), **L4** (0.03 mmol, 7.9 mg), anhydrous K_3PO_4 (0.39 mmol, 82.8 mg), phenethylpyridinium salt **1a** (0.3 mmol, 150.0 mg), ethynyltrimethylsilane (0.45 mmol, 44.2 mg) and tetrahydrofuran (1.5 mL) were stirred at 80 °C for 24 h. Then the reaction mixture was filtered through a short pad of silica gel and washed with dichloromethane. Purification of the crude product by column chromatography on silica gel (eluent: petroleum ether: dichloromethane = 100:1) afforded the title product in 75% yield (45.4 mg) as a colorless oil. ^{1}H NMR (400 MHz, $CDCl_3$): δ 0.10 (s, 9H), 2.45 (t, *J* = 7.6 Hz, 2H), 2.79 (t, *J* = 7.6 Hz, 2H), 7.16-7.19 (m, 3H), 7.22-7.26 (m, 2H). ^{13}C NMR (100 MHz, $CDCl_3$): δ 0.06, 22.17, 35.09, 85.23, 106.64, 126.25, 128.29, 128.51, 140.60. IR (neat): 3852, 3746, 3029, 2958, 2176, 1603, 1497, 1454, 1339, 1250, 1077, 1042, 996, 841, 760, 698, 646, 514, 436, 415 cm^{-1}. HRMS (EI) calcd. for $C_{13}H_{18}Si$ $[M]^+$: 202.1172, found 202.1175.

General procedure C: Sonogashira coupling of primary alkylpyridinium salts

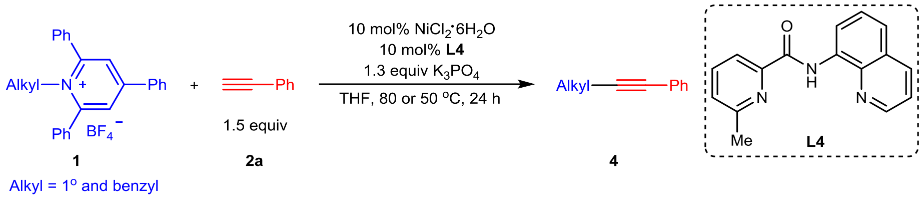

In a nitrogen-filled glovebox, $NiCl_2 \cdot 6H_2O$ (0.03 mmol, 7.1 mg), **L4** (0.03 mmol, 7.9 mg), anhydrous K_3PO_4 (0.39 mmol, 82.8 mg), primary alkylpyridinium salt (0.3 mmol) and tetrahydrofuran (1.5 mL) were successively added to an oven-dried sealable Schlenk tube (10.0 mL) followed by addition of phenylacetylene (0.45 mmol, 46.0 mg) via microliter syringe. Then the tube was securely sealed and taken outside the glovebox. And it was immersed into an oil bath preheated at 80 or 50 °C. After stirring for 24 h, the reaction mixture was cooled to room temperature and filtered through a short pad of silica gel. Then the filter cake was washed with dichloromethane or ethyl acetate. The resulting solution was concentrated under vacuum and the residue was purified by column chromatography on silica gel.

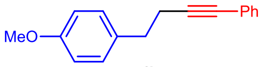

4b

1-Methoxy-4-(4-phenylbut-3-yn-1-yl)benzene (4b). 0.3 mmol scale, $NiCl_2 \cdot 6H_2O$ (0.03 mmol, 7.1 mg), **L4** (0.03 mmol, 7.9 mg), anhydrous K_3PO_4 (0.39 mmol, 82.8 mg), *p*-methoxyphenethylpyridinium salt **1b** (0.3 mmol, 158.8 mg), phenylacetylene (0.45 mmol, 46.0 mg) and tetrahydrofuran (1.5 mL) were stirred at 80 °C for 24 h. Then the reaction mixture was filtered through a short pad of silica gel and washed with dichloromethane. Purification of the crude product by column chromatography on silica gel (eluent: petroleum ether: dichloromethane = 20:1) afforded the title product in 93% yield (66.0 mg) as a light-yellow solid. ^{1}H NMR (400 MHz, $CDCl_3$): δ 2.64 (t, *J* = 7.6 Hz, 2H), 2.85 (t, *J* = 7.6 Hz, 2H), 3.76 (s, 3H), 6.84 (d, *J* = 8.4 Hz, 2H), 7.17 (d, *J* = 8.4 Hz, 2H), 7.24-7.28 (m, 3H), 7.36-7.38 (m, 2H). ^{13}C NMR (100 MHz, $CDCl_3$): δ 21.91, 34.25, 55.17, 81.25, 89.60, 113.73, 123.84, 127.55, 128.14, 129.43, 131.47, 132.80, 158.09. IR (neat): 3045, 3017, 2924, 2857, 2835, 1885, 1612, 1586, 1511, 1489, 1462, 1441, 1424, 1320, 1301, 1241,

1178, 1155, 1107, 1071, 1035, 1004, 917, 829, 815, 757, 714, 691, 637, 566, 535, 518, 489, 412 cm^{-1}. HRMS (ESI) calcd. for $C_{17}H_{17}O$ $[M+H]^+$: 237.1274, found 237.1274.

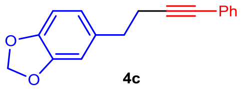

4c

5-(4-Phenylbut-3-yn-1-yl)benzo[*d*][1,3]dioxole (4c). 0.3 mmol scale, $NiCl_2 \cdot 6H_2O$ (0.03 mmol, 7.1 mg), **L4** (0.03 mmol, 7.9 mg), anhydrous K_3PO_4 (0.39 mmol, 82.8 mg), 1-(2-(benzo[*d*][1,3]dioxol-5-yl)ethyl)-2,4,6-triphenylpyridin-1-ium tetrafluoroborate **1c** (0.3 mmol, 163.0 mg), phenylacetylene (0.45 mmol, 46.0 mg) and tetrahydrofuran (1.5 mL) were stirred at 80 °C for 24 h. Then the reaction mixture was filtered through a short pad of silica gel and washed with dichloromethane. Purification of the crude product by column chromatography on silica gel (eluent: petroleum ether to petroleum ether: dichloromethane = 100:3, gradient) afforded the title product in 91% yield (68.3 mg) as a colorless oil. ^{1}H NMR (600 MHz, $CDCl_3$): δ 2.55 (t, *J* = 7.2 Hz, 2H), 2.74 (t, *J* = 7.2 Hz, 2H), 5.83 (s, 2H), 6.62-6.68 (m, 3H), 7.14-7.18 (m, 3H), 7.28-7.29 (m, 2H). ^{13}C NMR (151 MHz, $CDCl_3$): δ 21.95, 34.87, 81.39, 89.37, 100.78, 108.11, 108.96, 121.36, 123.78, 127.60, 128.17, 131.49, 134.52, 145.97, 147.53. IR (neat): 2900, 1598, 1502, 1488, 1441, 1363, 1337, 1243, 1188, 1122, 1097, 1070, 1038, 935, 890, 856, 806, 780, 754, 725, 690, 634, 603, 549, 525, 473, 421 cm^{-1}. HRMS (ESI) calcd. for $C_{17}H_{15}O_2$ $[M+H]^+$: 251.1067, found 251.1067.

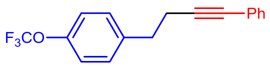

4d

1-(4-Phenylbut-3-yn-1-yl)-4-(trifluoromethoxy)benzene (4d). 0.3 mmol scale, $NiCl_2 \cdot 6H_2O$ (0.03 mmol, 7.1 mg), **L4** (0.03 mmol, 7.9 mg), anhydrous K_3PO_4 (0.39 mmol, 82.8 mg), 2,4,6-triphenyl-1-(4-(trifluoromethoxy)phenethyl)pyridin-1-ium tetrafluoroborate **1d** (0.3 mmol, 175.0 mg), phenylacetylene (0.45 mmol, 46.0 mg) and tetrahydrofuran (1.5 mL) were stirred at 80 °C for 24 h. Then the reaction mixture was filtered through a short pad of silica gel and washed with dichloromethane. Purification of the crude product by column chromatography on silica gel (eluent: petroleum ether: dichloromethane = 100:1) afforded the title product in 94% yield (81.5 mg) as a white solid.

^{1}H NMR (400 MHz, $CDCl_3$): δ 2.68 (t, J = 7.2 Hz, 2H), 2.90 (t, J = 7.2 Hz, 2H), 7.15 (d, J = 8.4 Hz, 2H), 7.25-7.28 (m, 5H), 7.34-7.36 (m, 2H). ^{13}C NMR (100 MHz, $CDCl_3$): δ 21.49, 34.34, 81.71, 88.90, 120.53 (q, J = 255.0 Hz), 120.89, 123.65, 127.73, 128.22, 129.85, 131.48, 139.35, 147.78 (q, J = 2.1 Hz). IR (neat): 3036, 2937, 2364, 2327, 1594, 1506, 1490, 1444, 1211, 1155, 1105, 1070, 1018, 919, 848, 781, 758, 692, 671, 614, 527, 460 cm^{-1}. HRMS (ESI) calcd. for $C_{17}H_{14}F_3O$ $[M+H]^+$: 291.0991, found 291.0991.

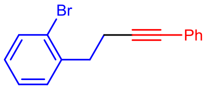

4e

1-Bromo-2-(4-phenylbut-3-yn-1-yl)benzene (4e). 0.3 mmol scale, $NiCl_2·6H_2O$ (0.03 mmol, 7.1 mg), **L4** (0.03 mmol, 7.9 mg), anhydrous K_3PO_4 (0.39 mmol, 82.8 mg), 1-(2-bromophenethyl)-2,4,6-triphenylpyridin-1-ium tetrafluoroborate **1e** (0.3 mmol, 173.5 mg), phenylacetylene (0.45 mmol, 46.0 mg) and tetrahydrofuran (1.5 mL) were stirred at 80 °C for 24 h. Then the reaction mixture was filtered through a short pad of silica gel and washed with dichloromethane. Purification of the crude product by column chromatography on silica gel (eluent: petroleum ether: dichloromethane = 100:1) afforded the title product in 90% yield (76.7 mg) as a colorless oil. ^{1}H NMR (600 MHz, $CDCl_3$): δ 2.72 (t, J = 7.2 Hz, 2H), 3.04 (t, J = 7.2 Hz, 2H), 7.07 (t, J = 7.2 Hz, 1H), 7.21-7.27 (m, 4H), 7.32 (d, J = 7.2 Hz, 1H), 7.36-7.37 (m, 2H), 7.53 (d, J = 7.8 Hz, 1H). ^{13}C NMR (151 MHz, $CDCl_3$): δ 19.80, 35.34, 81.51, 88.95, 123.73, 124.36, 127.31, 127.61, 128.08, 128.15, 130.83, 131.49, 132.77, 139.68. IR (neat): 3056, 2936, 1597, 1568, 1489, 1471, 1440, 1340, 1249, 1159, 1116, 1070, 1025, 913, 842, 748, 690, 657, 585, 534, 526, 446 cm^{-1}. HRMS (EI) calcd. for $C_{16}H_{13}Br$ $[M]^+$: 284.0195, found 284.0202.

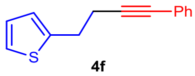

4f

2-(4-Phenylbut-3-yn-1-yl)thiophene (4f). 0.3 mmol scale, $NiCl_2·6H_2O$ (0.03 mmol, 7.1 mg), **L4** (0.03 mmol, 7.9 mg), anhydrous K_3PO_4 (0.39 mmol, 82.8 mg), 2,4,6-triphenyl-1-(2-(thiophen-2-yl)ethyl)pyridin-1-ium tetrafluoroborate **1f** (0.3 mmol, 151.6 mg), phenylacetylene (0.45 mmol, 46.0 mg) and tetrahydrofuran (1.5 mL) were stirred at 80 °C

for 24 h. Then the reaction mixture was filtered through a short pad of silica gel and washed with dichloromethane. Purification of the crude product by column chromatography on silica gel (eluent: petroleum ether: dichloromethane = 100:1) afforded the title product in 94% yield (60.1 mg) as a colorless oil. ^{1}H NMR (400 MHz, $CDCl_3$): δ 2.73 (t, J = 7.6 Hz, 2H), 3.12 (t, J = 7.6 Hz, 2H), 6.89-6.94 (m, 2H), 7.13 (dd, J = 1.2, 5.2 Hz, 1H), 7.25-7.29 (m, 3H), 7.37-7.40 (m, 2H). ^{13}C NMR (100 MHz, $CDCl_3$): δ 22.10, 29.40, 81.70, 88.90, 123.55, 123.68, 124.80, 126.69, 127.68, 128.17, 131.51, 143.09. IR (neat): 2925, 1598, 1489, 1440, 1339, 1252, 1070, 1041, 913, 848, 823, 755, 690, 529, 500, 436, 416, 406 cm^{-1}. HRMS (ESI) calcd. for $C_{14}H_{13}S$ $[M+H]^+$: 213.0732, found 213.0730.

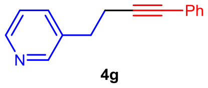

4g

3-(4-Phenylbut-3-yn-1-yl)pyridine (4g). 0.3 mmol scale, $NiCl_2{\cdot}6H_2O$ (0.03 mmol, 7.1 mg), **L4** (0.03 mmol, 7.9 mg), anhydrous K_3PO_4 (0.39 mmol, 82.8 mg), 2,4,6-triphenyl-1-(2-(pyridin-3-yl)ethyl)pyridin-1-ium tetrafluoroborate **1g** (0.3 mmol, 150.1 mg), phenylacetylene (0.45 mmol, 46.0 mg) and *N*, *N*-dimethylformamide (1.5 mL) were stirred at 80 °C for 24 h. Then the reaction mixture was extracted with ethyl acetate, washed with water and brine, and dried over anhydrous Na_2SO_4. Purification of the crude product by column chromatography on silica gel (eluent: petroleum ether: ethyl acetate = 5:1) afforded the title product in 92% yield (57.2 mg) as a yellow oil. ^{1}H NMR (400 MHz, $CDCl_3$): δ 2.70 (t, J = 7.2 Hz, 2H), 2.90 (t, J = 7.2 Hz, 2H), 7.24-7.29 (m, 4H) 7.34-7.36 (m, 2H), 7.60 (d, J = 7.6 Hz, 1H), 8.50 (s, 1H), 8.57 (s, 1H). ^{13}C NMR (100 MHz, $CDCl_3$): δ 21.23, 32.06, 81.95, 88.35, 123.26, 123.40, 127.70, 128.14, 131.40, 135.83, 136.00, 147.69, 149.91. IR (neat): 3030, 2929, 1597, 1575, 1490, 1479, 1442, 1423, 1343, 1191, 1157, 1104, 1070, 1028, 916, 797, 755, 712, 691, 629, 604, 531, 500 cm^{-1}. HRMS (ESI) calcd. for $C_{15}H_{14}N$ $[M+H]^+$: 208.1121, found 208.1126.

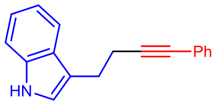

4h

3-(4-Phenylbut-3-yn-1-yl)-1*H*-indole (4h). 0.3 mmol scale, $NiCl_2{\cdot}6H_2O$ (0.03 mmol, 7.1 mg), **L4** (0.03 mmol, 7.9 mg), anhydrous K_3PO_4 (0.39 mmol, 82.8 mg), 1-(2-(1*H*-indol-3-yl)-ethyl)-2,4,6-triphenylpyridin-1-ium tetrafluoroborate **1h** (0.3 mmol, 161.5 mg), phenylacetylene (0.45 mmol, 46.0 mg) and *N*, *N*-dimethylformamide (1.5 mL) were stirred at 80 °C for 24 h. Then the reaction mixture was extracted with ethyl acetate, washed with water and brine, and dried over anhydrous Na_2SO_4. Purification of the crude product by column chromatography on silica gel (eluent: petroleum ether: ethyl acetate = 20:1) afforded the title product in 76% yield (56.1 mg) as a yellow oil. ^{1}H NMR (600 MHz, $CDCl_3$): δ 2.76 (t, *J* = 7.2 Hz, 2H), 3.06 (t, *J* = 7.2 Hz, 2H), 7.00 (s, 1H), 7.11 (t, *J* = 7.8 Hz, 1H), 7.18 (t, *J* = 8.4 Hz, 1H), 7.22-7.29 (m, 4H), 7.37 (d, *J* = 6.6 Hz, 2H), 7.63 (d, *J* = 7.8 Hz, 1H), 7.79 (br, 1H). ^{13}C NMR (151 MHz, $CDCl_3$): δ 20.77, 24.81, 81.06, 90.37, 111.09, 115.15, 118.75, 119.25, 121.60, 121.93, 123.91, 127.23, 127.54, 128.17, 131.51, 136.14. IR (neat): 3396, 3048, 2939, 2909, 2847, 1599, 1488, 1456, 1442, 1424, 1354, 1334, 1302, 1245, 1227, 1169, 1091, 1069, 1008, 910, 823, 774, 748, 686, 594, 583, 547, 524, 503, 466, 449, 424 cm^{-1}. HRMS (ESI) calcd. for $C_{18}H_{16}N$ $[M+H]^+$: 246.1277, found 246.1274.

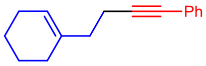

4i

(4-(Cyclohex-1-en-1-yl)but-1-yn-1-yl)benzene (4i). 0.3 mmol scale, $NiCl_2{\cdot}6H_2O$ (0.03 mmol, 7.1 mg), **L4** (0.03 mmol, 7.9 mg), anhydrous K_3PO_4 (0.39 mmol, 82.8 mg), 1-(2-(cyclohex-1-en-1-yl)ethyl)-2,4,6-triphenylpyridin-1-ium tetrafluoroborate **1i** (0.3 mmol, 151.0 mg), phenylacetylene (0.45 mmol, 46.0 mg) and tetrahydrofuran (1.5 mL) were stirred at 80 °C for 24 h. Then the reaction mixture was filtered through a short pad of silica gel and washed with dichloromethane. Purification of the crude product by column chromatography on silica gel (eluent: petroleum ether) afforded the title product in 90% yield (56.6 mg) as a colorless oil. ^{1}H NMR (400 MHz, $CDCl_3$): δ 1.53-1.66 (m, 4H), 1.97-2.01 (m, 4H), 2.24 (t, *J* = 7.6 Hz, 2H), 2.48 (t, *J* = 7.6 Hz, 2H), 5.50 (s, 1H), 7.22-7.29 (m, 3H), 7.37-7.39 (m, 2H). ^{13}C NMR (100 MHz, $CDCl_3$): δ 18.41, 22.43, 22.91, 25.21, 28.14, 37.08, 80.77, 90.25, 122.05, 124.07, 127.43, 128.14, 131.49, 136.26. IR (neat): 3054, 2924, 2856, 2834, 1598, 1490, 1441, 1333, 1270, 1133, 1070, 913, 801, 754, 691, 527, 452,

431 cm^{-1}. HRMS (EI) calcd. for $C_{16}H_{18}$ [M]$^{+}$: 210.1403, found 210.1398.

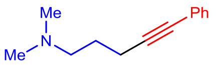

4j

***N*, *N*-Dimethyl-5-phenylpent-4-yn-1-amine (4j).** 0.3 mmol scale, $NiCl_2·6H_2O$ (0.03 mmol, 7.1 mg), **L4** (0.03 mmol, 7.9 mg), anhydrous K_3PO_4 (0.39 mmol, 82.8 mg), 1-(3-(dimethylamino)propyl)-2,4,6-triphenylpyridin-1-ium tetrafluoroborate **1j** (0.3 mmol, 144.1 mg), phenylacetylene (0.45 mmol, 46.0 mg) and tetrahydrofuran (1.5 mL) were stirred at 80 °C for 24 h. Then the reaction mixture was filtered through a short pad of silica gel and washed with methanol. Purification of the crude product by column chromatography on silica gel (eluent: dichloromethane: acetone: methanol = 15:1:1) afforded the title product in 80% yield (45.2 mg) as a yellow oil. ^{1}H NMR (600 MHz, $CDCl_3$): δ 1.80 (br, 2H), 2.37 (br, 5H), 2.47 (s, 3H), 2.56 (br, 2H), 7.27 (br, 3H), 7.39 (d, *J* = 5.4 Hz, 2H). ^{13}C NMR (151 MHz, $CDCl_3$): δ 17.20, 26.32, 44.74, 58.22, 80.97, 89.28, 123.74, 127.53, 128.12, 131.46. IR (neat): 3401, 2948, 2677, 1598, 1490, 1466, 1442, 1341, 1070, 1029, 975, 828, 756, 692, 557, 526, 416 cm^{-1}. HRMS (ESI) calcd. for $C_{13}H_{18}N$ [M+H]$^{+}$: 188.1434, found 188.1434.

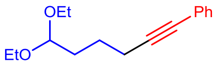

4k

(6,6-Diethoxyhex-1-yn-1-yl)benzene (4k). 0.3 mmol scale, $NiCl_2·6H_2O$ (0.03 mmol, 7.1 mg), **L4** (0.03 mmol, 7.9 mg), anhydrous K_3PO_4 (0.39 mmol, 82.8 mg), 1-(4,4-diethoxybutyl)-2,4,6-triphenylpyridin-1-ium tetrafluoroborate **1k** (0.3 mmol, 161.8 mg), phenylacetylene (0.45 mmol, 46.0 mg) and tetrahydrofuran (1.5 mL) were stirred at 80 °C for 24 h. Then the reaction mixture was filtered through a short pad of silica gel and washed with dichloromethane. Purification of the crude product by column chromatography on silica gel (eluent: petroleum ether: ethyl acetate = 100:1) afforded the title product in 83% yield (61.3 mg) as a light-yellow oil. ^{1}H NMR (400 MHz, $CDCl_3$): δ 1.12-1.21 (m, 6H), 1.56-1.64 (m, 2H), 1.69-1.74 (m, 2H), 2.36 (t, *J* = 7.2 Hz, 2H), 3.39-3.46 (m, 2H), 3.54-3.62 (m, 2H), 4.46 (t, *J* = 6.0 Hz, 1H), 7.18-7.22 (m, 3H),

7.30-7.32 (m, 2H). ^{13}C NMR (100 MHz, $CDCl_3$): δ 15.28, 19.20, 23.96, 32.71, 60.94, 80.86, 89.78, 102.48, 123.91, 127.47, 128.12, 131.47. IR (neat): 2974, 2928, 2875, 1599, 1490, 1442, 1374, 1343, 1128, 1059, 991, 913, 755, 691, 526 cm^{-1}. HRMS (ESI) calcd. for $C_{16}H_{22}NaO_2$ [M+Na]$^+$: 269.1512, found 269.1507.

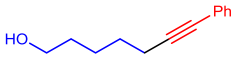

4l

7-Phenylhept-6-yn-1-ol (4l). 0.3 mmol scale, $NiCl_2·6H_2O$ (0.03 mmol, 7.1 mg), **L4** (0.03 mmol, 7.9 mg), anhydrous K_3PO_4 (0.39 mmol, 82.8 mg), 1-(5-hydroxypentyl)-2,4,6-triphenylpyridin-1-ium tetrafluoroborate **1l** (0.3 mmol, 144.4 mg), phenylacetylene (0.45 mmol, 46.0 mg) and tetrahydrofuran (1.5 mL) were stirred at 80 °C for 24 h. Then the reaction mixture was filtered through a short pad of silica gel and washed with ethyl acetate. Purification of the crude product by column chromatography on silica gel (eluent: petroleum ether: ethyl acetate = 5:1) afforded the title product in 91% yield (51.3 mg) as a light-yellow oil. ^{1}H NMR (400 MHz, $CDCl_3$): δ 1.49-1.66 (m, 6H), 1.93 (br, 1H), 2.41 (t, J = 6.8 Hz, 2H), 3.64 (t, J = 6.4 Hz, 2H), 7.25-7.29 (m, 3H), 7.38-7.39 (m, 2H). ^{13}C NMR (100 MHz, $CDCl_3$): δ 19.27, 24.98, 28.41, 32.14, 62.63, 80.68, 90.02, 123.88, 127.44, 128.11, 131.44. IR (neat): 3324, 2934, 2860, 1598, 1489, 1441, 1331, 1070, 1051, 914, 754, 691, 525, 421 cm^{-1}. HRMS (ESI) calcd. for $C_{13}H_{16}NaO$ [M+Na]$^+$: 211.1093, found 211.1093.

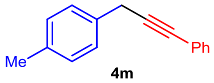

4m

1-Methyl-4-(3-phenylprop-2-yn-1-yl)benzene (4m). 0.3 mmol scale, $NiCl_2·6H_2O$ (0.03 mmol, 7.1 mg), **L4** (0.03 mmol, 7.9 mg), anhydrous K_3PO_4 (0.39 mmol, 82.8 mg), 1-(4-methylbenzyl)-2,4,6-triphenylpyridin-1-ium tetrafluoroborate **1m** (0.3 mmol, 150.0 mg), phenylacetylene (0.45 mmol, 46.0 mg) and tetrahydrofuran (1.5 mL) were stirred at 50 °C for 24 h. Then the reaction mixture was filtered through a short pad of silica gel and washed with dichloromethane. Purification of the crude product by column chromatography on silica gel (eluent: petroleum ether) afforded the title product in 86%

yield (53.5 mg) as a light-yellow oil. ^{1}H NMR (400 MHz, $CDCl_3$): δ 2.24 (s, 3H), 3.69 (s, 2H), 7.05 (d, J = 8.0 Hz, 2H), 7.18-7.22 (m, 5H), 7.34-7.36 (m, 2H). ^{13}C NMR (100 MHz, $CDCl_3$): δ 20.99, 25.29, 82.42, 87.83, 123.74, 127.72, 127.81, 128.18, 129.19, 131.60, 133.68, 136.14. IR (neat): 3024, 2921, 2198, 1690, 1638, 1602, 1513, 1490, 1450, 1416, 1317, 1285, 1210, 1172, 1115, 1070, 1022, 913, 808, 755, 712, 690, 663, 595, 571, 525, 509, 477, 408 cm^{-1}. HRMS (EI) calcd. for $C_{16}H_{14}$ $[M]^+$: 206.1090, found 206.1092.

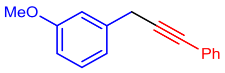

4n

1-Methoxy-3-(3-phenylprop-2-yn-1-yl)benzene (4n). 0.3 mmol scale, $NiCl_2·6H_2O$ (0.03 mmol, 7.1 mg), **L4** (0.03 mmol, 7.9 mg), anhydrous K_3PO_4 (0.39 mmol, 82.8 mg), 1-(3-methoxybenzyl)-2,4,6-triphenylpyridin-1-ium tetrafluoroborate **1n** (0.3 mmol, 154.6 mg), phenylacetylene (0.45 mmol, 46.0 mg) and tetrahydrofuran (1.5 mL) were stirred at 50 °C for 24 h. Then the reaction mixture was filtered through a short pad of silica gel and washed with dichloromethane. Purification of the crude product by column chromatography on silica gel (eluent: petroleum ether) afforded the title product in 86% yield (57.5 mg) as a light-yellow oil. ^{1}H NMR (400 MHz, $CDCl_3$): δ 3.80 (s, 5H), 6.78-6.80 (m, 1H), 6.99 (d, J = 6.0 Hz, 2H), 7.22-7.29 (m, 4H), 7.43-7.45 (m, 2H). ^{13}C NMR (100 MHz, $CDCl_3$): δ 25.70, 55.15, 82.69, 87.34, 112.03, 113.70, 120.31, 123.63, 127.80, 128.20, 129.48, 131.60, 138.29, 159.80. IR (neat): 2937, 2835, 1694, 1599, 1585, 1489, 1465, 1453, 1435, 1318, 1283, 1258, 1145, 1070, 1047, 996, 916, 877, 776, 756, 740, 714, 690, 548, 527, 512, 480, 444 cm^{-1}. HRMS (ESI) calcd. for $C_{16}H_{15}O$ $[M+H]^+$: 223.1117, found 223.1114.

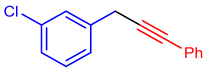

4o

1-Chloro-3-(3-phenylprop-2-yn-1-yl)benzene (4o). 0.3 mmol scale, $NiCl_2·6H_2O$ (0.03 mmol, 7.1 mg), **L4** (0.03 mmol, 7.9 mg), anhydrous K_3PO_4 (0.39 mmol, 82.8 mg), 1-(3-chlorobenzyl)-2,4,6-triphenylpyridin-1-ium tetrafluoroborate **1o** (0.3 mmol, 156.0 mg), phenylacetylene (0.45 mmol, 46.0 mg) and tetrahydrofuran (1.5 mL) were stirred at

50 °C for 24 h. Then the reaction mixture was filtered through a short pad of silica gel and washed with dichloromethane. Purification of the crude product by column chromatography on silica gel (eluent: petroleum ether) afforded the title product in 91% yield (62.0 mg) as a colorless oil. ^{1}H NMR (400 MHz, $CDCl_3$): δ 3.79 (s, 2H), 7.22 (d, J = 8.0 Hz, 2H), 7.25-7.32 (m, 4H), 7.41 (s, 1H), 7.44-7.46 (m, 2H). ^{13}C NMR (100 MHz, $CDCl_3$): δ 25.41, 83.15, 86.47, 123.36, 126.13, 126.86, 127.99, 128.12, 128.26, 129.73, 131.64, 134.36, 138.73. IR (neat): 3062, 2200, 1693, 1596, 1574, 1490, 1474, 1443, 1430, 1317, 1286, 1196, 1177, 1094, 1077, 1027, 999, 846, 775, 755, 712, 689, 527, 431, 418 cm^{-1}. HRMS (EI) calcd. for $C_{15}H_{11}Cl$ [M]$^+$: 226.0544, found 226.0542.

General procedure D: Sonogashira coupling of secondary alkylpyridinium salts

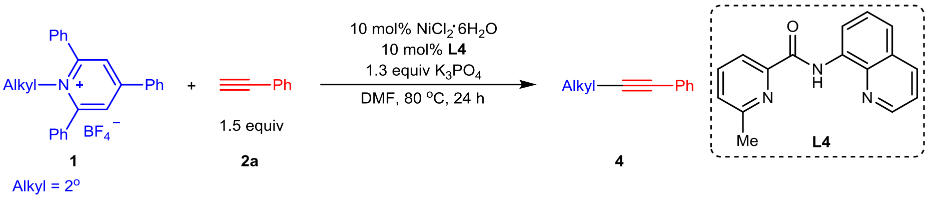

In a nitrogen-filled glovebox, $NiCl_2 \cdot 6H_2O$ (0.03 mmol, 7.1 mg), **L4** (0.03 mmol, 7.9 mg), anhydrous K_3PO_4 (0.39 mmol, 82.8 mg), secondary alkylpyridinium salt (0.3 mmol) and *N, N*-dimethylformamide (1.5 mL) were successively added to an oven-dried sealable Schlenk tube (10.0 mL) followed by addition of phenylacetylene (0.45 mmol, 46.0 mg) via microliter syringe. Then the tube was securely sealed and taken outside the glovebox. And it was immersed into an oil bath preheated at 80 °C. After stirring for 24 h, the reaction mixture was cooled to room temperature and quenched with water. Then it was extracted with ethyl acetate or diethyl ether, washed with water and brine, and dried over anhydrous Na_2SO_4. The resulting solution was concentrated under vacuum and the residue was purified by column chromatography on silica gel.

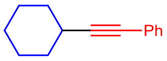

4p

(Cyclohexylethynyl)benzene (4p). 0.3 mmol scale, $NiCl_2 \cdot 6H_2O$ (0.03 mmol, 7.1 mg), **L4** (0.03 mmol, 7.9 mg), anhydrous K_3PO_4 (0.39 mmol, 82.8 mg), 1-cyclohexyl-2,4,6-

triphenylpyridin-1-ium tetrafluoroborate **1p** (0.3 mmol, 143.2 mg), phenylacetylene (0.45 mmol, 46.0 mg) and *N*, *N*-dimethylformamide (1.5 mL) were stirred at 80 °C for 24 h. Then the reaction mixture was extracted with diethyl ether, washed with water and brine, and dried over anhydrous Na_2SO_4. Purification of the crude product by column chromatography on silica gel (eluent: petroleum ether: dichloromethane = 100:1) afforded the title product in 92% yield (50.8 mg) as a colorless oil. ^{1}H NMR (400 MHz, $CDCl_3$): δ 1.31-1.40 (m, 3H), 1.49-1.58 (m, 3H), 1.72-1.77 (m, 2H), 1.86-1.89 (m, 2H), 2.55-2.61 (m, 1H), 7.21-7.28 (m, 3H),7.36-7.40 (m, 2H). ^{13}C NMR (100 MHz, $CDCl_3$): δ 24.87, 25.91, 29.63, 32.69, 80.49, 94.41, 124.12, 127.36, 128.11, 131.55. IR (neat): 3423, 2931, 2855, 1703, 1598, 1490, 1444, 1339, 1306, 1161, 1070, 1047, 1027, 933, 913, 889, 847, 818, 754, 690, 639, 585, 559, 535 cm^{-1}. HRMS (EI) calcd. for $C_{14}H_{16}$ $[M]^+$: 184.1247, found 184.1245.

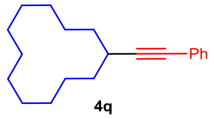

4q

(Phenylethynyl)cyclododecane (4q). 0.3 mmol scale, $NiCl_2{\cdot}6H_2O$ (0.03 mmol, 7.1 mg), **L4** (0.03 mmol, 7.9 mg), anhydrous K_3PO_4 (0.39 mmol, 82.8 mg), 1-cyclododecyl-2,4,6-triphenylpyridin-1-ium tetrafluoroborate **1q** (0.3 mmol, 168.5 mg), phenylacetylene (0.45 mmol, 46.0 mg) and *N*, *N*-dimethylformamide (1.5 mL) were stirred at 80 °C for 24 h. Then the reaction mixture was extracted with ethyl acetate, washed with water and brine, and dried over anhydrous Na_2SO_4. Purification of the crude product by column chromatography on silica gel (eluent: petroleum ether: dichloromethane = 100:1) afforded the title product in 86% yield (69.0 mg) as a colorless oil. ^{1}H NMR (400 MHz, $CDCl_3$): δ 1.36-1.50 (m, 16H), 1.54-1.57 (m, 4H), 1.59-1.73 (m, 2H), 2.65-2.71 (m, 1H), 7.20-7.28 (m, 3H), 7.35-7.39 (m, 2H). ^{13}C NMR (100 MHz, $CDCl_3$): δ 22.19, 23.37, 23.44, 23.80, 23.88, 27.42, 29.89, 80.19, 94.90, 124.20, 127.29, 128.09, 131.55. IR (neat): 2925, 2899, 2847, 2223, 1598, 1489, 1469, 1439, 1334, 1298, 1286, 1250, 1070, 1042, 1026, 964, 952, 910, 754, 722, 707, 690, 663, 558, 549, 523, 496, 478, 408 cm^{-1}. HRMS (EI) calcd. for $C_{20}H_{28}$ $[M]^+$: 268.2186, found 268.2190.

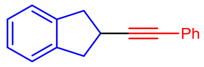

4r

2-(Phenylethynyl)-2,3-dihydro-1*H*-indene (4r). 0.3 mmol scale, $NiCl_2 \cdot 6H_2O$ (0.03 mmol, 7.1 mg), **L4** (0.03 mmol, 7.9 mg), anhydrous K_3PO_4 (0.39 mmol, 82.8 mg), 1-(2,3-dihydro-1*H*-inden-2-yl)-2,4,6-triphenylpyridin-1-ium tetrafluoroborate **1r** (0.3 mmol, 153.4 mg), phenylacetylene (0.45 mmol, 46.0 mg) and *N*, *N*-dimethylformamide (1.5 mL) were stirred at 80 °C for 24 h. Then the reaction mixture was extracted with ethyl acetate, washed with water and brine, and dried over anhydrous Na_2SO_4. Purification of the crude product by column chromatography on silica gel (eluent: petroleum ether) afforded the title product in 90% yield (59.0 mg) as a white solid. ^{1}H NMR (600 MHz, $CDCl_3$): δ 3.11 (dd, *J* = 8.4, 15.3 Hz, 2H), 3.29 (dd, *J* = 8.4, 15.3 Hz, 2H), 3.39-3.43 (m, 1H), 7.15 (d, *J* = 3.6 Hz, 2H), 7.20 (d, *J* = 4.2 Hz, 2H), 7.23-7.26 (m, 3H), 7.40 (d, *J* = 7.8 Hz, 2H). ^{13}C NMR (151 MHz, $CDCl_3$): δ 30.17, 40.29, 80.56, 92.99, 123.71, 124.30, 126.50, 127.63, 128.16, 131.58, 141.96. IR (neat): 3035, 2901, 2844, 1595, 1487, 1473, 1458, 1442, 1346, 1316, 1222, 1203, 1175, 1160, 1095, 1070, 1017, 1000, 931, 912, 859, 787, 752, 739, 689, 663, 603, 540, 532, 499, 478, 416 cm^{-1}. HRMS (EI) calcd. for $C_{17}H_{14}$ $[M]^+$: 218.1090, found 218.1091.

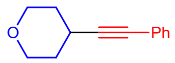

4s

4-(Phenylethynyl)tetrahydro-2*H*-pyran (4s). 0.3 mmol scale, $NiCl_2 \cdot 6H_2O$ (0.03 mmol, 7.1 mg), **L4** (0.03 mmol, 7.9 mg), anhydrous K_3PO_4 (0.39 mmol, 82.8 mg), 2,4,6-triphenyl-1-(tetrahydro-2*H*-pyran-4-yl)pyridin-1-ium tetrafluoroborate **1s** (0.3 mmol, 143.8 mg), phenylacetylene (0.45 mmol, 46.0 mg) and *N*, *N*-dimethylformamide (1.5 mL) were stirred at 80 °C for 24 h. Then the reaction mixture was extracted with ethyl acetate, washed with water and brine, and dried over anhydrous Na_2SO_4. Purification of the crude product by column chromatography on silica gel (eluent: petroleum ether: ethyl acetate = 100:1) afforded the title product in 91% yield (50.8 mg) as a light-yellow oil. ^{1}H NMR (600 MHz, $CDCl_3$): δ 1.73-1.78 (m, 2H), 1.89-1.91 (m, 2H), 2.84 (s, 1H), 3.52-3.56 (m, 2H), 3.94-3.95 (m, 2H), 7.27 (br, 3H), 7.40-7.41 (m, 2H). ^{13}C NMR (151 MHz, $CDCl_3$): δ

26.74, 32.24, 66.33, 81.48, 92.13, 123.57, 127.70, 128.17, 131.54. IR (neat): 2949, 2923, 2846, 1598, 1490, 1465, 1442, 1386, 1361, 1315, 1298, 1239, 1207, 1183, 1127, 1103, 1084, 1064, 1034, 1011, 980, 968, 945, 914, 859, 811, 755, 691, 596, 536, 503, 443, 420 cm^{-1}. HRMS (FI) calcd. for $C_{13}H_{14}O$ $[M]^+$: 186.1039, found 186.1038.

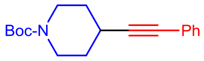

4t

***tert*-Butyl 4-(phenylethynyl)piperidine-1-carboxylate (4t).** 0.3 mmol scale, $NiCl_2{\cdot}6H_2O$ (0.03 mmol, 7.1 mg), **L4** (0.03 mmol, 7.9 mg), anhydrous K_3PO_4 (0.39 mmol, 82.8 mg), 1-(1-(*tert*-butoxycarbonyl)piperidin-4-yl)-2,4,6-triphenylpyridin-1-ium tetrafluoroborate **1t** (0.3 mmol, 173.5 mg), phenylacetylene (0.45 mmol, 46.0 mg) and *N*, *N*-dimethylformamide (1.5 mL) were stirred at 80 °C for 24 h. Then the reaction mixture was extracted with ethyl acetate, washed with water and brine, and dried over anhydrous Na_2SO_4. Purification of the crude product by column chromatography on silica gel (eluent: petroleum ether: ethyl acetate = 30:1) afforded the title product in 98% yield (83.5 mg) as a colorless oil. ^1H NMR (600 MHz, $CDCl_3$): δ 1.38 (s, 9H), 1.58-1.59 (m, 2H), 1.76 (br, 2H), 2.69-2.72 (m, 1H), 3.14-3.18 (m, 2H), 3.65 (br, 2H), 7.19-7.21 (m, 3H), 7.30-7.32 (m, 2H). ^{13}C NMR (151 MHz, $CDCl_3$): δ 27.47, 28.35, 31.32, 42.12, 79.34, 81.89, 91.69, 123.46, 127.67, 128.12, 131.48, 154.69. IR (neat): 2927, 2857, 1689, 1491, 1443, 1419, 1365, 1322, 1301, 1272, 1230, 1164, 1117, 1070, 1049, 1029, 1001, 988, 960, 936, 913, 864, 755, 691, 532, 461, 412 cm^{-1}. HRMS (ESI) calcd. for $C_{18}H_{23}NNaO_2$ $[M+Na]^+$: 308.1621, found 308.1621.

*However, when the reaction was conducted using tetrahydrofuran as the solvent (following **General procedure C**), a dramatic drop in reaction efficiency was observed and **4t** was obtained in only 74% yield.*

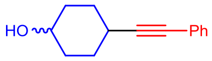

4u

4-(Phenylethynyl)cyclohexan-1-ol (4u). 0.3 mmol scale, $NiCl_2{\cdot}6H_2O$ (0.03 mmol, 7.1 mg), **L4** (0.03 mmol, 7.9 mg), anhydrous K_3PO_4 (0.39 mmol, 82.8 mg),

cis-1-(4-hydroxycyclohexyl)-2,4,6-triphenylpyridin-1-ium tetrafluoroborate **1u** (0.3 mmol, 148.0 mg), phenylacetylene (0.45 mmol, 46.0 mg) and *N*, *N*-dimethylformamide (1.5 mL) were stirred at 80 °C for 24 h. Then the reaction mixture was extracted with ethyl acetate, washed with water and brine, and dried over anhydrous Na_2SO_4. Purification of the crude product by column chromatography on silica gel (eluent: petroleum ether: ethyl acetate = 3:1) afforded a mixture of products (*trans*/*cis* = 2:1) in 94% yield (56.5 mg) as a light-yellow oil. ^{1}H NMR (600 MHz, $CDCl_3$): δ 1.29-1.35 (m, 2H), 1.48-1.54 (m, 2H), 1.62-1.64 (m, 1H), 1.76-1.77 (m, 1.5H), 1.90-1.91 (m, 1H), 1.99-2.07 (m, 6H), 2.45-2.48 (m, 1H, major), 2.78 (s, 0.5H, minor), 3.65-3.68 (m, 1.5H), 7.26 (br, 4.5H), 7.38 (d, *J* = 6.0 Hz, 2H, major), 7.40 (d, *J* = 6.6 Hz, 1H, minor). ^{13}C NMR (151 MHz, $CDCl_3$): δ 27.39 (minor), 28.60 (major), 28.98 (major), 30.37 (minor), 31.59 (major), 34.03 (minor), 68.85 (minor), 69.28 (major), 80.51 (major), 81.62 (minor), 92.86 (minor), 93.20 (major), 123.73 (major), 123.80 (minor), 127.50, 128.09, 131.47. IR (neat): 3264, 3078, 2931, 2859, 1598, 1488, 1443, 1365, 1325, 1282, 1221, 1204, 1175, 1061, 1035, 1004, 995, 968, 944, 918, 900, 866, 842, 756, 691, 664, 591, 558, 545, 527, 509, 494, 459, 438, 420 cm^{-1}. HRMS (ESI) calcd. for $C_{14}H_{17}O$ [M+H]$^+$: 201.1274, found 201.1272.

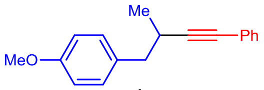

4v

1-Methoxy-4-(2-methyl-4-phenylbut-3-yn-1-yl)benzene (4v). 0.3 mmol scale, $NiCl_2·6H_2O$ (0.03 mmol, 7.1 mg), **L4** (0.03 mmol, 7.9 mg), anhydrous K_3PO_4 (0.39 mmol, 82.8 mg), 1-(1-(4-methoxyphenyl)propan-2-yl)-2,4,6-triphenylpyridin-1-ium tetrafluoroborate **1v** (0.3 mmol, 163.0 mg), phenylacetylene (0.45 mmol, 46.0 mg) and *N*, *N*-dimethylformamide (1.5 mL) were stirred at 80 °C for 24 h. Then the reaction mixture was extracted with ethyl acetate, washed with water and brine, and dried over anhydrous Na_2SO_4. Purification of the crude product by column chromatography on silica gel (eluent: petroleum ether: dichloromethane = 20:1) afforded the title product in 84% yield (62.8 mg) as a colorless oil. ^{1}H NMR (400 MHz, $CDCl_3$): δ 1.24 (d, *J* = 6.4 Hz, 2H), 2.69-2.75 (m, 1H), 2.80-2.88 (m, 3H), 3.77 (s, 3H), 6.82-6.86 (m, 2H), 7.16-7.21 (m, 2H), 7.23-7.28 (m, 3H), 7.34-7.37 (m, 2H). ^{13}C NMR (100 MHz, $CDCl_3$): δ 20.44, 28.75, 42.22, 55.16, 81.51,

94.18, 113.51, 123.91, 127.48, 128.12, 130.25, 131.47, 131.69, 158.11. IR (neat): 2968, 2931, 2834, 2230, 1611, 1584, 1511, 1489, 1455, 1442, 1373, 1336, 1300, 1244, 1177, 1113, 1070, 1034, 914, 835, 807, 754, 691, 621, 575, 537 cm^{-1}. HRMS (ESI) calcd. for $C_{18}H_{19}O$ $[M+H]^+$: 251.1430, found 251.1430.

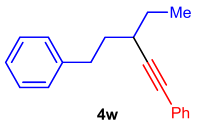

4w

(3-Ethylpent-1-yne-1,5-diyl)dibenzene (4w). 0.3 mmol scale, $NiCl_2 \cdot 6H_2O$ (0.03 mmol, 7.1 mg), **L4** (0.03 mmol, 7.9 mg), anhydrous K_3PO_4 (0.39 mmol, 82.8 mg), 2,4,6-triphenyl-1-(1-phenylpentan-3-yl)pyridin-1-ium tetrafluoroborate **1w** (0.3 mmol, 162.4 mg), phenylacetylene (0.45 mmol, 46.0 mg) and *N*, *N*-dimethylformamide (1.5 mL) were stirred at 80 °C for 24 h. Then the reaction mixture was extracted with ethyl acetate, washed with water and brine, and dried over anhydrous Na_2SO_4. Purification of the crude product by column chromatography on silica gel (eluent: petroleum ether: dichloromethane = 100:1) afforded the title product in 82% yield (61.2 mg) as a colorless oil. ^1H NMR (400 MHz, $CDCl_3$): δ 1.06 (t, J = 7.6 Hz, 3H), 1.53-1.62 (m, 2H), 1.79-1.86 (m, 2H), 2.45-2.52 (m, 2H), 2.73-2.81 (m, 1H), 2.87-2.94 (m, 1H), 7.16-7.19 (m, 1H), 7.22-7.30 (m, 7H), 7.41-7.44 (m, 2H). ^{13}C NMR (100 MHz, $CDCl_3$): δ 11.81, 28.15, 33.48, 33.77, 36.59, 82.46, 93.04, 124.09, 125.77, 127.48, 128.17, 128.33, 128.51, 131.59, 142.17. IR (neat): 3061, 3026, 2963, 2927, 2859, 1599, 1489, 1454, 1443, 1380, 1347, 1070, 1029, 913, 754, 691, 544, 516, 492, 403 cm^{-1}. HRMS (EI) calcd. for $C_{19}H_{20}$ $[M]^+$: 248.1560, found 248.1559.

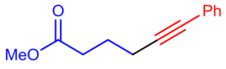

4x

Methyl 6-phenylhex-5-ynoate (4x). 0.3 mmol scale, $NiCl_2 \cdot 6H_2O$ (0.03 mmol, 7.1 mg), **L4** (0.03 mmol, 7.9 mg), anhydrous K_3PO_4 (0.39 mmol, 82.8 mg), 1-(4-methoxy-4-oxobutyl)-2,4,6-triphenylpyridin-1-ium tetrafluoroborate **1x** (0.3 mmol, 148.6 mg), phenylacetylene (0.45 mmol, 46.0 mg) and tetrahydrofuran (1.5 mL) were stirred at 80 °C

for 24 h. Then the reaction mixture was filtered through a short pad of silica gel and washed with ethyl acetate. Purification of the crude product by column chromatography on silica gel (eluent: petroleum ether: ethyl acetate = 300:1) afforded the title product in 81% yield (48.9 mg) as a colorless oil. ^{1}H NMR (400 MHz, $CDCl_3$): δ 1.90-1.97 (m, 2H), 2.47-2.53 (m, 4H), 3.68 (s, 3H), 7.26-7.30 (m, 3H), 7.38-7.40 (m, 2H). ^{13}C NMR (100 MHz, $CDCl_3$): δ 18.81, 23.85, 32.84, 51.53, 81.40, 88.77, 123.67, 127.63, 128.16, 131.51, 173.59. IR (neat): 2951, 1734, 1599, 1490, 1436, 1369, 1315, 1216, 1156, 1070, 1019, 916, 868, 756, 691, 550, 526 cm^{-1}. HRMS (ESI) calcd. for $C_{13}H_{15}O_2$ $[M+H]^+$: 203.1067, found 203.1064.

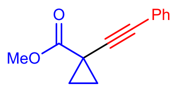

4y

Methyl 1-(phenylethynyl)cyclopropane-1-carboxylate (4y). 0.3 mmol scale, $NiCl_2·6H_2O$ (0.03 mmol, 7.1 mg), **L4** (0.03 mmol, 7.9 mg), anhydrous K_3PO_4 (0.39 mmol, 82.8 mg), 1-(1-(methoxycarbonyl)cyclopropyl)-2,4,6-triphenylpyridin-1-ium tetrafluoroborate **1y** (0.3 mmol, 148.0 mg), phenylacetylene (0.45 mmol, 46.0 mg) and *N*, *N*-dimethylformamide (1.5 mL) were stirred at 80 °C for 24 h. Then the reaction mixture was extracted with ethyl acetate, washed with water and brine, and dried over anhydrous Na_2SO_4. Purification of the crude product by column chromatography on silica gel (eluent: petroleum ether: ethyl acetate = 500:1) afforded the title product in 44% yield (26.4 mg) as a colorless oil. ^{1}H NMR (600 MHz, $CDCl_3$): δ 1.41-1.43 (m, 2H), 1.63-1.65 (m, 2H), 3.77 (s, 3H), 7.28-7.29 (m, 3H), 7.41-7.44 (m, 2H). ^{13}C NMR (151 MHz, $CDCl_3$): δ 16.08, 21.12, 52.88, 79.30, 88.41, 123.11, 128.00, 128.17, 131.79, 172.46. IR (neat): 2997, 2949, 2845, 1720, 1494, 1435, 1415, 1351, 1296, 1203, 1158, 1075, 1043, 1006, 959, 929, 885, 824, 763, 747, 696, 566, 544, 509, 467 cm^{-1}. HRMS (ESI) calcd. for $C_{13}H_{13}O_2$ $[M+H]^+$: 201.0910, found 201.0907.

Late-stage modification of natural products and medicinally relevant molecules

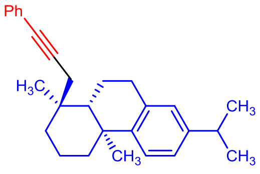

5

(1*S*,4a*S*,10a*S*)-7-Isopropyl-1,4a-dimethyl-1-(3-phenylprop-2-yn-1-yl)-1,2,3,4,4a,9,10,10a-octahydrophenanthrene (5). Following **General procedure C**, 0.3 mmol scale, $NiCl_2 \cdot 6H_2O$ (0.03 mmol, 7.1 mg), **L4** (0.03 mmol, 7.9 mg), anhydrous K_3PO_4 (0.39 mmol, 82.8 mg), 1-(((1*R*,4a*S*,10a*R*)-7-isopropyl-1,4a-dimethyl-1,2,3,4,4a,9,10,10a-octahydro-phenanthren-1-yl)methyl)-2,4,6-triphenylpyridin-1-ium tetrafluoroborate **S5** (0.3 mmol, 199.1 mg), phenylacetylene (0.45 mmol, 46.0 mg) and tetrahydrofuran (1.5 mL) were stirred at 80 °C for 24 h. Then the reaction mixture was filtered through a short pad of silica gel and washed with dichloromethane. Purification of the crude product by column chromatography on silica gel (eluent: petroleum ether: dichloromethane = 100:3) afforded the title product in 83% yield (92.4 mg) as a white solid. ^{1}H NMR (600 MHz, $CDCl_3$): δ 1.03 (s, 3H), 1.21-1.22 (m, 9H), 1.40-1.47 (m, 1H), 1.54-1.63 (m, 2H), 1.66-1.72 (m, 2H), 1.74-1.79 (m, 2H), 1.83-1.85 (m, 1H), 2.27 (d, *J* = 12.6 Hz, 1H), 2.36 (d, *J* = 16.8 Hz, 1H), 2.43 (d, *J* = 16.8 Hz, 1H), 2.79-2.83 (m, 1H), 2.87-2.90 (m, 2H), 6.88 (s, 1H), 6.98 (d, *J* = 7.8 Hz, 1H), 7.17 (d, *J* = 7.8 Hz, 1H), 7.22-7.23 (m, 3H), 7.35 (d, *J* = 6.0 Hz, 2H). ^{13}C NMR (151 MHz, $CDCl_3$): δ 19.08, 20.50, 23.96, 25.29, 30.63, 33.40, 34.91, 37.11, 37.70, 38.00, 38.48, 47.52, 82.91, 88.11, 123.84, 124.04, 124.43, 126.83, 127.43, 128.12, 131.57, 134.82, 145.40, 147.24 *(1 aliphatic carbon signal is not observed due to signal overlap)*. IR (neat): 2982, 2958, 2925, 1597, 1489, 1455, 1442, 1422, 1380, 1362, 1340, 1299, 1176, 1086, 1070, 1026, 985, 974, 914, 889, 822, 754, 691, 627, 526, 432, 416 cm^{-1}. HRMS (EI) calcd. for $C_{28}H_{34}$ $[M]^+$: 370.2655, found 370.2654.

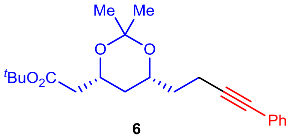

6

***tert*-Butyl 2-((4*R*,6*R*)-2,2-dimethyl-6-(4-phenylbut-3-yn-1-yl)-1,3-dioxan-4-yl)acetate**

(6). Following **General procedure C**, 0.3 mmol scale, $NiCl_2·6H_2O$ (0.03 mmol, 7.1 mg), **L4** (0.03 mmol, 7.9 mg), anhydrous K_3PO_4 (0.39 mmol, 82.8 mg), 1-(2-((4*R*,6*R*)-6-(2-(*tert*-butoxy)-2-oxoethyl)-2,2-dimethyl-1,3-dioxan-4-yl)ethyl)-2,4,6-triphenylpyridin-1-ium tetrafluoroborate **S6** (0.3 mmol, 195.5 mg), phenylacetylene (0.45 mmol, 46.0 mg) and tetrahydrofuran (1.5 mL) were stirred at 80 °C for 24 h. Then the reaction mixture was filtered through a short pad of silica gel and washed with ethyl acetate. Purification of the crude product by column chromatography on silica gel (eluent: petroleum ether: ethyl acetate = 25:1) afforded the title product in 94% yield (100.8 mg) as a colorless oil. ^1H NMR (600 MHz, $CDCl_3$): δ 1.19-1.26 (m, 1H), 1.38 (s, 3H), 1.44 (s, 9H), 1.47 (s, 3H), 1.60 (d, *J* = 12.6 Hz, 1H), 1.69-1.77 (m, 2H), 2.31 (dd, *J* = 5.4, 15.0 Hz, 1H), 2.44 (dd, *J* = 6.6, 15.0 Hz, 1H), 2.47-2.54 (m, 2H), 4.07 (br, 1H), 4.25-4.29 (m, 1H), 7.26-7.27 (m, 3H), 7.38 (d, *J* = 6.0 Hz, 2H). ^{13}C NMR (151 MHz, $CDCl_3$): δ 15.06, 19.62, 28.01, 30.01, 34.96, 36.25, 42.63, 66.19, 67.29, 80.48, 80.73, 89.47, 98.72, 123.86, 127.50, 128.14, 131.44, 170.21. IR (neat): 2993, 2977, 2950, 2912, 1717, 1490, 1459, 1442, 1375, 1342, 1317, 1295, 1284, 1262, 1203, 1191, 1167, 1144, 1121, 1087, 1069, 1040, 1030, 988, 968, 953, 927, 912, 869, 838, 777, 752, 690, 655, 525, 502, 479, 422 cm^{-1}. HRMS (ESI) calcd. for $C_{22}H_{30}NaO_4$ $[M+Na]^+$: 381.2036, found 381.2034.

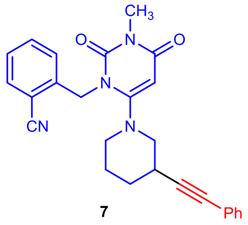

7

2-((3-Methyl-2,4-dioxo-6-(3-(phenylethynyl)piperidin-1-yl)-3,4-dihydropyrimidin-1(2*H*)-yl)methyl)benzonitrile (7). Following **General procedure D**, 0.3 mmol scale, $NiCl_2·6H_2O$ (0.03 mmol, 7.1 mg), **L4** (0.03 mmol, 7.9 mg), anhydrous K_3PO_4 (0.39 mmol, 82.8 mg), 1-(1-(3-(2-cyanobenzyl)-1-methyl-2,6-dioxo-1,2,3,6-tetrahydropyrimidin-4-yl)-piperidin-3-yl)-2,4,6-triphenylpyridin-1-ium tetrafluoroborate **S7** (0.3 mmol, 215.3 mg), phenylacetylene (0.45 mmol, 46.0 mg) and *N*, *N*-dimethylformamide (1.5 mL) were stirred at 80 °C for 24 h. Then the reaction mixture was extracted with ethyl acetate, washed with water and brine, and dried over anhydrous Na_2SO_4. Purification of the crude product by

column chromatography on silica gel (eluent: petroleum ether: ethyl acetate: dichloromethane = 3:1:3) afforded the title product in 94% yield (120.1 mg) as a light-yellow solid. ^{1}H NMR (400 MHz, $CDCl_3$): δ 1.65-1.68 (m, 2H), 1.96-2.03 (m, 2H), 2.87-2.94 (m, 4H), 3.10-3.13 (m, 1H), 3.28 (s, 3H), 5.28 (d, J = 16.0 Hz, 1H), 5.41-5.44 (m, 2H), 7.15 (d, J = 8.0 Hz, 1H), 7.18-7.26 (m, 5H), 7.29-7.32 (m, 1H), 7.50 (t, J = 7.6 Hz, 1H), 7.58 (d, J = 7.6 Hz, 1H). ^{13}C NMR (100 MHz, $CDCl_3$): δ 23.10, 27.71, 28.48, 29.80, 46.06, 51.76, 55.80, 82.48, 89.24, 90.20, 110.84, 116.94, 122.65, 126.61, 127.65, 127.81, 128.00, 131.32, 132.89, 132.93, 140.52, 152.31, 159.52, 162.92. IR (neat): 2943, 2855, 2224, 1702, 1651, 1608, 1489, 1434, 1376, 1312, 1284, 1223, 1164, 1104, 1028, 1008, 947, 918, 862, 806, 756, 692, 659, 625, 607, 587, 559, 536, 519, 432 cm^{-1}. HRMS (ESI) calcd. for $C_{26}H_{25}N_4O_2$ $[M+H]^+$: 425.1972, found 425.1972.

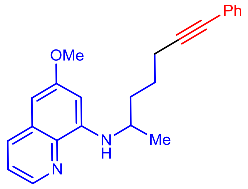

8

6-Methoxy-*N*-(7-phenylhept-6-yn-2-yl)quinolin-8-amine (8). Following **General procedure C**, 0.3 mmol scale, $NiCl_2{\cdot}6H_2O$ (0.03 mmol, 7.1 mg), **L4** (0.03 mmol, 7.9 mg), anhydrous K_3PO_4 (0.39 mmol, 82.8 mg), 1-(4-((6-methoxyquinolin-8-yl)amino)pentyl)-2,4,6-triphenylpyridin-1-ium tetrafluoroborate **S8** (0.3 mmol, 191.3 mg), phenylacetylene (0.45 mmol, 46.0 mg) and tetrahydrofuran (1.5 mL) were stirred at 80 °C for 24 h. Then the reaction mixture was filtered through a short pad of silica gel and washed with ethyl acetate. Purification of the crude product by column chromatography on silica gel (eluent: petroleum ether: ethyl acetate = 25:1) afforded the title product in 75% yield (77.7 mg) as a yellow oil. ^{1}H NMR (400 MHz, $CDCl_3$): δ 1.32 (d, J = 6.4 Hz, 3H), 1.70-1.82 (m, 3H), 1.83-1.90 (m, 1H), 2.43 (t, J = 6.4 Hz, 2H), 3.66 (br, 1H), 3.84 (s, 3H), 6.06 (br, 1H), 6.30-6.32 (m, 2H), 7.21-7.28 (m, 4H), 7.36-7.39 (m, 2H), 7.88 (dd, J = 1.6, 8.2 Hz, 1H), 8.51 (dd, J = 1.6, 4.0 Hz, 1H). ^{13}C NMR (100 MHz, $CDCl_3$): δ 19.36, 20.46, 25.35, 35.85, 47.65, 55.07, 80.92, 89.85, 91.50, 96.63, 121.74, 123.88, 127.43, 128.08, 129.84, 131.50, 134.70, 135.30, 144.19, 144.95, 159.40. IR (neat): 3385, 2935, 2862, 1614, 1595, 1576,

1517, 1489, 1455, 1422, 1386, 1335, 1261, 1217, 1196, 1160, 1070, 1050, 1030, 968, 900, 819, 790, 755, 691, 624, 525, 463 cm^{-1}. HRMS (ESI) calcd. for $C_{23}H_{25}N_2O$ $[M+H]^+$: 345.1961, found 345.1961.

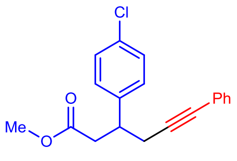

9

Methyl 3-(4-chlorophenyl)-6-phenylhex-5-ynoate (9). Following **General procedure D**, 0.3 mmol scale, $NiCl_2{\cdot}6H_2O$ (0.03 mmol, 7.1 mg), **L4** (0.03 mmol, 7.9 mg), anhydrous K_3PO_4 (0.39 mmol, 82.8 mg), 1-(2-(4-chlorophenyl)-4-methoxy-4-oxobutyl)-2,4,6-triphenylpyridin-1-ium tetrafluoro- borate **S9** (0.3 mmol, 181.8 mg), phenylacetylene (0.45 mmol, 46.0 mg) and *N*, *N*-dimethylformamide (1.5 mL) were stirred at 80 °C for 24 h. Then the reaction mixture was extracted with ethyl acetate, washed with water and brine, and dried over anhydrous Na_2SO_4. Purification of the crude product by column chromatography on silica gel (eluent: petroleum ether: ethyl acetate = 100:1) afforded the title product in 70% yield (65.9 mg) as a light-yellow oil. ^{1}H NMR (600 MHz, $CDCl_3$): δ 2.67-2.76 (m, 3H), 2.96 (dd, J = 6.6, 15.6 Hz, 1H), 3.42-3.46 (m, 1H), 3.59 (s, 3H), 7.22 (d, J = 8.4 Hz, 2H), 7.26-7.29 (m, 5H), 7.33-7.34 (m, 2H). ^{13}C NMR (151 MHz, $CDCl_3$): δ 26.67, 39.03, 40.21, 51.61, 82.97, 86.88, 123.33, 127.83, 128.19, 128.57, 128.67, 131.46, 132.62, 141.18, 172.17. IR (neat): 2951, 2913, 1734, 1596, 1489, 1442, 1429, 1367, 1344, 1308, 1287, 1253, 1217, 1200, 1190, 1179, 1157, 1106, 1092, 1070, 1026, 1013, 1000, 974, 956, 919, 890, 827, 792, 756, 731, 692, 667, 544, 527, 489, 441 cm^{-1}. HRMS (ESI) calcd. for $C_{19}H_{17}ClNaO_2$ $[M+Na]^+$: 335.0809, found 335.0803.

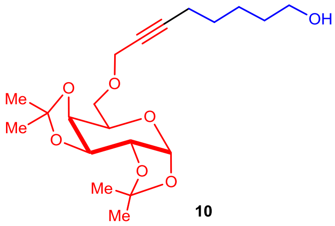

10

8-(((3a*R*,5*R*,5a*S*,8a*S*,8b*R*)-2,2,7,7-Tetramethyltetrahydro-5*H*-bis([1,3]dioxolo)[4,5-*b*:4'

,5'-*d*]pyran-5-yl)methoxy)oct-6-yn-1-ol (10). Following **General procedure C**, 0.3 mmol scale, $NiCl_2·6H_2O$ (0.03 mmol, 7.1 mg), **L4** (0.03 mmol, 7.9 mg), anhydrous K_3PO_4 (0.39 mmol, 82.8 mg), 1-(5-hydroxypentyl)-2,4,6-triphenylpyridin-1-ium tetrafluoroborate **1l** (0.3 mmol, 144.4 mg), (3a*R*,5*R*,5a*S*,8a*S*,8b*R*)-2,2,7,7-tetramethyl-5-((prop-2-yn-1-yloxy)methyl)tetrahydro-5*H*-bis([1,3]dioxolo)[4,5-*b*:4',5'-*d*]pyran **S10** (0.45 mmol, 134.3 mg) and tetrahydrofuran (1.5 mL) were stirred at 80 °C for 24 h. Then the reaction mixture was filtered through a short pad of silica gel and washed with ethyl acetate. Purification of the crude product by column chromatography on silica gel (eluent: petroleum ether: ethyl acetate: dichloromethane = 3:1:1) afforded the title product in 89% yield (102.7 mg) as a light-yellow oil. ^{1}H NMR (600 MHz, $CDCl_3$): δ 1.33 (s, 3H), 1.35 (s, 3H), 1.44-1.49 (m, 5H), 1.52-1.60 (m, 7H), 2.11 (br, 1H), 2.22-2.25 (m, 2H), 3.62-3.66 (m, 3H), 3.73 (dd, *J* = 5.4, 9.9 Hz, 1H), 3.98-4.00 (m, 1H), 4.15-4.23 (m, 2H), 4.27 (dd, *J* = 1.2, 7.8 Hz, 1H), 4.32 (dd, *J* = 2.4, 5.4 Hz, 1H), 4.60 (dd, *J* = 2.4, 7.8 Hz, 1H), 5.54 (d, *J* = 5.4 Hz, 1H). ^{13}C NMR (151 MHz, $CDCl_3$): δ 18.59, 24.35, 24.78, 24.86, 25.83, 25.89, 28.14, 32.05, 58.89, 62.42, 66.57, 68.16, 70.35, 70.52, 71.06, 75.86, 86.89, 96.21, 108.48, 109.18. IR (neat): 3461, 2987, 2935, 1457, 1372, 1308, 1255, 1210, 1168, 1097, 1066, 1000, 917, 889, 864, 824, 804, 771, 693, 651, 512, 422 cm^{-1}. HRMS (ESI) calcd. for $C_{20}H_{32}NaO_7$ $[M+Na]^+$: 407.2040, found 407.2042.

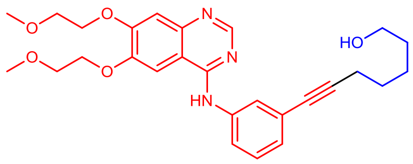

11

7-(3-((6,7-Bis(2-methoxyethoxy)quinazolin-4-yl)amino)phenyl)hept-6-yn-1-ol (11). Following **General procedure C**, 0.3 mmol scale, $NiCl_2·6H_2O$ (0.03 mmol, 7.1 mg), **L4** (0.03 mmol, 7.9 mg), anhydrous K_3PO_4 (0.39 mmol, 82.8 mg), 1-(5-hydroxypentyl)-2,4,6-triphenylpyridin-1-ium tetrafluoroborate **1l** (0.3 mmol, 144.4 mg), *N*-(3-ethynylphenyl)-6,7-bis(2-methoxyethoxy)quinazolin-4-amine **S11** (0.45 mmol, 177.0 mg) and tetrahydrofuran (1.5 mL) were stirred at 80 °C for 24 h. Then the reaction mixture was filtered through a short pad of silica gel and washed with acetone. Purification of the

crude product by column chromatography on silica gel (eluent: dichloromethane: acetone = 3:1) afforded the title product in 87% yield (124.7 mg) as a light-yellow solid. ^{1}H NMR (400 MHz, $CDCl_3$): δ 1.38-1.48 (m, 6H), 2.26 (t, *J* = 6.4 Hz, 2H), 3.25 (s, 6H), 3.54 (t, *J* = 6.4 Hz, 3H), 3.60 (br, 4H), 3.95 (br, 2H), 4.09 (br, 2H), 7.00 (d, *J* = 8.0 Hz, 1H), 7.07-7.14 (m, 2H), 7.33 (s, 1H), 7.57 (d, *J* = 8.0 Hz, 1H), 7.64 (s, 1H), 8.44 (s, 1H), 8.53 (br, 1H). ^{13}C NMR (100 MHz, $CDCl_3$): δ 19.18, 25.02, 28.25, 32.10, 58.98, 59.01, 62.30, 68.09, 68.63, 70.20, 70.63, 80.42, 90.23, 102.77, 107.76, 109.15, 121.50, 124.41, 124.95, 127.10, 128.56, 138.65, 146.61, 148.49, 153.13, 154.19, 156.60. IR (neat): 3311, 2928, 2858, 1621, 1578, 1529, 1509, 1480, 1430, 1391, 1354, 1286, 1251, 1213, 1124, 1095, 1070, 1030, 931, 897, 859, 785, 686, 658, 588, 546, 513, 501, 482, 462, 450, 430, 421, 408 cm^{-1}. HRMS (ESI) calcd. for $C_{27}H_{34}N_3O_5$ $[M+H]^+$: 480.2493, found 480.2493.

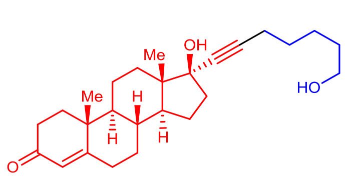

12

(8*R*,9*S*,10*R*,13*S*,14*S*,17*S*)-17-Hydroxy-17-(7-hydroxyhept-1-yn-1-yl)-10,13-dimethyl-1,2,6,7,8,9,10,11,12,13,14,15,16,17-tetradecahydro-3*H*-cyclopenta[*a*]phenanthren-3-one (12). Following **General procedure C**, 0.3 mmol scale, $NiCl_2 \cdot 6H_2O$ (0.03 mmol, 7.1 mg), **L4** (0.03 mmol, 7.9 mg), anhydrous K_3PO_4 (0.39 mmol, 82.8 mg), 1-(5-hydroxypentyl)-2,4,6-triphenylpyridin-1-ium tetrafluoroborate **1l** (0.3 mmol, 144.4 mg), (8*R*,9*S*,10*R*,13*S*,14*S*,17*R*)-17-ethynyl-17-hydroxy-10,13-dimethyl-1,2,6,7,8,9,10,11,12,13,14,15,16,17-tetradecahydro-3*H*-cyclopenta[*a*]phenanthren-3-one **S12** (0.45 mmol, 140.6 mg) and tetrahydrofuran (1.5 mL) were stirred at 80 °C for 24 h. Then the reaction mixture was filtered through a short pad of silica gel and washed with ethyl acetate. Purification of the crude product by column chromatography on silica gel (eluent: petroleum ether: ethyl acetate: dichloromethane = 1:1:1) afforded the title product in 89% yield (106.6 mg) as a light-yellow solid. ^{1}H NMR (400 MHz, $CDCl_3$): δ 0.80 (s, 3H), 0.85-0.92 (m, 1H), 0.96-1.03 (m, 1H), 1.13 (s, 3H), 1.22-1.29 (m, 1H), 1.32-1.70 (m, 14H), 1.78 (d, *J* = 12.0 Hz, 1H), 1.87-1.99 (m, 2H), 2.10-2.40 (m, 7H), 2.61 (s, 2H), 3.54 (t, *J* = 6.0 Hz, 2H), 5.67

(s, 1H). ^{13}C NMR (100 MHz, $CDCl_3$): δ 12.65, 17.22, 18.50, 20.56, 22.84, 24.78, 28.21, 31.36, 31.81, 32.36, 32.63, 33.69, 35.43, 36.01, 38.46, 38.78, 46.48, 49.63, 53.34, 62.21, 79.36, 83.65, 85.76, 123.52, 171.66, 199.74. IR (neat): 3386, 2934, 2858, 1658, 1614, 1449, 1434, 1379, 1357, 1330, 1273, 1232, 1188, 1128, 1067, 1052, 1023, 954, 867, 698, 676, 571, 514, 462, 437, 413 cm^{-1}. HRMS (ESI) calcd. for $C_{26}H_{39}O_3$ $[M+H]^+$: 399.2894, found 399.2894.

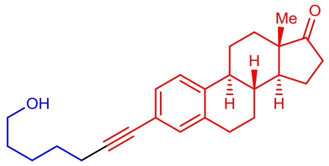

13

(8*R*,9*S*,13*S*,14*S*)-3-(7-Hydroxyhept-1-yn-1-yl)-13-methyl-6,7,8,9,11,12,13,14,15,16-decahydro-17*H*-cyclopenta[*a*]phenanthren-17-one (13). Following **General procedure C**, 0.3 mmol scale, $NiCl_2 \cdot 6H_2O$ (0.03 mmol, 7.1 mg), **L4** (0.03 mmol, 7.9 mg), anhydrous K_3PO_4 (0.39 mmol, 82.8 mg), 1-(5-hydroxypentyl)-2,4,6-triphenylpyridin-1-ium tetrafluoroborate **1l** (0.3 mmol, 144.4 mg), (8*R*,9*S*,13*S*,14*S*)-3-ethynyl-13-methyl-6,7,8,9,11,12,13,14,15,16-decahydro-17*H*-cyclopenta[*a*]phenanthren-17-one **S13** (0.45 mmol, 125.3 mg) and tetrahydrofuran (1.5 mL) were stirred at 80 °C for 24 h. Then the reaction mixture was filtered through a short pad of silica gel and washed with ethyl acetate. Purification of the crude product by column chromatography on silica gel (eluent: petroleum ether: ethyl acetate: dichloromethane = 5:1:3) afforded the title product in 91% yield (99.5 mg) as a white solid. ^{1}H NMR (400 MHz, $CDCl_3$): δ 0.89 (s, 3H), 1.34-1.66 (m, 12H), 1.91-2.17 (m, 5H), 2.21-2.26 (m, 1H), 2.36-2.41 (m, 3H), 2.49 (dd, *J* = 8.8, 18.8 Hz, 1H), 2.85 (dd, *J* = 4.0, 8.8 Hz, 2H), 3.65 (t, *J* = 6.4 Hz, 2H), 7.12-7.19 (m, 3H). ^{13}C NMR (100 MHz, $CDCl_3$): δ 13.68, 19.25, 21.42, 24.95, 25.43, 26.21, 28.45, 28.94, 31.38, 32.14, 35.71, 37.83, 44.19, 47.81, 50.31, 62.58, 80.56, 89.23, 121.16, 125.10, 128.72, 131.84, 136.26, 139.26, 220.86. IR (neat): 3482, 2933, 2858, 1721, 1495, 1466, 1452, 1402, 1261, 1085, 1069, 1045, 1009, 963, 896, 823, 781, 578, 519, 498, 471, 441 cm^{-1}. HRMS (ESI) calcd. for $C_{25}H_{32}NaO_2$ $[M+Na]^+$: 387.2295, found 387.2294.

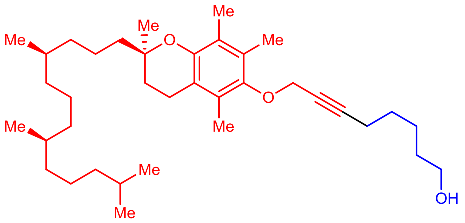

14

8-(((*R*)-2,5,7,8-Tetramethyl-2-((4*R*,8*R*)-4,8,12-trimethyltridecyl)chroman-6-yl)oxy)oct-6-yn-1-ol (14). Following **General procedure C**, 0.3 mmol scale, $NiCl_2 \cdot 6H_2O$ (0.03 mmol, 7.1 mg), **L4** (0.03 mmol, 7.9 mg), anhydrous K_3PO_4 (0.39 mmol, 82.8 mg), 1-(5-hydroxypentyl)-2,4,6-triphenylpyridin-1-ium tetrafluoroborate **1l** (0.3 mmol, 144.4 mg), (*R*)-2,5,7,8-tetramethyl-6-(prop-2-yn-1-yloxy)-2-((4*R*,8*R*)-4,8,12-trimethyltridecyl)-chromane **S14** (0.45 mmol, 211.0 mg) and tetrahydrofuran (1.5 mL) were stirred at 80 °C for 24 h. Then the reaction mixture was filtered through a short pad of silica gel and washed with ethyl acetate. Purification of the crude product by column chromatography on silica gel (eluent: petroleum ether: acetone = 5:1) afforded the title product in 77% yield (128.8 mg) as a colorless oil. ^1H NMR (600 MHz, $CDCl_3$): δ 0.84-0.87 (m, 12H), 1.02-1.09 (m, 3H), 1.10-1.17 (m, 3H), 1.20-1.33 (m, 11H), 1.34-1.40 (m, 3H), 1.43-1.48 (m, 3H), 1.51-1.59 (m, 7H), 1.72-1.76 (m, 1H), 1.78-1.83 (m, 2H), 2.07 (s, 3H), 2.15 (s, 3H), 2.20 (s, 3H), 2.24-2.27 (m, 2H), 2.56 (t, *J* = 6.6 Hz, 2H), 3.62 (t, *J* = 6.6 Hz, 2H), 4.32 (t, *J* = 1.8 Hz, 2H). ^{13}C NMR (151 MHz, $CDCl_3$): δ 11.72, 12.13, 12.99, 18.76, 19.59, 19.68, 20.58, 20.95, 22.56, 22.65, 23.78, 24.37, 24.73, 24.93, 27.90, 28.18, 31.19, 32.16, 32.62, 32.72, 37.22, 37.35, 37.38, 37.40, 39.30, 40.01, 61.08, 62.62, 74.77, 76.14, 86.79, 117.40, 122.76, 126.03, 127.98, 147.85, 147.99. IR (neat): 3364, 2926, 2863, 1458, 1412, 1376, 1365, 1332, 1251, 1157, 1085, 983, 925, 864, 736, 669, 611, 476, 431, 419, 406 cm^{-1}. HRMS (ESI) calcd. for $C_{37}H_{63}O_3$ $[M+H]^+$: 555.4772, found 555.4768.

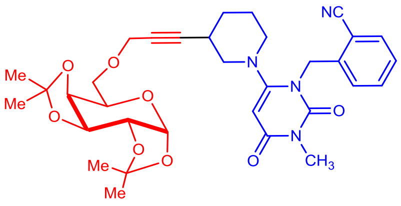

15

2-((3-Methyl-2,4-dioxo-6-(3-(3-(((3a*R*,5*R*,5a*S*,8a*S*,8b*R*)-2,2,7,7-tetramethyltetrahydro-

5*H*-bis([1,3]dioxolo)[4,5-*b*:4',5'-*d*]pyran-5-yl)methoxy)prop-1-yn-1-yl)piperidin-1-yl)-3,4-dihydropyrimidin-1(2*H*)-yl)methyl)benzonitrile (15). Following **General procedure D**, 0.3 mmol scale, $NiCl_2·6H_2O$ (0.03 mmol, 7.1 mg), **L4** (0.03 mmol, 7.9 mg), anhydrous K_3PO_4 (0.39 mmol, 82.8 mg), 1-(1-(3-(2-cyanobenzyl)-1-methyl-2,6-dioxo-1,2,3,6-tetrahydropyrimidin-4-yl)piperidin-3-yl)-2,4,6-triphenylpyridin-1-ium tetrafluoro-borate **S7** (0.3 mmol, 215.3 mg), (3a*R*,5*R*,5a*S*,8a*S*,8b*R*)-2,2,7,7-tetramethyl-5-((prop-2-yn-1-yloxy)methyl)tetrahydro-5*H*-bis([1,3]dioxolo)[4,5-*b*:4',5'-*d*]pyran **S10** (0.45 mmol, 134.3 mg) and *N*, *N*-dimethylformamide (1.5 mL) were stirred at 80 °C for 24 h. Then the reaction mixture was extracted with ethyl acetate, washed with water and brine, and dried over anhydrous Na_2SO_4. Purification of the crude product by column chromatography on silica gel (eluent: petroleum ether: ethyl acetate: dichloromethane = 1:1:1) afforded the title product in 92% yield (171.5 mg) as a white solid. ^1H NMR (400 MHz, $CDCl_3$): δ 1.25 (s, 3H), 1.27 (s, 3H), 1.37 (s, 3H), 1.46 (s, 3H), 1.52 (br, 2H), 1.76 (br, 1H), 1.85 (br, 1H), 2.59 (br, 1H), 2.69 (br, 2H), 2.84 (br, 1H), 2.96 (d, *J* = 10.4 Hz, 1H), 3.21 (s, 3H), 3.45-3.50 (m, 1H), 3.52-3.59 (m, 1H), 3.87 (t, *J* = 5.6 Hz, 1H), 3.96-4.05 (m, 2H), 4.16 (d, *J* = 7.6 Hz, 1H), 4.24 (dd, *J* = 2.4, 4.8 Hz, 1H), 4.52 (dd, *J* = 2.0, 7.6 Hz, 1H), 5.19-5.23 (m, 2H), 5.30 (s, 1H), 5.45 (d, *J* = 4.4 Hz, 1H), 7.09 (d, *J* = 7.6 Hz, 1H), 7.32 (t, *J* = 7.6 Hz, 1H), 7.50 (d, *J* = 7.6 Hz, 1H), 7.61 (d, *J* = 7.6 Hz, 1H). ^{13}C NMR (100 MHz, $CDCl_3$): δ 23.13, 24.25, 24.66, 25.75, 25.82, 27.63, 27.92, 29.68, 46.04, 51.68, 55.51, 58.47, 66.40, 68.21, 70.19, 70.37, 70.88, 77.87, 86.20, 90.22, 96.05, 108.28, 108.98, 110.64, 116.84, 126.47, 127.69, 132.88, 132.98, 140.45, 152.30, 159.37, 162.75. IR (neat): 2934, 2224, 1703, 1653, 1610, 1436, 1376, 1310, 1255, 1210, 1167, 1099, 1067, 1002, 955, 917, 889, 863, 806, 765, 730, 694, 650, 630, 607, 588, 559, 543, 514, 464, 430, 420 cm^{-1}. HRMS (ESI) calcd. for $C_{33}H_{41}N_4O_8$ $[M+H]^+$: 621.2919, found 621.2913.

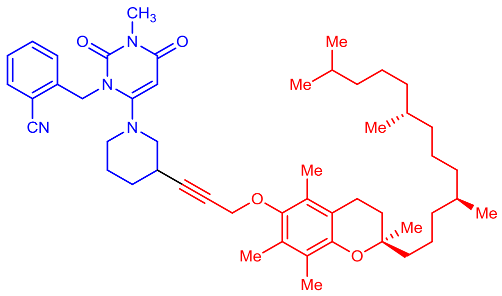

16

2-((3-Methyl-2,4-dioxo-6-(3-(3-(((*R*)-2,5,7,8-tetramethyl-2-((4*R*,8*R*)-4,8,12-trimethyltridecyl)chroman-6-yl)oxy)prop-1-yn-1-yl)piperidin-1-yl)-3,4-dihydropyrimidin-1(2*H*)-yl)methyl)benzonitrile (16). Following **General procedure D**, 0.3 mmol scale, $NiCl_2·6H_2O$ (0.03 mmol, 7.1 mg), **L4** (0.03 mmol, 7.9 mg), anhydrous K_3PO_4 (0.39 mmol, 82.8 mg), 1-(1-(3-(2-cyanobenzyl)-1-methyl-2,6-dioxo-1,2,3,6-tetrahydropyrimidin-4-yl)-piperidin-3-yl)-2,4,6-triphenylpyridin-1-ium tetrafluoroborate **S7** (0.3 mmol, 215.3 mg), (*R*)-2,5,7,8-tetramethyl-6-(prop-2-yn-1-yloxy)-2-((4*R*,8*R*)-4,8,12-trimethyltridecyl)chromane **S14** (0.45 mmol, 211.0 mg) and *N*, *N*-dimethylformamide (1.5 mL) were stirred at 80 °C for 24 h. Then the reaction mixture was extracted with ethyl acetate, washed with water and brine, and dried over anhydrous Na_2SO_4. Purification of the crude product by column chromatography on silica gel (eluent: petroleum ether: ethyl acetate: dichloromethane = 3:1:1) afforded the title product in 90% yield (212.8 mg) as a white solid. ^1H NMR (400 MHz, $CDCl_3$): δ 0.84-0.87 (m, 12H), 1.03-1.46 (m, 21H), 1.47-1.60 (m, 5H), 1.72-1.84 (m, 3H), 1.90-1.93 (m, 1H), 2.06 (s, 3H), 2.09 (s, 3H), 2.13 (s, 3H), 2.55 (t, *J* = 6.4 Hz, 2H), 2.69-2.90 (m, 4H), 3.02-3.05 (m, 1H), 3.29 (s, 3H), 4.24 (s, 2H), 5.25-5.38 (m, 3H), 7.16 (d, *J* = 7.6 Hz, 1H), 7.33 (t, *J* = 7.6 Hz, 1H), 7.52 (t, *J* = 7.6 Hz, 1H), 7.62 (d, *J* = 7.6 Hz, 1H). ^{13}C NMR (100 MHz, $CDCl_3$): δ 11.61, 12.04, 12.89, 19.49, 19.58, 20.45, 20.81, 22.45, 22.55, 23.10, 23.63, 24.22, 24.58, 27.68, 27.76, 28.03, 29.64, 31.06, 32.47, 32.57, 37.07, 37.20, 37.23, 37.26, 39.16, 39.86, 46.12, 51.71, 55.57, 60.53, 74.63, 78.19, 86.16, 90.29, 110.78, 116.91, 117.25, 122.61, 125.78, 126.65, 127.65, 127.67, 132.88, 132.93, 140.55, 147.61, 147.89, 152.34, 159.46, 162.80. IR (neat): 2925, 2862, 2226, 1705, 1657, 1612, 1436, 1376, 1364, 1327, 1250, 1228, 1164, 1085, 997, 925, 862, 807, 764, 697, 657, 608, 588, 558, 522, 417 cm^{-1}. HRMS (ESI) calcd. for $C_{50}H_{71}N_4O_4$ $[M+H]^+$: 791.5470, found 791.5468.

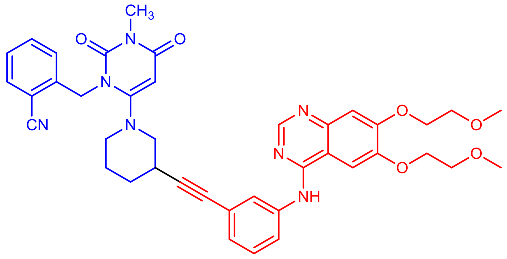

17

2-((6-(3-((3-((6,7-Bis(2-methoxyethoxy)quinazolin-4-yl)amino)phenyl)ethynyl)piperidin-1-yl)-3-methyl-2,4-dioxo-3,4-dihydropyrimidin-1(2*H*)-yl)methyl)benzonitrile (17). Following **General procedure D**, 0.3 mmol scale, $NiCl_2·6H_2O$ (0.03 mmol, 7.1 mg), **L4** (0.03 mmol, 7.9 mg), anhydrous K_3PO_4 (0.39 mmol, 82.8 mg), 1-(1-(3-(2-cyanobenzyl)-1-methyl-2,6-dioxo-1,2,3,6-tetrahydropyrimidin-4-yl)piperidin-3-yl)-2,4,6-triphenylpyridin-1-ium tetrafluoroborate **S7** (0.3 mmol, 215.3 mg), *N*-(3-ethynylphenyl)-6,7-bis(2-methoxyethoxy)quinazolin-4-amine **S11** (0.45 mmol, 177.0 mg) and *N*, *N*-dimethylformamide (1.5 mL) were stirred at 80 °C for 24 h. Then the reaction mixture was extracted with ethyl acetate, washed with water and brine, and dried over anhydrous Na_2SO_4. Purification of the crude product by column chromatography on silica gel (eluent: dichloromethane: ethyl acetate: acetone = 1:2:1) afforded the title product in 85% yield (183.3 mg) as a yellow solid. ^1H NMR (400 MHz, $CDCl_3$): δ 1.60 (br, 1H), 1.70-1.95 (m, 3H), 2.89 (br, 3H), 3.02 (br, 2H), 3.27 (s, 3H), 3.39 (s, 3H), 3.42 (s, 3H), 3.79 (dt, *J* = 4.4, 16.0 Hz, 4H), 4.21-4.24 (m, 4H), 5.27 (d, *J* = 16.0 Hz, 1H), 5.37-5.46 (m, 2H), 6.98 (d, *J* = 7.6 Hz, 1H), 7.14 (d, *J* = 8.0 Hz, 1H), 7.20-7.24 (m, 2H), 7.29 (t, *J* = 7.6 Hz, 1H), 7.52 (t, *J* = 7.6 Hz, 1H), 7.57-7.61 (m, 3H), 7.94 (d, *J* = 7.2 Hz, 1H), 8.46 (br, 1H), 8.63 (s, 1H). ^{13}C NMR (100 MHz, $CDCl_3$): δ 22.26, 27.65, 28.23, 29.29, 46.38, 51.56, 55.36, 58.95, 68.04, 68.48, 70.15, 70.43, 82.48, 89.33, 89.70, 102.28, 108.12, 109.26, 110.16, 117.18, 121.52, 123.05, 124.30, 126.25, 126.34, 127.61, 128.54, 132.91, 133.14, 138.95, 140.47, 146.95, 148.56, 152.20, 153.18, 154.00, 156.28, 159.62, 163.08 *(1 aliphatic carbon signal is not observed due to signal overlap)*. IR (neat): 2927, 2225, 1702, 1650, 1621, 1574, 1531, 1503, 1485, 1427, 1330, 1285, 1210, 1123, 1070, 1029, 926, 859, 786, 765, 687, 658, 587, 557, 520, 415 cm^{-1}. HRMS (ESI) calcd. for $C_{40}H_{42}N_7O_6$ $[M+H]^+$: 716.3191, found 716.3196.

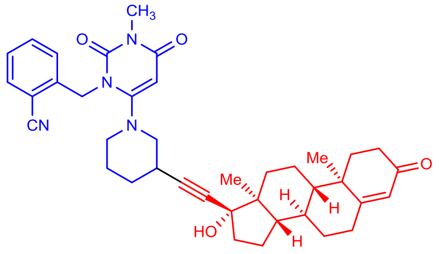

18

2-((6-(3-(((8*R*,9*S*,10*R*,13*S*,14*S*,17*S*)-17-Hydroxy-10,13-dimethyl-3-oxo-2,3,6,7,8,9,10,11,12,13,14,15,16,17-tetradecahydro-1*H*-cyclopenta[*a*]phenanthren-17-yl)ethynyl)piperidin-1-yl)-3-methyl-2,4-dioxo-3,4-dihydropyrimidin-1(2*H*)-yl)methyl)benzonitrile (18). Following **General procedure D**, 0.3 mmol scale, $NiCl_2·6H_2O$ (0.03 mmol, 7.1 mg), **L4** (0.03 mmol, 7.9 mg), anhydrous K_3PO_4 (0.39 mmol, 82.8 mg), 1-(1-(3-(2-cyanobenzyl)-1-methyl-2,6-dioxo-1,2,3,6-tetrahydropyrimidin-4-yl)piperidin-3-yl)-2,4,6-triphenylpyridin-1-ium tetrafluoroborate **S7** (0.3 mmol, 215.3 mg), (8*R*,9*S*,10*R*,13*S*,14*S*,17*R*)-17-ethynyl-17-hydroxy-10,13-dimethyl-1,2,6,7,8,9,10,11,12,13,14,15,16,17-tetradecahydro-3*H*-cyclopenta[*a*]phenanthren-3-one **S12** (0.45 mmol, 140.6 mg) and *N*, *N*-dimethylformamide (1.5 mL) were stirred at 80 °C for 24 h. Then the reaction mixture was extracted with ethyl acetate, washed with water and brine, and dried over anhydrous Na_2SO_4. Purification of the crude product by column chromatography on silica gel (eluent: petroleum ether: ethyl acetate: dichloromethane = 1:2:1) afforded a mixture of diastereoisomers (d.r. 3:2) in 90% yield (171.5 mg) as a white solid. ^{1}H NMR (400 MHz, DMSO-d_6): δ 0.70-0.71 (m, 3H), 0.76-0.87 (m, 2H), 1.05-1.13 (m, 4H), 1.21-1.32 (m, 3H), 1.44-1.54 (m, 7H), 1.63-1.80 (m, 5H), 1.92-1.99 (m, 1H), 2.13-2.24 (m, 2H), 2.34-2.44 (m, 2H), 2.63-2.77 (m, 2H), 2.87 (br, 2H), 2.94-3.03 (m, 1H), 3.06-3.08 (m, 3H), 4.99-5.01 (m, 1H), 5.09 (d, *J* = 6.0 Hz, 0.4H, minor), 5.12 (d, *J* = 6.0 Hz, 0.6H, major), 5.22 (d, *J* = 16.0 Hz, 0.4H, minor), 5.30 (d, *J* = 15.6 Hz, 0.6H, major), 5.36-5.37 (m, 1H), 5.62 (br, 1H), 7.26-7.29 (m, 1H), 7.44 (d, *J* = 7.6 Hz, 1H), 7.61-7.65 (m, 1H), 7.81 (d, *J* = 7.6 Hz, 1H). ^{13}C NMR (151 MHz, $CDCl_3$): δ 12.64, 17.25, 20.55, 22.87, 22.96, 23.17, 27.77, 27.79, 28.01, 30.09, 31.35, 32.51, 32.59, 33.77, 35.52, 36.03, 38.46, 38.47, 38.92, 46.38, 46.55, 46.57, 49.85, 49.88, 51.71, 51.77, 53.37, 53.41, 55.61, 55.69, 79.25, 85.50, 85.57, 85.68, 90.18, 110.52, 110.67, 117.03, 123.72, 123.76, 126.76, 126.81, 127.81, 127.83, 132.88, 132.91, 133.13, 133.15, 140.61,

140.66, 152.48, 152.54, 159.48, 159.51, 162.88, 170.97, 171.05, 199.32, 199.35. IR (neat): 3422, 2941, 2225, 1702, 1650, 1611, 1435, 1377, 1329, 1271, 1229, 1165, 1127, 1105, 1068, 1026, 947, 863, 806, 765, 732, 698, 608, 588, 556, 517, 416 cm^{-1}. HRMS (ESI) calcd. for $C_{39}H_{47}N_4O_4$ $[M+H]^+$: 635.3592, found 635.3592.

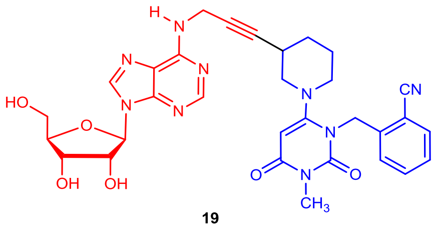

19

2-((6-(3-(3-((9-((2*R*,3*R*,4*S*,5*R*)-3,4-Dihydroxy-5-(hydroxymethyl)tetrahydrofuran-2-yl)-9*H*-purin-6-yl)amino)prop-1-yn-1-yl)piperidin-1-yl)-3-methyl-2,4-dioxo-3,4-dihydropyrimidin-1(2*H*)-yl)methyl)benzonitrile (19). Following **General procedure D**, 0.3 mmol scale, $NiCl_2·6H_2O$ (0.03 mmol, 7.1 mg), **L4** (0.03 mmol, 7.9 mg), anhydrous K_3PO_4 (0.39 mmol, 82.8 mg), 1-(1-(3-(2-cyanobenzyl)-1-methyl-2,6-dioxo-1,2,3,6-tetrahydro-pyrimidin-4-yl)piperidin-3-yl)-2,4,6-triphenylpyridin-1-ium tetrafluoroborate **S7** (0.3 mmol, 215.3 mg), (2*R*,3*S*,4*R*,5*R*)-2-(hydroxymethyl)-5-(6-(prop-2-yn-1-ylamino)-9*H*-purin-9-yl)tetrahydrofuran-3,4-diol **S19** (0.45 mmol, 137.4 mg) and *N*, *N*-dimethyl-formamide (1.5 mL) were stirred at 80 °C for 24 h. Then the reaction mixture was extracted with dichloromethane, washed with water and brine, and dried over anhydrous Na_2SO_4. Purification of the crude product by column chromatography on silica gel which was pretreated with triethylamine before loading the sample (eluent: dichloromethane: methanol = 15:1) afforded the title product in 51% yield (95.1 mg) as a white solid. ^1H NMR (600 MHz, $CDCl_3$): δ 1.51-1.56 (m, 2H), 1.80 (br, 2H), 2.65-2.79 (m, 4H), 2.97 (br, 1H), 3.23 (s, 3H), 3.72-3.73 (m, 1H), 3.90-3.92 (m, 1H), 4.30-4.45 (m, 5H), 4.98 (s, 1H), 5.21-5.31 (m, 4H), 5.86 (s, 1H), 6.71-6.74 (m, 2H), 7.14 (br, 1H), 7.32-7.33 (m, 1H), 7.50-7.51 (m, 1H), 7.60-7.62 (m, 1H), 7.90 (br, 1H), 8.17 (br, 1H). ^{13}C NMR (151 MHz, $CDCl_3$): δ 22.95, 27.91, 28.00, 29.63, 30.35, 46.48, 51.88, 55.46, 63.07, 72.38, 73.78, 78.30, 83.50, 87.58, 90.10, 91.01, 110.56, 117.16, 120.75, 126.72, 127.85, 133.05, 133.22, 140.48, 140.53, 147.51, 152.17, 152.50, 153.99, 159.73, 163.29. IR (neat): 3293, 2925, 2857, 2226, 1700, 1638, 1609, 1512, 1440, 1376, 1334, 1301, 1228, 1178, 1106, 1084,

1035, 1005, 984, 863, 815, 795, 758, 733, 691, 647, 609, 588, 535, 519, 414 cm^{-1}. HRMS (ESI) calcd. for $C_{31}H_{34}N_9O_6$ $[M+H]^+$: 628.2627, found 628.2622.

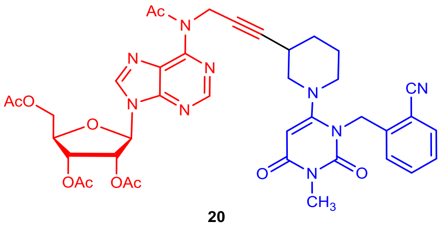

20

(2*R*,3*R*,4*R*,5*R*)-2-(Acetoxymethyl)-5-(6-(*N*-(3-(1-(3-(2-cyanobenzyl)-1-methyl-2,6-dioxo-1,2,3,6-tetrahydropyrimidin-4-yl)piperidin-3-yl)prop-2-yn-1-yl)acetamido)-9*H*-purin-9-yl)tetrahydrofuran-3,4-diyl diacetate (20). Following **General procedure D**, 0.3 mmol scale, $NiCl_2 \cdot 6H_2O$ (0.03 mmol, 7.1 mg), **L4** (0.03 mmol, 7.9 mg), anhydrous K_3PO_4 (0.39 mmol, 82.8 mg), 1-(1-(3-(2-cyanobenzyl)-1-methyl-2,6-dioxo-1,2,3,6-tetrahydropyrimidin-4-yl)piperidin-3-yl)-2,4,6-triphenylpyridin-1-ium tetrafluoroborate **S7** (0.3 mmol, 215.3 mg), (2*R*,3*R*,4*R*,5*R*)-2-(acetoxymethyl)-5-(6-(*N*-(prop-2-yn-1-yl)-acetamido)-9*H*-purin-9-yl)tetrahydrofuran-3,4-diyl diacetate **S20** (0.45 mmol, 213.0 mg) and *N*, *N*-dimethylformamide (1.5 mL) were stirred at 80 °C for 24 h. Then the reaction mixture was extracted with dichloromethane, washed with water and brine, and dried over anhydrous Na_2SO_4. Purification of the crude product by column chromatography on silica gel (eluent: dichloromethane: ethanol = 100:1) afforded a mixture of diastereoisomers (d.r. 10:1) in 88% yield (210.0 mg) as a white solid. ^1H NMR (400 MHz, $CDCl_3$): δ 1.34-1.36 (m, 1H), 1.43-1.49 (m, 1H), 1.62 (br, 1H), 1.74 (br, 1H), 2.08-2.17 (m, 9H), 2.30 (s, 3H), 2.46 (br, 1H), 2.65 (br, 2H), 2.86-2.89 (m, 2H), 3.27-3.29 (m, 3H), 4.38-4.51 (m, 3H), 4.88-4.97 (m, 2H), 5.19-5.36 (m, 3H), 5.70-5.71 (m, 1H), 5.97-5.99 (m, 1H), 6.17 (d, *J* = 4.8 Hz, 0.09H, minor), 6.24 (d, *J* = 4.4 Hz, 0.9H, major), 7.12-7.17 (m, 1H), 7.36 (t, *J* = 7.6 Hz, 1H), 7.54 (t, *J* = 7.6 Hz, 1H), 7.65 (d, *J* = 7.6 Hz, 1H), 7.94 (s, 0.09H, minor), 8.26 (s, 0.9H, major), 8.39 (s, 0.09H, minor), 8.80 (s, 0.9H, major). ^{13}C NMR (151 MHz, $CDCl_3$): δ 20.23, 20.31, 20.56, 22.90, 24.02, 27.61, 27.74, 29.52, 36.35, 45.96, 46.15 (minor), 51.60, 51.73 (minor), 55.29, 62.74, 62.89 (minor), 70.18, 70.39 (minor), 72.87, 79.93 (minor), 80.01, 83.31, 86.01 (minor), 86.77, 90.05, 110.52 (minor), 110.80, 116.97, 126.43 (minor), 126.58, 127.07 (minor), 127.09, 127.69, 132.85, 132.97, 138.36 (minor),

140.39, 140.46 (minor), 142.39, 151.91, 152.23, 152.31, 152.35, 159.33, 159.43 (minor), 162.80, 169.25, 169.39, 170.09, 170.36. IR (neat): 2925, 2861, 2226, 1745, 1703, 1655, 1611, 1433, 1364, 1329, 1227, 1164, 1085, 980, 946, 925, 862, 806, 764, 696, 657, 608, 587, 558, 522, 416 cm^{-1}. HRMS (ESI) calcd. for $C_{39}H_{41}N_9NaO_{10}$ $[M+Na]^+$: 818.2869, found 818.2867.

Gram scale study

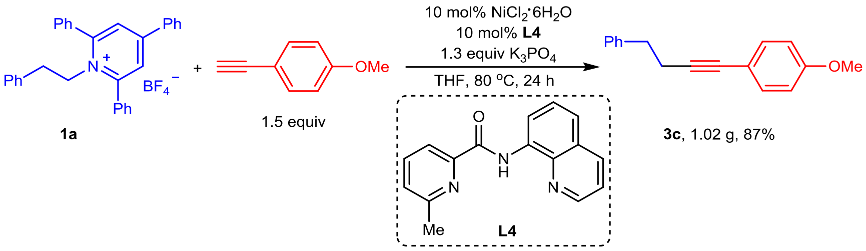

To an oven-dried sealable Schlenk tube (100.0 mL) were added $NiCl_2·6H_2O$ (0.5 mmol, 118.8 mg), **L4** (0.5 mmol, 131.7 mg), anhydrous K_3PO_4 (6.5 mmol, 1.38 g) and phenethylpyridinium salt **1a** (5.0 mmol, 2.5 g) using standard Schlenk technique under nitrogen atmosphere. Subsequently, tetrahydrofuran (25.0 mL) was added via syringe followed by addition of 4-methoxyphenylacetylene (7.5 mmol, 991.2 mg). Then the Schlenk tube was securely sealed and immersed into an oil bath preheated at 80 °C. After stirring for 24 h, the reaction mixture was cooled to room temperature and filtered through a short pad of silica gel. Then the filter cake was washed with dichloromethane. The resulting solution was concentrated under vacuum and the residue was purified by column chromatography on silica gel (eluent: petroleum ether: dichloromethane = 20:1) to afford **3c** in 87% yield (1.02 g) as a colorless oil.

One-pot transformation

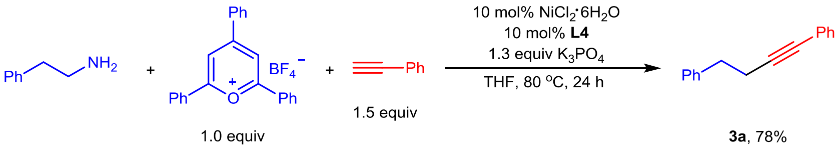

In a nitrogen-filled glovebox, $NiCl_2·6H_2O$ (0.03 mmol, 7.1 mg), **L4** (0.03 mmol, 7.9 mg), anhydrous K_3PO_4 (0.39 mmol, 82.8 mg), 2,4,6-triphenylpyrylium salt (0.3 mmol,

118.9 mg) and tetrahydrofuran (1.5 mL) were successively added to an oven-dried sealable Schlenk tube (10.0 mL). Subsequently, 2-phenylethylamine (0.3 mmol, 36.4 mg) was added via microliter syringe followed by addition of phenylacetylene (0.45 mmol, 46.0 mg). Then the tube was securely sealed and taken outside the glovebox. And it was immersed into an oil bath preheated at 80 °C. After stirring for 24 h, the reaction mixture was cooled to room temperature and filtered through a short pad of silica gel. Then the filter cake was washed with dichloromethane. The resulting solution was concentrated under vacuum and the residue was purified by column chromatography on silica gel (eluent: petroleum ether: dichloromethane = 100:1) to afford **3a** in 78% yield (48.1 mg) as a colorless oil.

Supplementary discussion

Mechanistic studies

Radical trap experiment

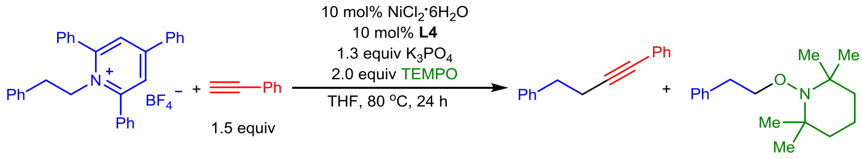

1a **3a**, trace **21**, 16%

In a nitrogen-filled glovebox, $NiCl_2{\cdot}6H_2O$ (0.03 mmol, 7.1 mg), **L4** (0.03 mmol, 7.9 mg), anhydrous K_3PO_4 (0.39 mmol, 82.8 mg), phenethylpyridinium salt **1a** (0.3 mmol, 150.0 mg), 2,2,6,6-tetramethylpiperidine-*N*-oxyl (TEMPO, 0.6 mmol, 93.8 mg) and tetrahydrofuran (1.5 mL) were successively added to an oven-dried sealable Schlenk tube (10.0 mL) followed by addition of phenylacetylene (0.45 mmol, 46.0 mg) via microliter syringe. Then the tube was securely sealed and taken outside the glovebox. And it was immersed into an oil bath preheated at 80 °C. After stirring for 24 h, the reaction mixture was cooled to room temperature and filtered through a short pad of silica gel. Then the filter cake was washed with dichloromethane. The resulting solution was concentrated under vacuum and the residue was purified by preparative TLC on silica gel (eluent: petroleum ether: dichloromethane = 100:1) to afford TEMPO-trapped adduct **21** in 16% yield (12.5 mg) as a colorless oil, while the normal cross-coupling product **3a** was obtained

in only 3% yield (1.7 mg).

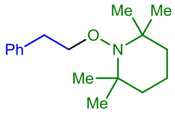

21

2,2,6,6-Tetramethyl-1-phenethoxypiperidine (21). ^{1}H NMR (600 MHz, $CDCl_3$): δ 1.07 (s, 12H), 1.26-1.31 (m, 1H), 1.41-1.42 (m, 4H), 1.53-1.54 (m, 1H), 2.82 (t, J = 6.6 Hz, 2H), 3.94 (t, J = 6.6 Hz, 2H), 7.18 (t, J = 7.2 Hz, 1H), 7.23 (d, J = 7.2 Hz, 2H), 7.27 (t, J = 7.2 Hz, 2H). ^{13}C NMR (151 MHz, $CDCl_3$): δ 17.11, 20.11, 32.91, 35.37, 39.59, 59.68, 77.46, 125.89, 128.09, 129.10, 139.62. IR (neat): 2975, 2929, 2872, 1496, 1470, 1454, 1373, 1359, 1262, 1246, 1184, 1133, 1083, 1064, 1032, 994, 957, 750, 710, 697, 557, 512 cm^{-1}. HRMS (ESI) calcd. for $C_{17}H_{28}NO$ [M+H]$^{+}$: 262.2165, found 262.2166.

Radical clock experiment

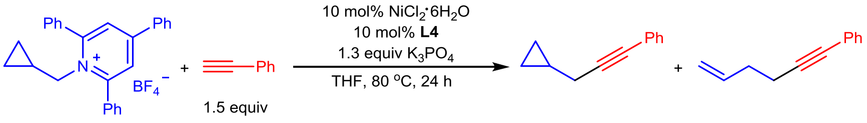

In a nitrogen-filled glovebox, $NiCl_2{\cdot}6H_2O$ (0.03 mmol, 7.1 mg), **L4** (0.03 mmol, 7.9 mg), anhydrous K_3PO_4 (0.39 mmol, 82.8 mg), 1-(cyclopropylmethyl)-2,4,6-triphenylpyridin-1-ium tetrafluoroborate **1x** (0.3 mmol, 134.8 mg) and tetrahydrofuran (1.5 mL) were successively added to an oven-dried sealable Schlenk tube (10.0 mL) followed by addition of phenylacetylene (0.45 mmol, 46.0 mg) via microliter syringe. Then the tube was securely sealed and taken outside the glovebox. And it was immersed into an oil bath preheated at 80 °C. After stirring for 24 h, the reaction mixture was cooled to room temperature and filtered through a short pad of silica gel. Then the filter cake was washed with dichloromethane. The resulting solution was concentrated under vacuum and the residue was purified by column chromatography on silica gel (eluent: petroleum ether) to afford the ring-opened product **22** in 80% yield (37.6 mg) as a colorless oil, and no cross-coupling product **4x** was observed. Due to the lower boiling point of product **22**, the NMR yield was determined to be 95% by ^{1}H NMR analysis of the crude mixture using

1,3,5-trimethoxybenzene (0.3 mmol, 50.5 mg) as an internal standard.

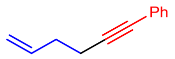

22

Hex-5-en-1-yn-1-ylbenzene (22). ^{1}H NMR (400 MHz, $CDCl_3$): δ 2.33-2.38 (m, 2H), 2.49 (t, *J* = 7.2 Hz, 2H), 5.06 (d, *J* = 10.0 Hz, 1H), 5.12 (d, *J* = 17.2 Hz, 1H), 5.87-5.97 (m, 1H), 7.23-7.29 (m, 3H), 7.38-7.40 (m, 2H). ^{13}C NMR (100 MHz, $CDCl_3$): δ 19.23, 32.93, 80.99, 89.47, 115.67, 123.88, 127.54, 128.15, 131.54, 136.93. IR (neat): 3080, 2925, 1642, 1599, 1490, 1442, 1333, 995, 914, 755, 691, 537, 451 cm^{-1}. HRMS (EI) calcd. for $C_{12}H_{12}$ $[M]^+$: 156.0934, found 156.0938.

Synthesis of Ni complex Int-1

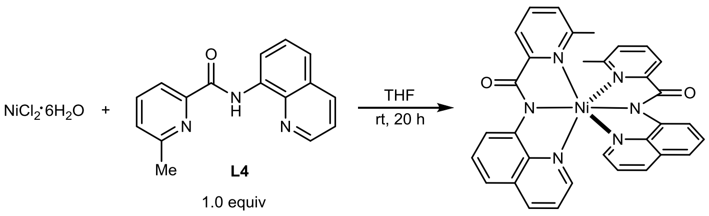

To an oven-dried round-bottomed flask (25.0 mL) were added $NiCl_2\cdot 6H_2O$ (0.3 mmol, 71.3 mg), **L4** (0.3 mmol, 79.0 mg) and tetrahydrofuran (15.0 mL). Then the reaction mixture was stirred at room temperature for 20 h, and a light-brown suspension was obtained. Then it was filtrated and washed with tetrahydrofuran, the residue was dried under high vacuum at room temperature to afford **Int-1** in 78% yield (67.9 mg) as a yellow solid. ^{1}H NMR (400 MHz, CD_3OD): δ 2.70 (s, 6H), 7.57 (d, *J* = 7.6 Hz, 2H), 7.79-7.86 (m, 4H), 7.96-8.00 (m, 4H), 8.10 (d, *J* = 7.6 Hz, 2H), 8.50 (d, *J* = 7.6 Hz, 2H), 8.80 (d, *J* = 8.4 Hz, 2H), 9.03 (d, *J* = 4.0 Hz, 2H). IR (neat): 3186, 1638, 1580, 1561, 1500, 1463, 1428, 1409, 1392, 1319, 1300, 1255, 1050, 1008, 952, 821, 759, 704, 679, 617, 562, 500, 449 cm^{-1}. HRMS (ESI) calcd. for $C_{32}H_{25}N_6NiO_2$ $[M+H]^+$: 583.1387, found 583.1387.

Ni complex Int-1 catalyzed deaminative Sonogashira coupling of 1a

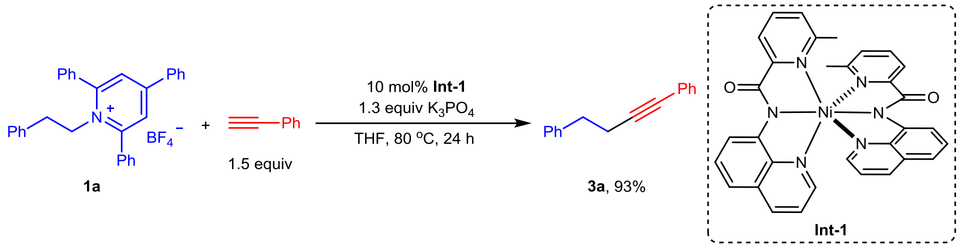

In a nitrogen-filled glovebox, **Int-1** (0.03 mmol, 17.5 mg), anhydrous K_3PO_4 (0.39 mmol, 82.8 mg), phenethylpyridinium salt **1a** (0.3 mmol, 150.0 mg) and tetrahydrofuran (1.5 mL) were successively added to an oven-dried sealable Schlenk tube (10.0 mL) followed by addition of phenylacetylene (0.45 mmol, 46.0 mg) via microliter syringe. Then the tube was securely sealed and taken outside the glovebox. And it was immersed into an oil bath preheated at 80 °C. After stirring for 24 h, the reaction mixture was cooled to room temperature and filtered through a short pad of silica gel. Then the filter cake was washed with dichloromethane. The resulting solution was concentrated under vacuum and the residue was purified by column chromatography on silica gel (eluent: petroleum ether: dichloromethane = 100:1) to afford **3a** in 93% yield (57.6 mg) as a colorless oil.

Ni(cod)2 catalyzed deaminative Sonogashira coupling of 1a

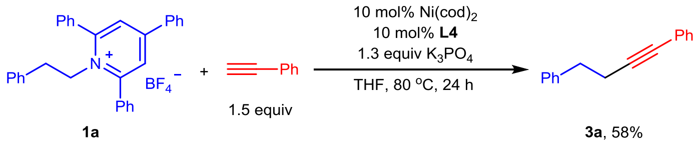

In a nitrogen-filled glovebox, $Ni(cod)_2$ (0.03 mmol, 8.3 mg), **L4** (0.03 mmol, 7.9 mg), anhydrous K_3PO_4 (0.39 mmol, 82.8 mg), phenethylpyridinium salt **1a** (0.3 mmol, 150.0 mg) and tetrahydrofuran (1.5 mL) were successively added to an oven-dried sealable Schlenk tube (10.0 mL) followed by addition of phenylacetylene (0.45 mmol, 46.0 mg) via microliter syringe. Then the tube was securely sealed and taken outside the glovebox. And it was immersed into an oil bath preheated at 80 °C. After stirring for 24 h, the reaction mixture was cooled to room temperature and filtered through a short pad of silica gel. Then the filter cake was washed with dichloromethane. The resulting solution was concentrated under vacuum and the residue was purified by column chromatography on silica gel

(eluent: petroleum ether: dichloromethane = 100:1) to afford **3a** in 58% yield (36.1 mg) as a colorless oil. *(Note: in this case, the reaction mixture turned out to be more complicated, and the competitive alkyne cyclotrimerization and/or oligomerization products catalyzed by low-valent nickel species was observed.)*

Synthesis of complex A1

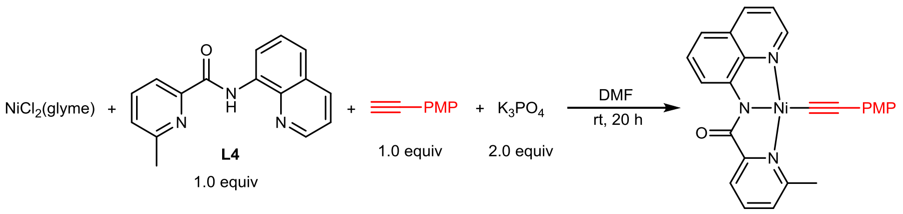

Complex **A1**, 47%

In a nitrogen-filled glovebox, $NiCl_2$(glyme) (1.0 mmol, 220.0 mg), **L4** (1.0 mmol, 263.3 mg), *p*-methoxyphenylethyne (1.0 mmol, 132.2 mg), anhydrous K_3PO_4 (2.0 mmol, 424.5 mg) and *N*, *N*-dimethylformamide (20.0 mL) were successively added to an oven-dried round-bottomed flask (100.0 mL). Then the reaction mixture was stirred at room temperature for 20 h, and a dark-brown solution was obtained. After evaporation of the solvent, the resulting solid was dissolved in toluene (ca. 200 mL). Then filtration was needed to remove the insoluble salts, and a clear wine-red solution was obtained. The excess solvent was evaporated under the reduced pressure to afford a saturated solution. After storing at -20 °C for one night, a large amount of precipitation was observed. Then it was filtrated and washed with minimum amount of toluene, the residue was dried under high vacuum at room temperature to give the complex **A1** in 47% yield (212.6 mg) as a deep yellow solid. And the eligible crystal of complex **A1** used for X-ray diffraction was obtained by recrystallization from a mixed solvent of dichloromethane and *n*-pentane at -20 °C. ^{1}H NMR (600 MHz, C_6D_6): δ 2.92 (s, 3H), 3.30 (s, 3H), 6.14 (dd, *J* = 1.8, 7.8 Hz, 1H), 6.31 (dd, *J* = 5.4, 8.1 Hz, 1H), 6.66 (t, *J* = 7.8 Hz, 1H), 6.70 (dd, *J* = 0.6, 7.8 Hz, 1H), 6.83-6.84 (m, 2H), 7.14-7.18 (m, 2H), 7.62-7.64 (m, 2H), 7.72 (dd, *J* = 1.2, 7.2 Hz, 1H), 9.22 (dd, *J* = 1.2, 7.8 Hz, 1H), 9.36 (dd, *J* = 1.2, 5.4 Hz, 1H). IR (neat): 2949, 2825, 2106, 1635, 1606, 1577, 1502, 1468, 1417, 1390, 1371, 1319, 1281, 1237, 1209, 1170, 1157, 1104, 1033, 970, 827, 818, 780, 762, 728, 701, 630, 580, 533, 508 cm^{-1}. HRMS (MALDI-TOF-DCTB) calcd. for $C_{25}H_{19}N_3NiO_2$ $[M]^+$: 451.0831, found 451.0816.

Complex A1 catalyzed deaminative Sonogashira coupling of 1a

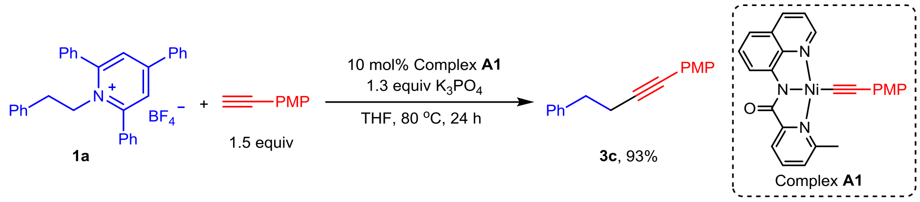

In a nitrogen-filled glovebox, complex **A1** (0.03 mmol, 13.6 mg), anhydrous K_3PO_4 (0.39 mmol, 82.8 mg), phenethylpyridinium salt **1a** (0.3 mmol, 150.0 mg) and tetrahydrofuran (1.5 mL) were successively added to an oven-dried sealable Schlenk tube (10.0 mL) followed by addition of *p*-methoxyphenylethyne (0.45 mmol, 59.5 mg) via microliter syringe. Then the tube was securely sealed and taken outside the glovebox. And it was immersed into an oil bath preheated at 80 °C. After stirring for 24 h, the reaction mixture was cooled to room temperature and filtered through a short pad of silica gel. Then the filter cake was washed with dichloromethane. The resulting solution was concentrated under vacuum and the residue was purified by column chromatography on silica gel (eluent: petroleum ether: dichloromethane = 10:1) to afford **3c** in 93% yield (66.2 mg) as a colorless oil.

Stoichiometric reaction of complex A1 with 1a

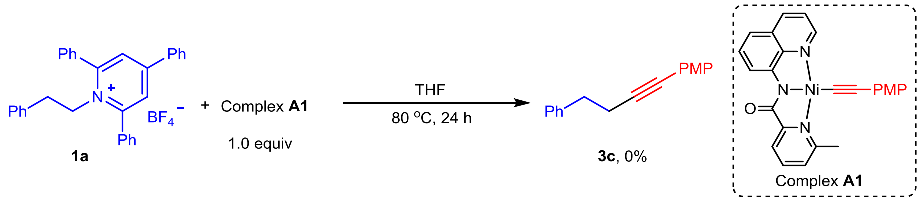

In a nitrogen-filled glovebox, complex **A1** (0.2 mmol, 90.4 mg), phenethylpyridinium salt **1a** (0.2 mmol, 100.0 mg) and tetrahydrofuran (10.0 mL) were successively added to an oven-dried sealable Schlenk tube (15.0 mL). Then the tube was securely sealed and taken outside the glovebox. And it was immersed into an oil bath preheated at 80 °C. After stirring for 24 h, the reaction mixture was cooled to room temperature and filtered through a short pad of silica gel. Then the filter cake was washed with dichloromethane. The resulting solution was concentrated under vacuum and the residue was dissolved in $CDCl_3$. The yield of **3c** was determined to be 0% by ^{1}H NMR analysis of the crude mixture using 1,3,5-trimethoxybenzene (0.2 mmol, 33.6 mg) as an internal standard. *This result indicates*

*that complex **A1** itself does not have the competence to react with **1a**, and it might act as a dormant species in the current catalysis.*

Stoichiometric reaction of complex A1 with 1a in the presence of alkyne and base

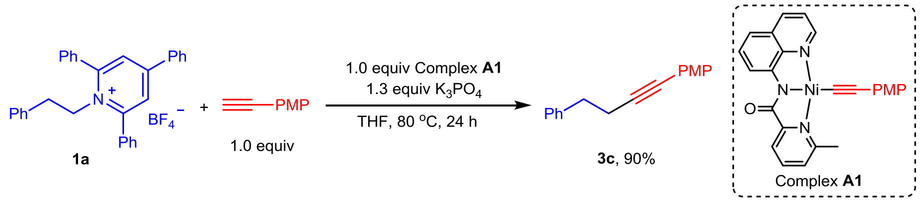

In a nitrogen-filled glovebox, complex **A1** (0.2 mmol, 90.4 mg), anhydrous K_3PO_4 (0.26 mmol, 55.2 mg), phenethylpyridinium salt **1a** (0.2 mmol, 100.0 mg) and tetrahydrofuran (10.0 mL) were successively added to an oven-dried sealable Schlenk tube (15.0 mL) followed by addition of *p*-methoxyphenylethyne (0.2 mmol, 26.4 mg) via microliter syringe. Then the tube was securely sealed and taken outside the glovebox. And it was immersed into an oil bath preheated at 80 °C. After stirring for 24 h, the reaction mixture was cooled to room temperature and filtered through a short pad of silica gel. Then the filter cake was washed with dichloromethane. The resulting solution was concentrated under vacuum and the residue was purified by preparative TLC on silica gel (eluent: petroleum ether: dichloromethane = 10:1) to afford **3c** in 90% yield (42.6 mg) as a colorless oil. *This result implies that the dormant complex **A1** could be futher converted into an activated species by reaction with additional alkyne and base.*

Crossover experiment of complex A1 and *p*-methylphenylacetylene with 1a

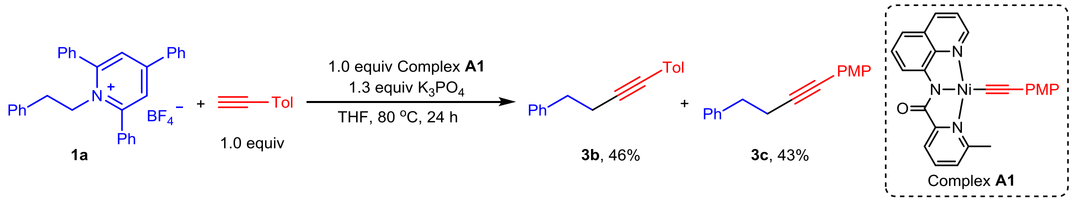

In a nitrogen-filled glovebox, complex **A1** (0.2 mmol, 90.4 mg), anhydrous K_3PO_4 (0.26 mmol, 55.2 mg), phenethylpyridinium salt **1a** (0.2 mmol, 100.0 mg) and tetrahydrofuran (10.0 mL) were successively added to an oven-dried sealable Schlenk tube (15.0 mL) followed by addition of *p*-methylphenylacetylene (0.2 mmol, 23.2 mg) via microliter syringe. Then the tube was securely sealed and taken outside the glovebox. And

it was immersed into an oil bath preheated at 80 °C. After stirring for 24 h, the reaction mixture was cooled to room temperature and filtered through a short pad of silica gel. Then the filter cake was washed with dichloromethane. The resulting solution was concentrated under vacuum and the residue was purified by preparative TLC on silica gel (eluent: petroleum ether: dichloromethane = 50:1 to 10:1, gradient) to afford **3b** in 46% yield (20.3 mg) and **3c** in 43% yield (20.3 mg) as a colorless oil, respectively. *Notably, the different alkynyl fragments from complex **A1** and p-methylphenylacetylene were transferred to the products in a similar probability, which means that the two alkynyl groups ligated on the active Ni-species are equivalent and/or exchangeable.*

Synthesis of complex A2

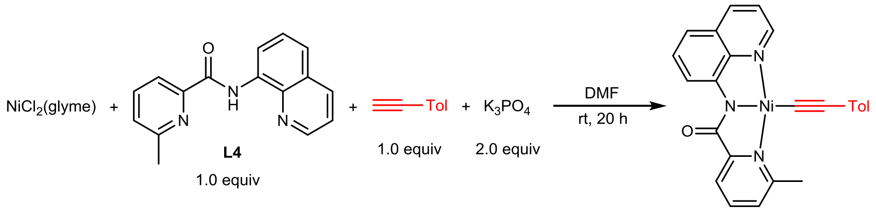

Complex **A2**, 42%

According to the procedure for the synthesis of complex **A1**, in a nitrogen-filled glovebox, $NiCl_2$(glyme) (0.5 mmol, 109.9 mg), **L4** (0.5 mmol, 131.7 mg), *p*-methylphenylacetylene (0.5 mmol, 58.1 mg), anhydrous K_3PO_4 (1.0 mmol, 212.3 mg) and *N*, *N*-dimethylformamide (10.0 mL) were successively added to an oven-dried round-bottomed flask (100.0 mL). Then the reaction mixture was stirred at room temperature for 20 h, and a dark-brown solution was obtained. After evaporation of the solvent, the resulting solid was dissolved in toluene (ca. 100 mL). Then filtration was needed to remove the insoluble salts, and a clear wine-red solution was obtained. The excess solvent was evaporated under the reduced pressure to afford a saturated solution. After storing at -20 °C for one night, a large amount of precipitation was observed. Then it was filtrated and washed with minimum amount of toluene, the residue was dried under high vacuum at room temperature to give the complex **A2** in 42% yield (91.0 mg) as an orange solid. ^{1}H NMR (400 MHz, C_6D_6): δ 2.14 (s, 3H), 2.90 (s, 3H), 6.12 (dd, J = 1.6, 7.6 Hz, 1H), 6.29 (dd, J = 5.2, 8.0 Hz, 1H), 6.65 (t, J = 7.6 Hz, 1H), 6.69 (dd, J = 0.8, 8.0 Hz, 1H), 7.04 (d, J = 8.0 Hz, 2H), 7.12-7.19 (m, 2H), 7.63-7.65 (m, 2H), 7.71 (dd, J = 1.2, 7.6

Hz, 1H), 9.21 (dd, J = 1.2, 7.6 Hz, 1H), 9.34 (dd, J = 1.2, 5.4 Hz, 1H). IR (neat): 3064, 2111, 1636, 1604, 1574, 1505, 1467, 1427, 1409, 1389, 1363, 1318, 1235, 1221, 1203, 1164, 1157, 1095, 1034, 1012, 971, 900, 864, 826, 789, 756, 699, 629, 584, 531, 519, 508, 456, 409 cm^{-1}. HRMS (MALDI-TOF-DCTB) calcd. for $C_{25}H_{19}N_3NiO$ $[M]^+$: 435.0882, found 435.0862.

Alkyne exchange experiment between complex A1 and *p*-methylphenylacetylene

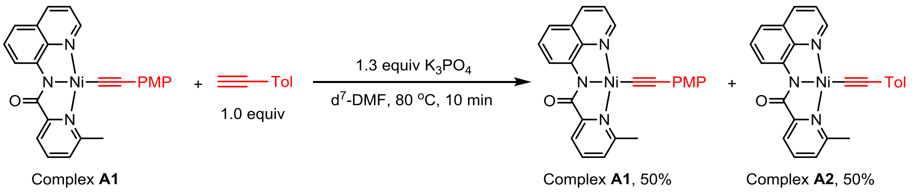

In a nitrogen-filled glovebox, complex **A1** (0.01 mmol, 4.5 mg), anhydrous K_3PO_4 (0.013 mmol, 2.8 mg), *p*-methylphenylacetylene (0.01 mmol, 1.2 mg) and d^7-DMF (0.6 mL) were successively added to an NMR tube containing a screw-cap. Then the NMR tube was securely sealed and taken outside the glovebox. And it was immersed into an oil bath preheated at 80 °C. The conversion between complex **A1** and *p*-methylphenylacetylene was recorded by ^{1}H NMR analysis of the reaction mixture in time. Figure S1 shows the outcomes of the alkyne exchange process. Obviously, complex **A2** was observed immediately by comparision the new formed signal with the standard ^{1}H NMR spectrum of **A2**, and it could quickly reach an equilibrium with complex **A1** in a roughly 1:1 ratio (ca. 10 minutes). *This result suggests that the alkynyl ligated on complex **A1** is exchangeable and a rapid alkynyl exchange reaction is probably involved under catalytically relevant conditions.*

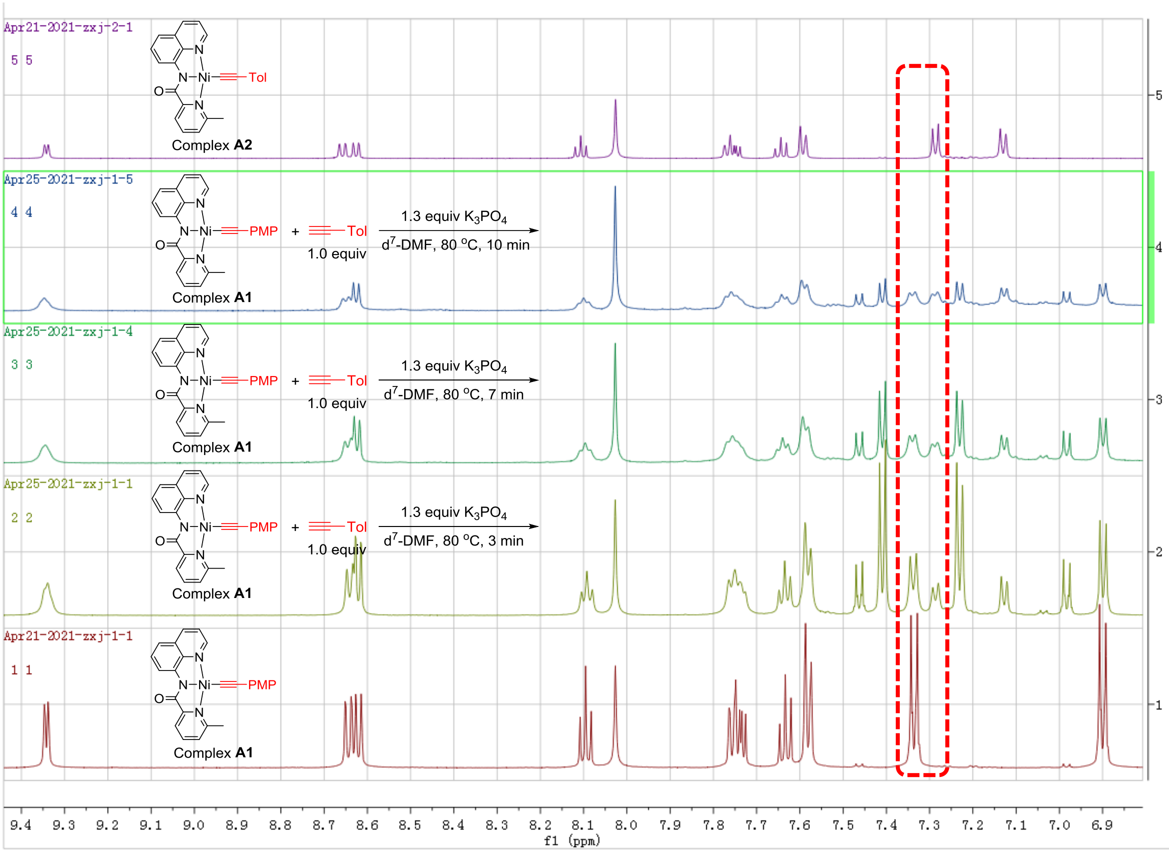

Supplementary Figure 1. NMR study of the alkyne exchange process; the featured signals were highlighted in the dashed box.

Cyclic voltammetry studies

To a dry electrolytic cell equipped with a platinum working electrode, platinum wire as the counter electrode and saturated calomel electrode (SCE) as the reference electrode, was added a solution of sample (1.0 mM) and $(n\text{-Bu})_4NClO_4$ (0.1 M) in anhydrous DMF. Then it was degassed thoroughly by argon bubbling before the measurement, and the cyclic voltammetry was performed under argon atmosphere at room temperature with a scan rate of 50 mV s^{-1}. Figure S2 shows two reversible reduction waves of **1a** in DMF, and the first one is at E_{red} = -0.90 V vs SCE. Figure S3 shows one reduction and two oxidation waves of complex **A1** in DMF. The reversible reduction potential is at $E_{1/2}^{red}$ = -1.35 V vs SCE, which is similar to the result of $E_{1/2}^{red}$ $[Ni^{II}/Ni^{0}]$ = -1.2 V vs SCE in DMF[9,10]. While the irreversible oxidation wave at E_{oxi} = 1.19 V vs SCE suggests a one-electron oxidation of Ni^{II} to form Ni^{III} is thermodynamically unfavorable. Figure S4 shows an irreversible oxidation wave of blank experiment at E_{oxi} = 0.92 V vs SCE.

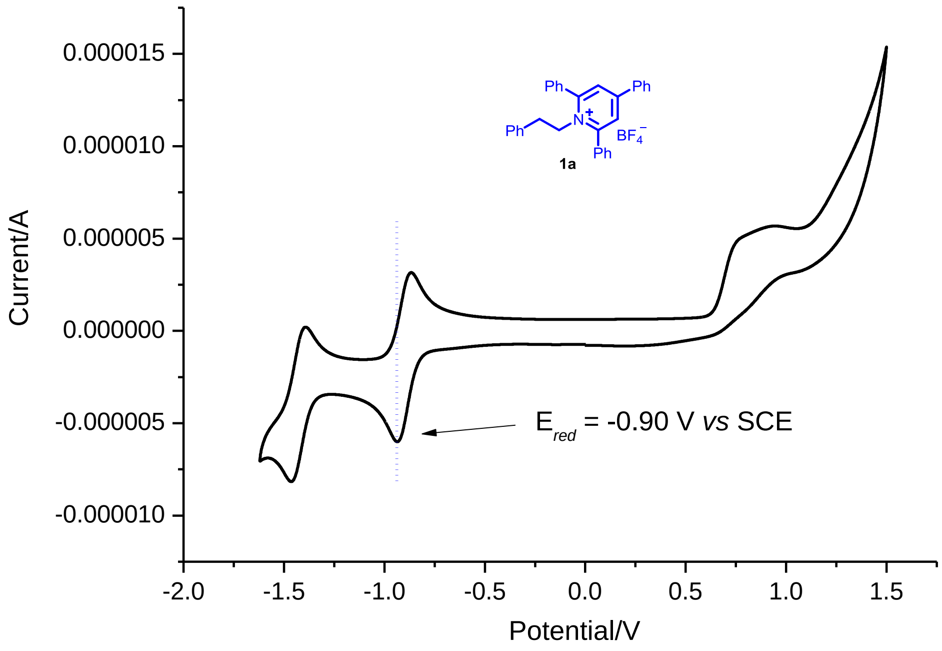

Supplementary Figure 2. Cyclic voltammogram of **1a**

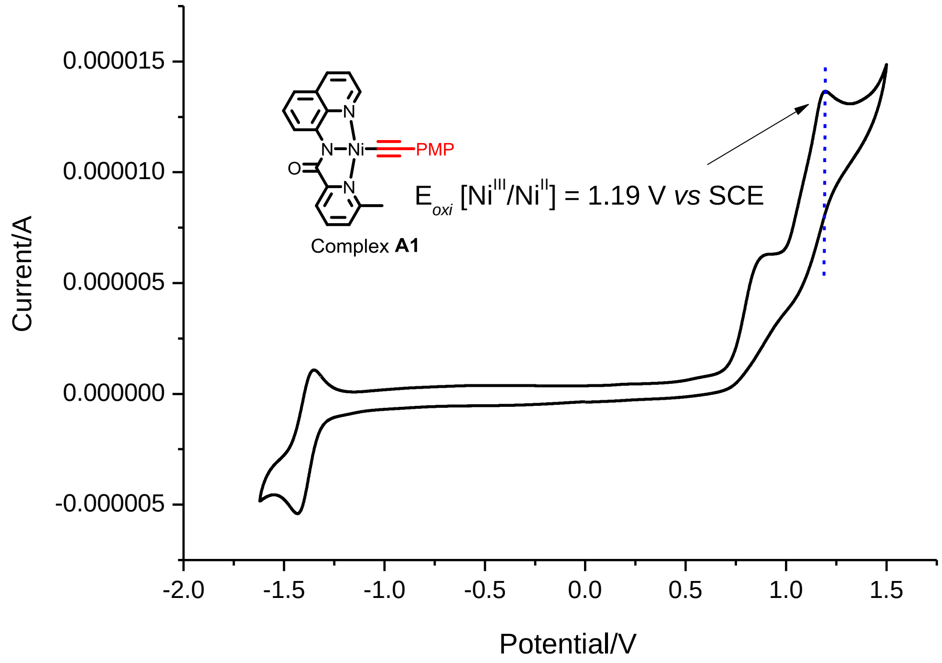

Supplementary Figure 3. Cyclic voltammogram of complex **A1**

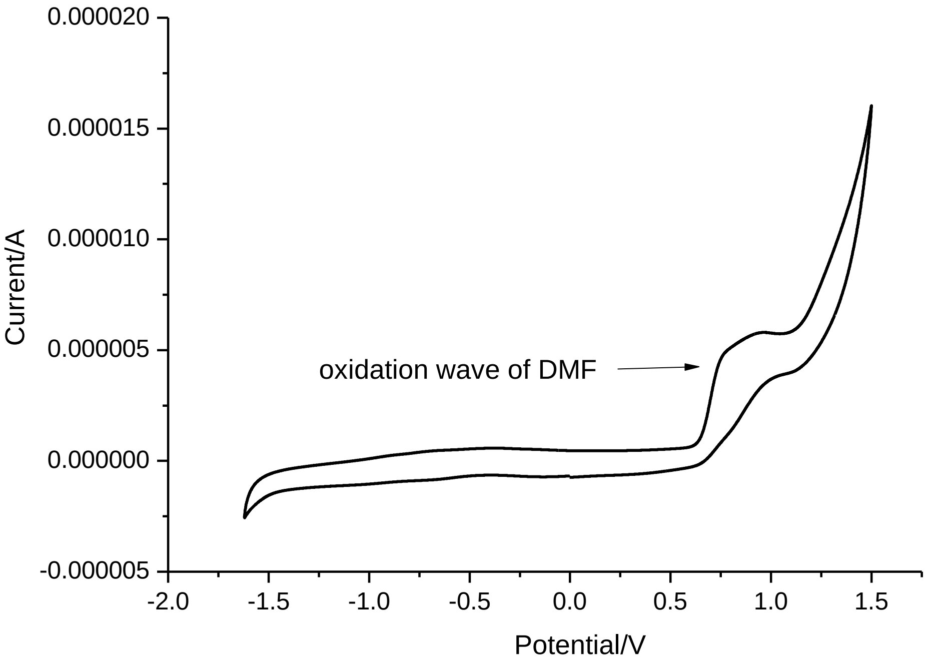

Supplementary Figure 4. Cyclic voltammogram of blank experiment

Supplementary references:

[1] Zhu, Z.-F., Zhang, M.-M. & Liu, F. Radical alkylation of isocyanides with amino acid-/peptide-derived Katritzky salts via photoredox catalysis. *Org. Biomol. Chem.* **17**, 1531-1534 (2019).

[2] Basch, C. H., Liao, J., Xu, J., Piane, J. J. & Watson, M. P. Harnessing alkyl amines as electrophiles for nickel-catalyzed cross couplings via C–N bond activation. *J. Am. Chem. Soc.* **139**, 5313-5316 (2017).

[3] Wu, J., He, L., Noble, A. & Aggarwal, V. K. Photoinduced deaminative borylation of alkylamines. *J. Am. Chem. Soc.* **140**, 10700-10704 (2018).

[4] Klauck, F. J. R., James, M. J. & Glorius, F. Deaminative strategy for the visible-light-mediated generation of alkyl radicals. *Angew. Chem. Int. Ed.* **56**, 12336-12339 (2017).

[5] Ito, H., Kamachi, T. & Yashima, E. Specific surface modification of the acetylene-linked glycolipid vesicle by click chemistry. *Chem. Commun.* **48**, 5650-5652 (2012).

[6] Wang, X. et al. Anticancer-active N-heteroaryl amines syntheses: nucleophilic amination of N-heteroaryl alkyl ethers with amines. *Org. Lett.* **21**, 5111-5115 (2019).

[7] Liu, E.-C. & Topczewski, J. J. Enantioselective copper catalyzed alkyne-azide cycloaddition by dynamic kinetic resolution. *J. Am. Chem. Soc.* **141**, 5135-5138 (2019).

[8] Tararov, V. I., Kolyachkina, S. V., Alexeev, C. S. & Mikhailov, S. N. N^6-Acetyl-2',3',5'-tri-*O*-acetyladenosine; a convenient, 'missed out' substrate for regioselective N^6-alkylations. *Synthesis* **15**, 2483-2489 (2011).

[9] Zuo, Z. et al. Merging photoredox with nickel catalysis: coupling of α-carboxyl sp^3-carbons with aryl halides. *Science* **345**, 437-440 (2014).

[10] Henne, J. B. & Bartak, D. E. Metal-vapor synthesis and electrochemistry of bis(bipyridyl)nickel(0). *Inorg. Chem.* **23**, 369-373 (1984).

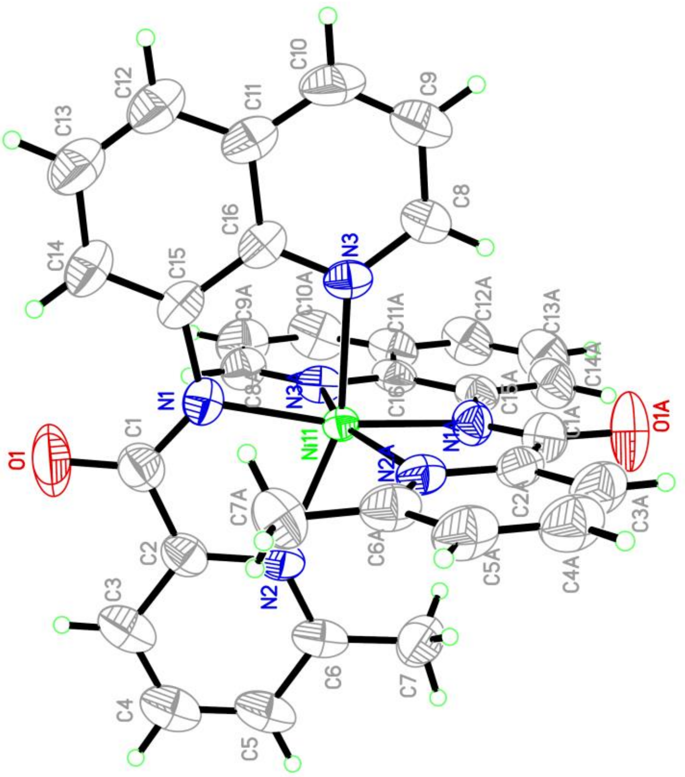

Supplementary Figure 5. X-ray crystal structure of Ni complex **Int-1**

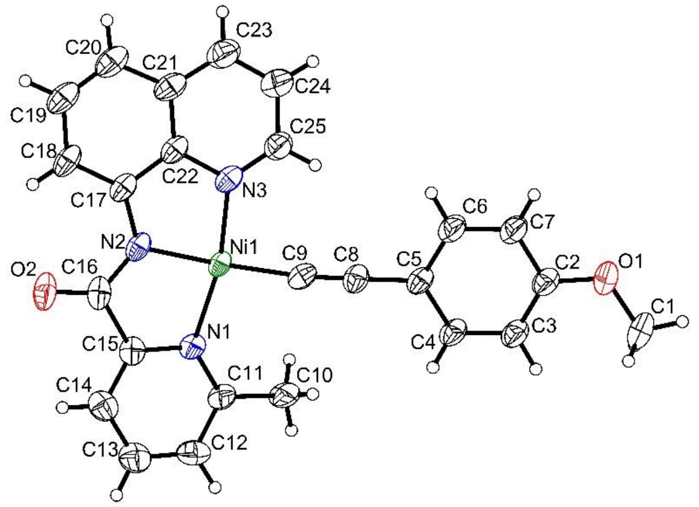

Supplementary Figure 6. X-ray crystal structure of complex **A1**

NMR spectra of all new compounds

Supplementary Figure 7. ^{1}H NMR(400 MHz, $CDCl_3$)

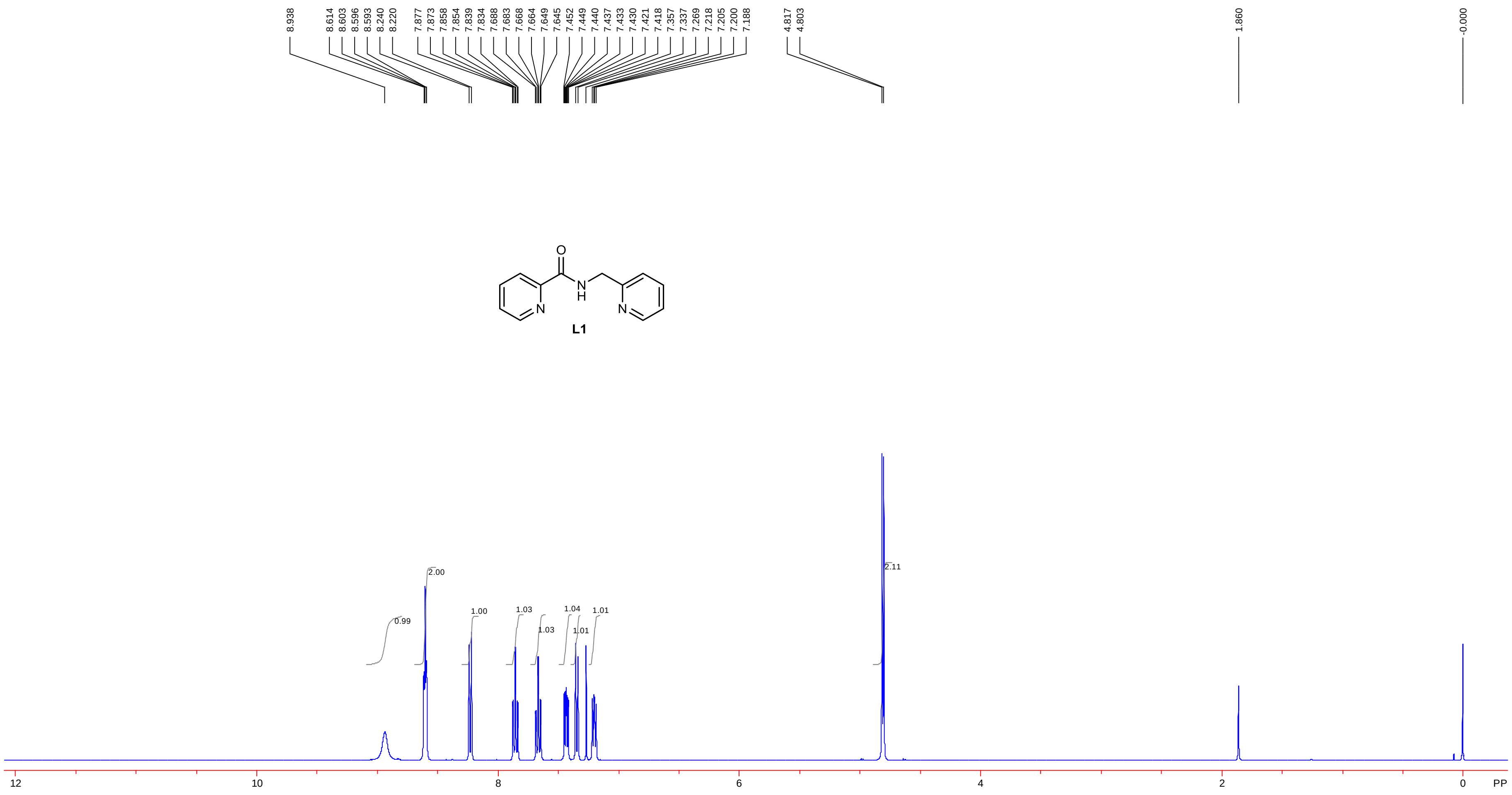

Supplementary Figure 8. ^{13}C NMR(100 MHz, $CDCl_3$)

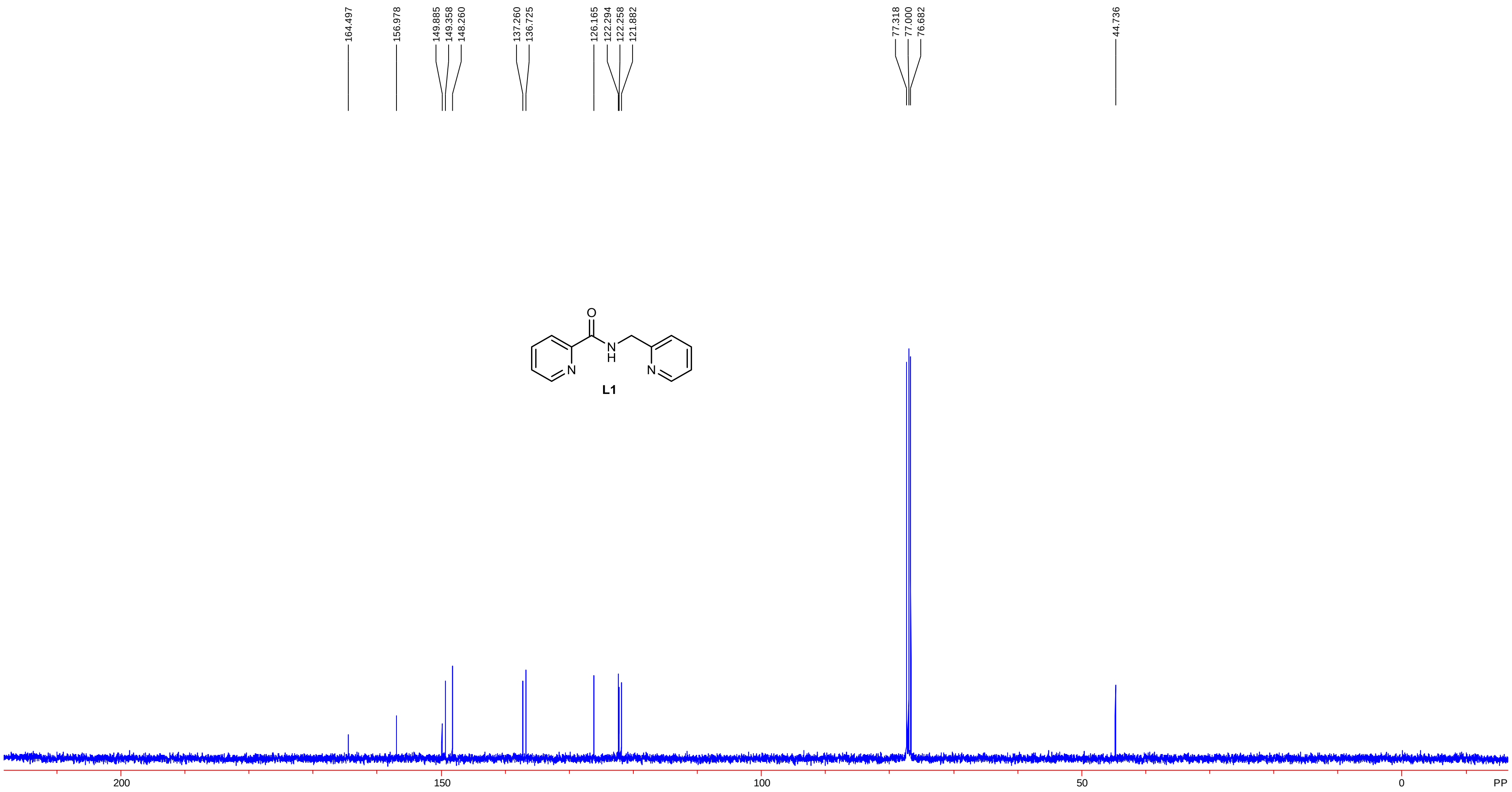

Supplementary Figure 9. ^{1}H NMR(400 MHz, $CDCl_3$)

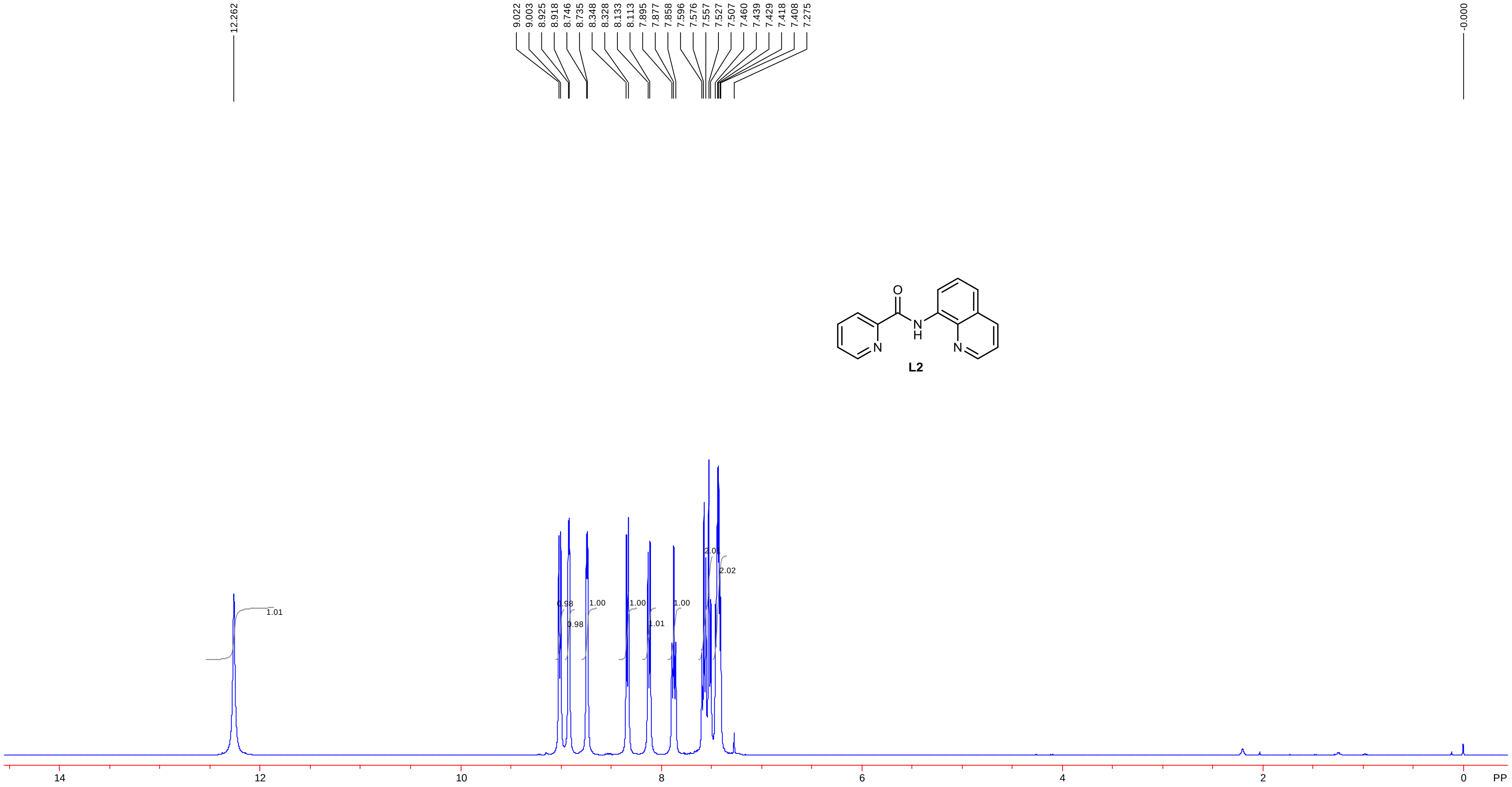

Supplementary Figure 10. ^{13}C NMR(100 MHz, $CDCl_3$)

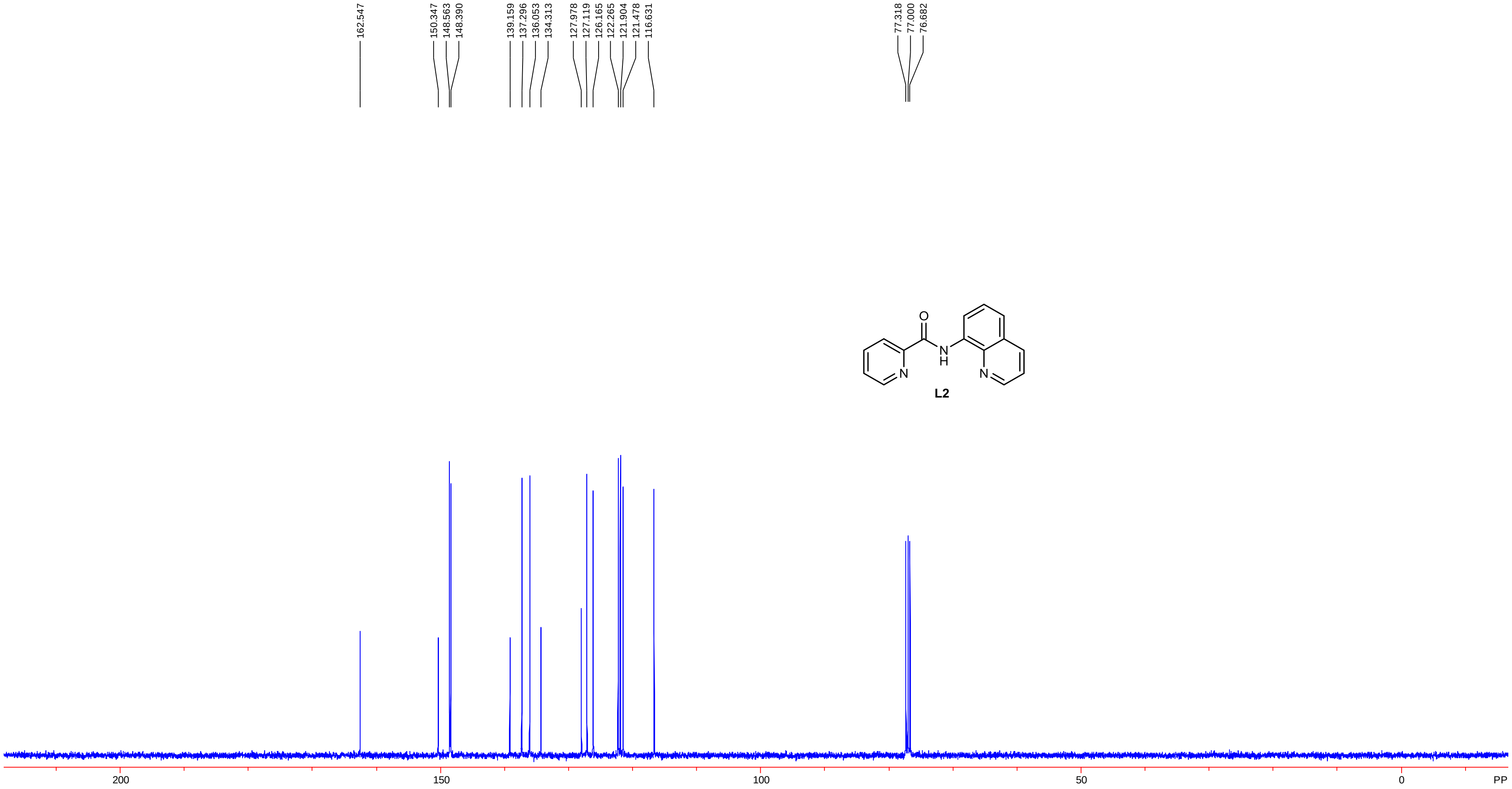

L2

Supplementary Figure 11. ^{1}H NMR(400 MHz, $CDCl_3$)

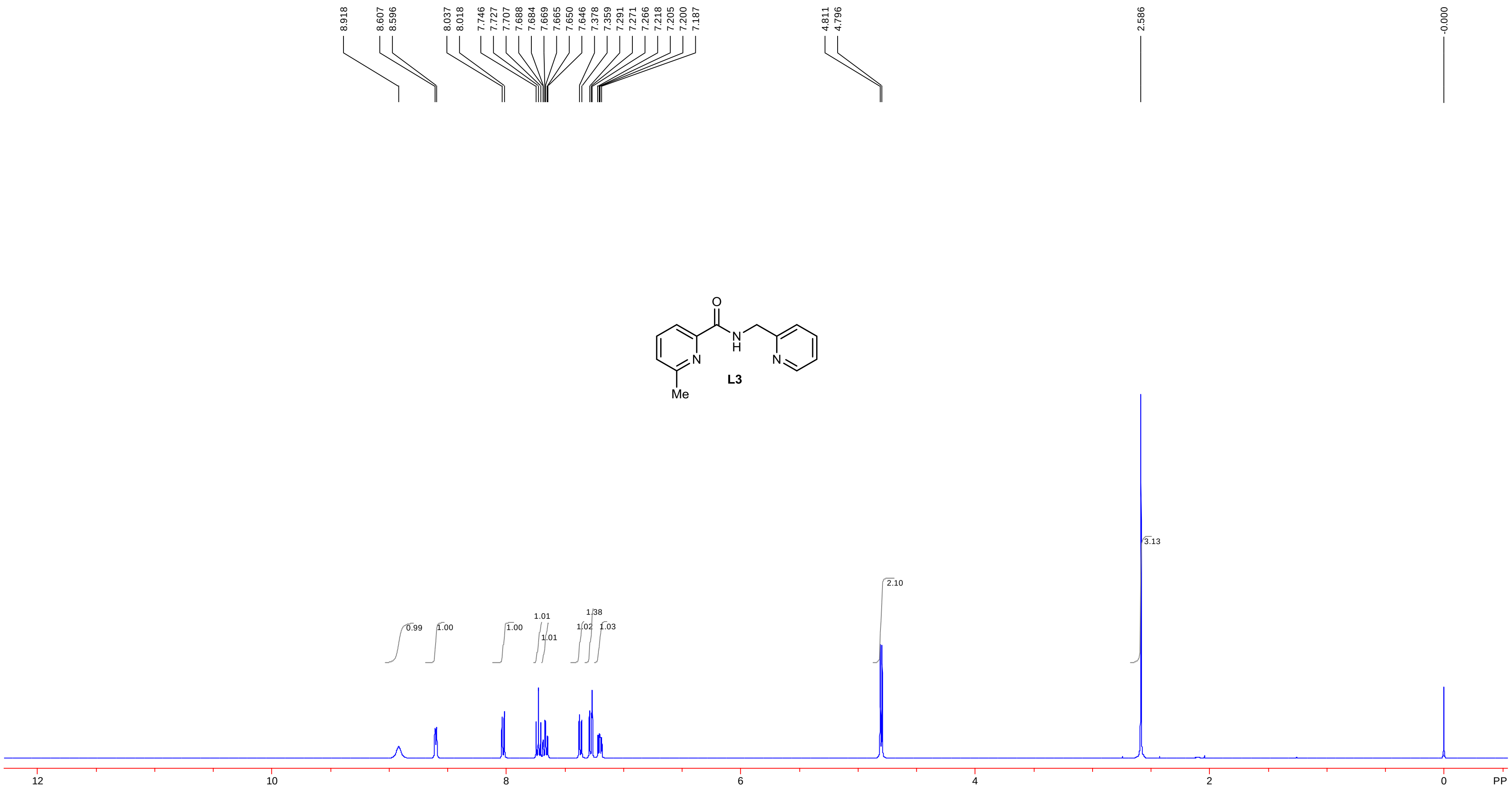

Supplementary Figure 12. ^{13}C NMR(100 MHz, $CDCl_3$)

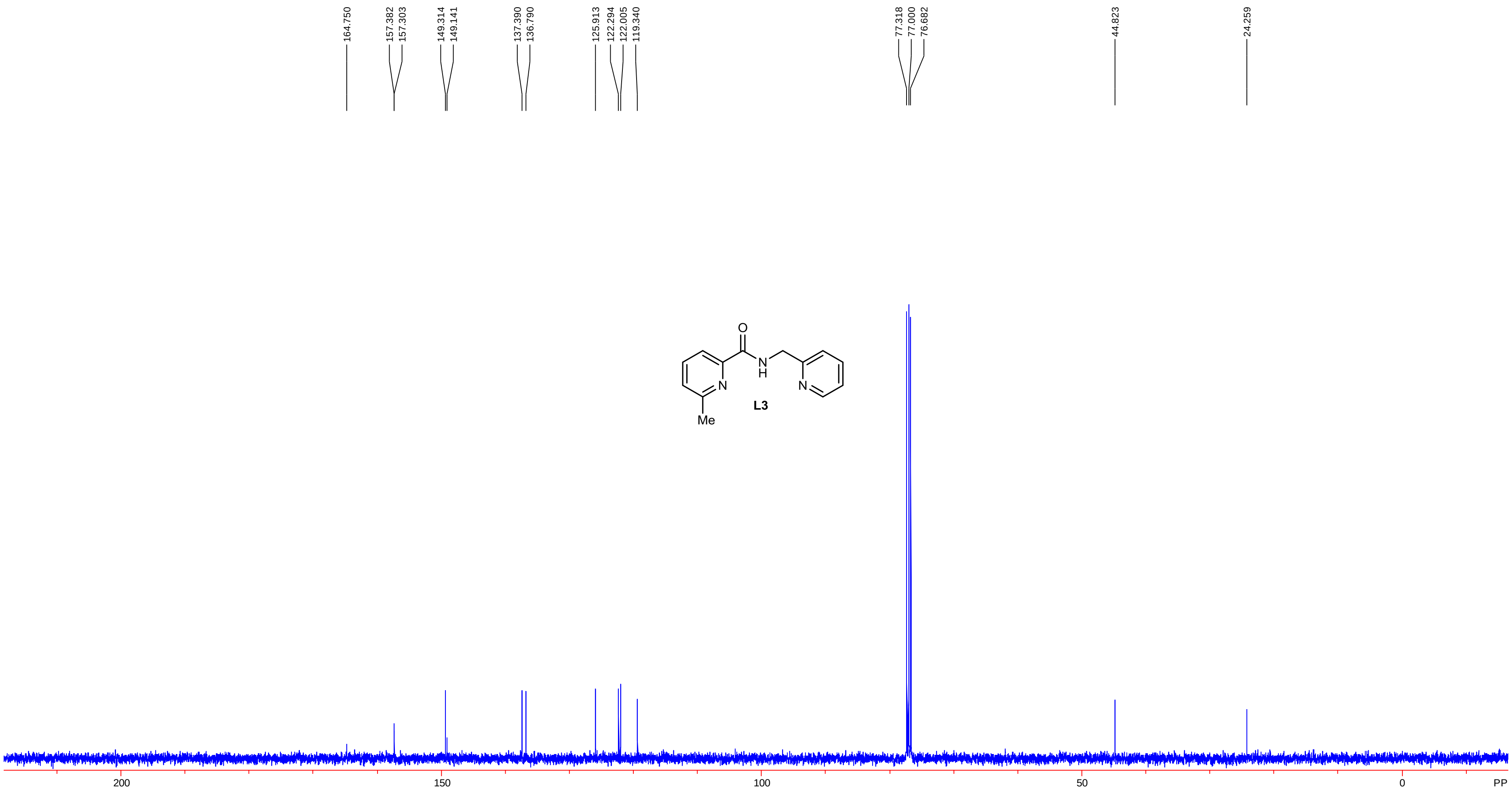

Supplementary Figure 13. ^{1}H NMR(600 MHz, $CDCl_3$)

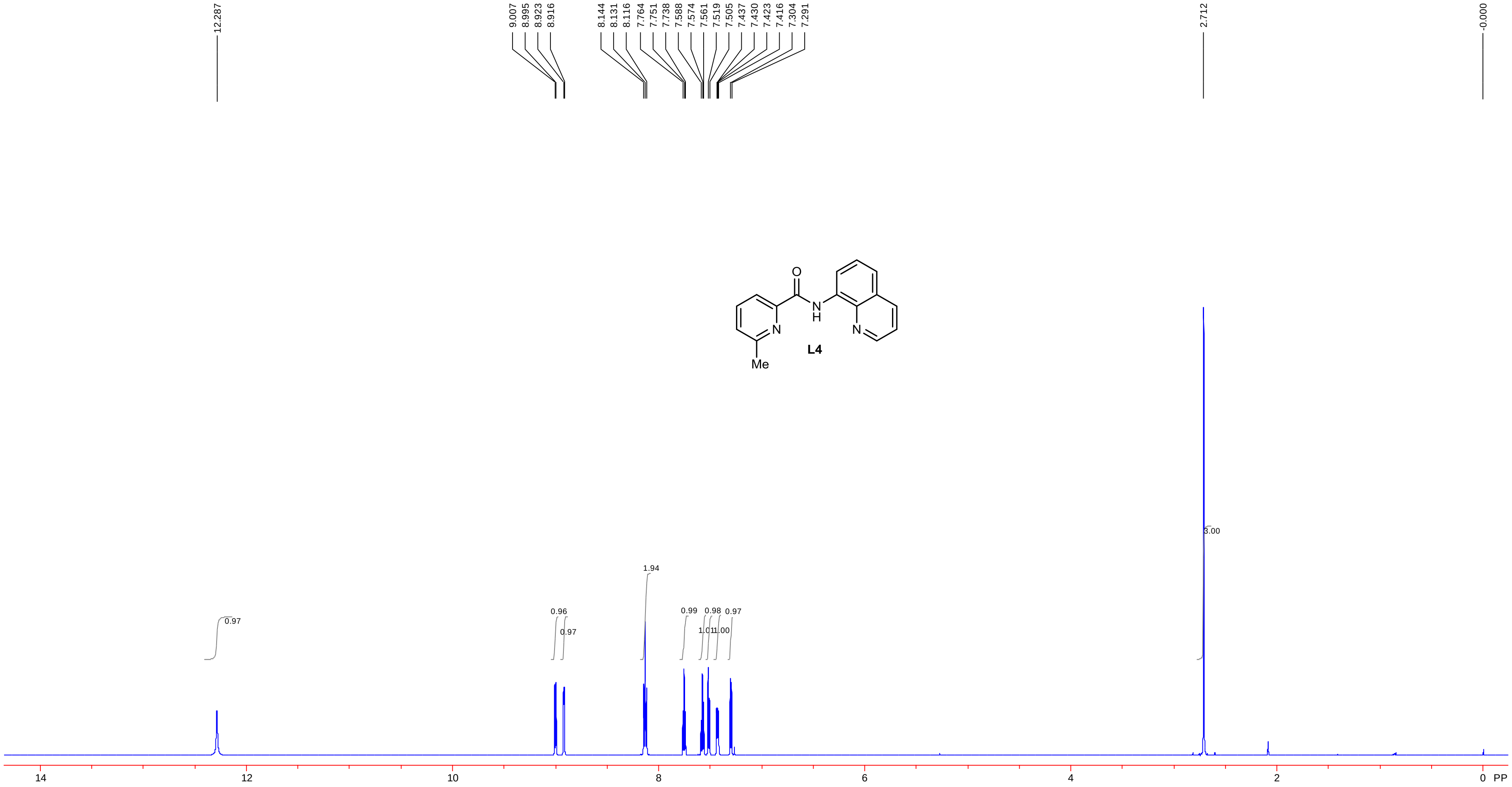

Supplementary Figure 14. ^{13}C NMR(151 MHz, $CDCl_3$)

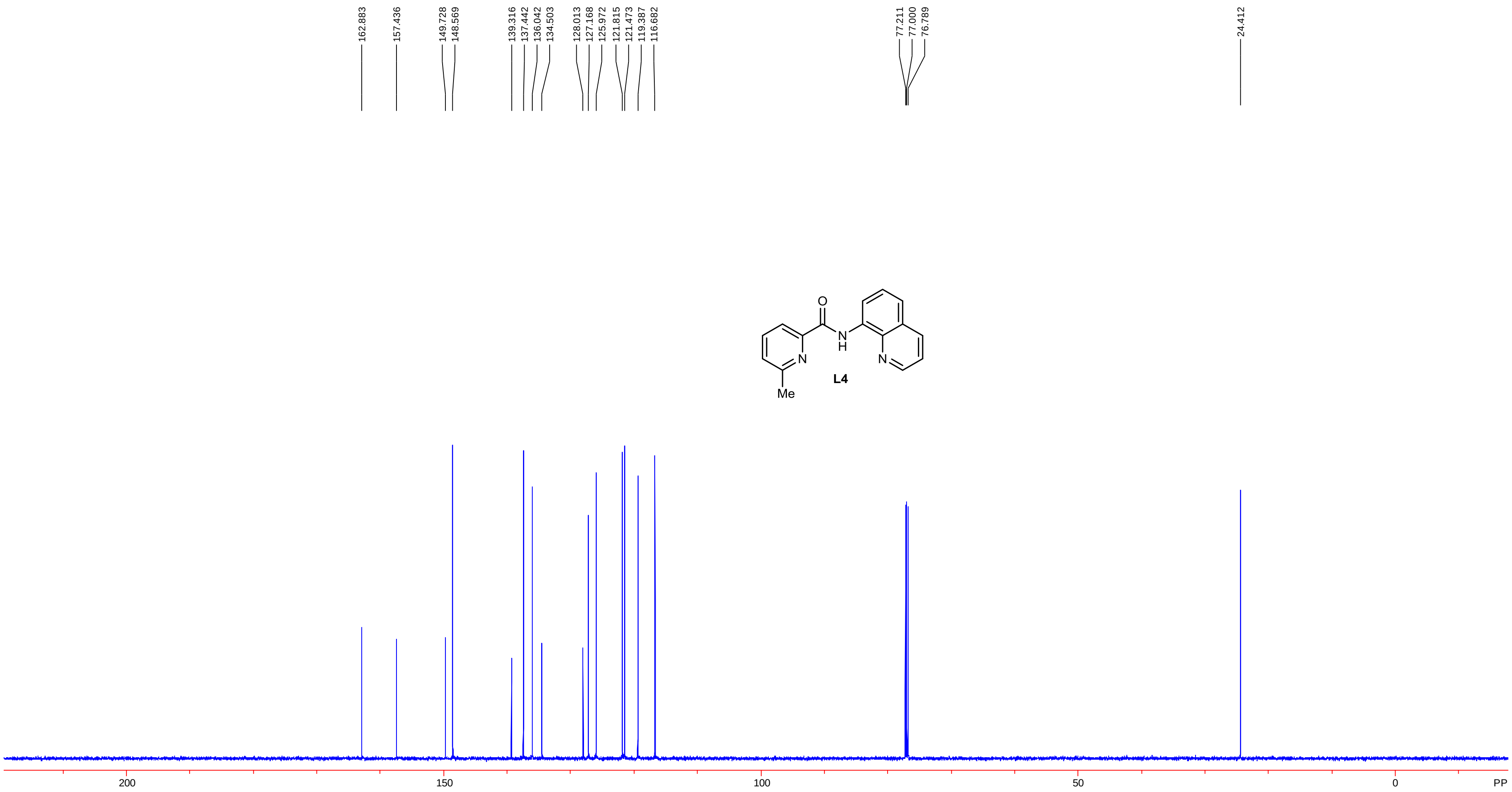

Supplementary Figure 15. ^{1}H NMR(400 MHz, $CDCl_3$)

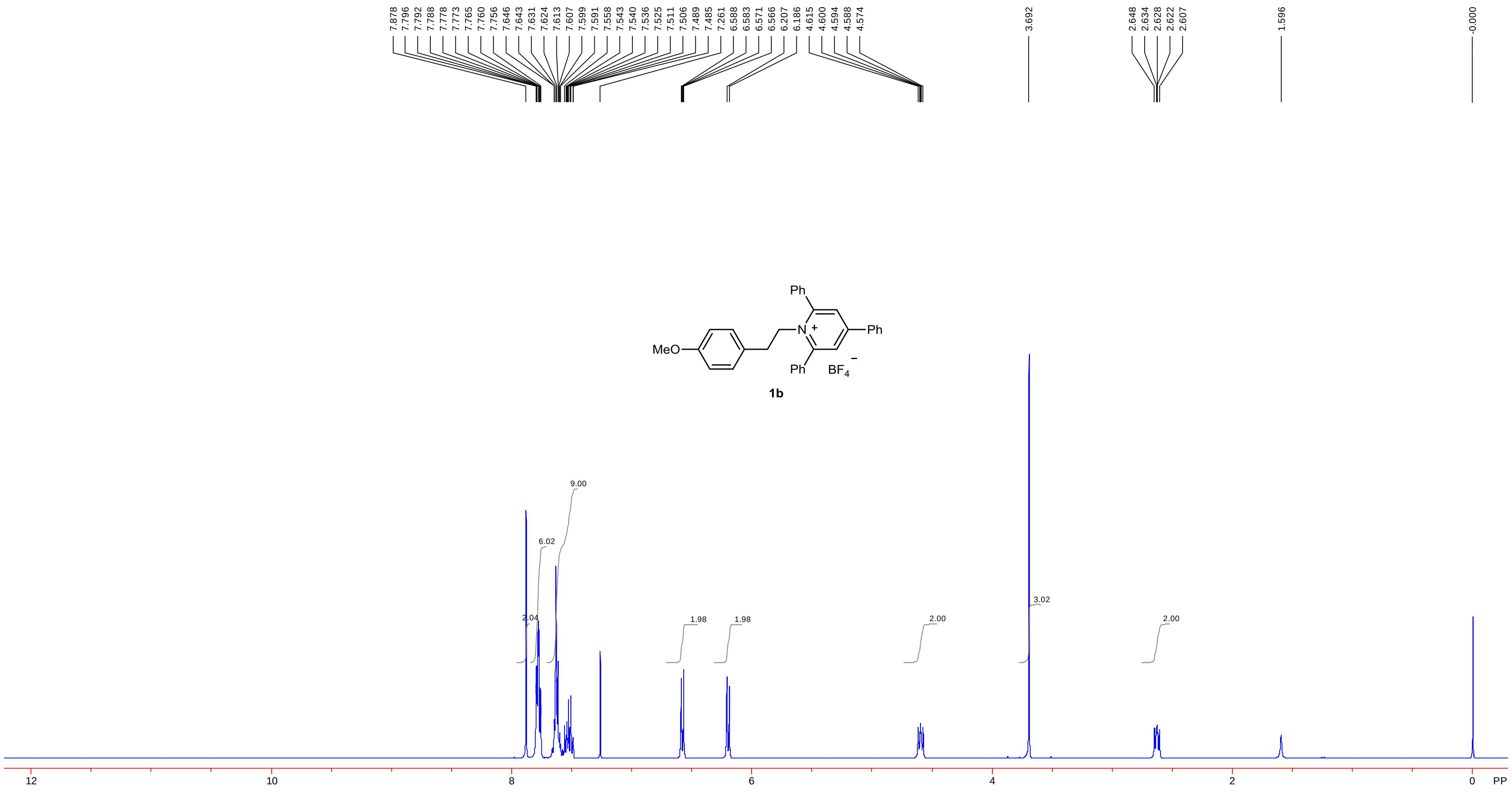

Supplementary Figure 16. ^{13}C NMR(100 MHz, $CDCl_3$)

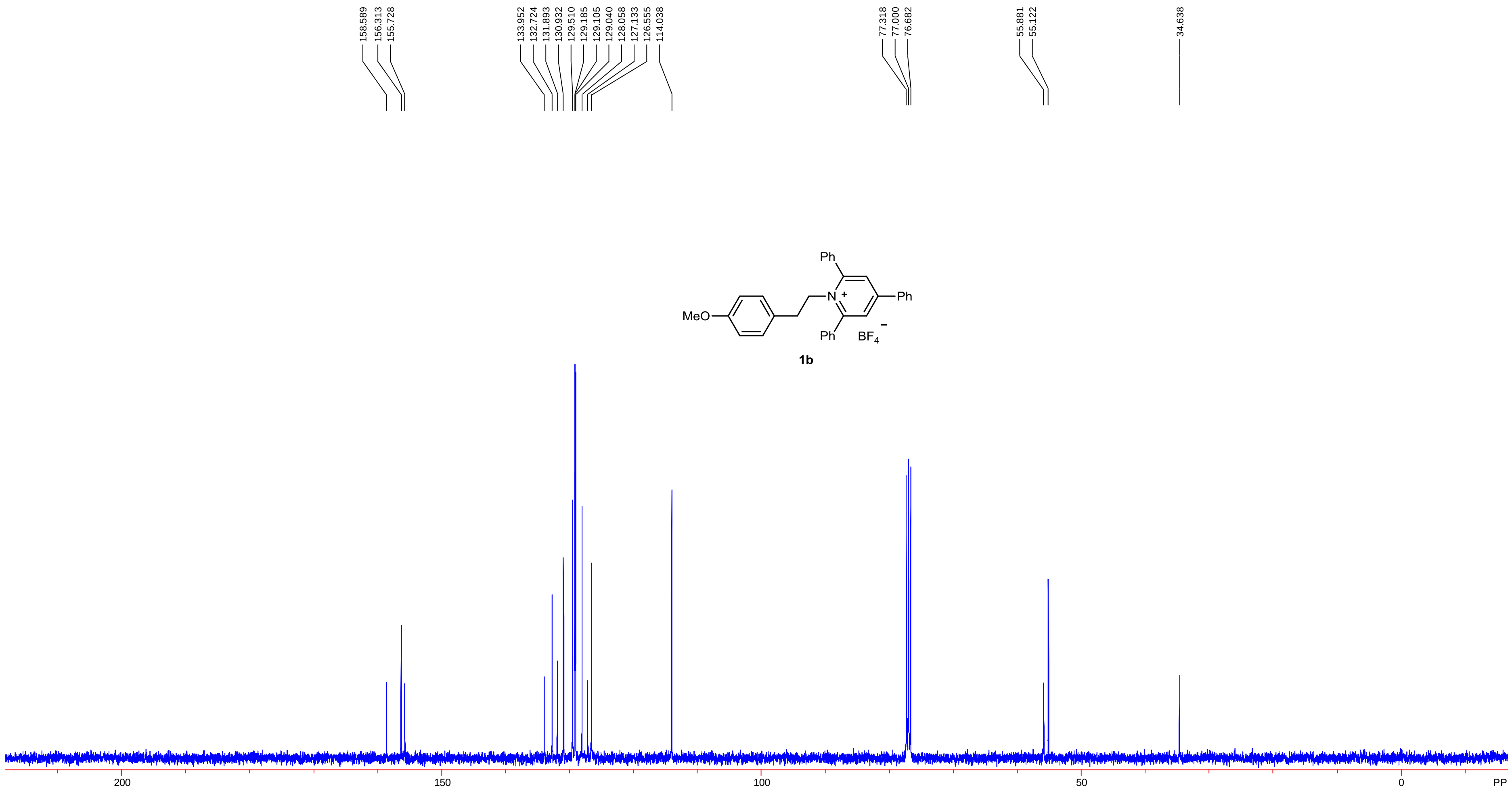

Supplementary Figure 17. ^{1}H NMR(400 MHz, $CDCl_3$)

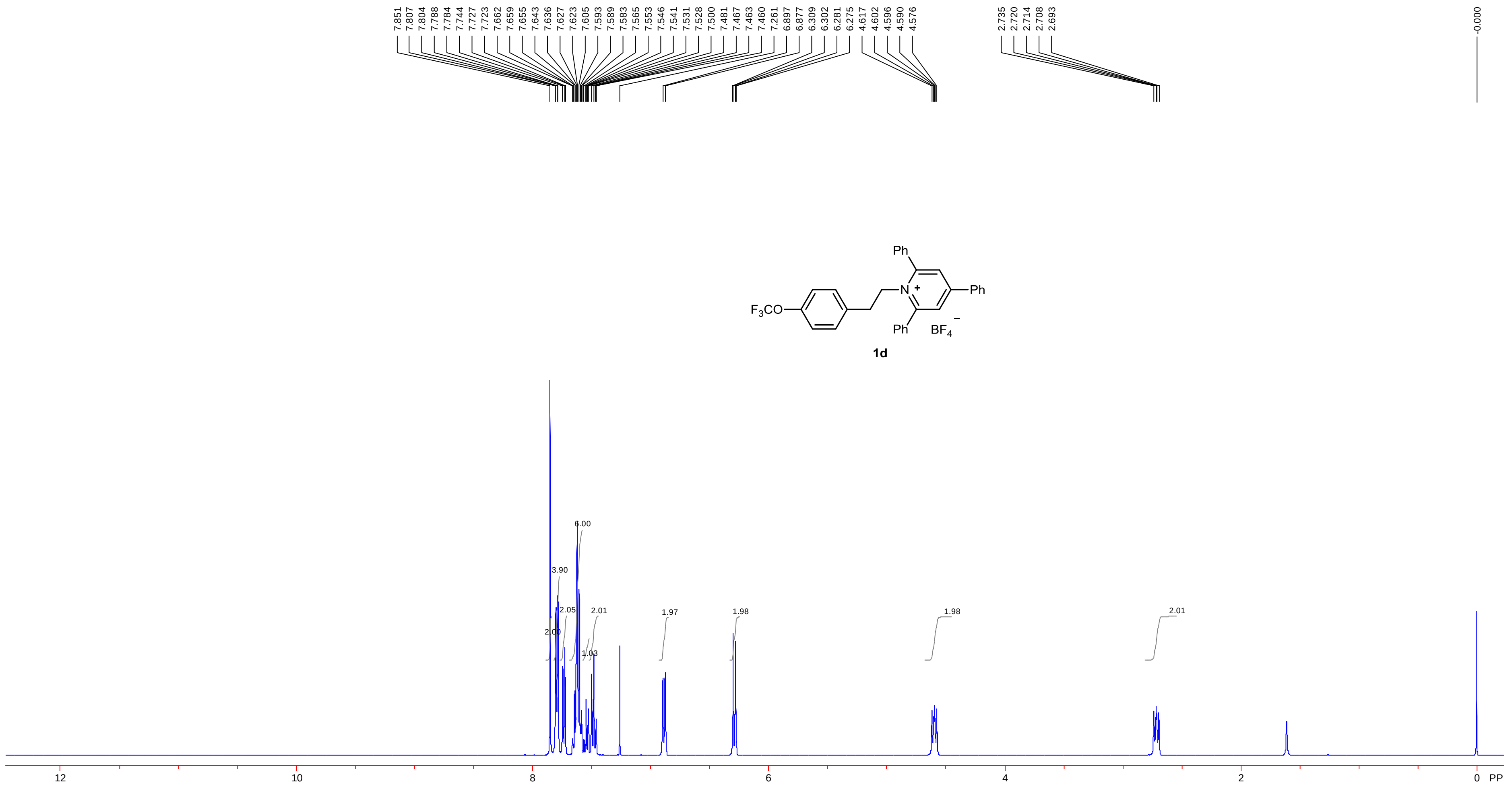

Supplementary Figure 18. ^{13}C NMR(100 MHz, $CDCl_3$)

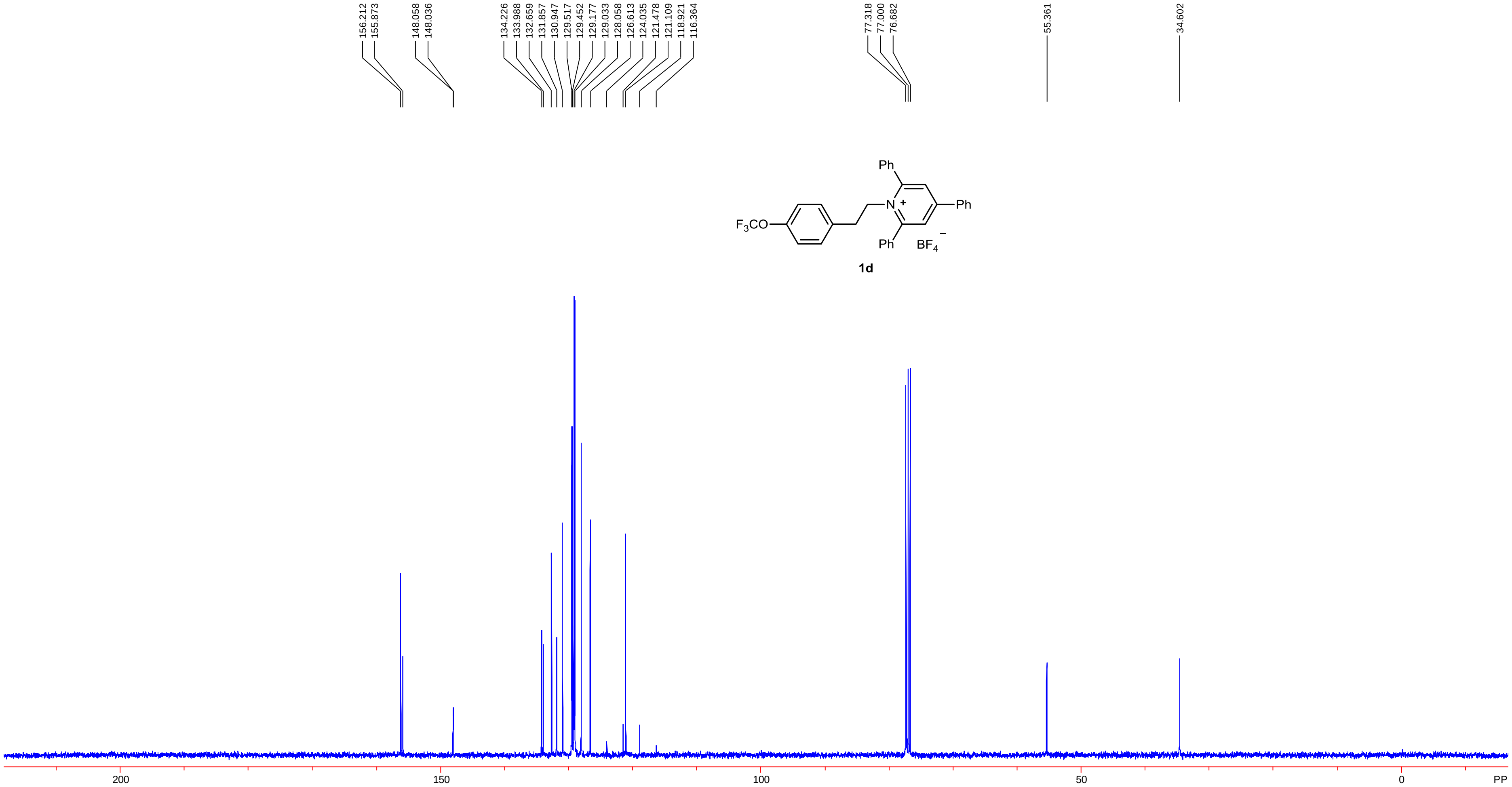

Supplementary Figure 19. ^{1}H NMR(600 MHz, $CDCl_3$)

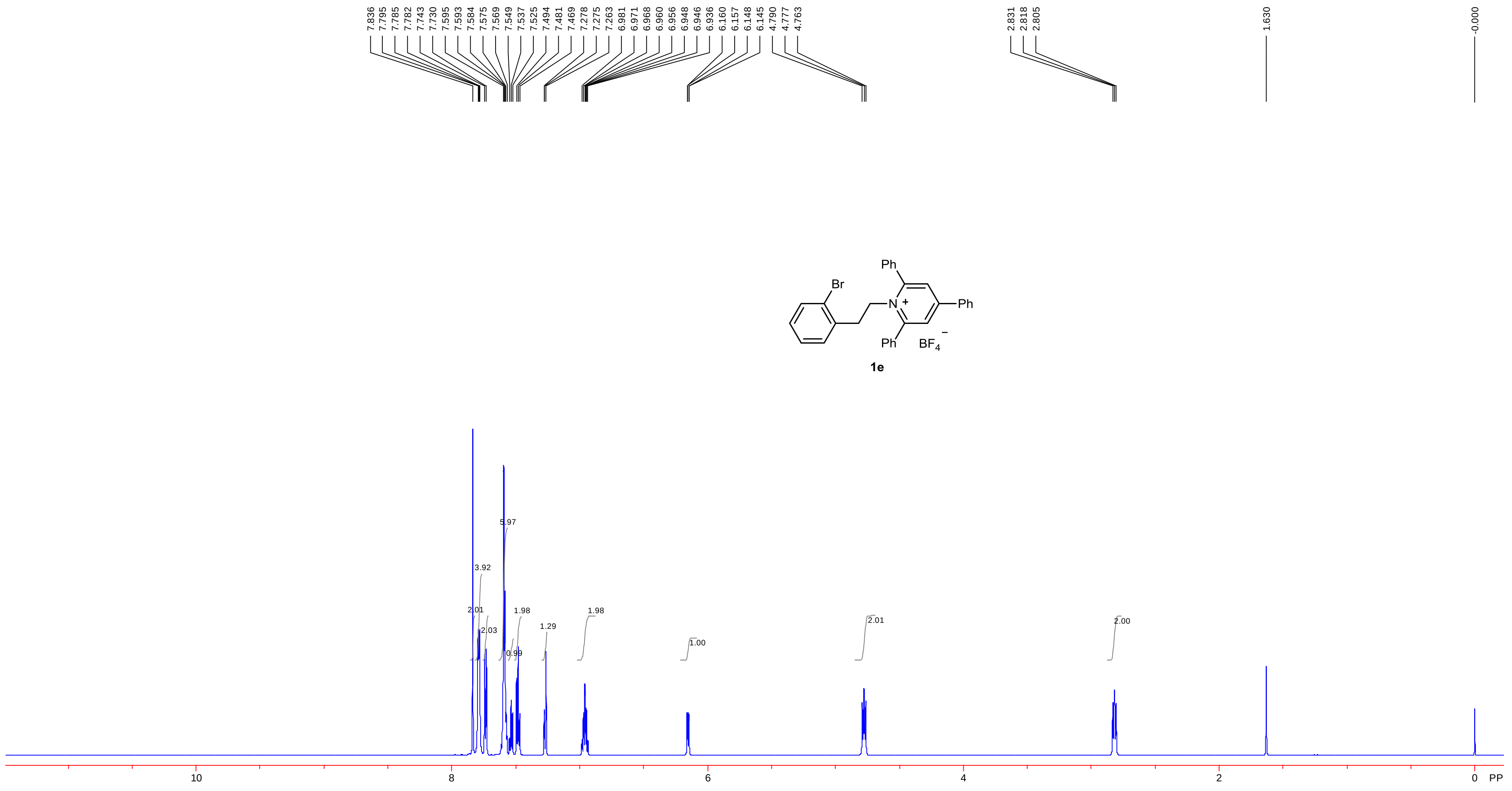

Supplementary Figure 20. ^{13}C NMR(151 MHz, $CDCl_3$)

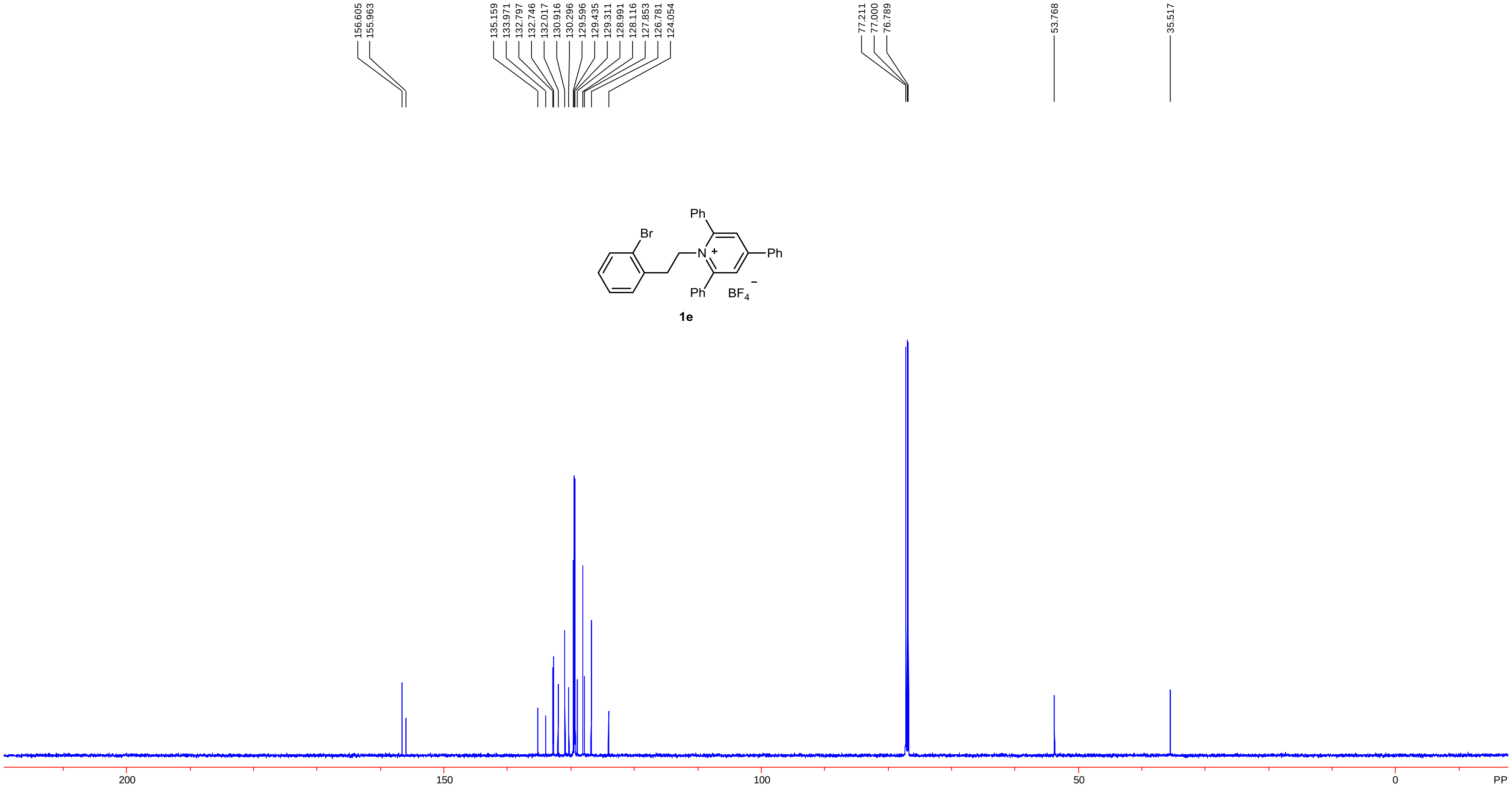

Supplementary Figure 21. ^{1}H NMR(400 MHz, $CDCl_3$)

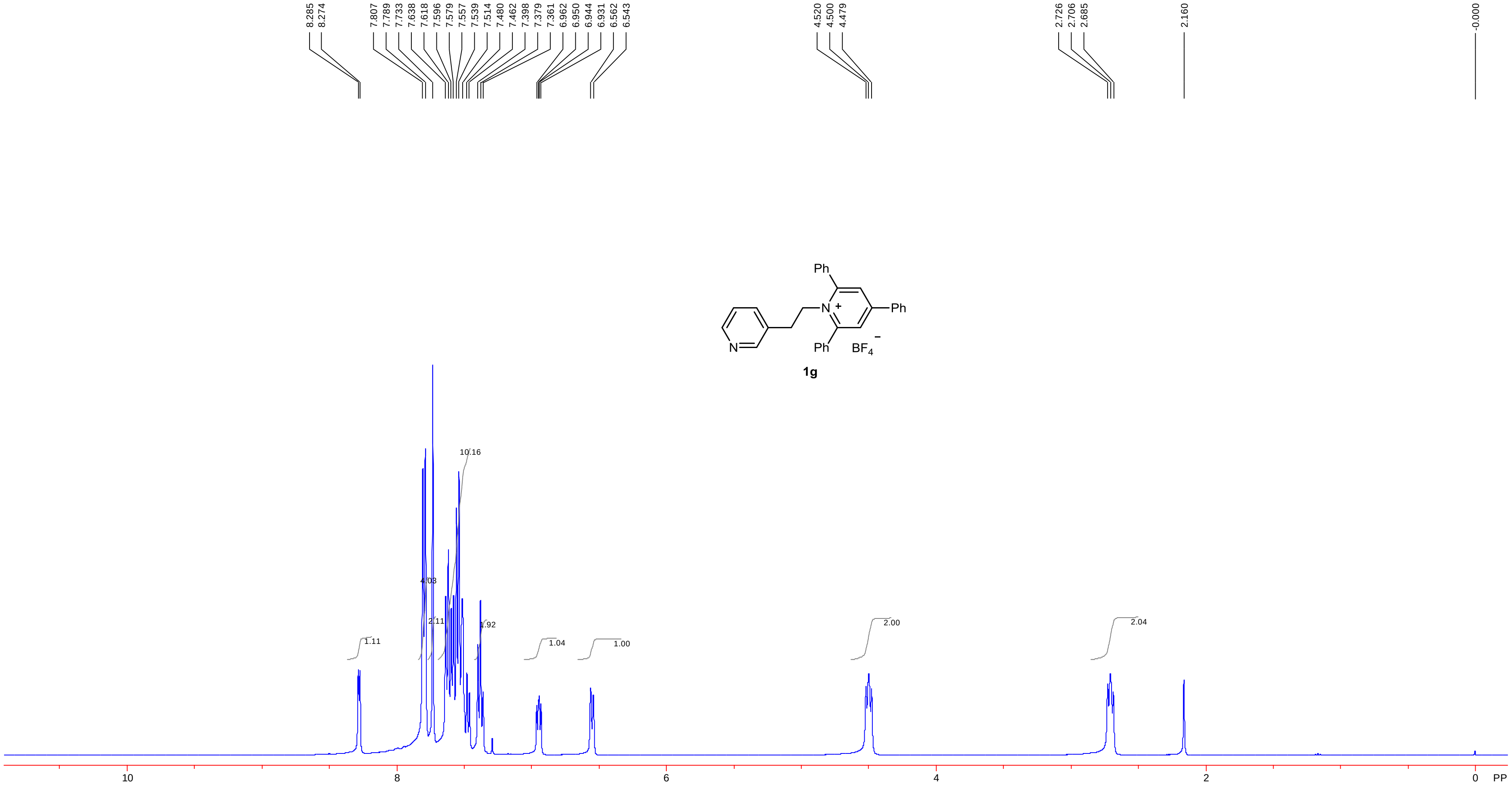

Supplementary Figure 22. ^{13}C NMR(100 MHz, $CDCl_3$)

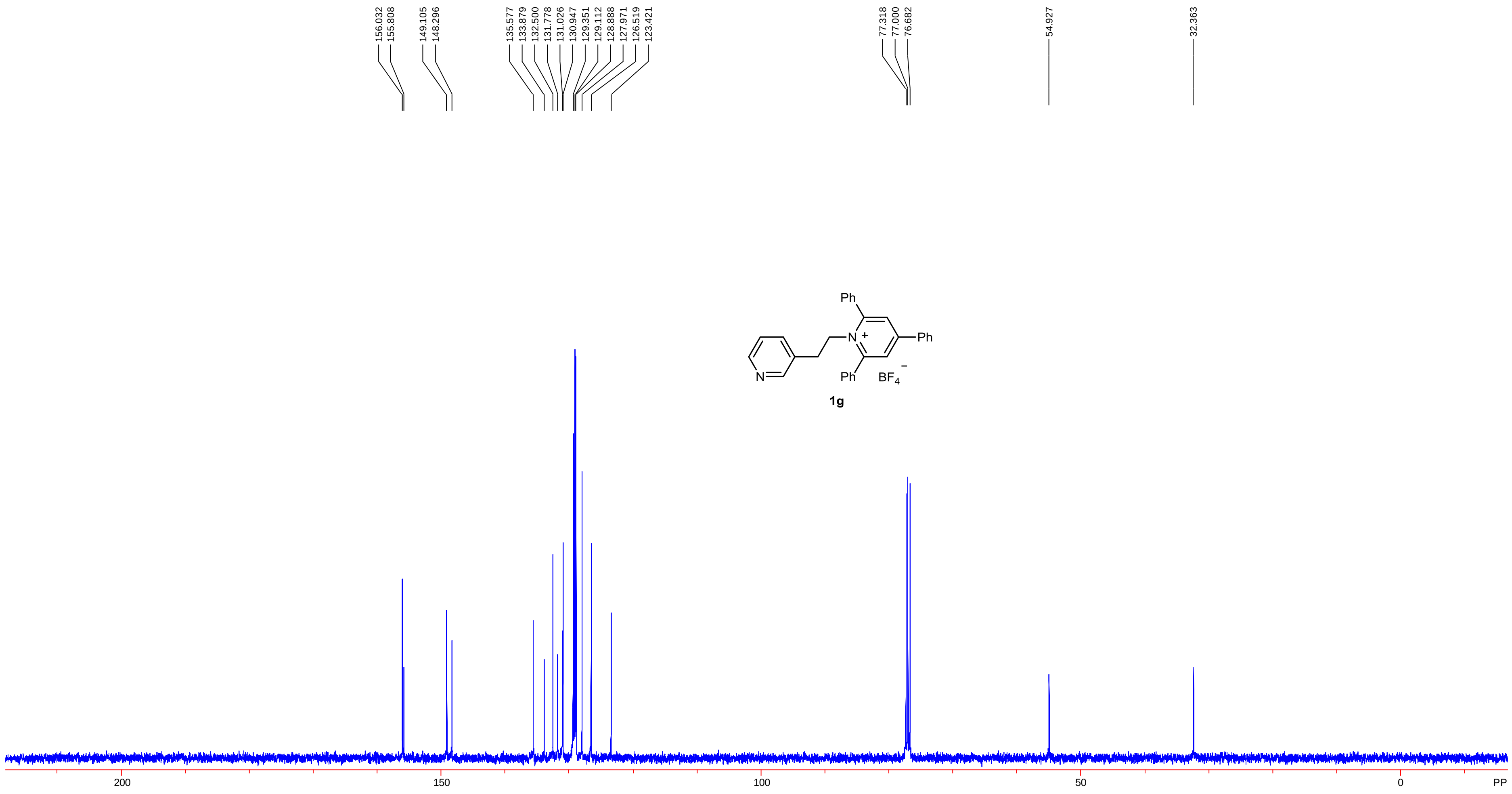

Supplementary Figure 23. ^{1}H NMR (600 MHz, DMSO-d_6)

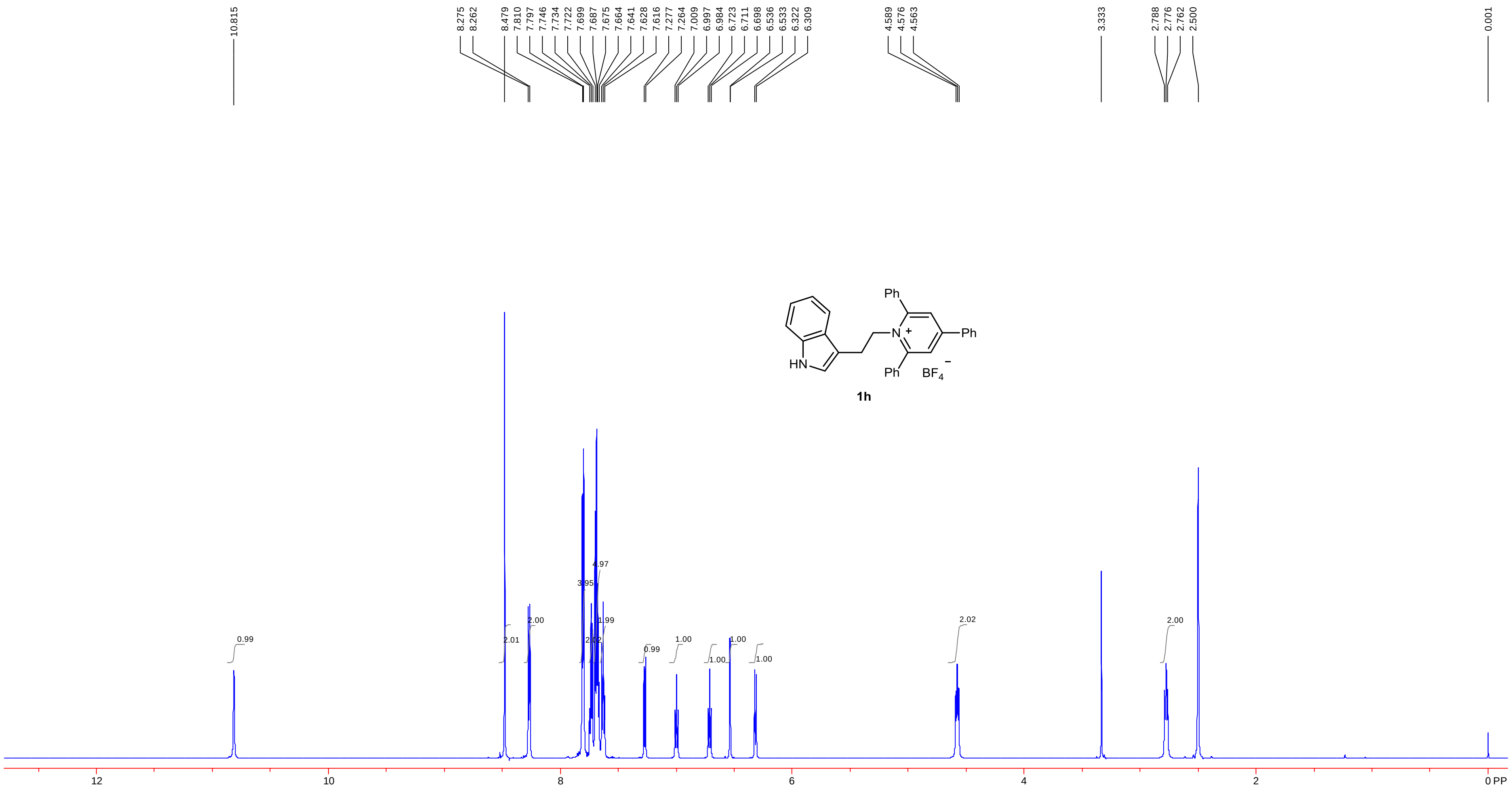

Supplementary Figure 24. ^{13}C NMR (151 MHz, DMSO-d_6)

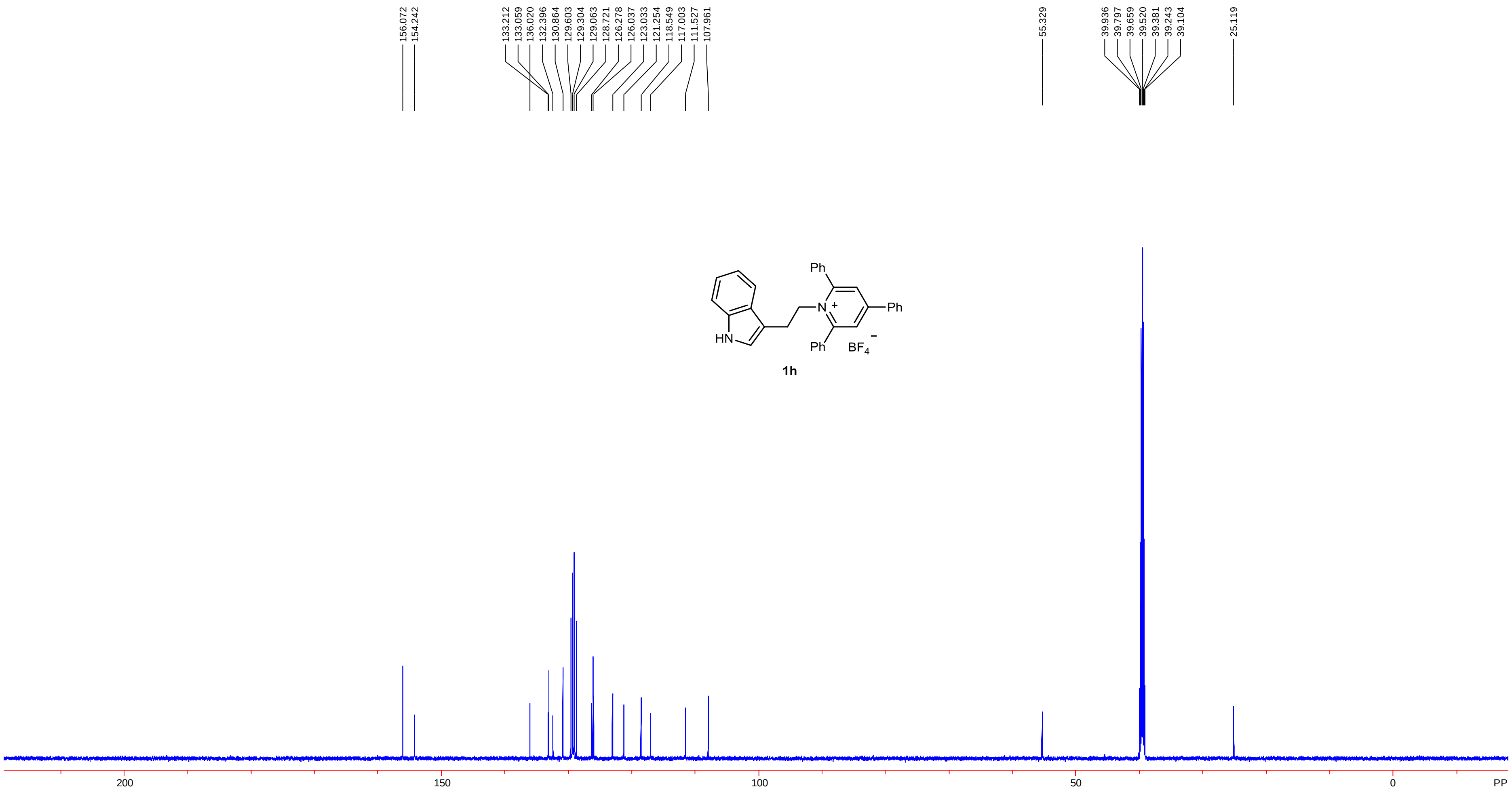

Supplementary Figure 25. ^{1}H NMR (600 MHz, $CDCl_3$)

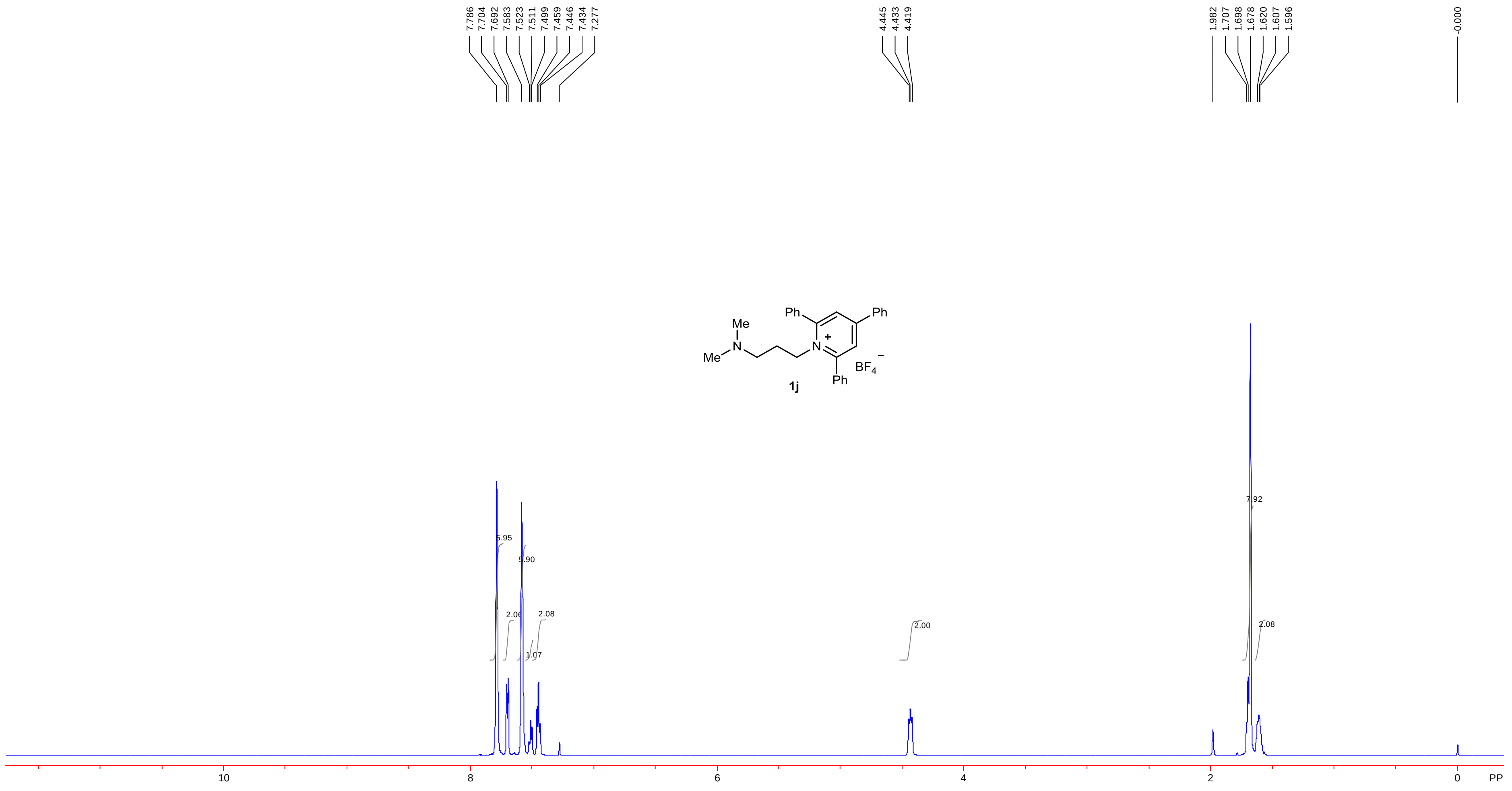

Supplementary Figure 26. ^{13}C NMR (151 MHz, $CDCl_3$)

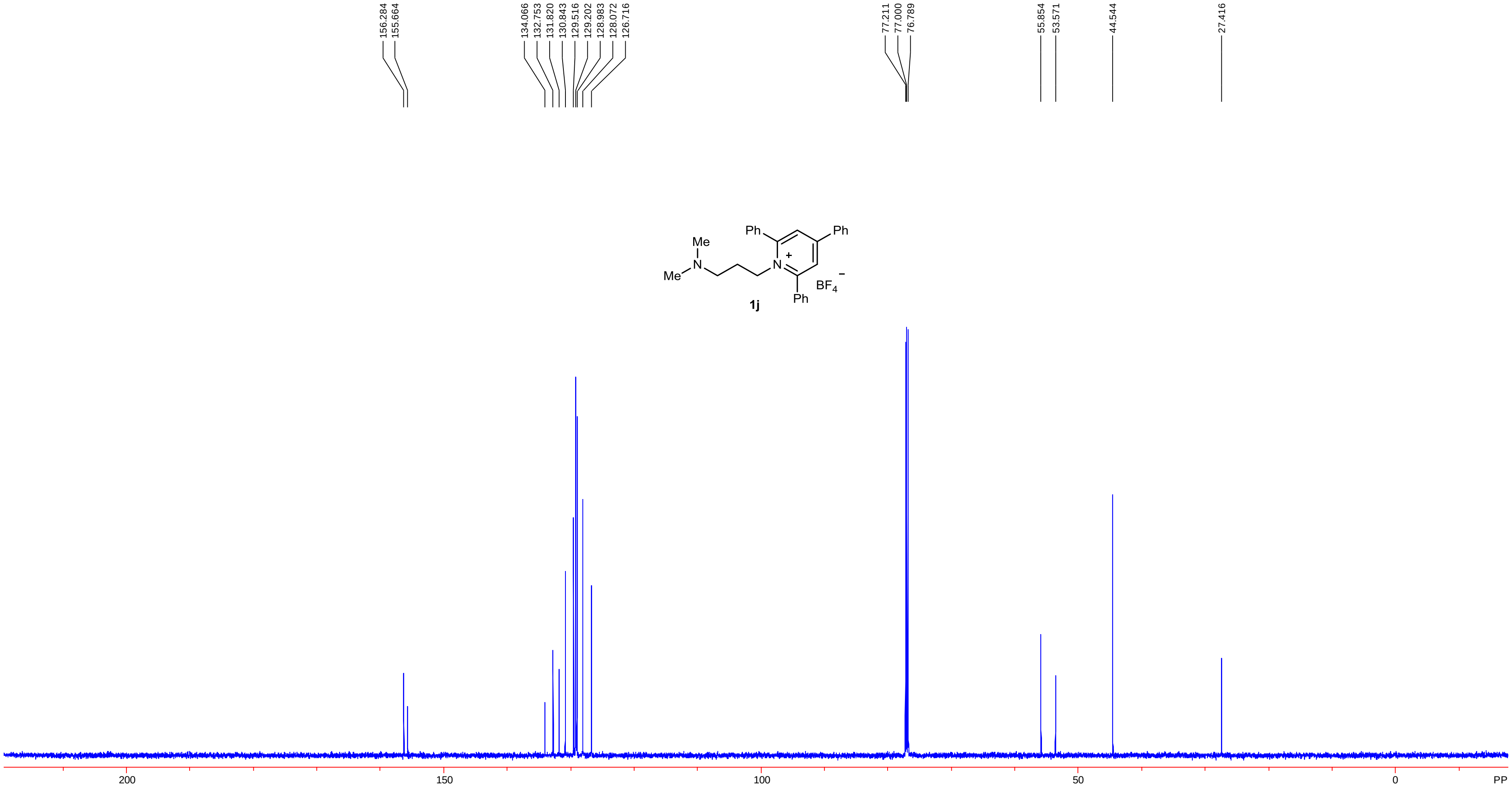

Supplementary Figure 27. ^{1}H NMR(400 MHz, $CDCl_3$)

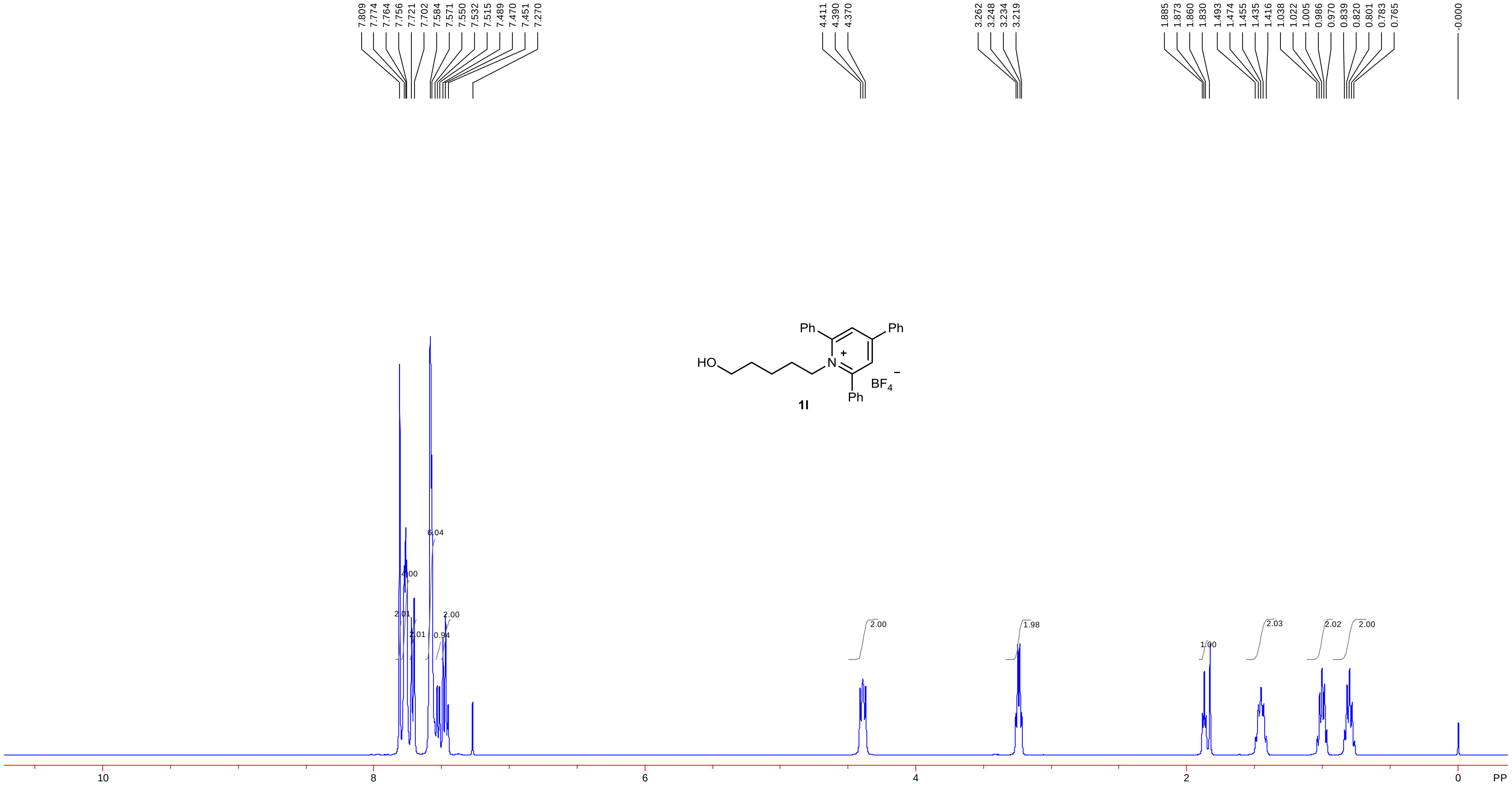

Supplementary Figure 28. ^{13}C NMR(100 MHz, $CDCl_3$)

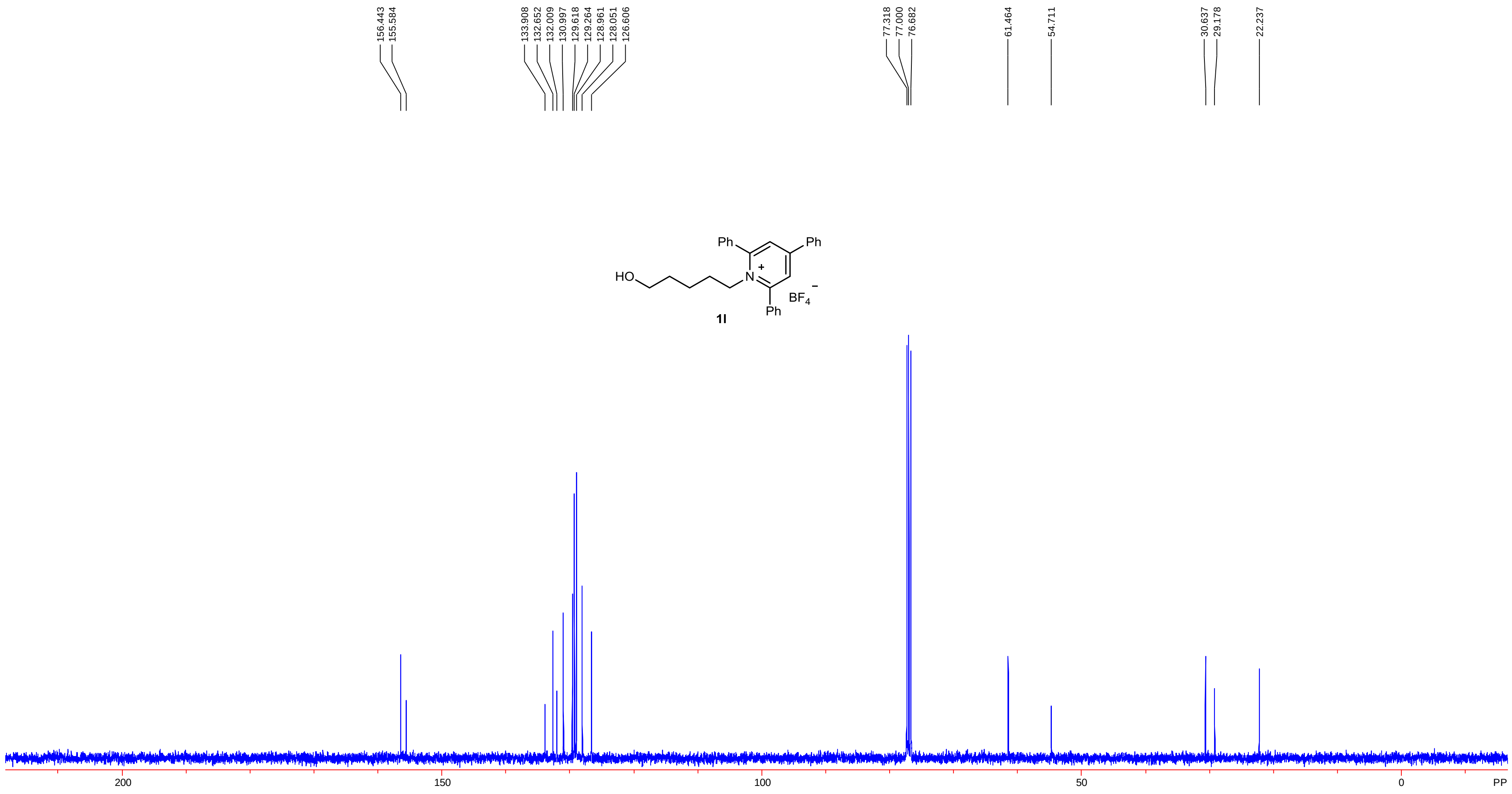

Supplementary Figure 29. ^{1}H NMR(400 MHz, $CDCl_3$)

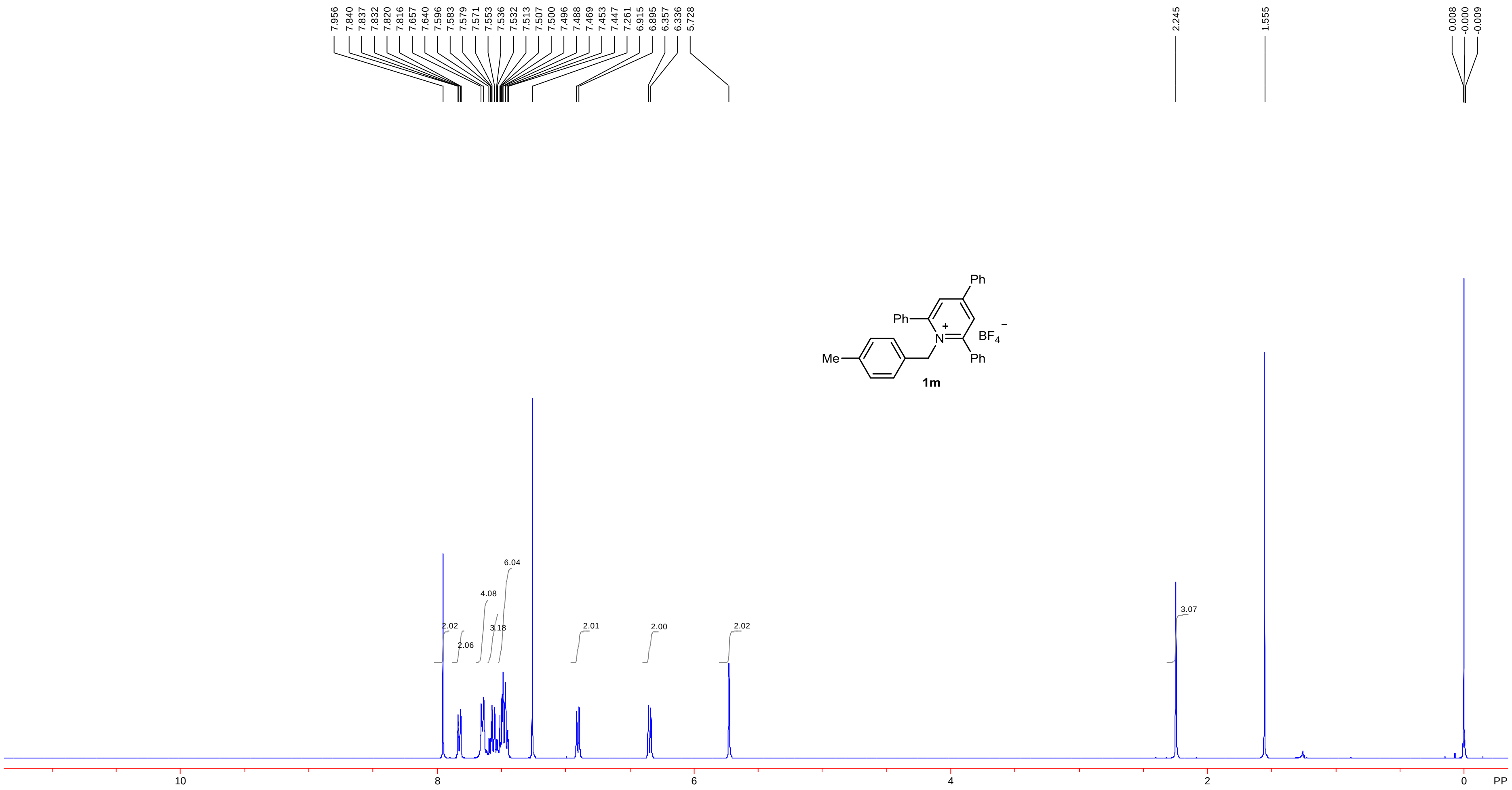

Supplementary Figure 30. ^{13}C NMR(100 MHz, $CDCl_3$)

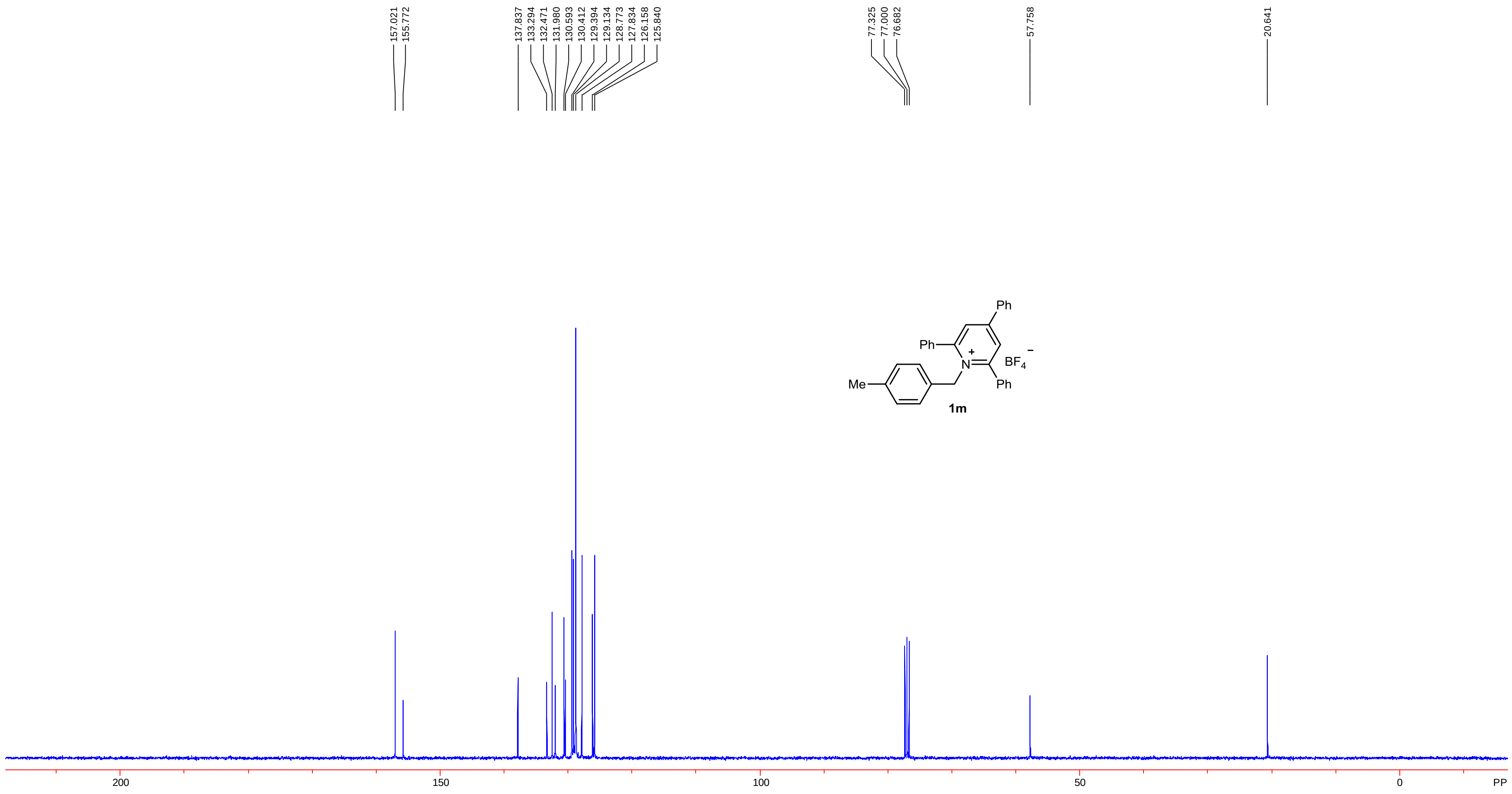

Supplementary Figure 31. ^{1}H NMR(400 MHz, $CDCl_3$)

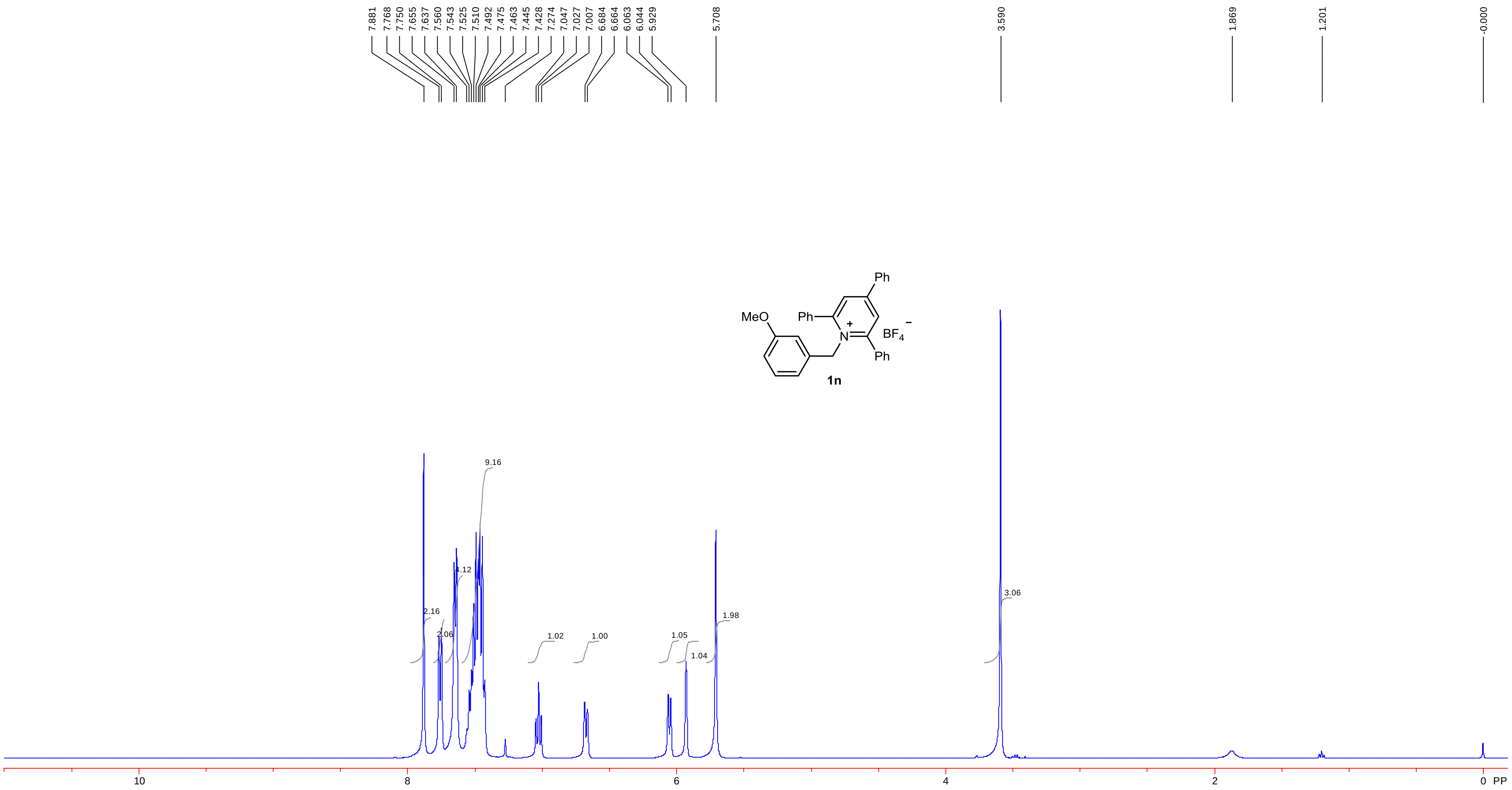

Supplementary Figure 32. ^{13}C NMR(100 MHz, $CDCl_3$)

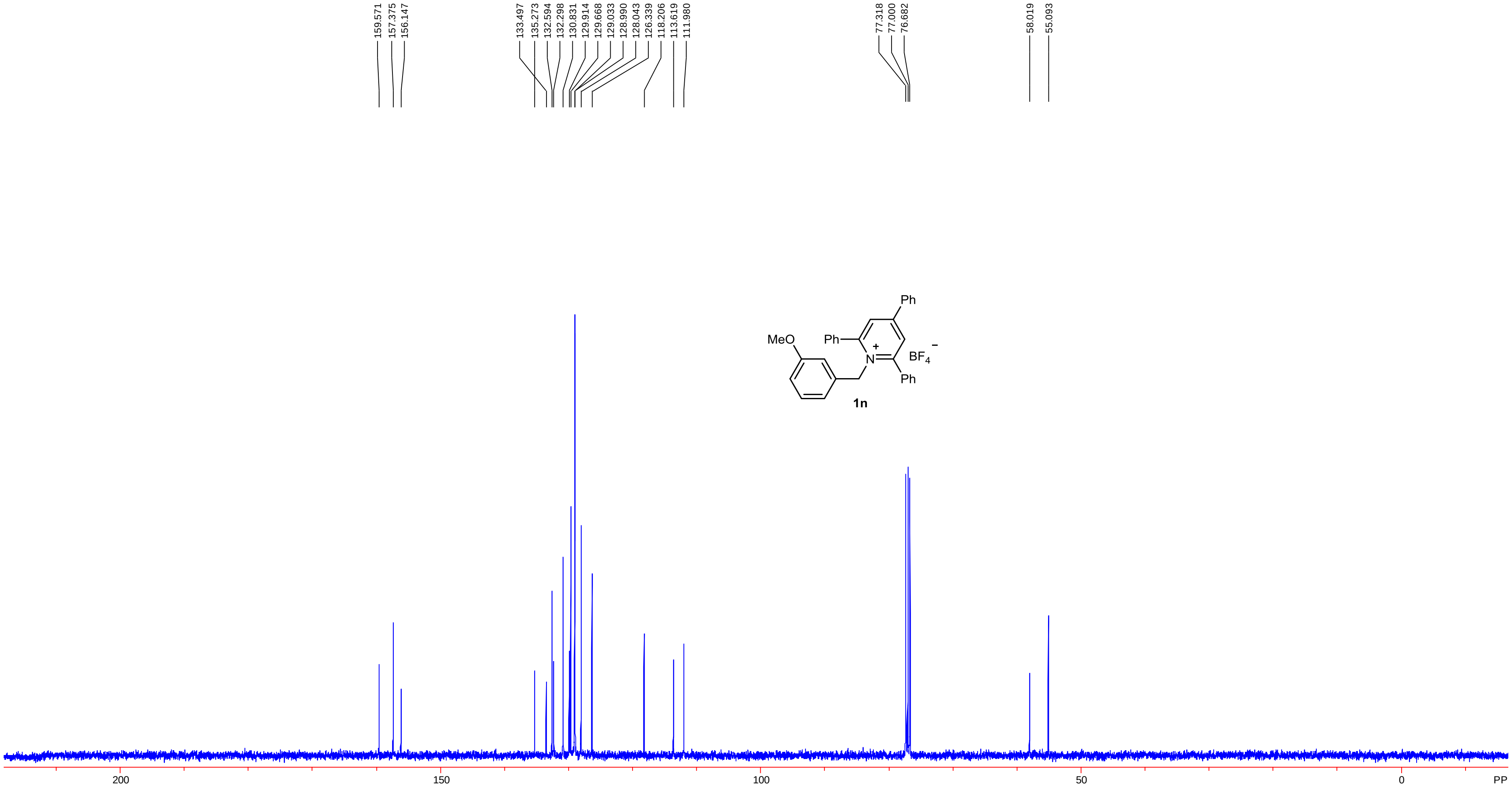

Supplementary Figure 33. ^{1}H NMR(400 MHz, $CDCl_3$)

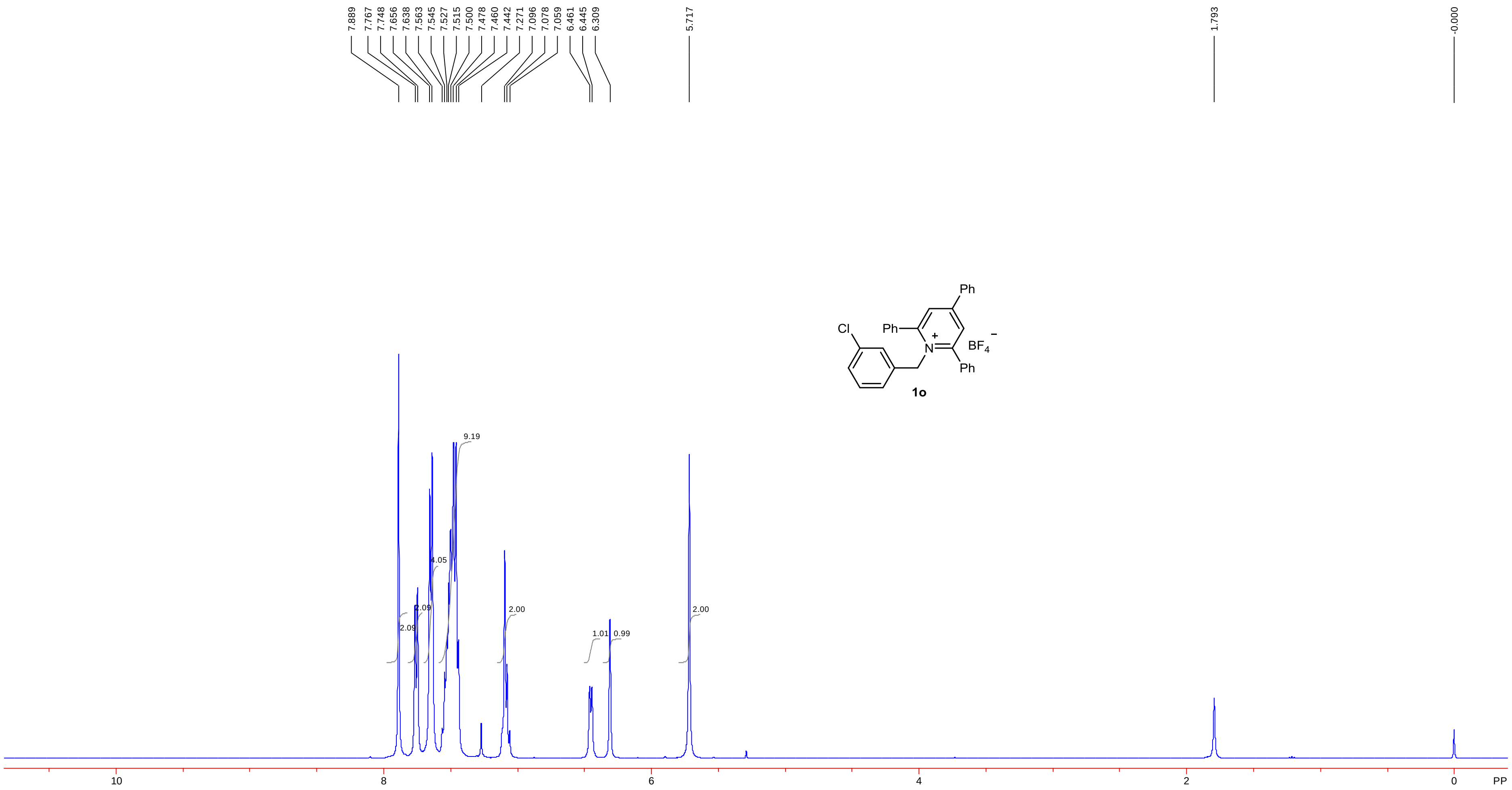

Supplementary Figure 34. ^{13}C NMR(100 MHz, $CDCl_3$)

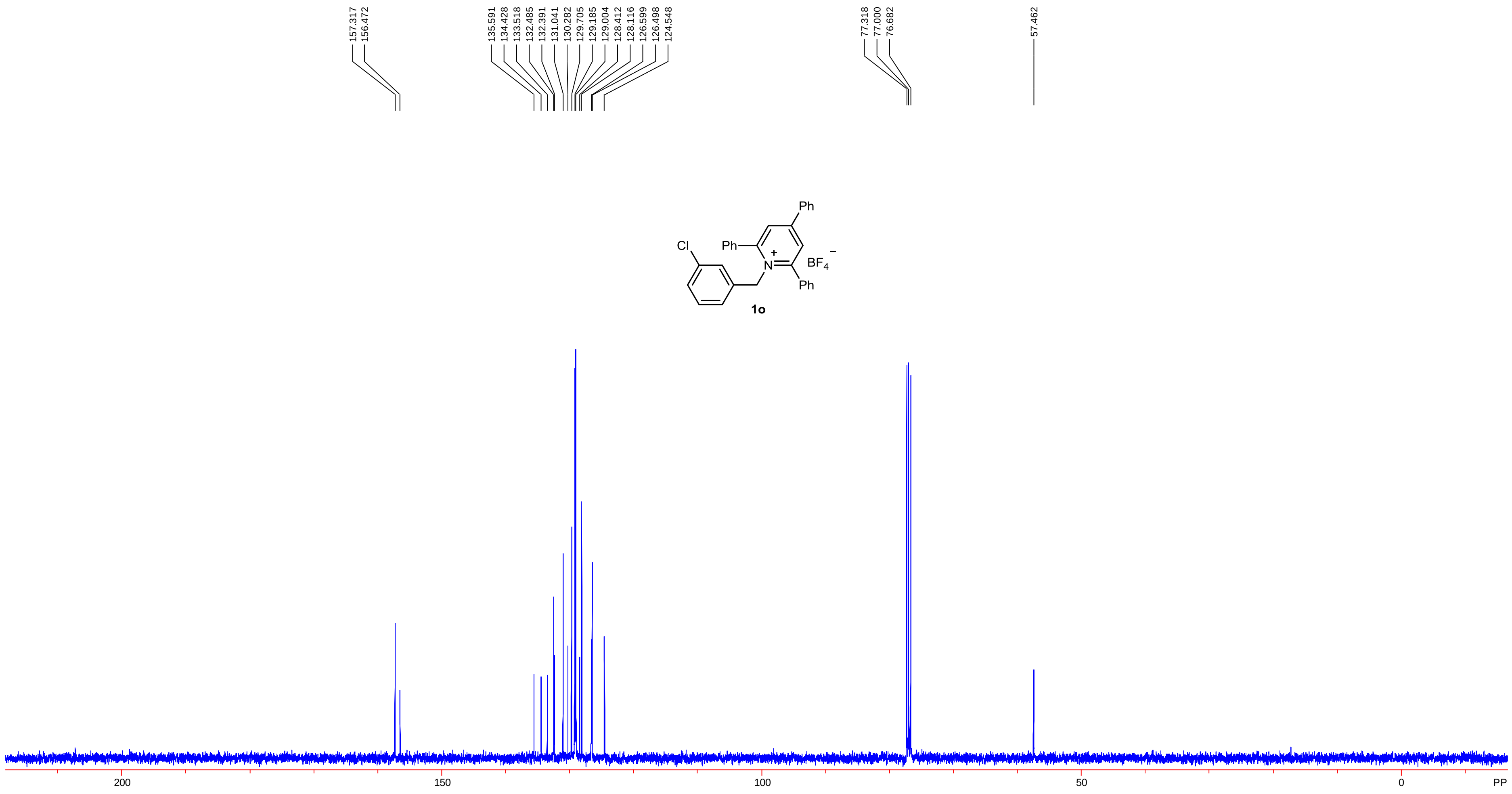

Supplementary Figure 35. ^{1}H NMR (600 MHz, $CDCl_3$)

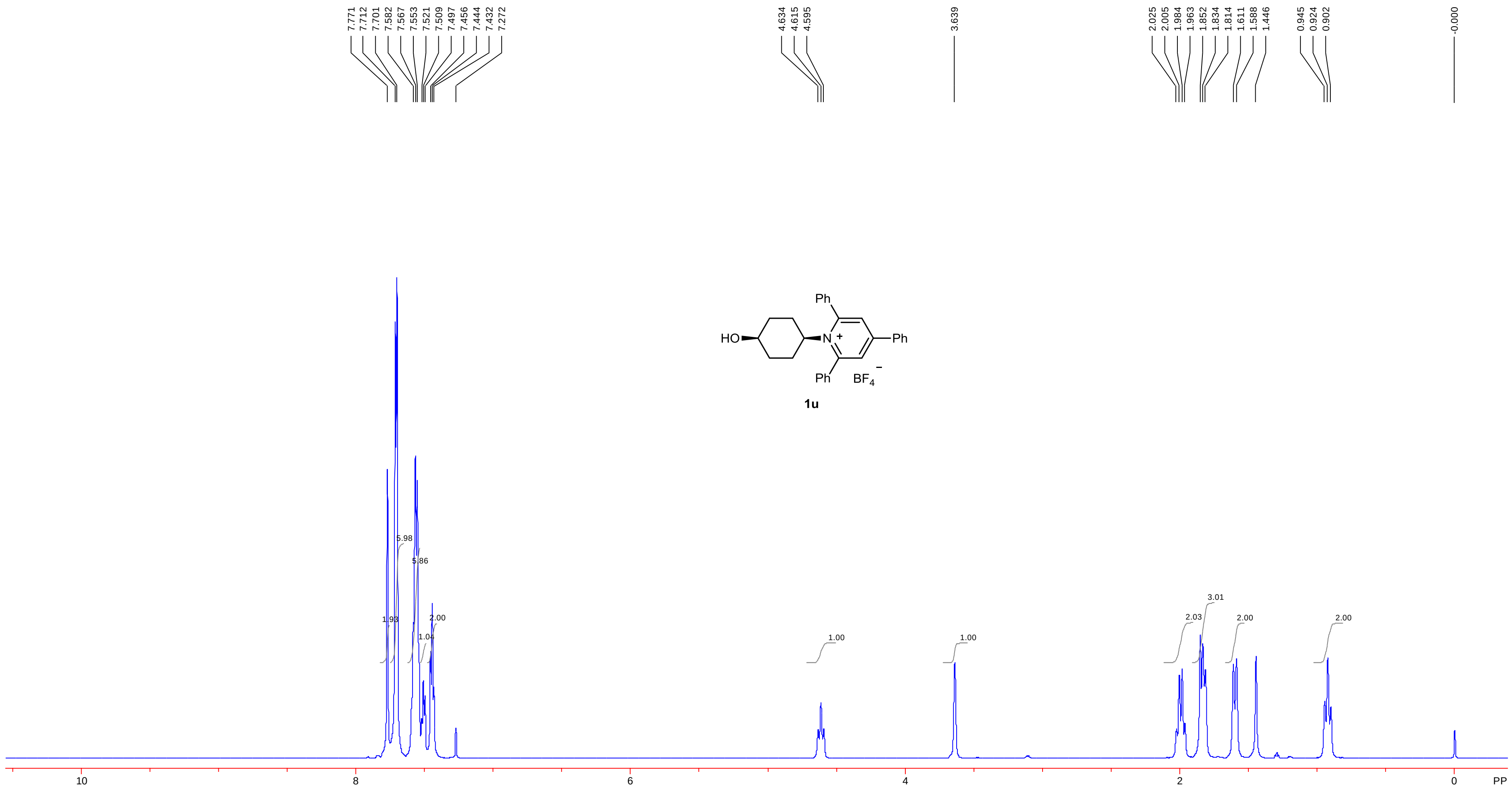

Supplementary Figure 36. ^{13}C NMR (151 MHz, $CDCl_3$)

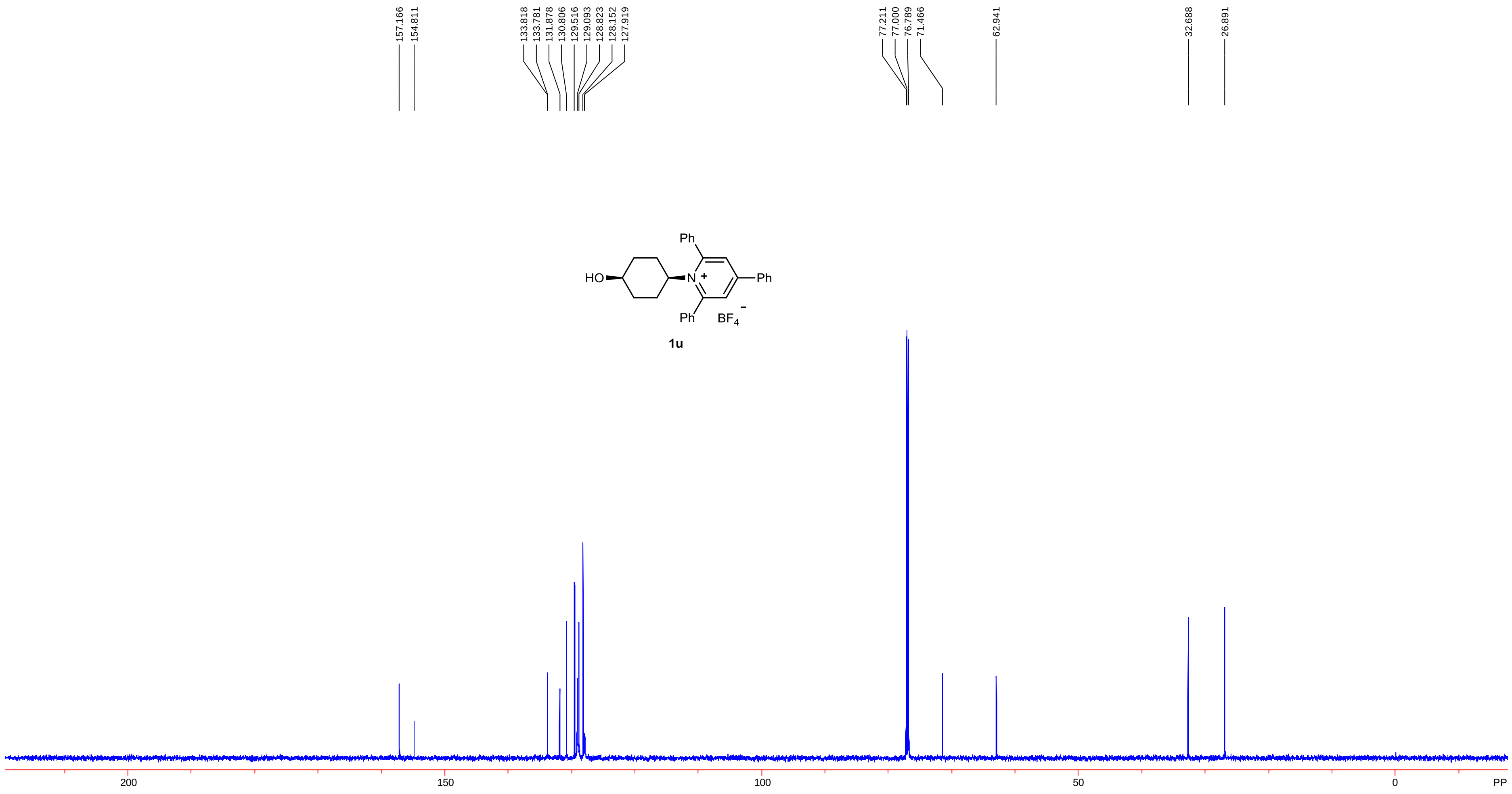

Supplementary Figure 37. ^{1}H NMR(400 MHz, $CDCl_3$)

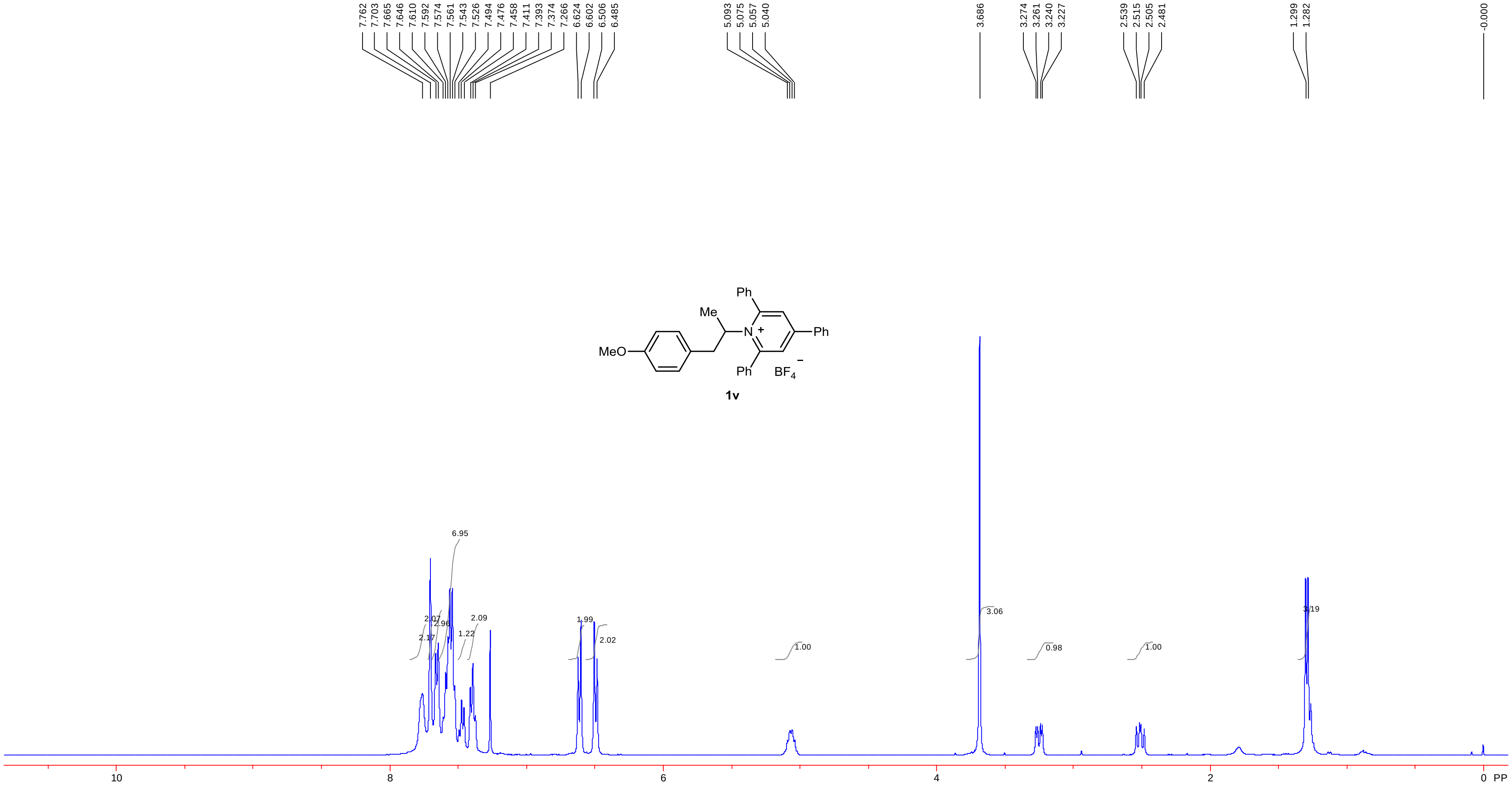

Supplementary Figure 38. ^{13}C NMR(100 MHz, $CDCl_3$)

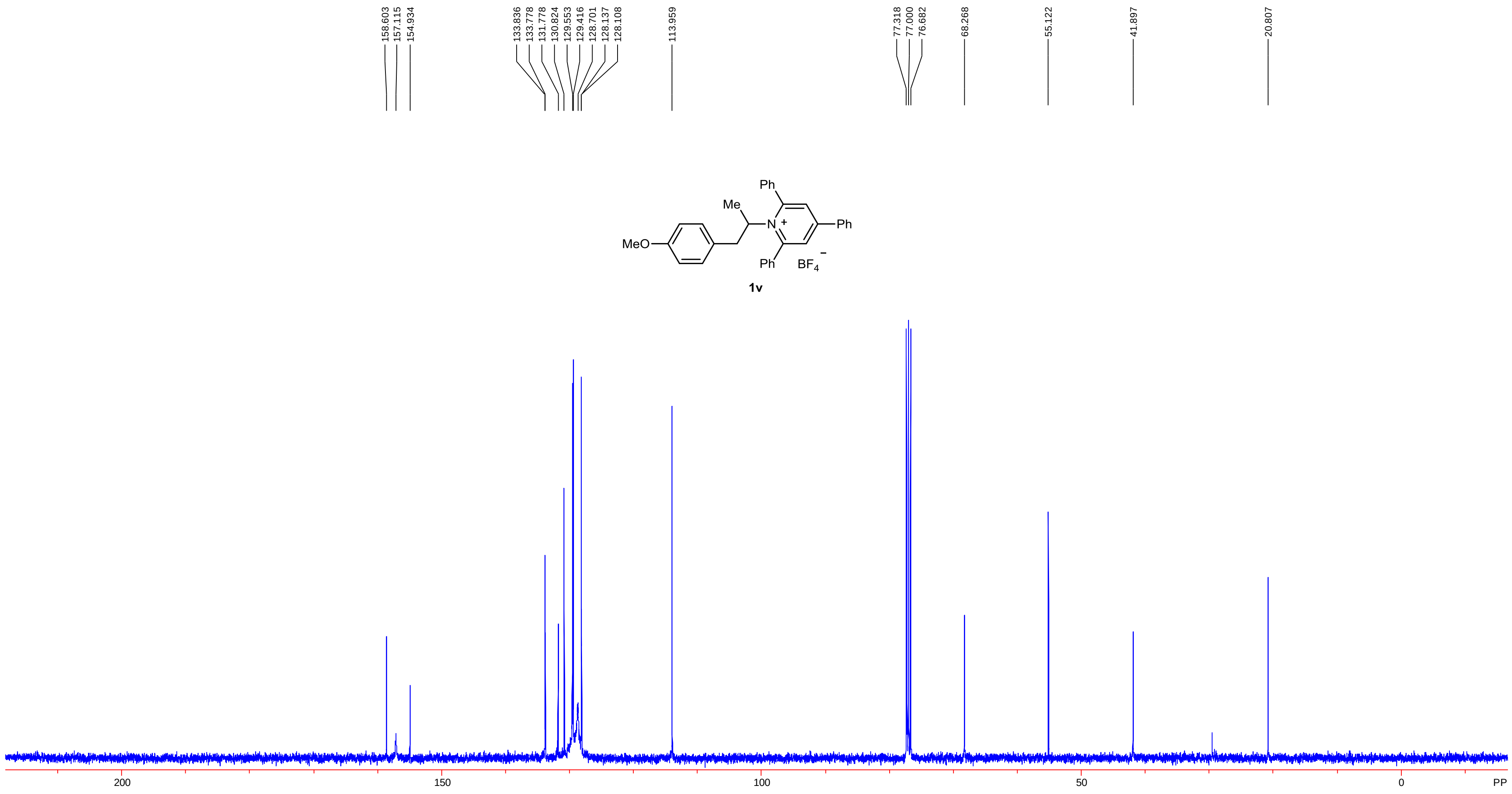

Supplementary Figure 39. ^{1}H NMR (600 MHz, $CDCl_3$)

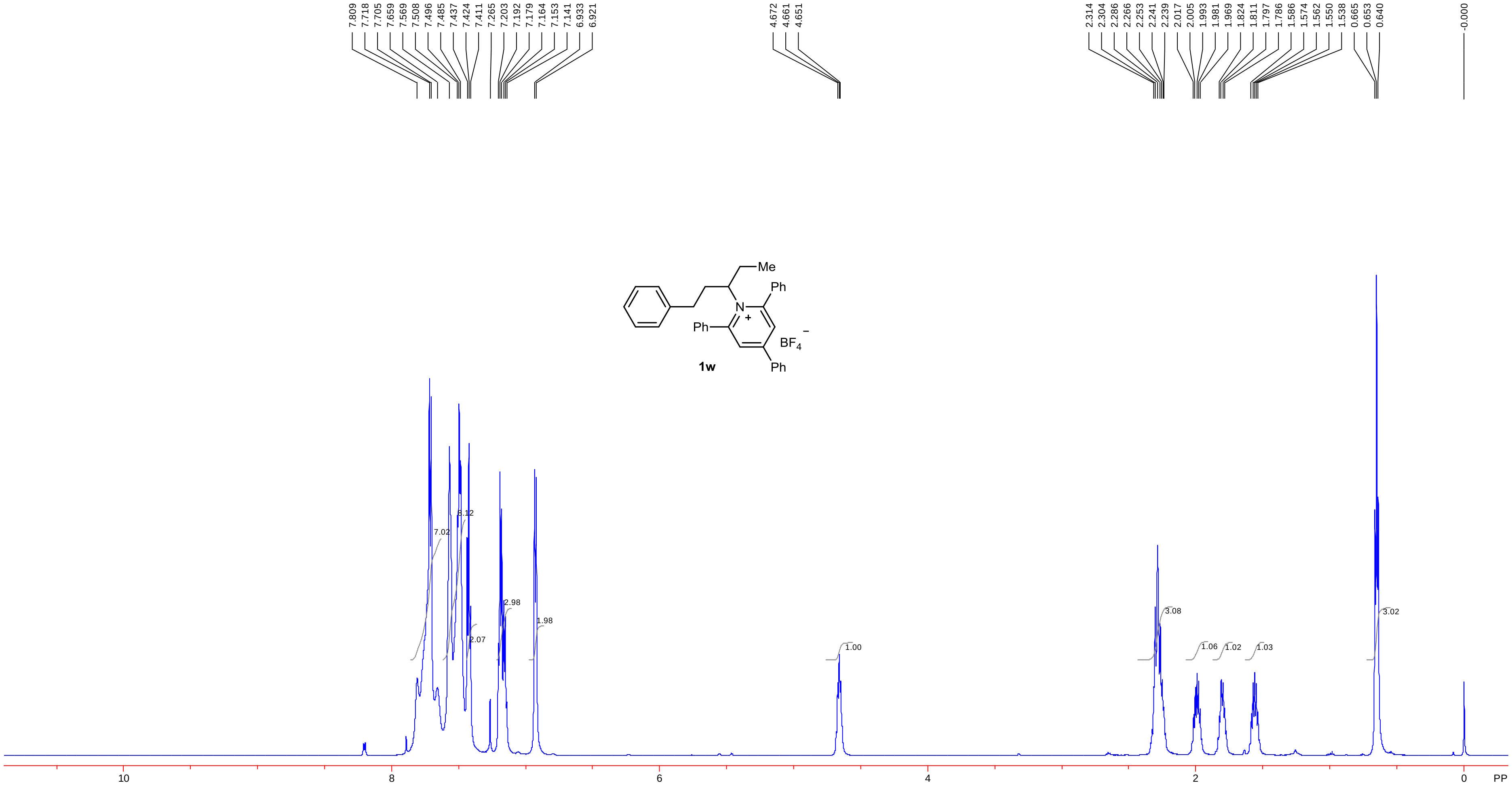

Supplementary Figure 40. ^{13}C NMR (151 MHz, $CDCl_3$)

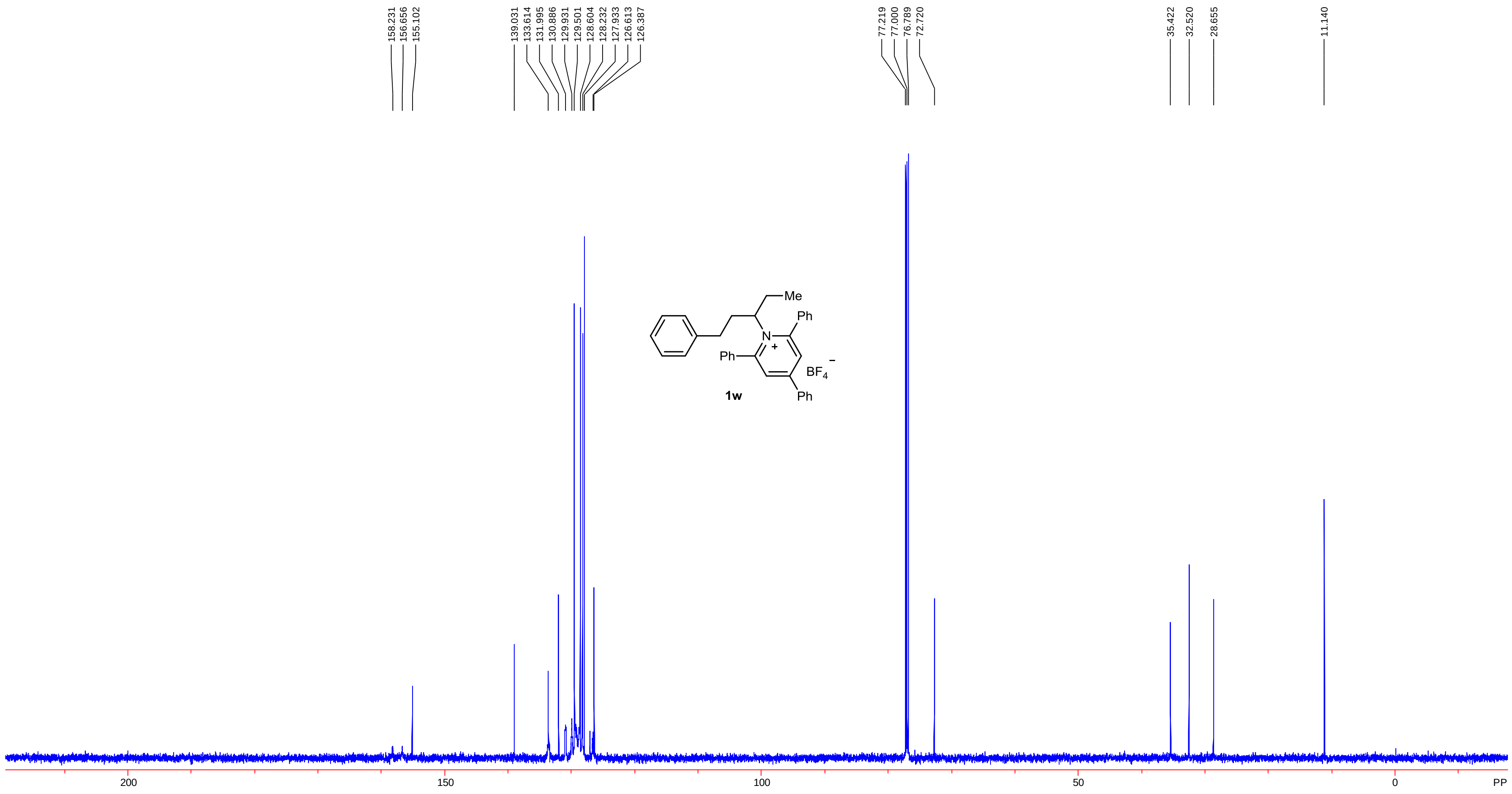

Supplementary Figure 41. ^1H NMR (600 MHz, $CDCl_3$)

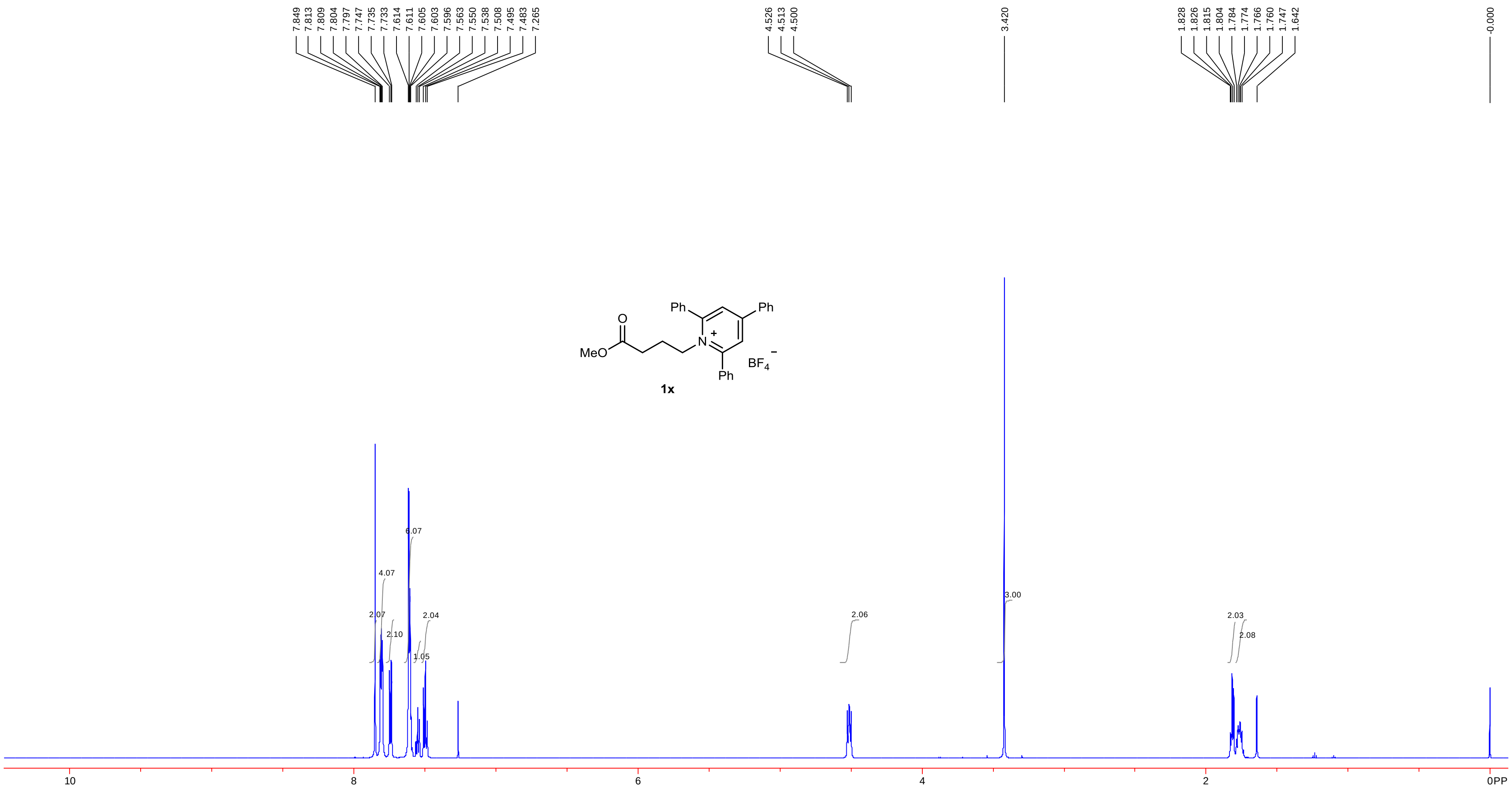

Supplementary Figure 42. ^{13}C NMR (100 MHz, $CDCl_3$)

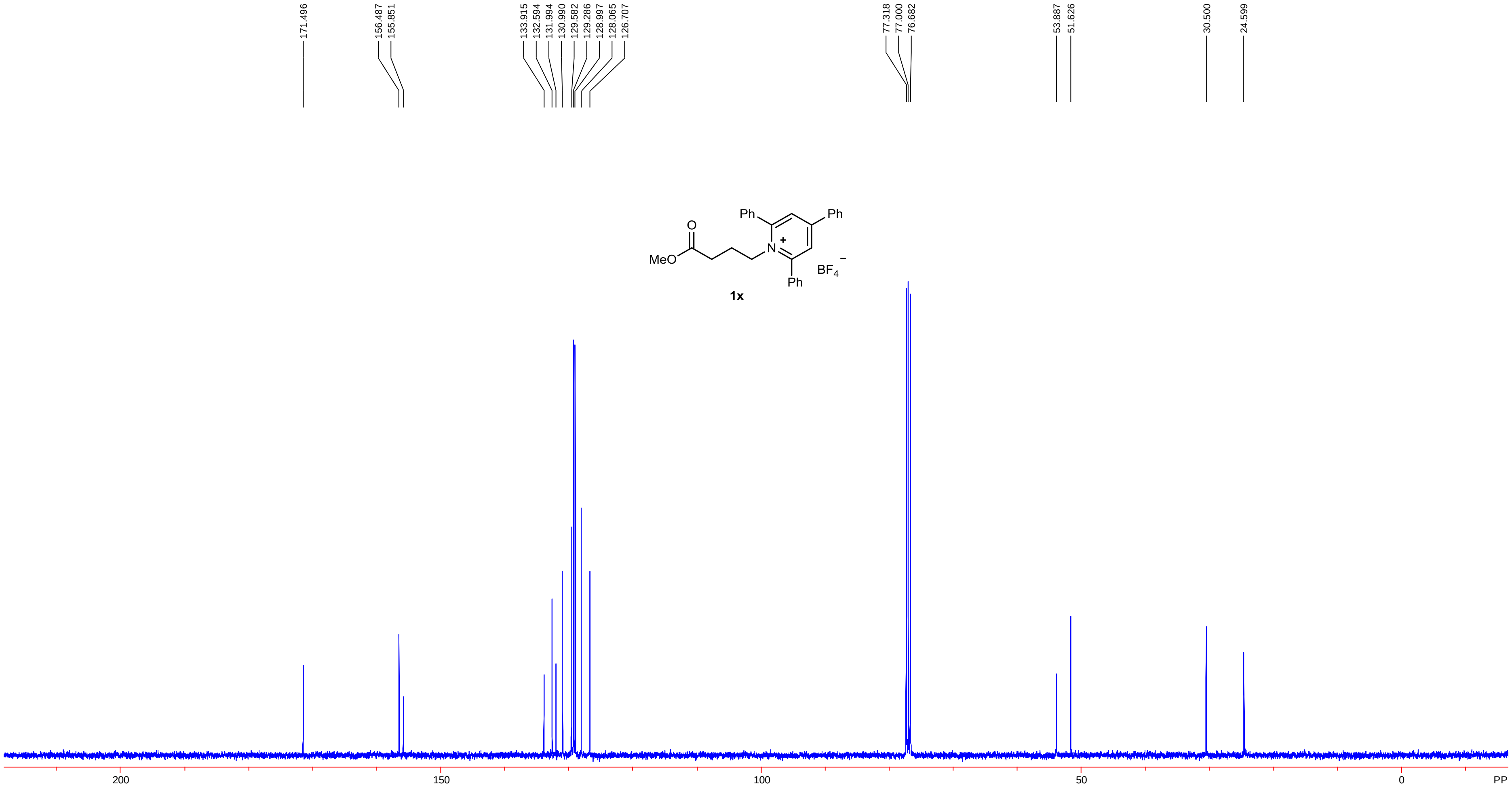

Supplementary Figure 43. ^{1}H NMR (600 MHz, $CDCl_3$)

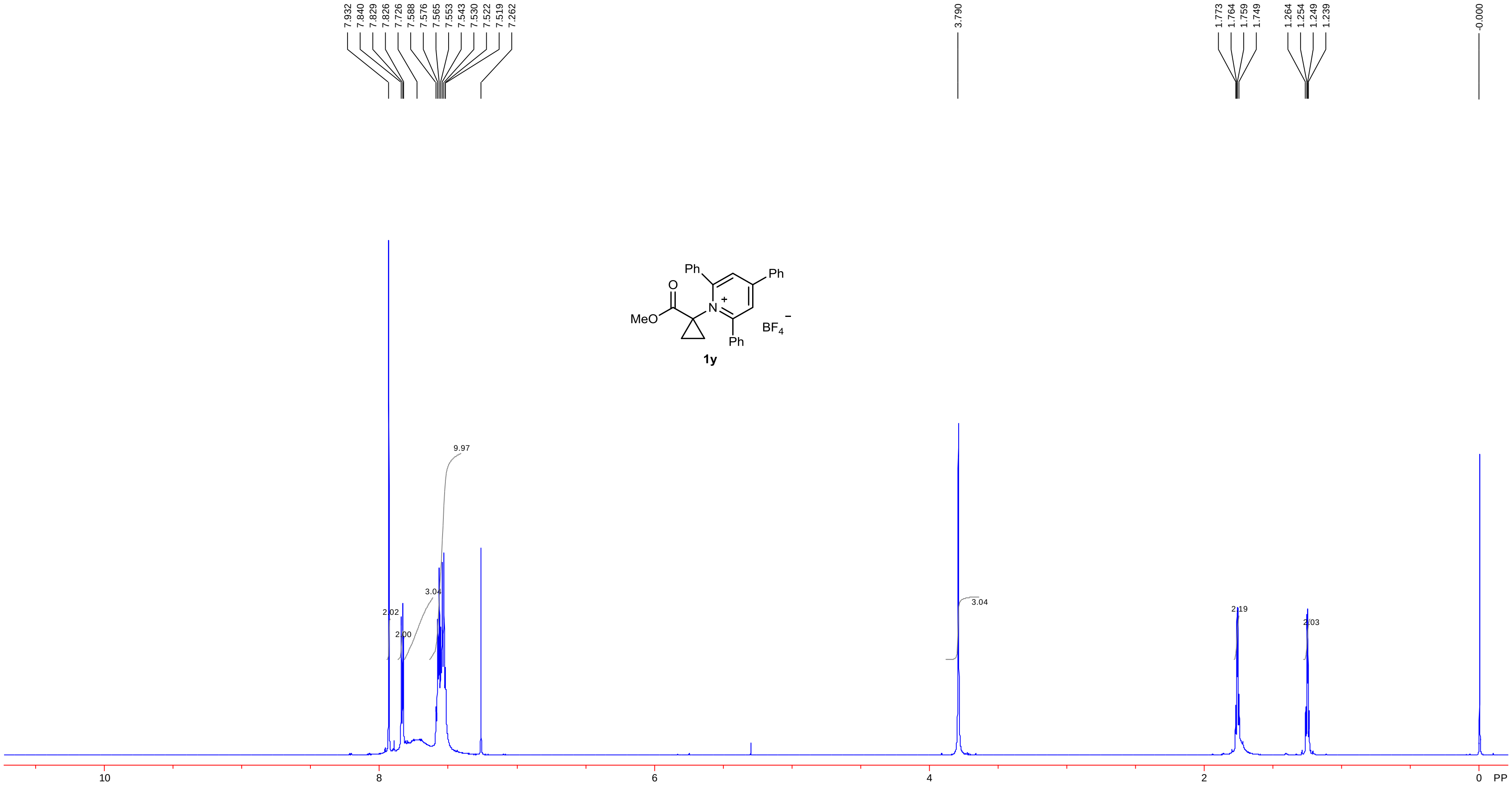

Supplementary Figure 44. ^{13}C NMR (100 MHz, $CDCl_3$)

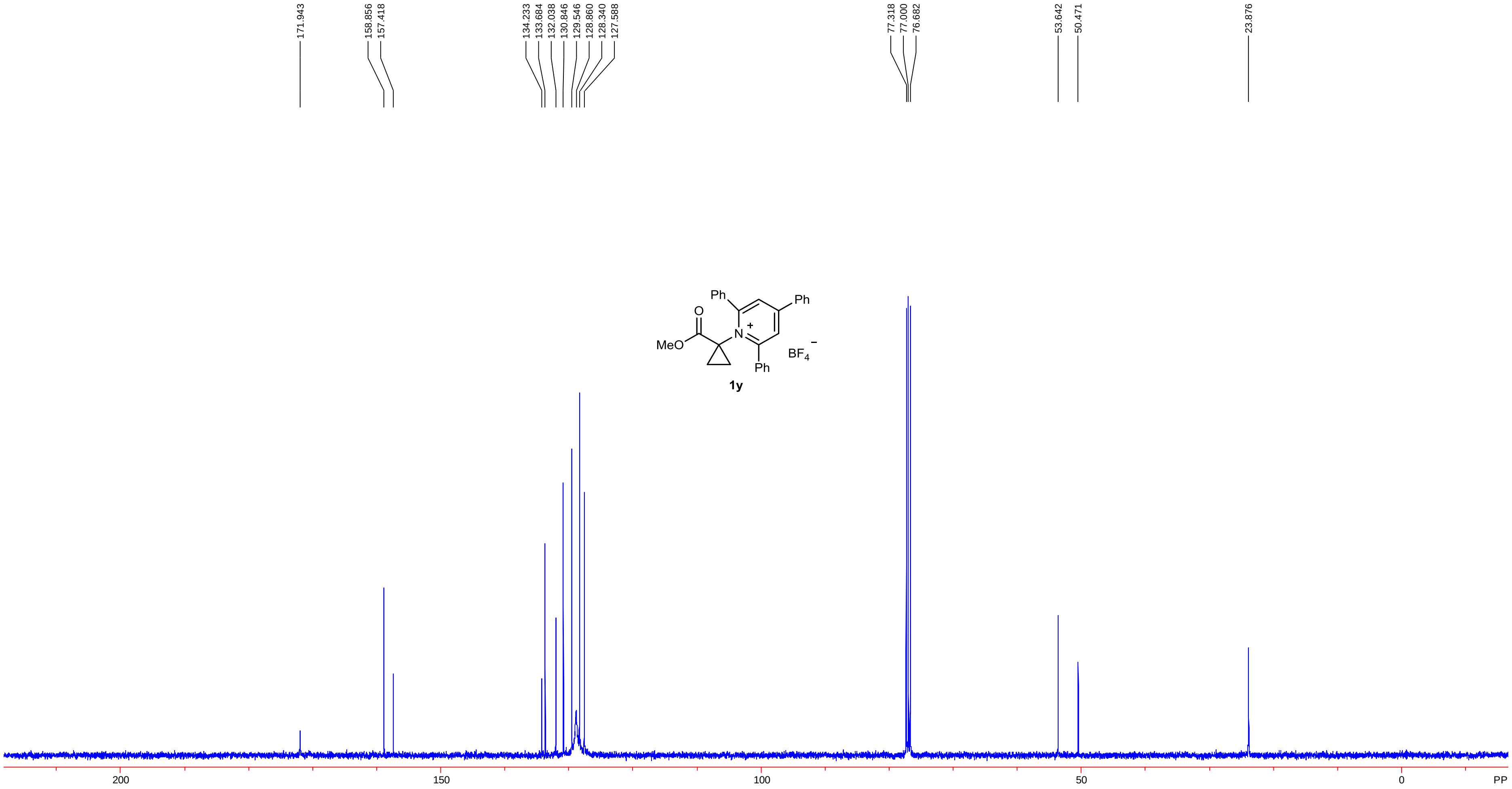

Supplementary Figure 45. ^{1}H NMR (600 MHz, $CDCl_3$)

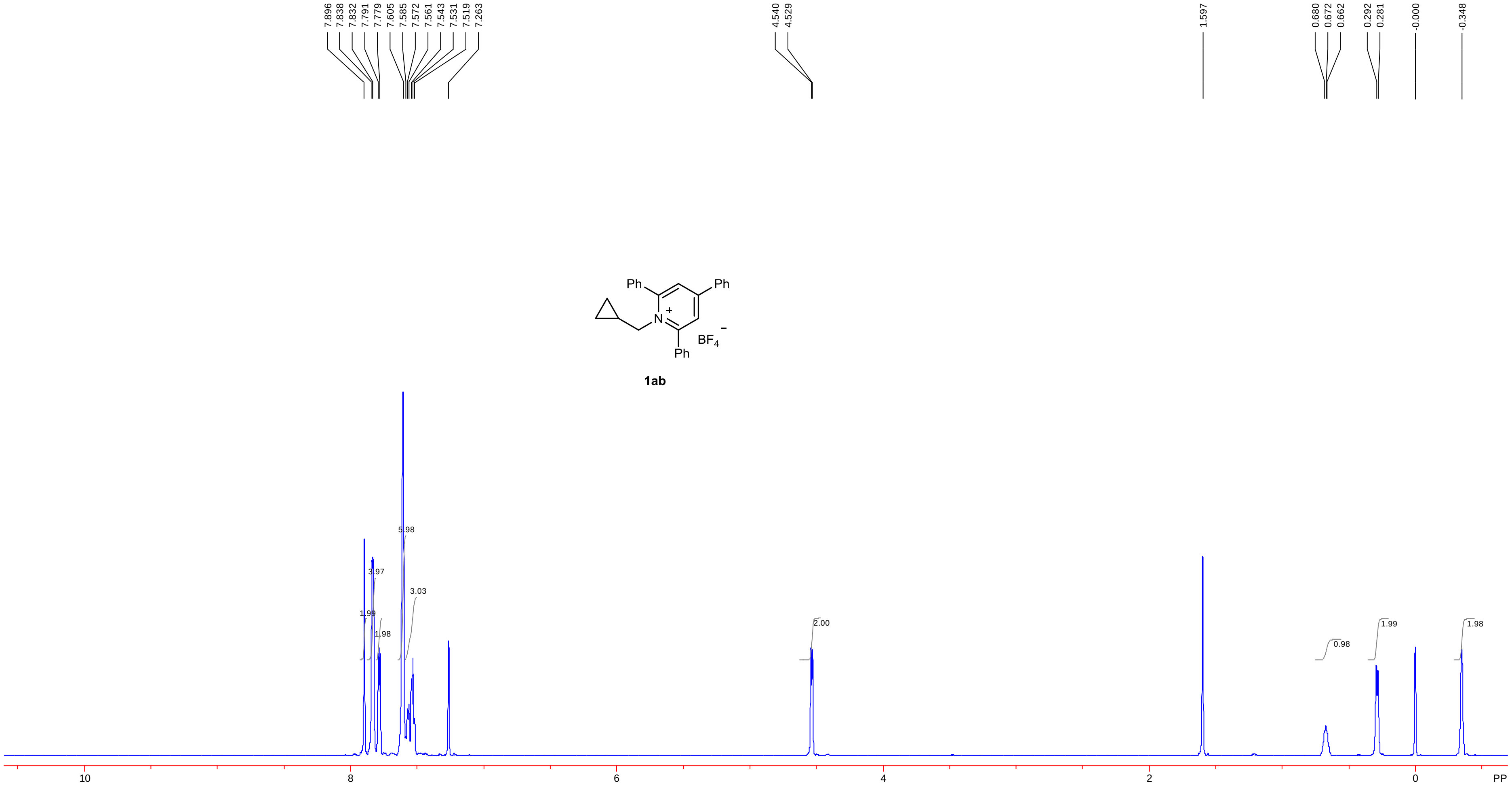

Supplementary Figure 46. ^{13}C NMR (151 MHz, $CDCl_3$)

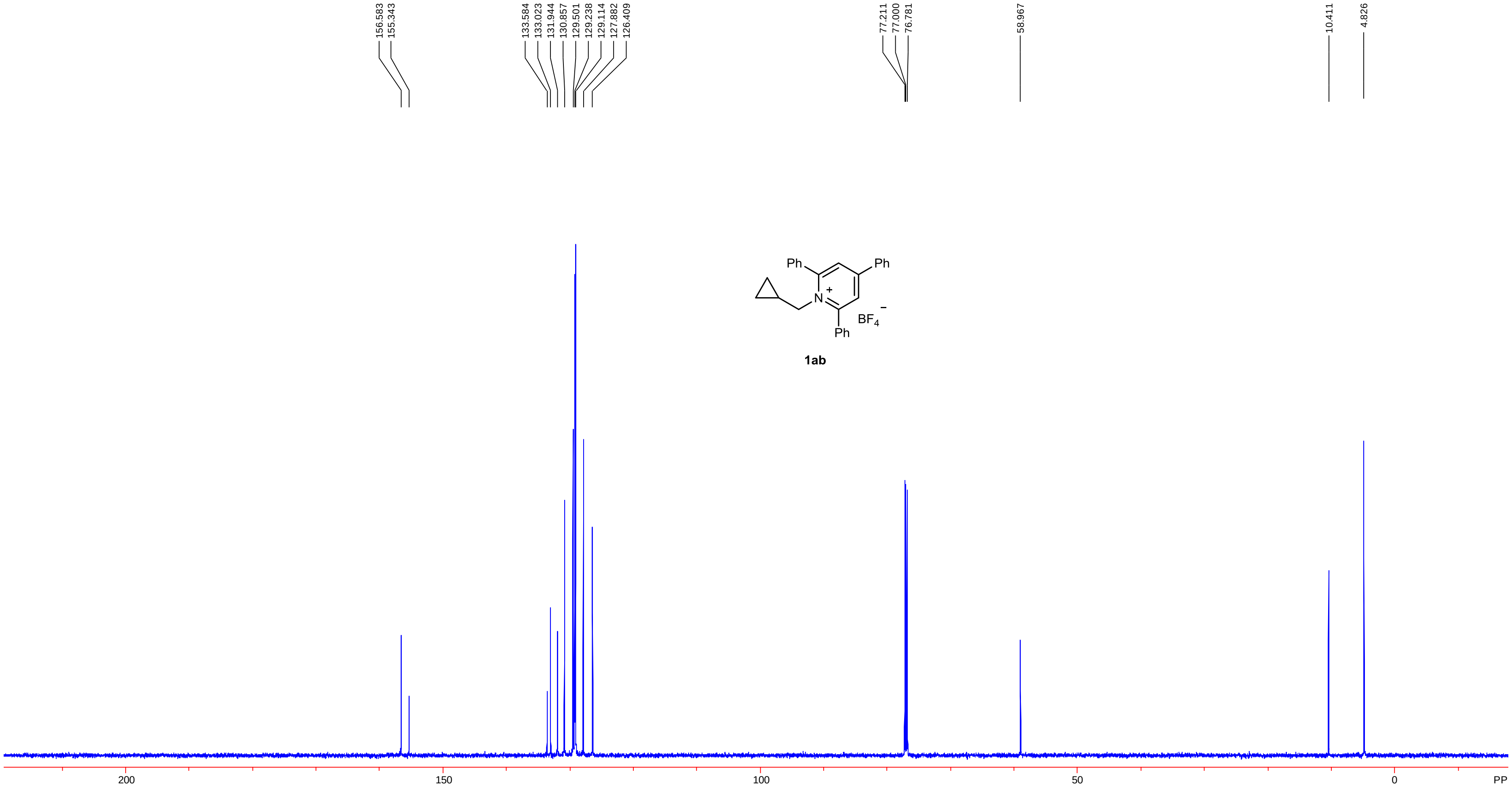

Supplementary Figure 47. ^{1}H NMR (600 MHz, $CDCl_3$)

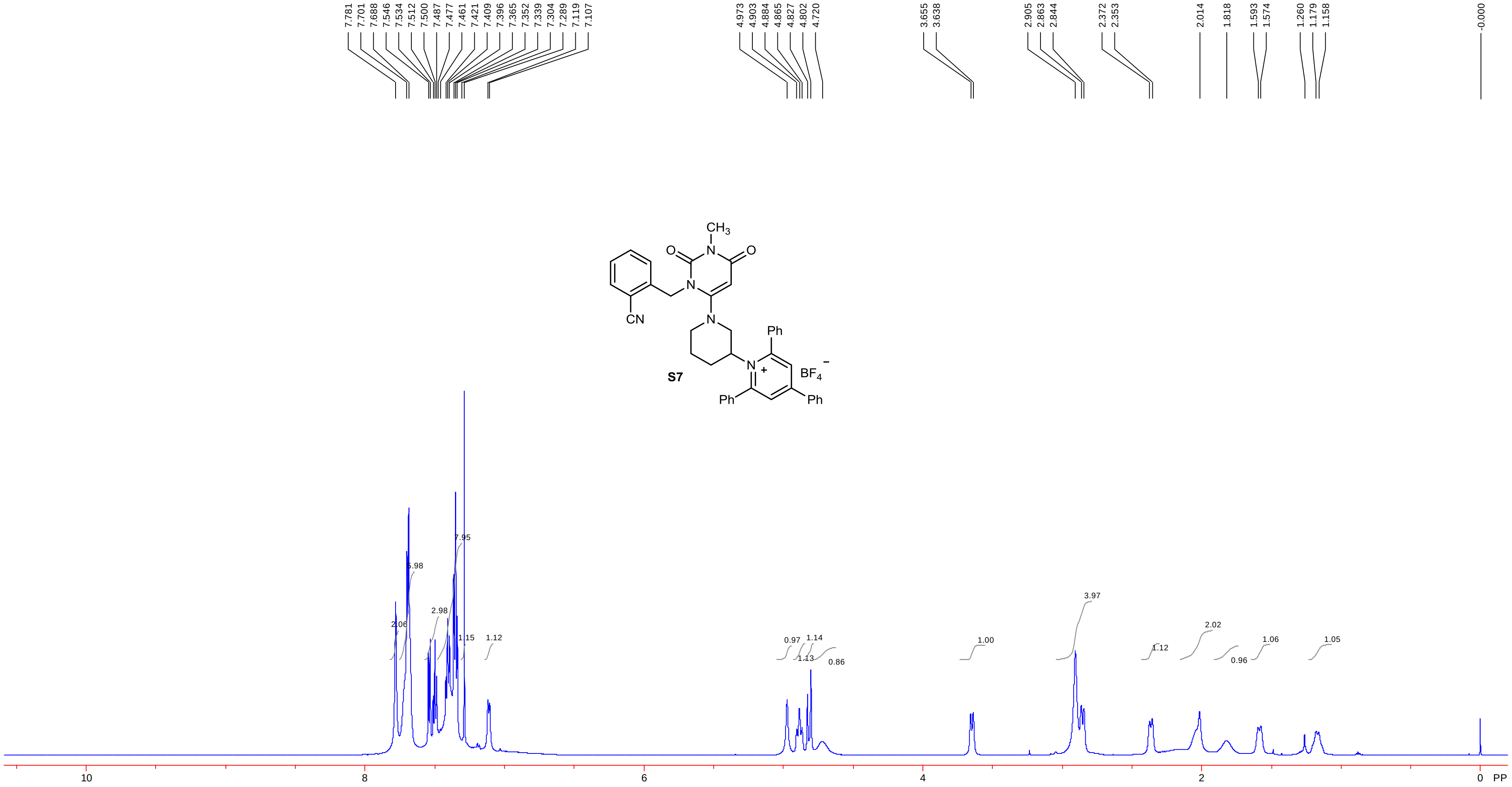

Supplementary Figure 48. ^{13}C NMR (100 MHz, $CDCl_3$)

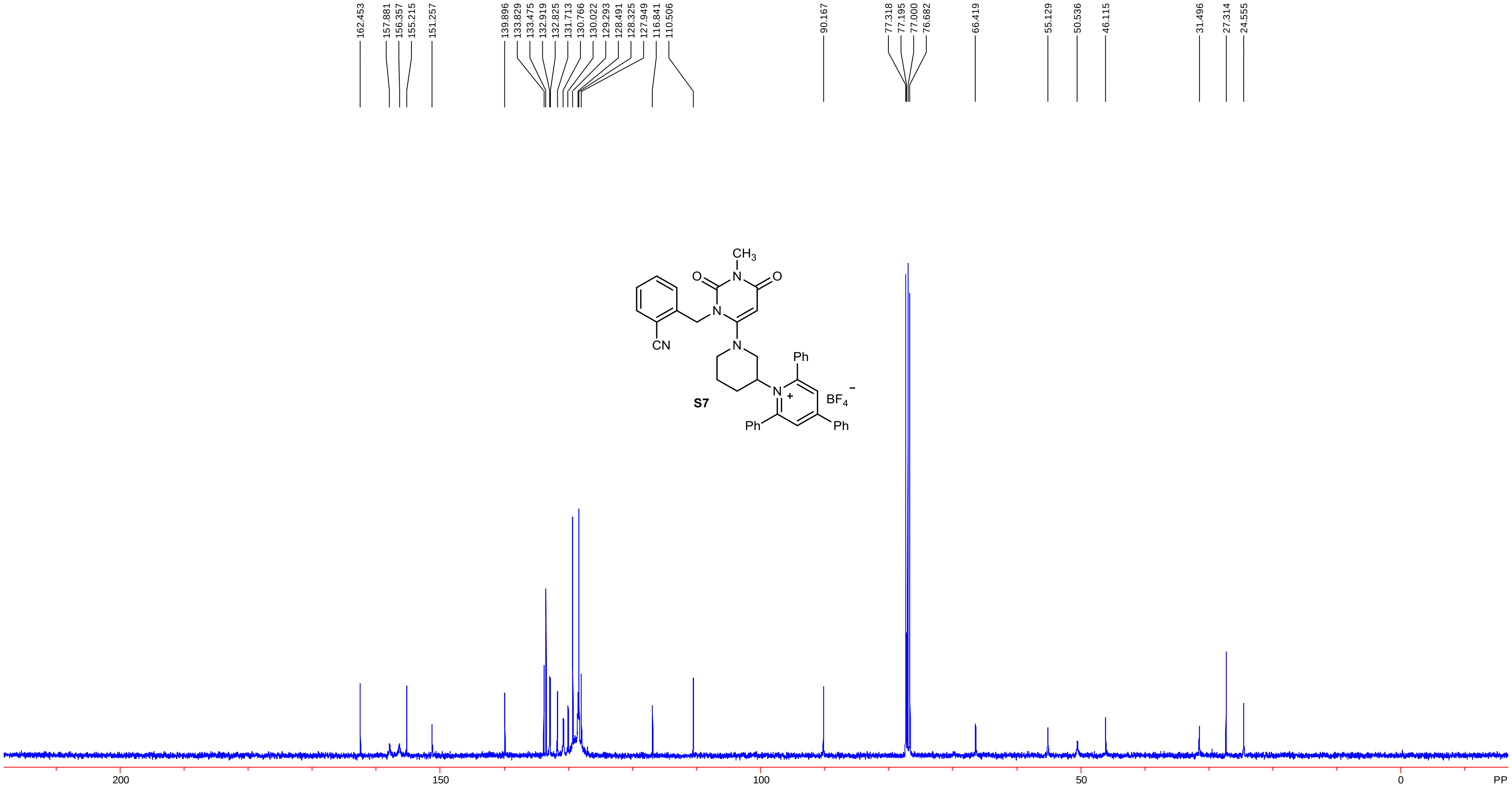

Supplementary Figure 49. ^{1}H NMR(400 MHz, $CDCl_3$)

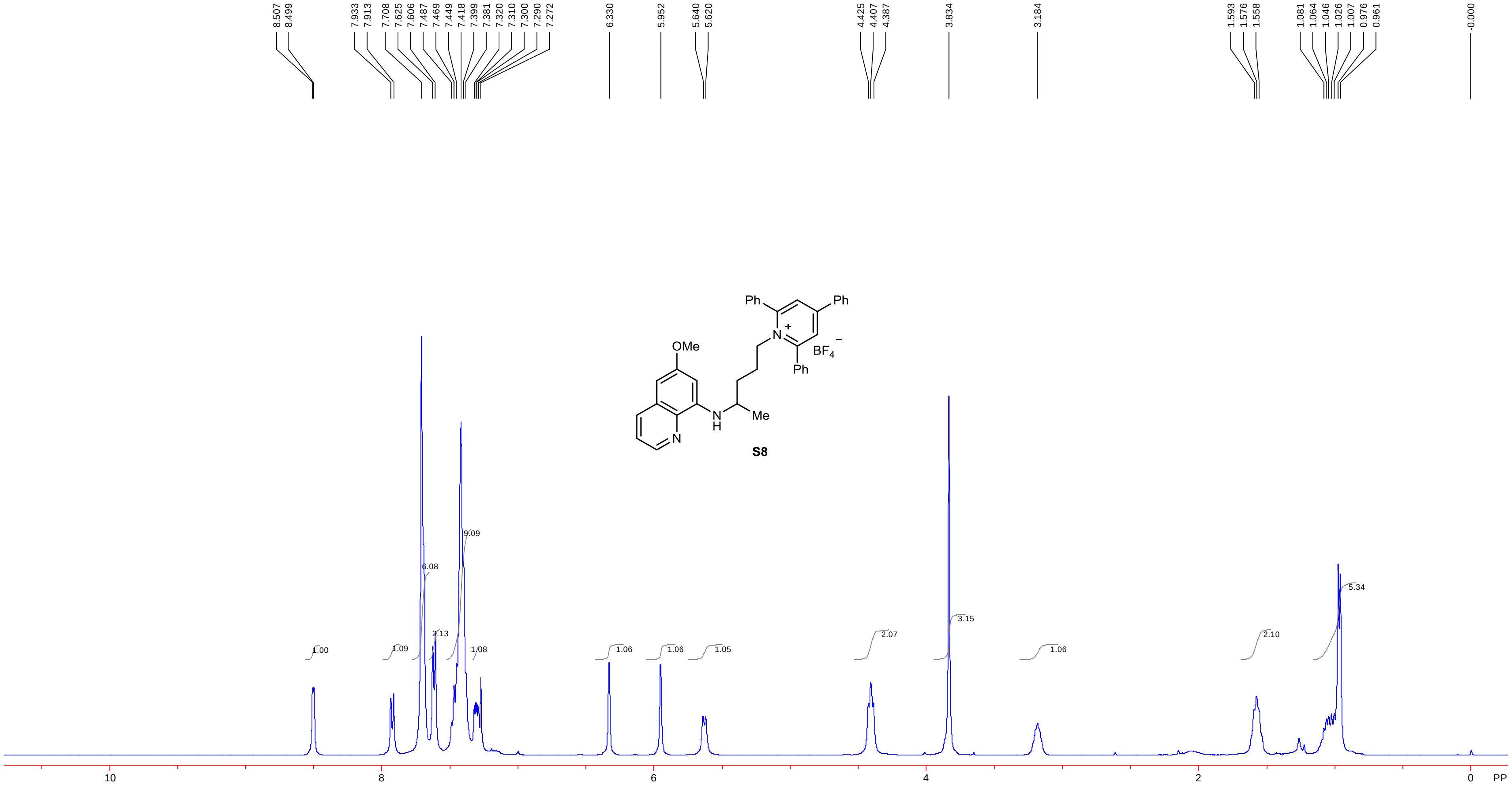

Supplementary Figure 50. ^{13}C NMR(100 MHz, $CDCl_3$)

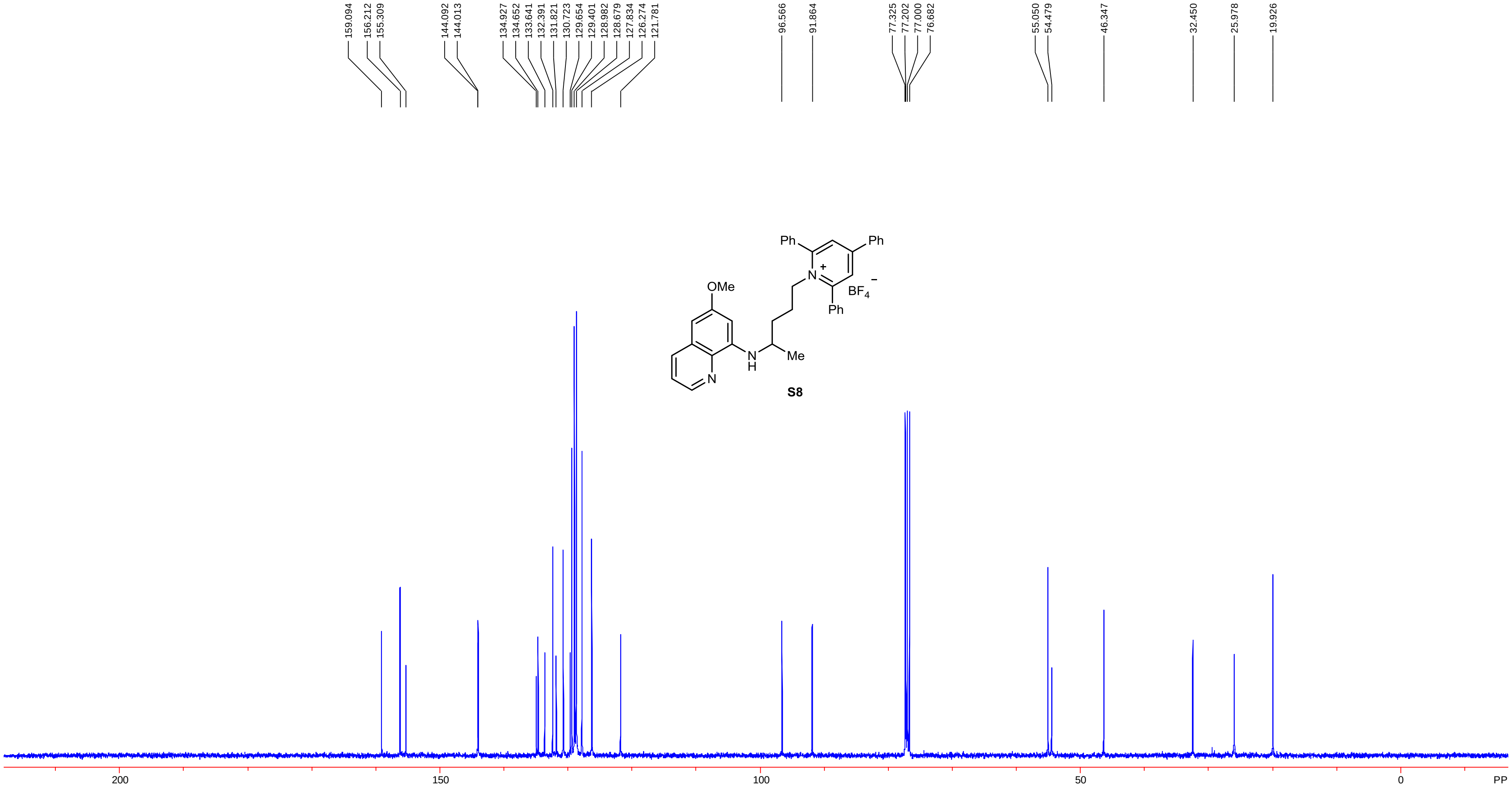

Supplementary Figure 51. ^{1}H NMR(400 MHz, $CDCl_3$)

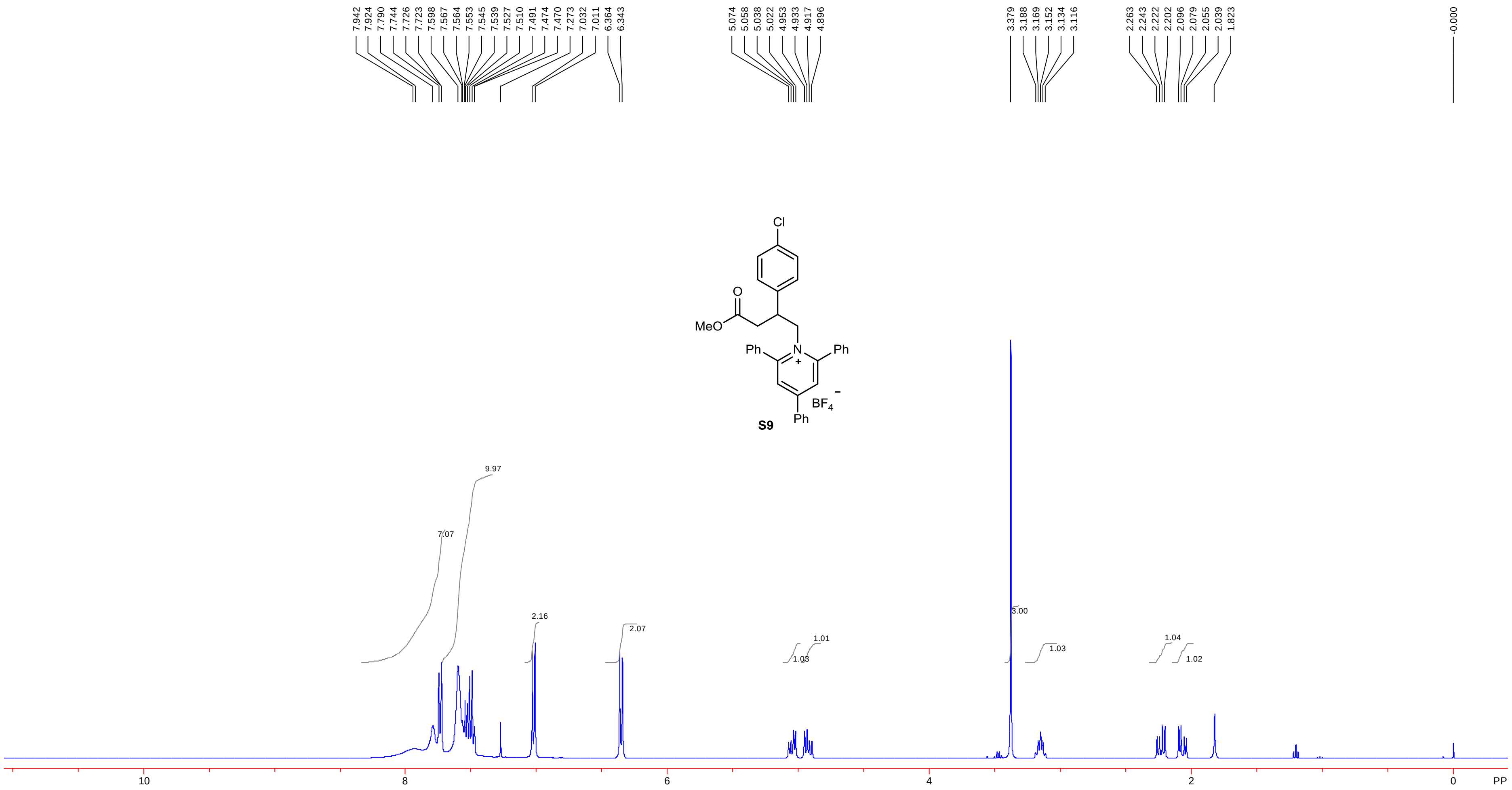

Supplementary Figure 52. ^{13}C NMR(100 MHz, $CDCl_3$)

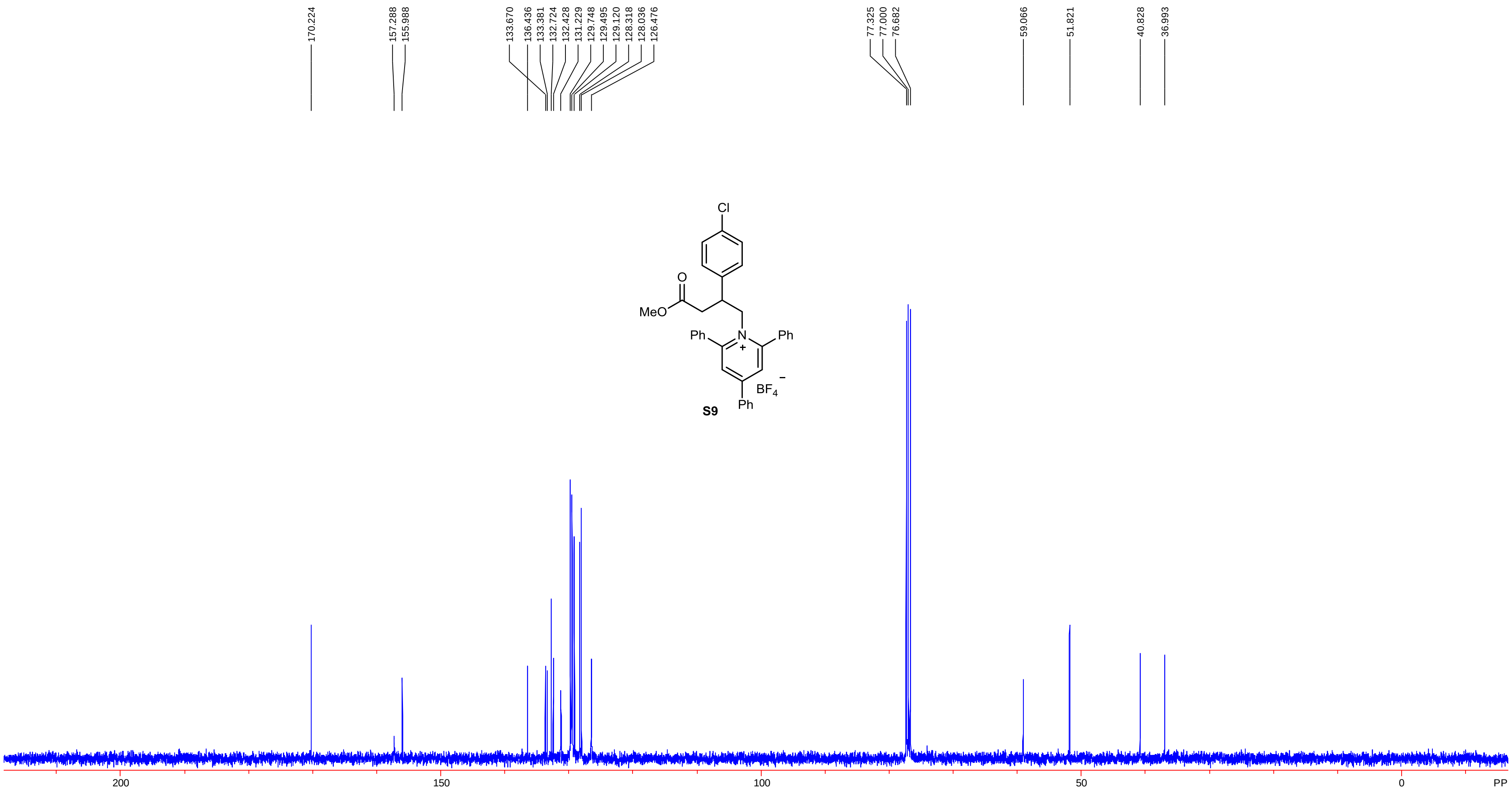

Supplementary Figure 53. ^{1}H NMR(400 MHz, $CDCl_3$)

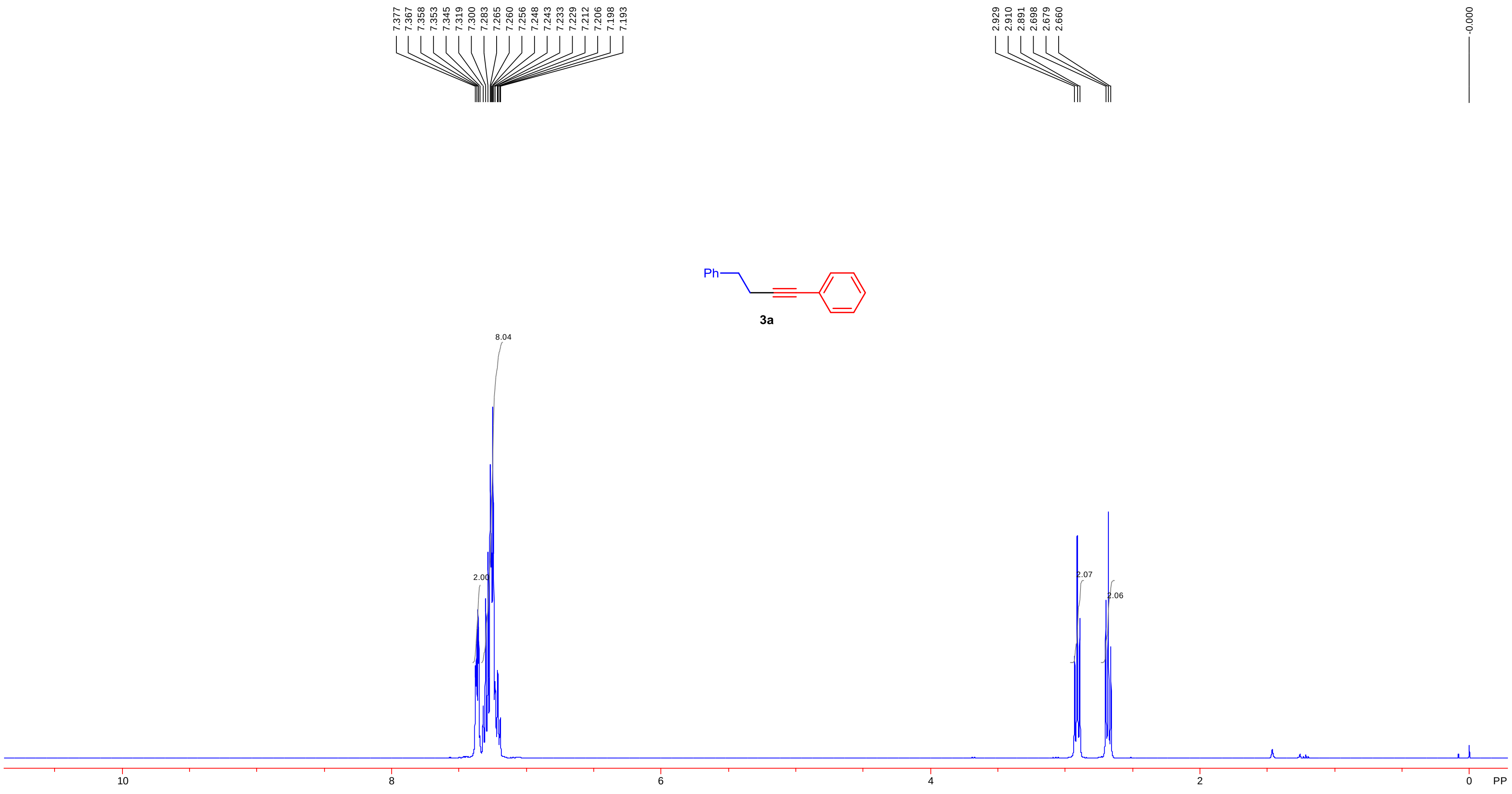

Supplementary Figure 54. ^{13}C NMR(100 MHz, $CDCl_3$)

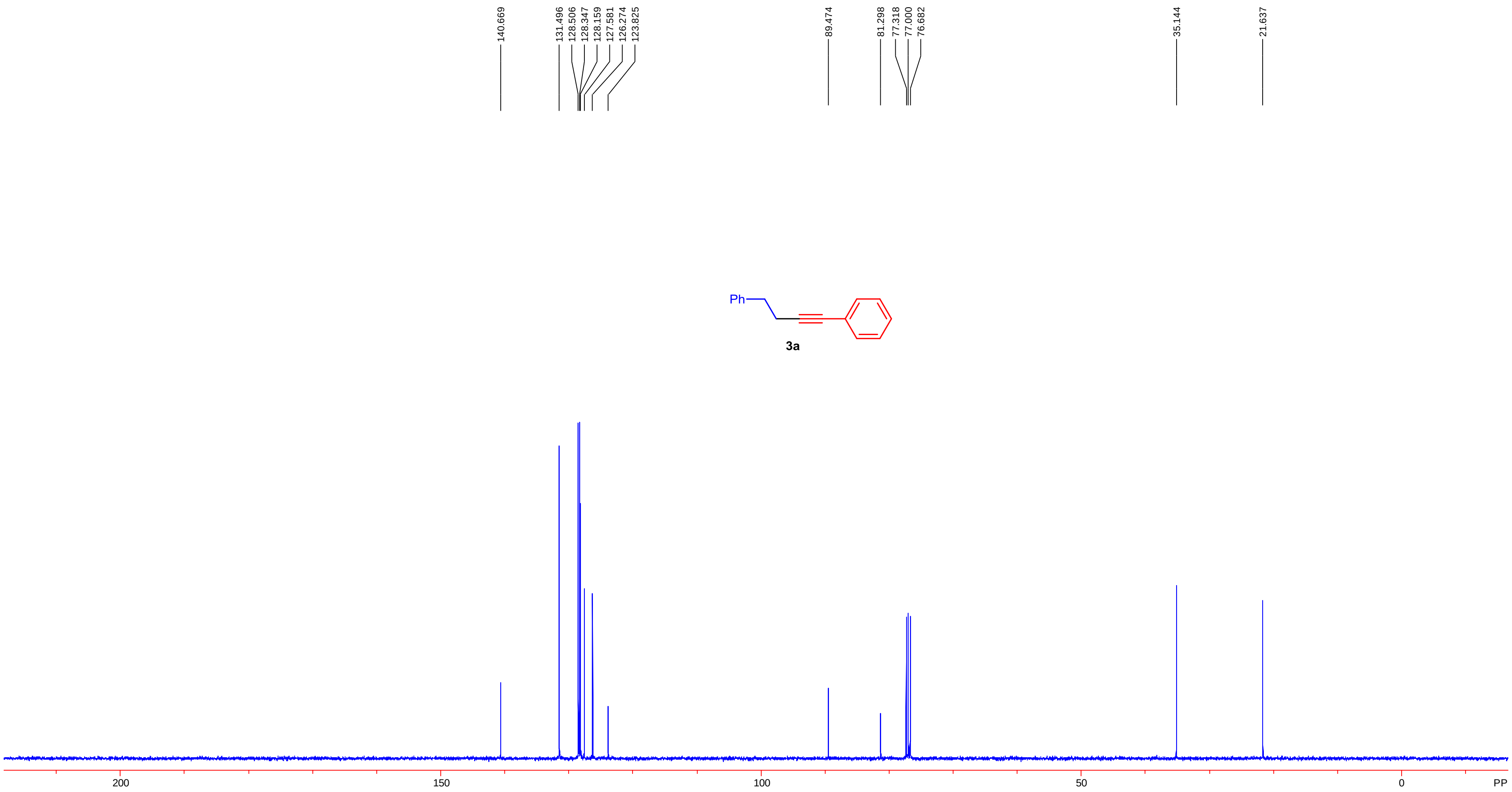

Supplementary Figure 55. ^{1}H NMR(400 MHz, $CDCl_3$)

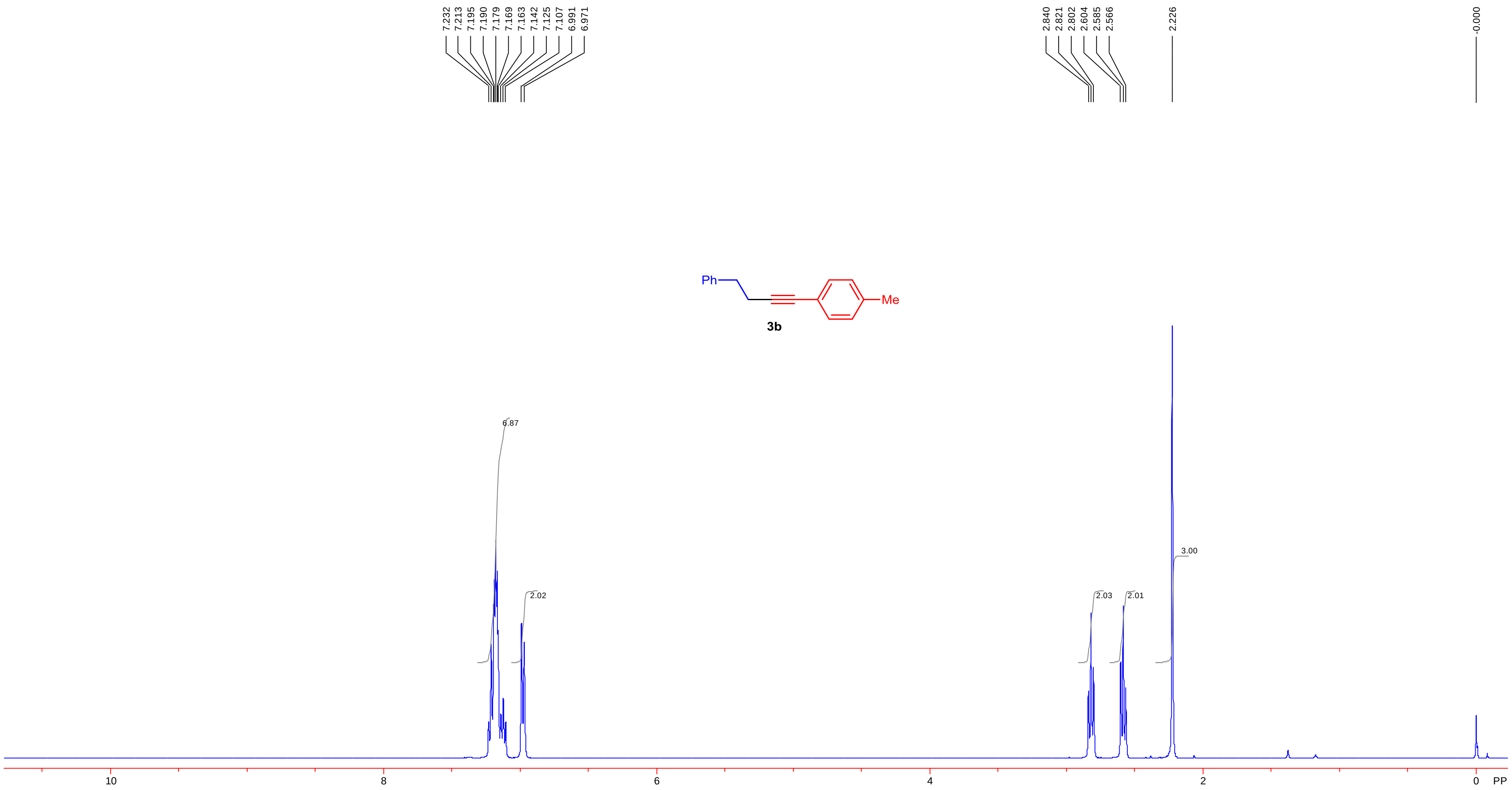

Supplementary Figure 56. ^{13}C NMR(100 MHz, $CDCl_3$)

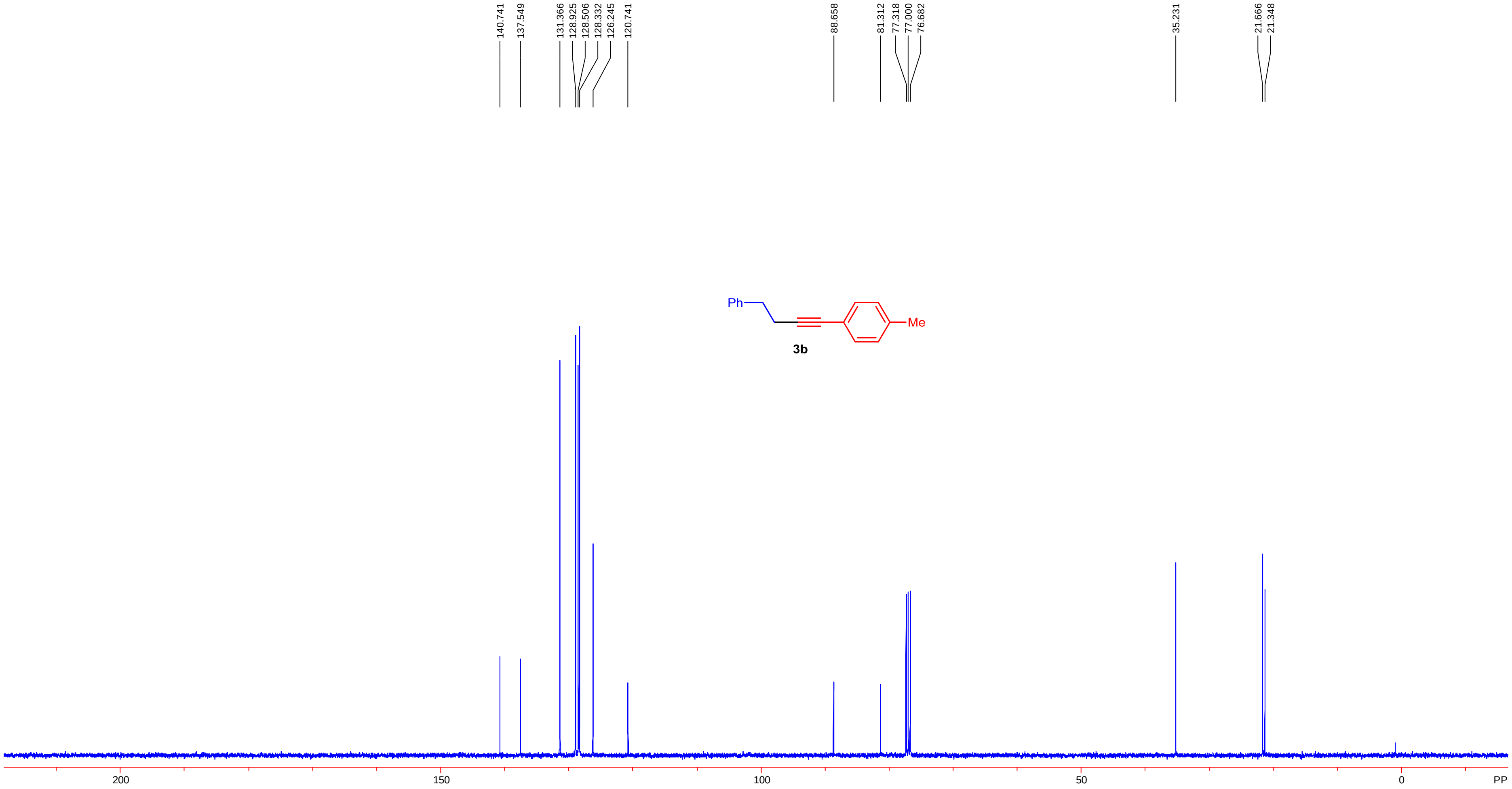

Supplementary Figure 57. ^{1}H NMR(400 MHz, $CDCl_3$)

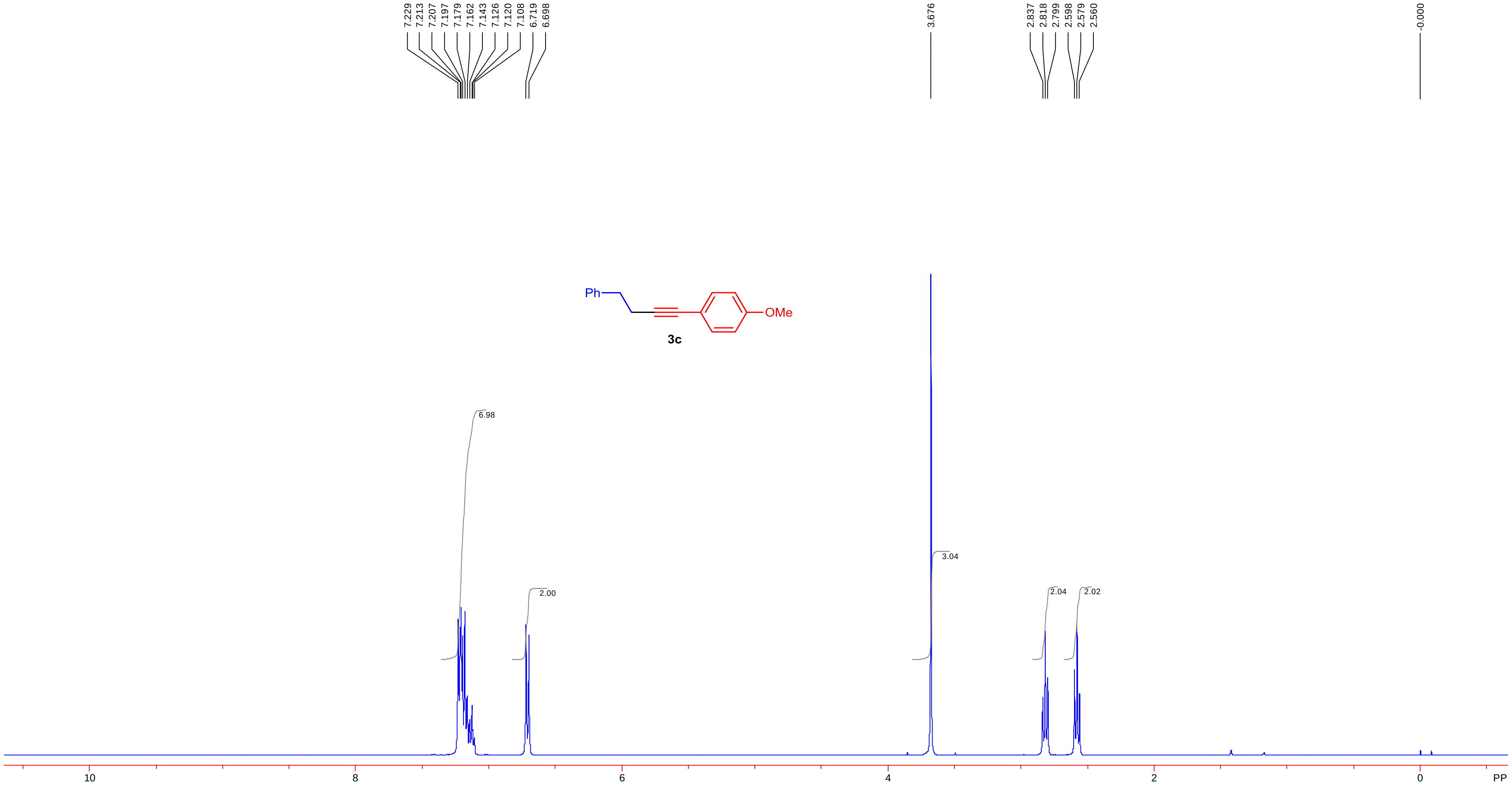

Supplementary Figure 58. ^{13}C NMR(100 MHz, $CDCl_3$)

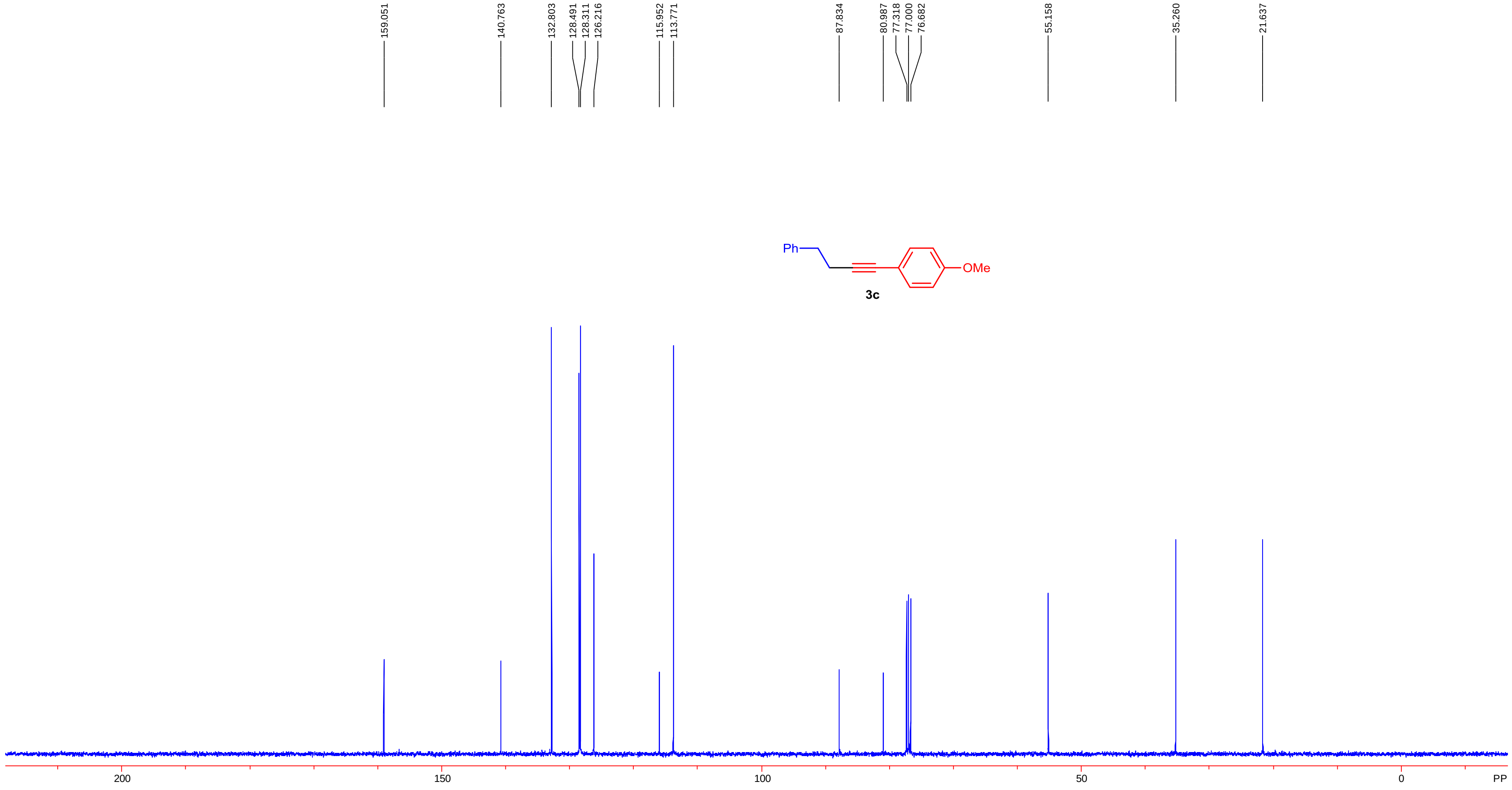

Supplementary Figure 59. ^{1}H NMR(400 MHz, $CDCl_3$)

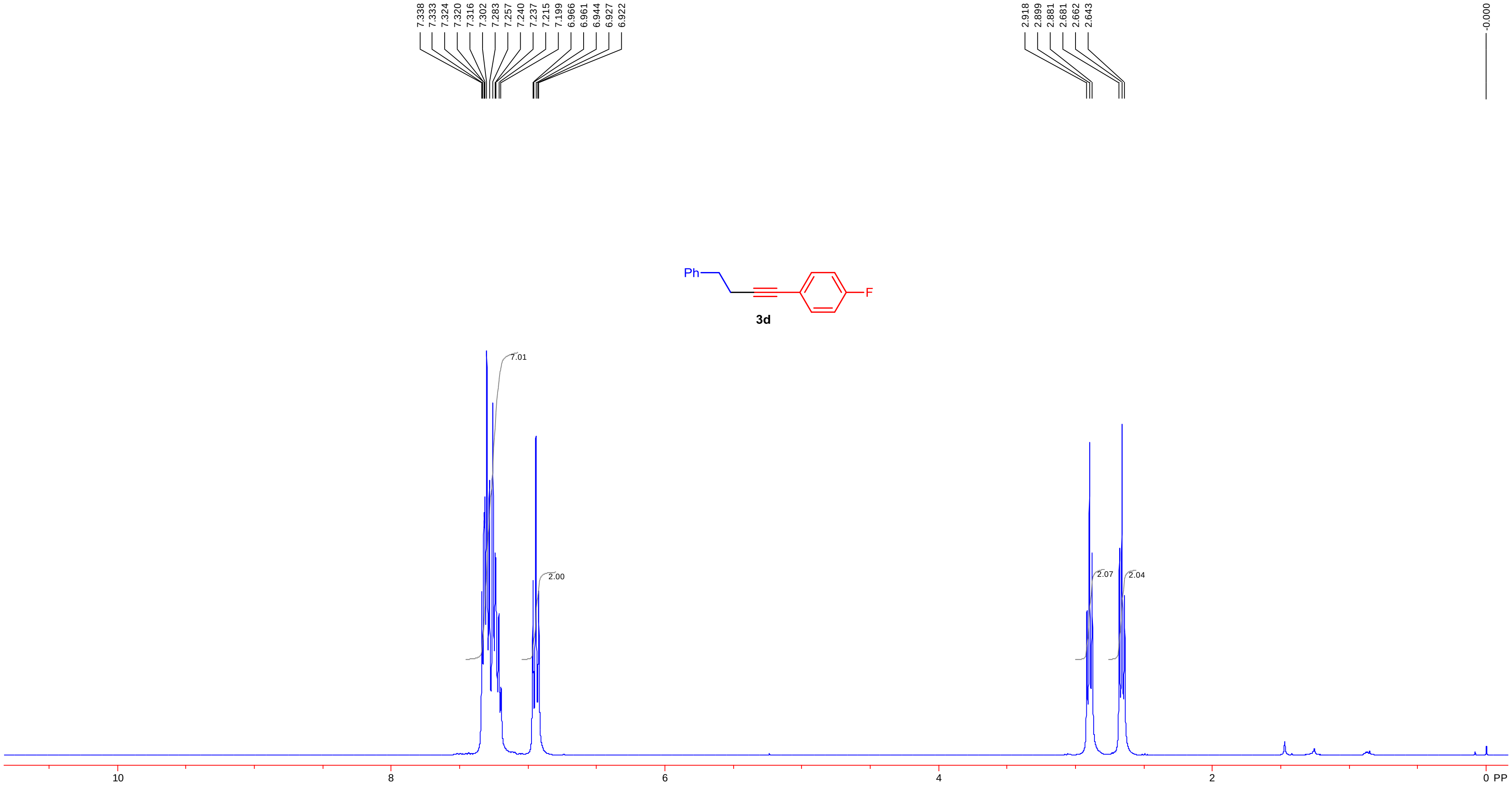

Supplementary Figure 60. ^{13}C NMR(100 MHz, $CDCl_3$)

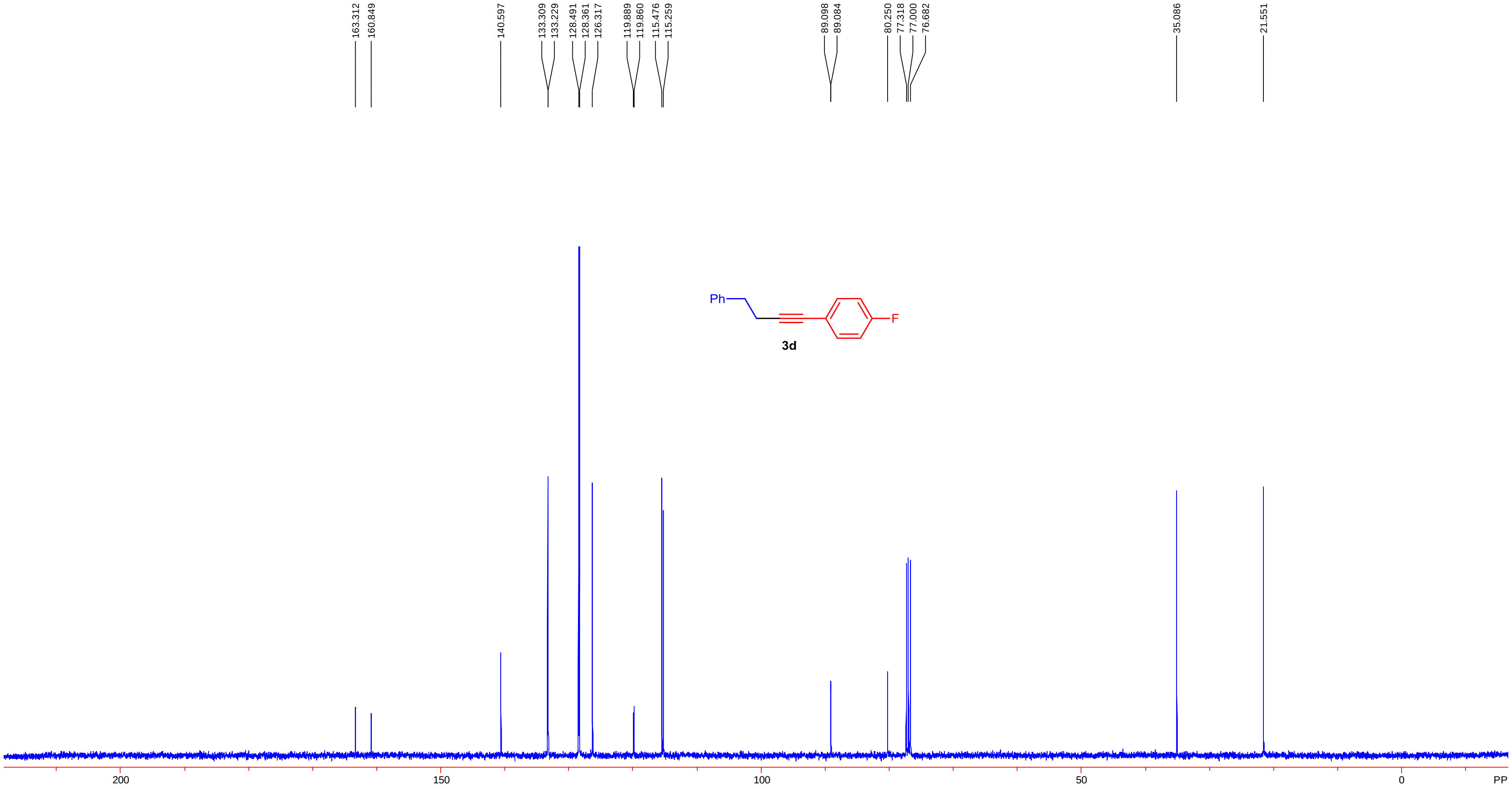

Supplementary Figure 61. ^{1}H NMR(400 MHz, $CDCl_3$)

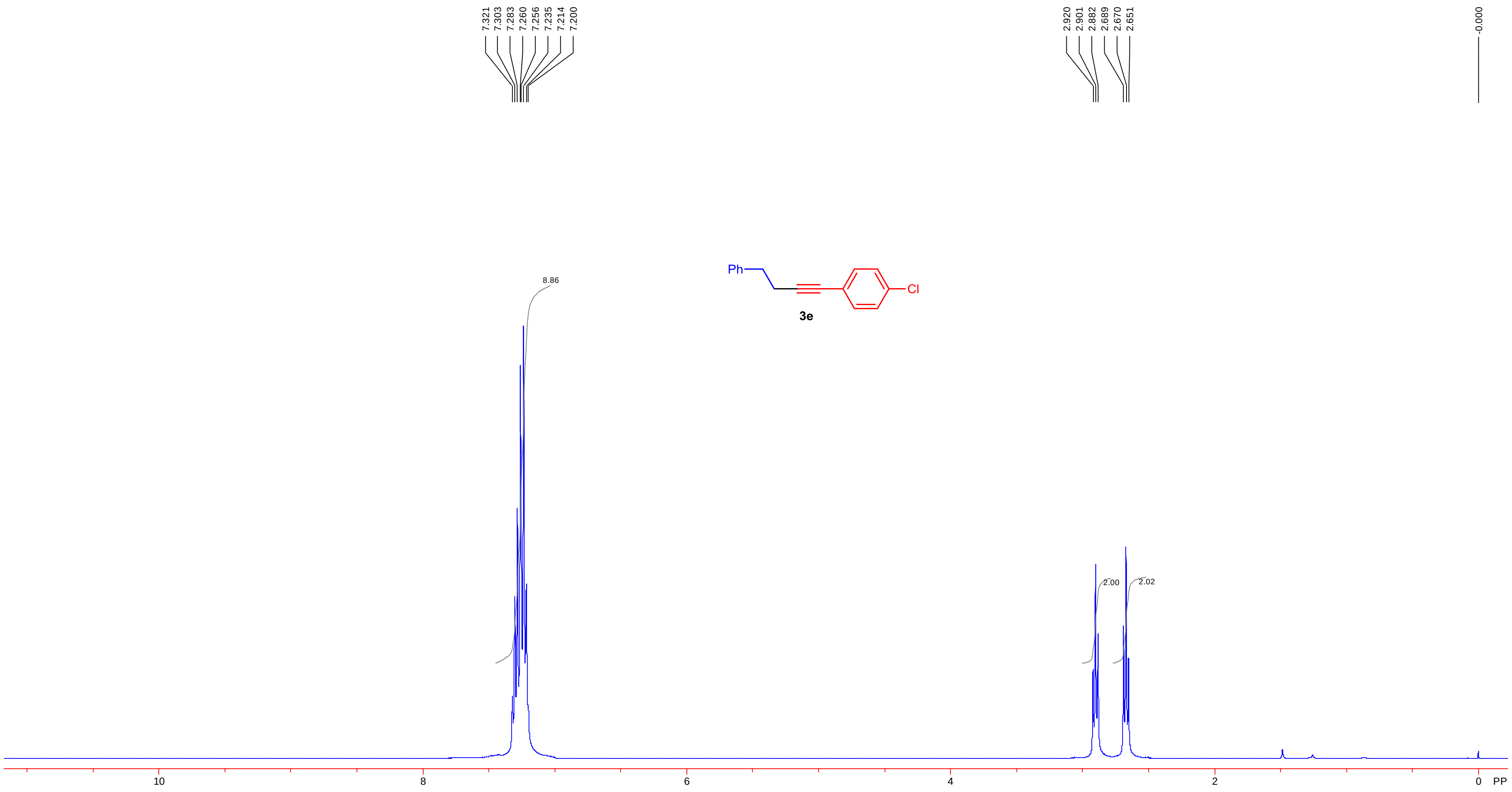

Supplementary Figure 62. ^{13}C NMR(100 MHz, $CDCl_3$)

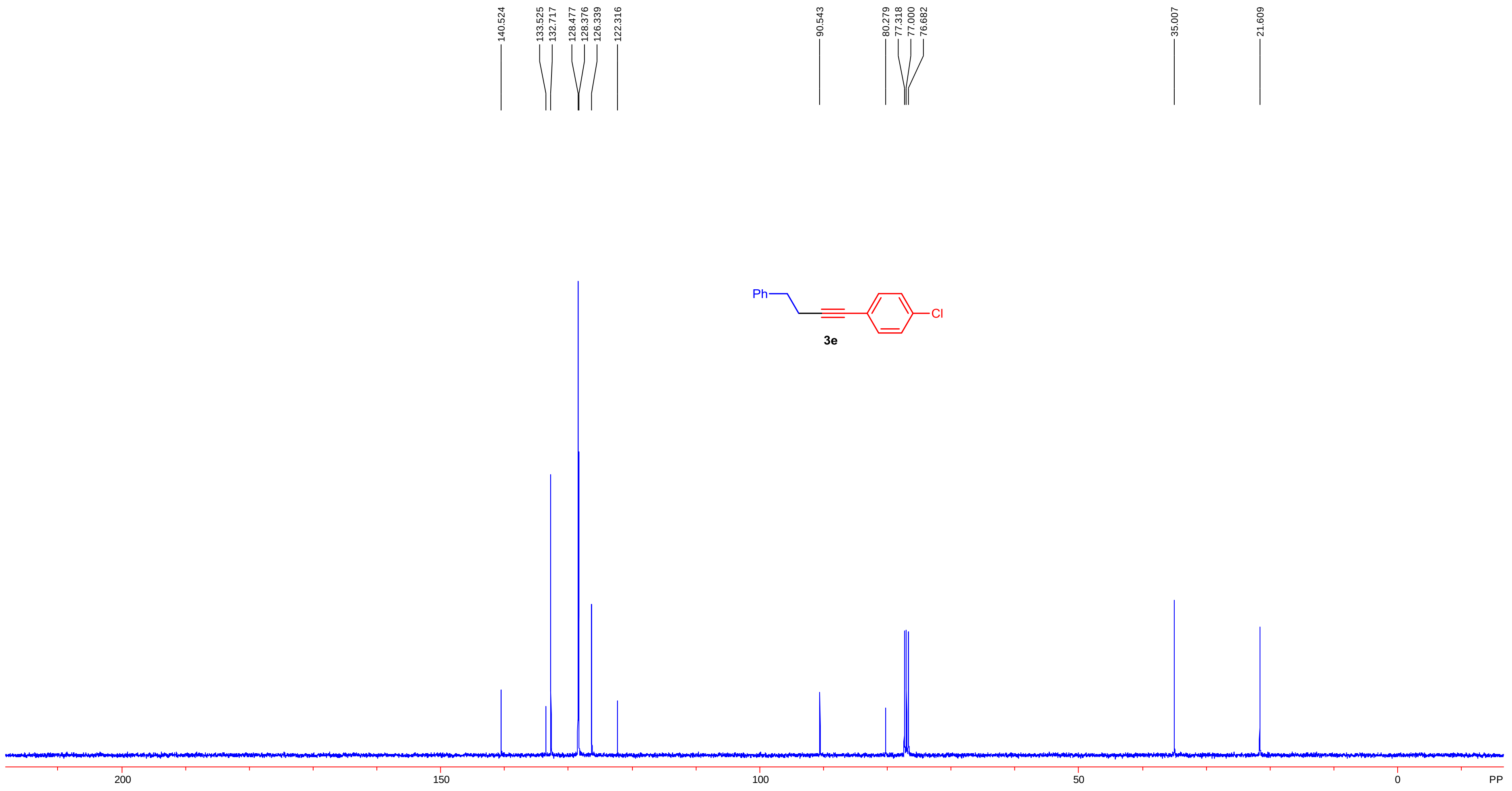

Supplementary Figure 63. ^{1}H NMR(400 MHz, $CDCl_3$)

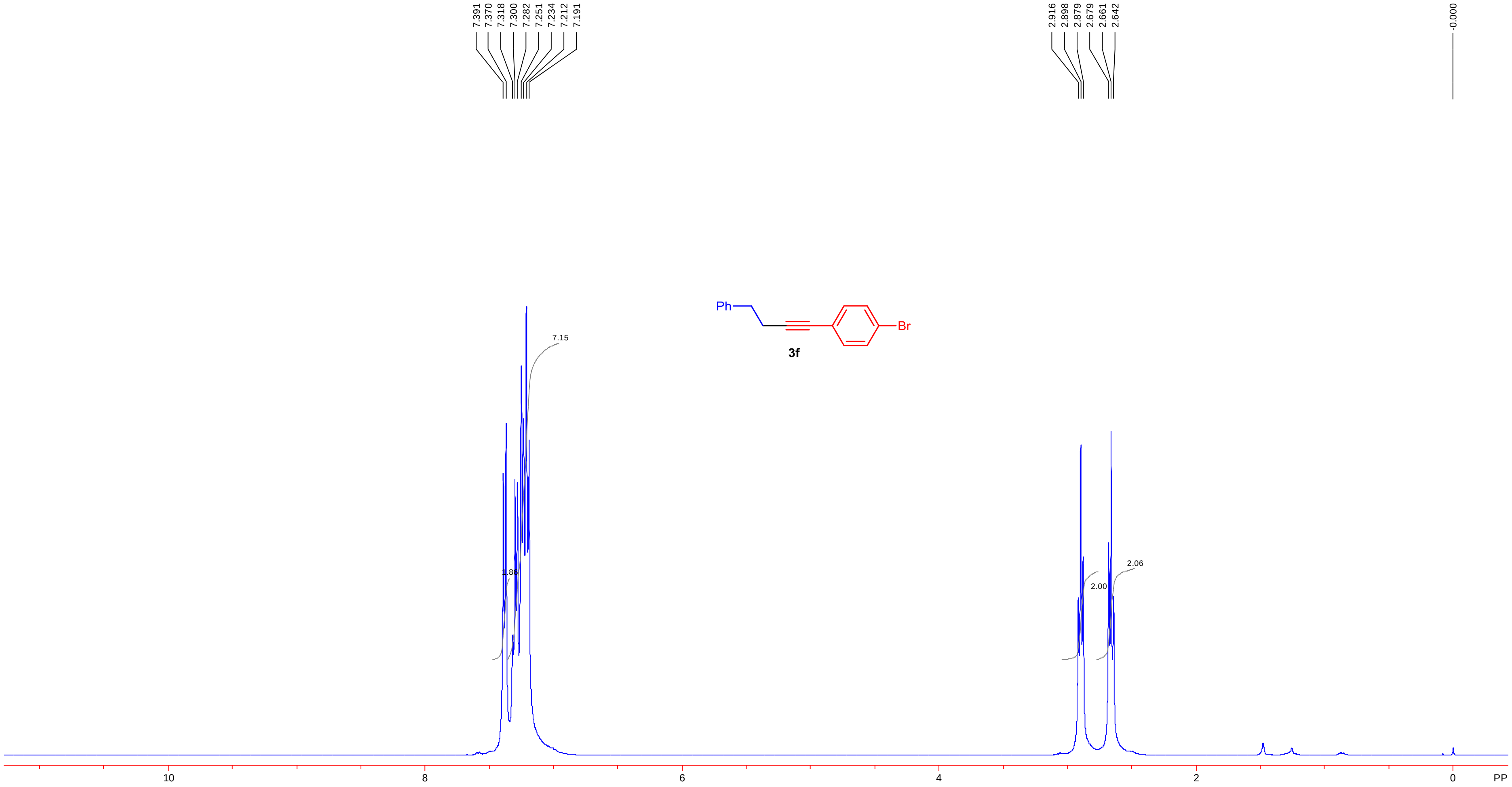

Supplementary Figure 64. ^{13}C NMR(100 MHz, $CDCl_3$)

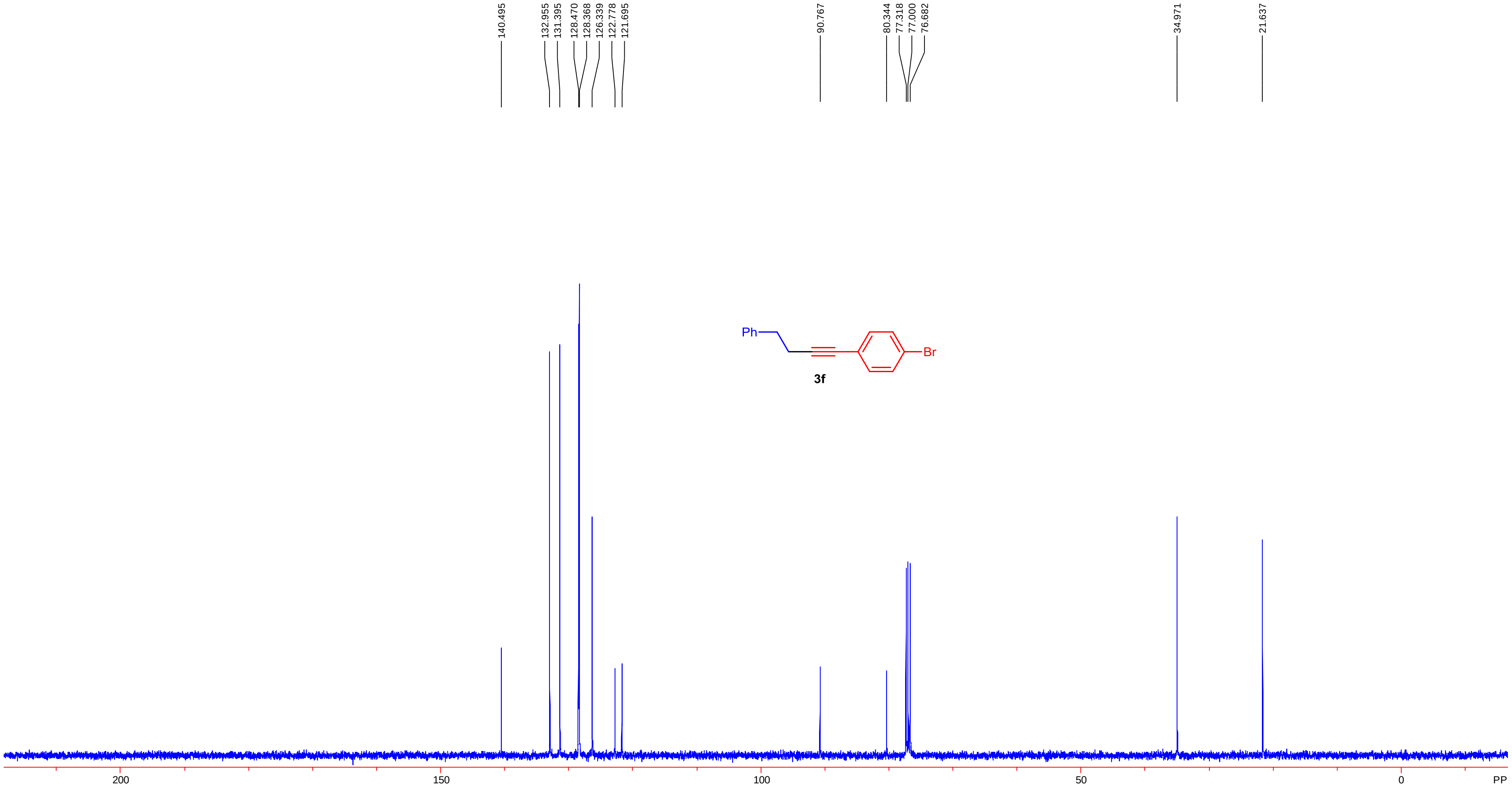

Supplementary Figure 65. ^1H NMR(400 MHz, $CDCl_3$)

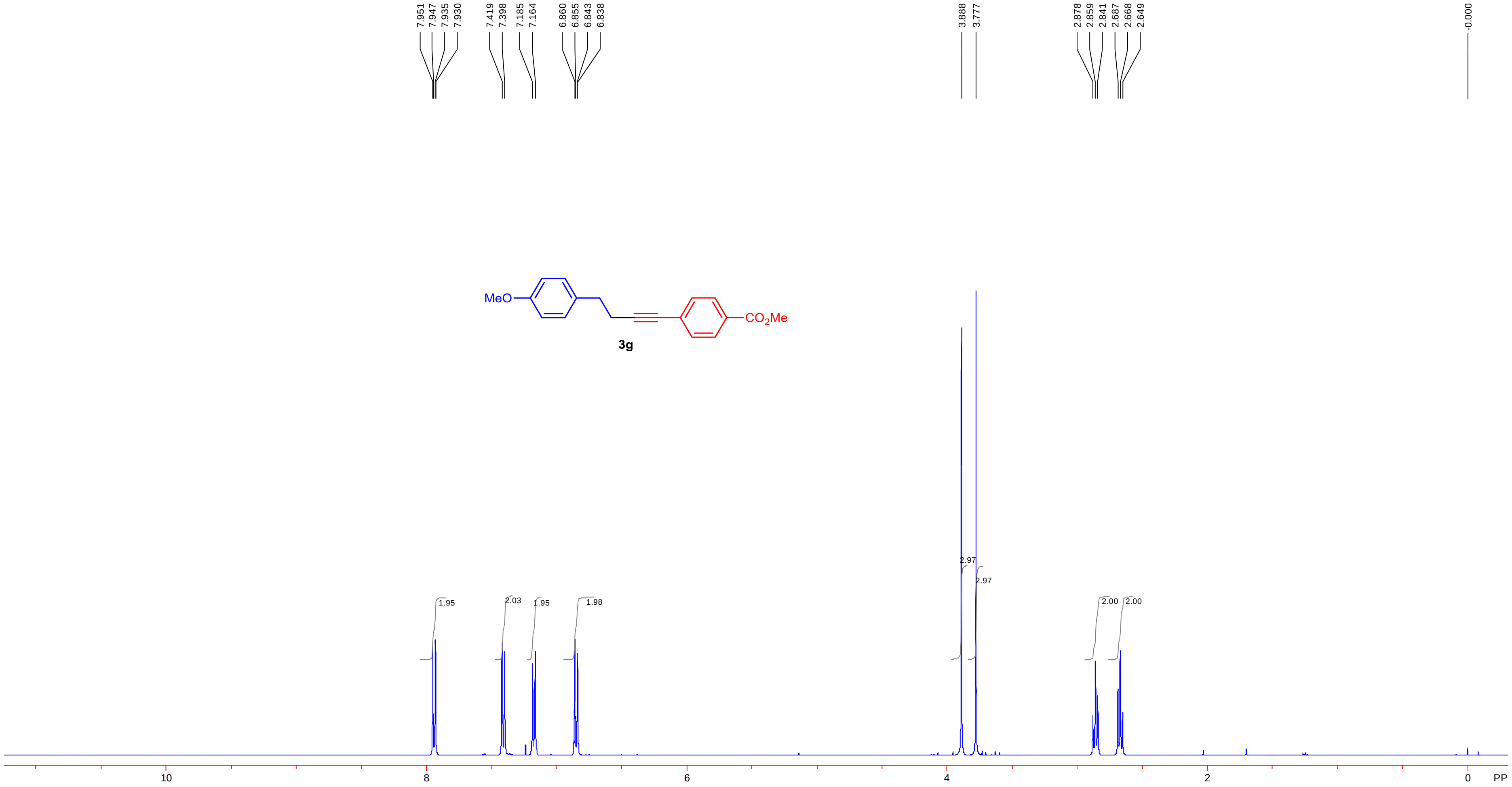

Supplementary Figure 66. ^{13}C NMR(100 MHz, $CDCl_3$)

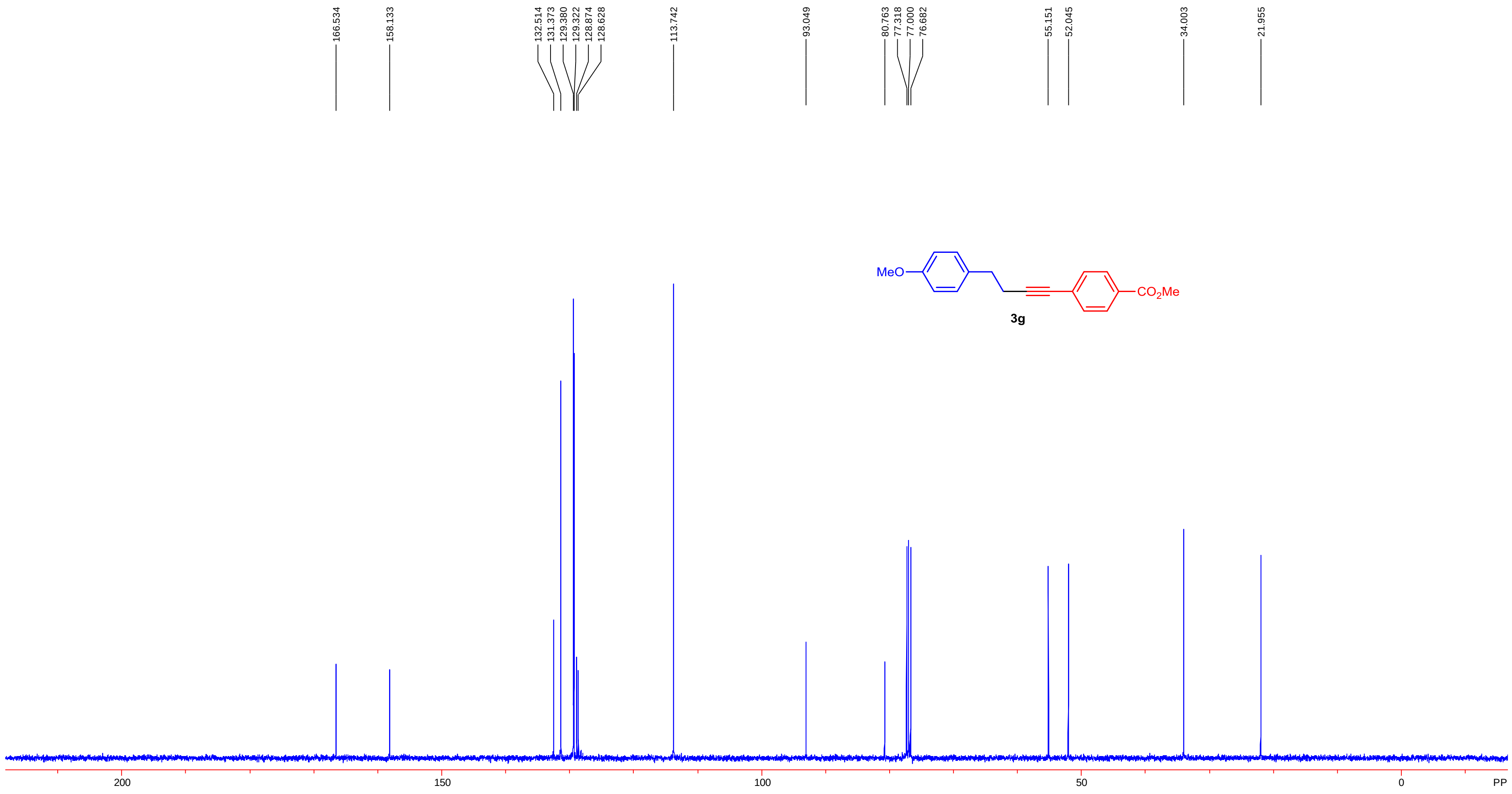

Supplementary Figure 67. ^{1}H NMR(400 MHz, $CDCl_3$)

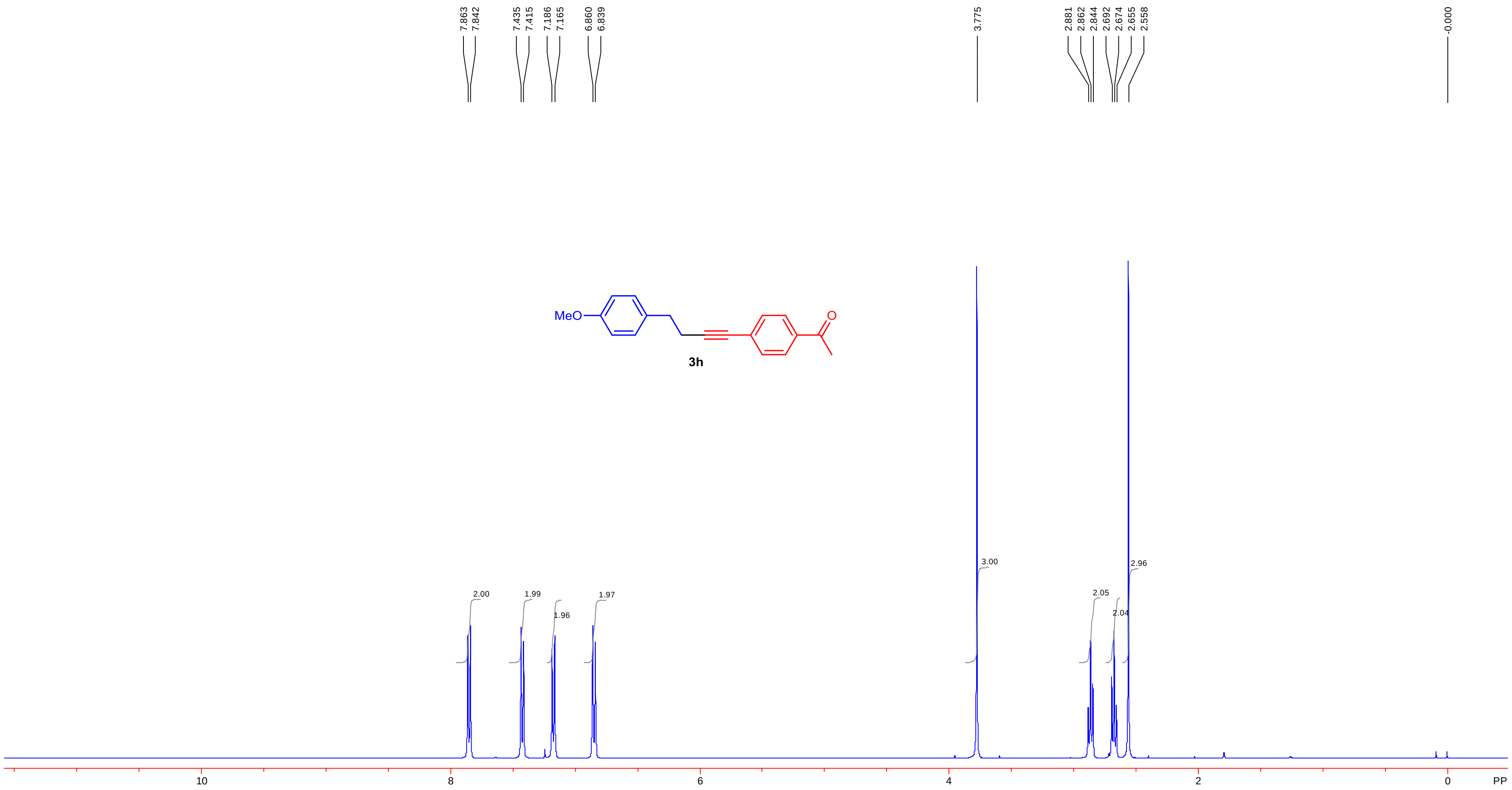

Supplementary Figure 68. ^{13}C NMR(100 MHz, $CDCl_3$)

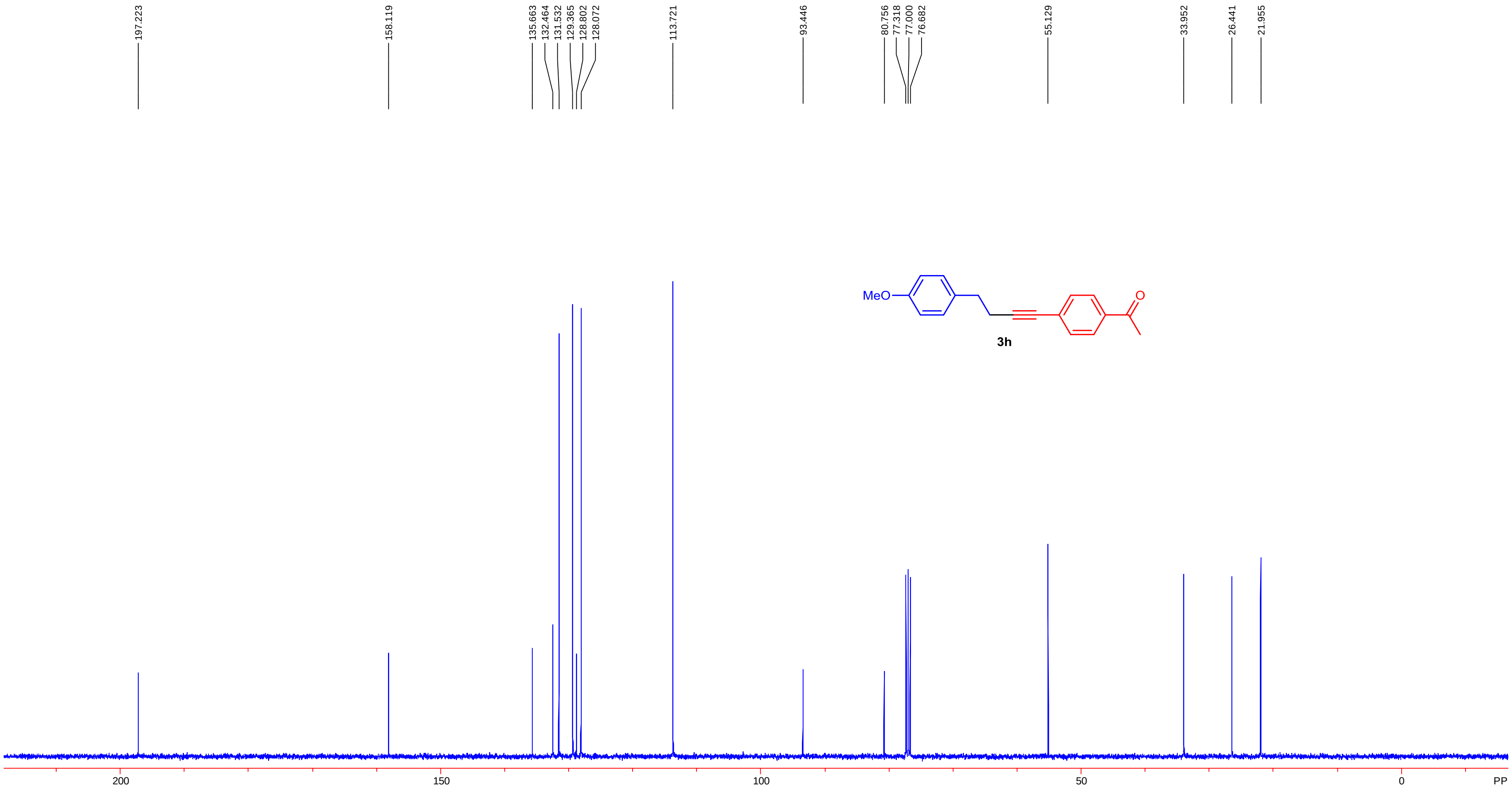

Supplementary Figure 69. ^{1}H NMR(400 MHz, $CDCl_3$)

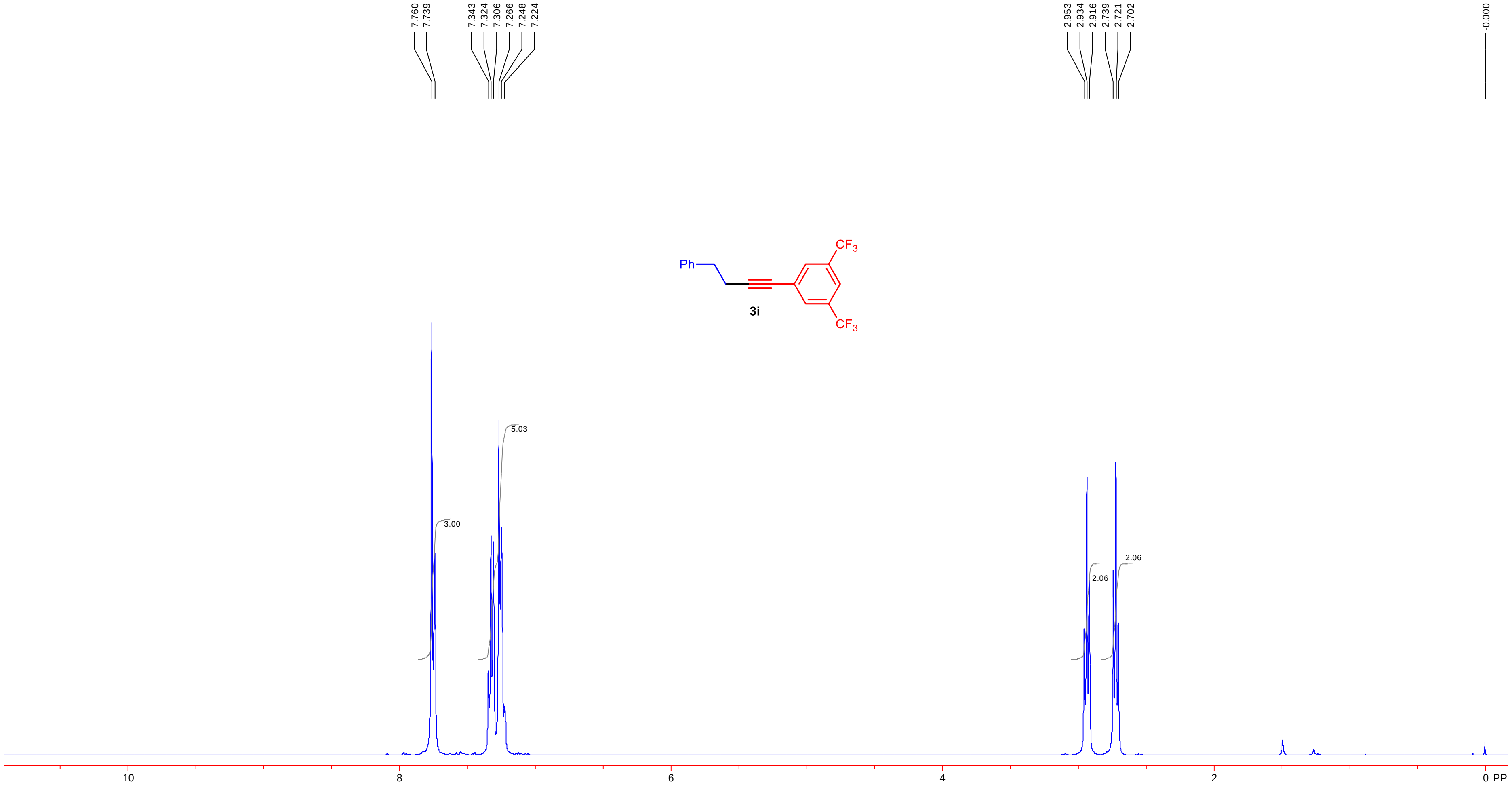

Supplementary Figure 70. ^{13}C NMR(100 MHz, $CDCl_3$)

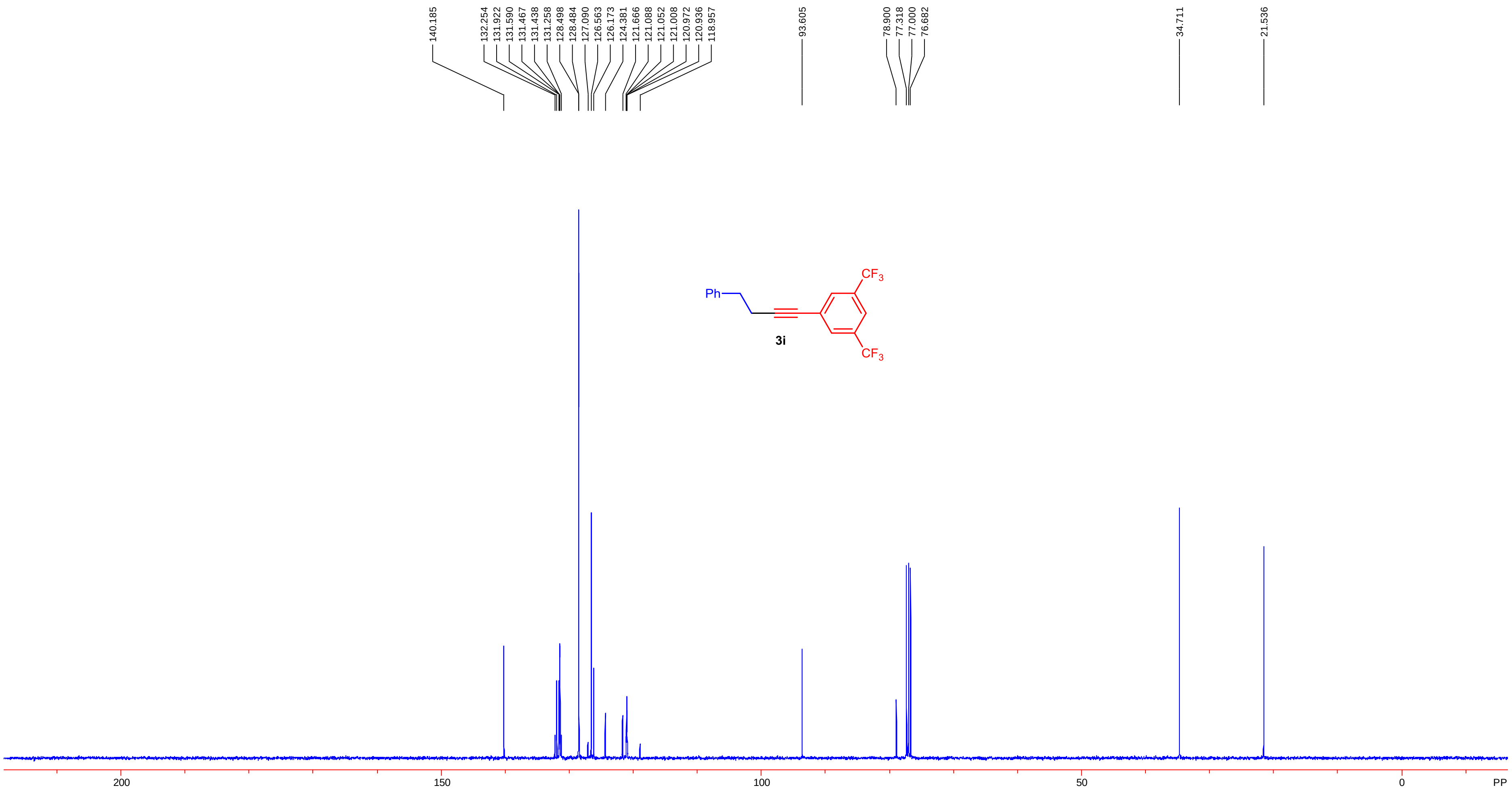

Supplementary Figure 71. ^{1}H NMR(400 MHz, $CDCl_3$)

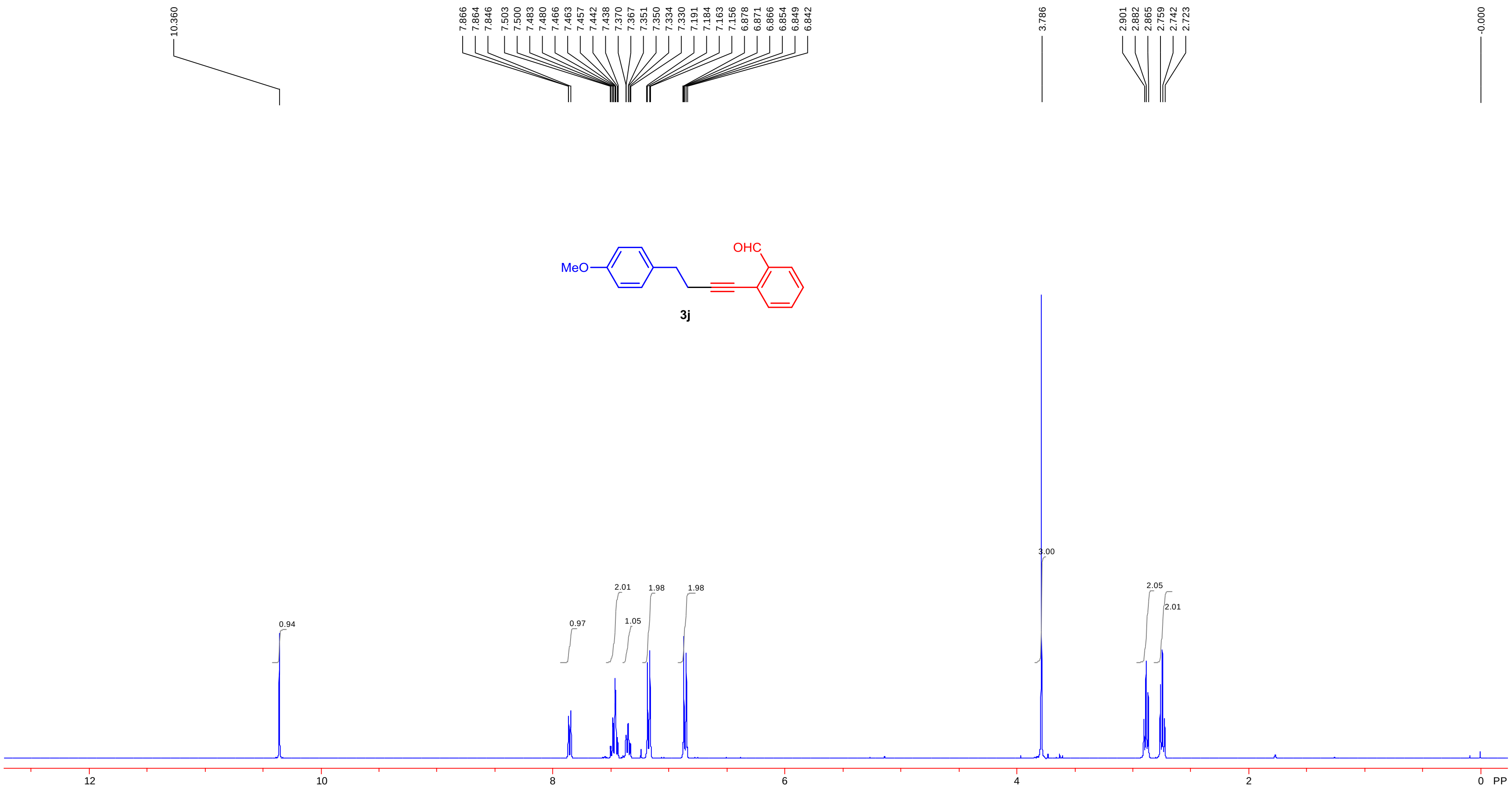

Supplementary Figure 72. ^{13}C NMR(100 MHz, $CDCl_3$)

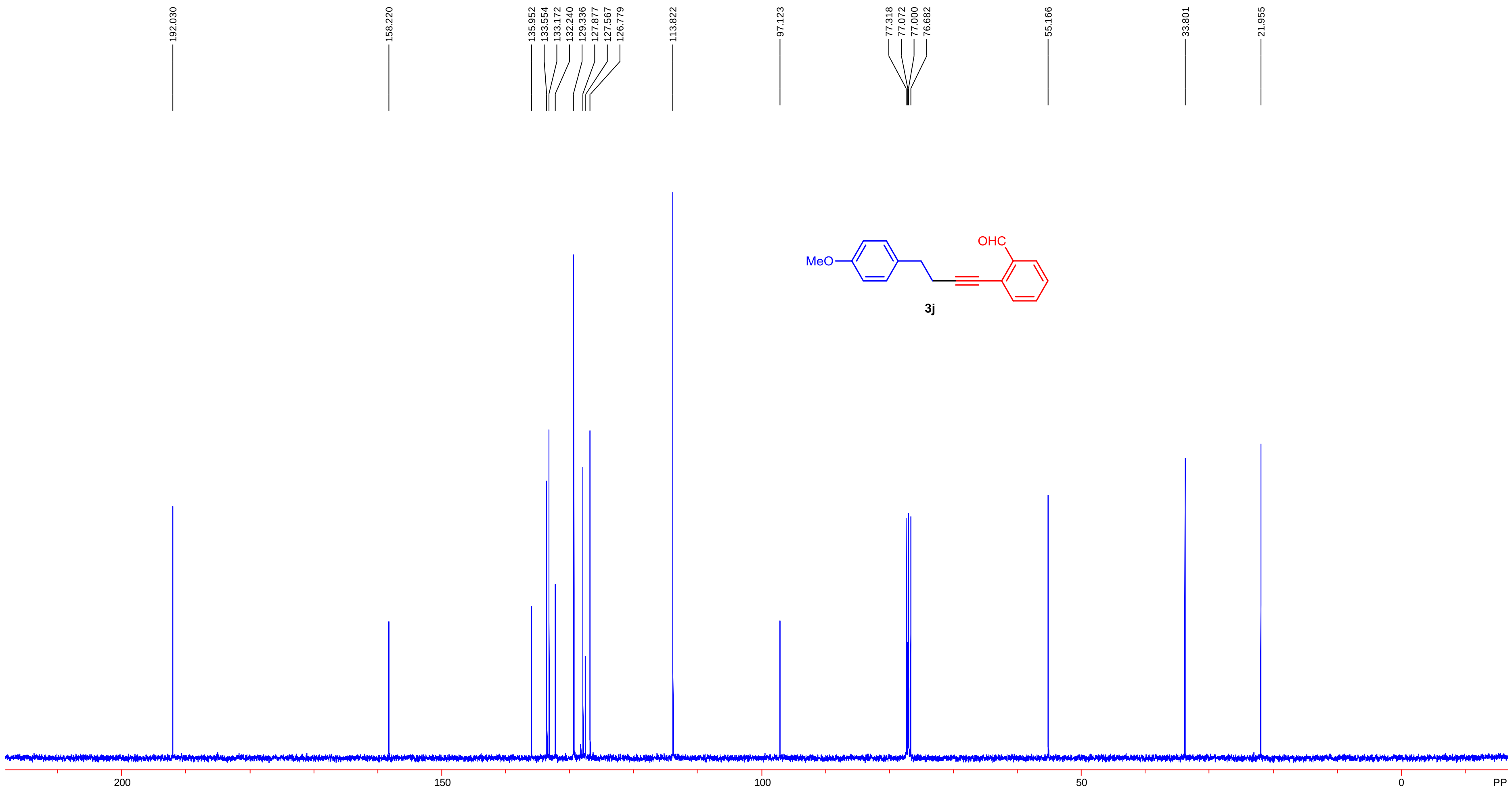

Supplementary Figure 73. ^{1}H NMR(400 MHz, $CDCl_3$)

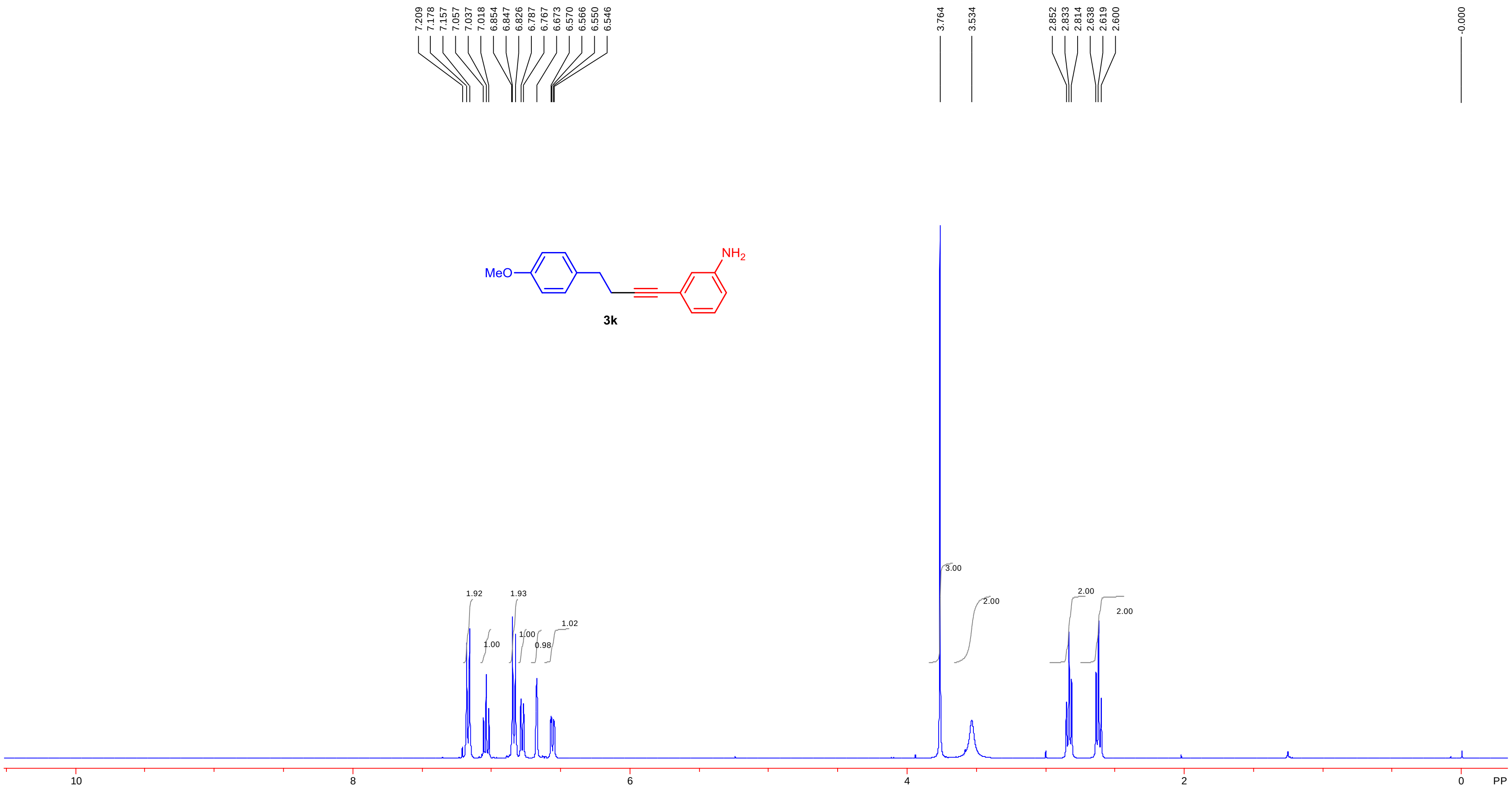

Supplementary Figure 74. ^{13}C NMR(100 MHz, $CDCl_3$)

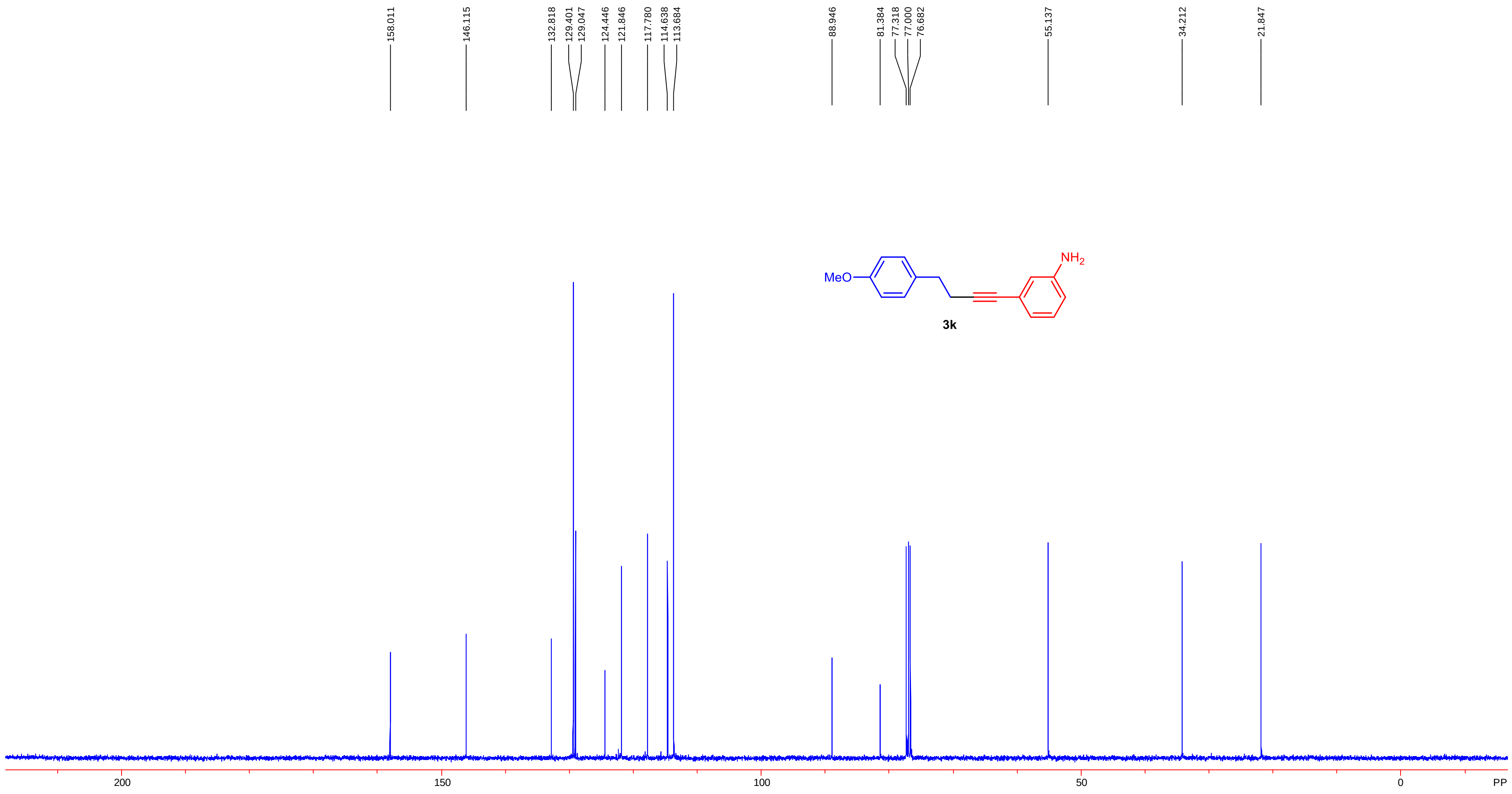

Supplementary Figure 75. ^{1}H NMR(400 MHz, $CDCl_3$)

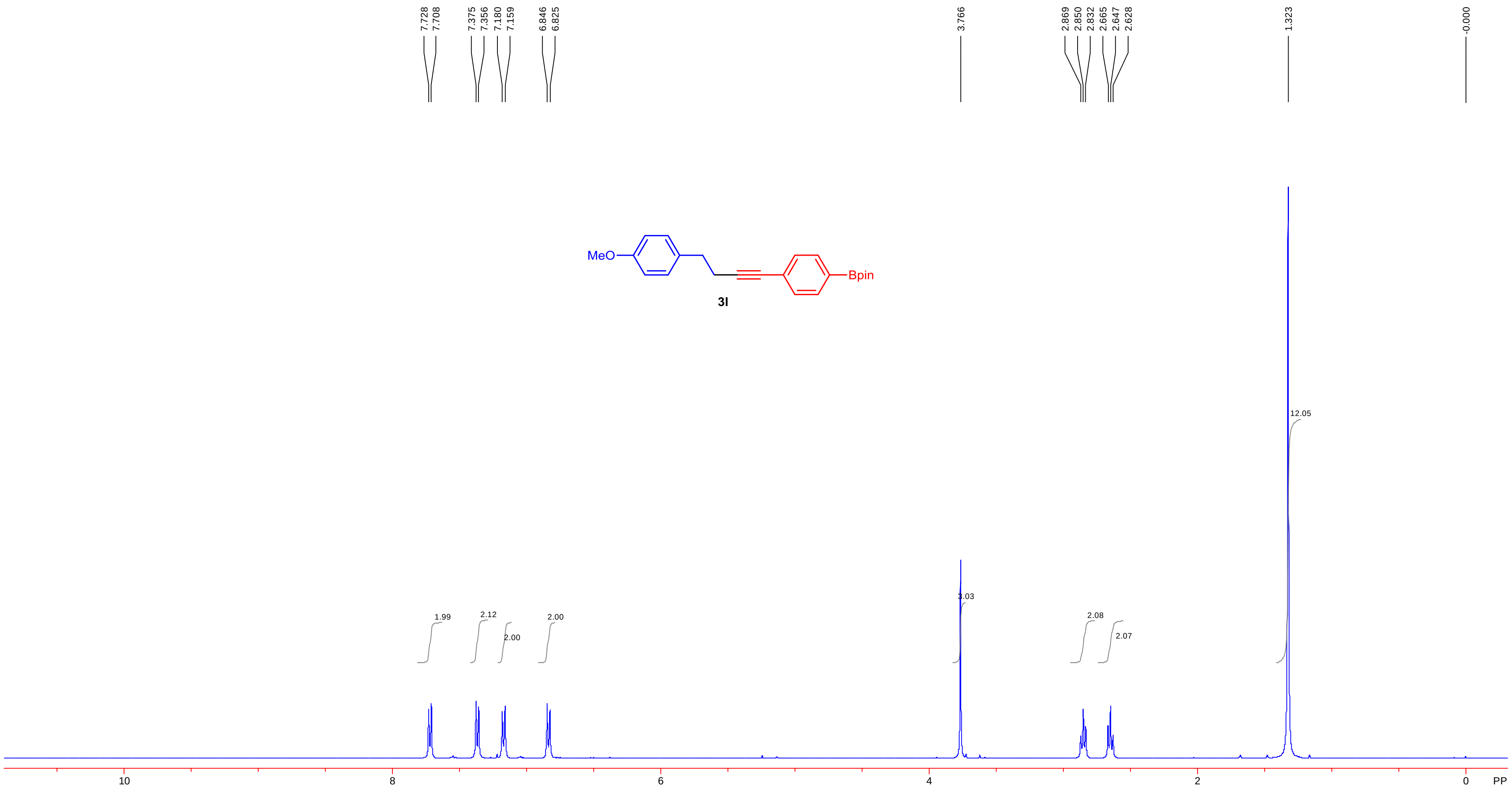

Supplementary Figure 76. ^{13}C NMR(100 MHz, $CDCl_3$)

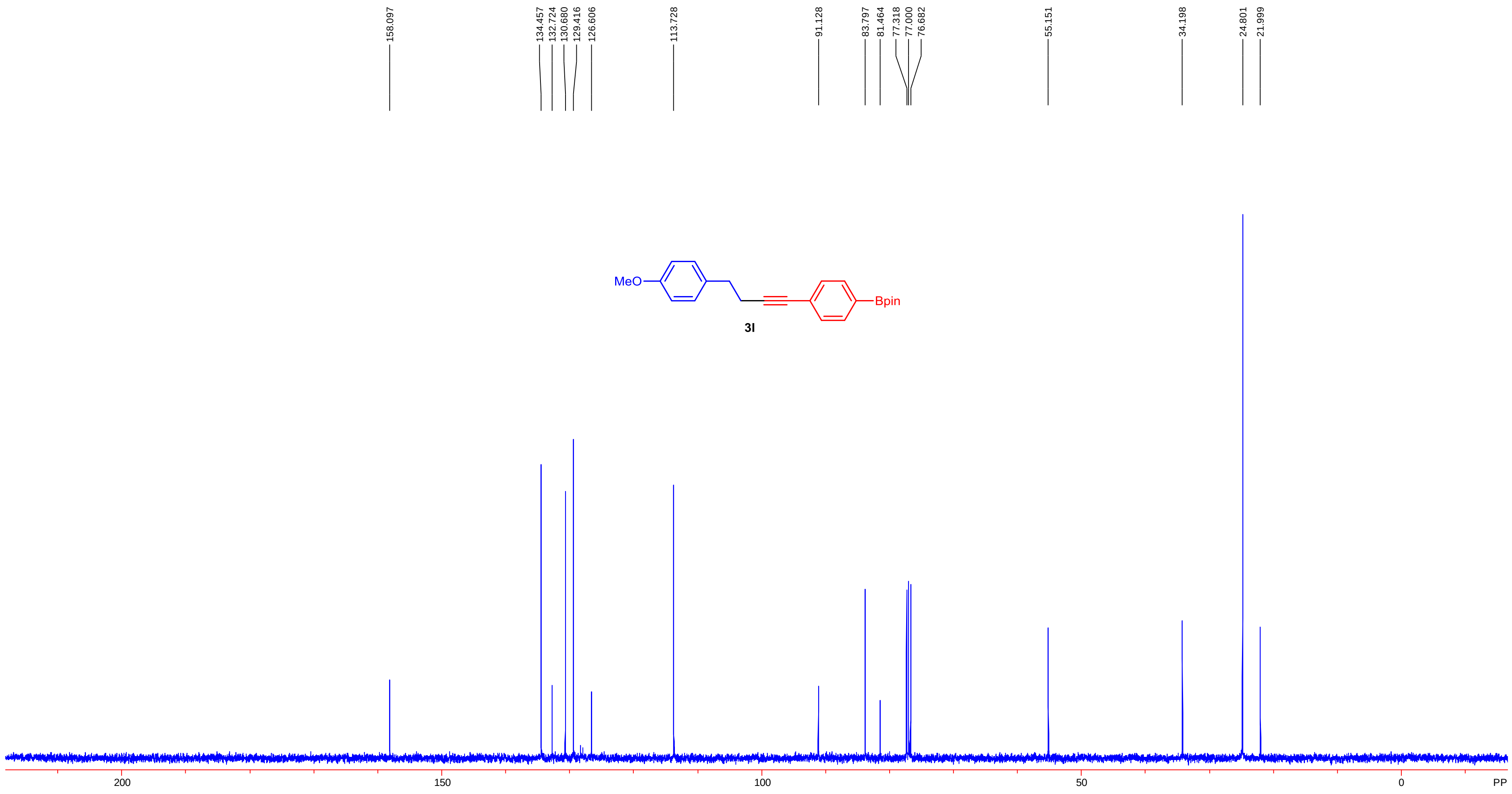

Supplementary Figure 77. ^{1}H NMR(400 MHz, $CDCl_3$)

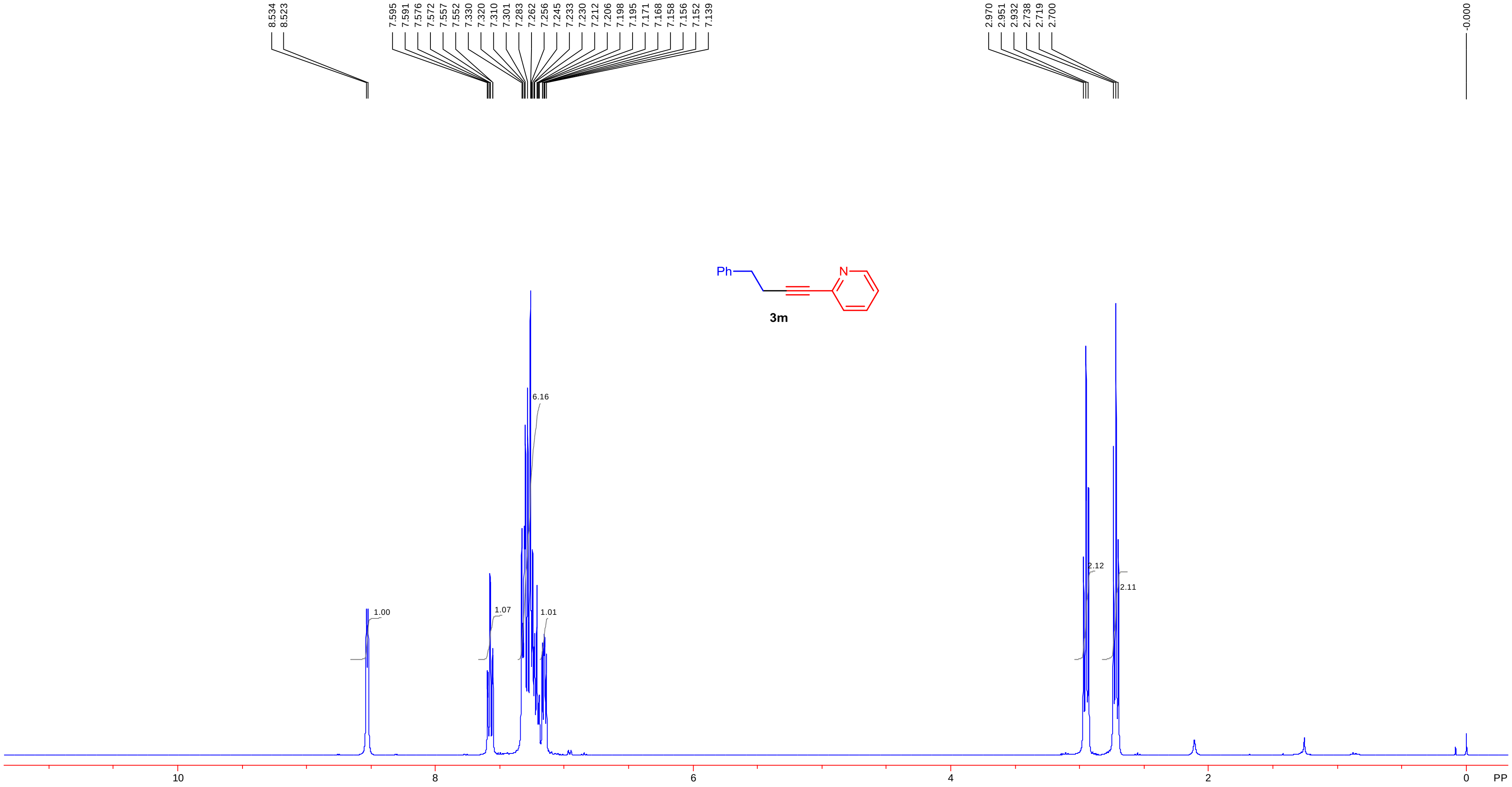

Supplementary Figure 78. ^{13}C NMR(100 MHz, $CDCl_3$)

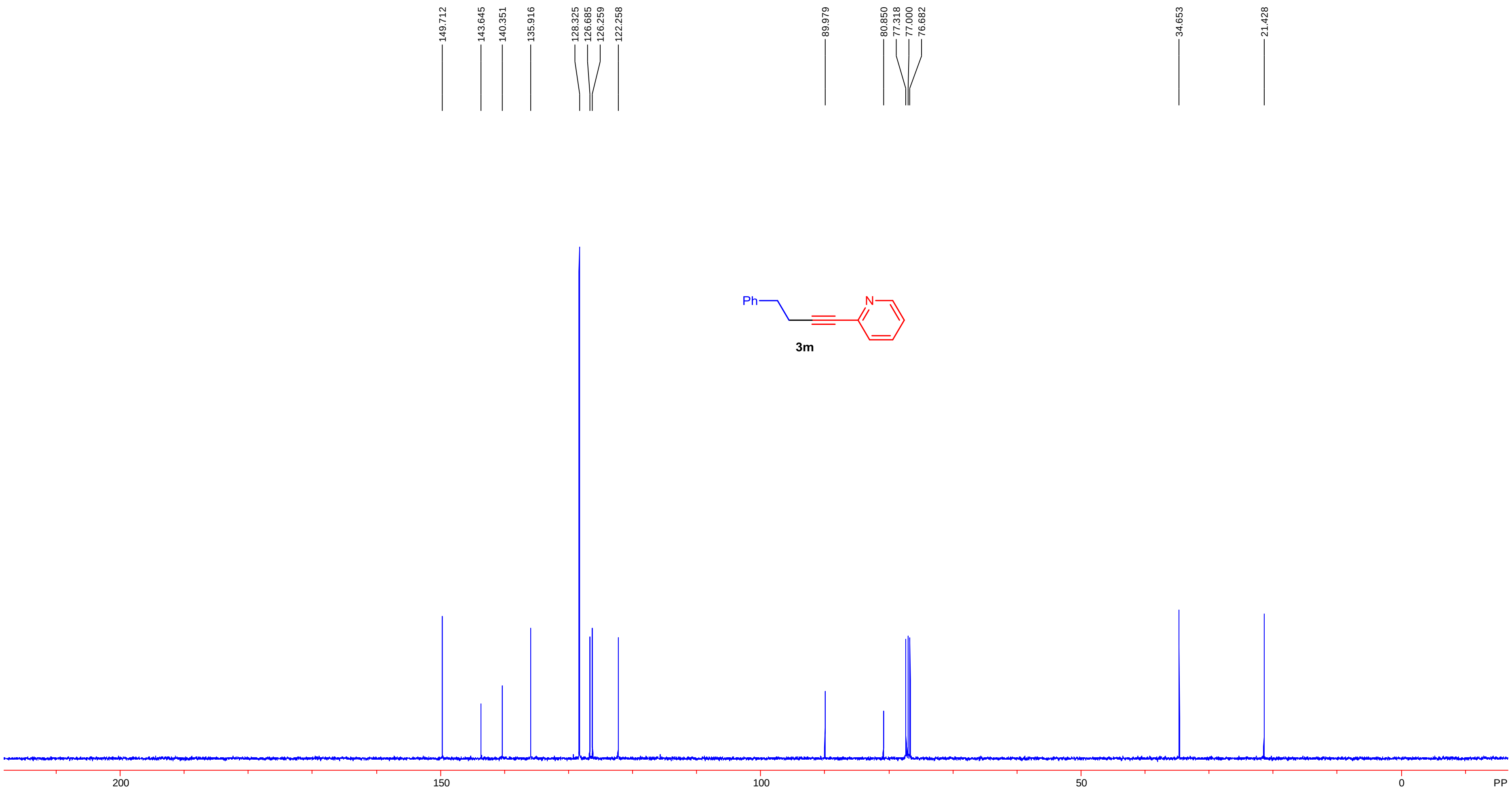

Supplementary Figure 79. ^{1}H NMR(400 MHz, $CDCl_3$)

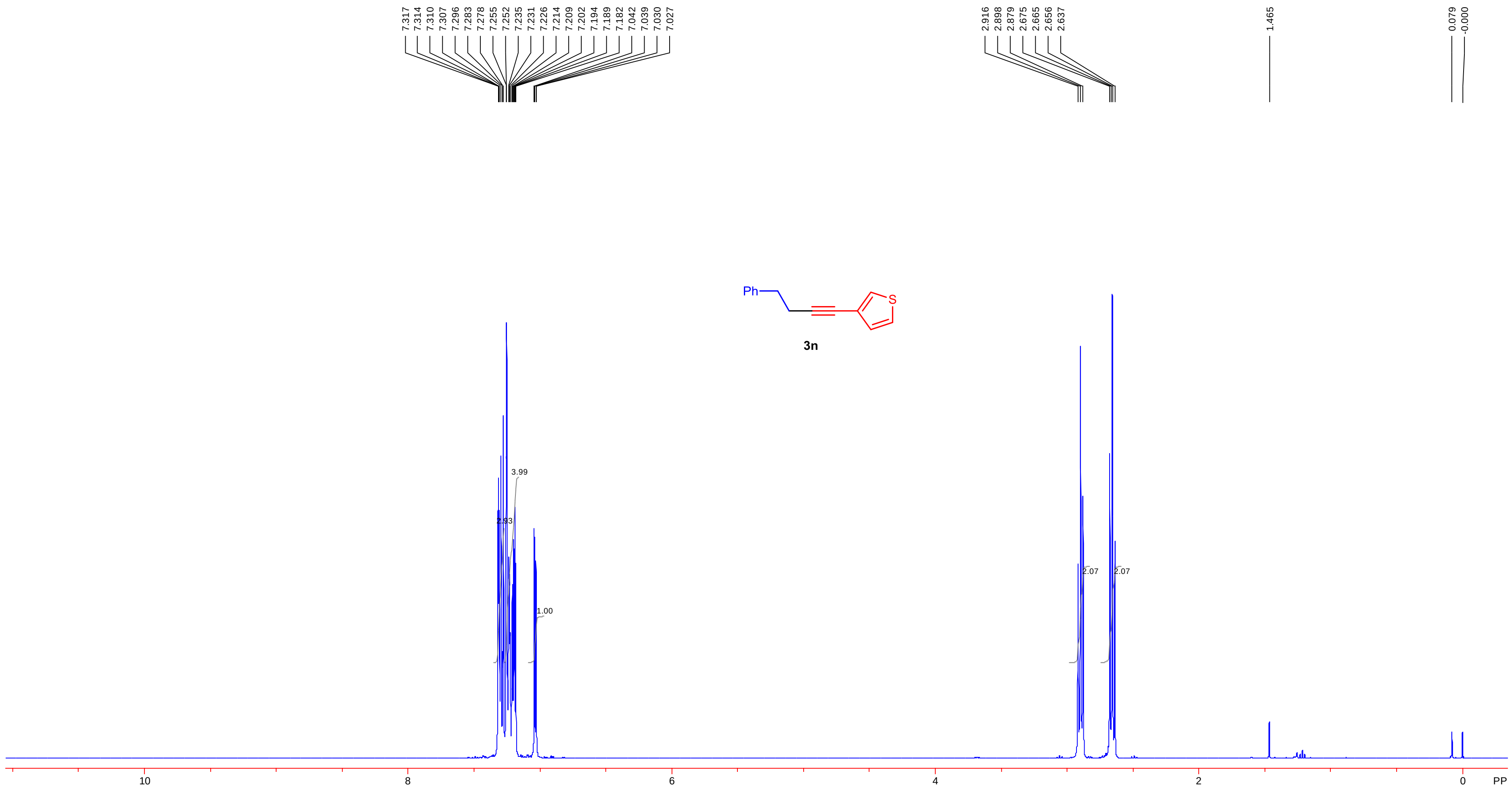

Supplementary Figure 80. ^{13}C NMR(100 MHz, $CDCl_3$)

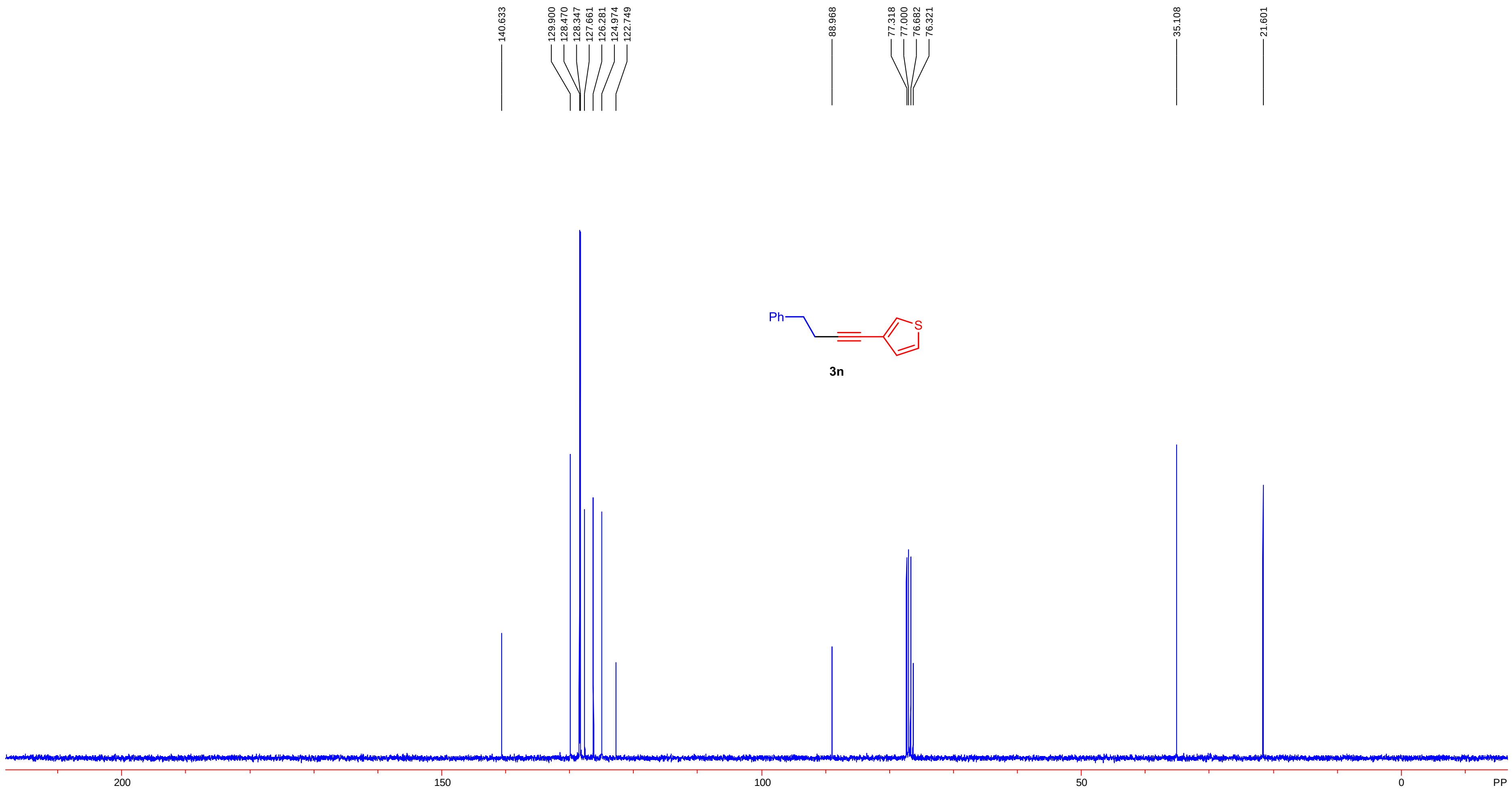

Supplementary Figure 81. ^{1}H NMR(400 MHz, $CDCl_3$)

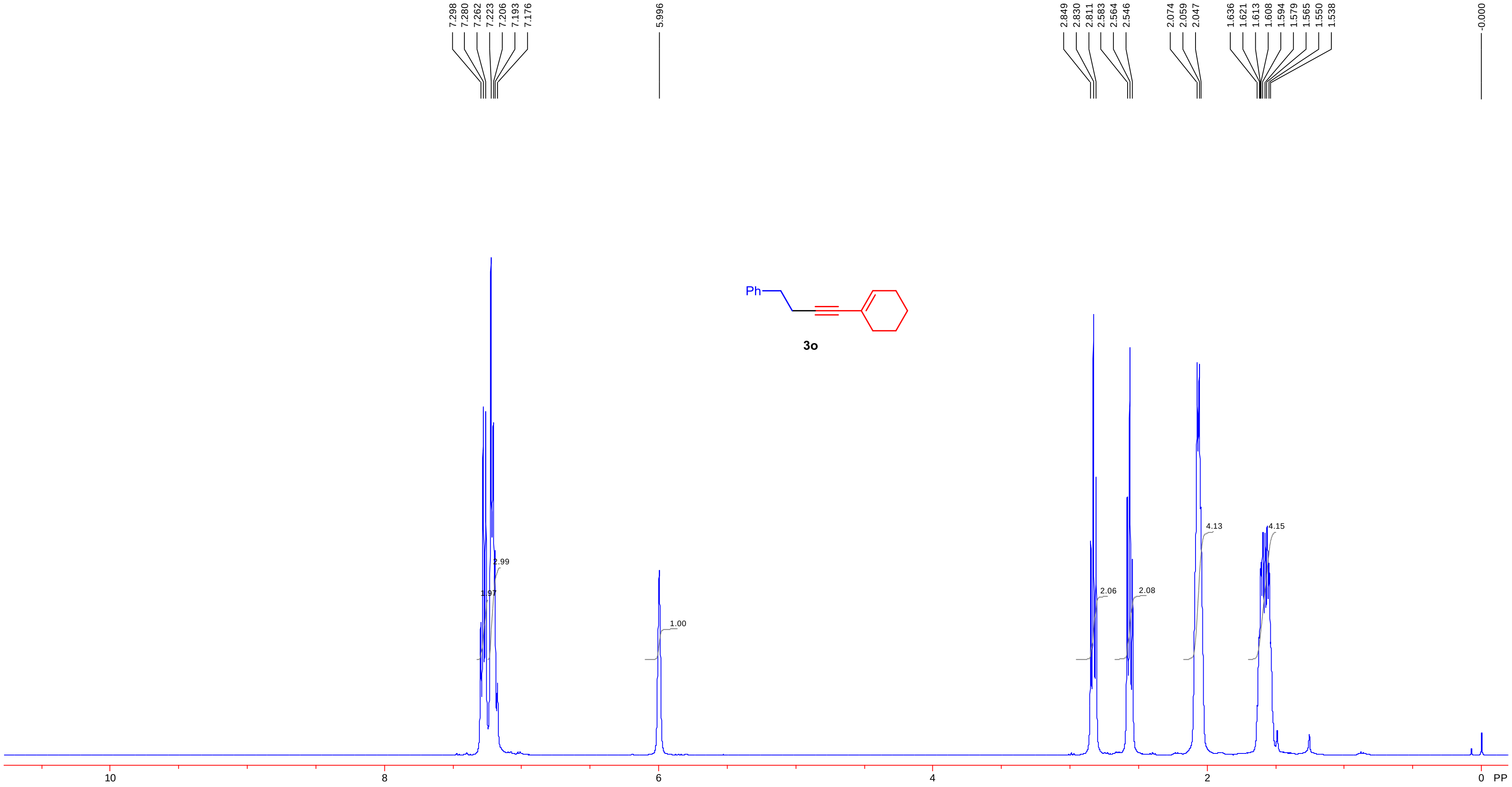

Supplementary Figure 82. ^{13}C NMR(100 MHz, $CDCl_3$)

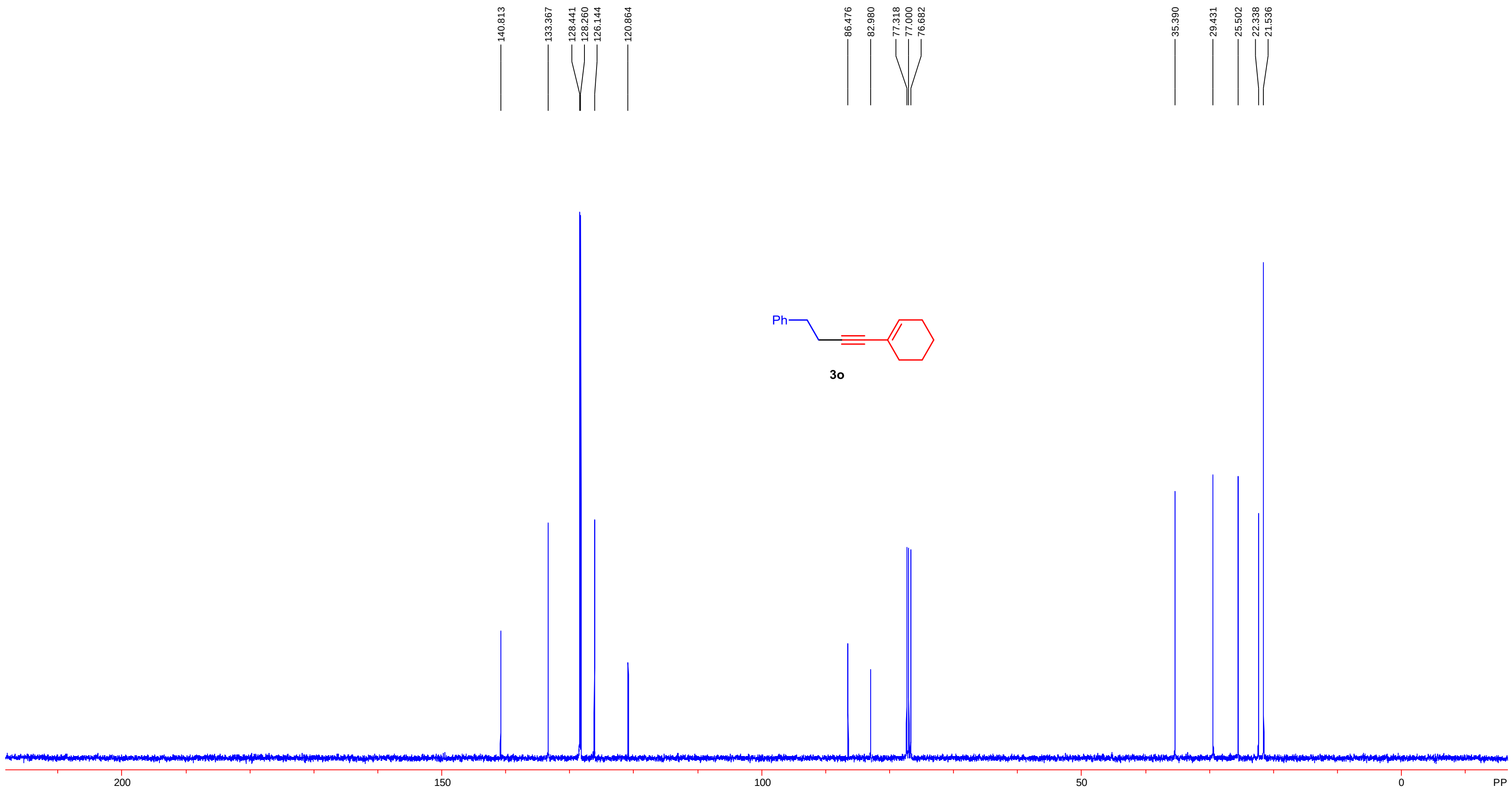

Supplementary Figure 83. ^{1}H NMR(400 MHz, $CDCl_3$)

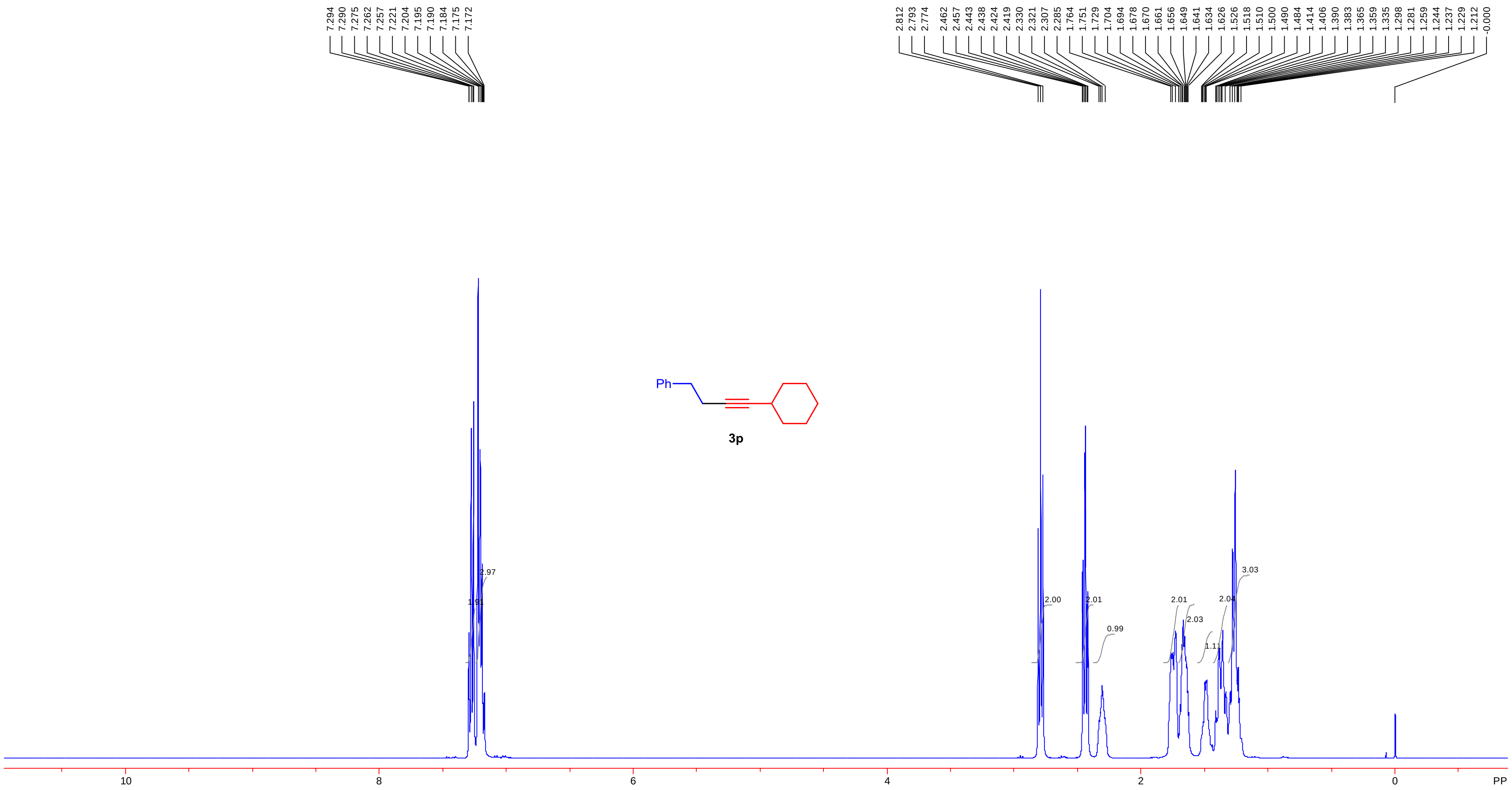

Supplementary Figure 84. ^{13}C NMR(100 MHz, $CDCl_3$)

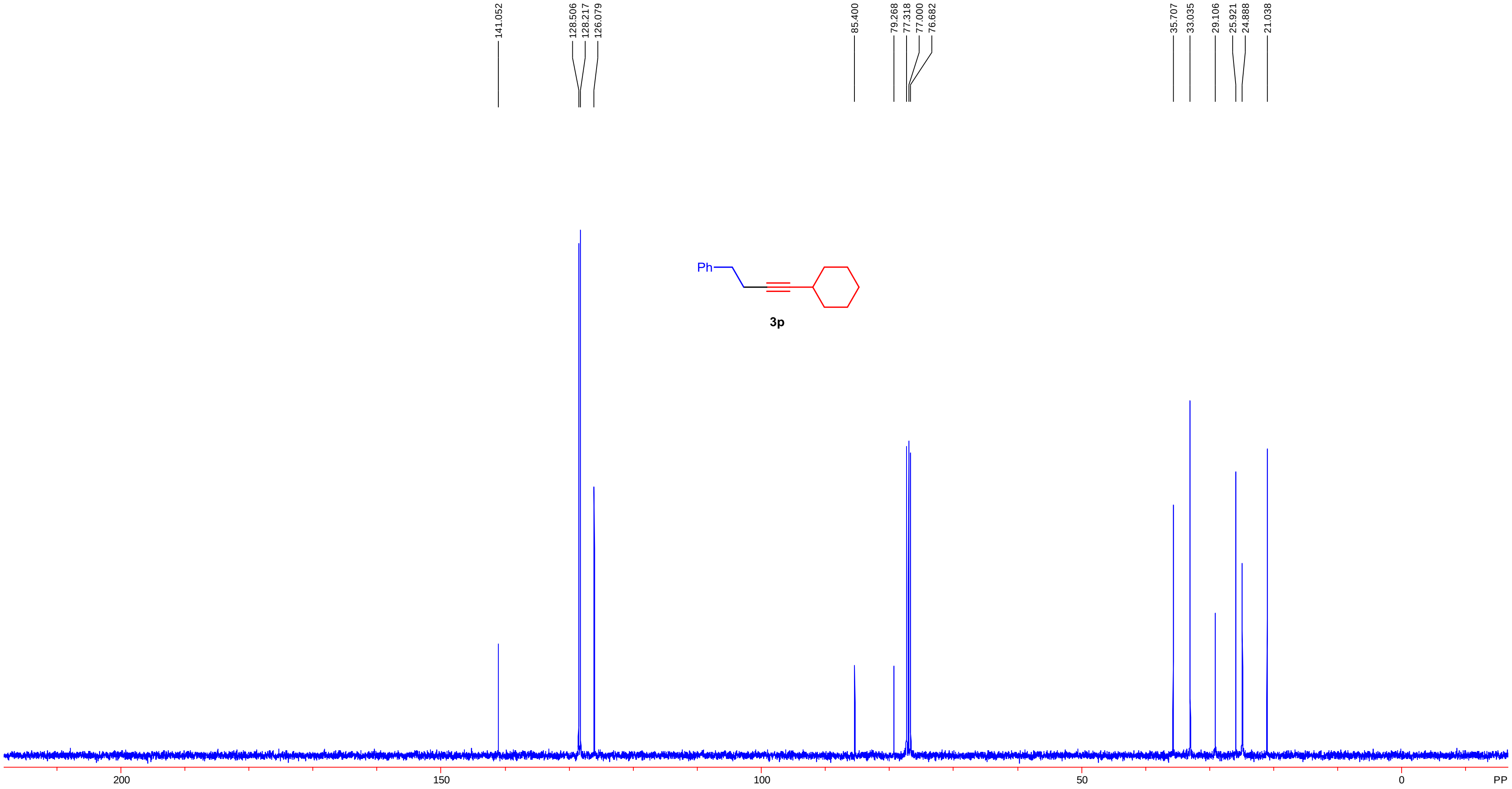

Supplementary Figure 85. ^{1}H NMR(400 MHz, $CDCl_3$)

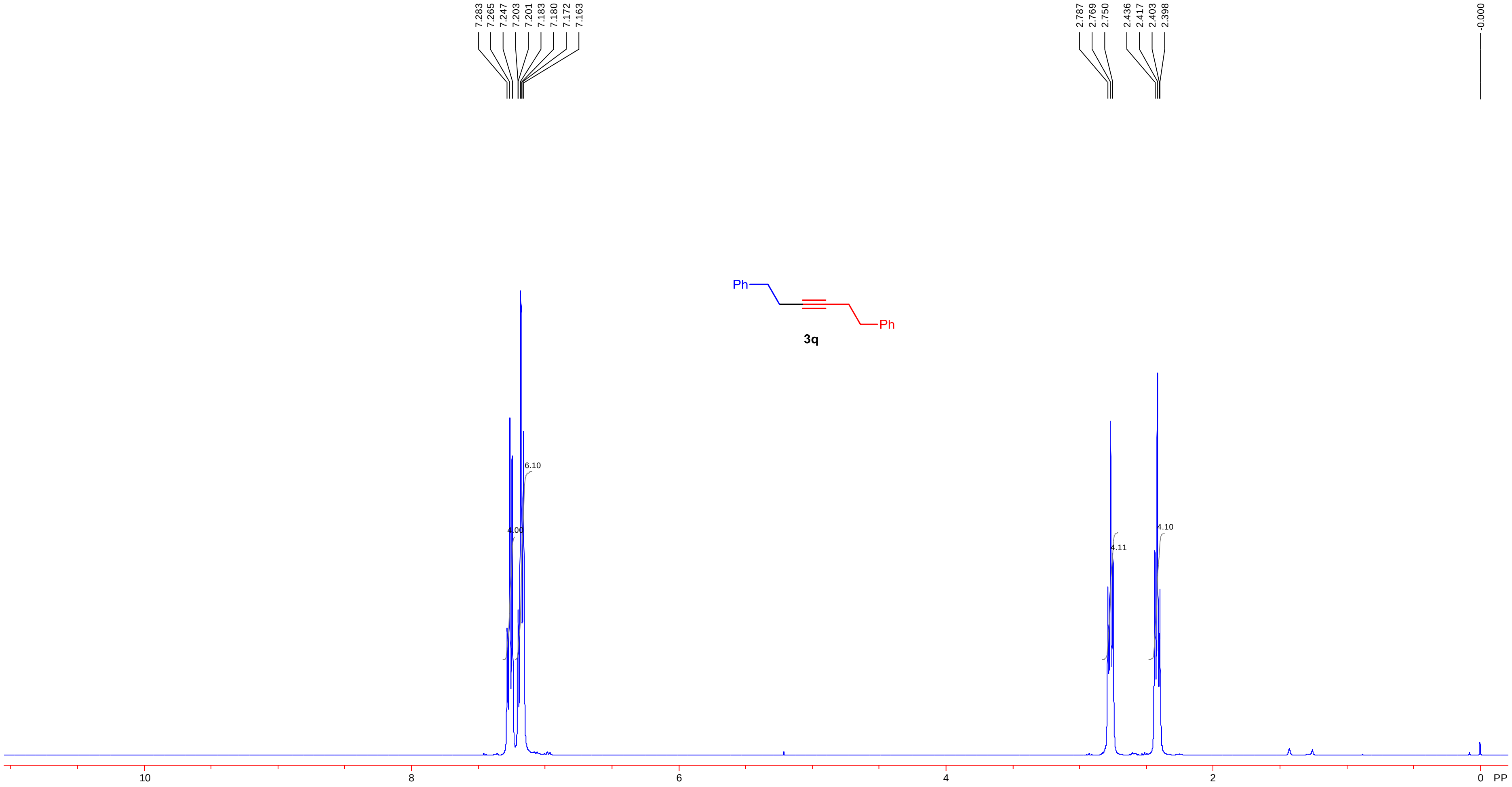

Supplementary Figure 86. ^{13}C NMR(100 MHz, $CDCl_3$)

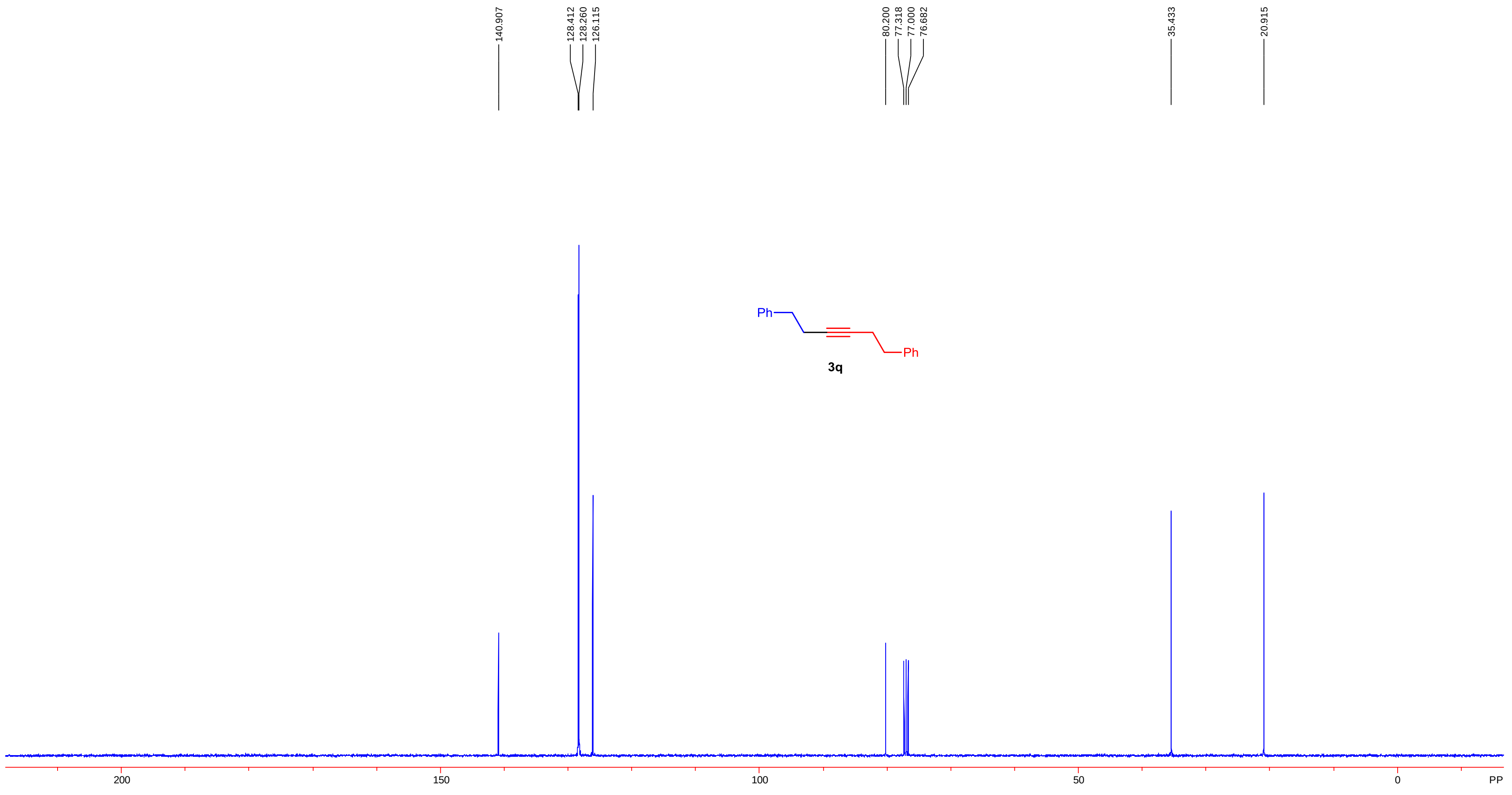

Supplementary Figure 87. ^{1}H NMR(600 MHz, $CDCl_3$)

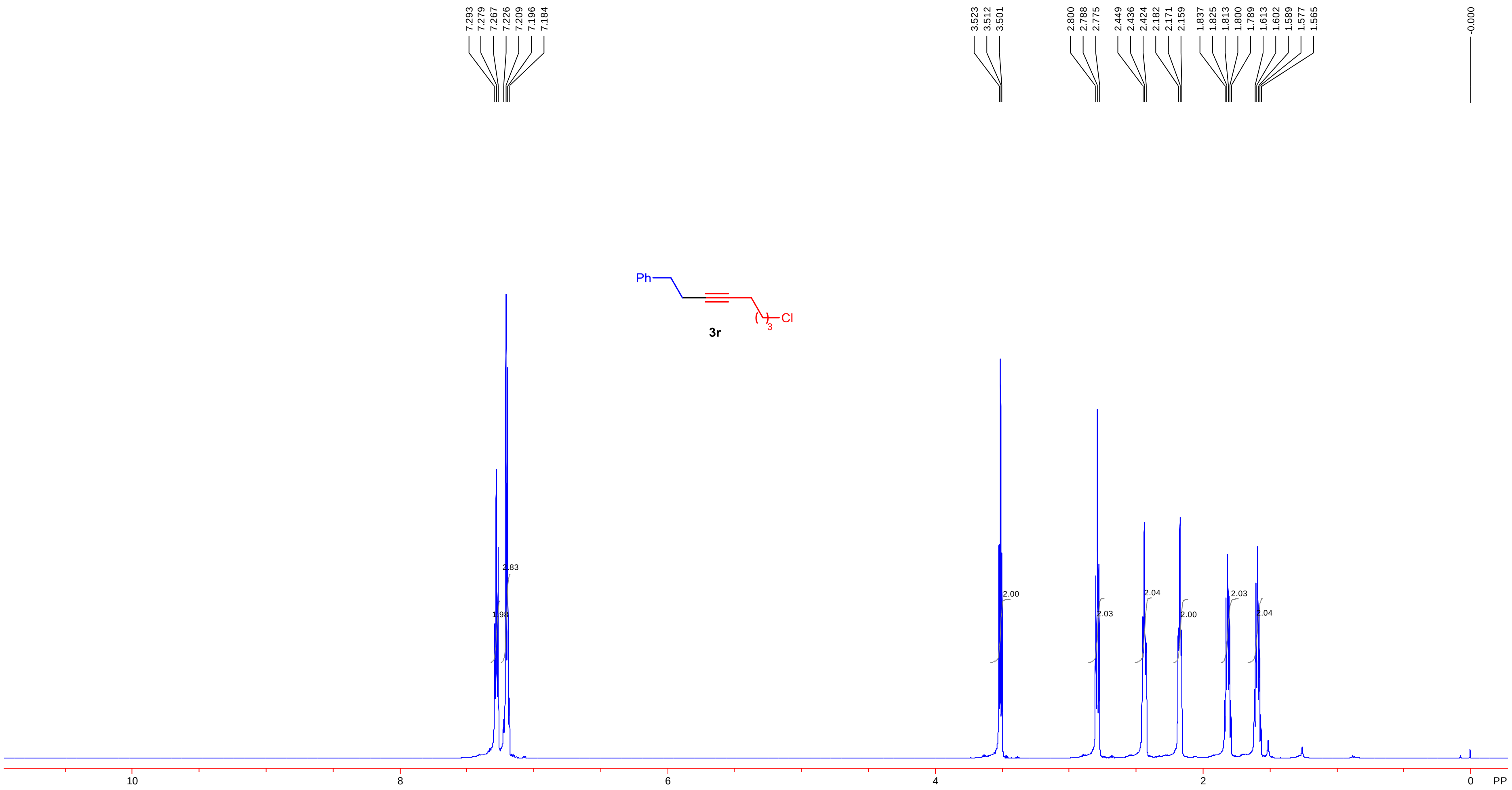

Supplementary Figure 88. ^{13}C NMR(151 MHz, $CDCl_3$)

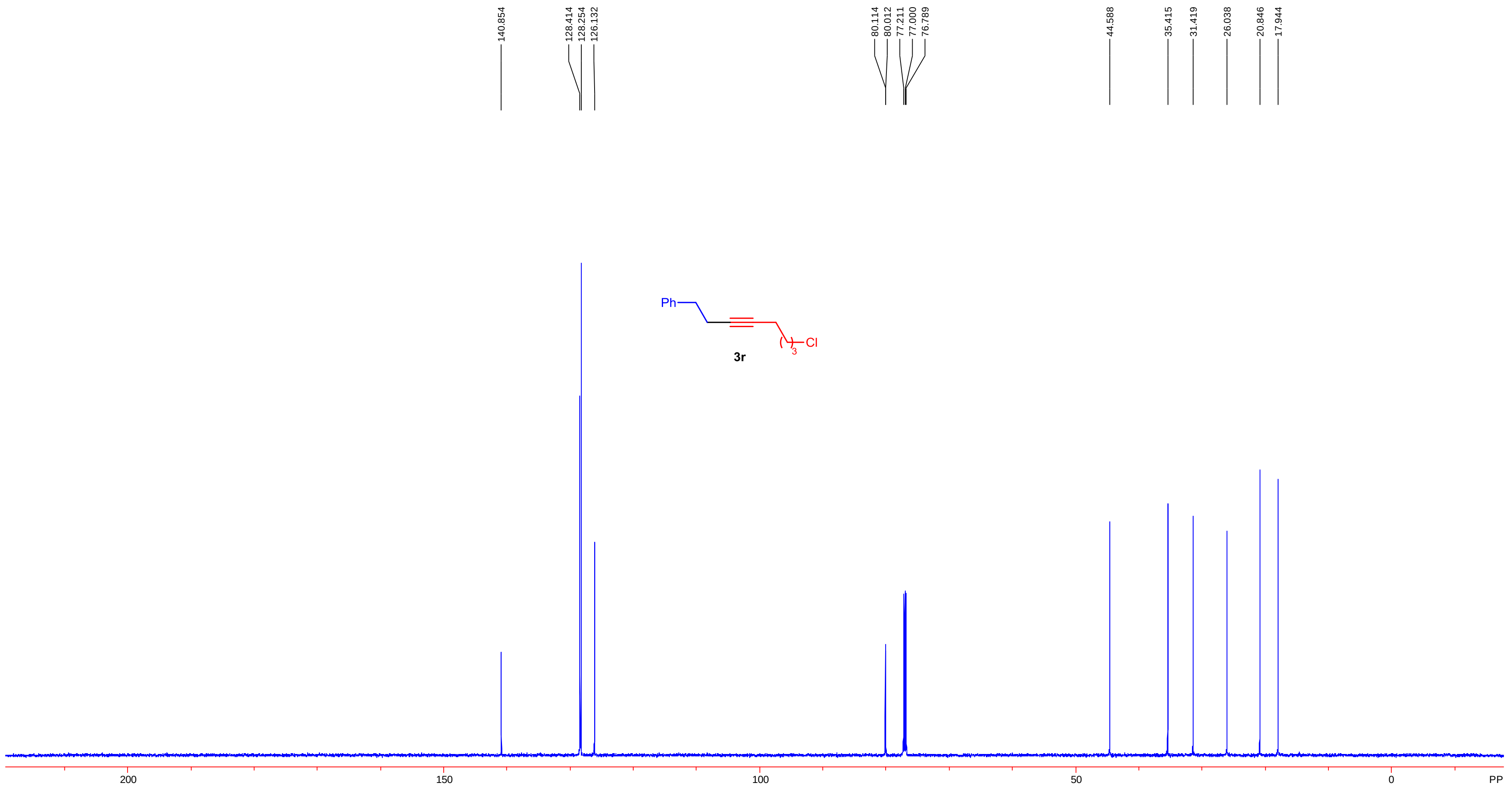

Supplementary Figure 89. ^{1}H NMR(400 MHz, $CDCl_3$)

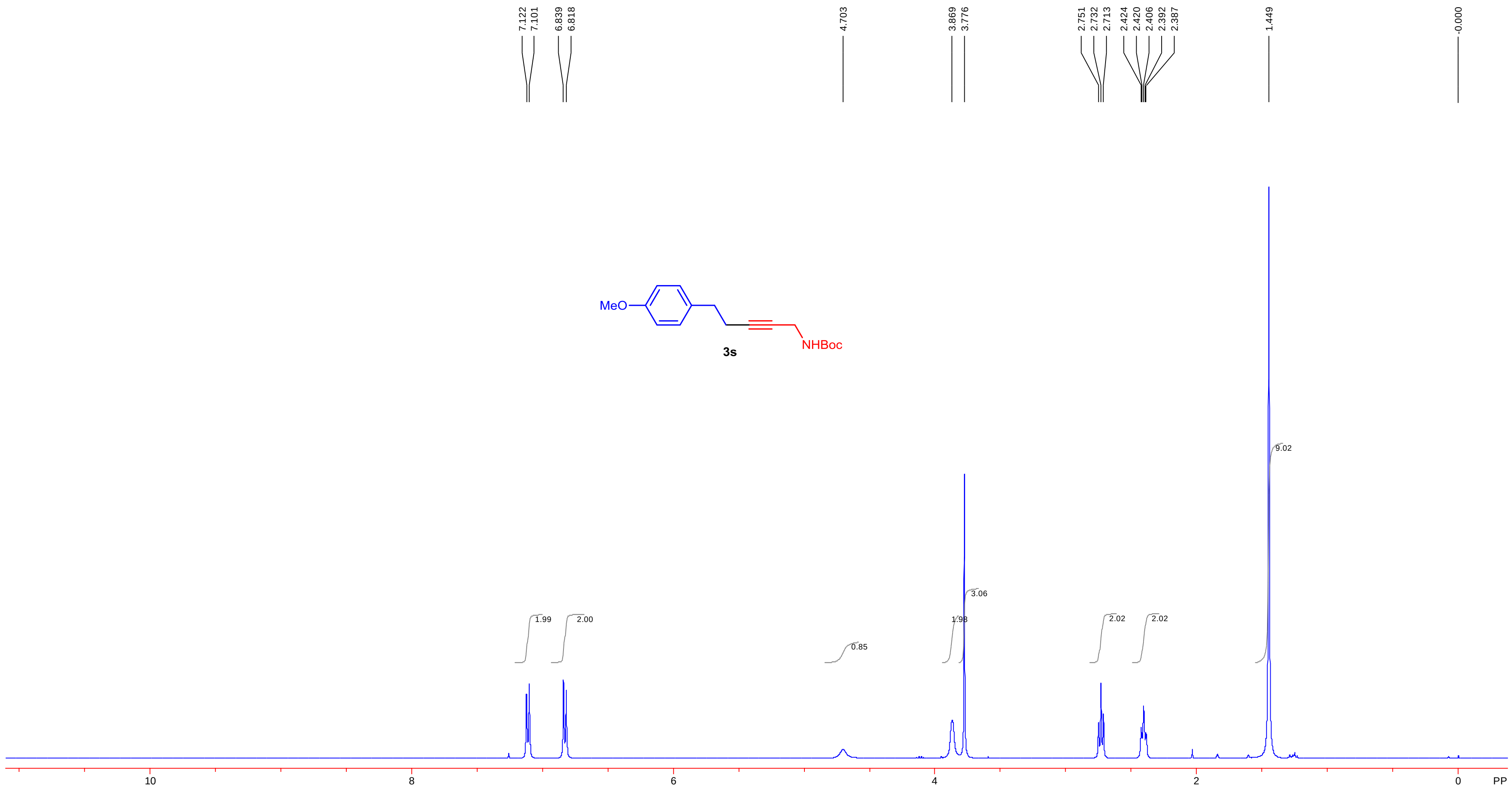

Supplementary Figure 90. ^{13}C NMR(100 MHz, $CDCl_3$)

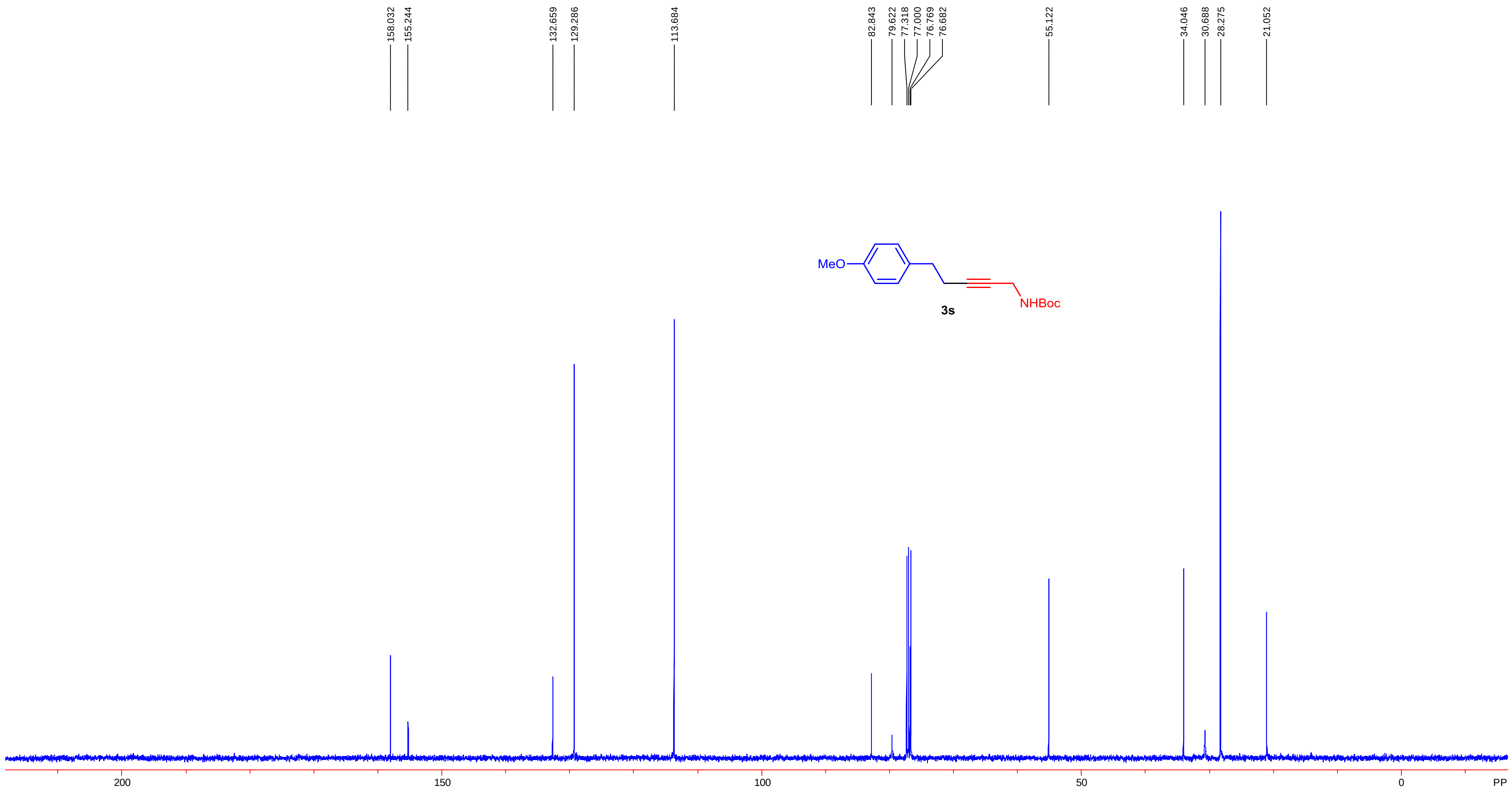

Supplementary Figure 91. ^{1}H NMR(400 MHz, $CDCl_3$)

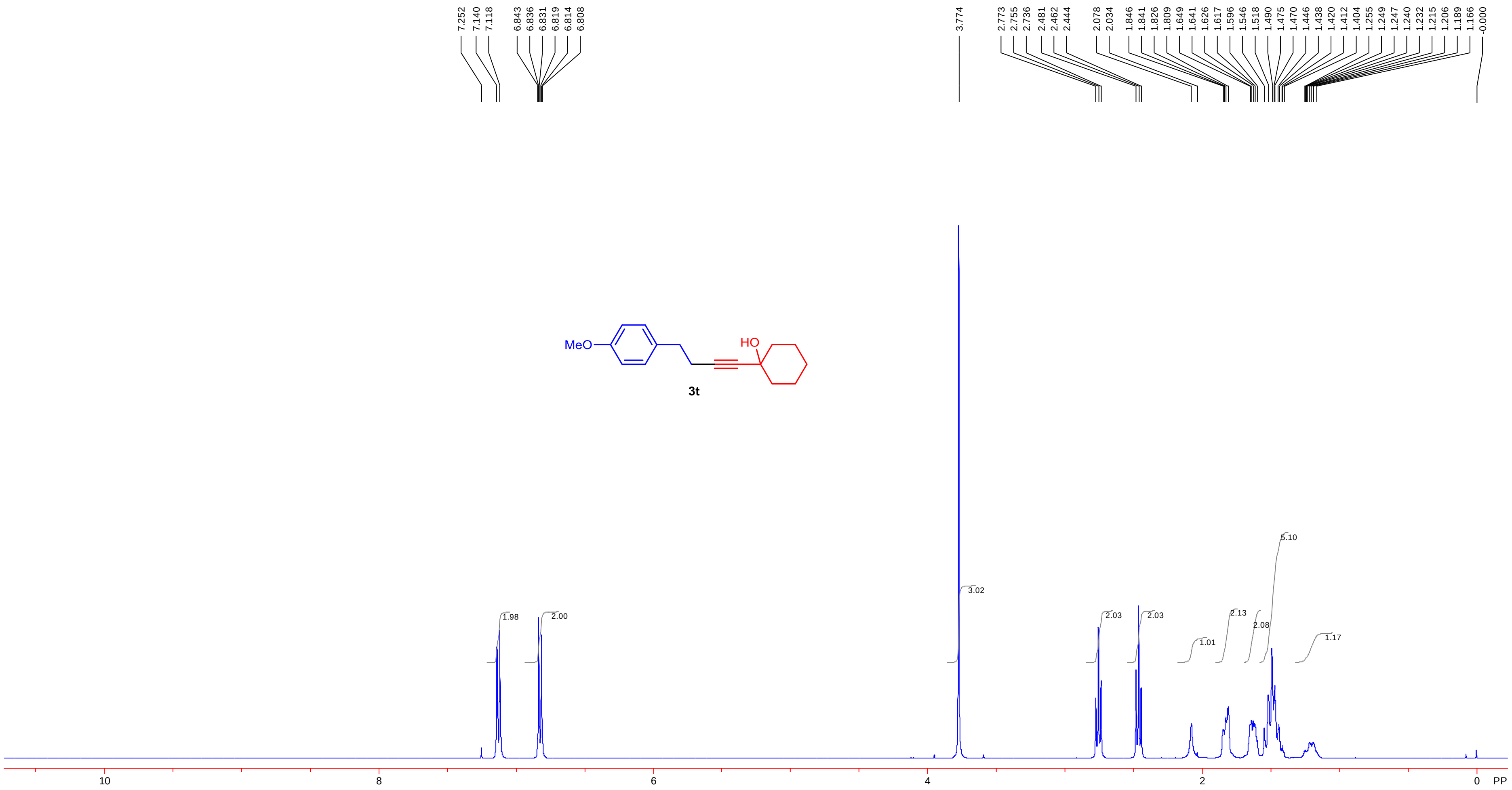

Supplementary Figure 92. ^{13}C NMR(100 MHz, $CDCl_3$)

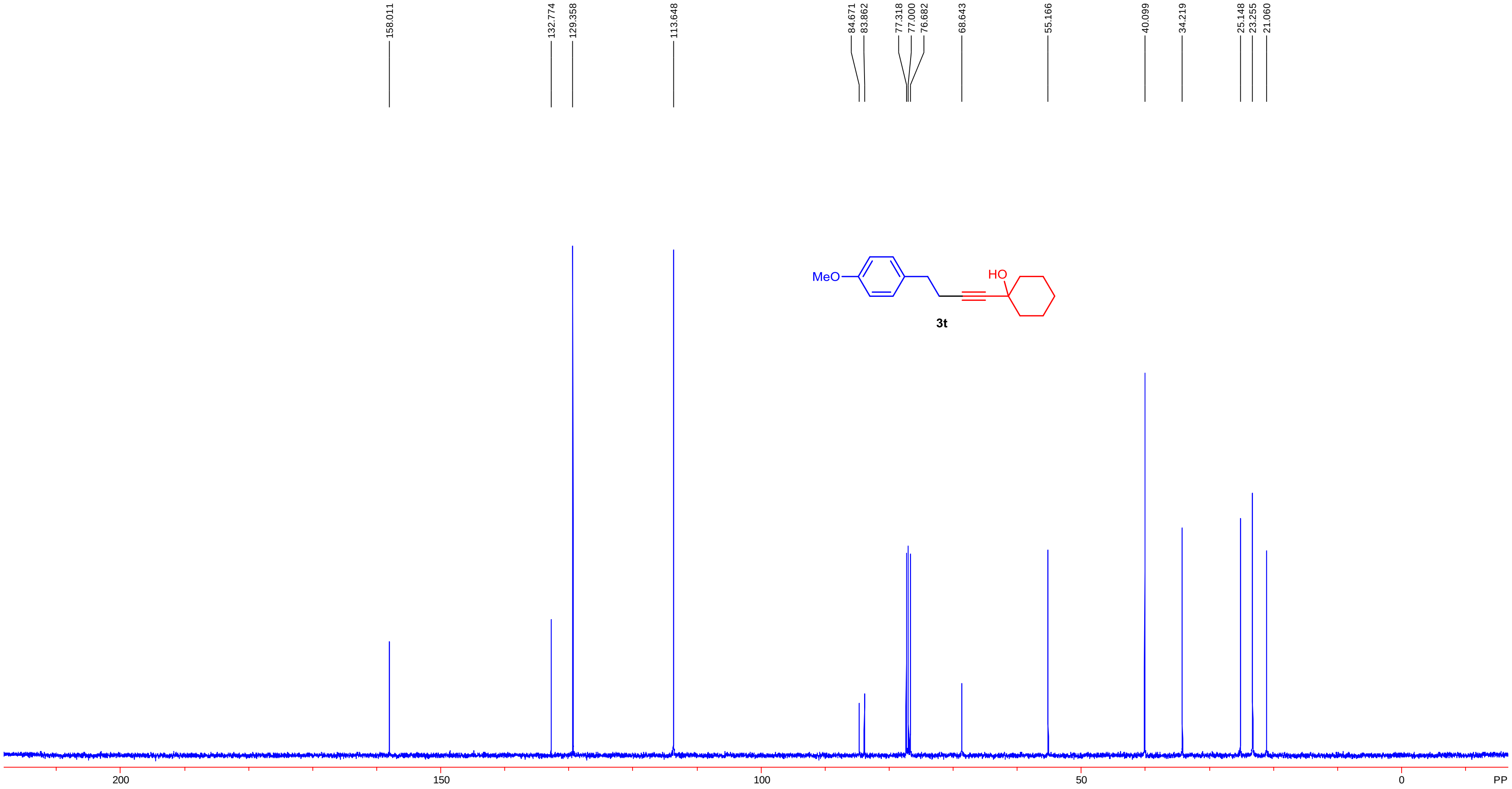

Supplementary Figure 93. ^{1}H NMR(400 MHz, $CDCl_3$)

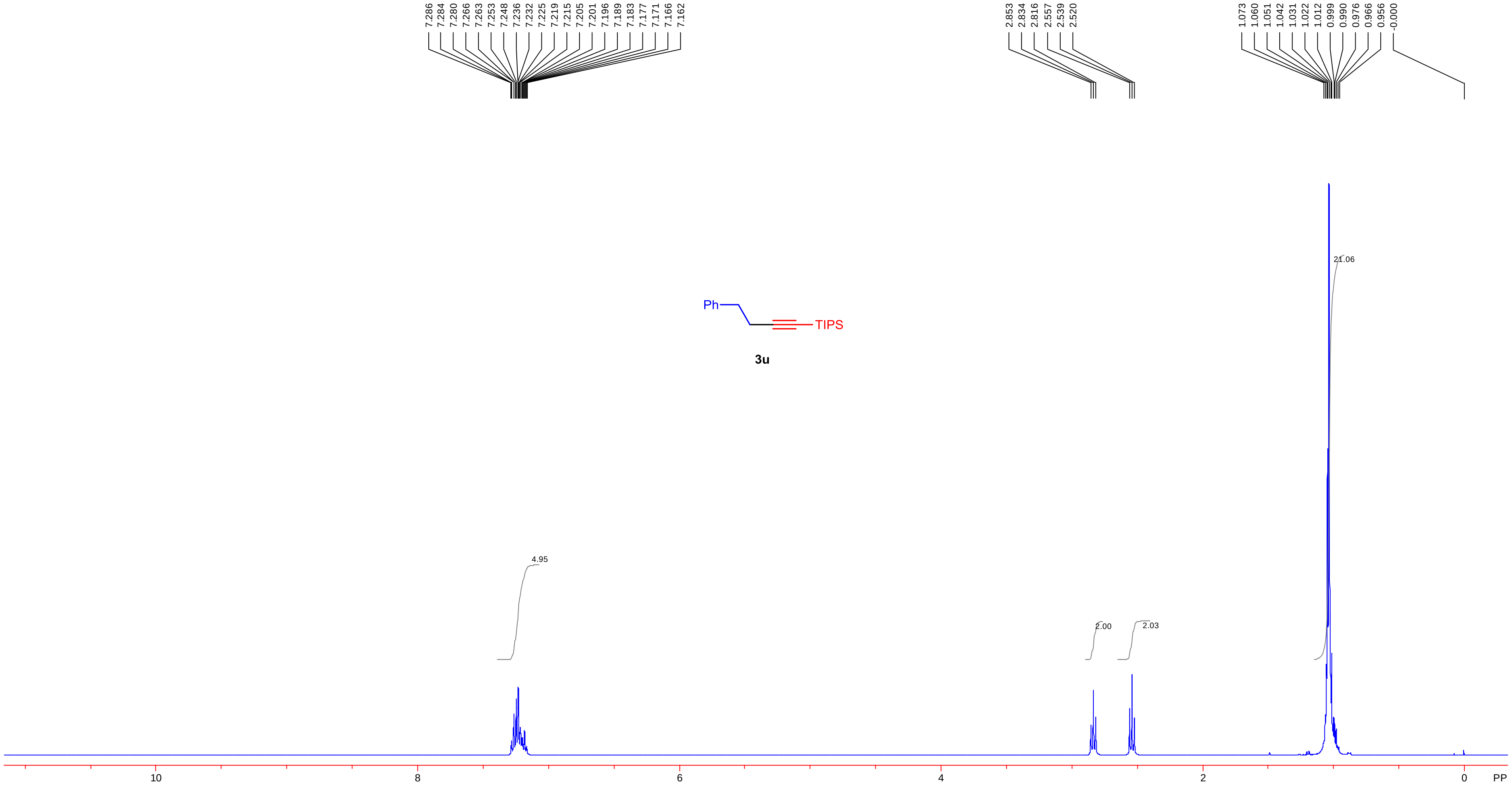

Supplementary Figure 94. ^{13}C NMR(100 MHz, $CDCl_3$)

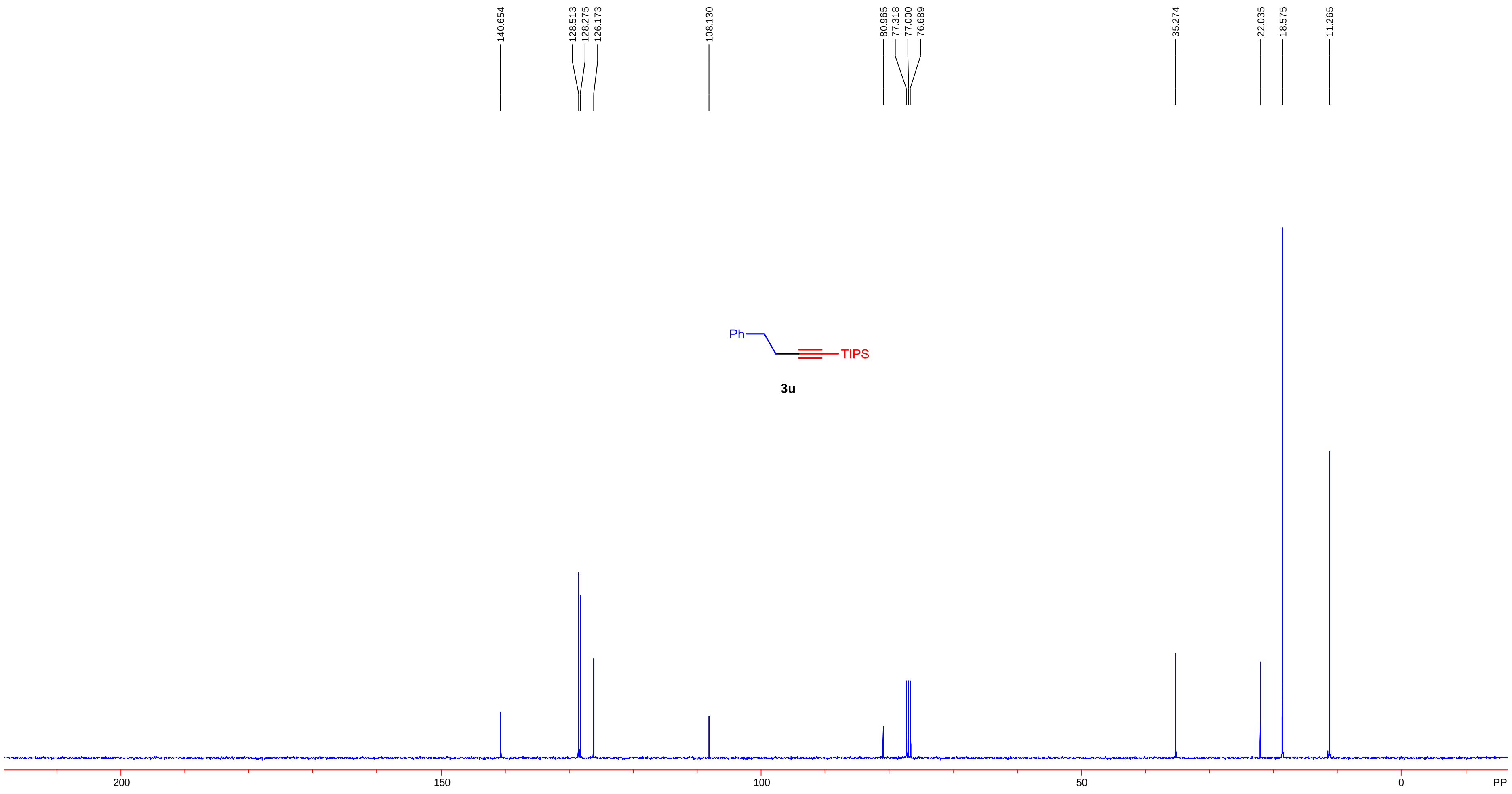

Supplementary Figure 95. ^{1}H NMR(400 MHz, $CDCl_3$)

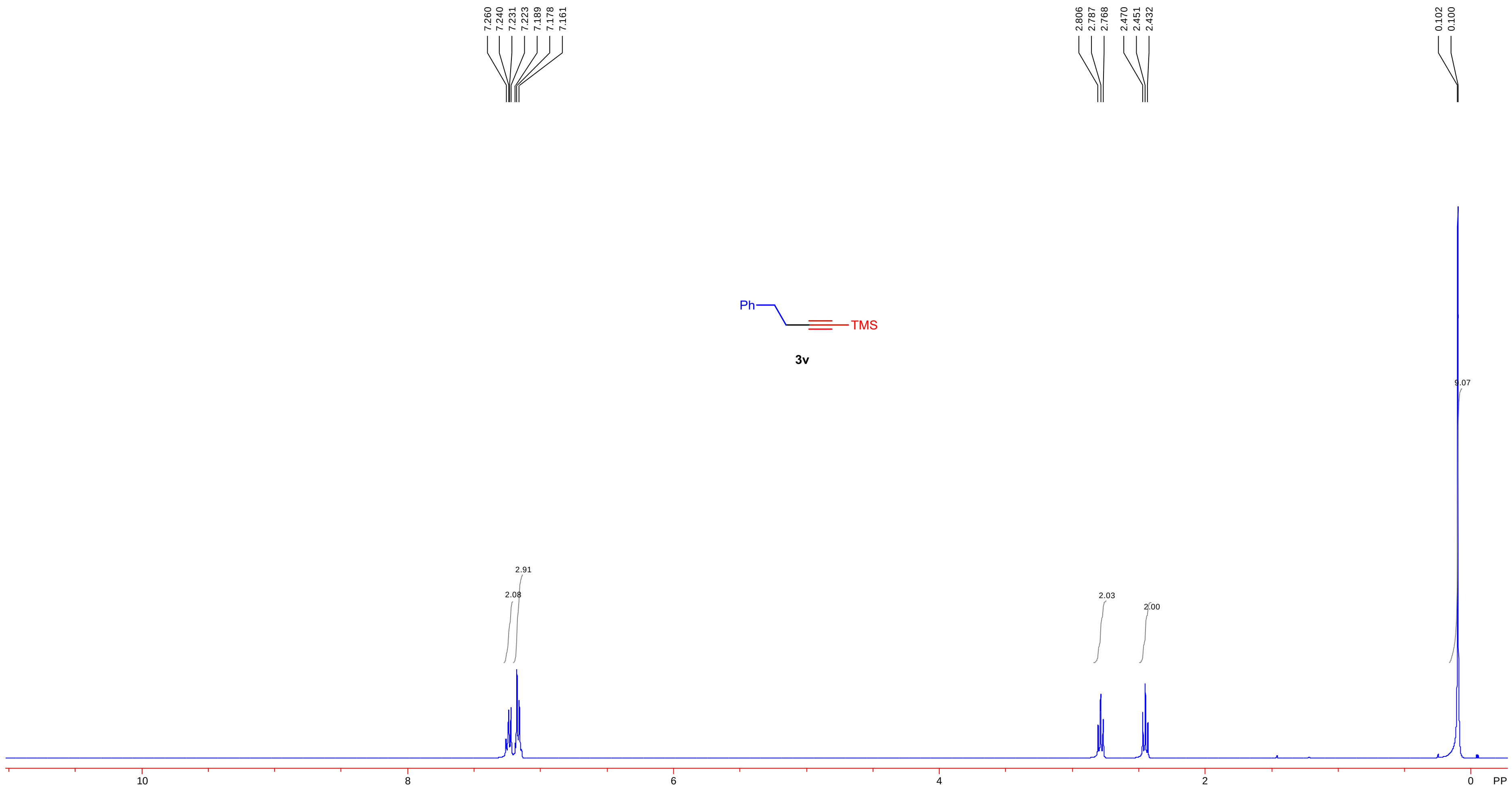

Supplementary Figure 96. ^{13}C NMR(100 MHz, $CDCl_3$)

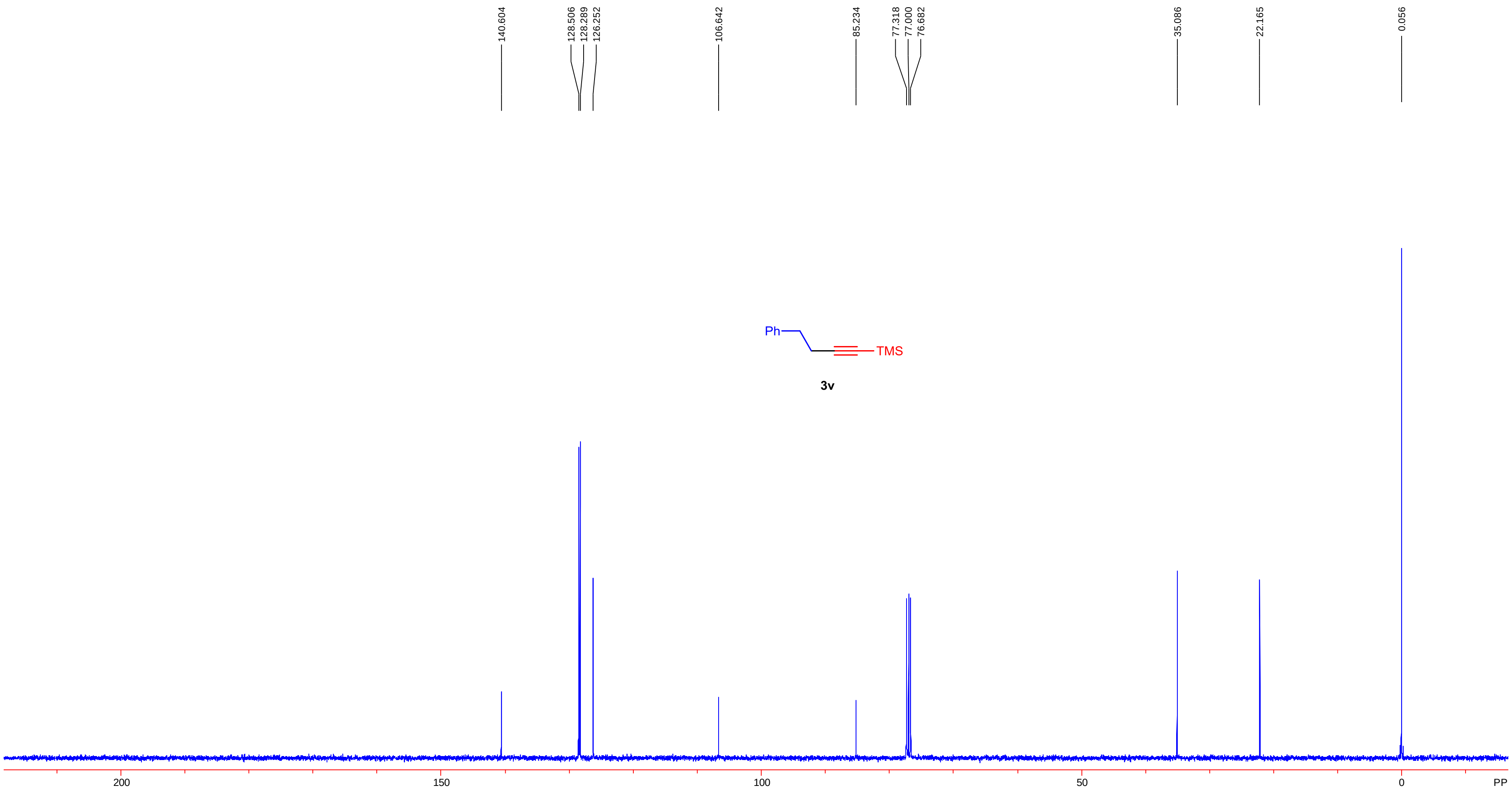

Supplementary Figure 97. ^{1}H NMR(400 MHz, $CDCl_3$)

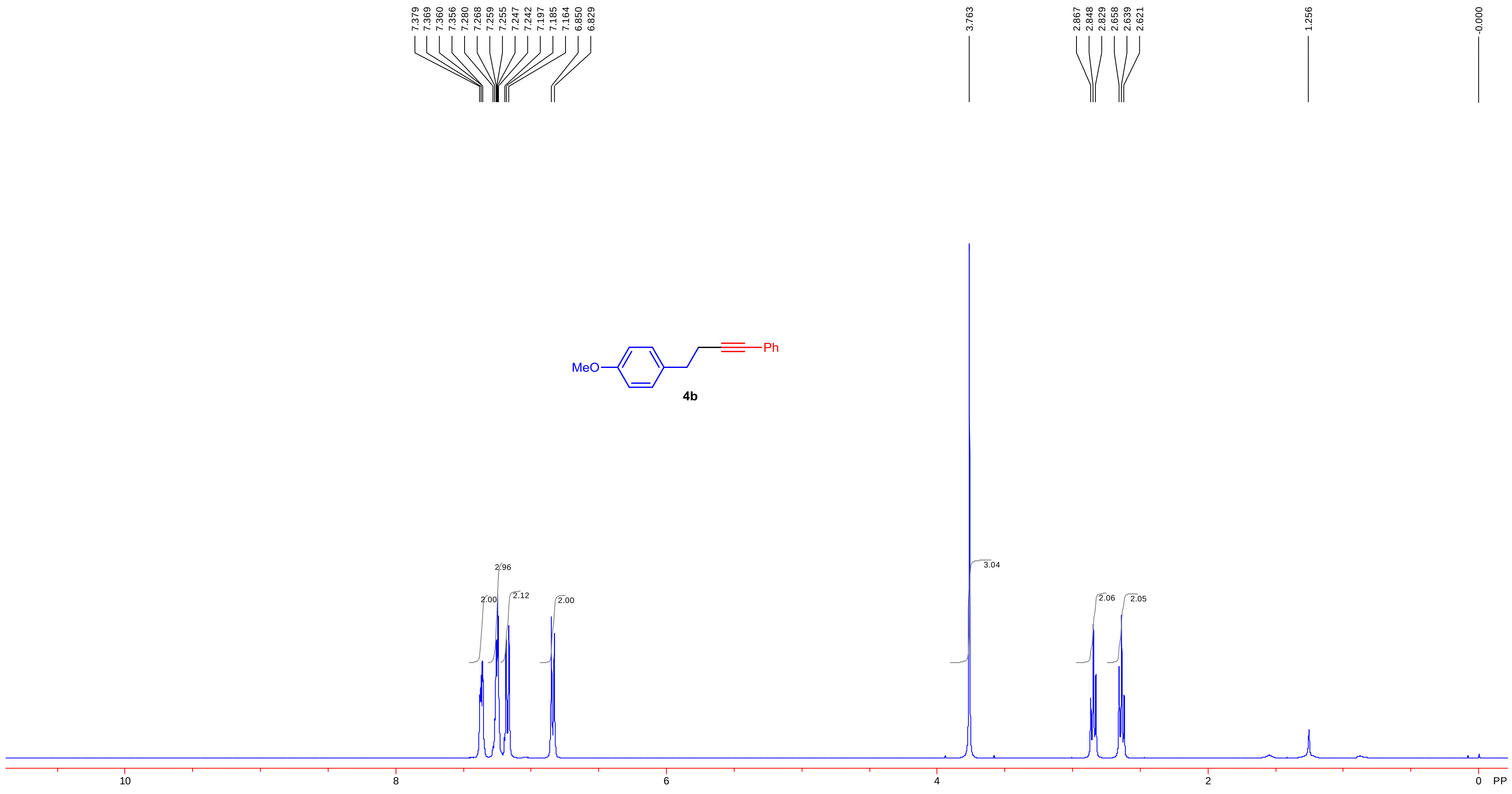

Supplementary Figure 98. ^{13}C NMR(100 MHz, $CDCl_3$)

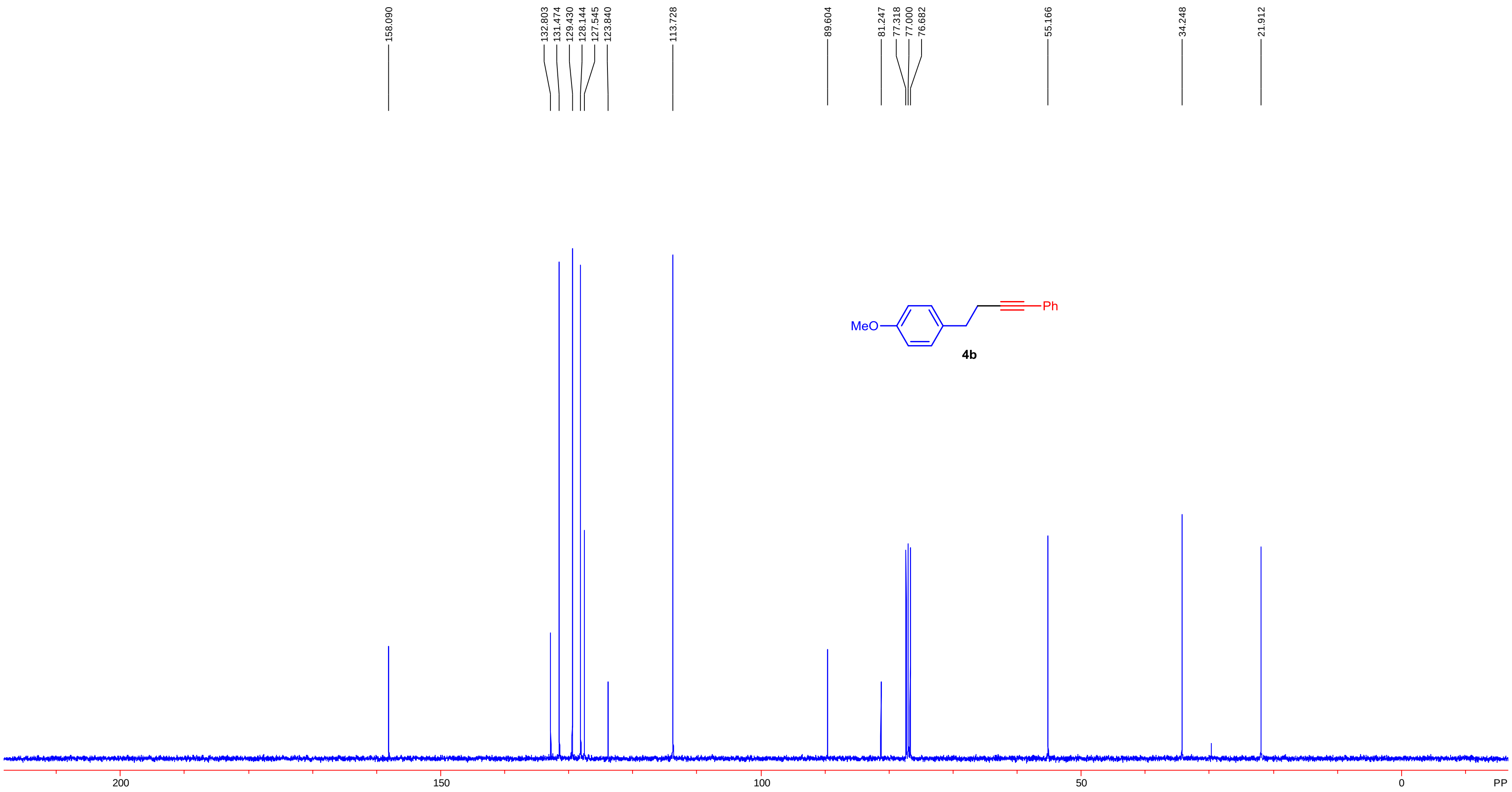

Supplementary Figure 99. ^{1}H NMR (600 MHz, $CDCl_3$)

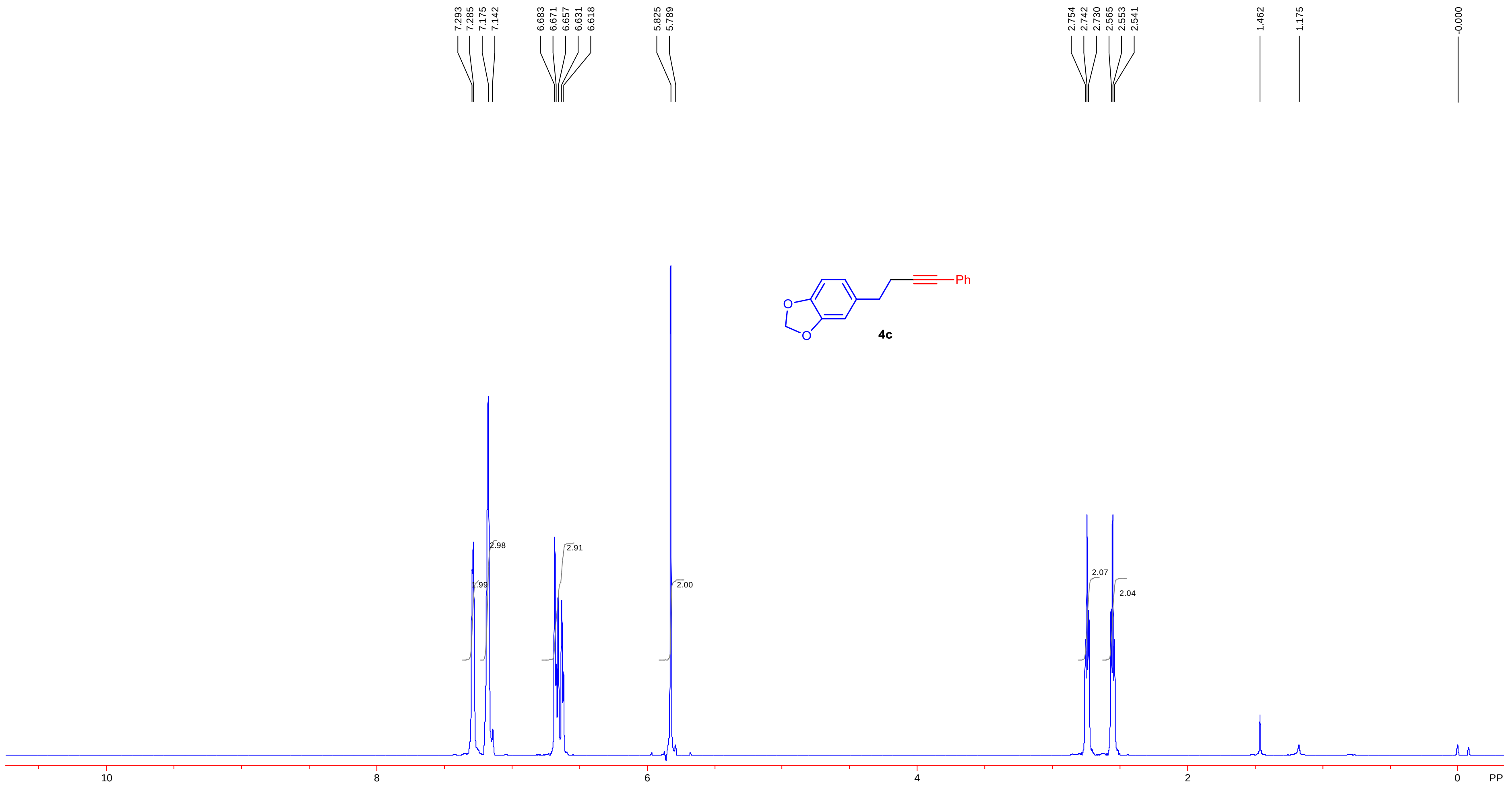

Supplementary Figure 100. ^{13}C NMR (151 MHz, $CDCl_3$)

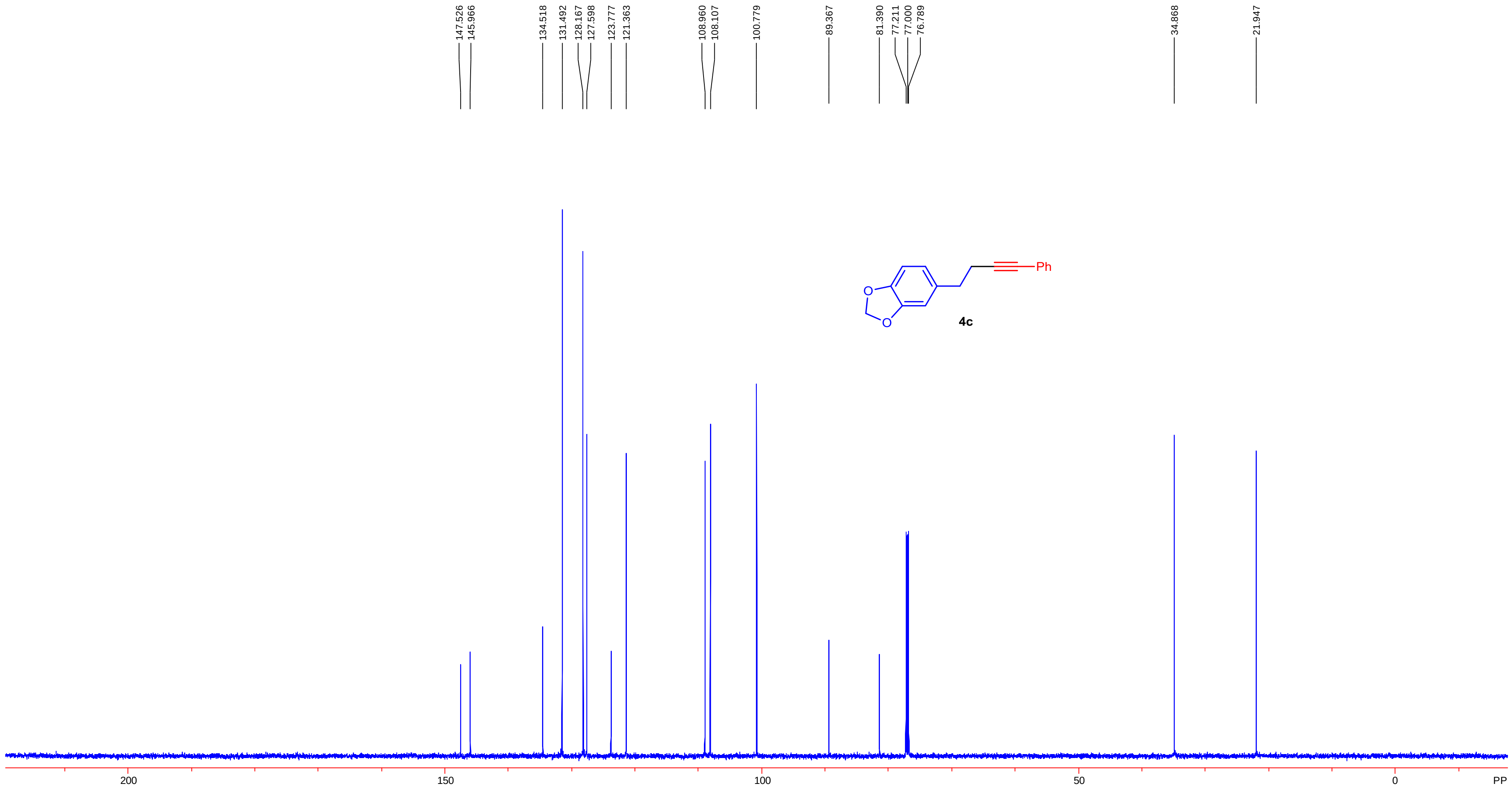

Supplementary Figure 101. ^{1}H NMR(400 MHz, $CDCl_3$)

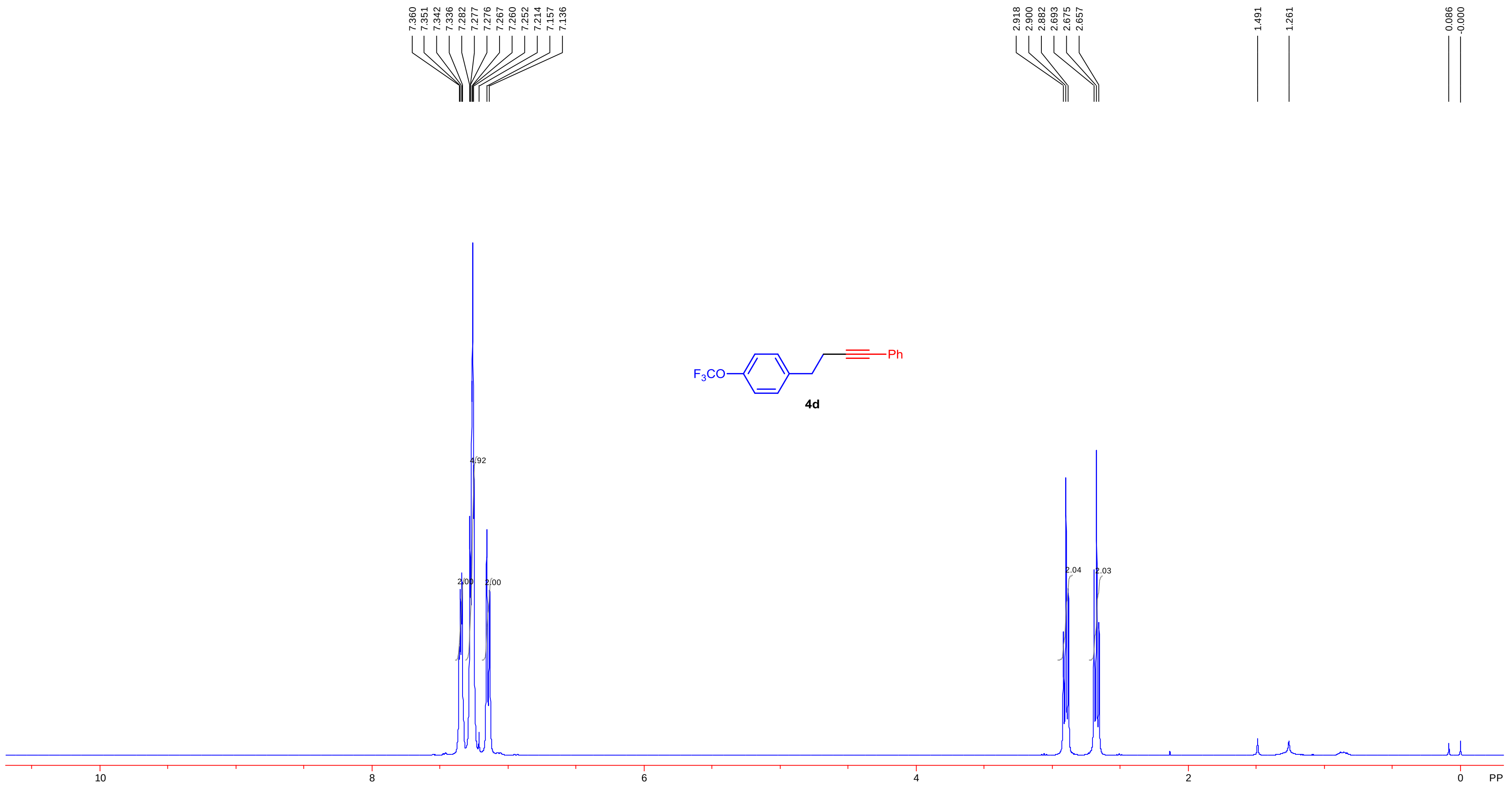

Supplementary Figure 102. ^{13}C NMR(100 MHz, $CDCl_3$)

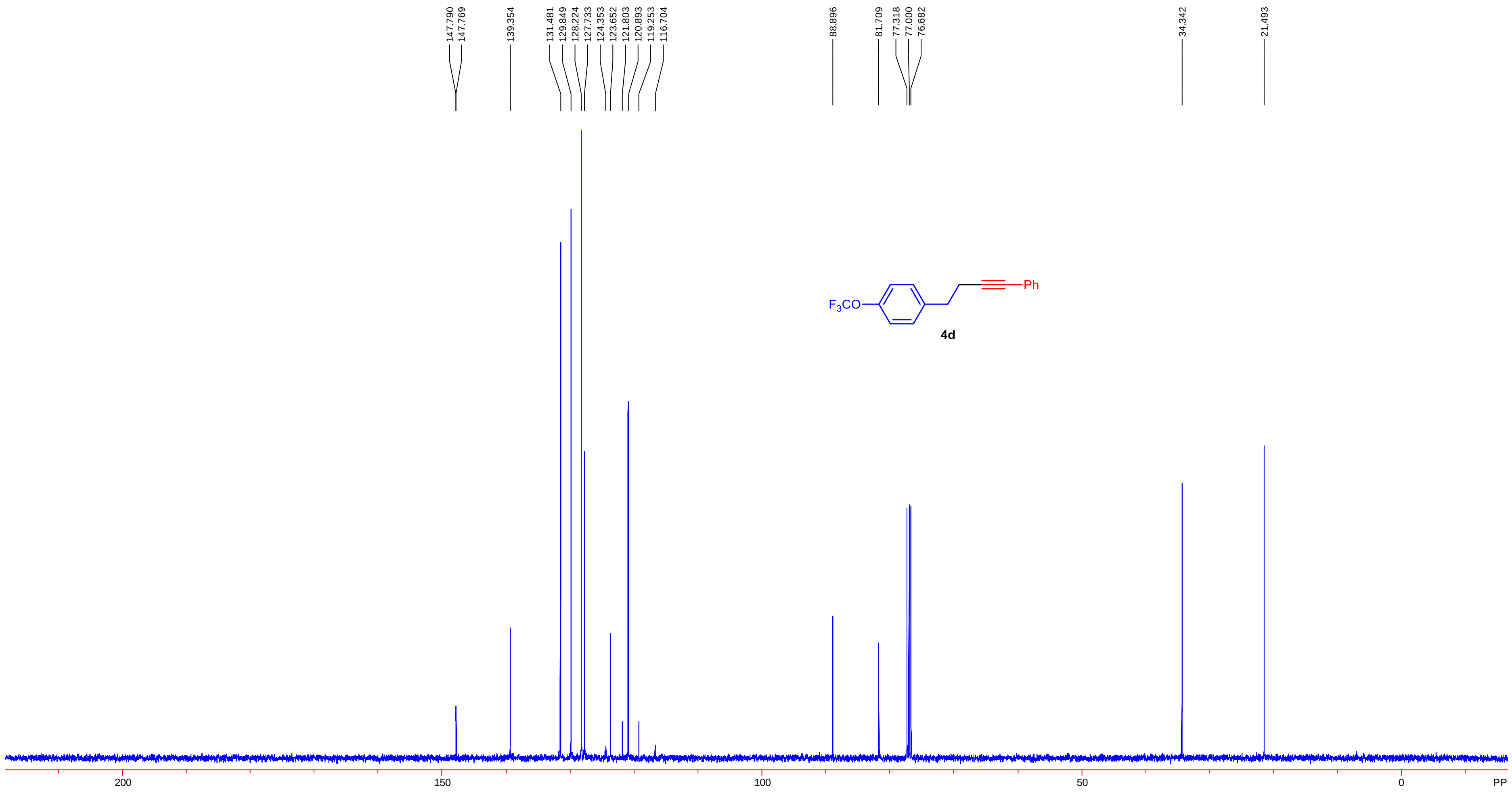

Supplementary Figure 103. ^{1}H NMR (600 MHz, $CDCl_3$)

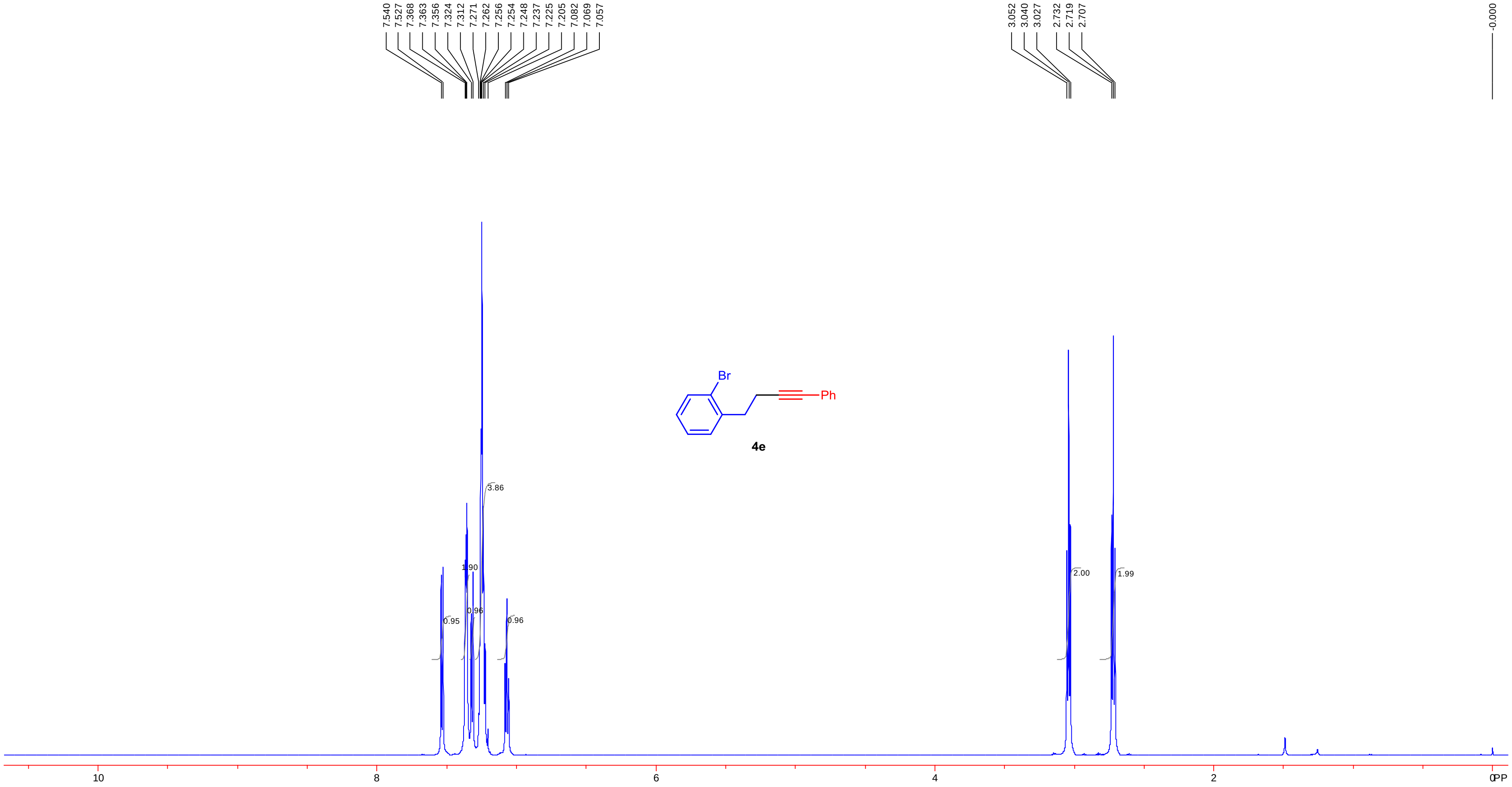

Supplementary Figure 104. ^{13}C NMR (151 MHz, $CDCl_3$)

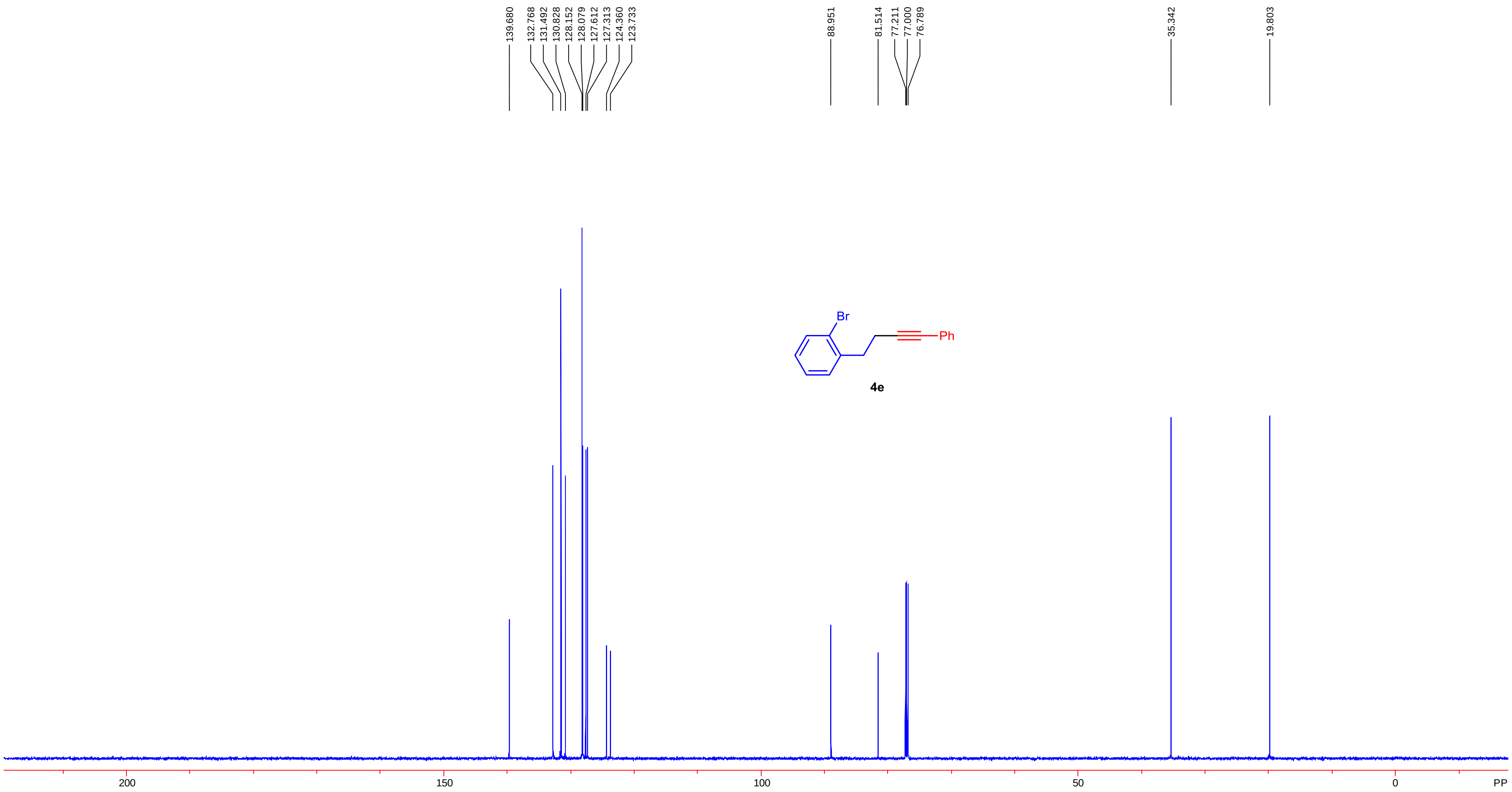

Supplementary Figure 105. ^{1}H NMR(400 MHz, $CDCl_3$)

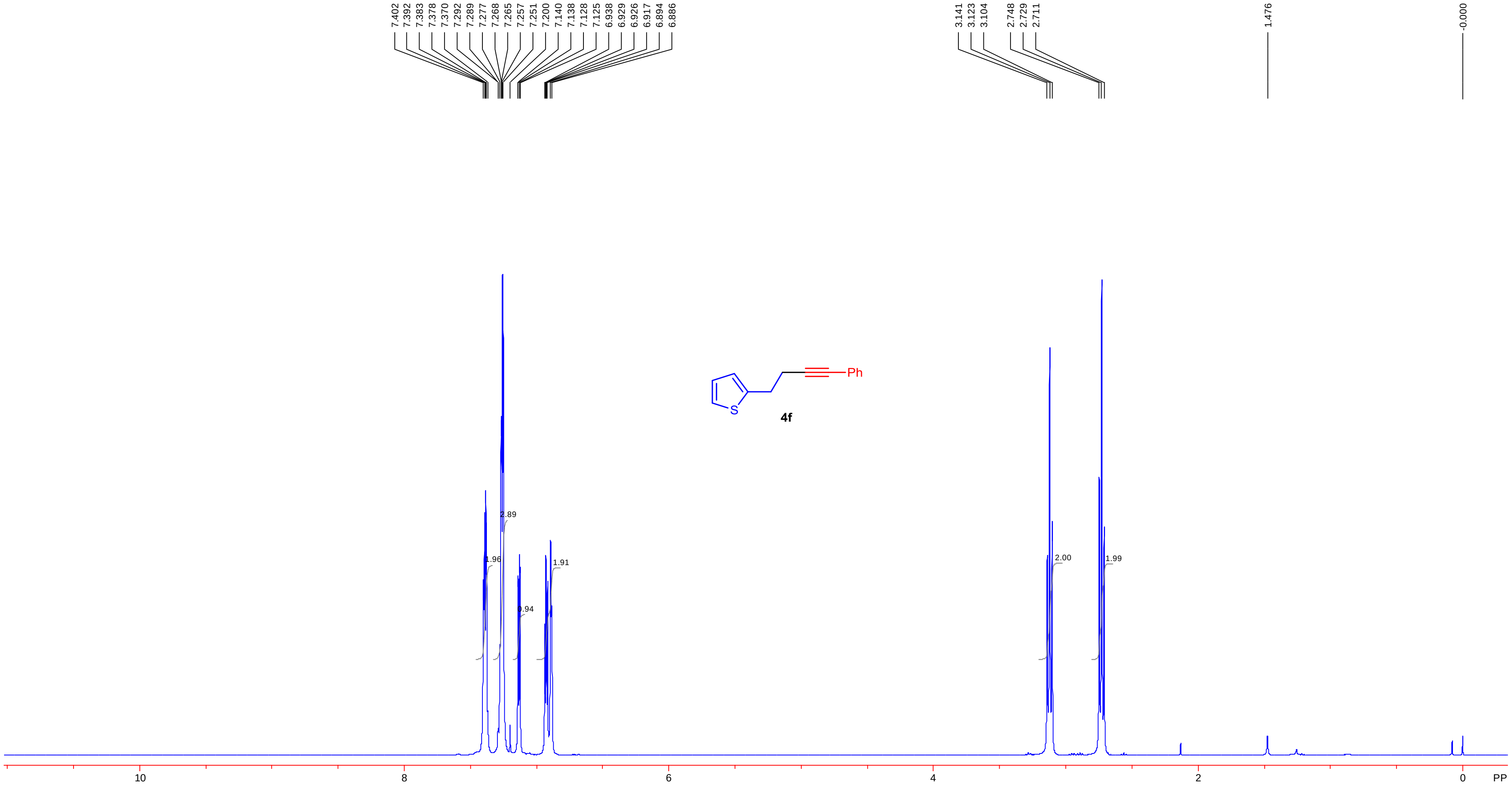

Supplementary Figure 106. ^{13}C NMR(100 MHz, $CDCl_3$)

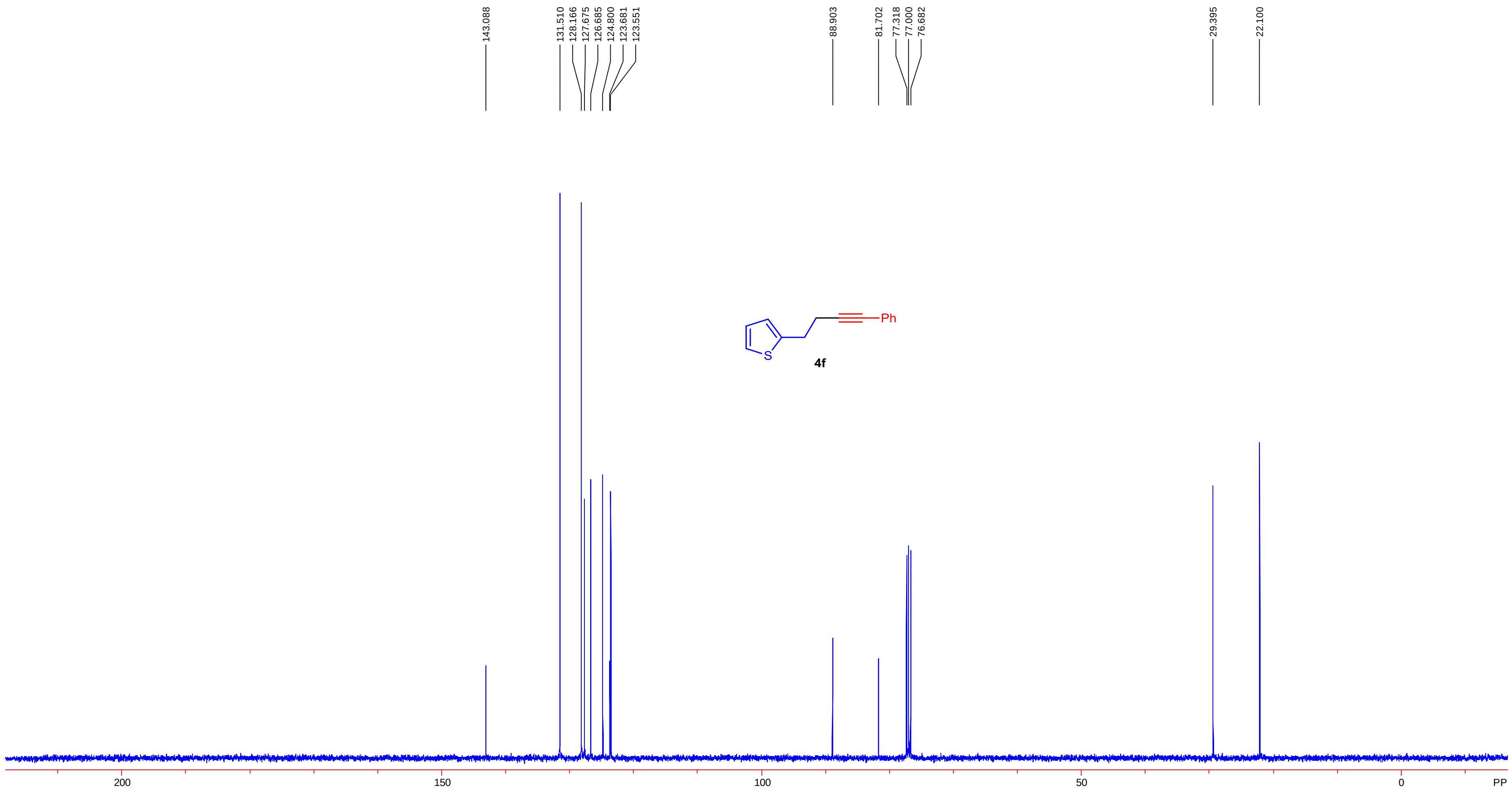

Supplementary Figure 107. ^{1}H NMR(400 MHz, $CDCl_3$)

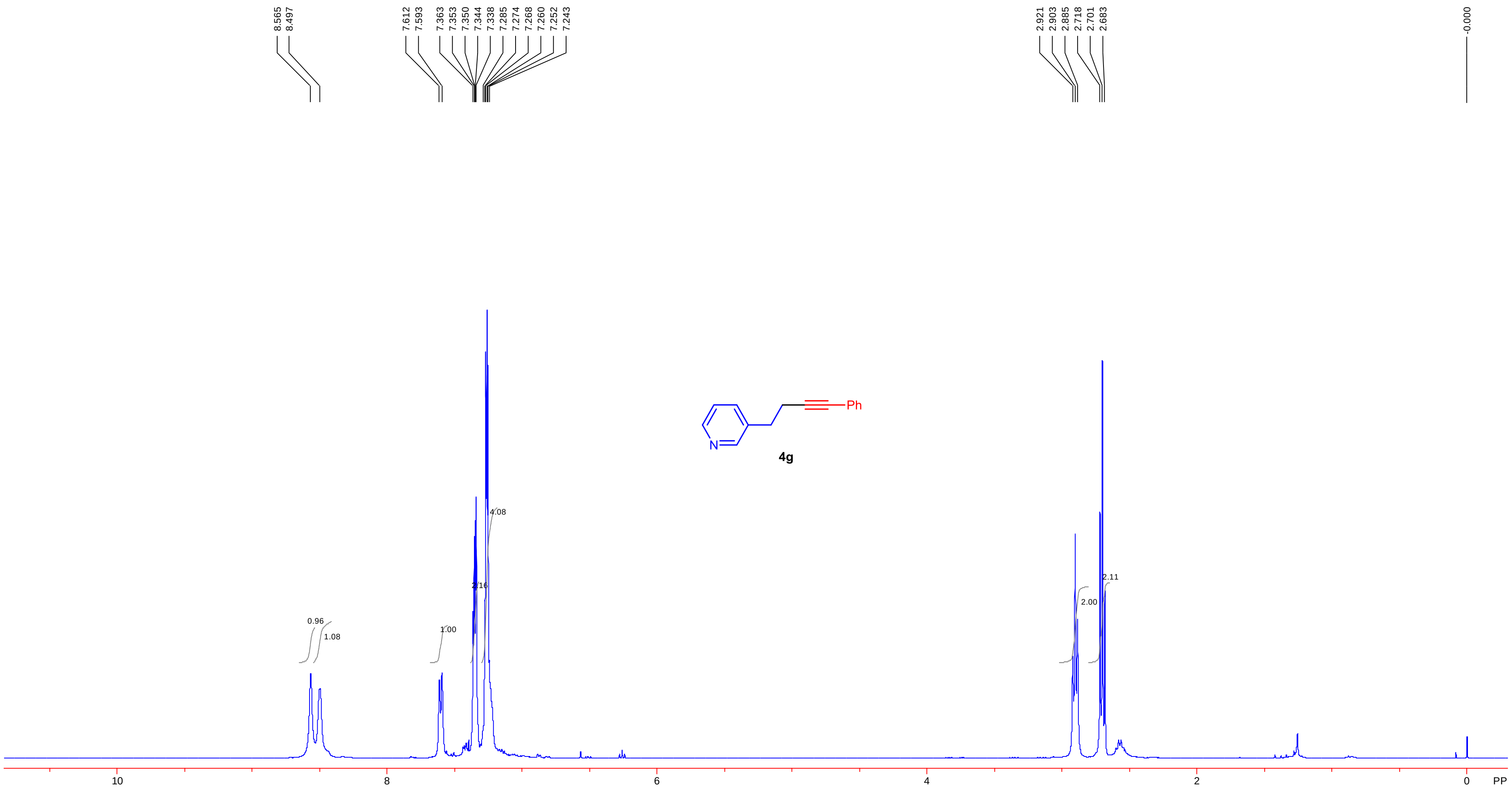

Supplementary Figure 108. ^{13}C NMR(100 MHz, $CDCl_3$)

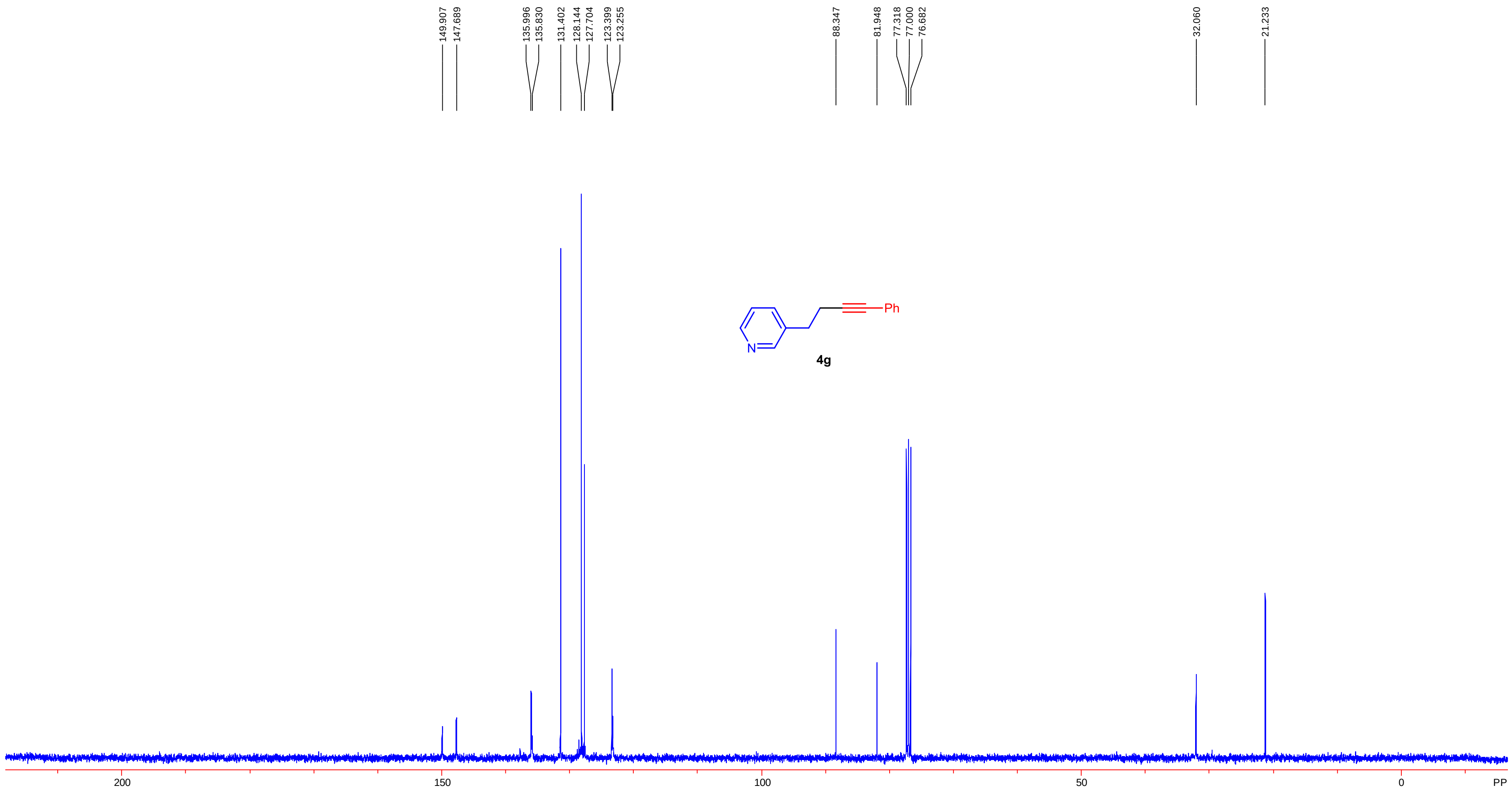

Supplementary Figure 109. ^{1}H NMR (600 MHz, $CDCl_3$)

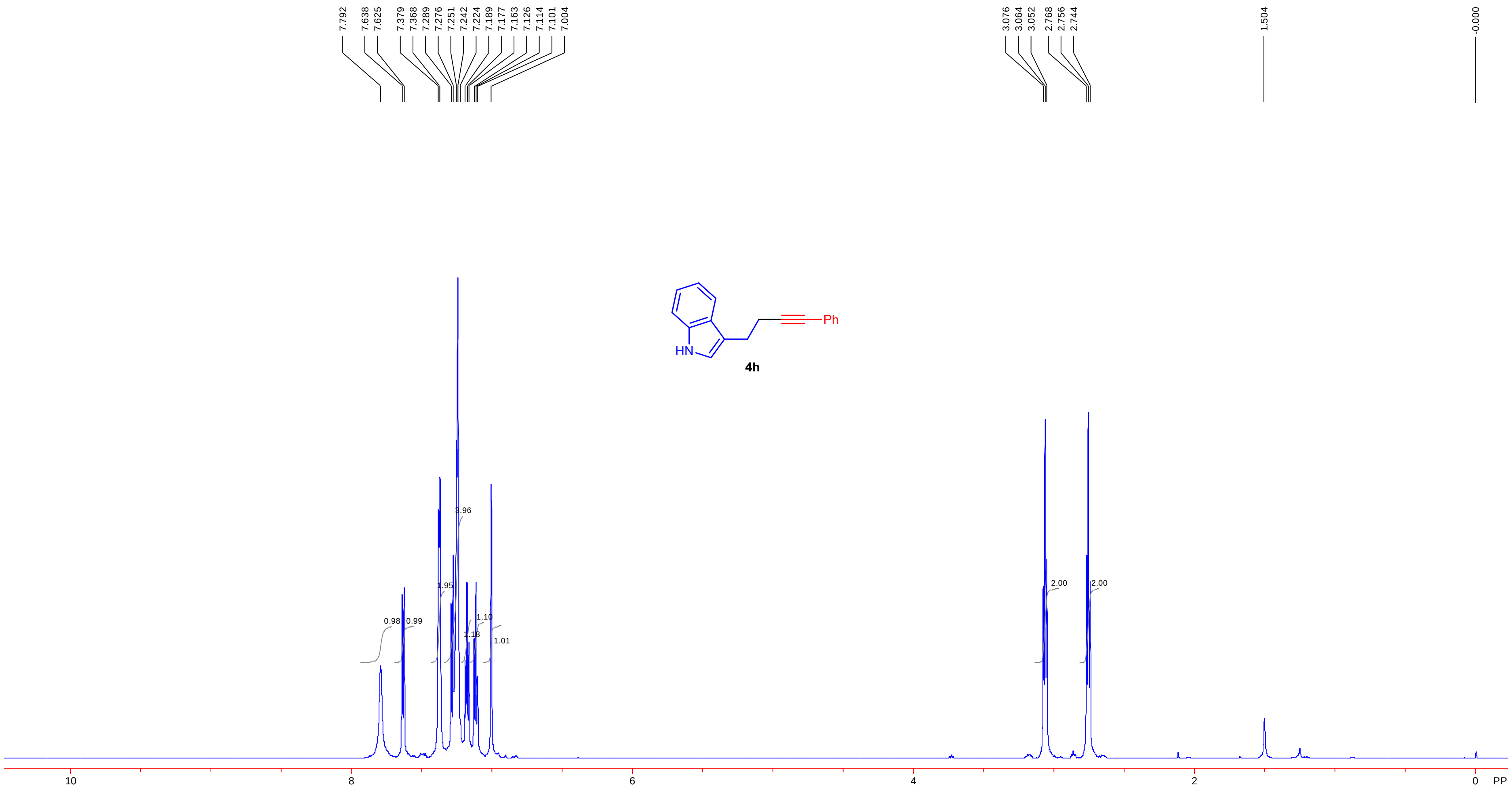

Supplementary Figure 110. ^{13}C NMR (151 MHz, $CDCl_3$)

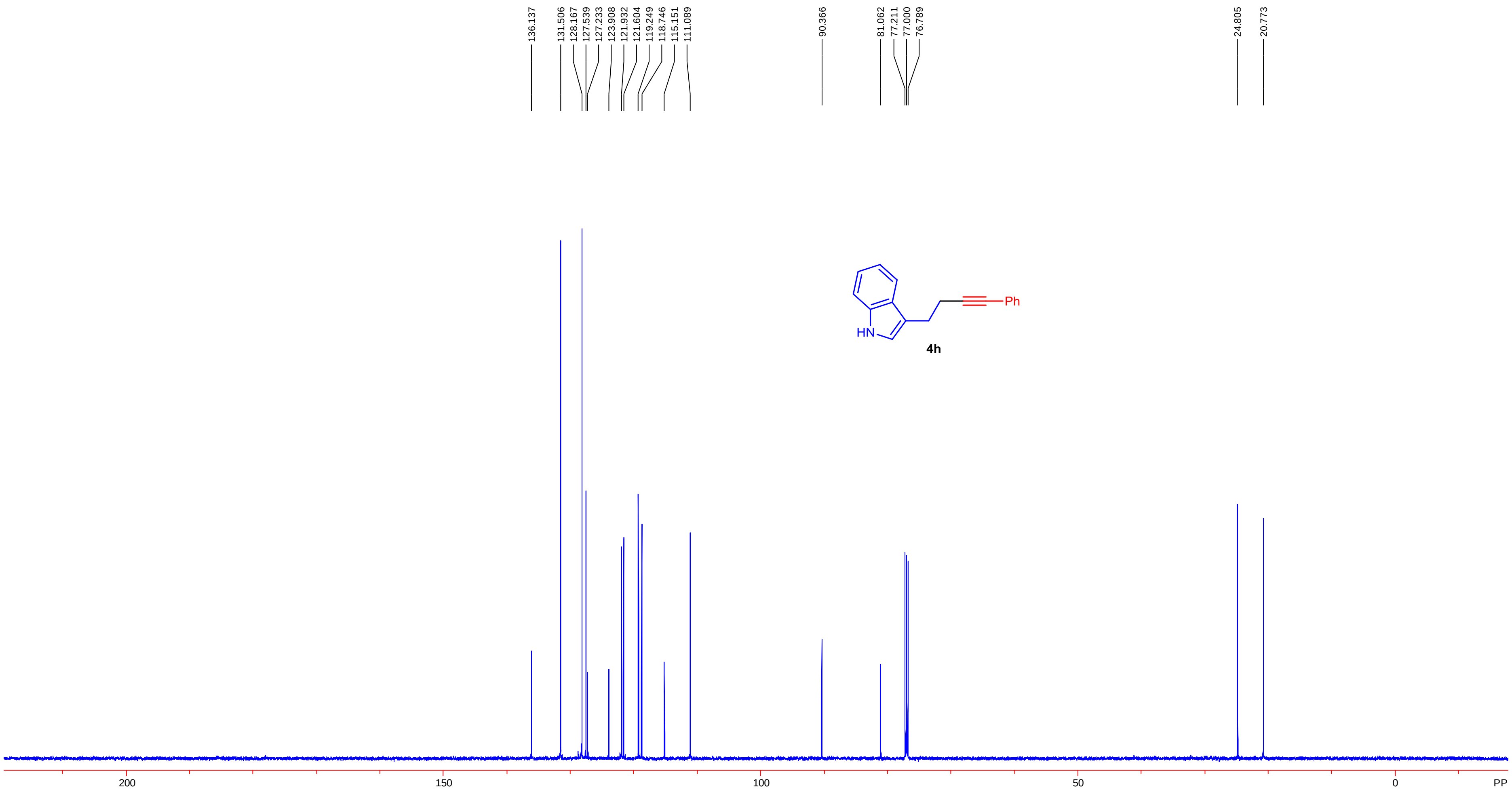

Supplementary Figure 111. ^{1}H NMR(400 MHz, $CDCl_3$)

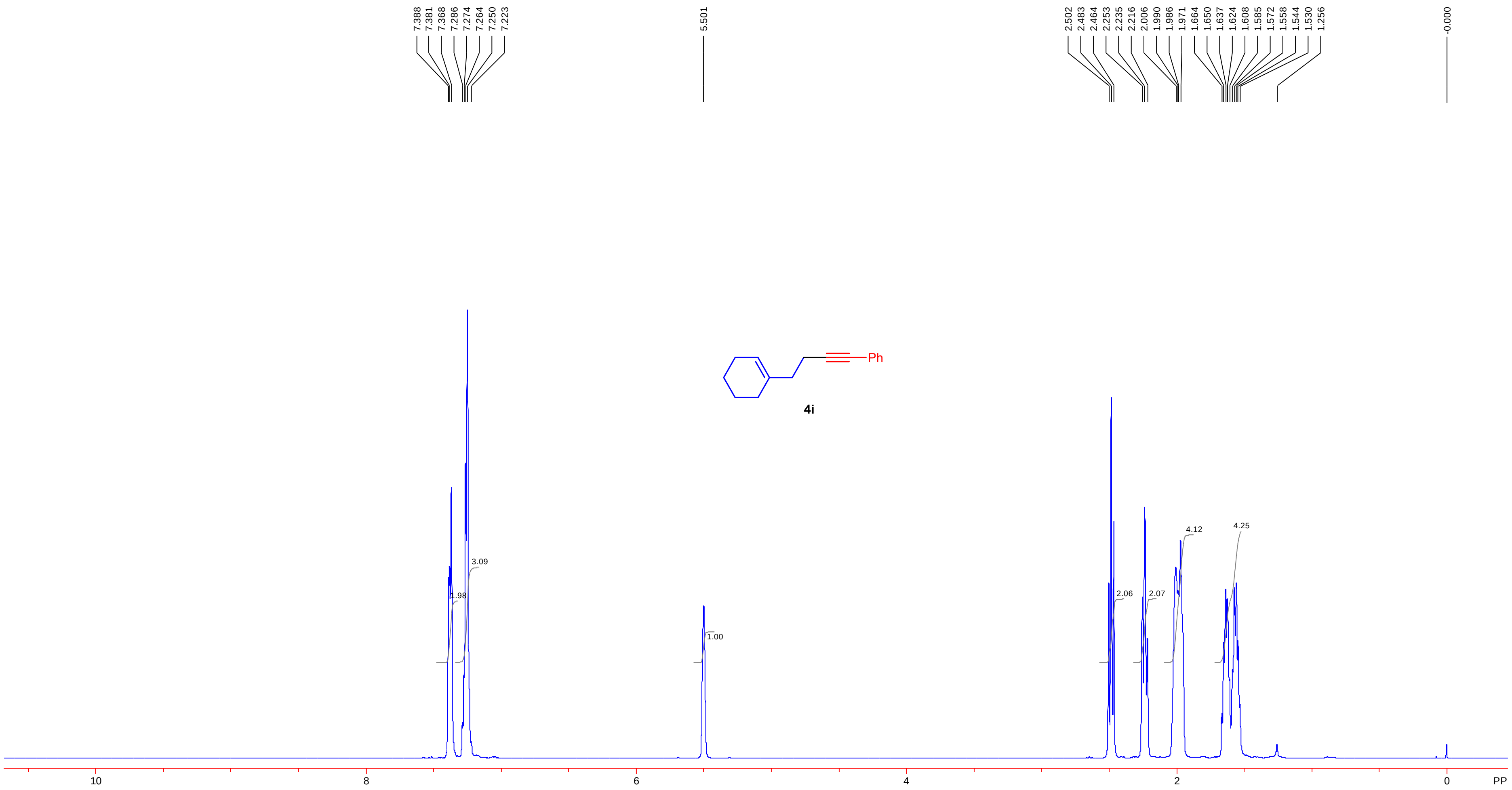

Supplementary Figure 112. ^{13}C NMR(100 MHz, $CDCl_3$)

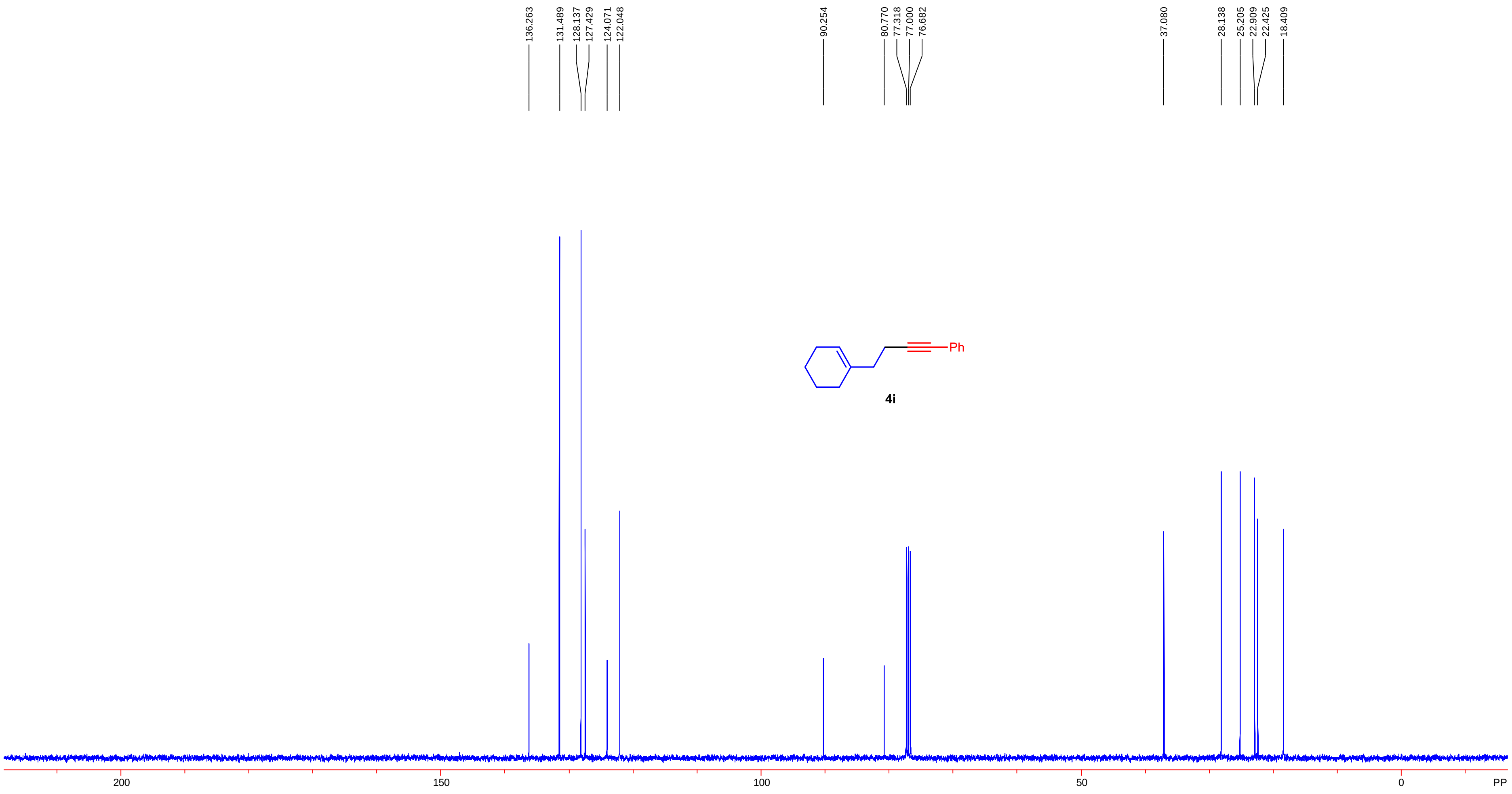

Supplementary Figure 113. ^{1}H NMR (600 MHz, $CDCl_3$)

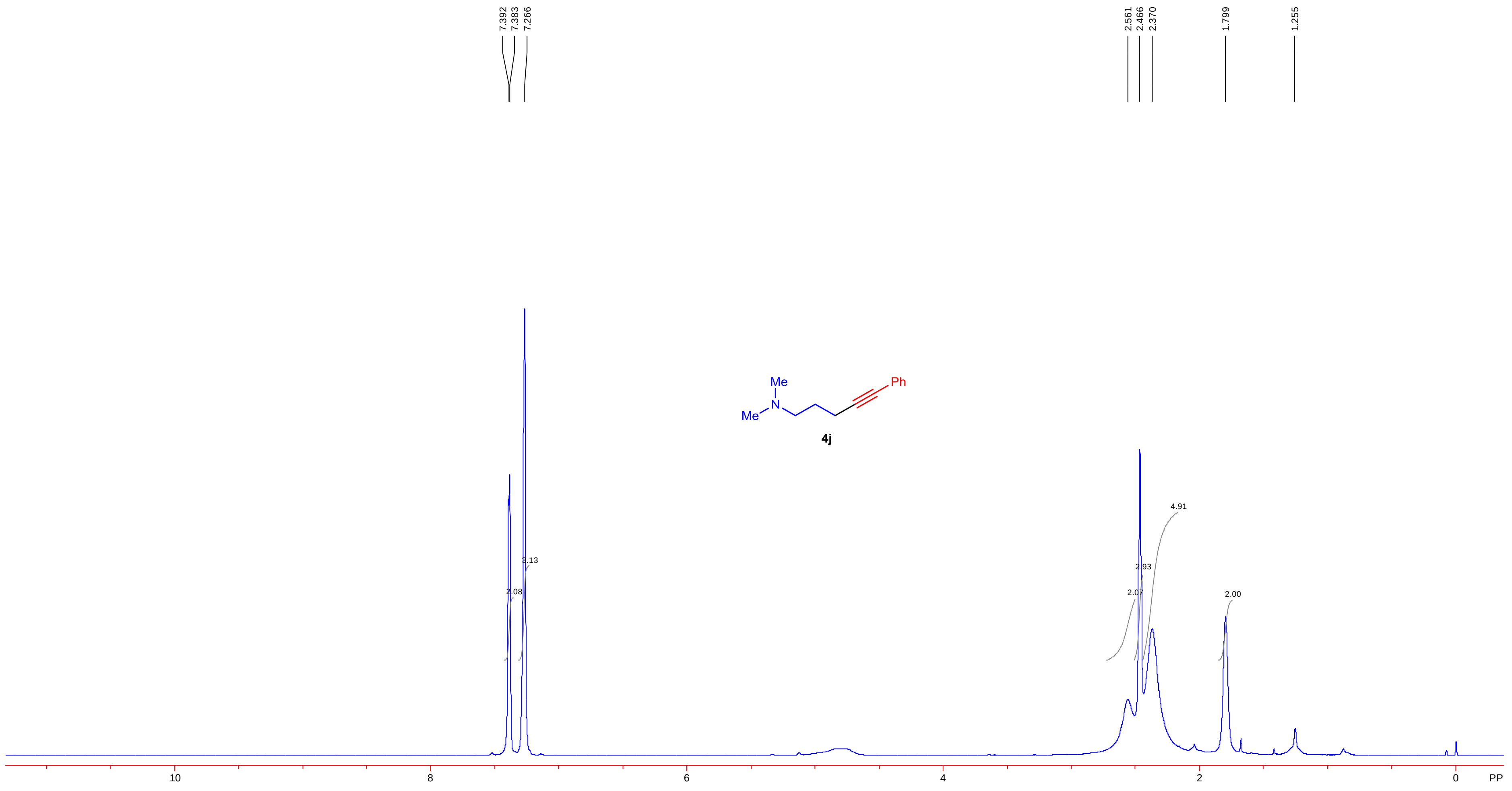

Supplementary Figure 114. ^{13}C NMR (151 MHz, $CDCl_3$)

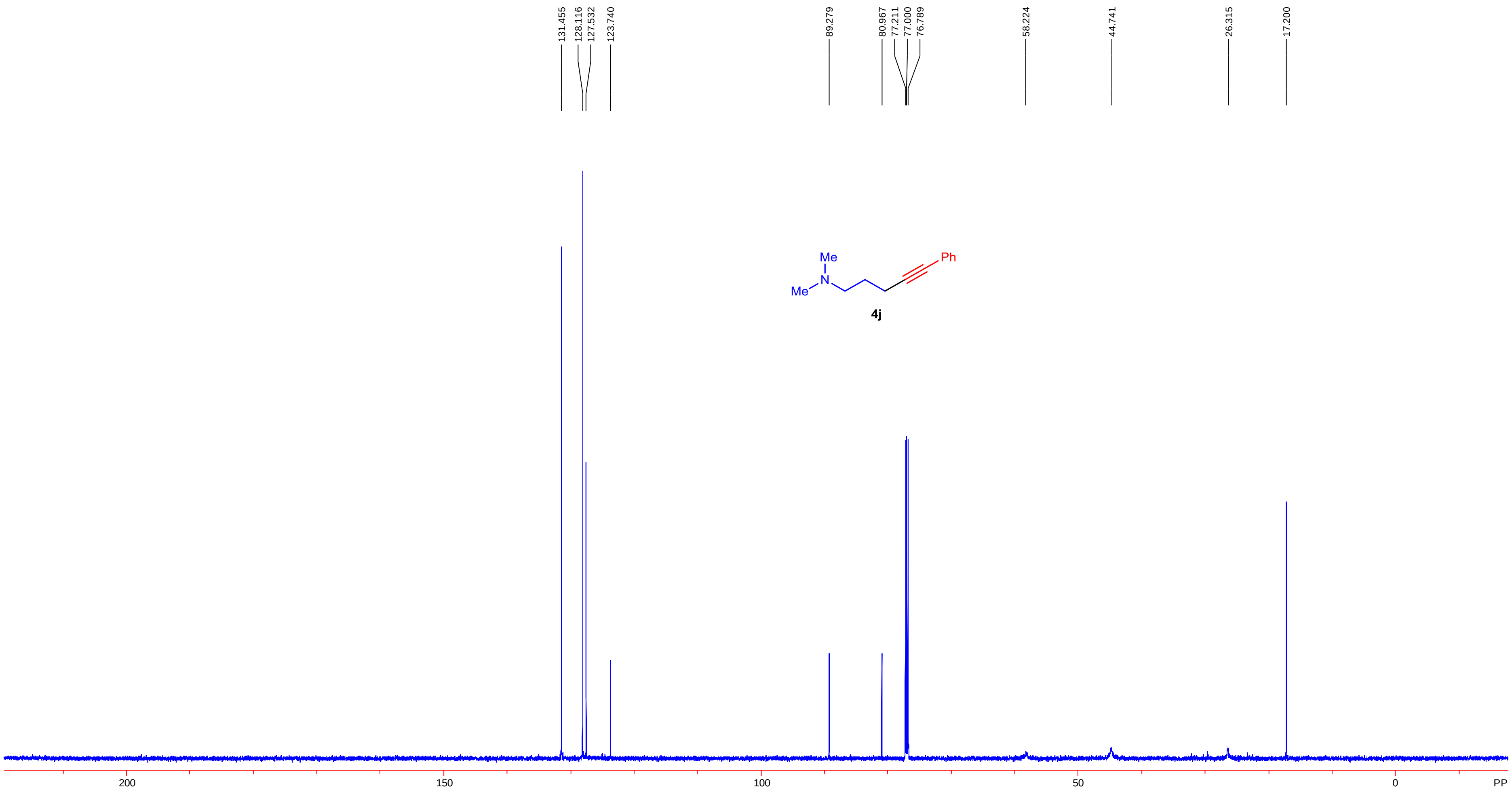

Supplementary Figure 115. ^{1}H NMR(400 MHz, $CDCl_3$)

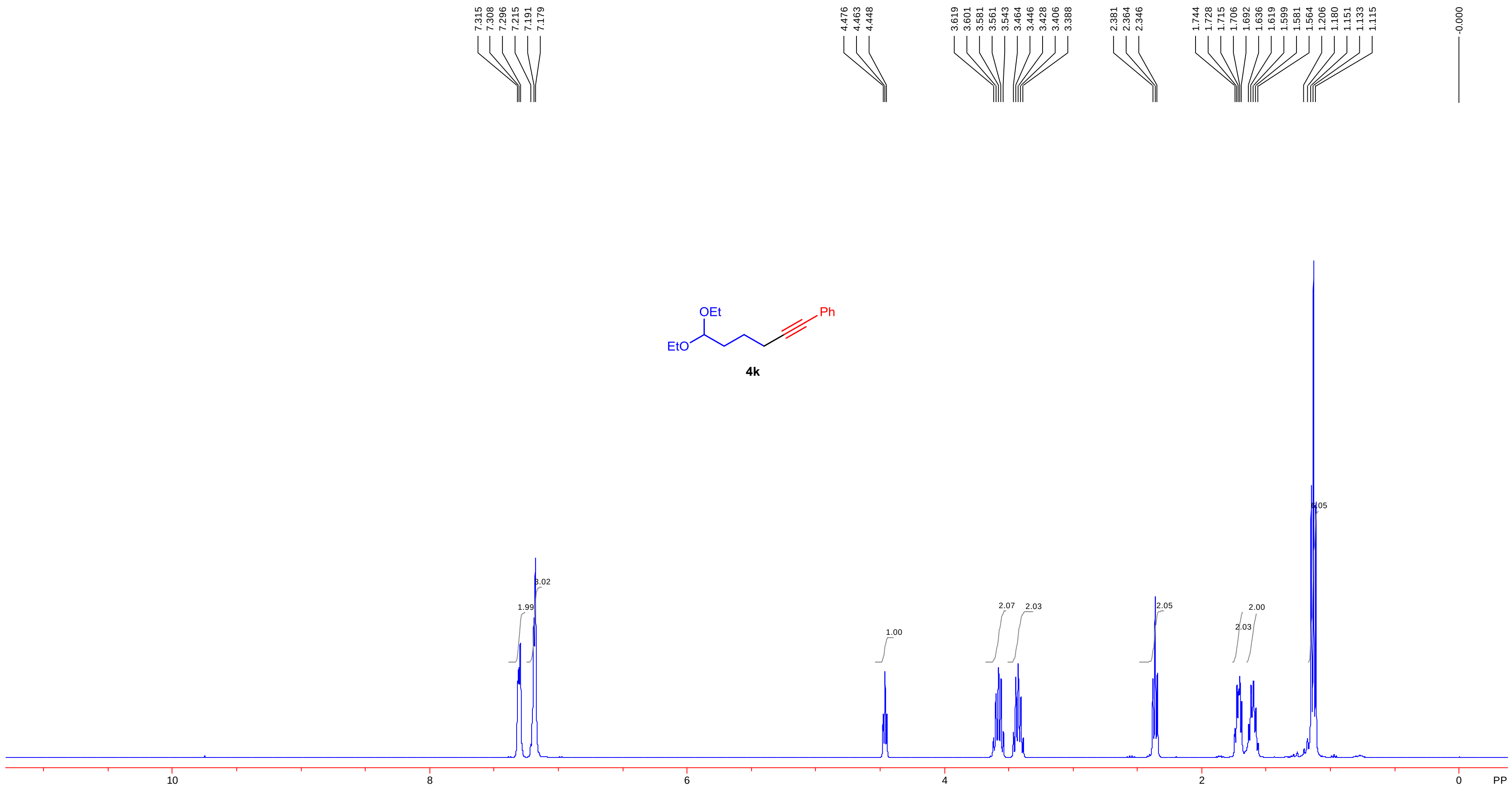

Supplementary Figure 116. ^{13}C NMR(100 MHz, $CDCl_3$)

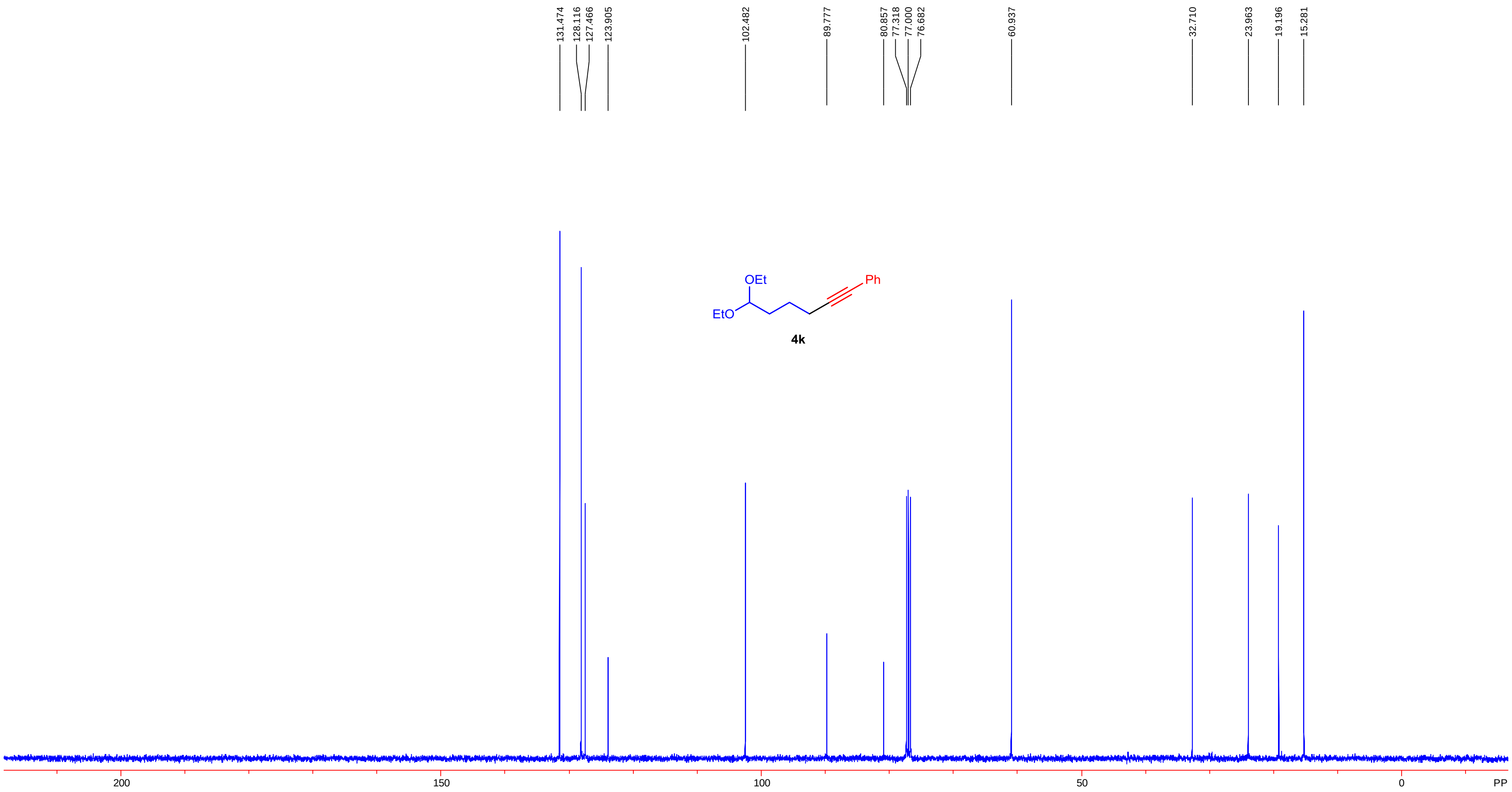

Supplementary Figure 117. ^{1}H NMR(400 MHz, $CDCl_3$)

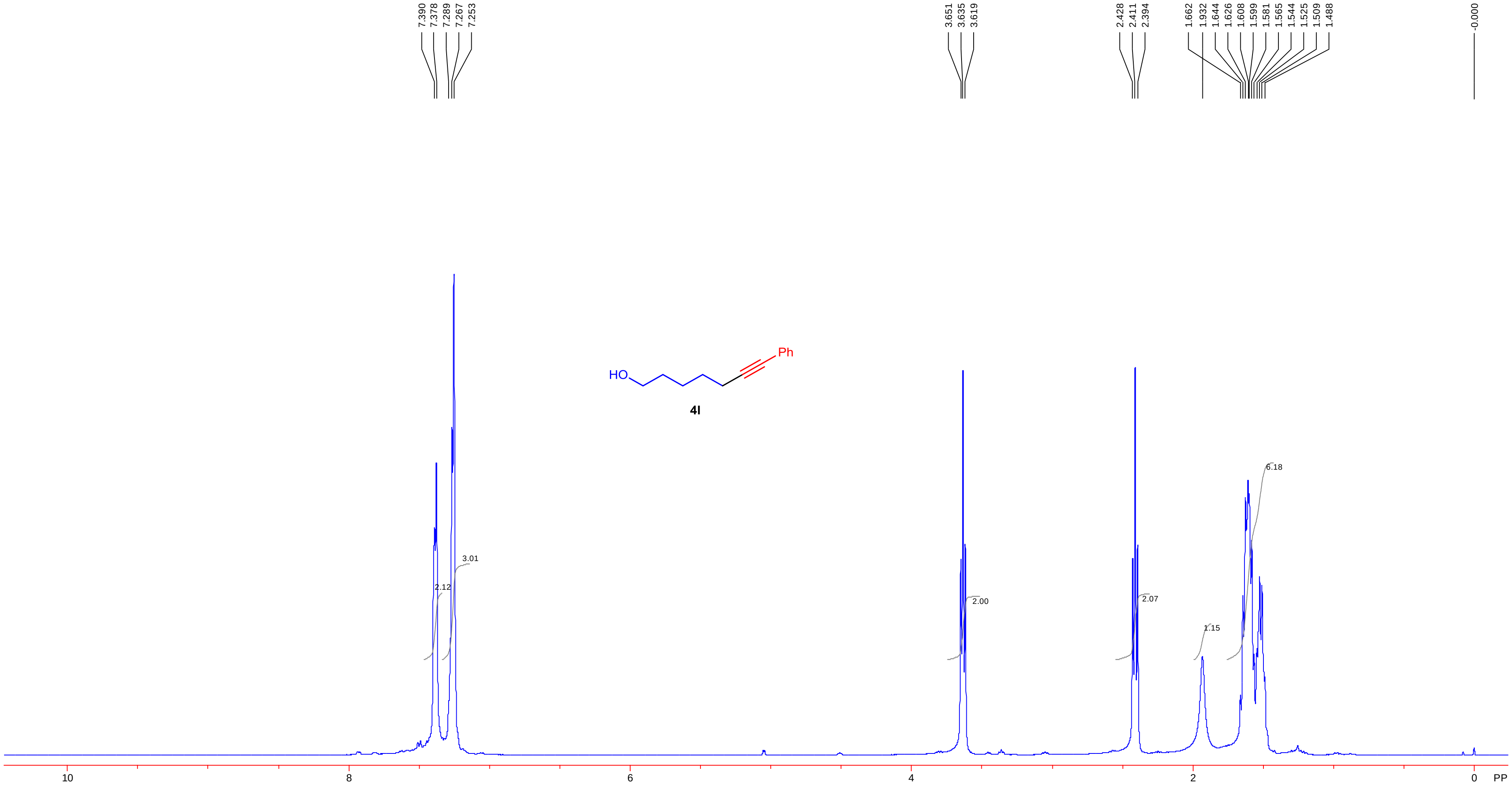

Supplementary Figure 118. ^{13}C NMR(100 MHz, $CDCl_3$)

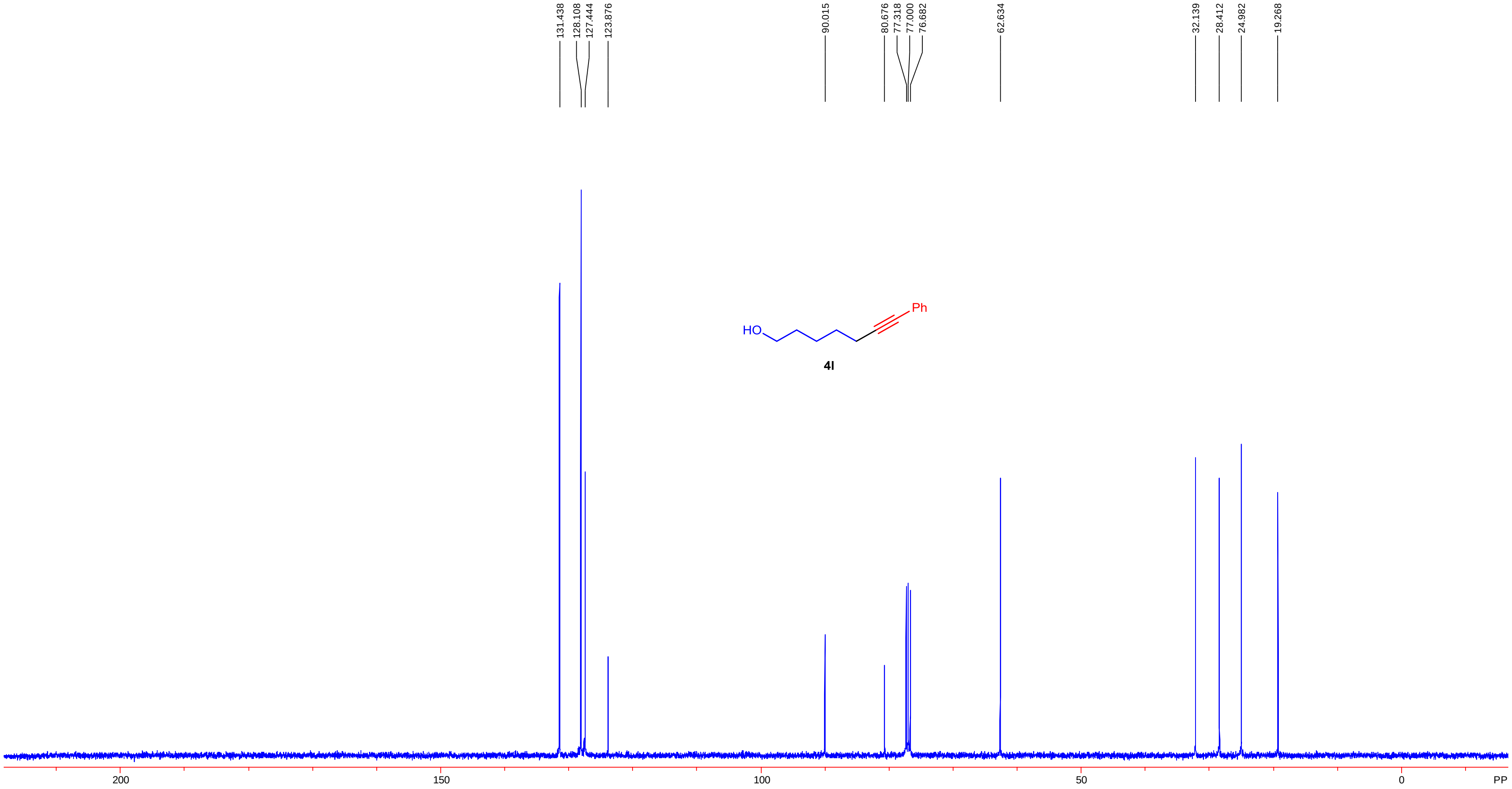

Supplementary Figure 119. ^{1}H NMR(400 MHz, $CDCl_3$)

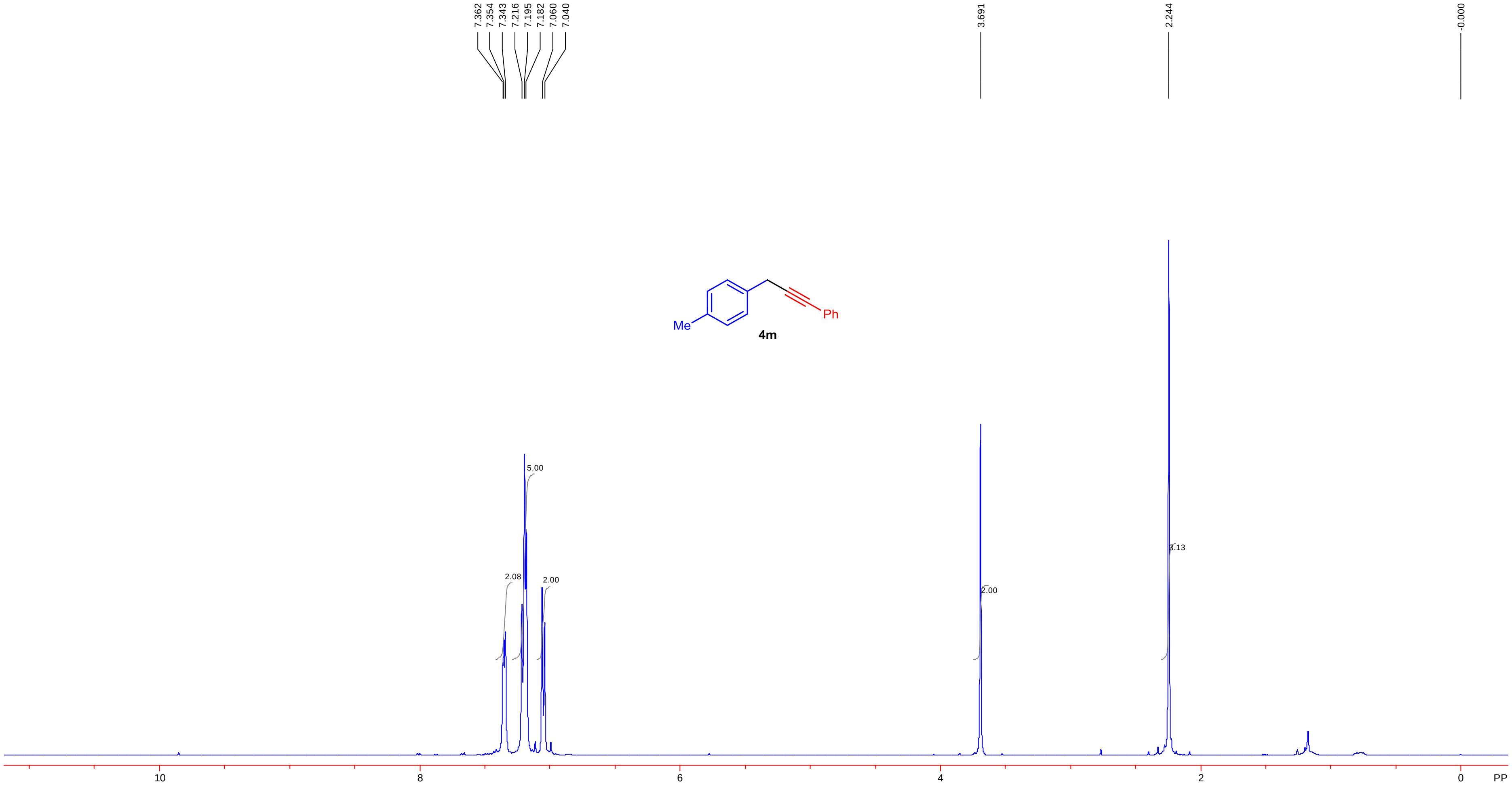

Supplementary Figure 120. ^{13}C NMR(100 MHz, $CDCl_3$)

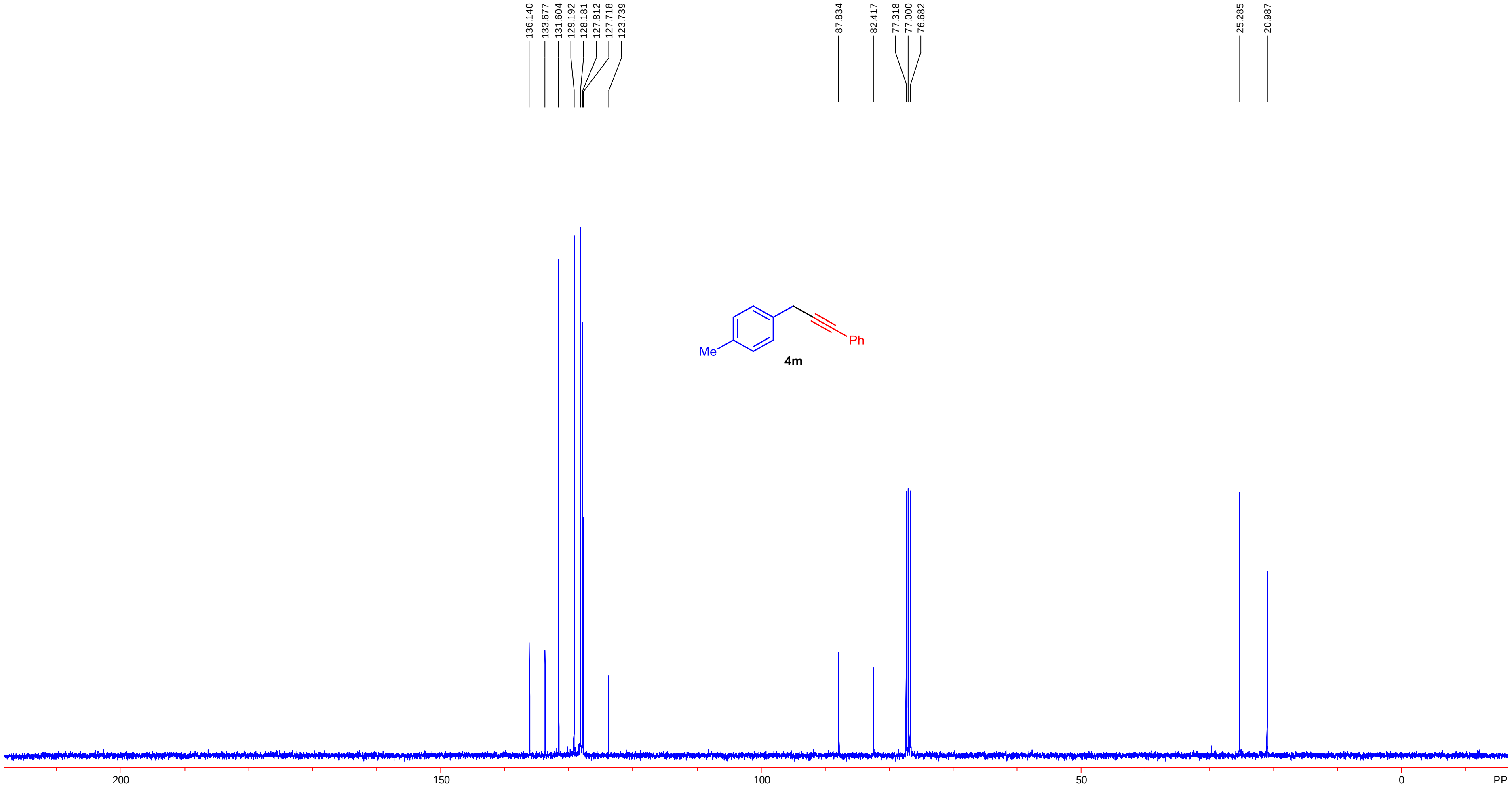

Supplementary Figure 121. ^{1}H NMR(400 MHz, $CDCl_3$)

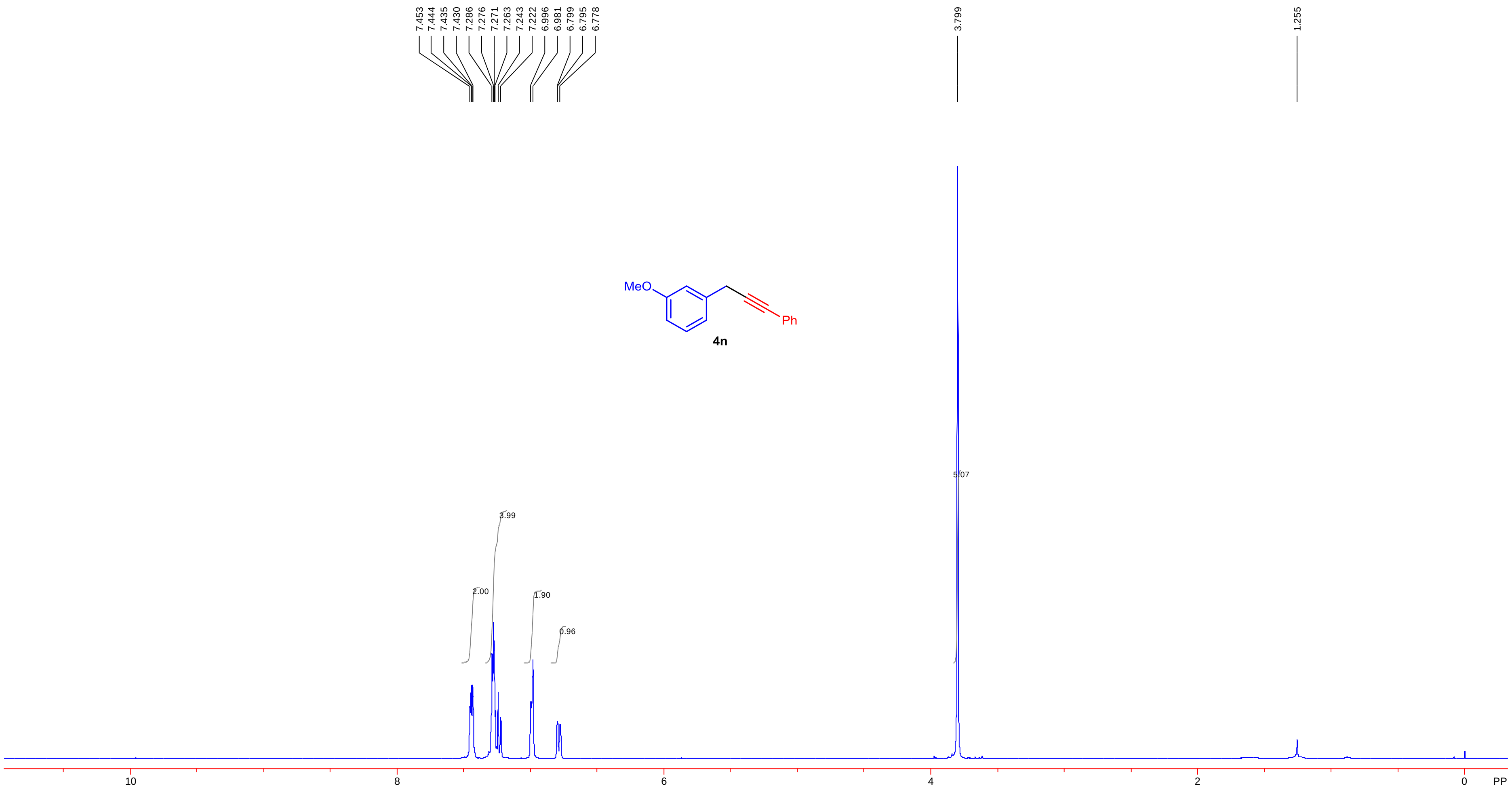

Supplementary Figure 122. ^{13}C NMR(100 MHz, $CDCl_3$)

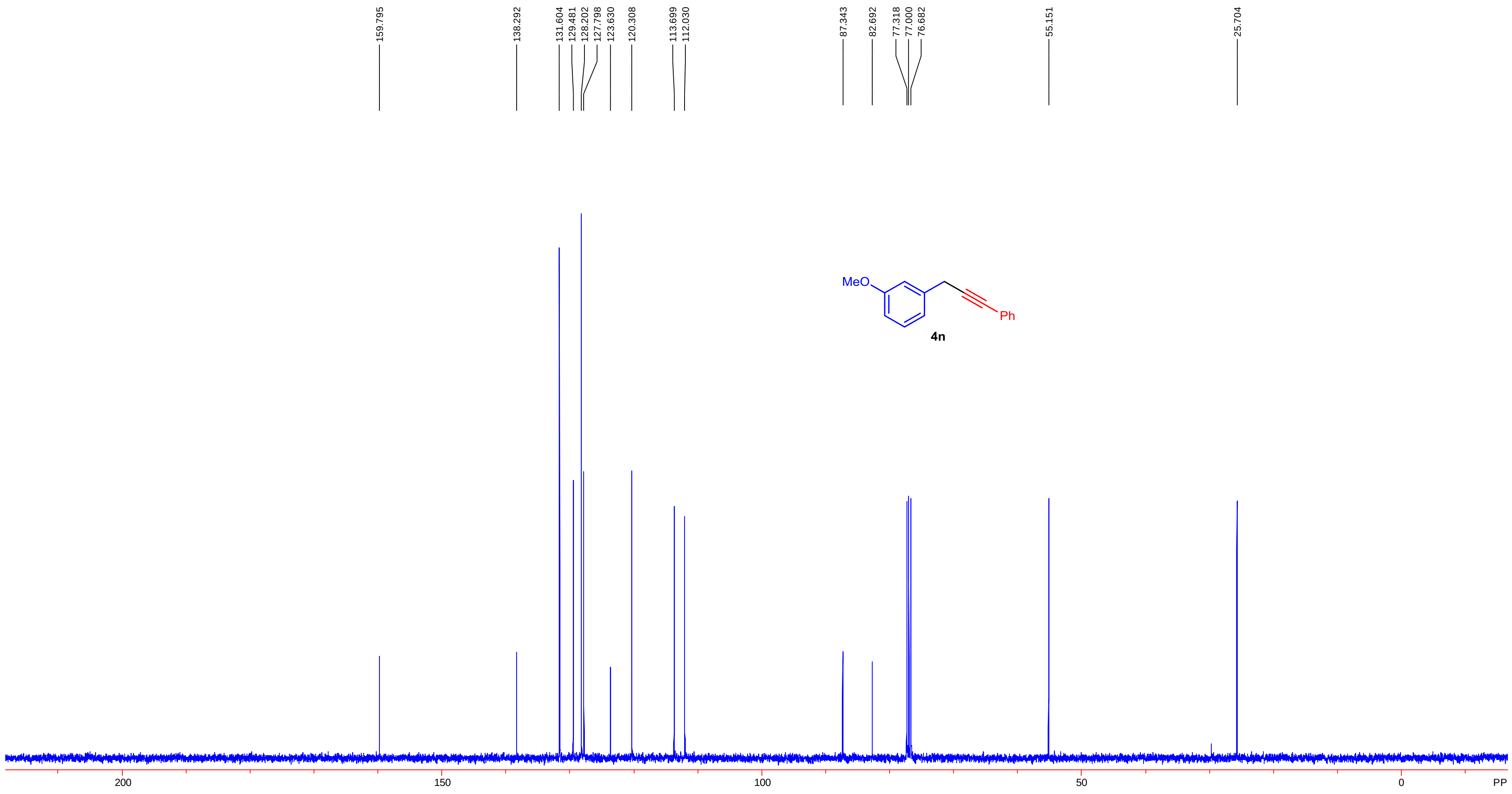

Supplementary Figure 123. ^{1}H NMR(400 MHz, $CDCl_3$)

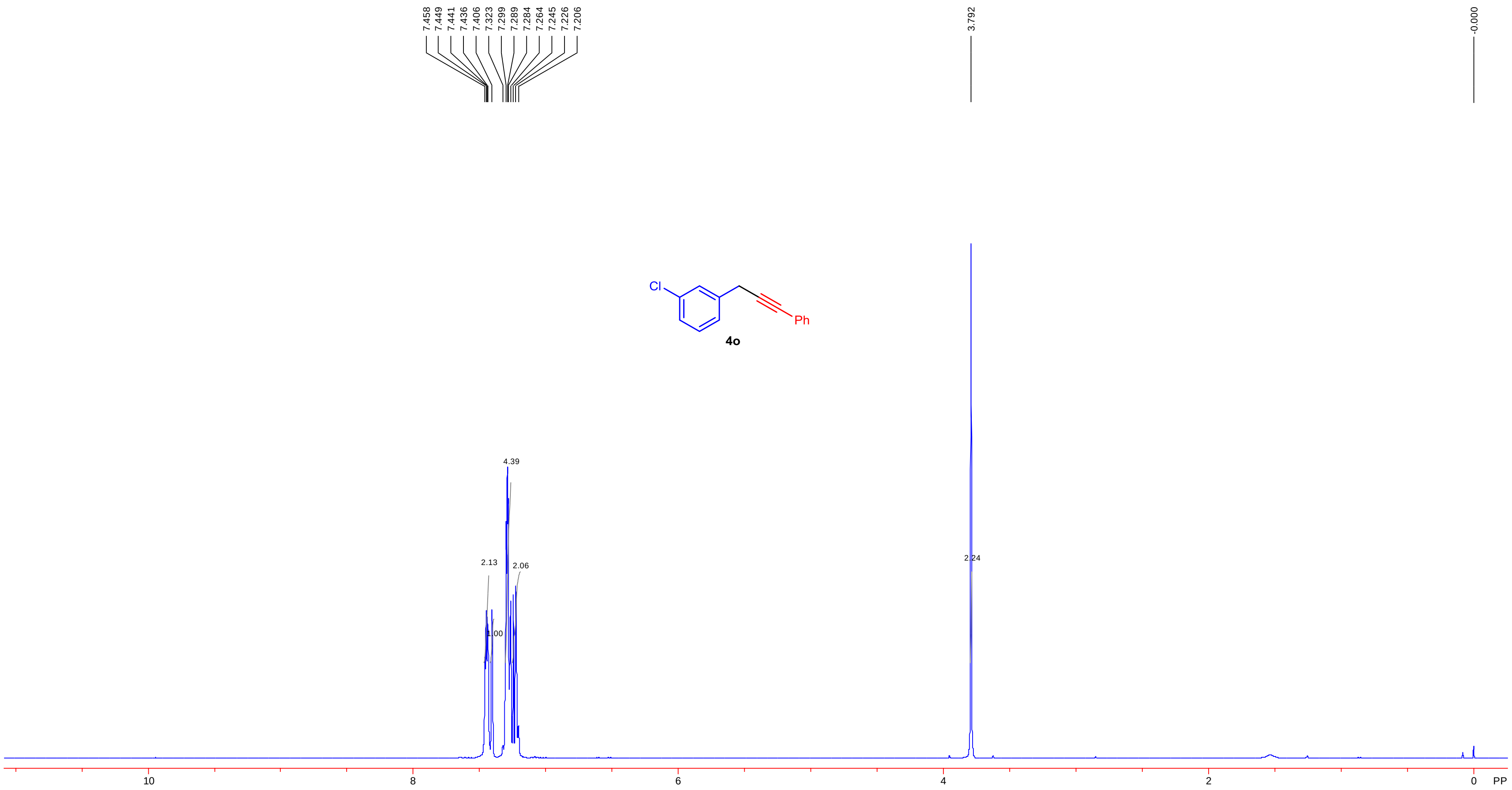

Supplementary Figure 124. ^{13}C NMR(100 MHz, $CDCl_3$)

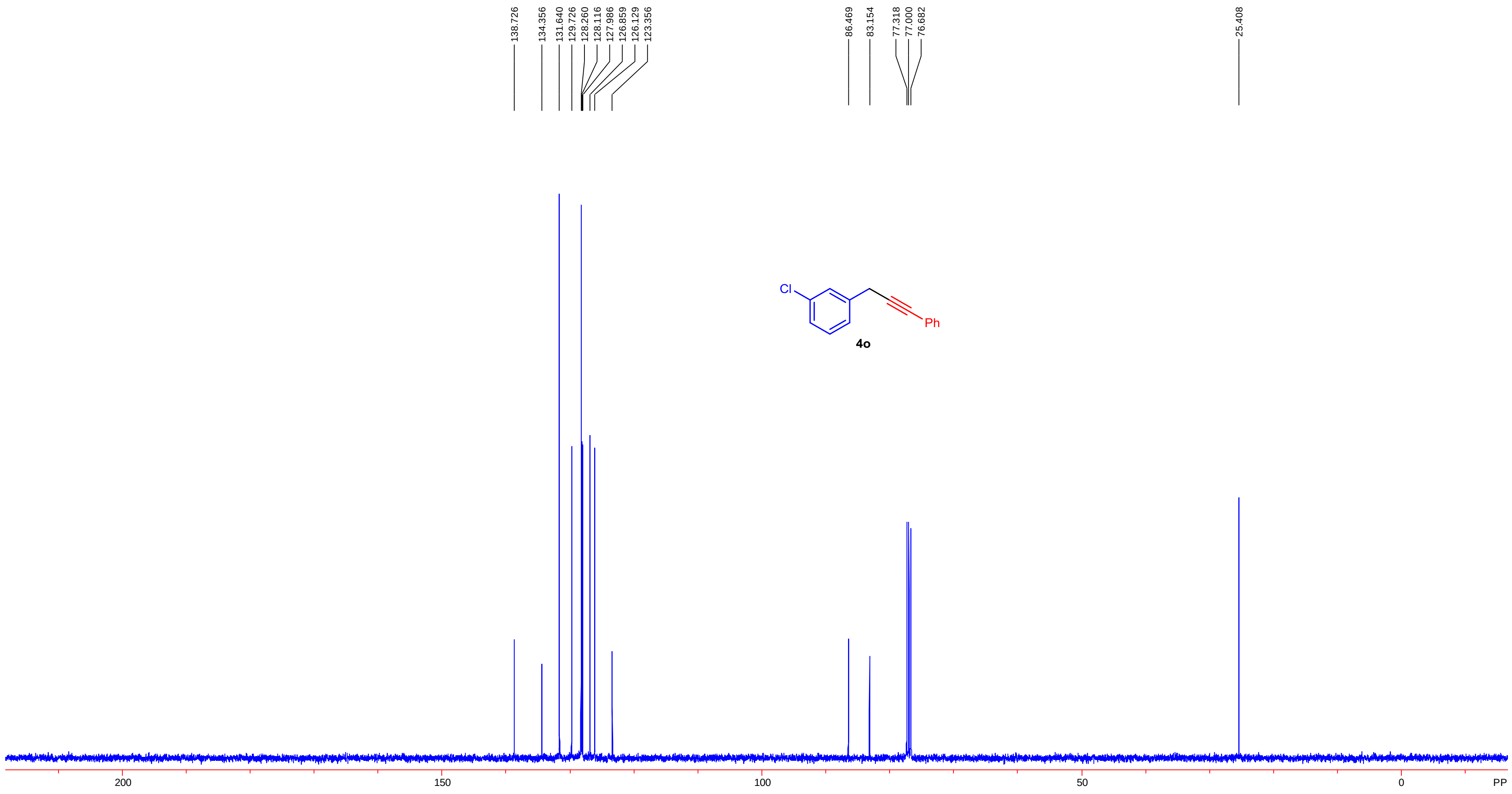

Supplementary Figure 125. ^{1}H NMR(400 MHz, $CDCl_3$)

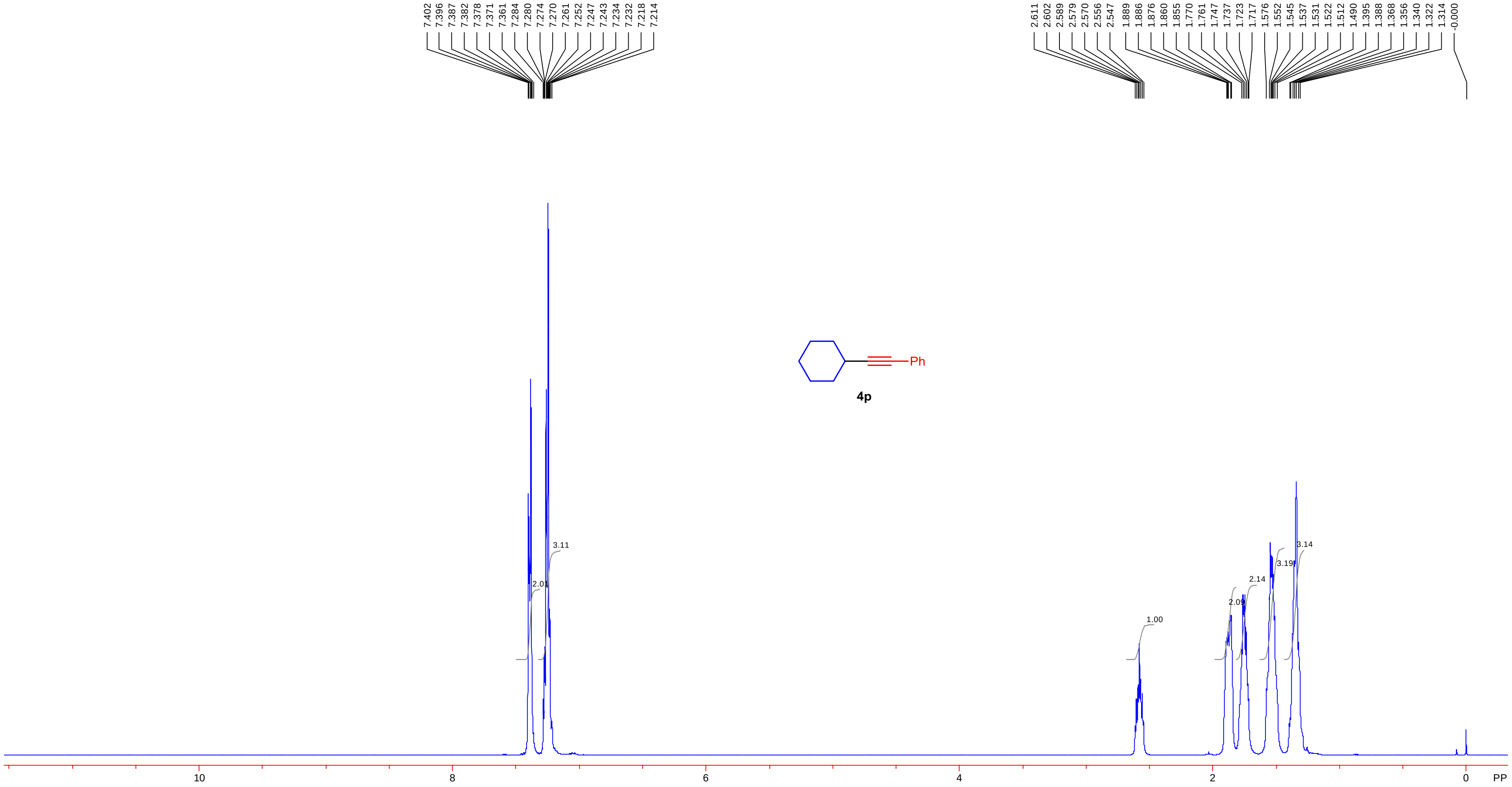

Supplementary Figure 126. ^{13}C NMR(100 MHz, $CDCl_3$)

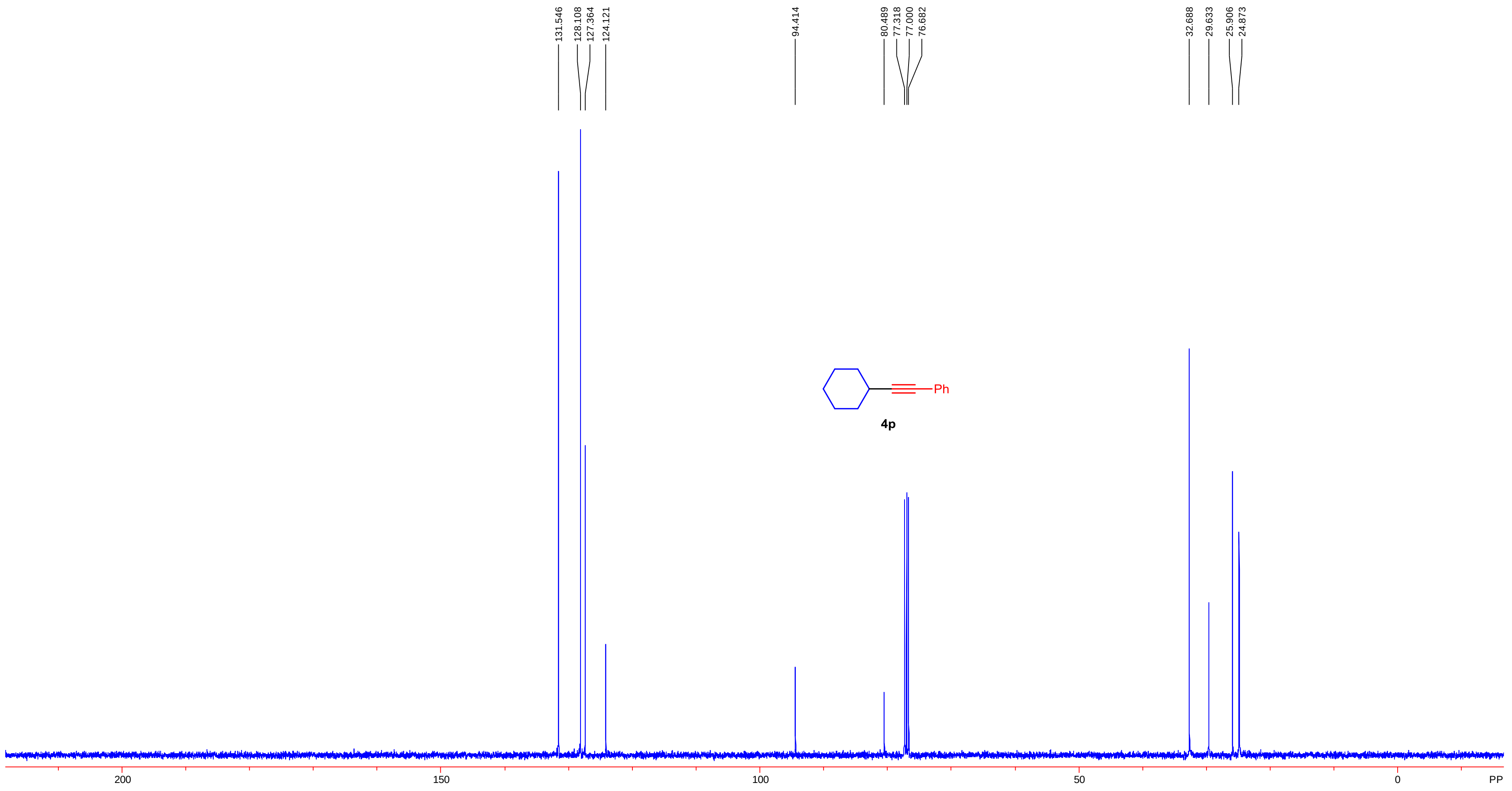

Supplementary Figure 127. ^{1}H NMR(400 MHz, $CDCl_3$)

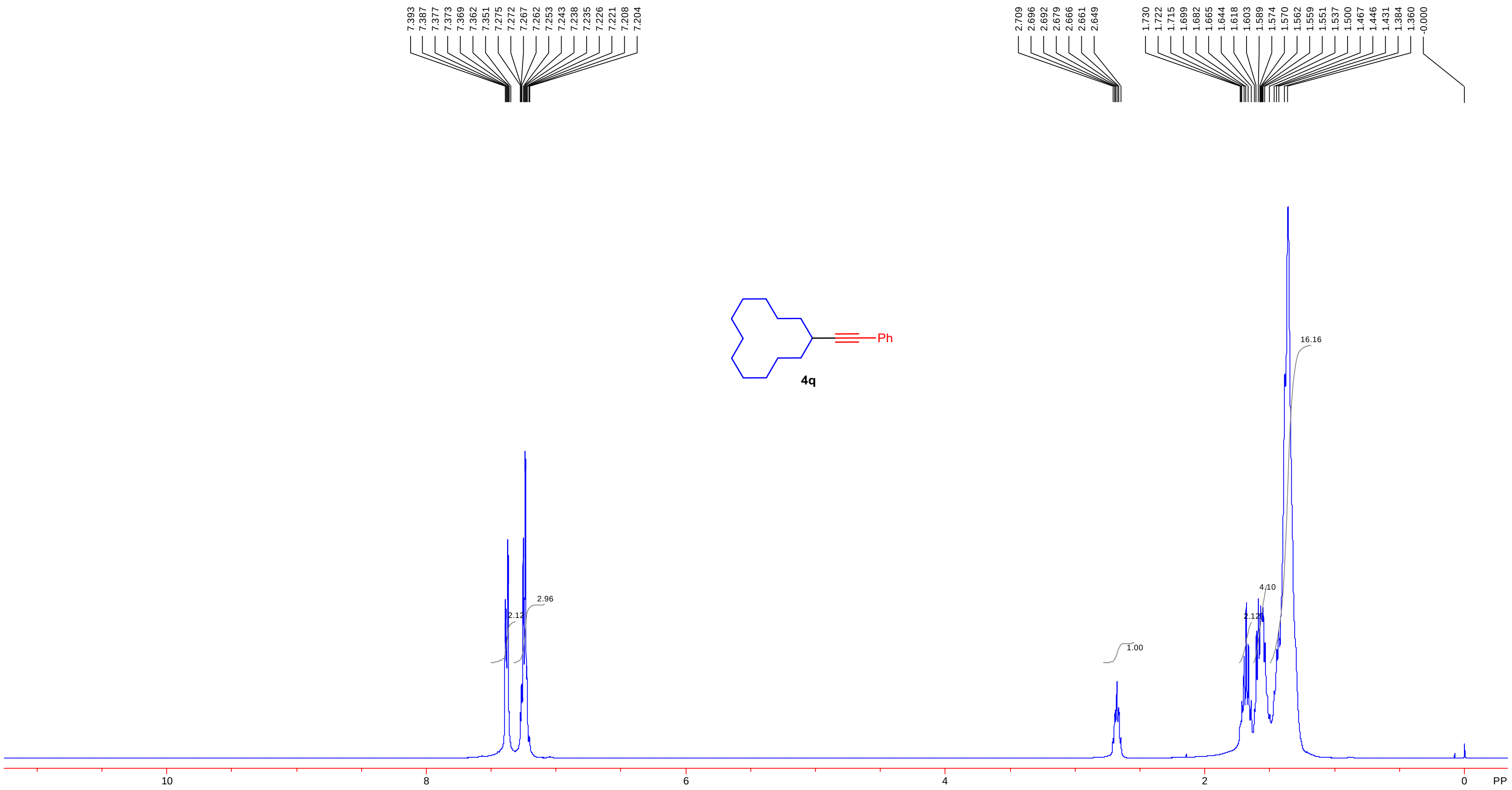

Supplementary Figure 128. ^{13}C NMR(100 MHz, $CDCl_3$)

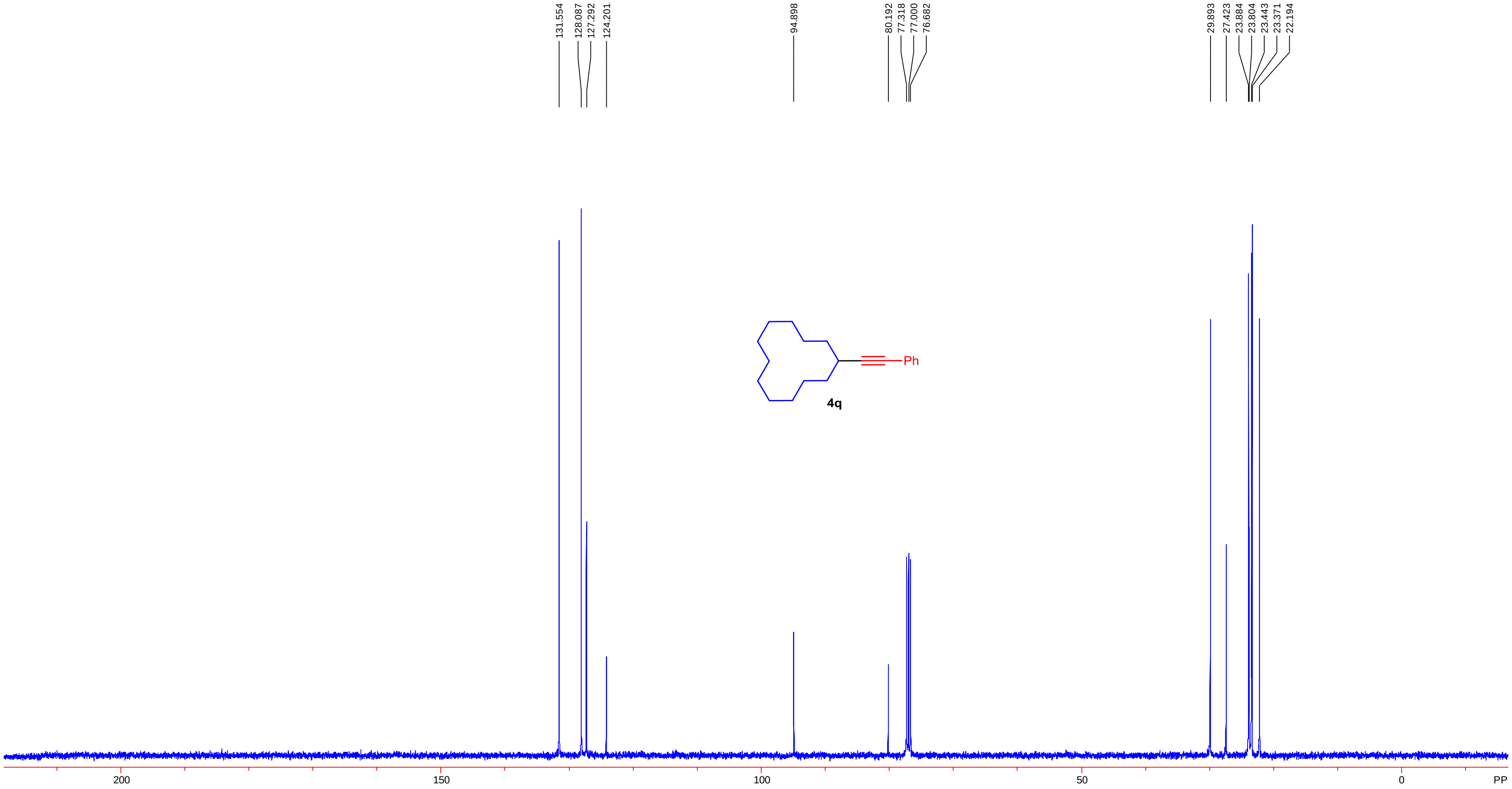

Supplementary Figure 129. ^{1}H NMR (600 MHz, $CDCl_3$)

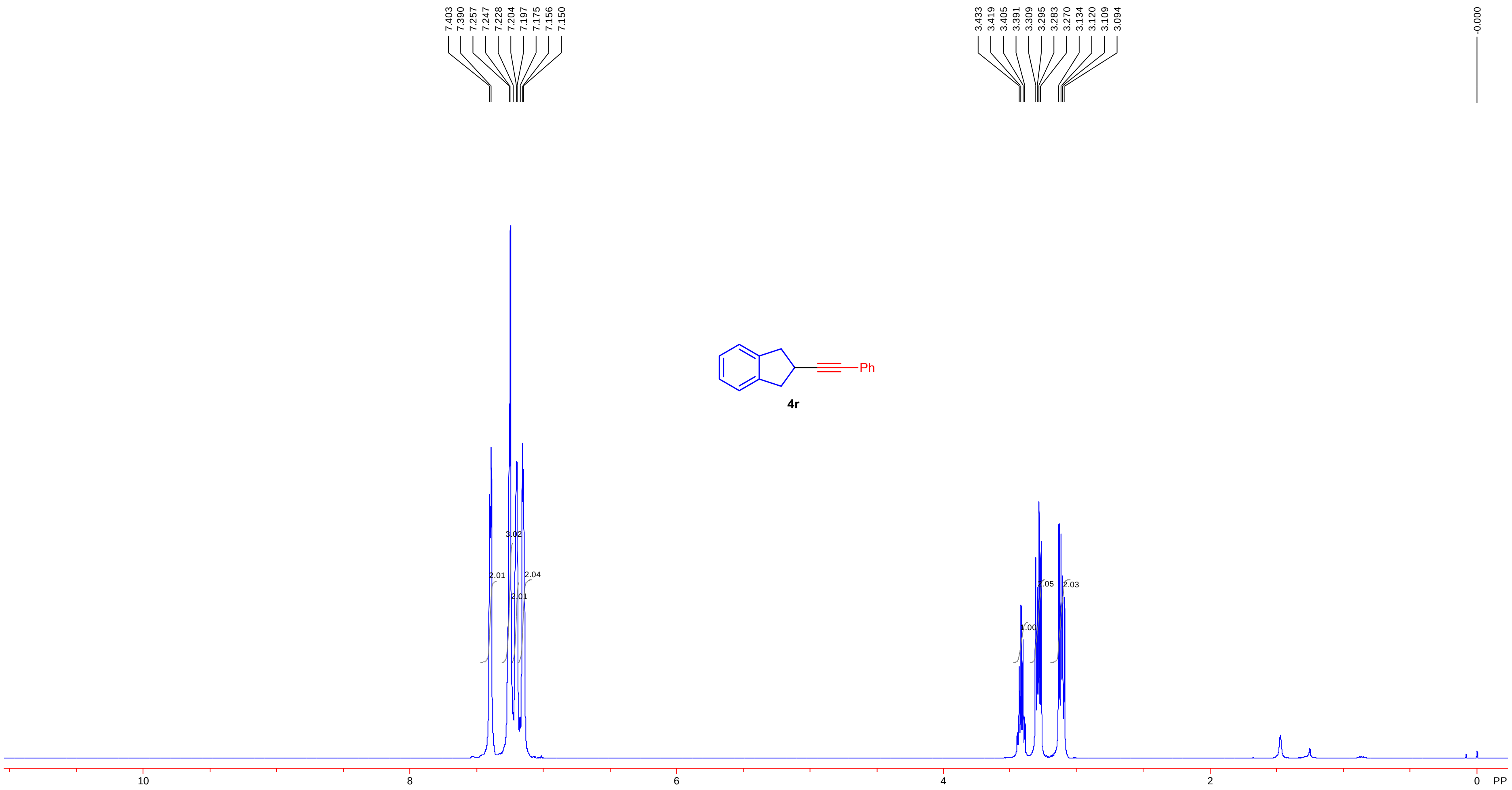

Supplementary Figure 130. ^{13}C NMR (151 MHz, $CDCl_3$)

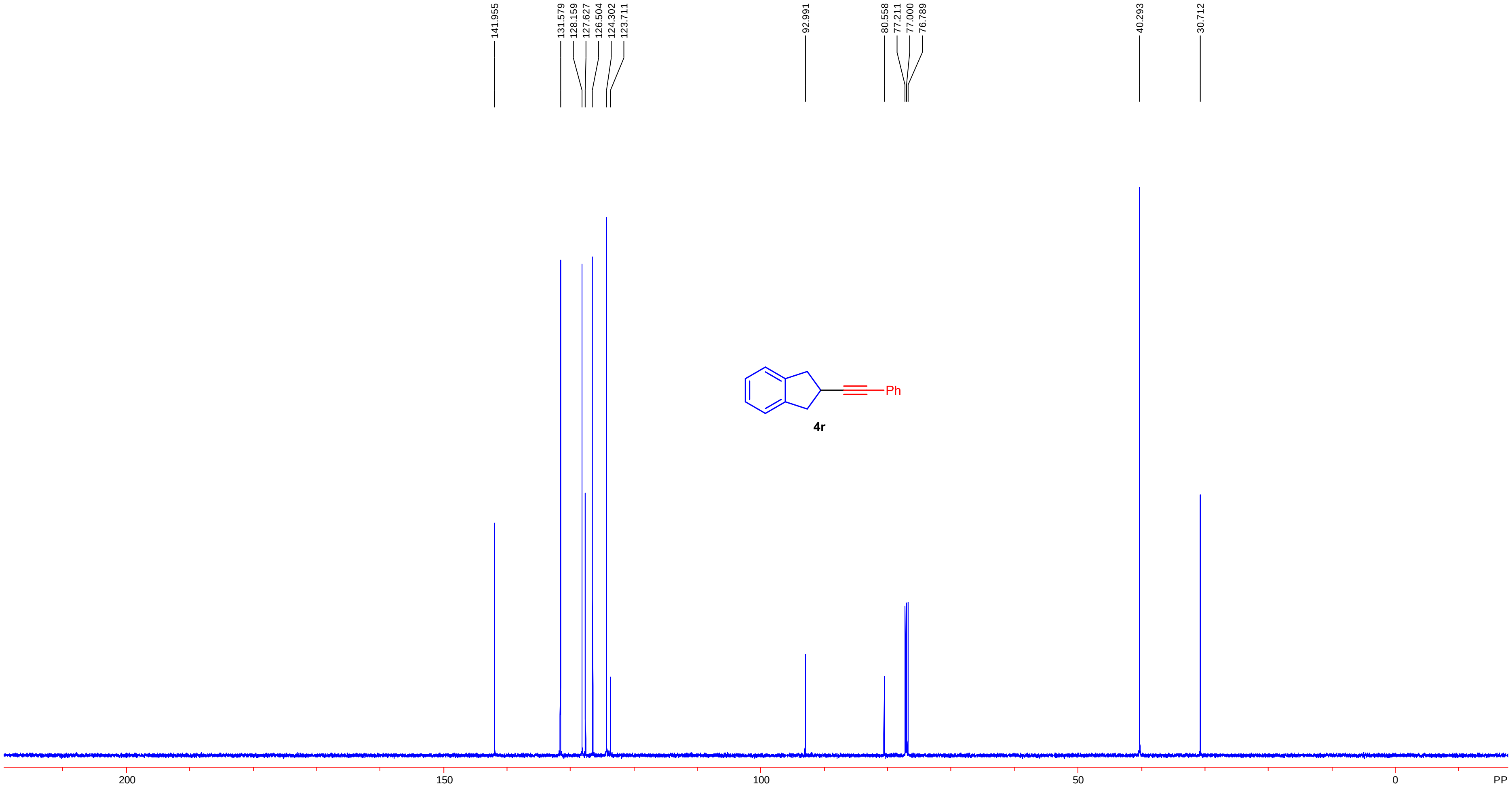

Supplementary Figure 131. ^{1}H NMR (600 MHz, $CDCl_3$)

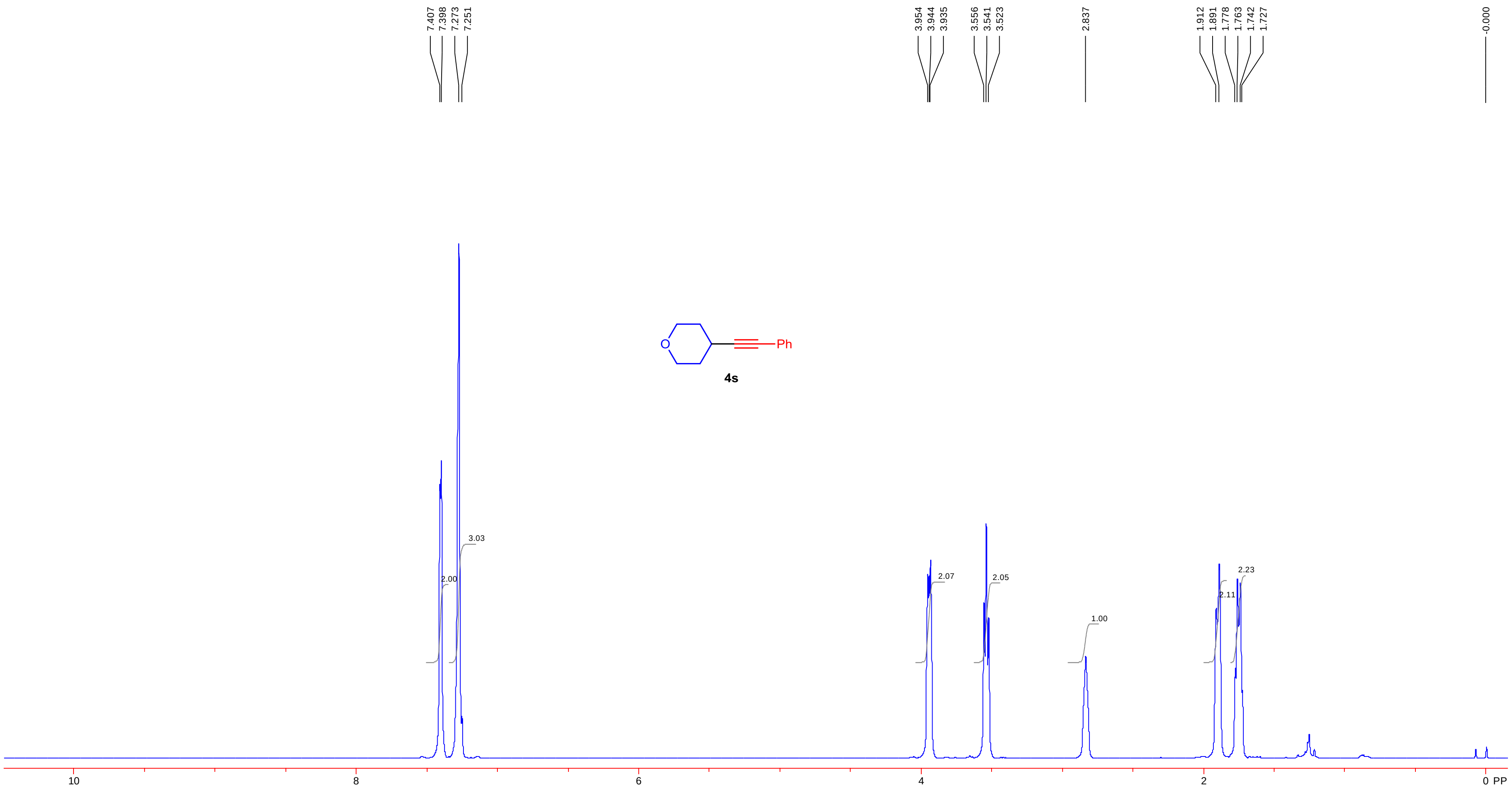

Supplementary Figure 132. ^{13}C NMR (151 MHz, $CDCl_3$)

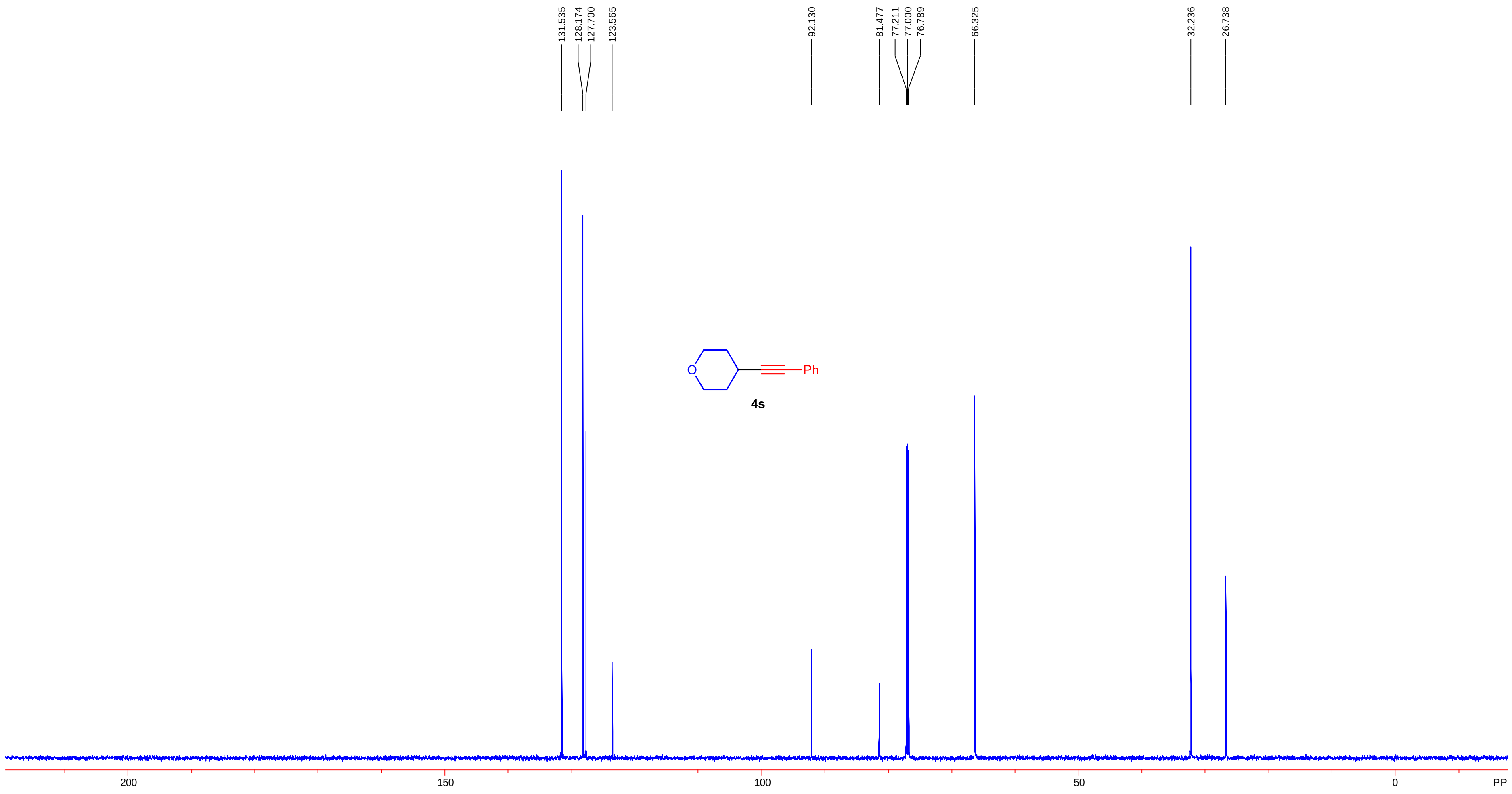

Supplementary Figure 133. ^{1}H NMR (600 MHz, $CDCl_3$)

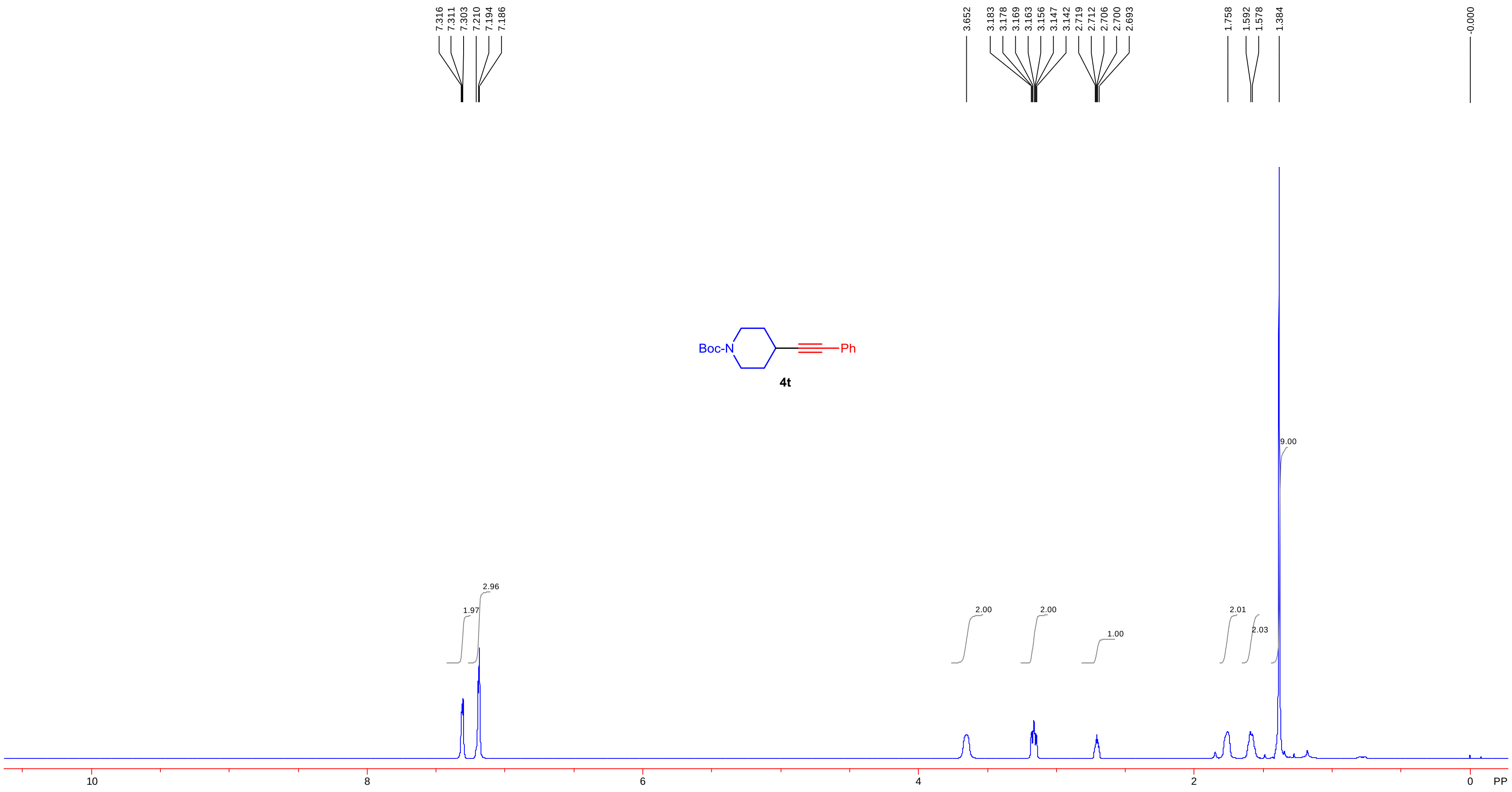

Supplementary Figure 134. ^{13}C NMR (151 MHz, $CDCl_3$)

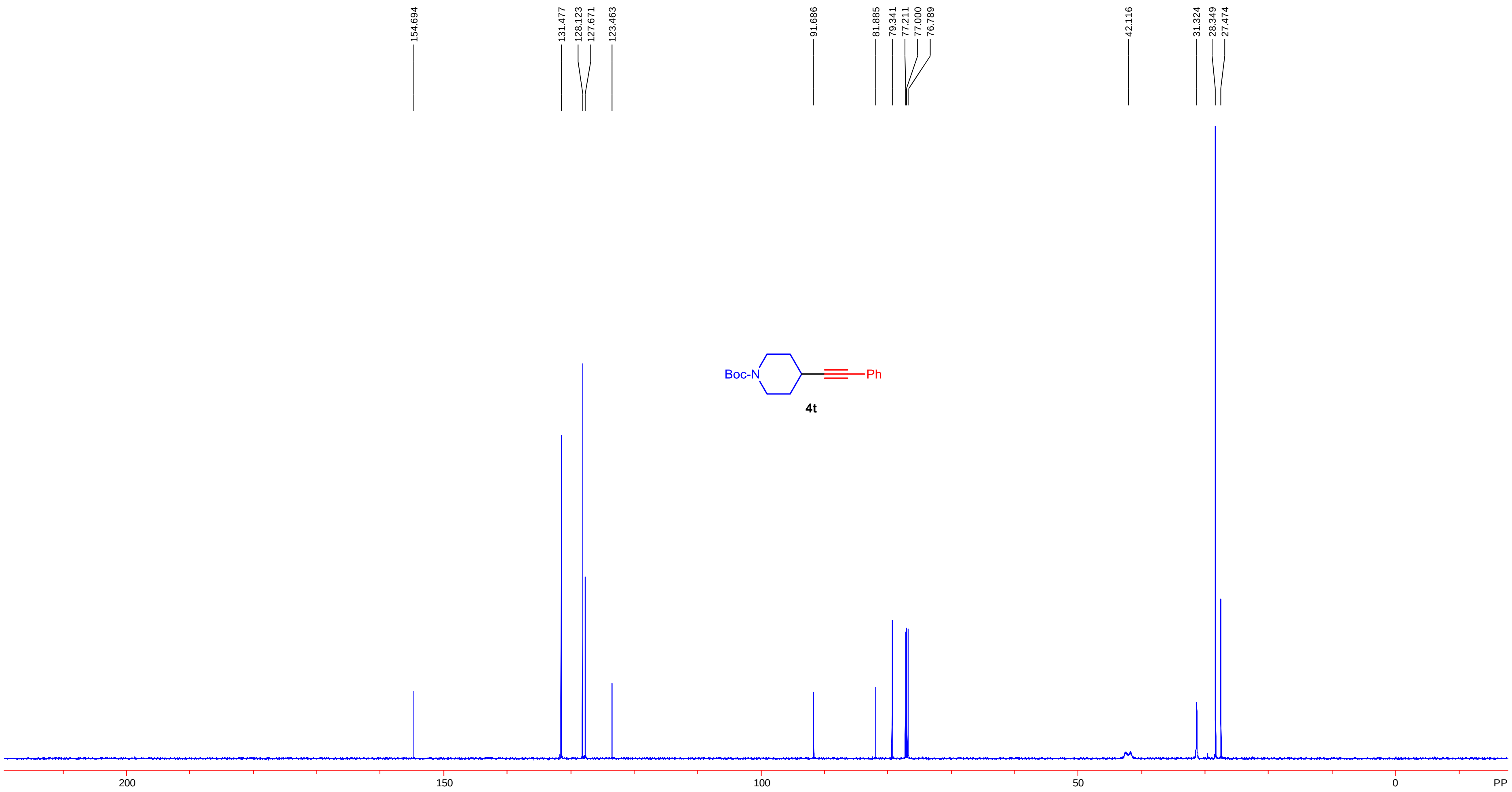

Supplementary Figure 135. ^{1}H NMR (600 MHz, $CDCl_3$)

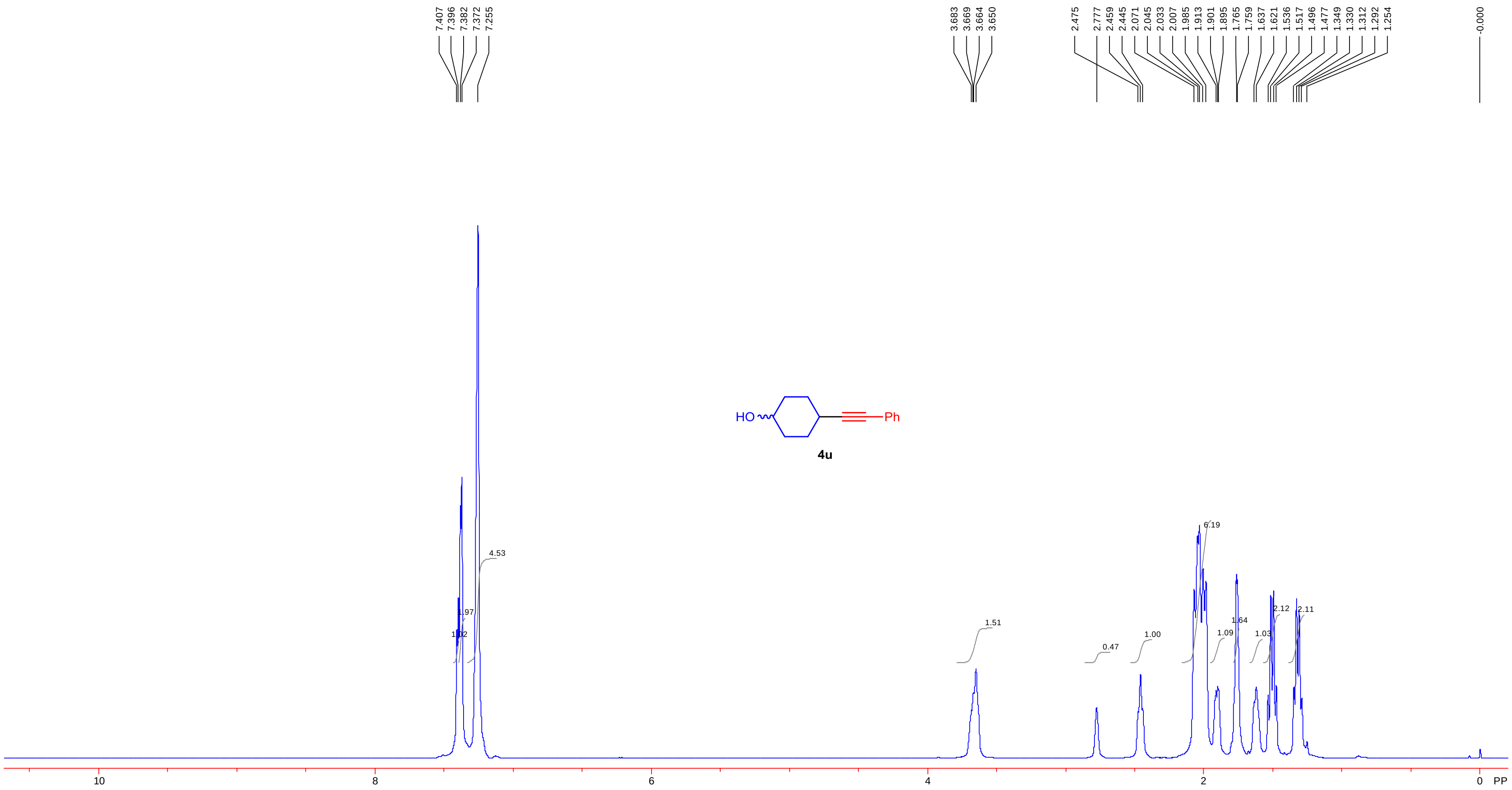

Supplementary Figure 136. ^{13}C NMR (151 MHz, $CDCl_3$)

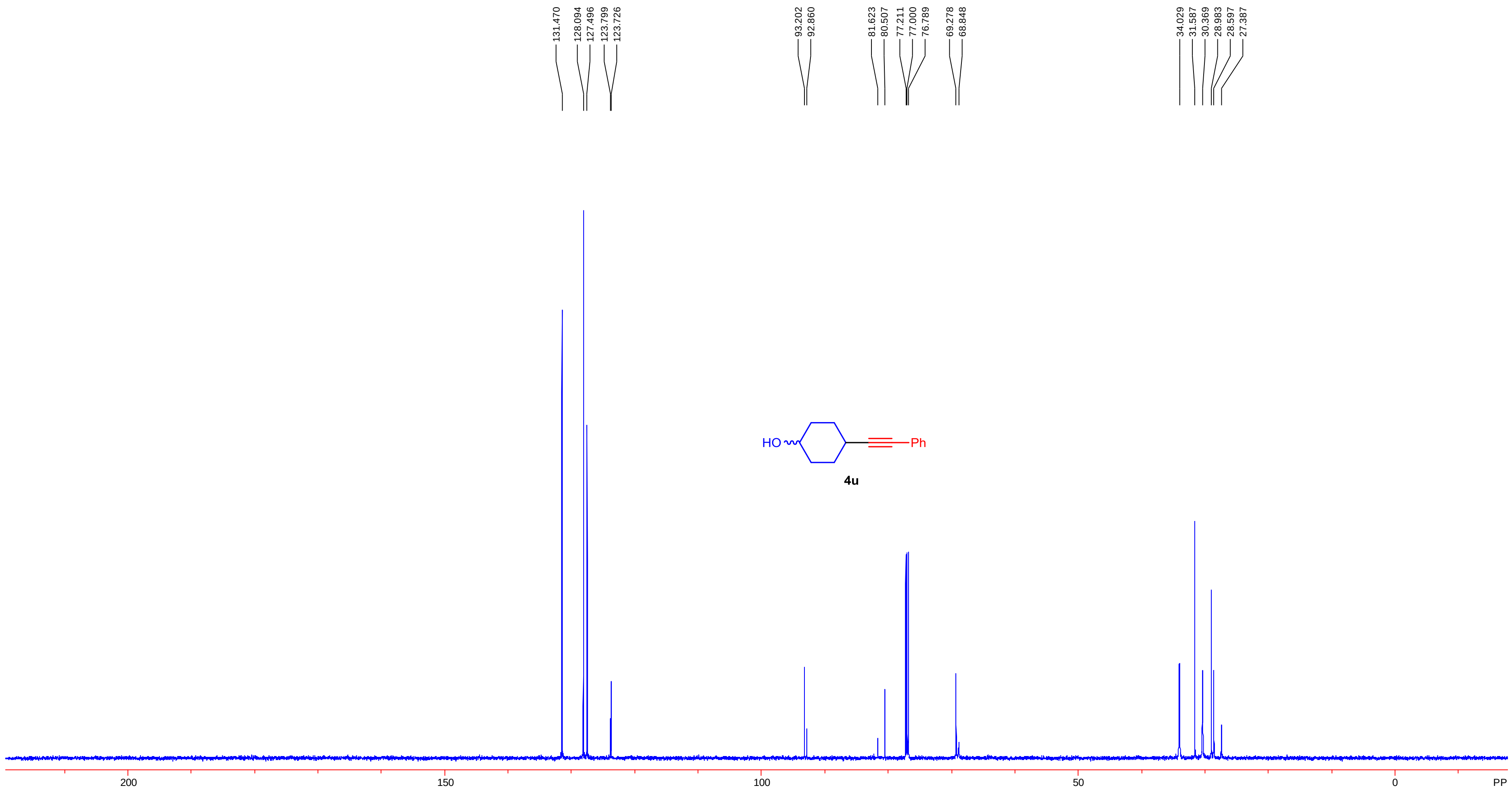

Supplementary Figure 137. ^{1}H NMR(400 MHz, $CDCl_3$)

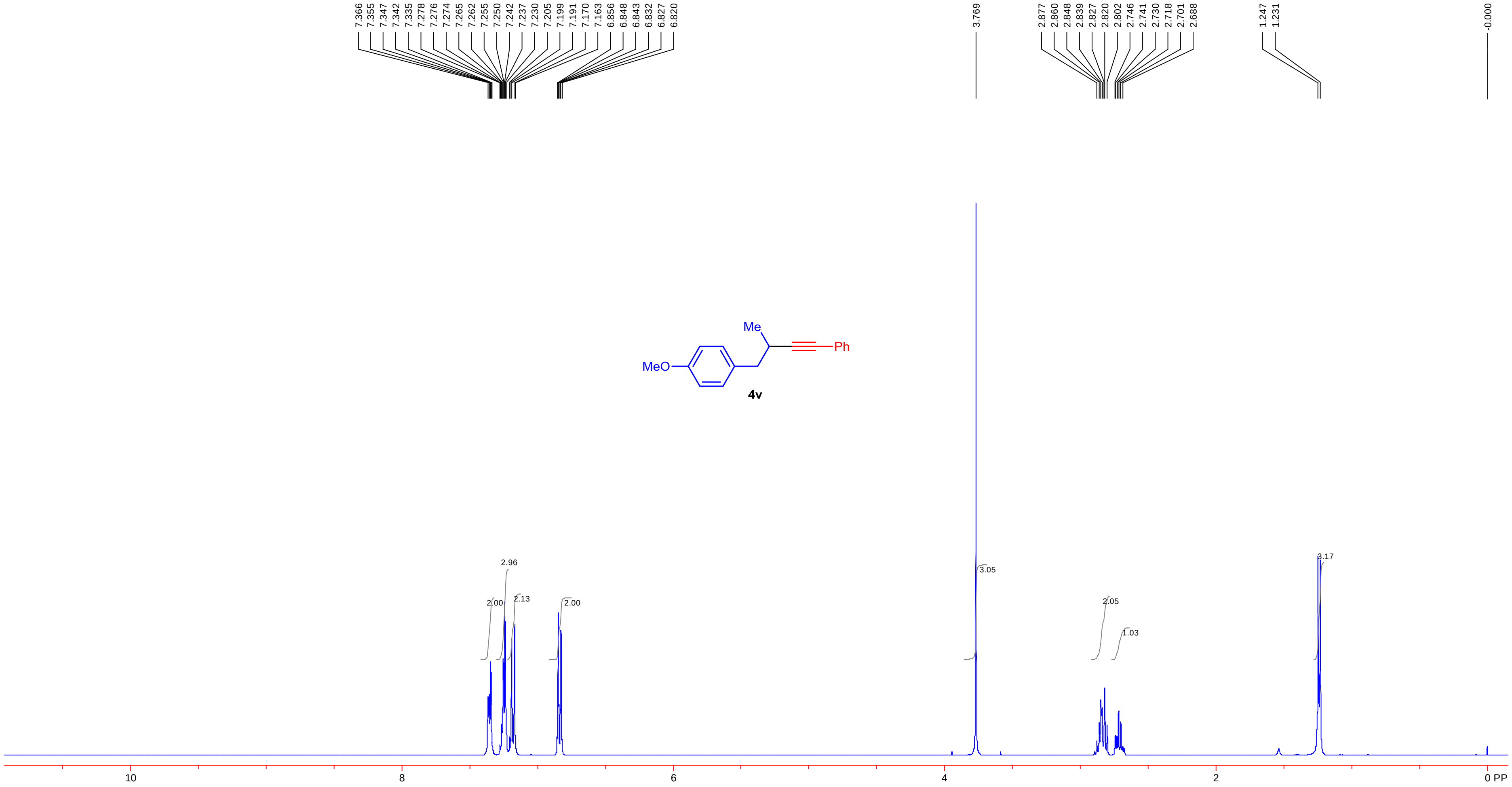

Supplementary Figure 138. ^{13}C NMR(100 MHz, $CDCl_3$)

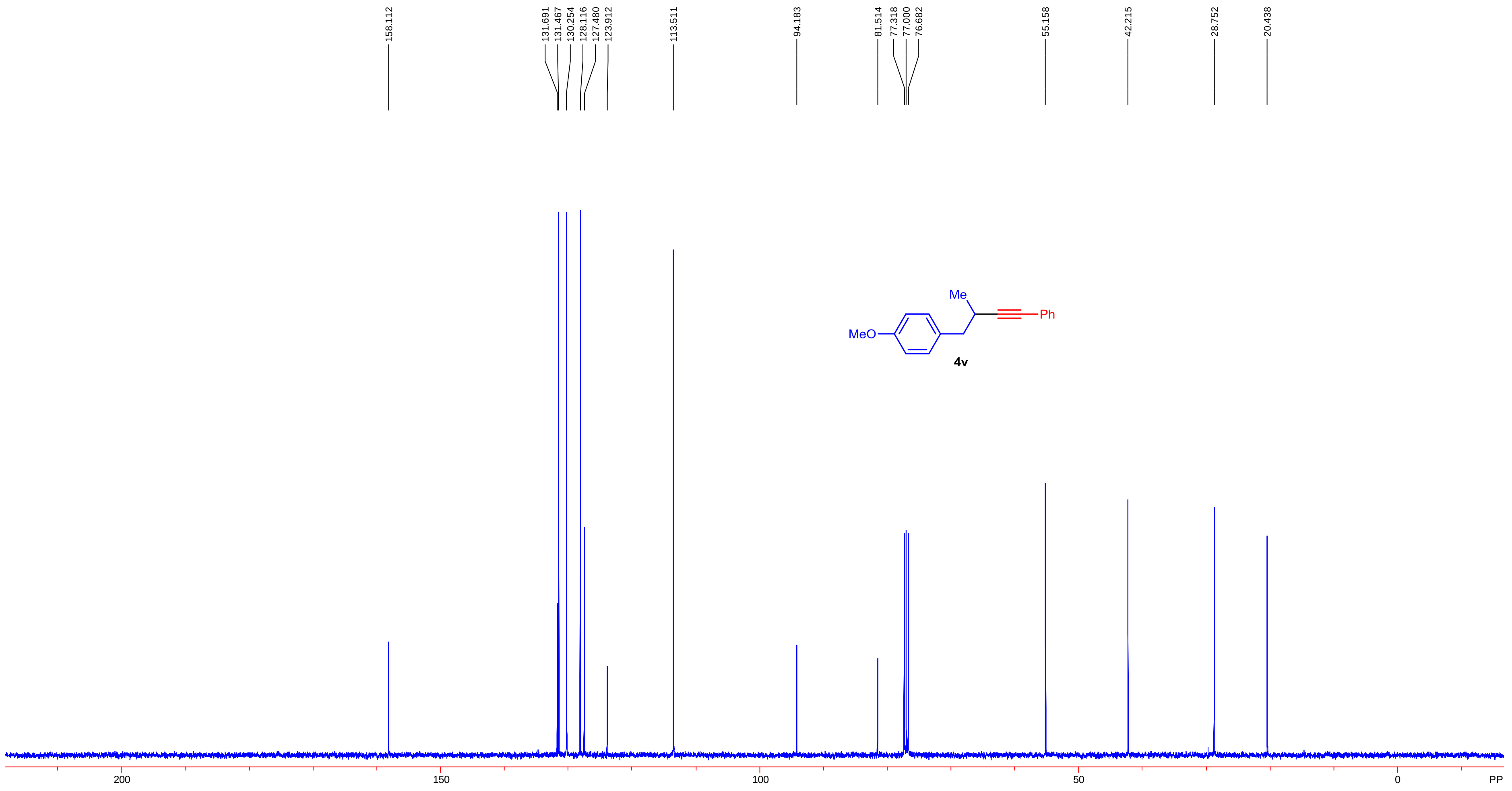

Supplementary Figure 139. ^{1}H NMR(400 MHz, $CDCl_3$)

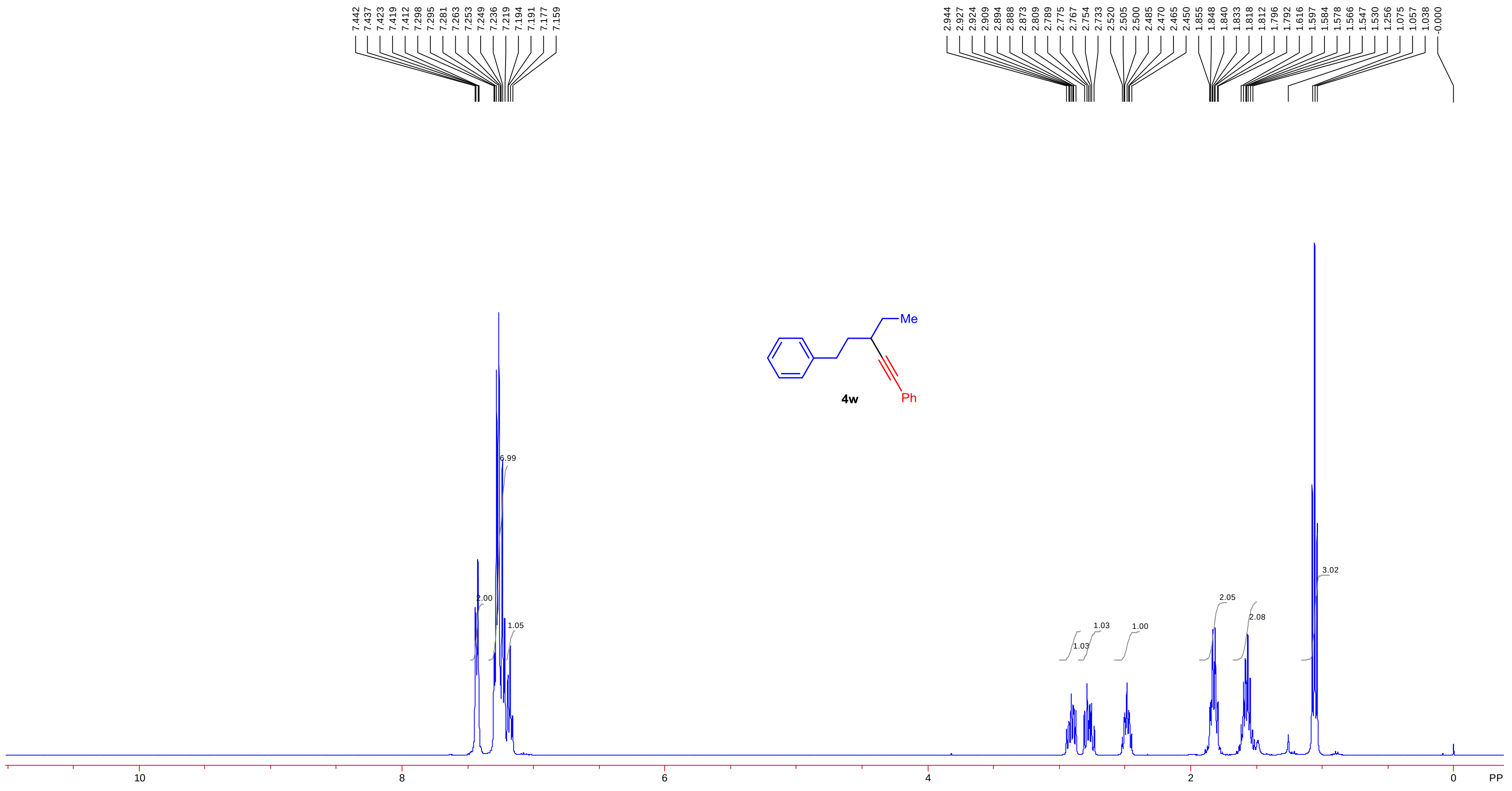

Supplementary Figure 140. ^{13}C NMR(100 MHz, $CDCl_3$)

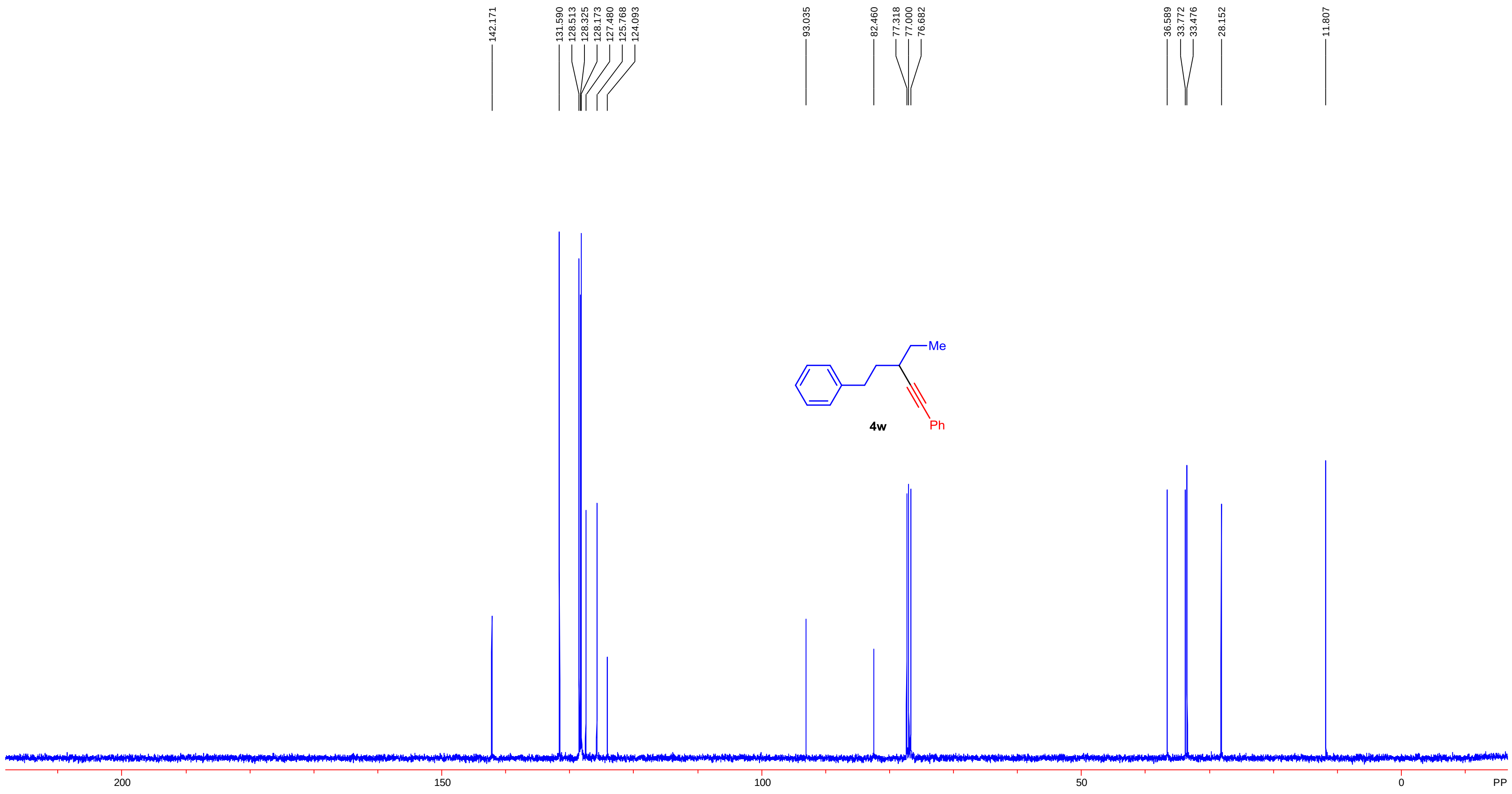

Supplementary Figure 141. ^{1}H NMR(400 MHz, $CDCl_3$)

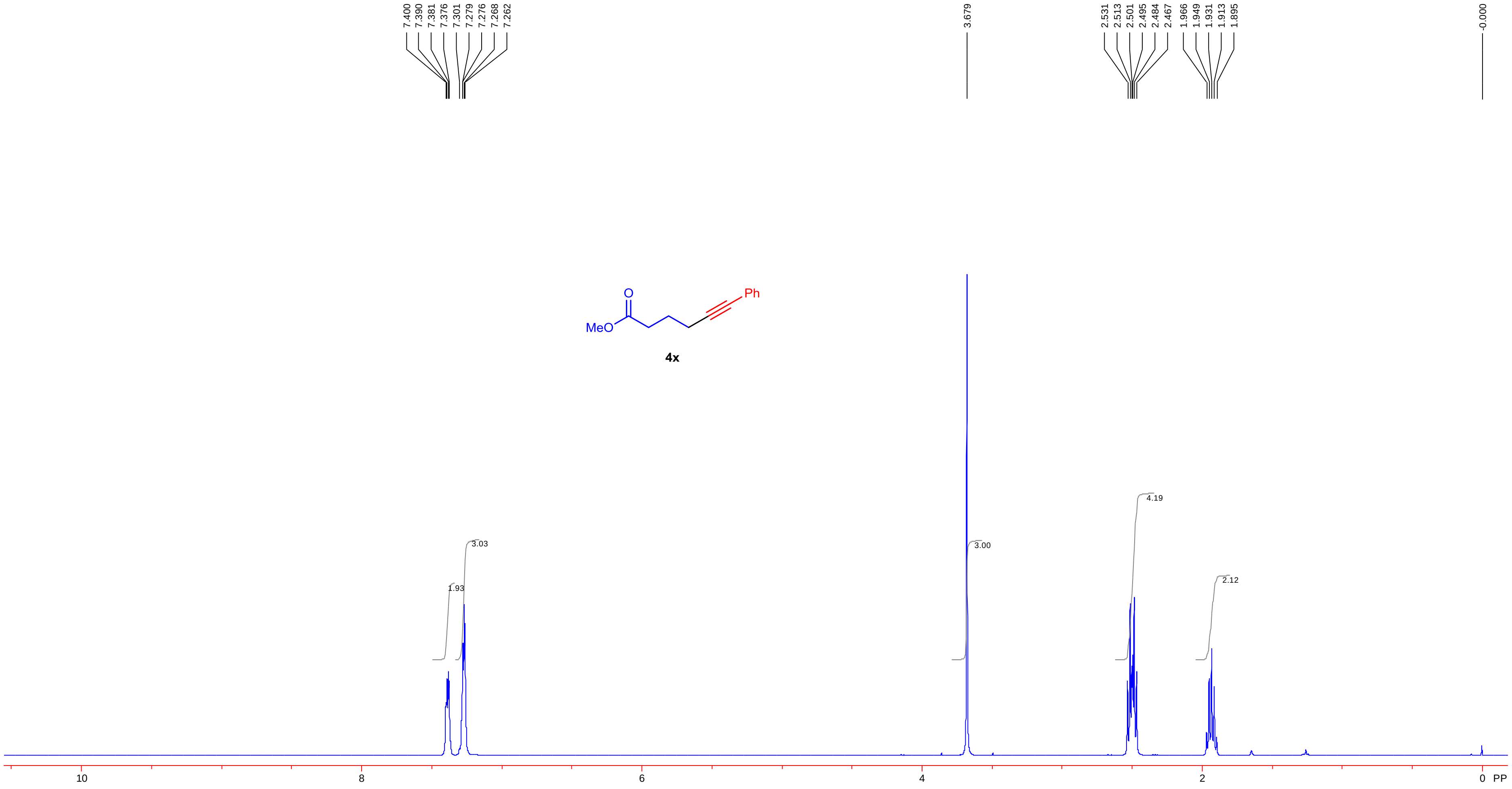

Supplementary Figure 142. ^{13}C NMR(100 MHz, $CDCl_3$)

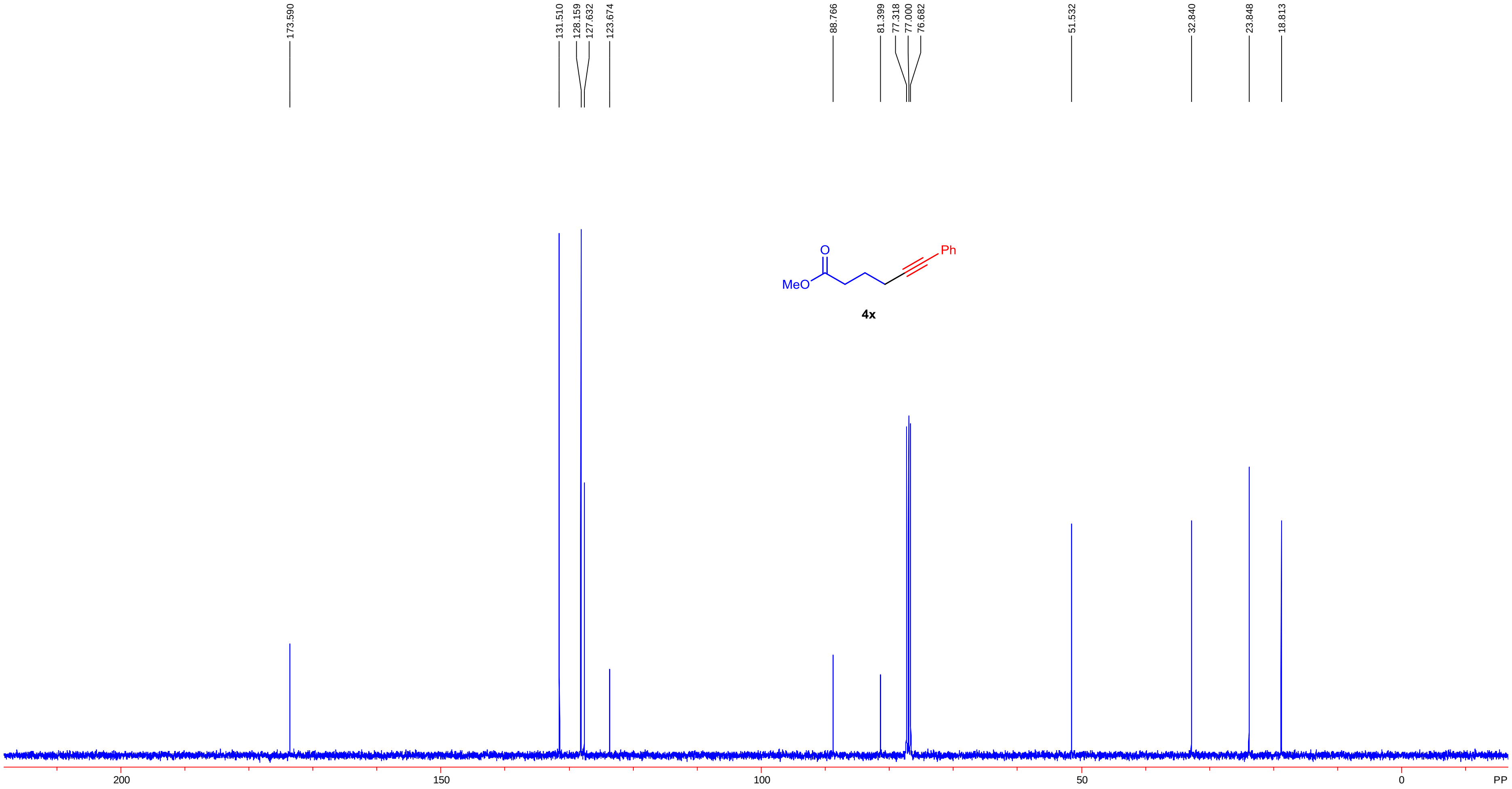

Supplementary Figure 143. ^{1}H NMR (600 MHz, $CDCl_3$)

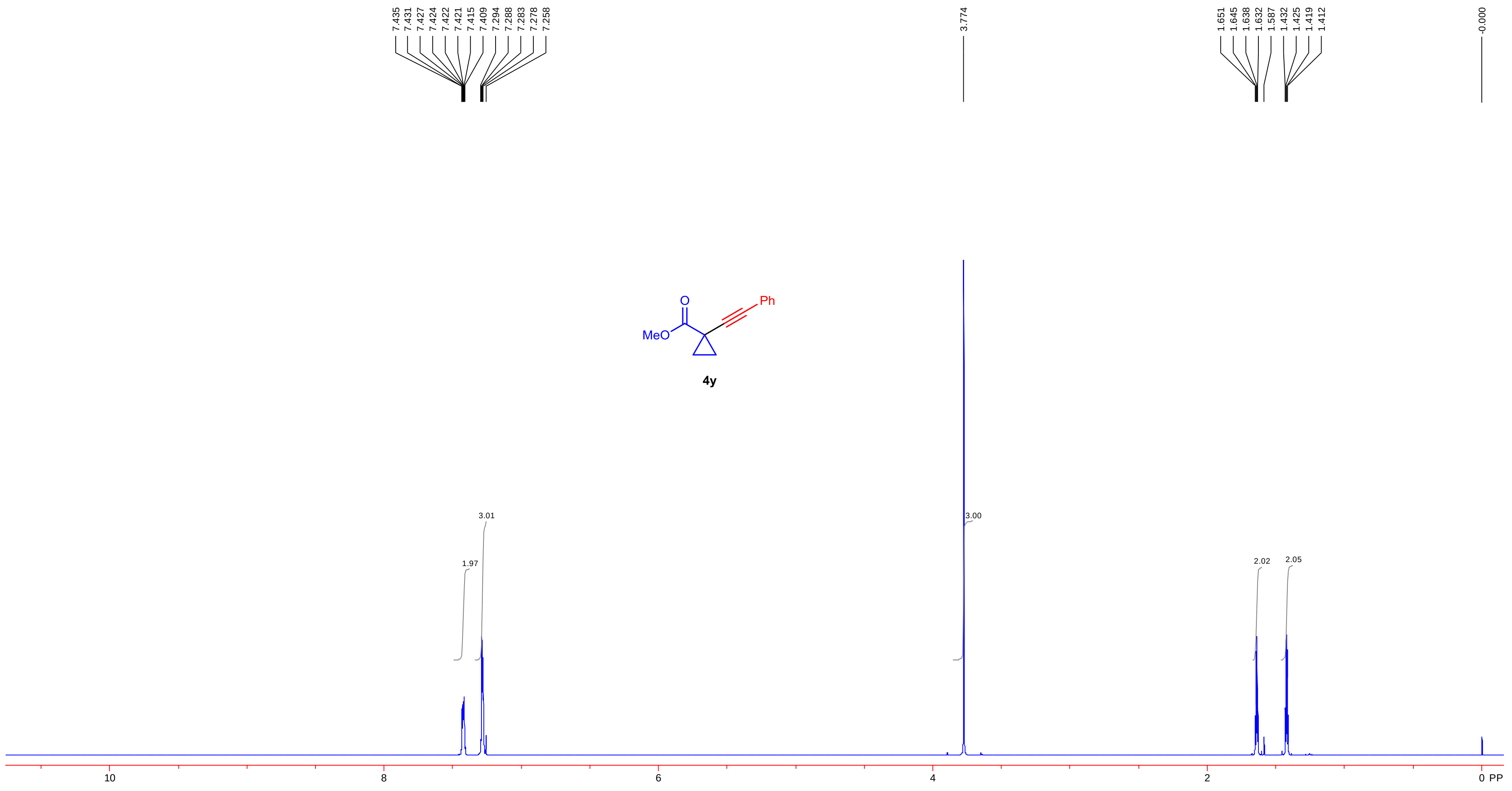

Supplementary Figure 144. ^{13}C NMR (151 MHz, $CDCl_3$)

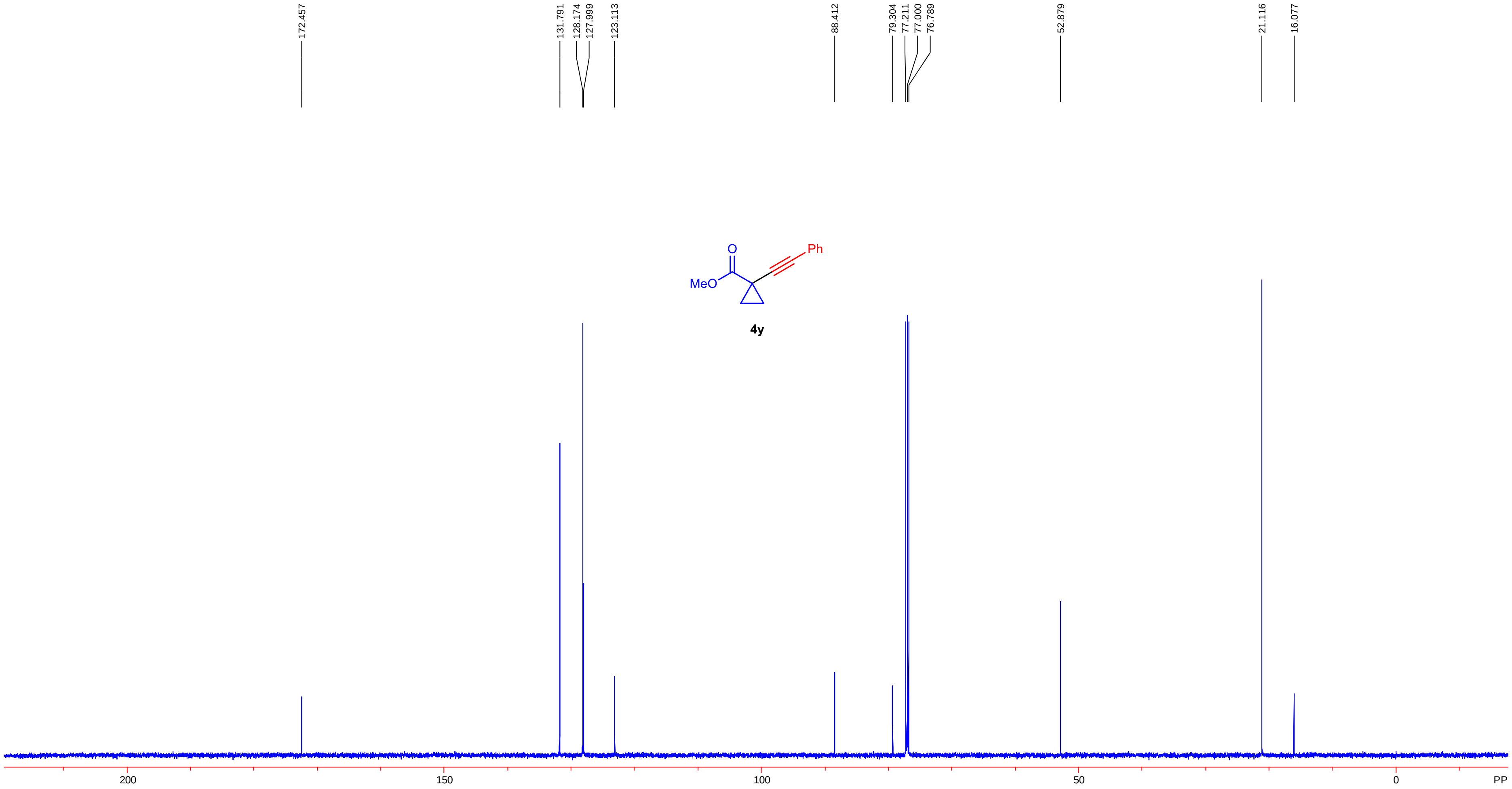

Supplementary Figure 145. ^{1}H NMR (600 MHz, $CDCl_3$)

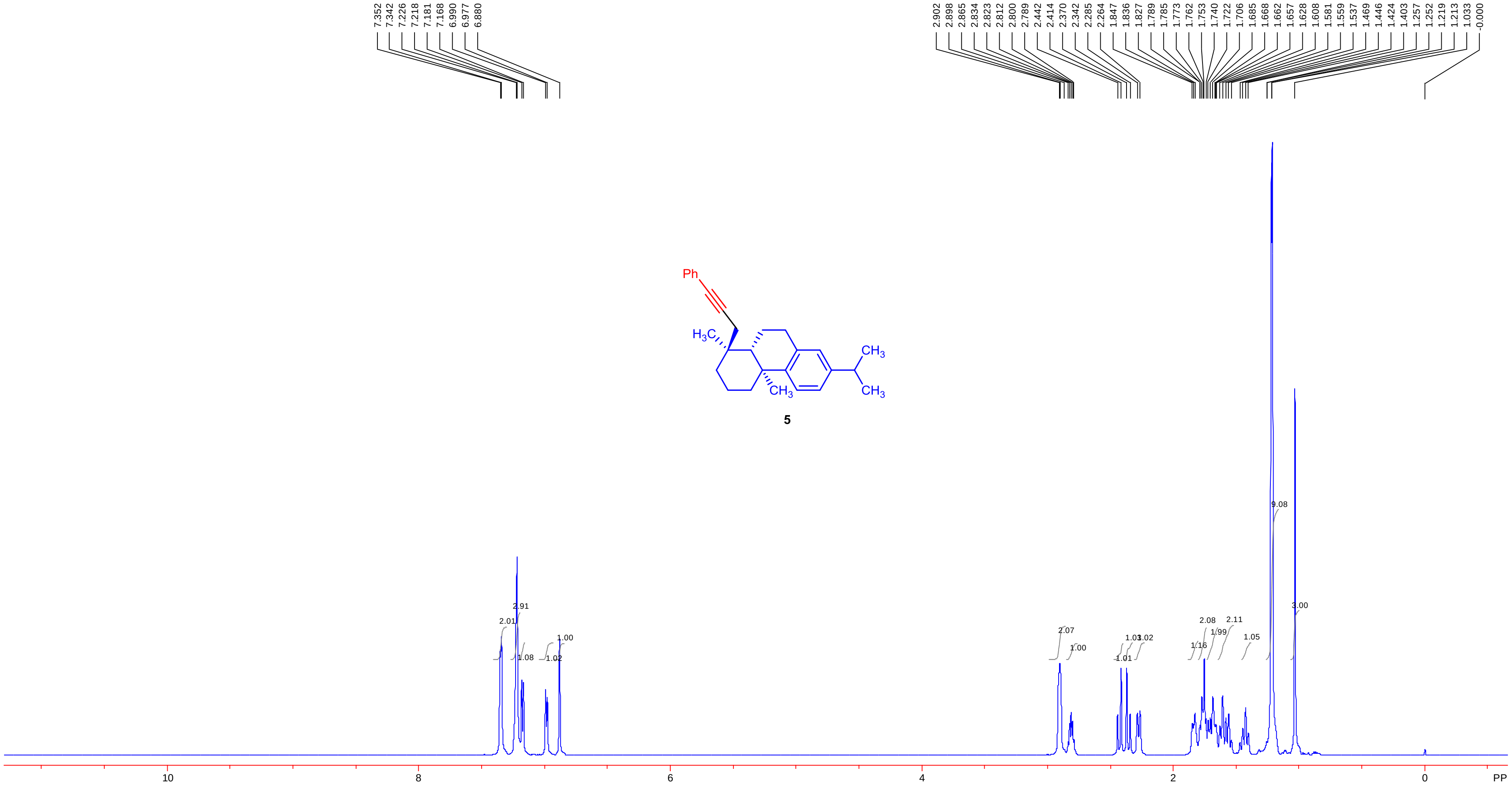

Supplementary Figure 146. ^{13}C NMR (151 MHz, $CDCl_3$)

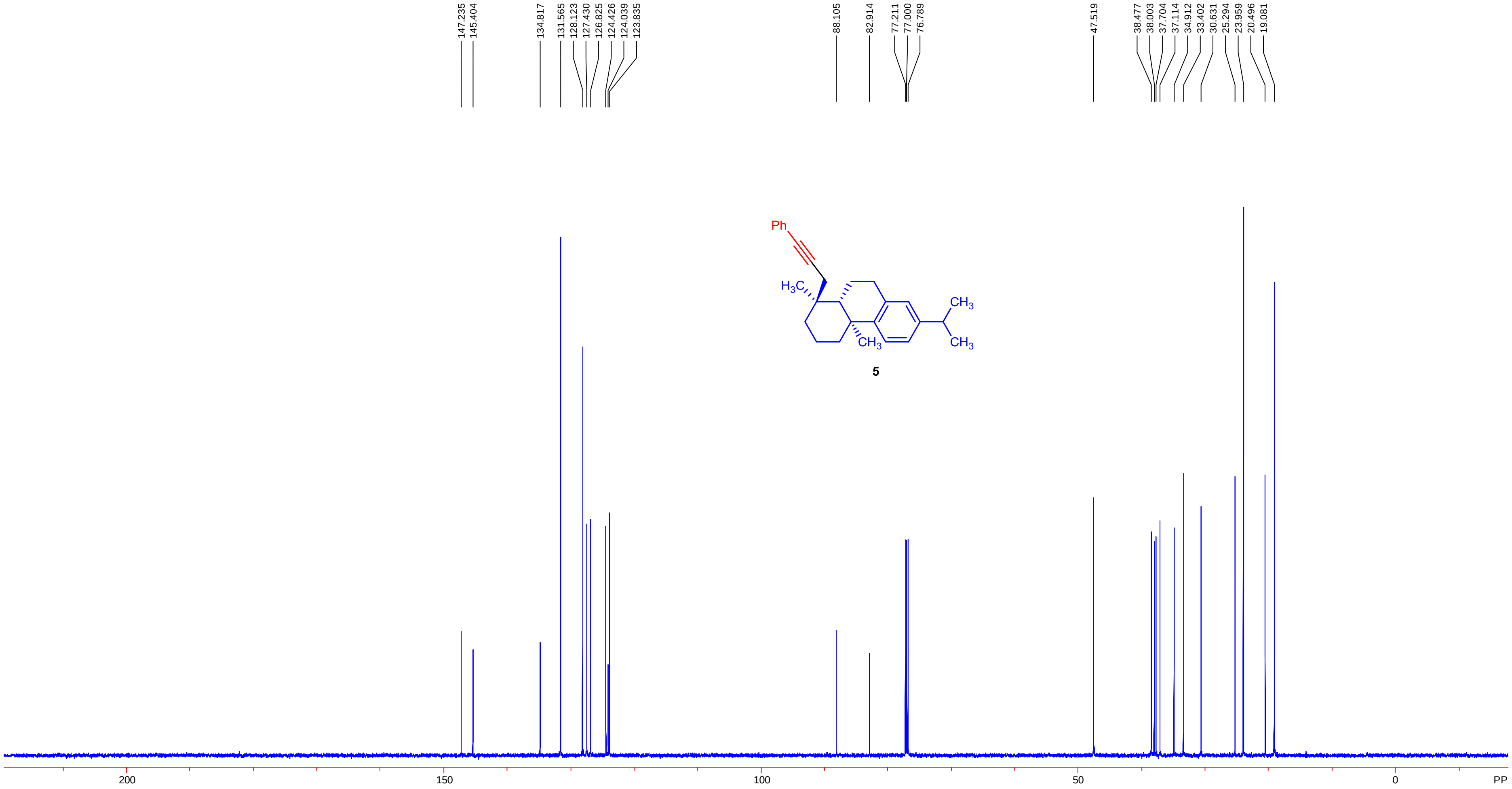

Supplementary Figure 147. ^{1}H NMR (600 MHz, $CDCl_3$)

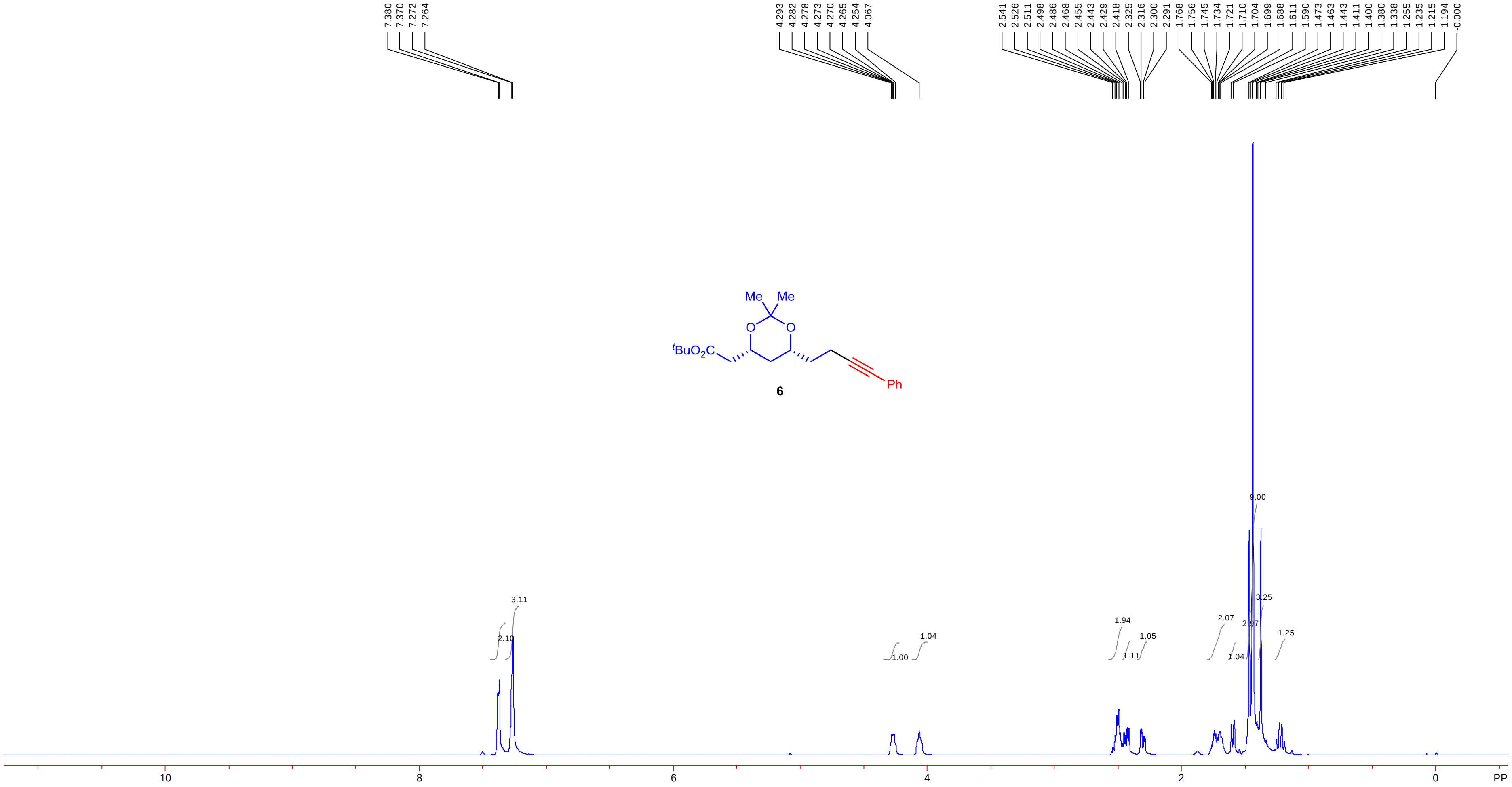

Supplementary Figure 148. ^{13}C NMR (151 MHz, $CDCl_3$)

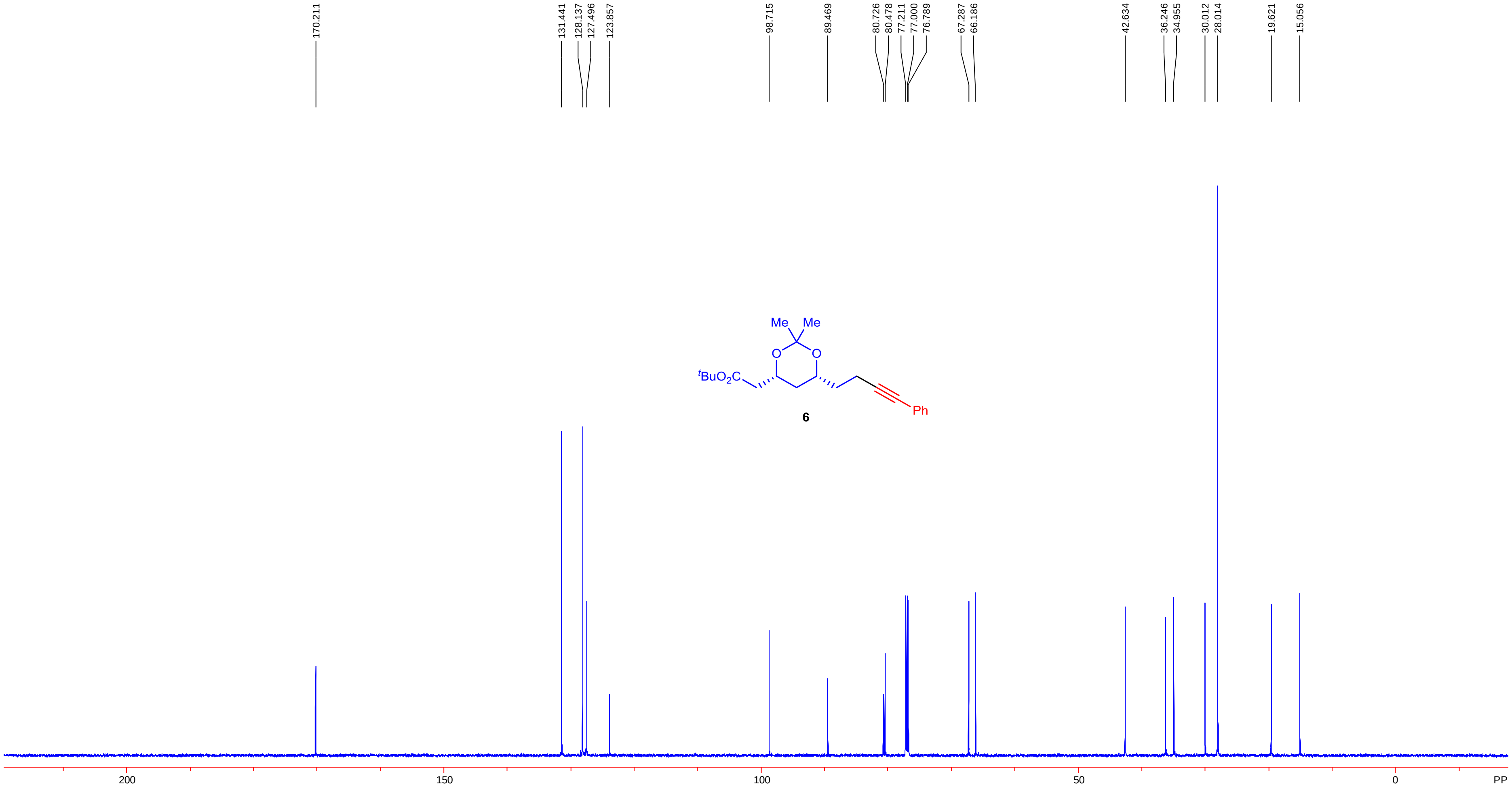

Supplementary Figure 149. ^{1}H NMR(400 MHz, $CDCl_3$)

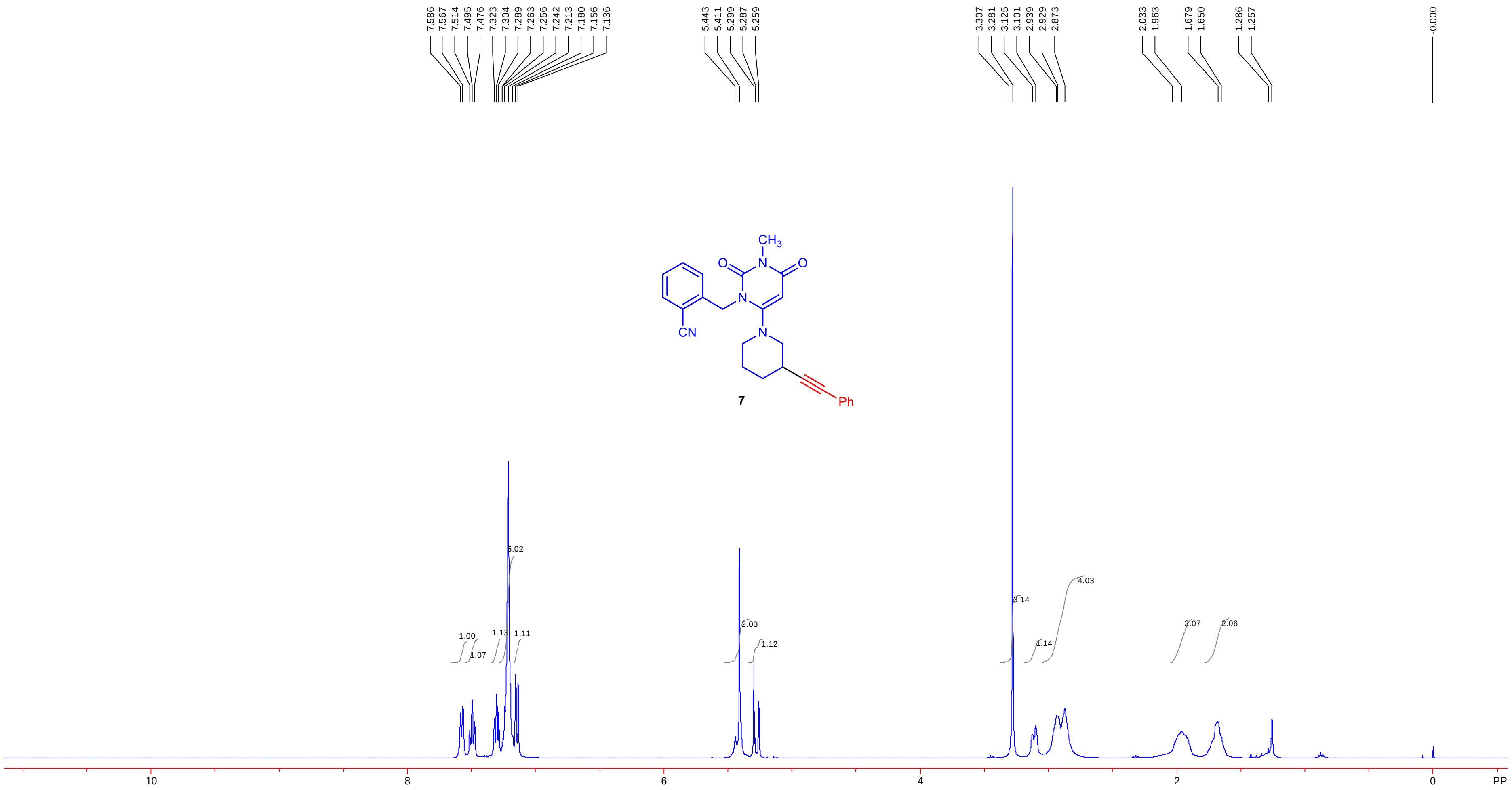

Supplementary Figure 150. ^{13}C NMR(100 MHz, $CDCl_3$)

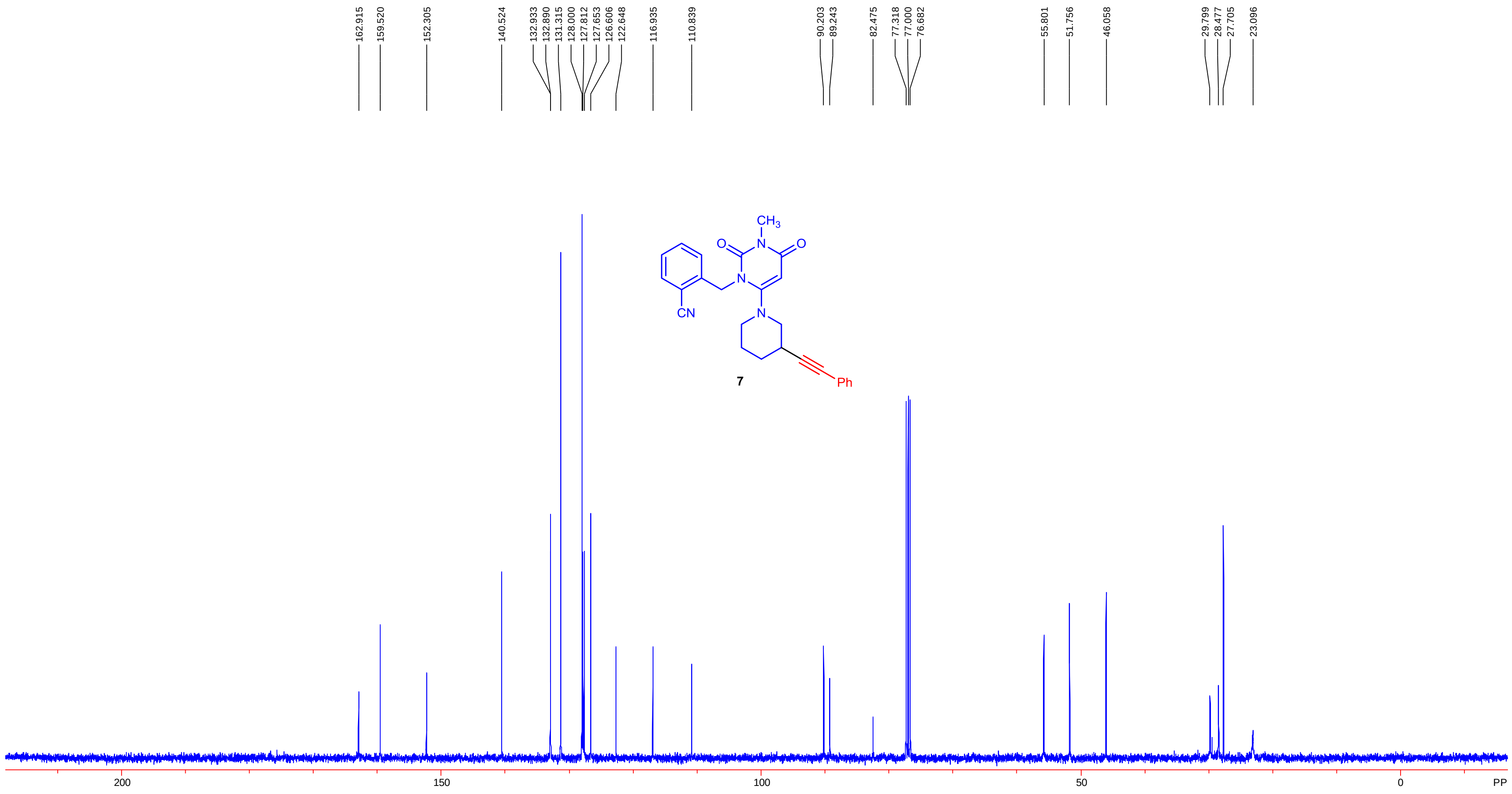

Supplementary Figure 151. ^{1}H NMR(400 MHz, $CDCl_3$)

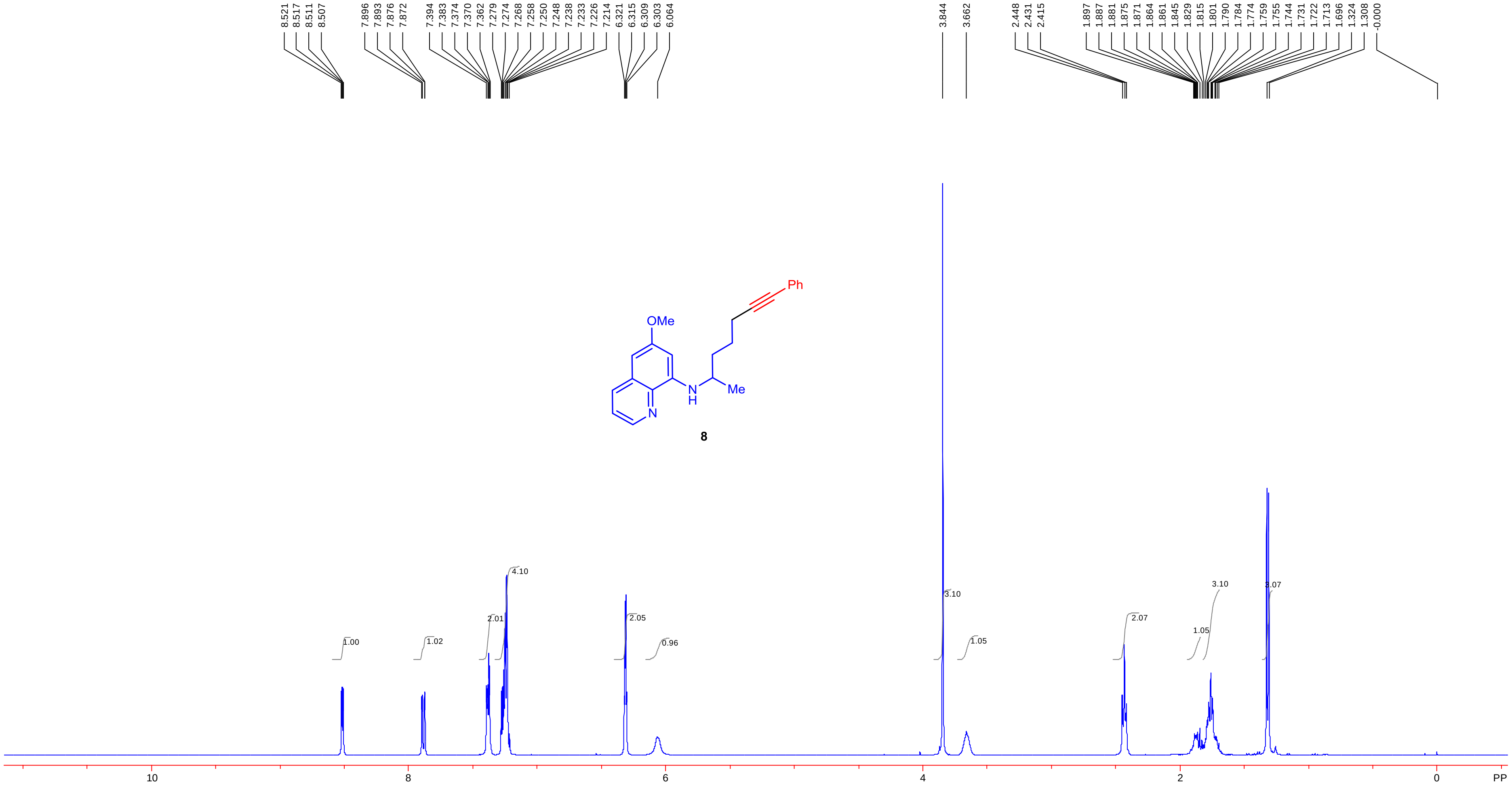

Supplementary Figure 152. ^{13}C NMR(100 MHz, $CDCl_3$)

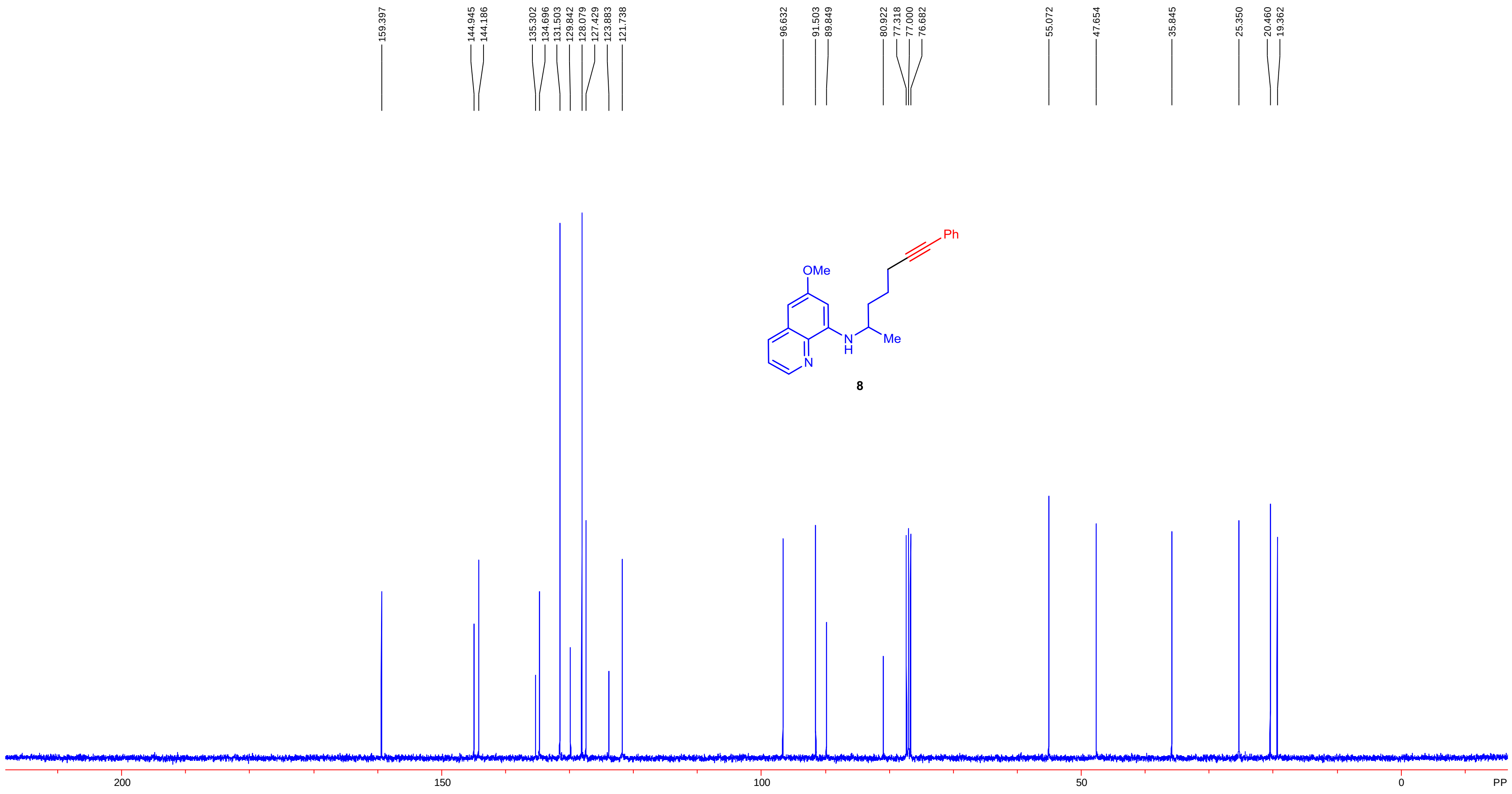

Supplementary Figure 153. ^{1}H NMR (600 MHz, $CDCl_3$)

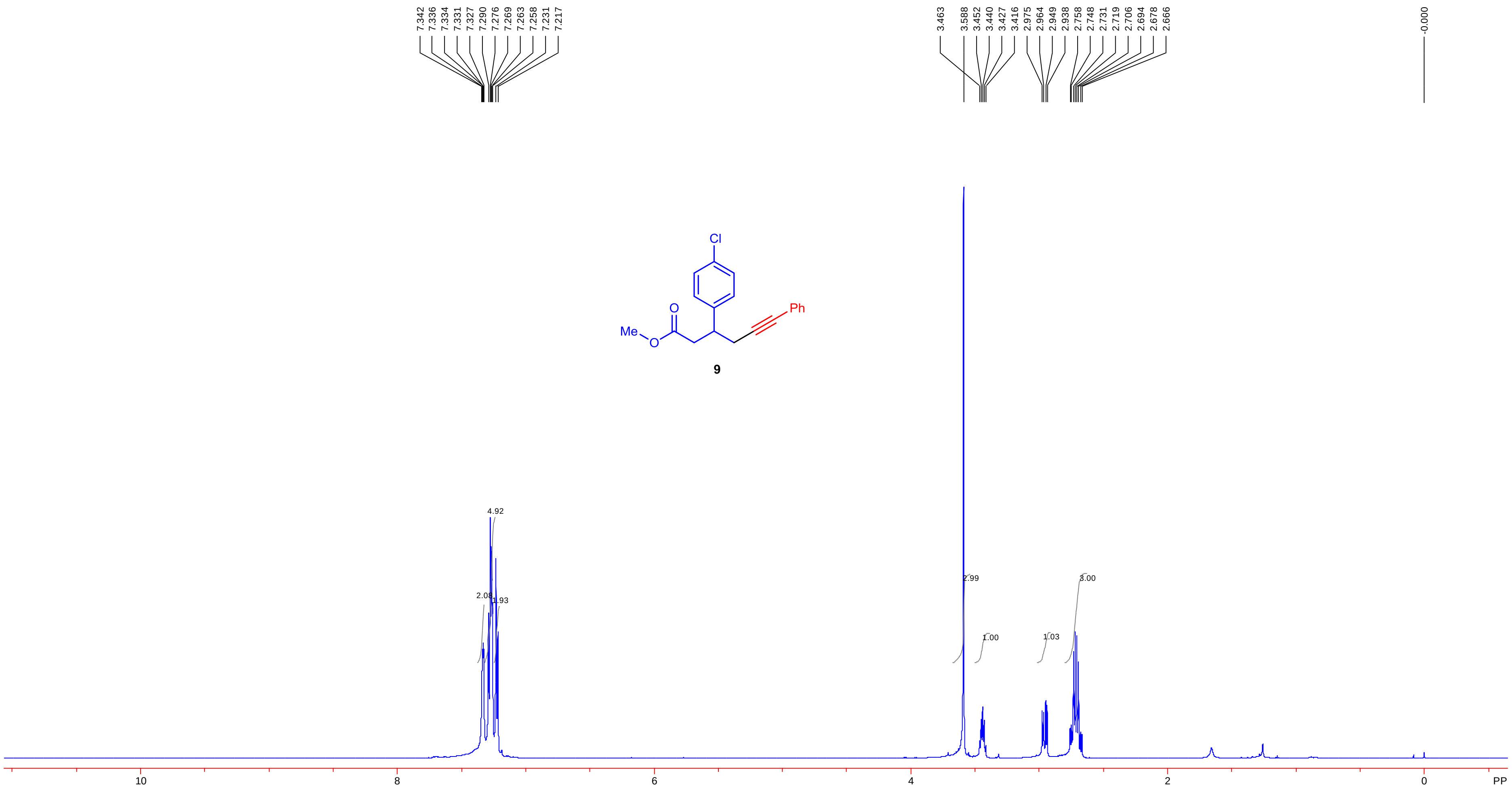

Supplementary Figure 154. ^{13}C NMR (151 MHz, $CDCl_3$)

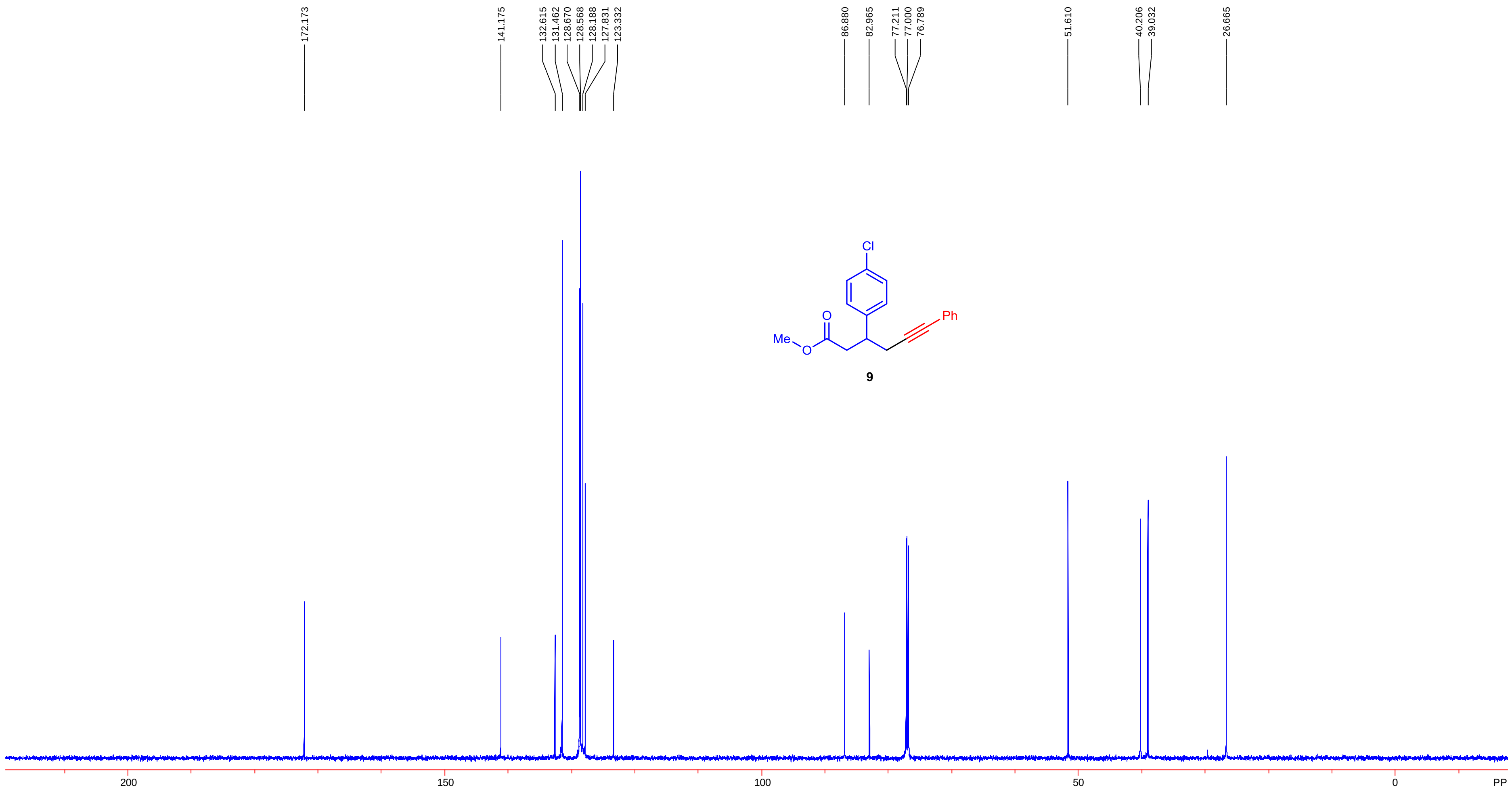

Supplementary Figure 155. ^{1}H NMR (600 MHz, $CDCl_3$)

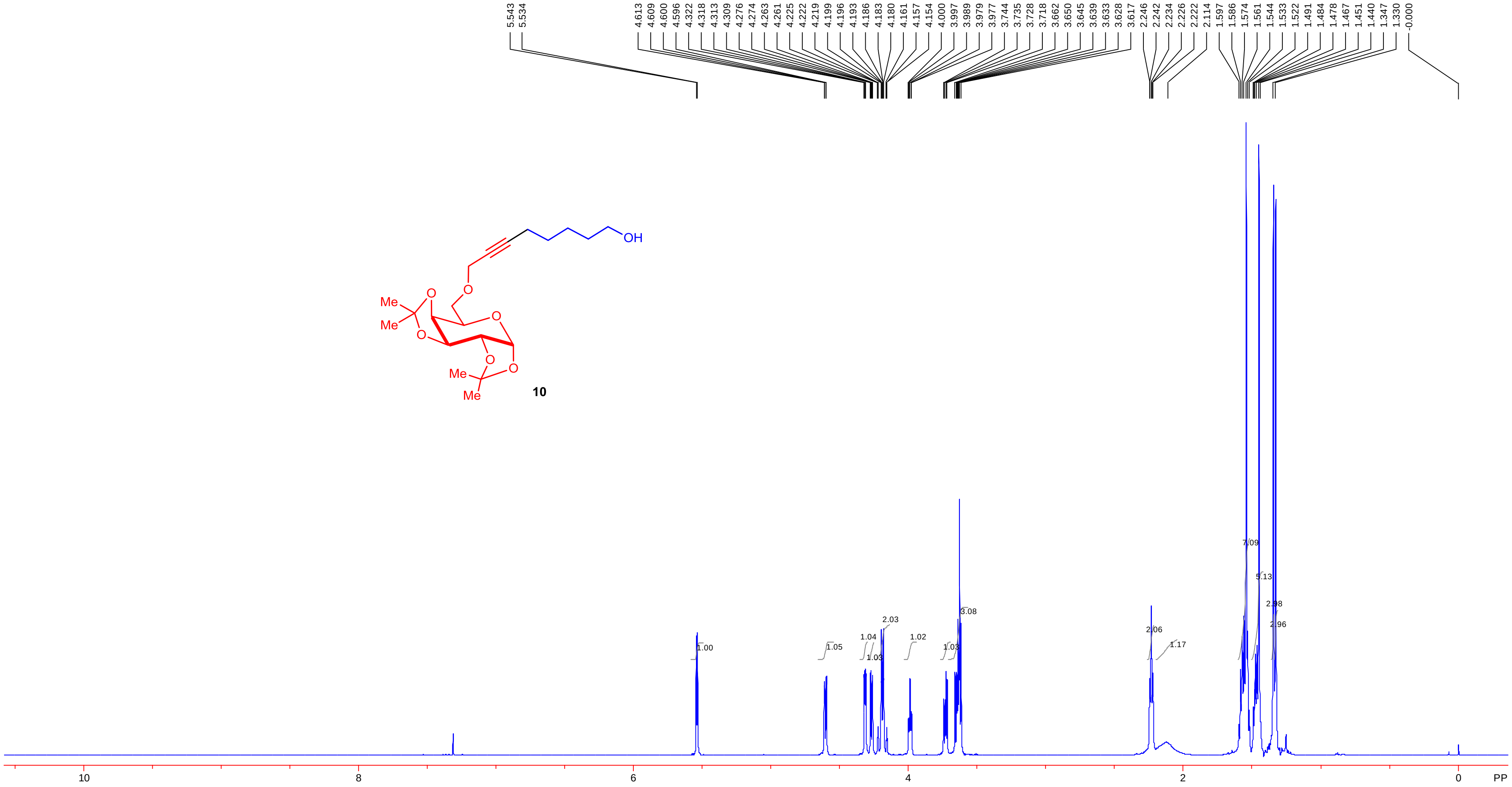

Supplementary Figure 156. ^{13}C NMR (151 MHz, $CDCl_3$)

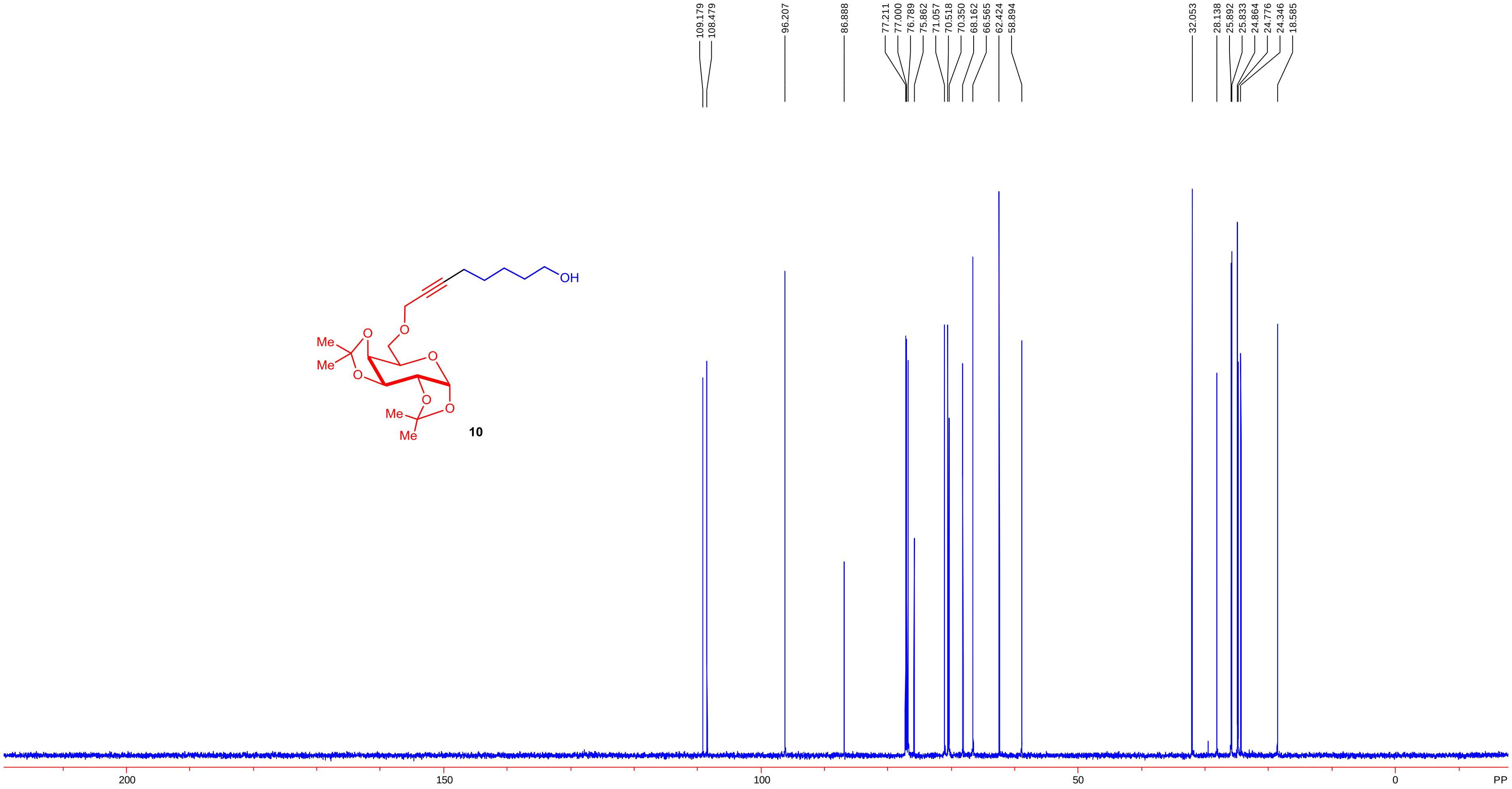

Supplementary Figure 157. ^{1}H NMR(400 MHz, $CDCl_3$)

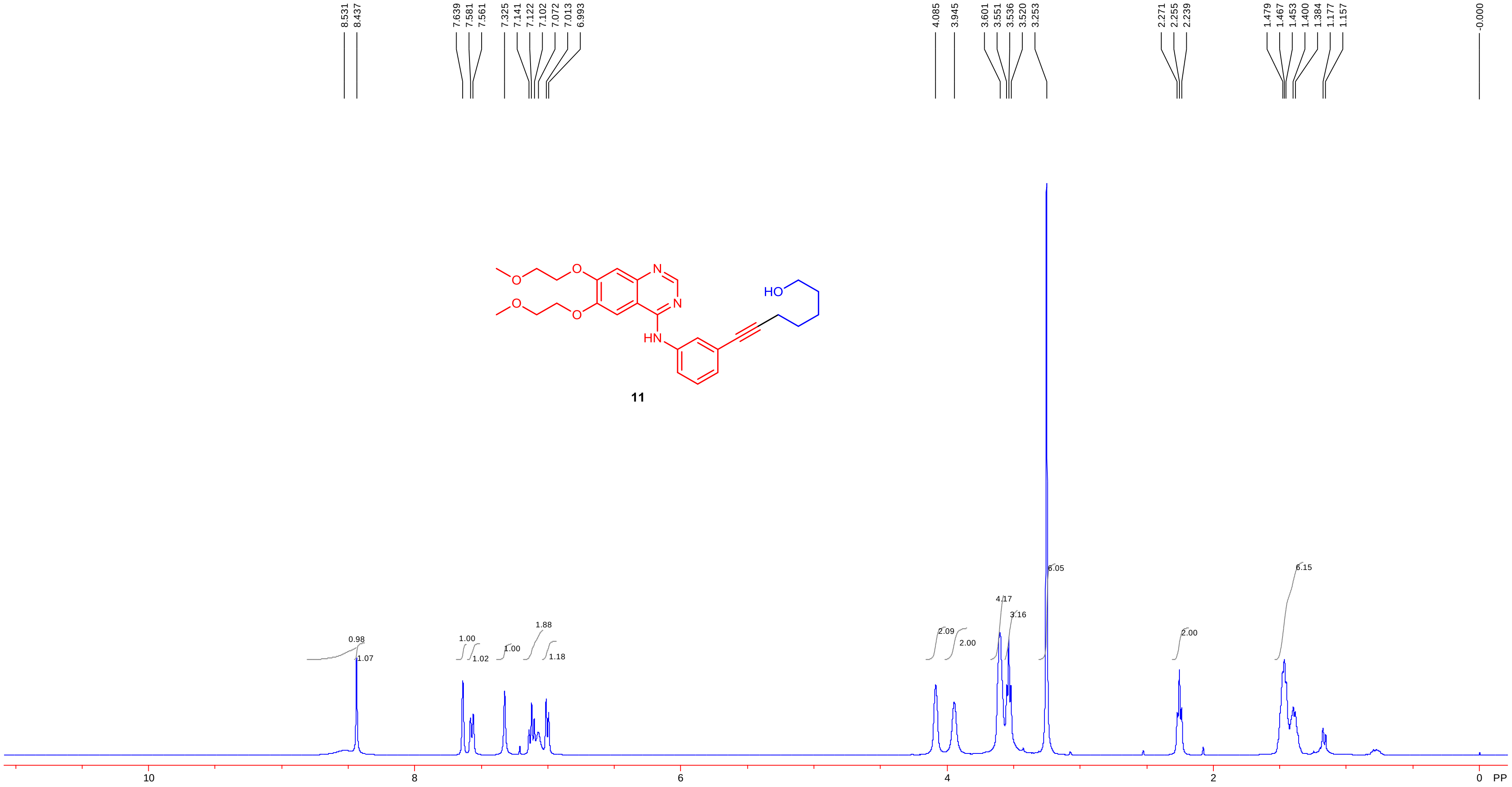

Supplementary Figure 158. ^{13}C NMR(100 MHz, $CDCl_3$)

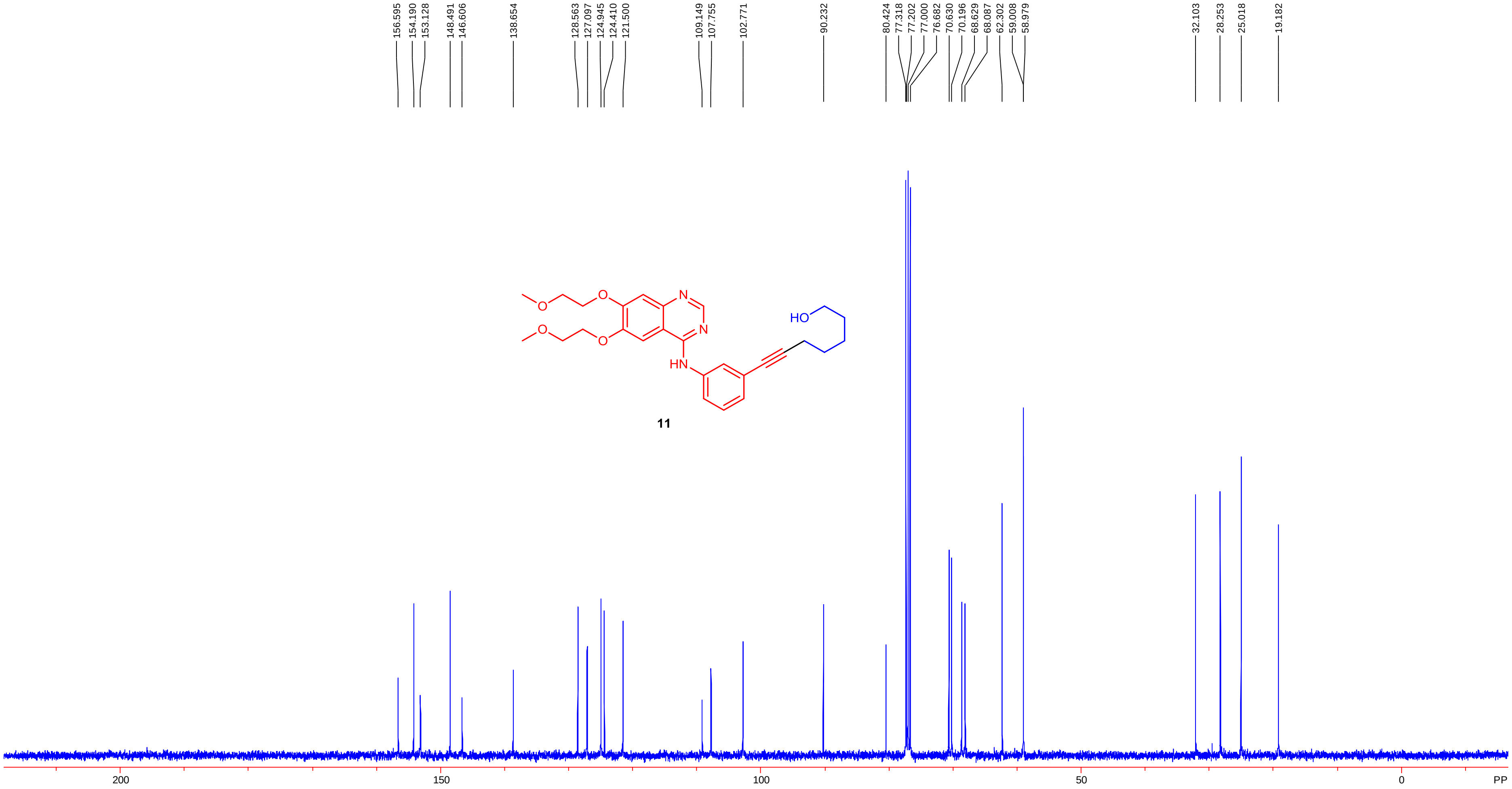

Supplementary Figure 159. ^{1}H NMR(400 MHz, $CDCl_3$)

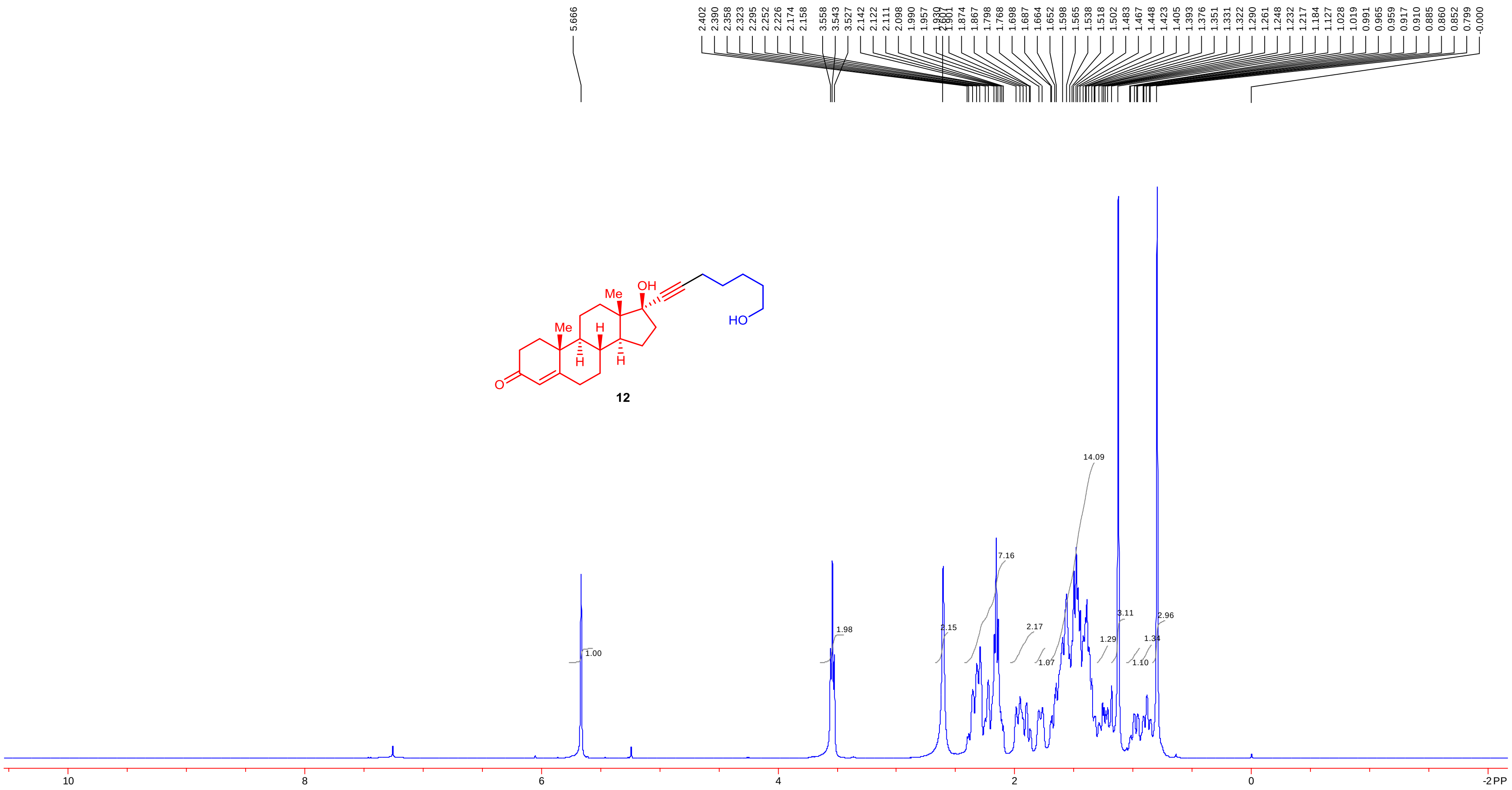

Supplementary Figure 160. ^{13}C NMR(100 MHz, $CDCl_3$)

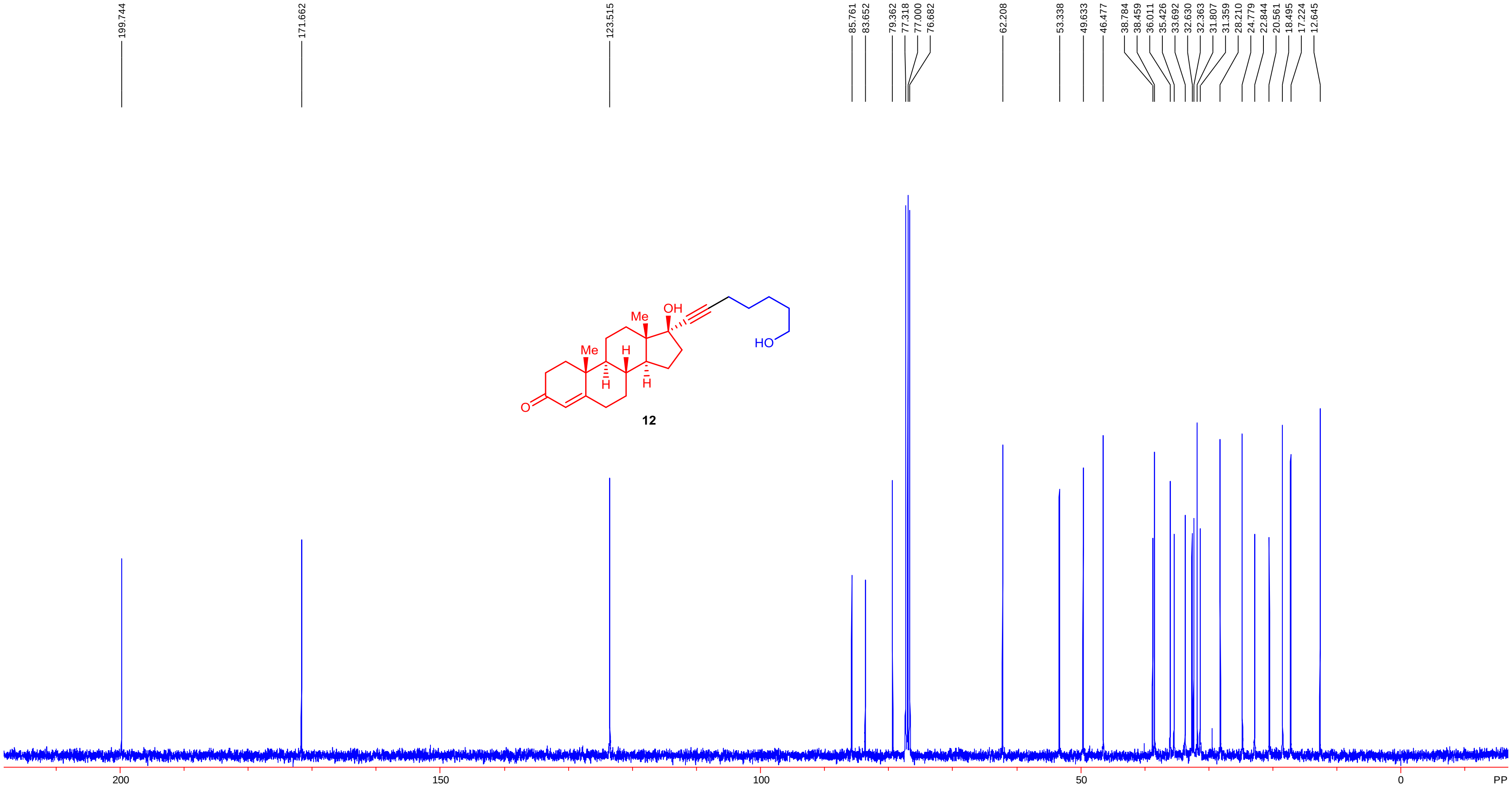

Supplementary Figure 161. ^{1}H NMR(400 MHz, $CDCl_3$)

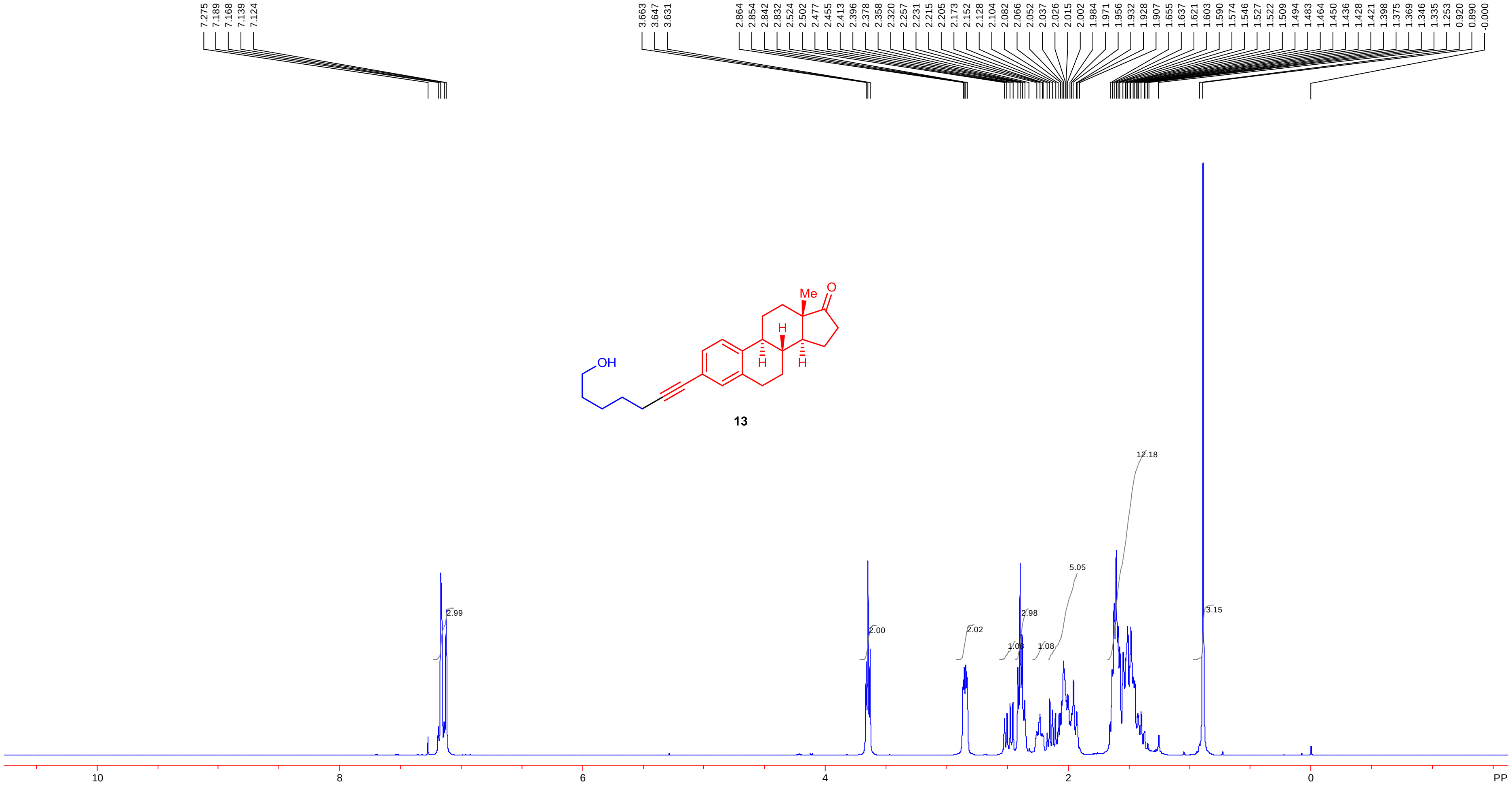

Supplementary Figure 162. ^{13}C NMR(100 MHz, $CDCl_3$)

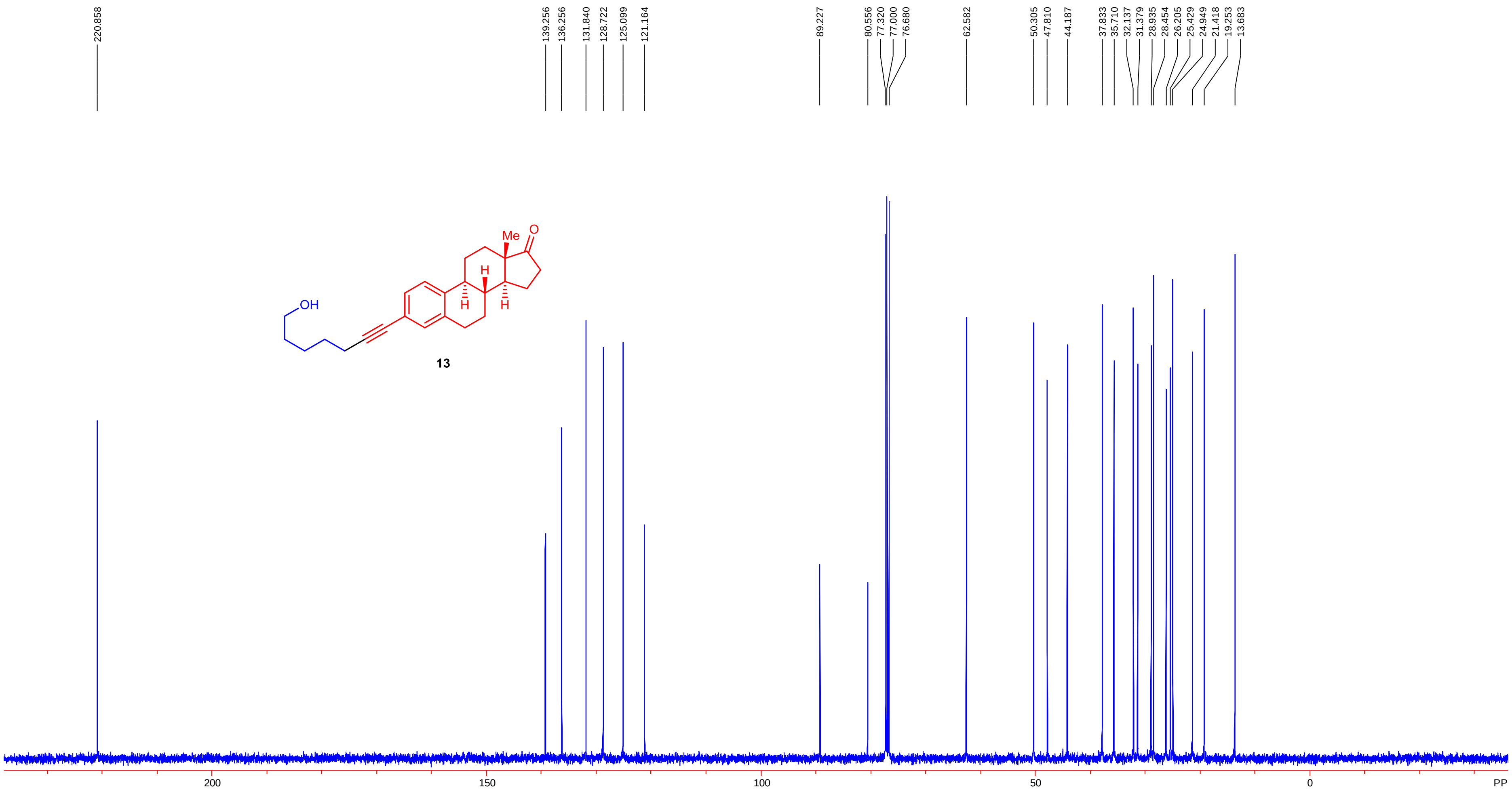

Supplementary Figure 163. ^{1}H NMR (600 MHz, $CDCl_3$)

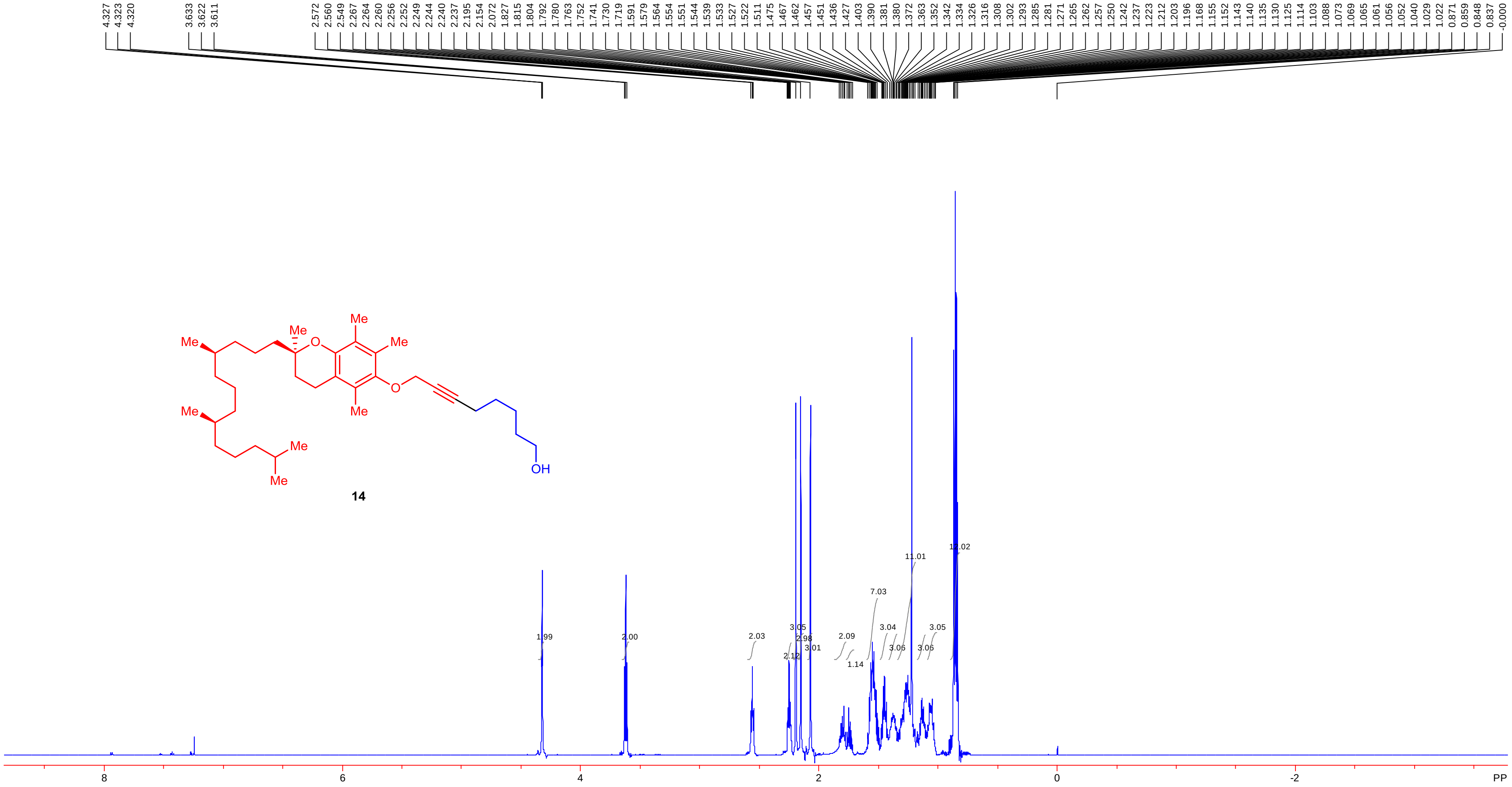

Supplementary Figure 164. ^{13}C NMR (151 MHz, $CDCl_3$)

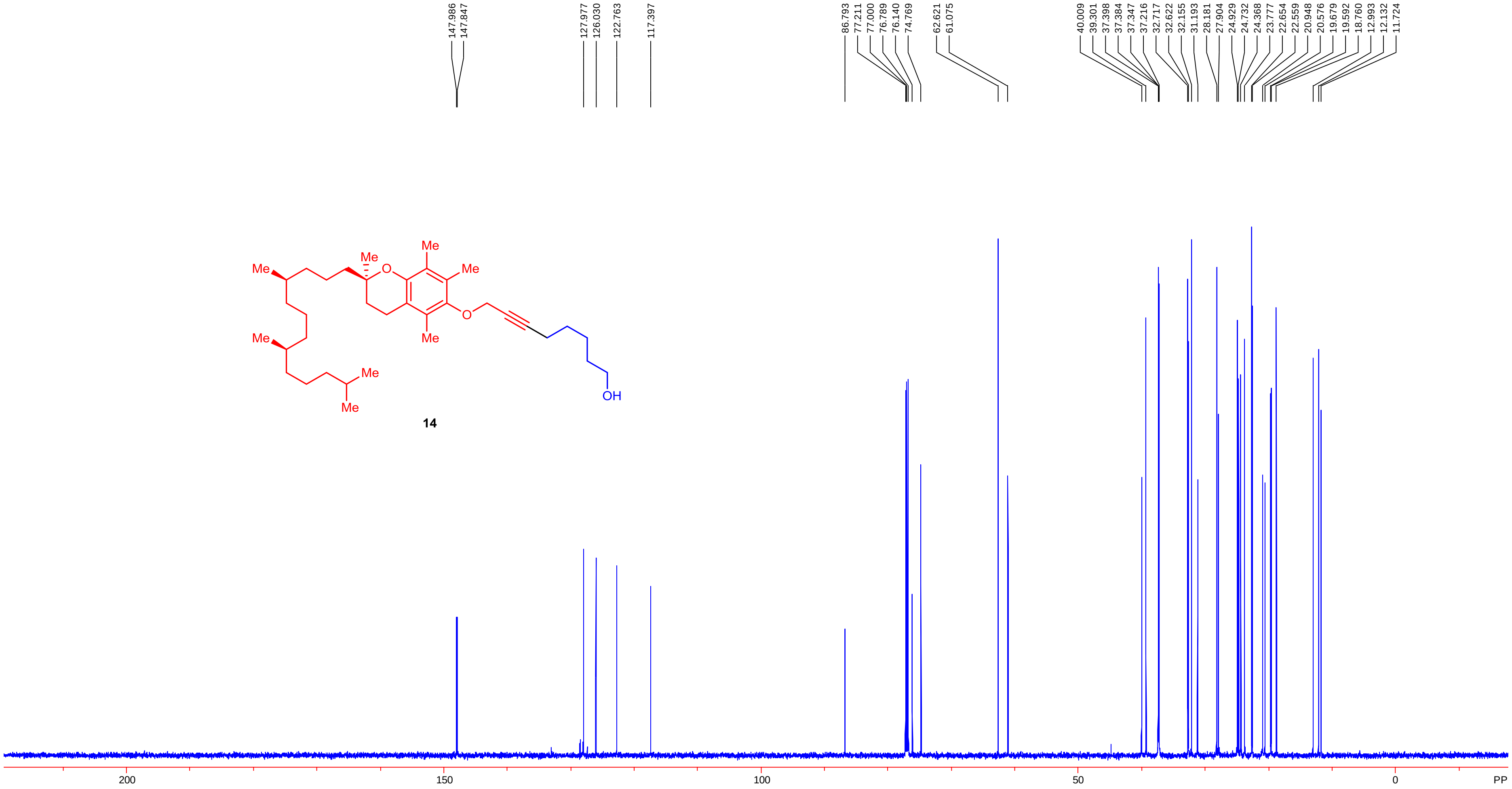

Supplementary Figure 165. ^{1}H NMR(400 MHz, $CDCl_3$)

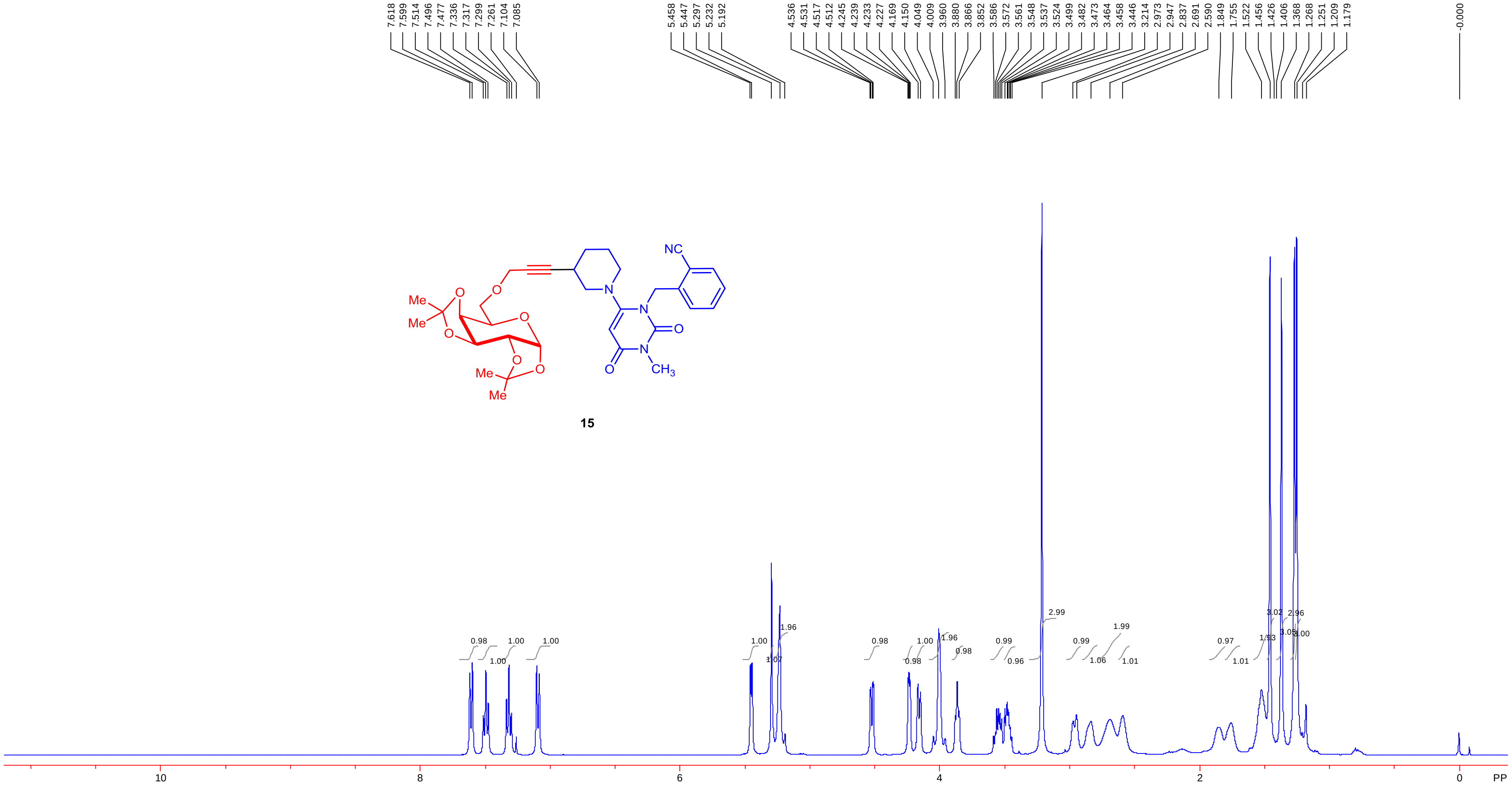

Supplementary Figure 166. ^{13}C NMR(100 MHz, $CDCl_3$)

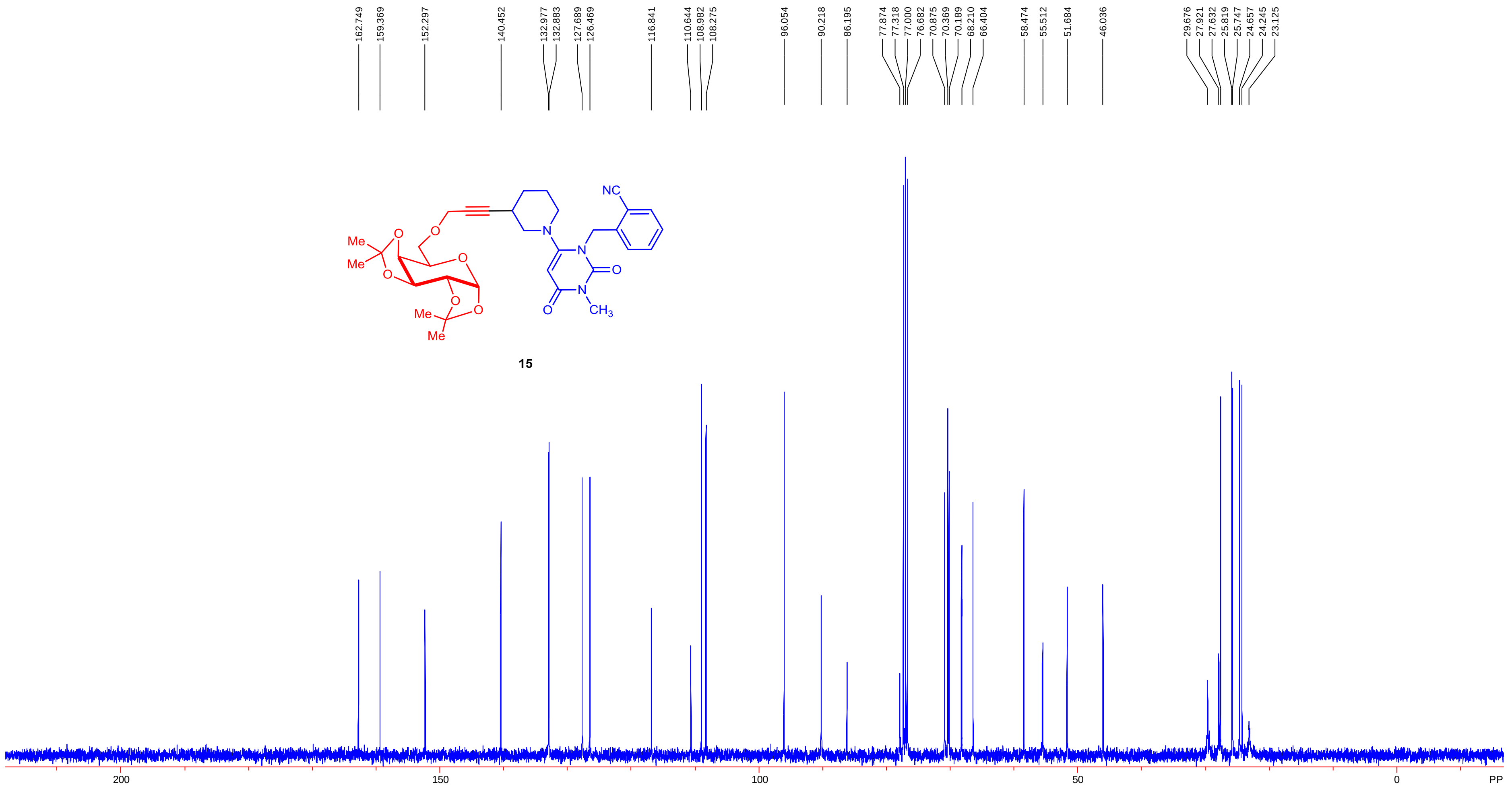

Supplementary Figure 167. ^{1}H NMR(400 MHz, $CDCl_3$)

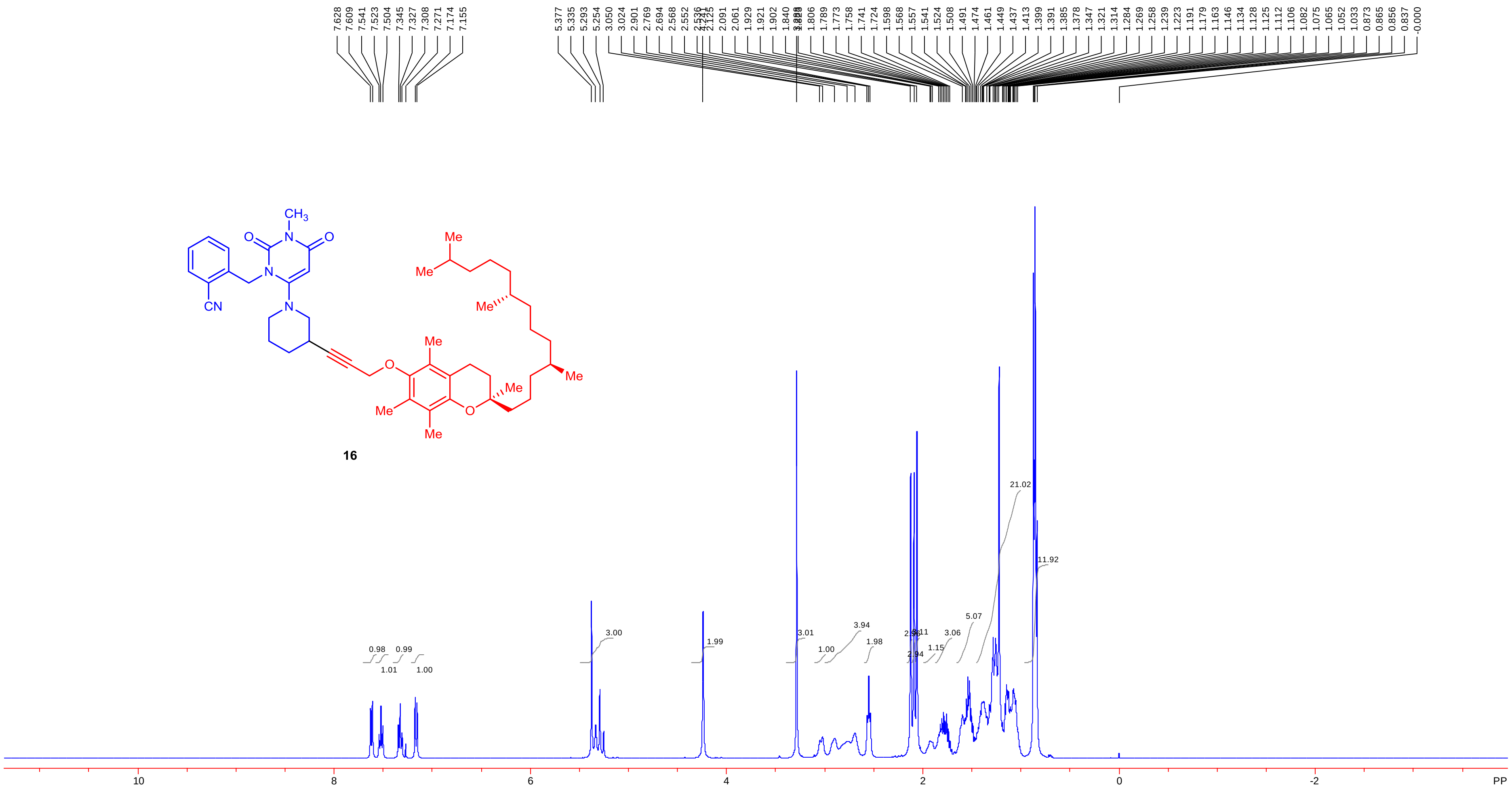

Supplementary Figure 168. ^{13}C NMR(100 MHz, $CDCl_3$)

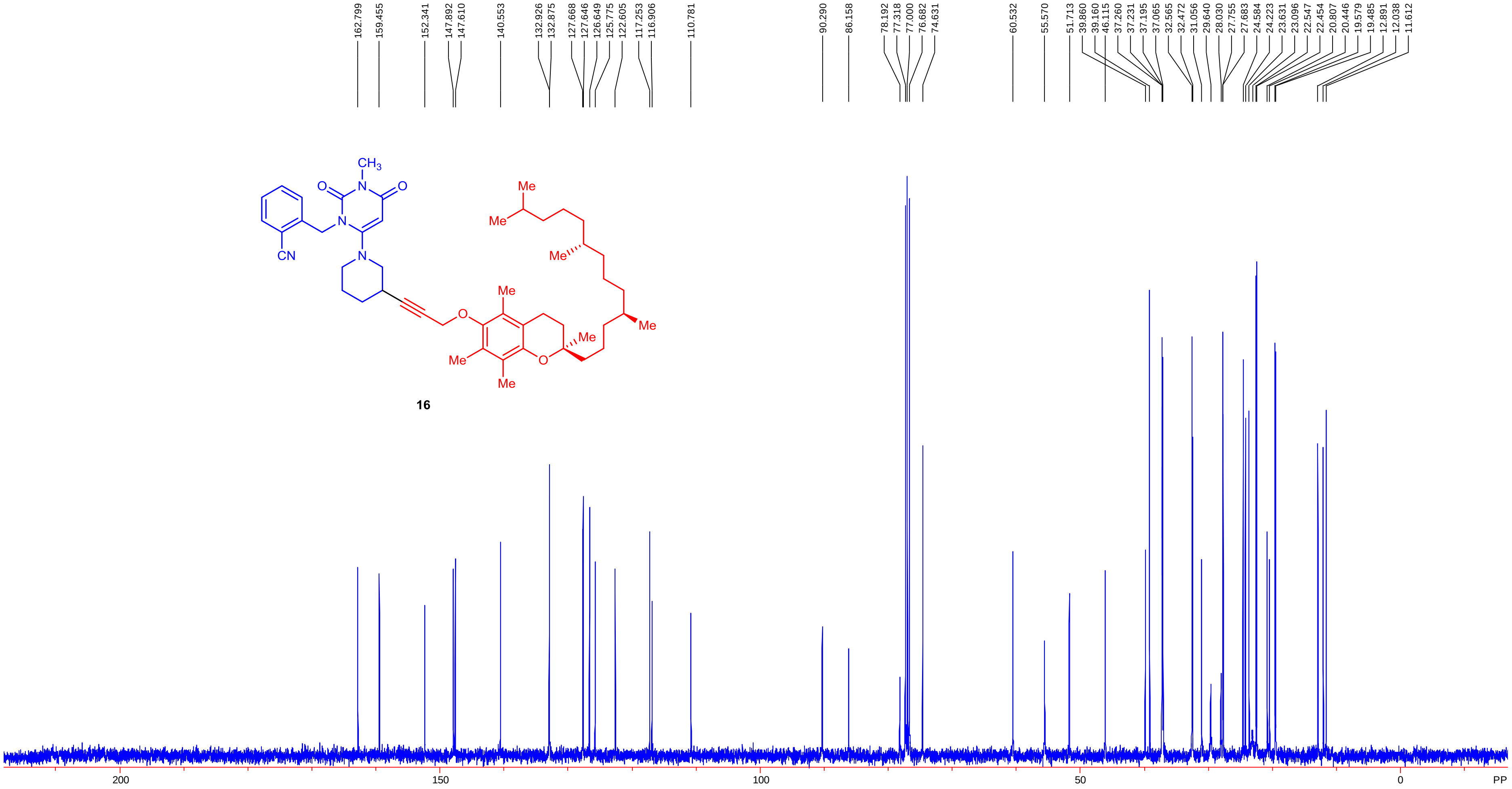

Supplementary Figure 169. ^{1}H NMR(400 MHz, $CDCl_3$)

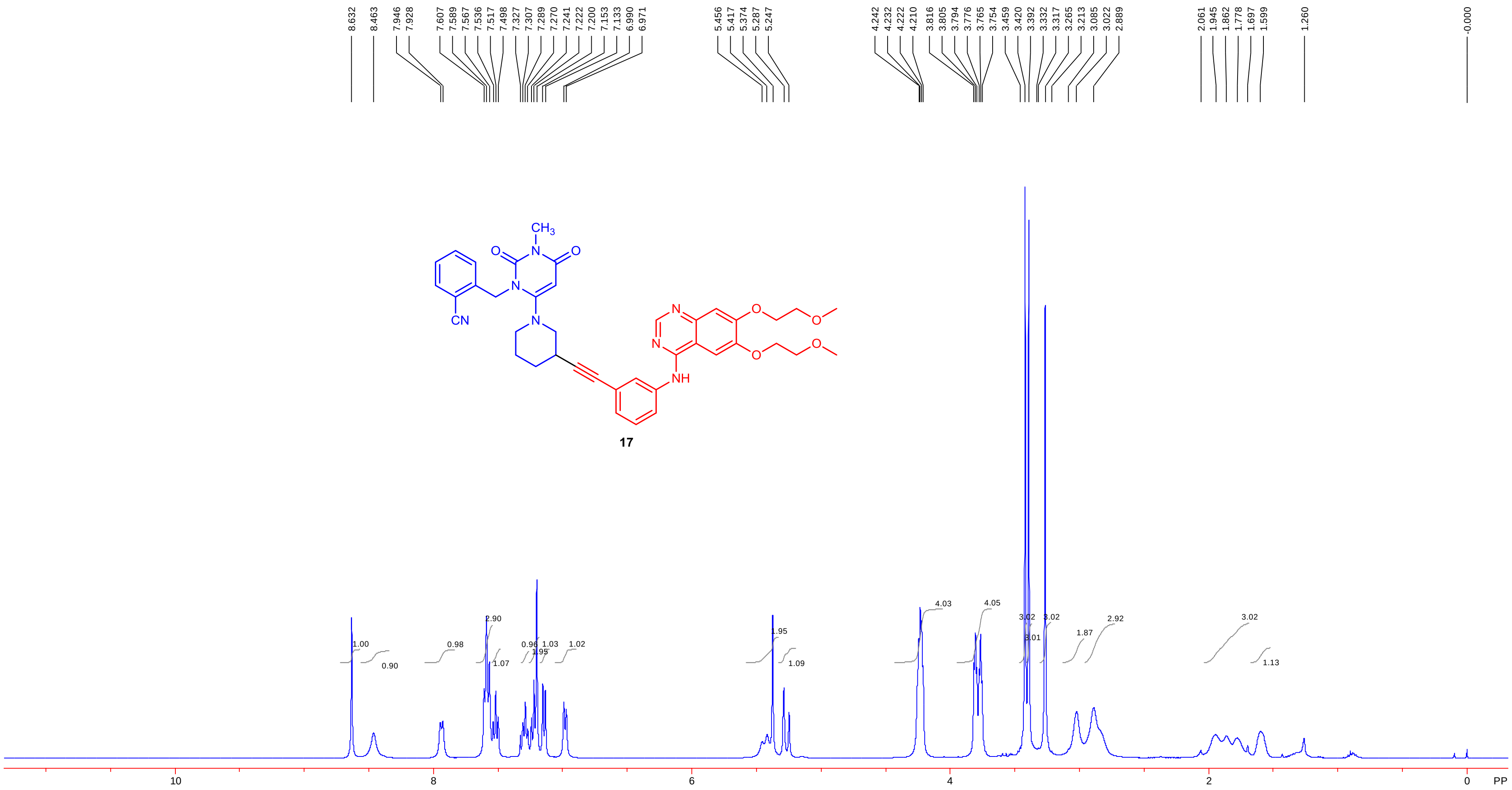

Supplementary Figure 170. ^{13}C NMR(100 MHz, $CDCl_3$)

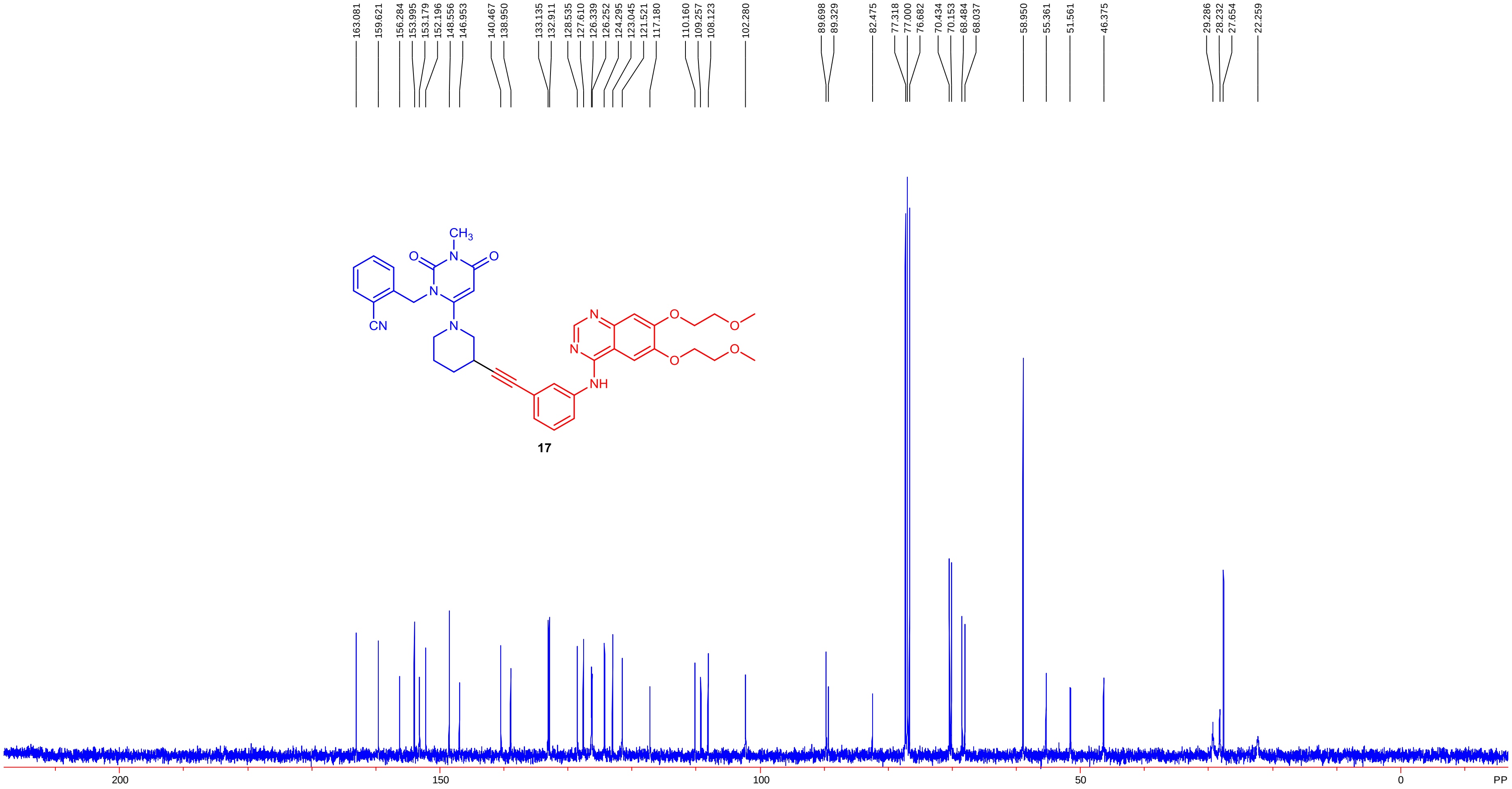

Supplementary Figure 171. ^{1}H NMR(400 MHz, DMSO-d_6)

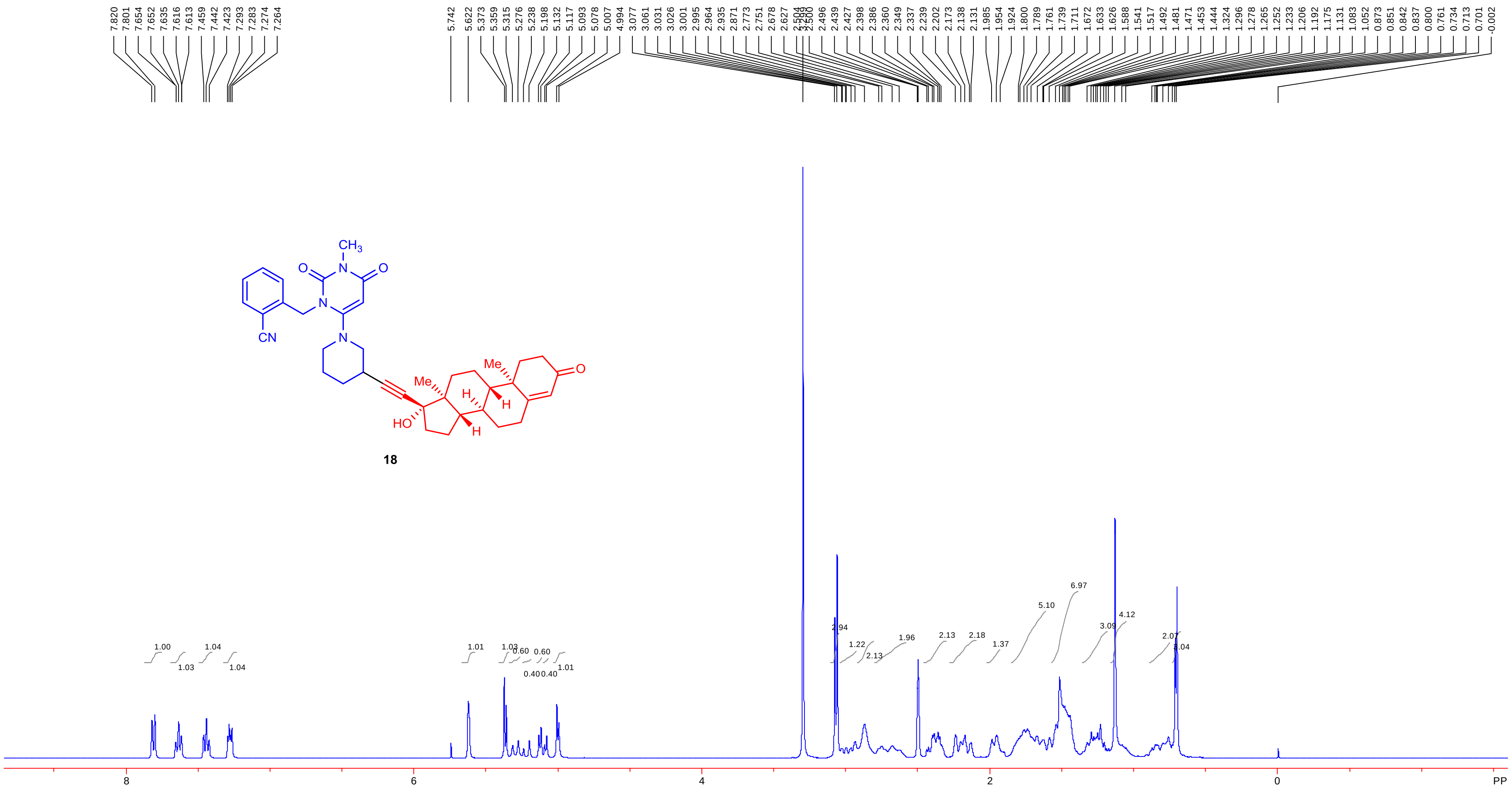

Supplementary Figure 172. ^{13}C NMR (151 MHz, $CDCl_3$)

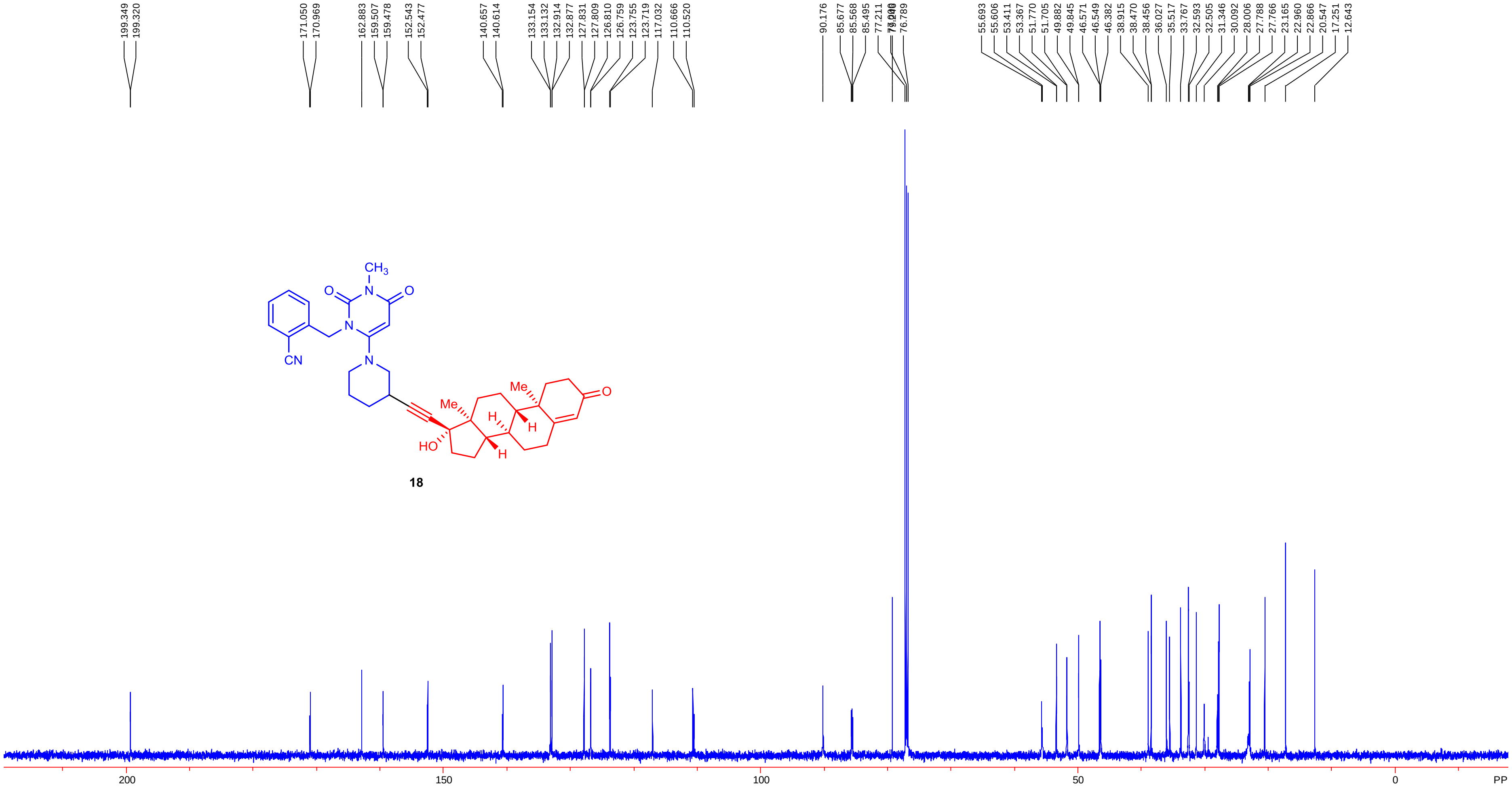

Supplementary Figure 173. ^{1}H NMR(600 MHz, $CDCl_3$)

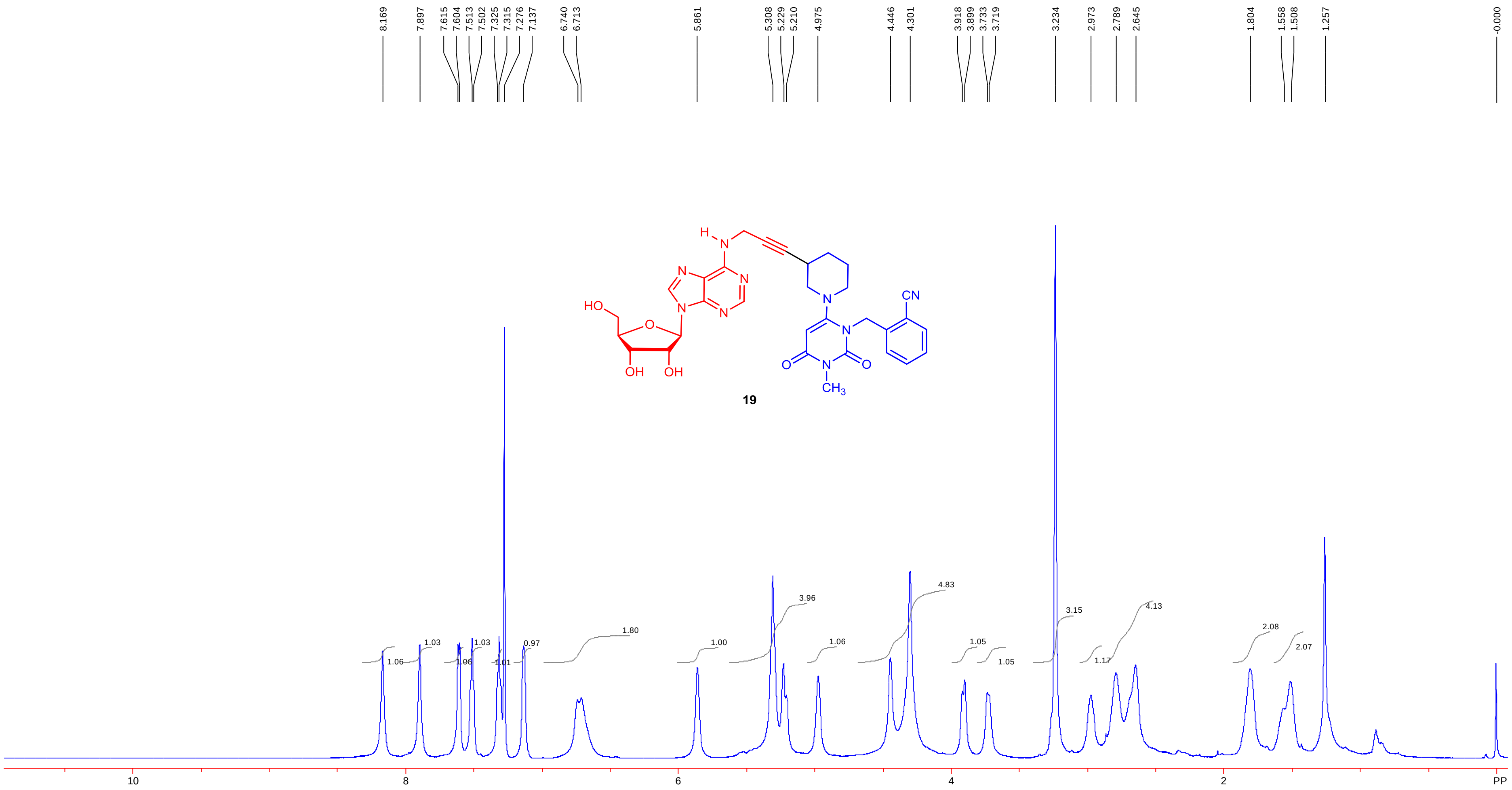

Supplementary Figure 174. ^{13}C NMR (151 MHz, $CDCl_3$)

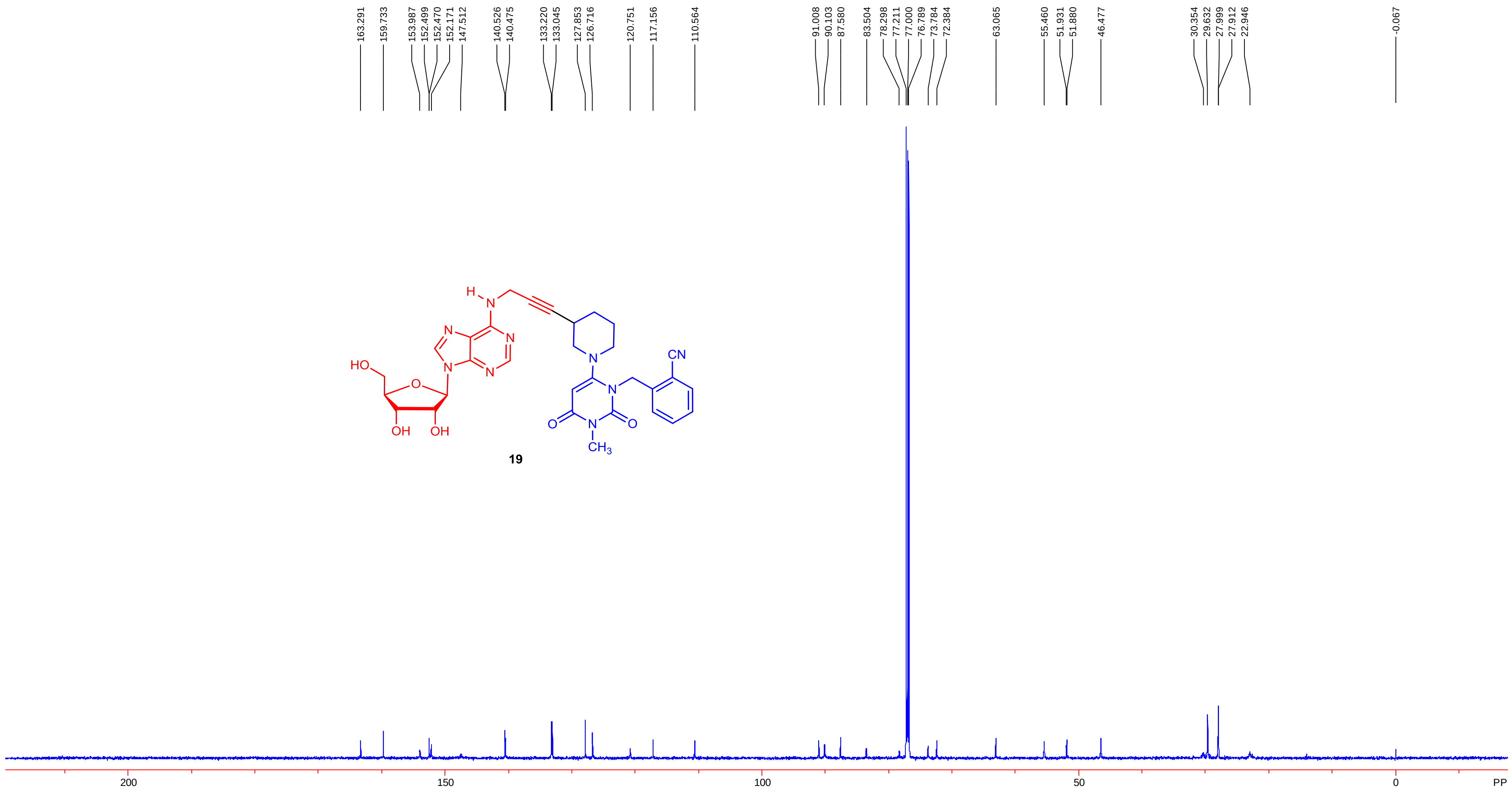

Supplementary Figure 175. ^1H NMR (400 MHz, $CDCl_3$)

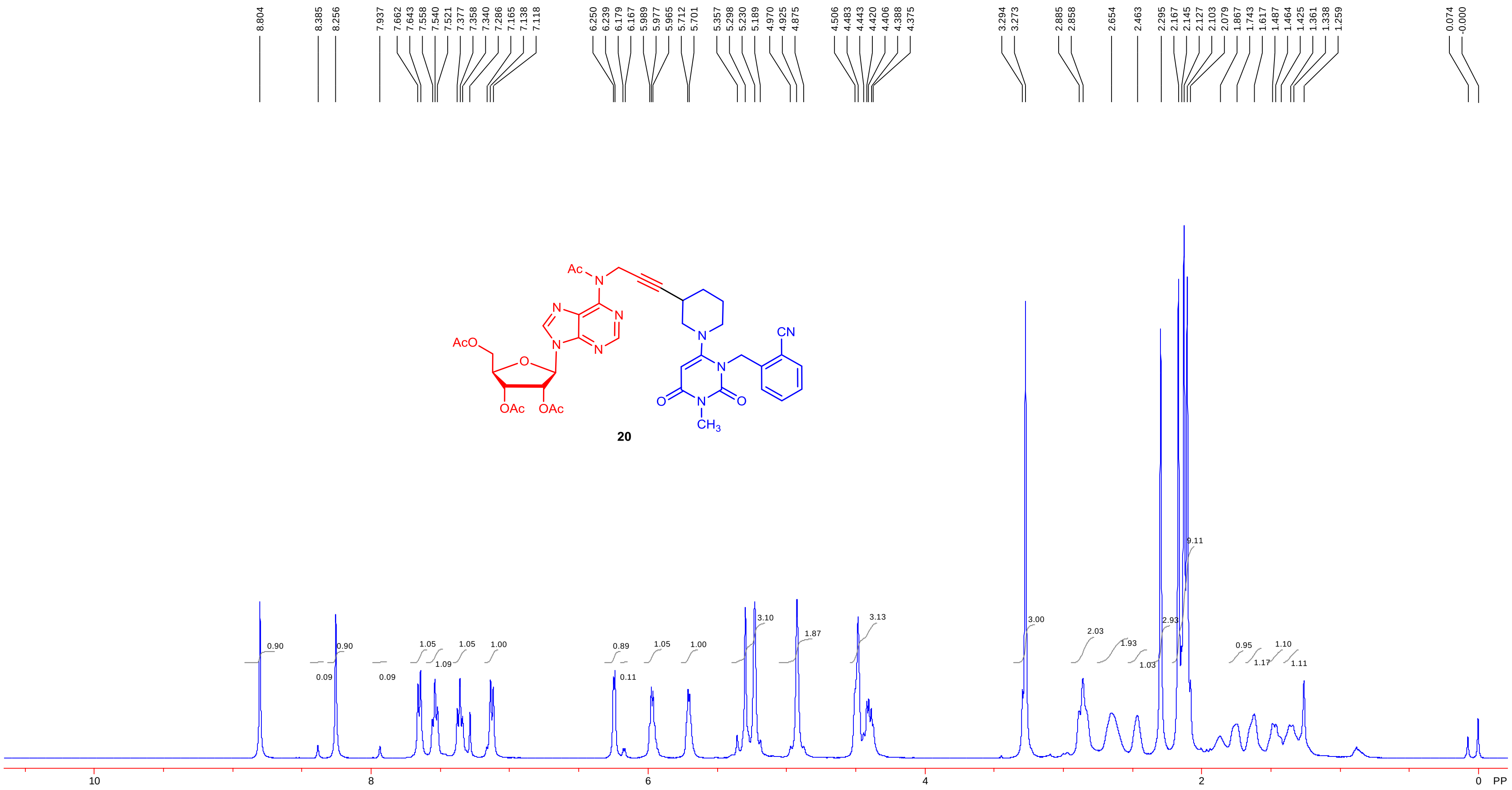

Supplementary Figure 176. ^{13}C NMR (151 MHz, $CDCl_3$)

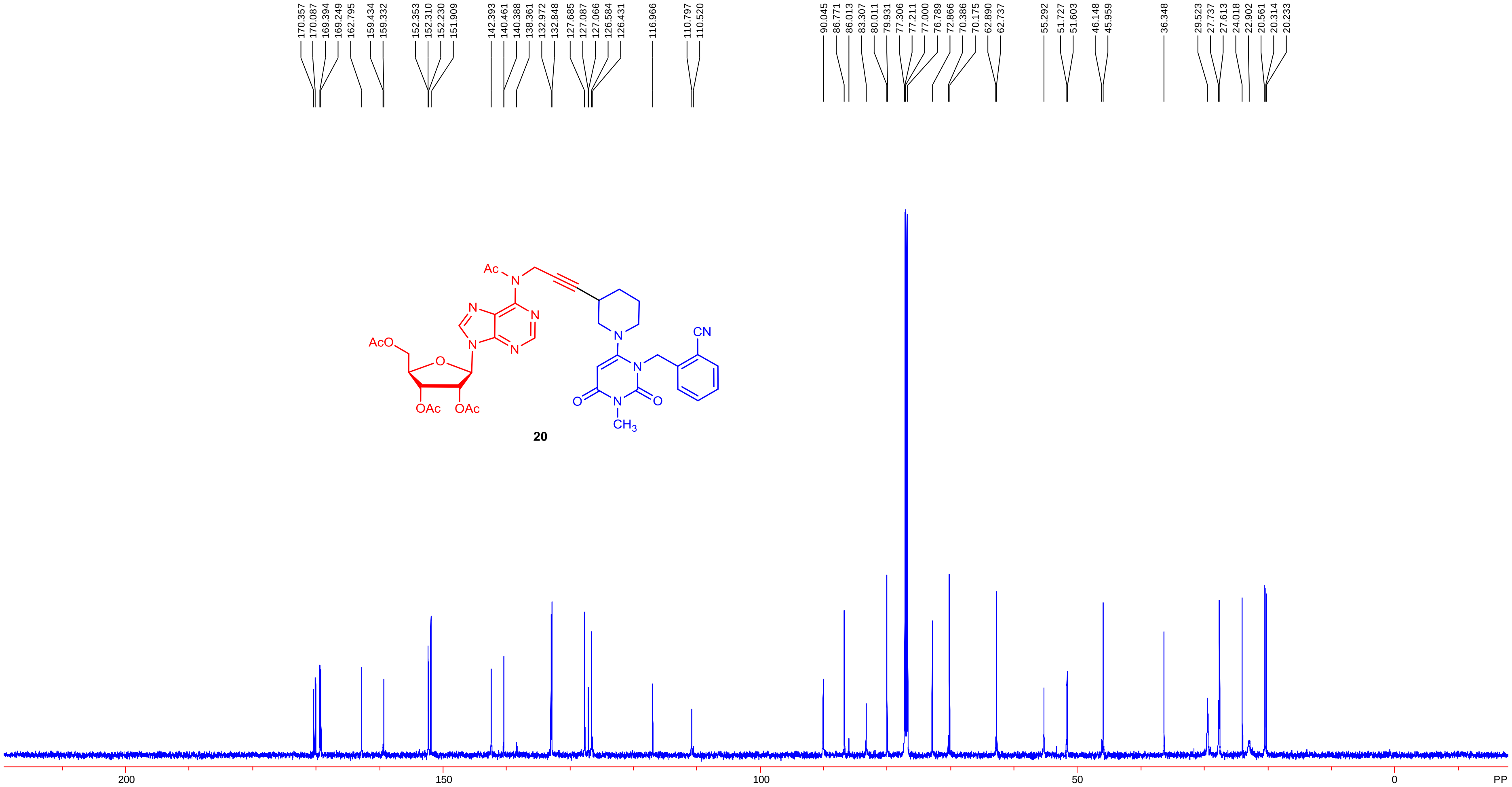

Supplementary Figure 177. ^{1}H NMR(600 MHz, $CDCl_3$)

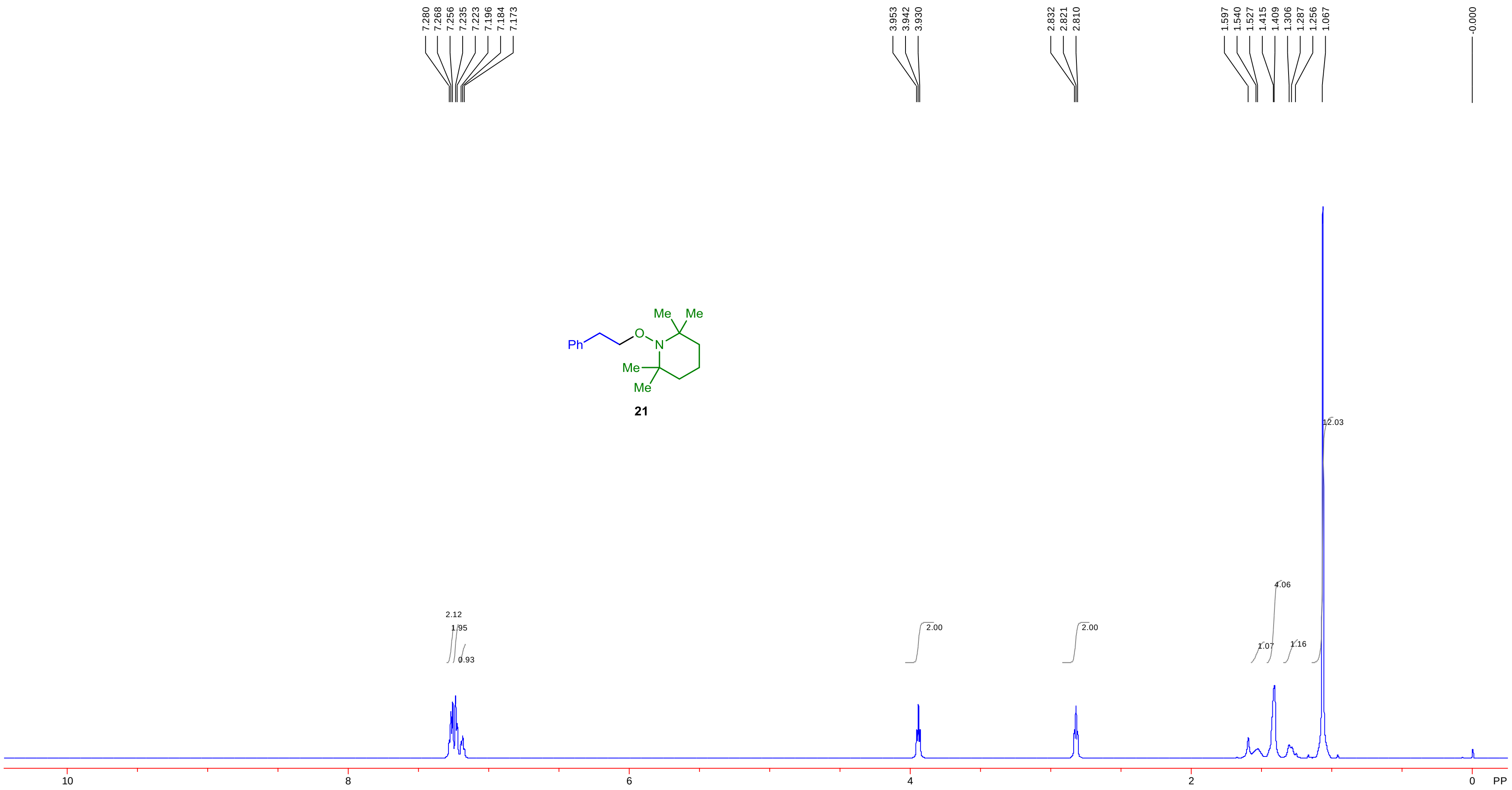

Supplementary Figure 178. ^{13}C NMR (151 MHz, $CDCl_3$)

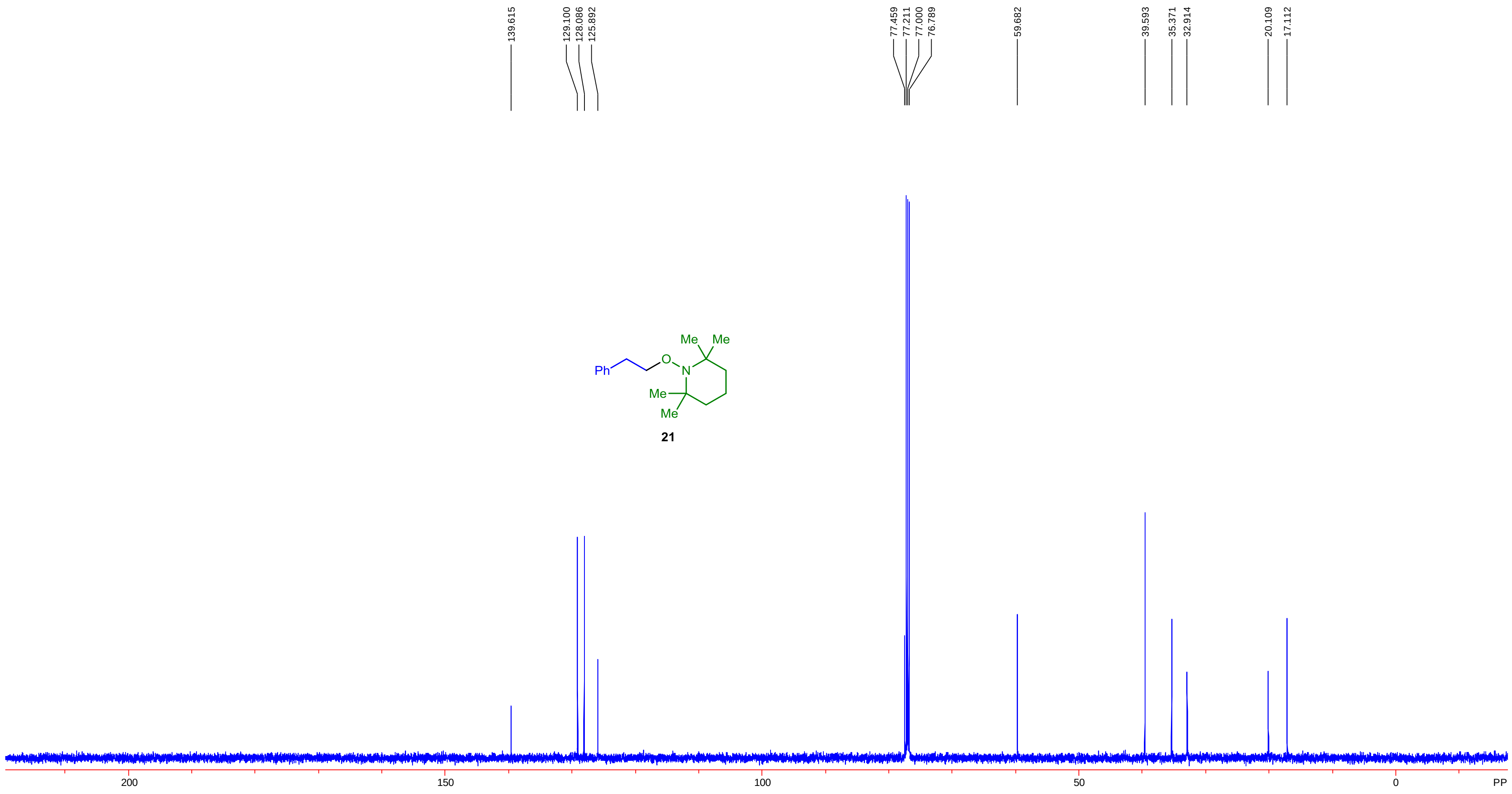

Supplementary Figure 179. ^{1}H NMR(400 MHz, $CDCl_3$)

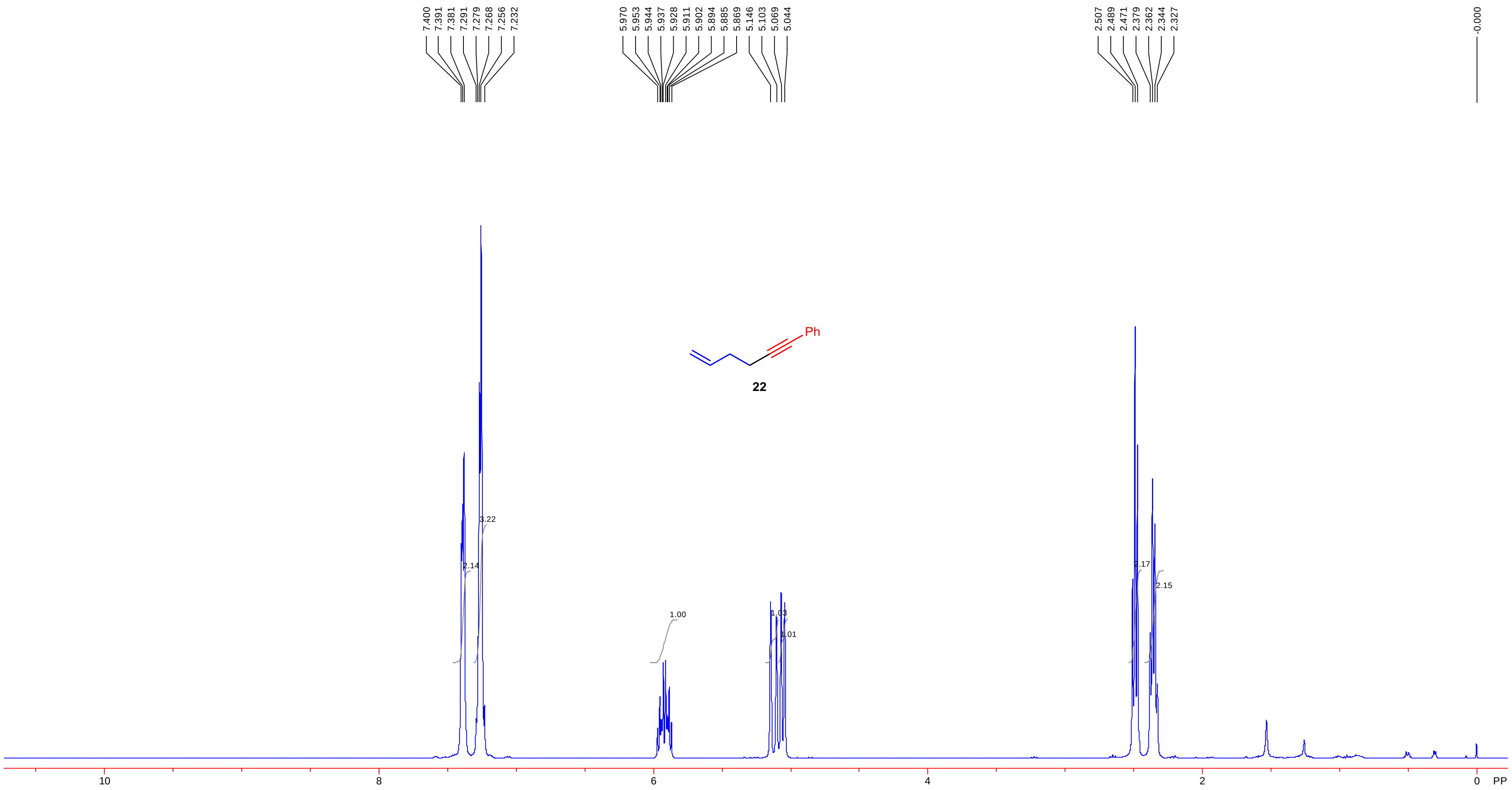

Supplementary Figure 180. ^{13}C NMR (100 MHz, $CDCl_3$)

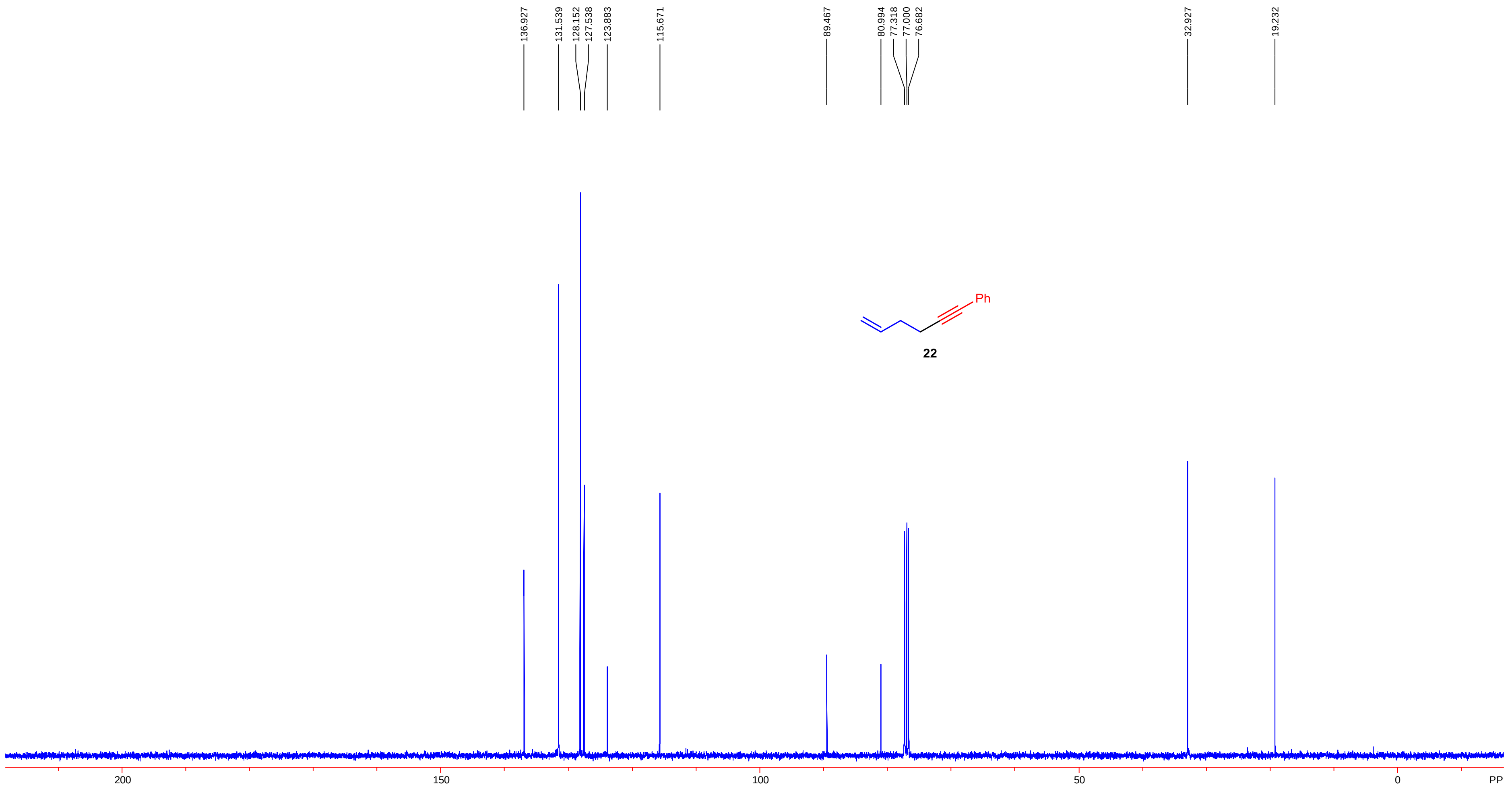

Supplementary Figure 181. ^{1}H NMR(400 MHz, CD_3OD)

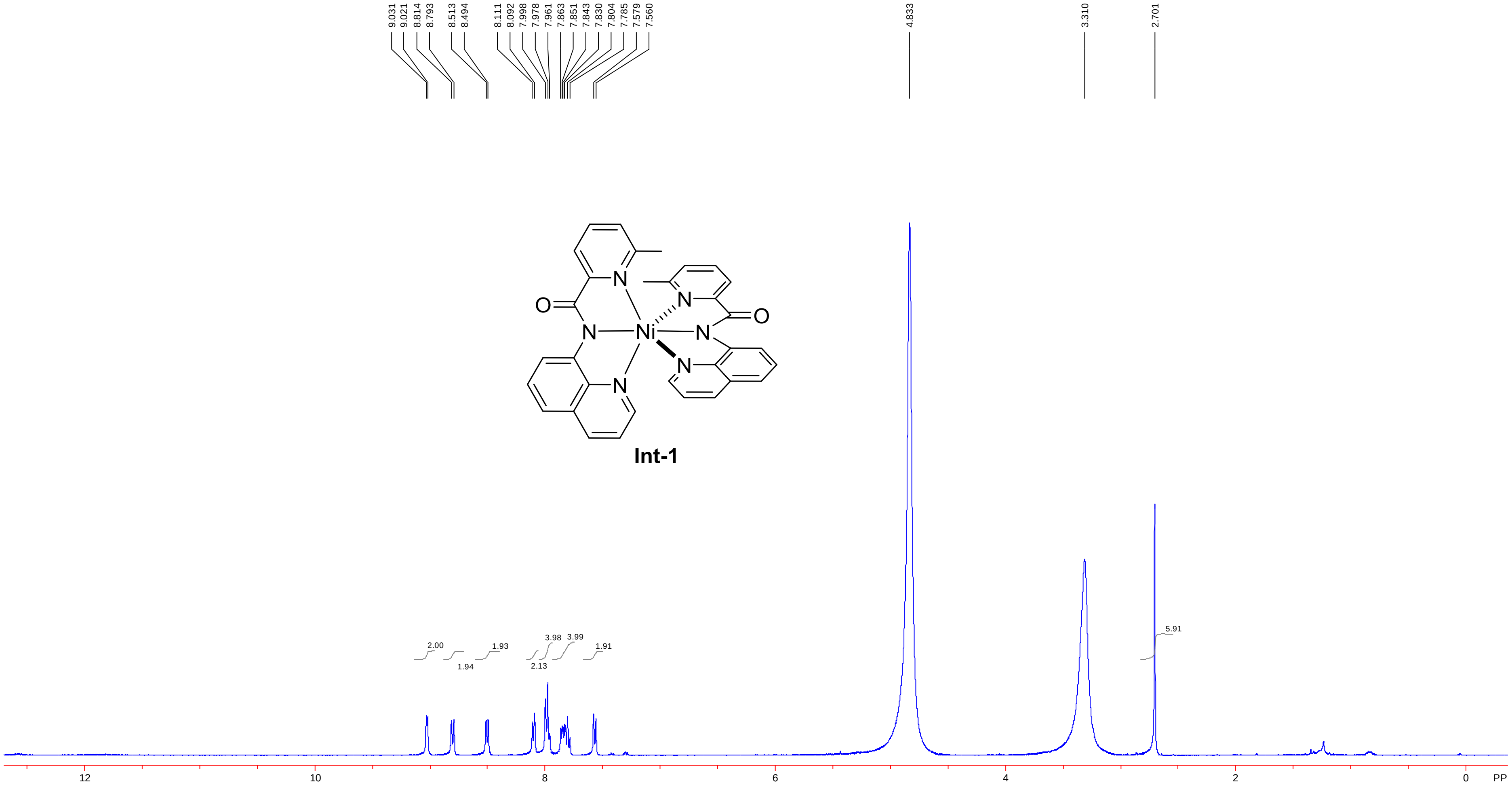

Supplementary Figure 182. ^{1}H NMR(600 MHz, C_6D_6)

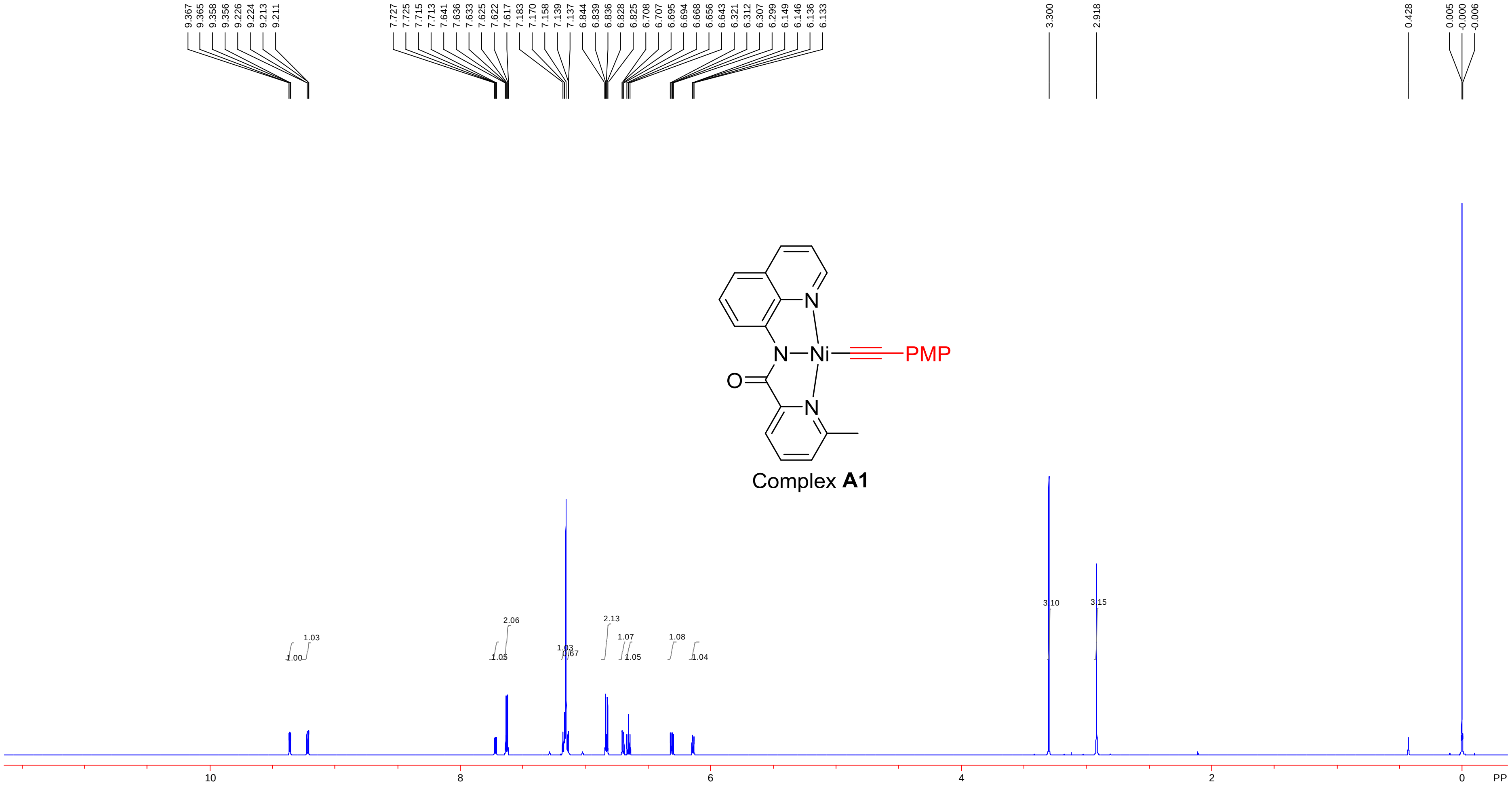

Supplementary Figure 183. ^{1}H NMR(400 MHz, C_6D_6)

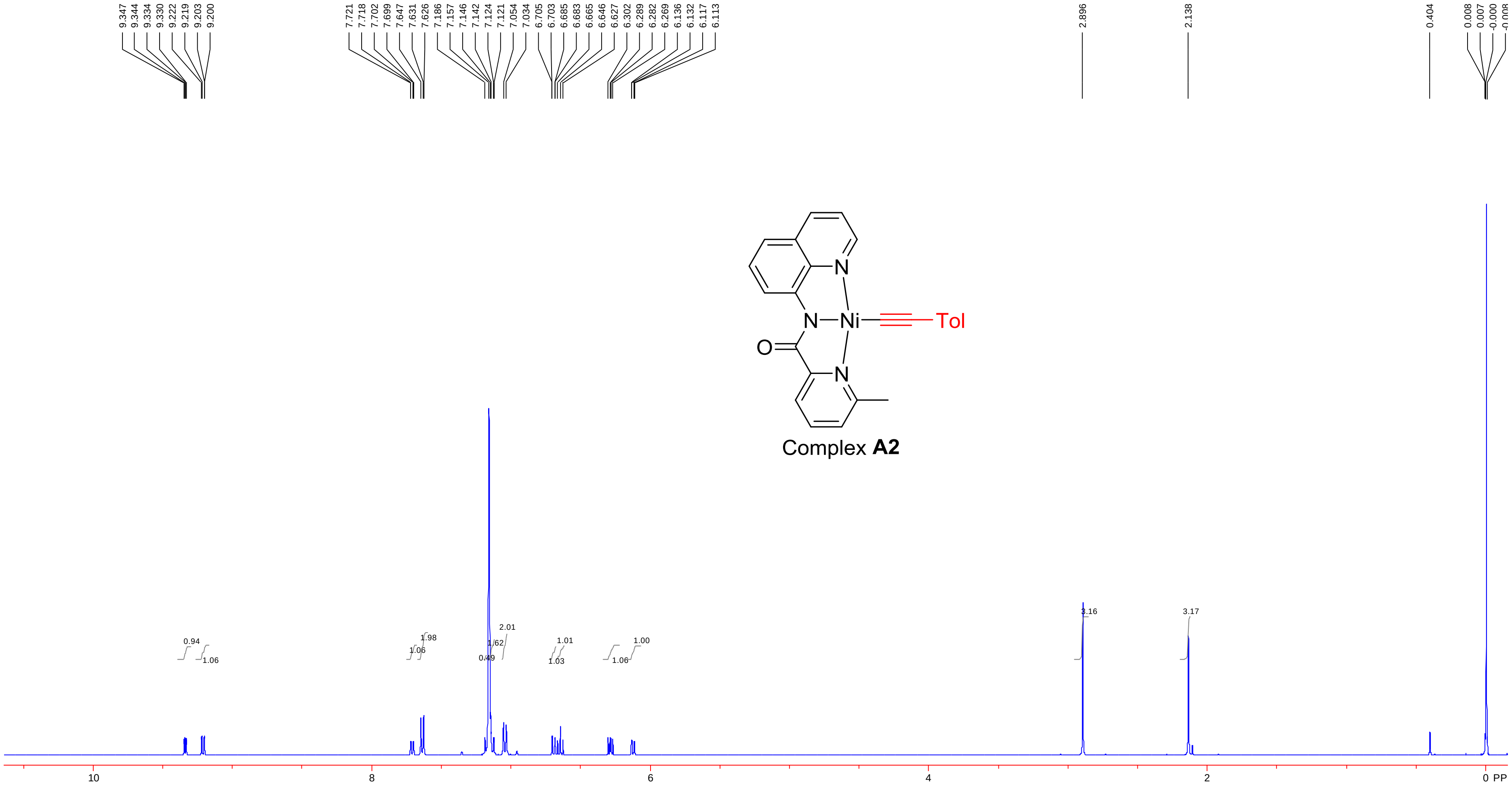

Supplement: Supplementary file 1 — Supplementary Information [file 41467_2021_25222_MOESM1_ESM.pdf]
